# Supplementary material for: Enantioselective functionalization of unactivated C(sp3)–H bonds through copper-catalyzed diyne cyclization by kinetic resolution
Source: Nat Commun. 2024 Mar 12;15:2232. doi: 10.1038/s41467-024-46288-7 (PMC10933314; doi:10.1038/s41467-024-46288-7)
Supplement: Supplementary file 1 — Supplementary Information [file 41467_2024_46288_MOESM1_ESM.pdf]

Supplementary Information

Enantioselective functionalization of unactivated C(sp^3)–H bonds through copper-catalyzed diyne cyclization by kinetic resolution

Yang-Bo Chen,[1] Li-Gao Liu,[1] Zhe-Qi Wang,[1] Rong Chang,[1] Xin Lu[*,1] Bo Zhou[1] and Long-Wu Ye[*,1,2]

[1]State Key Laboratory of Physical Chemistry of Solid Surfaces, Key Laboratory of Chemical Biology of Fujian Province, and College of Chemistry and Chemical Engineering, Xiamen University, Xiamen 361005, China.

[2]State Key Laboratory of Organometallic Chemistry, Shanghai Institute of Organic Chemistry, Chinese Academy of Sciences, Shanghai 200032, China.

*Email: longwuye@xmu.edu.cn; xinlu@xmu.edu.cn

Content	Page Number
1. General Information	2
2. More Substrate Scope and Mechanism Studies	3
3. Preparation of Starting Materials	9
4. General Procedure for the Copper-Catalyzed Kinetic Resolution	46
5. Synthetic Transformations	101
6. Crystal Data	109
7. Computational Studies	112
8. HPLC Chromatograms	114
9. NMR Spectra	208
10. References	328

1. General Information

Ethyl acetate (ACS grade), hexanes (ACS grade), anhydrous *m*xylene (ACS grade) and toluene (ACS grade) were obtained commercially and used without further purification. Methylene chloride, tetrahydrofuran and diethyl ether were purified according to standard methods unless otherwise noted. Commercially available reagents were used without further purification. Reactions were monitored by thin layer chromatography (TLC) using silicycle pre-coated silica gel plates. Flash column chromatography was performed over silica gel (300-400 mesh). Infrared spectra were recorded on a Nicolet iS50 FTIR Spectrometer as thin film and are reported in reciprocal centimeter (cm^{-1}). Mass spectra were recorded with Agilent 6230 ESI-TOF mass spectrometer.

^{1}H NMR spectra and ^{13}C NMR spectra were recorded on a Bruker AV-400 spectrometer, a Bruker AV-500 spectrometer or a Bruker AV-600 spectrometer in chloroform-d_3. For ^{1}H NMR spectra, chemical shifts are reported in ppm with the internal TMS signal at 0.0 ppm as a standard. For ^{13}C NMR spectra, chemical shifts are reported in ppm with the internal chloroform signal at 77.0 ppm as a standard. The data is being reported as (s = singlet, d = doublet, t = triplet, q = quartet, m = multiplet or unresolved, coupling constant(s) in Hz, integration).

Enantiomeric excesses (ee) were determined by an UltiMate 3000 chiral HPLC. The chiral columns used for the determination of enantiomeric excesses by chiral HPLC were Chiralpak columns (IA, IB, IC, IE, IF, IG, AD-H, AS-H, OD-H). The particle size is 5 μm and dimensions is 4.6 mm I.D * 250 mm L.

2. More Substrate Scope and Mechanism Studies

2.1 More Substrate Scope Studies

Our attempts to extend the reaction to the following diynes **1ad**, **1ae, 1at**–**1aw** under the optimal reaction conditions were unsuccessful (Supplementary Figure 1).

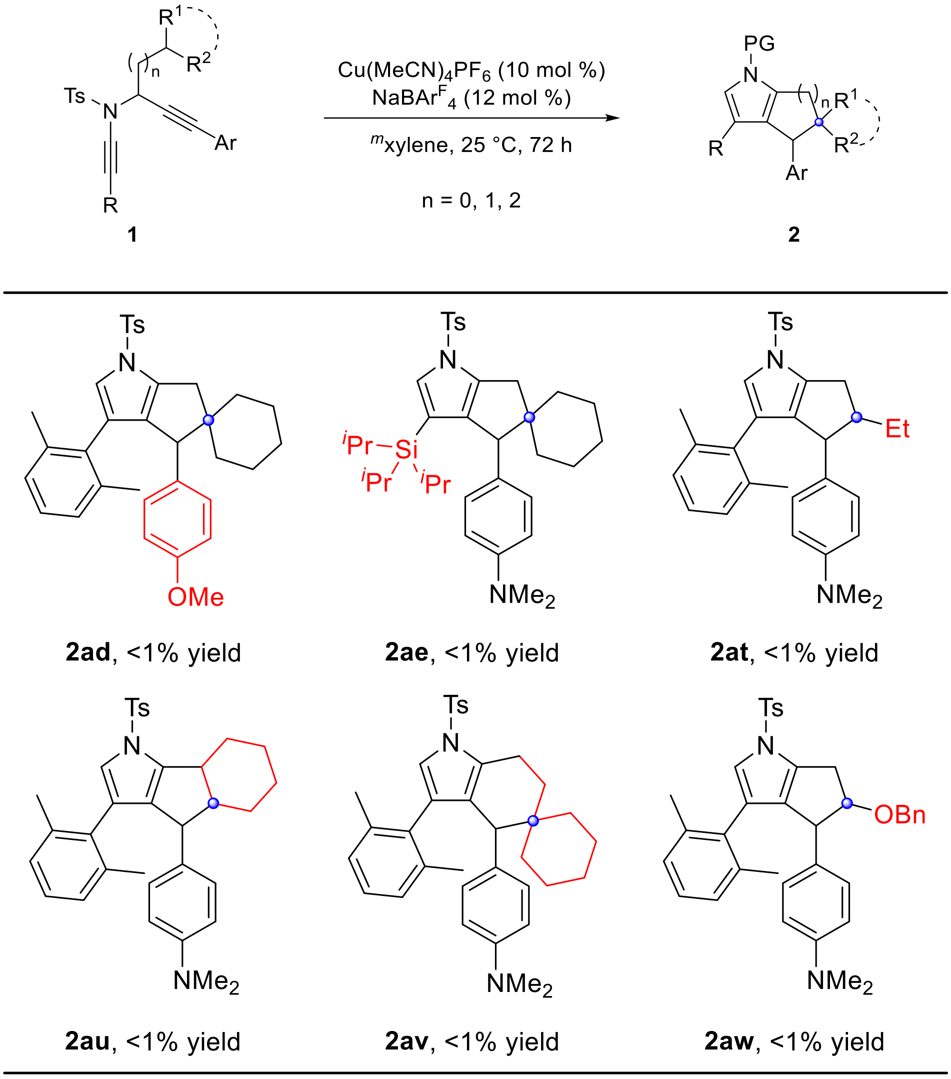

Reaction conditions: **1** (0.1 mmol), $Cu(MeCN)_4PF_6$ (0.01 mmol), $NaBArF_4$ (0.012 mmol), ^{m}xylene (2 mL), 25 °C, 72 h, in Schlenk tubes.

Supplementary Figure 1. The reaction of other diynes under the optimal conditions.

2.2 Mechanism Studies

2.2.1 The reaction of **(±)-[D]*-1a** under the standard conditions

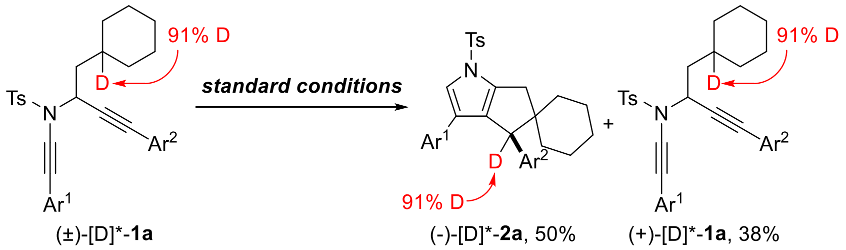

The reaction of (±)-**[D]*-1a** under the standard reaction conditions resulted in (-)-**[D]*-2a** in 50% yield and (+)-**[D]*-1a** in 38% yield. The deuterium atom was completely retained in both product (-)-**[D]*-2a** and recovered substrate (+)-**[D]*-1a** (Supplementary Figure 2).

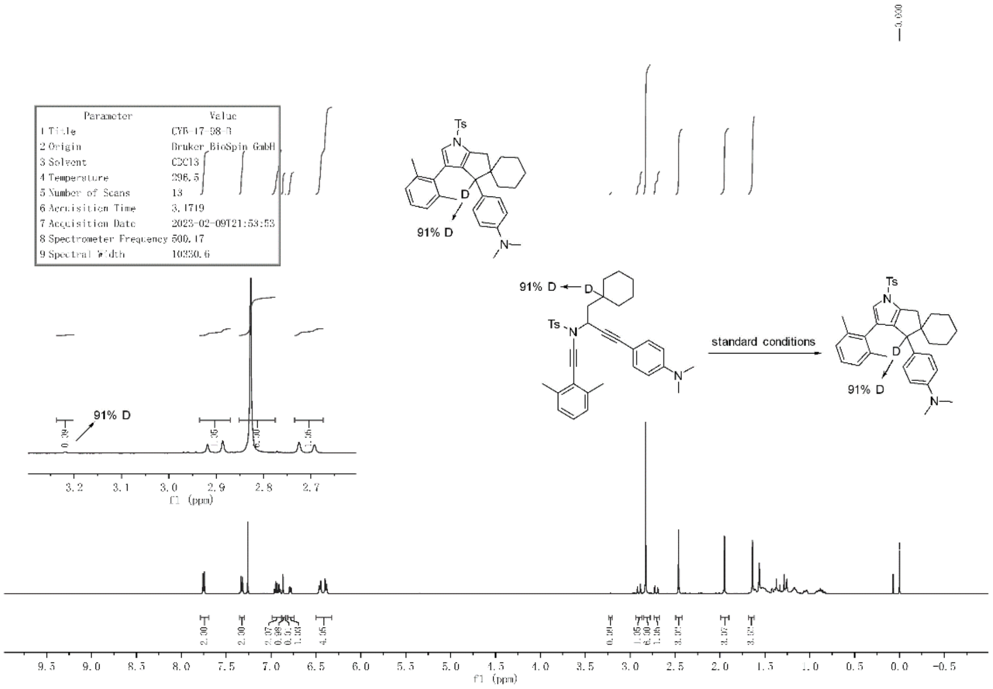

Supplementary Figure 2. ^{1}H NMR of compound **(-)-[D]*-2a**

2.2.2 Hydrogen/deuterium exchange experiments of **(±)-1a** and **(±)-[D]-1a**

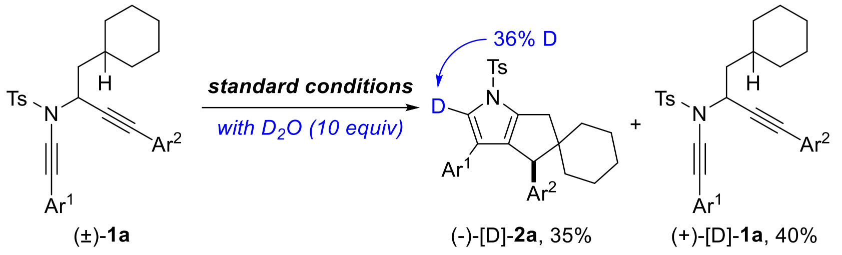

(±)**-1a** was subjected to the standard reaction conditions with 10 equiv of D_2O as addictive. It was found that the product (-)**-[D]-2a** could be obtained in 35% yield, with significant deuterium incorporation into the pyrrole ring (Supplementary Figure 3).

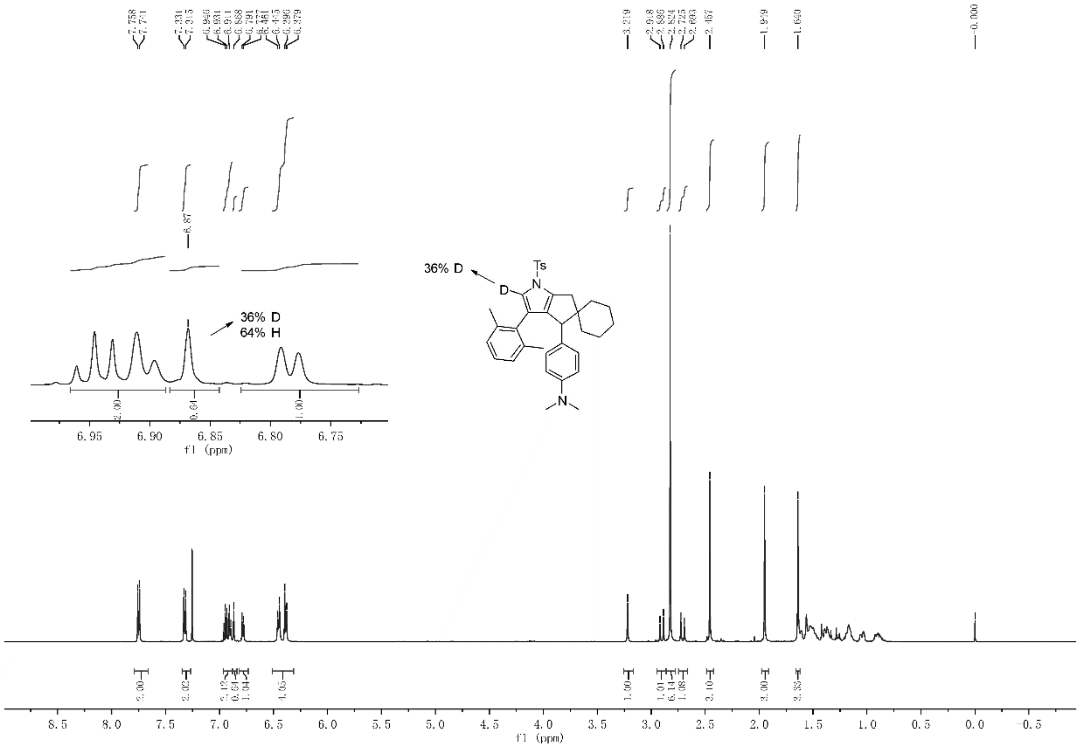

Supplementary Figure 3. ^{1}H NMR of compound **(-)-[D]-2a**

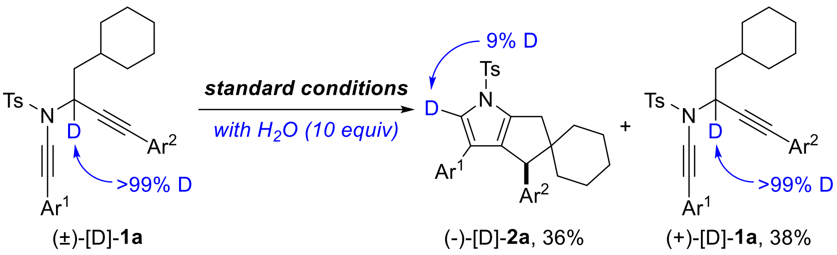

Meanwhile, the deuterium-labeled substrate (±)-**[D]-1a** was also subjected to the standard reaction conditions with 10 equiv of H_2O as addictive, but only 9% deuterium was observed in the product (Supplementary Figure 4). These findings are in agreement with our previous work[3].

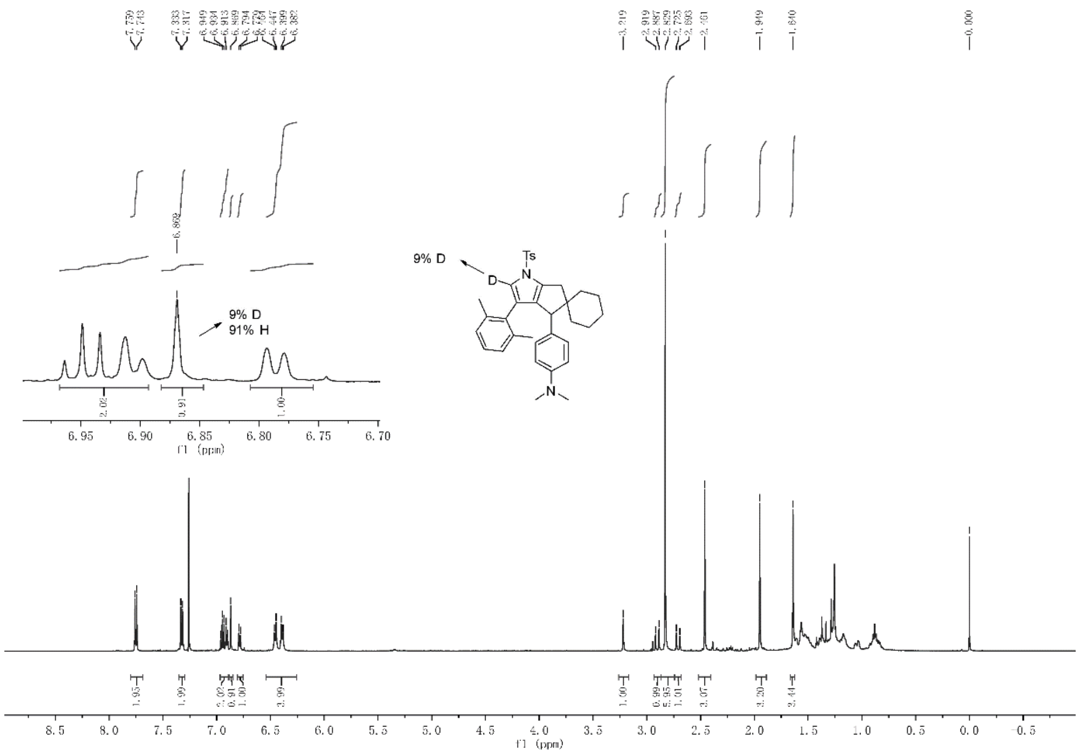

Supplementary Figure 4. ^{1}H NMR of compound **(-)-[D]-2a**

2.2.3 KIE Experiments

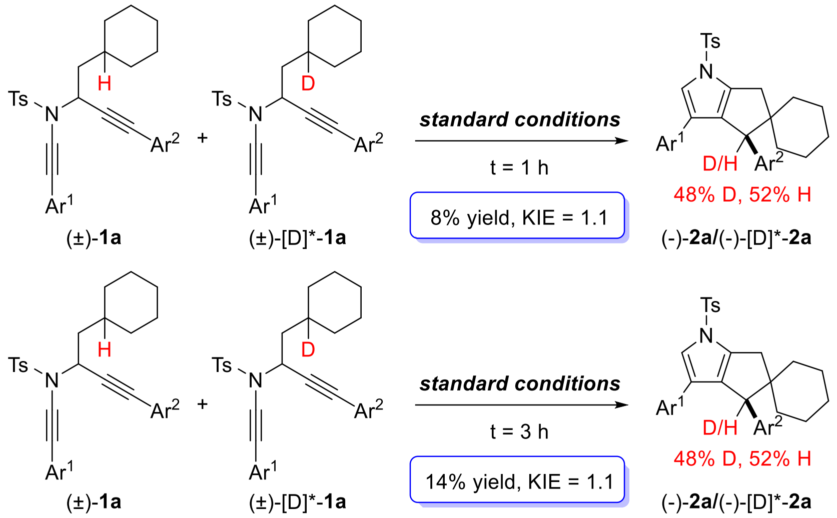

Supplementary Figure 5. KIE Experiments

The competitive reaction was performed with **(±)-1a** (24.9 mg, 0.045 mmol), **(±)-[D]*-1a** (30.5 mg, 0.055 mmol, 91% D), $Cu(MeCN)_4PF_6$ (3.7 mg, 0.01 mmol), **L10** (8.7 mg, 0.012 mmol), $NaBAr^F{}_4$ (10.6 mg, 0.012 mmol) and ^{m}xylene (2 mL) following the general procedure. After corresponding reaction time, the reaction was analyzed by crude ^1H NMR. The results (KIE = 1.1) suggest that the C(sp^3)–H bond cleavage is not involved in the rate-determining step (Supplementary Figure 6 and 7).

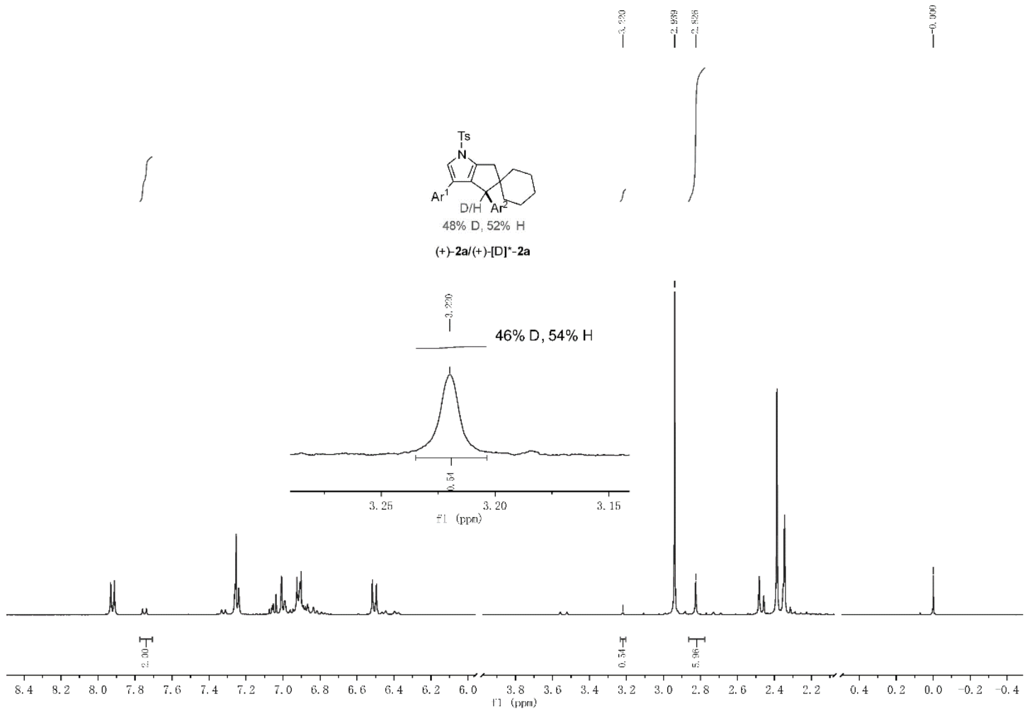

Supplementary Figure 6. Crude ^1H NMR for the competitive reaction after 1 h

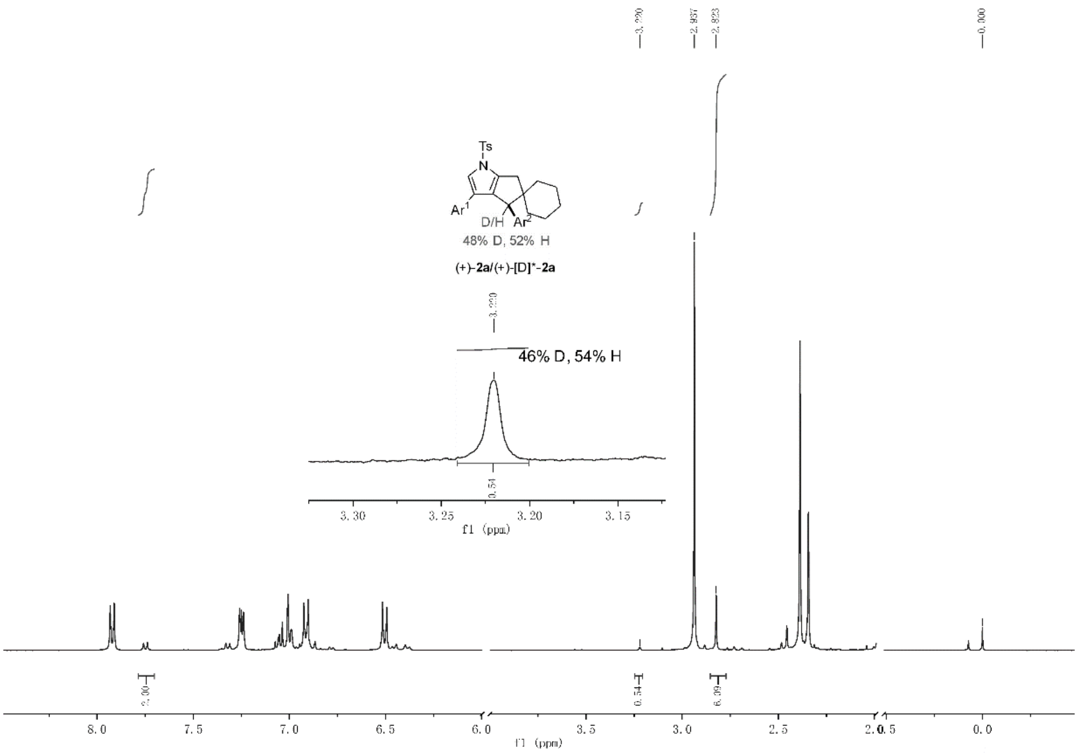

Supplementary Figure 7. Crude ^{1}H NMR for the competitive reaction after 3 h

3. Preparation of Starting Materials

***N*-propargyl ynamides 1 were prepared according to the following procedure**[1–3].

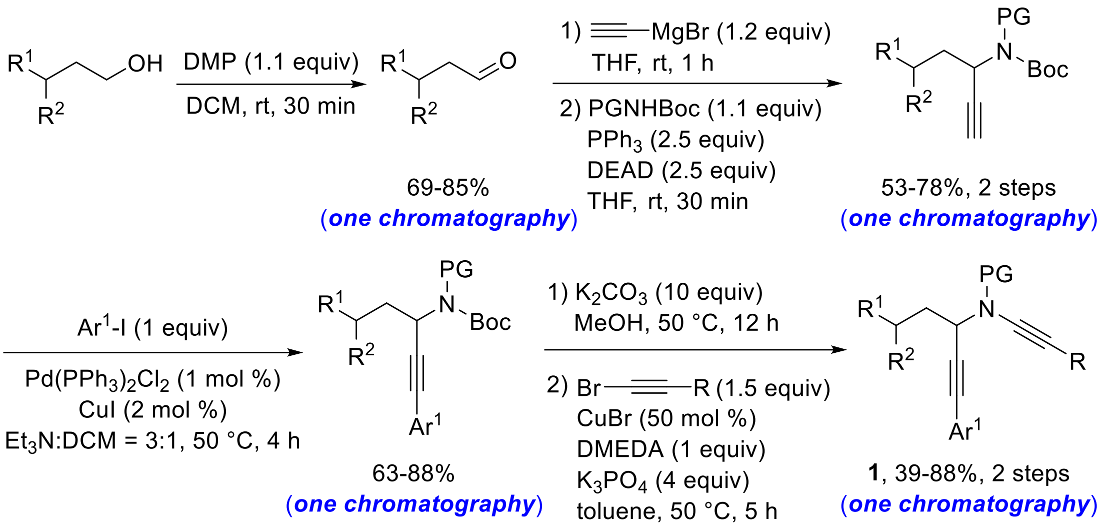

Supplementary Figure 8. General procedure for the synthesis of substrate **1**

To the solution of the substituted alcohol[1] (10 mmol) in DCM (50 mL) was added Dess-Martin Periodinane (11 mmol, 4.66 g), and the reaction was stirred at room temperature for 30 min. The progress of the reaction was monitored by TLC. Upon completion, the reaction was quenched with aqueous $NaHCO_3$ and extracted with DCM (3 x 50 mL). The combined organic layers was dried over anhydrous $MgSO_4$ and concentrated under vacuum. The crude product was purified by column chromatography on silica gel (eluent: hexanes/EtOAc) to afford the desired substituted aldehyde (69–85% yield).

To a dry Schlenk tube were added the substituted aldehyde (5 mmol) and freshly distilled THF (20 mL) under nitrogen atmosphere. Next, ethynyl magnesium bromide (0.5 M, 6 mmol, 12 mL) was added slowly at room temperature and stirred at this temperature for 1 h. The progress of the reaction was monitored by TLC. Upon completion, the reaction was quenched with aqueous NH_4Cl. The resulting mixture was extracted with DCM (3 x 50 mL). The combined organic layers was dried over anhydrous $MgSO_4$ and concentrated under vacuum. The crude propargyl alcohol was directly used in the next step without further purification.

To the solution of crude propargyl alcohol (5 mmol), PGNHBoc (5.5 mmol) and PPh_3 (12.5 mmol, 3.28 g) in THF (30 mL) was added DEAD (12.5 mmol, 2.0 mL) dropwise at room temperature and stirred for additional 30 min. The progress of the reaction was

monitored by TLC. Upon completion, the solution was concentrated under reduced pressure. The residue was purified by column chromatography on silica gel (eluent: hexanes/EtOAc) to give the desired product (53–78% yield, 2 steps).

To a solution of the above product (3 mmol) in DCM (3 mL) and Et_3N (9 mL) were added $Pd(PPh_3)_2Cl_2$ (0.03 mmol, 21.1 mg), CuI (0.06 mmol, 11.4 mg) and the corresponding aryl iodide derivative (3 mmol). The reaction was stirred at 50 °C for 5 h and the progress of the reaction was monitored by TLC. Upon completion, the mixture was concentrated under reduced pressure and purified by column chromatography on silica gel (eluent: hexanes/EtOAc) to give the protected propargylamide (63–88% yield).

To a solution of Boc-protected propargylamide (2 mmol) in MeOH (10 mL) was added K_2CO_3 (20 mmol, 2.76 g). After stirring at 50 °C for 12 h, the mixture was filtered through a pad of silica gel and the filtrate was concentrated under reduce pressure. The obtained crude product was directly used in the next step without further purification.

To a solution of the above propargylamide (1 mmol) in toluene (5 mL) were added copper bromide (0.5 mmol, 71.8 mg), DMEDA (1 mmol, 107 μL), K_3PO_4 (4 mmol, 0.85 g) and corresponding alkynyl bromide (1.5 mmol)[2,3]. The reaction was stirred at room temperature for 5 h and the progress of the reaction was monitored by TLC. Upon completion, the solution was filtered and concentrated under reduced pressure. The residue was purified by column chromatography on silica gel (eluent: hexanes/EtOAc) to give ynamide **1** (39–88% yield, 2 steps).

***N*-(1-cyclohexyl-4-(4-(dimethylamino)phenyl)but-3-yn-2-yl)-*N*-((2,6-dimethylphenyl)ethynyl)-4-methylbenzenesulfonamide (1a)**

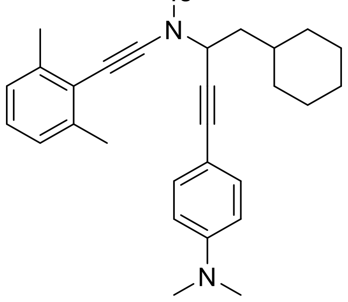

1a

Pale yellow oil (79%, 436.9 mg), (eluent: PE/EtOAc = 8/1). ^{1}H NMR (500 MHz, $CDCl_3$) δ 7.92 (d, *J* = 8.0 Hz, 2H), 7.24 (d, *J* = 8.0 Hz, 2H), 7.06 – 6.98 (m, 3H), 6.91 (d, *J* = 8.5 Hz, 2H), 6.50 (d, *J* = 9.0 Hz, 2H), 5.03 (dd, *J* = 9.0, 6.5 Hz, 1H), 2.92 (s, 6H), 2.39 (s, 6H), 2.34 (s, 3H), 2.00 – 1.89 (m, 2H) , 1.75 – 1.60 (m, 5H) , 1.34 – 1.20 (m, 4H) , 1.04 – 0.91 (m, 2H); ^{13}C NMR (125 MHz, $CDCl_3$) δ 150.0, 144.4, 139.8, 134.8, 132.6, 129.4, 128.2, 127.0, 126.5, 122.9, 111.4, 109.0, 88.1, 86.4, 82.9, 70.4, 51.6, 42.1, 40.1, 33.7, 33.2, 32.4, 26.4, 26.1, 26.0, 21.5, 21.2; IR (neat): 2923, 2851, 2230(s), 1609, 1522, 1447, 1367, 1187, 1169, 1090; HRESIMS Calcd for $[C_{35}H_{40}KN_2O_2S]^+$ (M + K^+) 591.2442, found 591.2443.

***N*-(1-cyclohexyl-4-(4-(dimethylamino)phenyl)but-3-yn-2-yl)-*N*-((2,6-dimethylphenyl)ethynyl)-4-methoxybenzenesulfonamide (1b)**

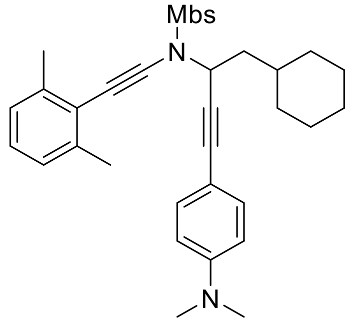

1b

Pale yellow oil (86%, 489.3 mg), (eluent: PE/EtOAc = 5/1). ^{1}H NMR (500 MHz, $CDCl_3$) δ 7.96 (d, *J* = 9.0 Hz, 2H), 7.08 – 6.97 (m, 3H), 6.93 (d, *J* = 8.5 Hz, 2H), 6.88 (d, *J* = 9.0 Hz, 2H), 6.48 (d, *J* = 8.5 Hz, 2H), 5.03 (dd, *J* = 9.0, 6.5 Hz, 1H), 3.71 (s, 3H), 2.90 (s, 6H), 2.40 (s, 6H), 2.02 – 1.86 (m, 2H), 1.81 – 1.59 (m, 6H), 1.35 – 1.14 (m, 3H), 1.05 – 0.90 (m, 2H); ^{13}C NMR (125 MHz, $CDCl_3$) δ 163.5, 149.9, 139.7, 132.5, 130.3, 129.2, 126.9, 126.4, 122.8, 113.9, 111.3, 108.9, 88.2, 86.4, 83.0, 70.3, 55.4, 51.4, 42.0, 40.0, 33.7, 33.2, 32.3, 26.4, 26.0, 25.9, 21.2; IR (neat): 2923, 2850, 2229(s), 1609, 1522, 1446, 1365, 1186, 1162, 1091; HRESIMS Calcd for $[C_{35}H_{40}KN_2O_3S]^+$ (M + K^+) 607.2391, found 607.2380.

***N*-(1-cyclohexyl-4-(4-(dimethylamino)phenyl)but-3-yn-2-yl)-*N*-((2,6-dimethylphenyl)ethynyl)benzenesulfonamide (1c)**

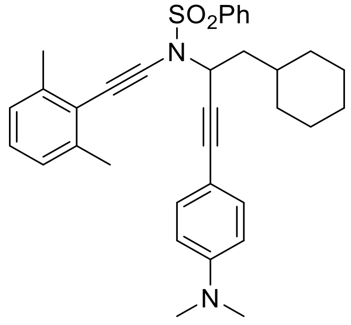

1c

Pale yellow oil (81%, 436.6 mg), (eluent: PE/EtOAc = 8/1). ^{1}H NMR (500 MHz, $CDCl_3$) δ 8.05 (d, *J* = 7.5 Hz, 2H), 7.58 – 7.53 (m, 1H), 7.51 – 7.43 (m, 2H), 7.07 – 6.98 (m, 3H), 6.93 (d, *J* = 9.0 Hz, 2H), 6.50 (d, *J* = 8.5 Hz, 2H), 5.07 (dd, *J* = 9.0, 6.5 Hz, 1H), 2.93 (s, 6H), 2.38 (s, 6H), 2.02 – 1.88 (m, 2H), 1.80 – 1.62 (m, 6H), 1.21 – 1.15 (m, 1H), 1.05 – 0.93 (m, 2H); ^{13}C NMR (125 MHz, $CDCl_3$) δ 150.0, 140.0, 137.8, 133.4, 132.7, 128.8, 128.2, 127.1, 126.5, 122.8, 111.4, 108.9, 87.8, 86.6, 82.9, 70.5, 51.7, 42.2, 40.1, 33.8, 33.3, 32.4, 26.5, 26.1, 26.0, 21.2; IR (neat): 2923, 2851, 2229(s), 1609, 1522, 1448, 1367, 1170, 1144, 1089; HRESIMS Calcd for $[C_{34}H_{38}N_2NaO_2S]^+$ (M + Na^+) 561.2546, found 561.2544.

4-bromo-*N*-(1-cyclohexyl-4-(4-(dimethylamino)phenyl)but-3-yn-2-yl)-*N*-((2,6-dimethylphenyl)ethynyl)benzenesulfonamide (1d)

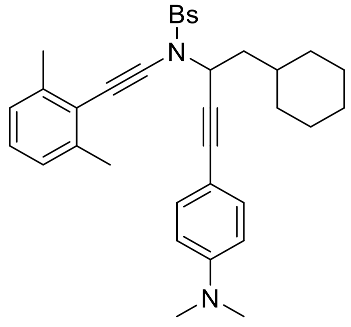

1d

Pale yellow oil (71%, 438.8 mg), (eluent: PE/EtOAc = 8/1). ^{1}H NMR (500 MHz, $CDCl_3$) δ 7.90 (d, *J* = 8.5 Hz, 2H), 7.59 (d, *J* = 9.0 Hz, 2H), 7.10 – 6.98 (m, 3H), 6.90 (d, *J* = 8.5 Hz, 2H), 6.54 (d, *J* = 9.0 Hz, 2H), 5.03 (dd, *J* = 9.0, 6.5 Hz, 1H), 2.94 (s, 6H), 2.39

(s, 6H), 2.03 – 1.88 (m, 2H), 1.79 – 1.61 (m, 6H), 1.39 – 1.22 (m, 3H), 1.04 – 0.92 (m, 2H); ^{13}C NMR (125 MHz, $CDCl_3$) δ 150.1, 140.0, 136.6, 132.6, 132.0, 129.7, 128.8, 127.3, 126.5, 122.5, 111.6, 108.5, 87.5, 87.0, 82.6, 70.6, 51.9, 42.0, 40.1, 33.7, 33.2, 32.3, 26.4, 26.1, 25.9, 21.2; IR (neat): 2923, 2851, 2230(s), 1608, 1522, 1447, 1371, 1172, 1144, 1088; HRESIMS Calcd for $[C_{34}H_{37}BrN_2NaO_2S]^+$ (M + Na^+) 639.1651, found 639.1644.

_N_-(1-cyclohexyl-4-(4-(dimethylamino)phenyl)but-3-yn-2-yl)-_N_-((2,6-dimethylphenyl)ethynyl)methanesulfonamide (1e)

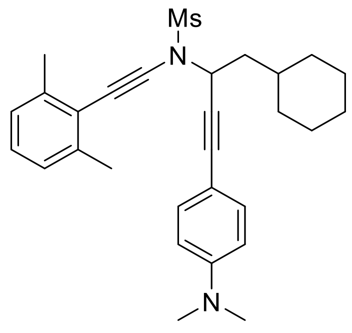

1e

Pale yellow oil (88%, 419.5 mg), (eluent: PE/EtOAc = 8/1). ^{1}H NMR (400 MHz, $CDCl_3$) δ 7.26 (d, *J* = 8.8 Hz, 2H), 7.13 – 6.92 (m, 3H), 6.59 (d, *J* = 8.8 Hz, 2H), 4.99 (dd, *J* = 8.8, 6.4 Hz, 1H), 3.25 (s, 3H), 2.95 (s, 6H), 2.45 (s, 6H), 2.09 – 1.96 (m, 1H), 1.94 – 1.58 (m, 7H), 1.34 – 1.13 (m, 3H), 1.07 – 0.87 (m, 2H); ^{13}C NMR (100 MHz, $CDCl_3$) δ 150.3, 139.7, 132.8, 127.2, 126.6, 122.5, 111.6, 108.4, 87.6, 87.3, 83.2, 70.9, 51.5, 41.8, 40.1, 37.9, 33.8, 33.2, 32.3, 26.4, 26.1, 25.9, 21.2; IR (neat): 2923, 2850, 2230(s), 1552, 1365, 1162; HRESIMS Calcd for $[C_{29}H_{36}N_2NaO_2S]^+$ (M + Na^+) 499.2390, found 499.2397.

_N_-(1-cyclohexyl-4-(4-(dibenzylamino)phenyl)but-3-yn-2-yl)-_N_-((2,6-dimethylphenyl)ethynyl)-4-methylbenzenesulfonamide (1f)

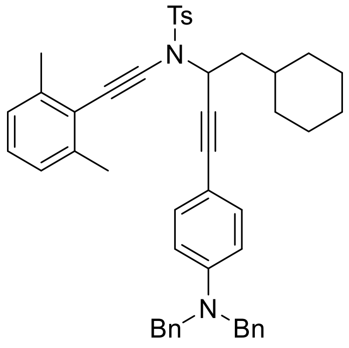

1f

Pale yellow oil (69%, 486.5 mg), (eluent: PE/EtOAc = 8/1). ^{1}H NMR (500 MHz, $CDCl_3$) δ 7.89 (d, *J* = 8.5 Hz, 2H), 7.35 – 7.24 (m, 6H), 7.22 – 7.17 (m, 6H), 7.07 – 7.02 (m, 1H), 7.02 – 6.97 (m, 2H), 6.84 (d, *J* = 8.5 Hz, 2H), 6.53 (d, *J* = 9.0 Hz, 2H), 5.01 (dd, *J* = 9.0, 6.5 Hz, 1H), 4.63 (s, 4H), 2.38 (s, 6H), 2.23 (s, 3H), 2.00 – 1.84 (m, 2H), 1.76 – 1.58 (m, 6H), 1.29 – 1.12 (m, 3H), 1.03 – 0.91 (m, 2H); ^{13}C NMR (125 MHz, $CDCl_3$) δ 148.8, 144.4, 139.7, 137.8, 134.8, 132.8, 129.4, 128.7, 128.2, 127.1, 126.9, 126.5, 126.4, 122.9, 111.7, 109.7, 88.2, 86.2, 83.1, 70.5, 54.1, 51.6, 42.1, 33.8, 33.2, 32.4, 26.5, 26.1, 26.0, 21.5, 21.2; IR (neat): 3061, 2920, 2851, 2228(s), 1607, 1519, 1361, 1168; HRESIMS Calcd for $[C_{47}H_{49}N_2O_2S]^+$ (M + H^+) 705.3509, found 705.3511.

***N*-(1-cyclohexyl-4-(4-(diethylamino)phenyl)but-3-yn-2-yl)-*N*-((2,6-dimethylphenyl)ethynyl)-4-methylbenzenesulfonamide (1g)**

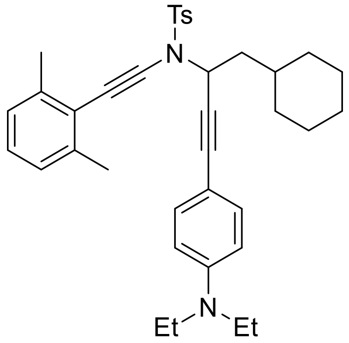

1g

Pale yellow oil (63%, 366.1 mg), (eluent: PE/EtOAc = 8/1). ^{1}H NMR (500 MHz, $CDCl_3$) δ 7.92 (d, *J* = 8.5 Hz, 2H), 7.24 (d, *J* = 7.5 Hz, 2H), 7.09 – 7.02 (m, 1H), 7.01 – 6.96 (m, 2H), 6.88 (d, *J* = 9.0 Hz, 2H), 6.45 (d, *J* = 9.5 Hz, 2H), 5.03 (dd, *J* = 9.0, 6.5 Hz, 1H), 3.31 (q, *J* = 7.0 Hz, 4H), 2.40 (s, 6H), 2.33 (s, 3H), 2.01 – 1.86 (m, 2H), 1.80 –

1.56 (m, 6H), 1.36 – 1.16 (m, 3H), 1.13 (t, *J* = 7.0 Hz, 6H), 1.05 – 0.89 (m, 2H); ^{13}C NMR (125 MHz, $CDCl_3$) δ 147.4, 144.4, 139.8, 134.8, 132.8, 129.4, 128.1, 126.9, 126.5, 122.9, 110.7, 107.8, 88.2, 86.6, 82.6, 51.6, 44.2, 42.1, 33.7, 33.2, 32.4, 26.4, 26.1, 26.0, 21.5, 21.2, 12.4; IR (neat): 3044, 2922, 2851, 2230(s), 1609, 1520, 1361, 1306, 1186, 1090, 942; HRESIMS Calcd for $[C_{37}H_{45}N_2O_2S]^+$ (M + H$^+$) 581.3196, found 581.3191.

***N*-(1-cyclohexyl-4-(4-(piperidin-1-yl)phenyl)but-3-yn-2-yl)-*N*-((2,6-dimethylphenyl)ethynyl)-4-methylbenzenesulfonamide (1h)**

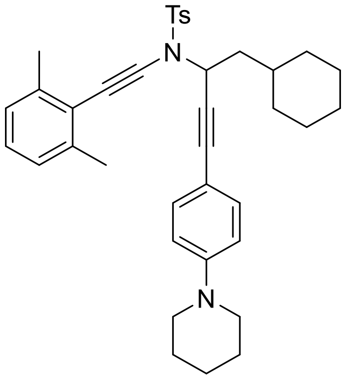

1h

Pale yellow oil (72%, 423.1 mg), (eluent: PE/EtOAc = 8/1). ^{1}H NMR (500 MHz, $CDCl_3$) δ 7.91 (d, *J* = 8.0 Hz, 2H), 7.22 (d, *J* = 8.0 Hz, 2H), 7.07 – 7.01 (m, 1H), 7.01 – 6.95 (m, 2H), 6.91 (d, *J* = 9.0 Hz, 2H), 6.70 (d, *J* = 9.0 Hz, 2H), 5.03 (dd, *J* = 9.0, 6.5 Hz, 1H), 3.22 – 3.05 (m, 4H), 2.39 (s, 6H), 2.31 (s, 3H), 2.03 – 1.86 (m, 2H), 1.77 – 1.55 (m, 12H), 1.32 – 1.12 (m, 3H), 1.04 – 0.91 (m, 2H); ^{13}C NMR (125 MHz, $CDCl_3$) δ 151.4, 144.4, 139.7, 134.7, 132.4, 129.3, 128.1, 126.9, 126.4, 122.8, 114.8, 111.2, 88.0, 86.1, 83.2, 70.4, 51.4, 49.4, 42.0, 33.7, 33.2, 32.3, 26.4, 26.0, 25.9, 25.4, 24.1, 21.5, 21.1; IR (neat): 2924, 2851, 2229(s), 1604, 1514, 1449, 1369, 1237, 1169; HRESIMS Calcd for $[C_{38}H_{44}N_2NaO_2S]^+$ (M + Na$^+$) 615.3016, found 615.3021.

***N*-(1-cyclohexyl-4-(4-morpholinophenyl)but-3-yn-2-yl)-*N*-((2,6-dimethylphenyl)ethynyl)-4-methylbenzenesulfonamide (1i)**

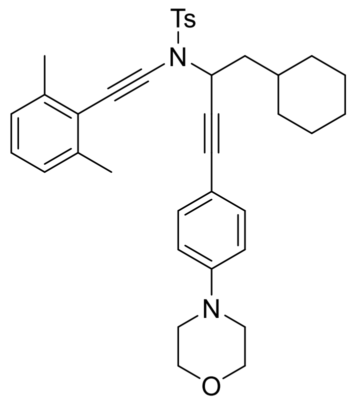

1i

Pale yellow oil (83%, 493.9 mg), (eluent: PE/EtOAc = 8/1). ^{1}H NMR (500 MHz, $CDCl_3$) δ 7.91 (d, *J* = 8.0 Hz, 2H), 7.23 (d, *J* = 8.0 Hz, 2H), 7.06 – 7.02 (m, 1H), 7.01 – 6.97 (m, 2H), 6.95 (d, *J* = 9.0 Hz, 2H), 6.70 (d, *J* = 9.0 Hz, 2H), 5.04 (dd, *J* = 9.0, 6.5 Hz, 1H), 3.82 – 3.77 (m, 4H), 3.14 – 3.07 (m, 4H), 2.39 (s, 6H), 2.32 (s, 3H), 2.01 – 1.87 (m, 2H), 1.83 – 1.49 (m, 7H), 1.26 – 1.12 (m, 2H), 1.04 – 0.90 (m, 2H); ^{13}C NMR (125 MHz, $CDCl_3$) δ 150.7, 144.4, 139.6, 134.6, 132.5, 129.3, 128.0, 126.9, 126.4, 122.7, 114.3, 112.5, 87.9, 85.7, 83.6, 70.3, 66.5, 51.3, 48.2, 41.9, 33.6, 33.1, 32.2, 26.3, 26.0, 25.8, 21.4, 21.1; IR (neat): 2923, 2851, 2230(s), 1606, 1514, 1367, 1168, 1089; HRESIMS Calcd for $[C_{37}H_{42}N_2NaO_3S]^+$ (M + Na$^+$) 617.2808, found 617.2809.

***N*-(1-cyclohexyl-4-(4-(dimethylamino)-2-methylphenyl)but-3-yn-2-yl)-*N*-((2,6-dimethylphenyl)ethynyl)-4-methylbenzenesulfonamide (1j)**

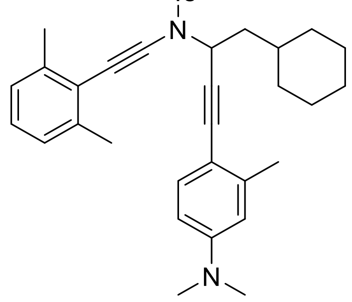

1j

Pale yellow oil (79%, 447.8 mg), (eluent: PE/EtOAc = 8/1). ^{1}H NMR (500 MHz, $CDCl_3$) δ 7.92 (d, *J* = 8.5 Hz, 2H), 7.24 (d, *J* = 9.0 Hz, 2H), 7.07 – 7.02 (m, 1H), 7.01 – 6.97 (m, 2H), 6.86 (d, *J* = 8.5 Hz, 1H), 6.42 – 6.39 (m, 1H), 6.37 (dd, *J* = 8.5, 2.5 Hz, 1H), 5.10 (dd, *J* = 9.0, 7.0 Hz, 1H), 2.92 (s, 6H), 2.37 (s, 6H), 2.33 (s, 3H), 2.08 (s, 3H), 2.01

– 1.52 (m, 10H), 1.21 – 1.14 (m, 1H), 1.06 – 0.92 (m, 2H); ^{13}C NMR (125 MHz, $CDCl_3$) δ 150.2, 144.4, 141.0, 139.7, 135.0, 132.9, 129.4, 128.1, 126.9, 126.5, 122.9, 112.6, 109.4, 109.2, 88.1, 86.7, 85.5, 70.7, 51.7, 42.4, 40.2, 33.9, 33.2, 32.6, 26.5, 26.1, 26.0, 21.5, 21.2, 20.9; IR (neat): 2922, 2851, 2230(s), 1608, 1510, 1471, 1366, 1000, 800; HRESIMS Calcd for $[C_{36}H_{42}N_2NaO_2S]^+$ (M + Na^+) 589.2859, found 589.2866.

_N_-(1-cyclohexyl-4-(4-(dimethylamino)phenyl)but-3-yn-2-yl)-_N_-(mesitylethynyl)-4-methylbenzenesulfonamide (1k)

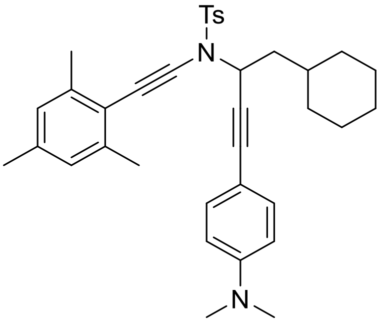

1k

Pale yellow oil (84%, 476.2 mg), (eluent: PE/EtOAc = 8/1). ^{1}H NMR (400 MHz, $CDCl_3$) δ 7.90 (d, J = 8.0 Hz, 2H), 7.18 (d, J = 8.0 Hz, 2H), 6.90 (d, J = 8.8 Hz, 2H), 6.79 (s, 2H), 6.45 (d, J = 8.8 Hz, 2H), 5.04 (dd, J = 8.8, 6.4 Hz, 1H), 2.85 (s, 6H), 2.35 (s, 6H), 2.28 (s, 3H), 2.21 (s, 3H), 2.01 – 1.89 (m, 2H), 1.80 – 1.60 (m, 6H), 1.32 – 1.12 (m, 3H), 1.06 – 0.91 (m, 2H); ^{13}C NMR (100 MHz, $CDCl_3$) δ 149.8, 144.2, 139.6, 136.8, 134.6, 132.4, 129.2, 127.9, 127.3, 119.6, 111.2, 108.7, 87.1, 86.4, 82.8, 70.1, 51.4, 42.0, 39.7, 33.6, 33.1, 32.2, 26.3, 26.0, 25.8, 21.3, 21.0, 20.9; IR (neat): 2922, 2850, 2229(s), 1608, 1522, 1446, 1365, 1167, 1089, 815; HRESIMS Calcd for $[C_{36}H_{42}N_2NaO_2S]^+$ (M + Na^+) 589.2859, found 589.2854.

_N_-(1-cyclohexyl-4-(4-(dimethylamino)phenyl)but-3-yn-2-yl)-_N_-((4-fluoro-2,6-dimethylphenyl)ethynyl)-4-methylbenzenesulfonamide (1l)

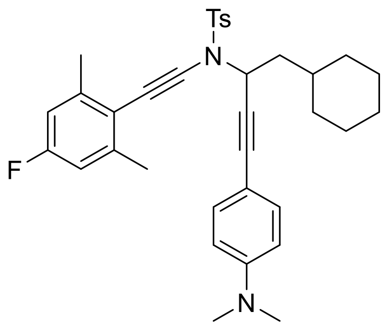

1l

Pale yellow oil (81%, 462.4 mg), (eluent: PE/EtOAc = 8/1). ^{1}H NMR (500 MHz, $CDCl_3$) δ 7.91 (d, *J* = 8.5 Hz, 2H), 7.27 (d, *J* = 7.0 Hz, 2H), 6.91 (d, *J* = 9.0 Hz, 2H), 6.72 (d, *J* = 9.0 Hz, 2H), 6.51 (d, *J* = 9.0 Hz, 2H), 5.02 (dd, *J* = 9.0, 6.5 Hz, 1H), 2.95 (s, 6H), 2.36 (s, 9H), 1.99 – 1.87 (m, 2H), 1.78 – 1.64 (m, 6H), 1.36 – 1.17 (m, 2H), 1.17 – 1.13 (m, 1H), 1.06 – 0.90 (m, 2H); ^{13}C NMR (150 MHz, $CDCl_3$) δ 161.5 (d, *J* = 247.4 Hz), 150.0, 144.5, 142.6 (d, *J* = 8.6 Hz), 134.8, 132.6, 129.4, 128.2, 118.8 (d, *J* = 2.8 Hz), 113.5 (d, *J* = 21.7 Hz), 111.4, 109.0, 87.5, 86.5, 82.9, 69.3, 51.6, 42.2, 40.1, 33.8, 33.3, 32.4, 26.4, 26.1, 26.0, 21.6, 21.3 (d, *J* = 1.3 Hz); ^{19}F NMR (376 MHz, $CDCl_3$) δ -113.6 – -113.9 (m); IR (neat): 2923, 2850, 2229(s), 1609, 1523, 1370, 1165; HRESIMS Calcd for $[C_{35}H_{39}FN_2NaO_2S]^+$ (M + Na^+) 593.2608, found 593.2611.

***N*-((4-chloro-2,6-dimethylphenyl)ethynyl)-*N*-(1-cyclohexyl-4-(4-(dimethylamino)phenyl)but-3-yn-2-yl)-4-methylbenzenesulfonamide (1m)**

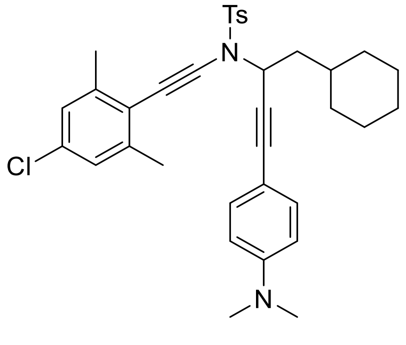

1m

Pale yellow oil (81%, 475.5 mg), (eluent: PE/EtOAc = 8/1). ^{1}H NMR (500 MHz, $CDCl_3$) δ 7.90 (d, *J* = 8.0 Hz, 2H), 7.26 (d, *J* = 8.5 Hz, 2H), 6.99 (s, 2H), 6.91 (d, *J* = 9.0 Hz, 2H), 6.50 (d, *J* = 9.0 Hz, 2H), 5.03 (dd, *J* = 9.0, 6.5 Hz, 1H), 2.93 (s, 6H), 2.35 (s, 3H), 2.34 (s, 6H), 1.97 – 1.85 (m, 2H), 1.81 – 1.54 (m, 6H), 1.35 – 1.23 (m, 2H), 1.22 – 1.10

(m, 1H), 1.05 – 0.91 (m, 2H); ^{13}C NMR (125 MHz, $CDCl_3$) δ 150.0, 144.6, 141.5, 134.7, 132.6, 132.4, 129.4, 128.1, 126.5, 121.5, 111.4, 108.9, 88.8, 86.5, 82.8, 69.6, 51.7, 42.2, 40.1, 33.8, 33.2, 32.4, 26.4, 26.1, 25.9, 21.6, 21.1; IR (neat): 2922, 2851, 2230(s), 1608, 1522, 1362, 1293, 1186, 1019, 815; HRESIMS Calcd for $[C_{35}H_{39}ClN_2NaO_2S]^+$ (M + Na^+) 609.2313, found 609.2319.

***N*-((4-bromo-2,6-dimethylphenyl)ethynyl)-*N*-(1-cyclohexyl-4-(4-(dimethylamino)phenyl)but-3-yn-2-yl)-4-methylbenzenesulfonamide (1n)**

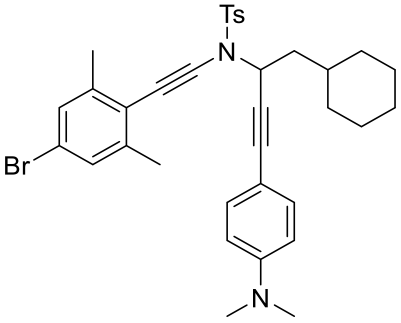

1n

Pale yellow oil (80%, 505.2 mg), (eluent: PE/EtOAc = 8/1). ^{1}H NMR (400 MHz, $CDCl_3$) δ 7.90 (d, *J* = 8.4 Hz, 2H), 7.25 (d, *J* = 8.0 Hz, 2H), 7.15 (s, 2H), 6.91 (d, *J* = 8.4 Hz, 2H), 6.50 (d, *J* = 8.4 Hz, 2H), 5.11 – 4.95 (m, 1H), 2.92 (s, 6H), 2.35 (s, 9H), 2.02 – 1.84 (m, 2H), 1.78 – 1.59 (m, 6H), 1.30 – 1.16 (m, 3H), 1.07 – 0.90 (m, 2H); ^{13}C NMR (100 MHz, $CDCl_3$) δ 150.0, 144.6, 141.6, 134.7, 132.6, 129.4, 129.4, 128.1, 122.0, 120.7, 111.4, 108.8, 89.1, 86.6, 82.8, 69.8, 51.7, 42.2, 40.1, 33.8, 33.2, 32.4, 26.4, 26.1, 25.9, 21.5, 21.0; IR (neat): 2923, 2850, 2229(s), 1609, 1521, 1367, 1315, 1190, 1001, 815; HRESIMS Calcd for $[C_{35}H_{39}BrN_2NaO_2S]^+$ (M + Na^+) 653.1808, found 653.1811.

***N*-(1-cyclohexyl-4-(4-(dimethylamino)phenyl)but-3-yn-2-yl)-*N*-((3,5-dimethyl-[1,1'-biphenyl]-4-yl)ethynyl)-4-methylbenzenesulfonamide (1o)**

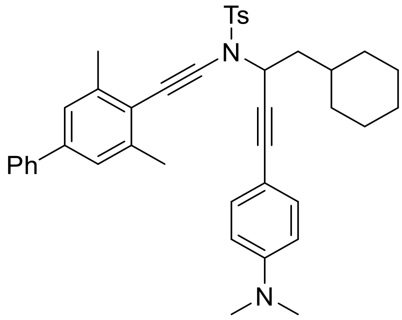

1o

Pale yellow oil (84%, 528.4 mg), (eluent: PE/EtOAc = 8/1). ^{1}H NMR (500 MHz, $CDCl_3$) δ 7.93 (d, *J* = 8.5 Hz, 2H), 7.59 – 7.50 (m, 2H), 7.40 (t, *J* = 7.5 Hz, 2H), 7.34 – 7.28 (m, 1H), 7.26 – 7.20 (m, 4H), 6.92 (d, *J* = 9.0 Hz, 2H), 6.50 (d, *J* = 9.0 Hz, 2H), 5.05 (dd, *J* = 9.0, 6.5 Hz, 1H), 2.92 (s, 6H), 2.45 (s, 6H), 2.34 (s, 3H), 2.03 – 1.88 (m, 2H), 1.81 – 1.59 (m, 6H), 1.33 – 1.23 (m, 2H), 1.21 – 1.12 (m, 1H), 1.06 – 0.90 (m, 2H); ^{13}C NMR (125 MHz, $CDCl_3$) δ 150.0, 144.4, 140.8, 140.2, 139.8, 134.8, 132.6, 129.4, 128.7, 128.2, 127.2, 127.0, 125.3, 122.0, 111.4, 109.0, 88.7, 86.5, 82.9, 70.4, 51.7, 42.2, 40.1, 33.8, 33.3, 32.4, 26.5, 26.1, 26.0, 21.6, 21.4; IR (neat): 2925, 2851, 2231(s), 1596, 1504, 1489, 1443, 1214, 1170, 814; HRESIMS Calcd for $[C_{41}H_{44}N_2NaO_2S]^+$ (M + Na^+) 651.3016, found 651.3021.

***N*-(1-cyclohexyl-4-(4-(dimethylamino)phenyl)but-3-yn-2-yl)-*N*-((2,6-dimethyl-4-((trimethylsilyl)ethynyl)phenyl)ethynyl)-4-methylbenzenesulfonamide (1p)**

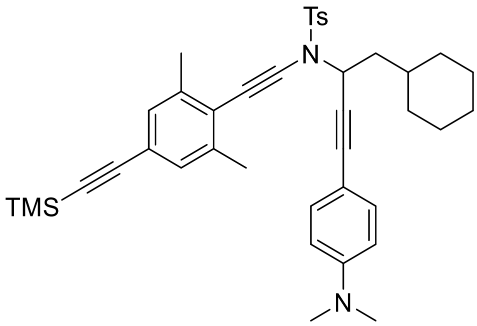

1p

Pale yellow oil (77%, 499.3 mg), (eluent: PE/EtOAc = 8/1). ^{1}H NMR (500 MHz, $CDCl_3$) δ 7.90 (d, *J* = 8.0 Hz, 2H), 7.27 – 7.22 (m, 2H), 7.12 (s, 2H), 6.91 (d, *J* = 8.5 Hz, 2H), 6.49 (d, *J* = 9.0 Hz, 2H), 5.03 (dd, *J* = 9.0, 6.5 Hz, 1H), 2.92 (s, 6H), 2.34 (s, 6H), 2.33 (s, 3H), 1.98 – 1.84 (m, 2H), 1.80 – 1.59 (m, 6H), 1.30 – 1.14 (m, 3H), 1.06 – 0.90 (m,

2H), 0.23 (s, 9H); ^{13}C NMR (125 MHz, $CDCl_3$) δ 150.0, 144.5, 139.4, 134.7, 132.6, 129.9, 129.4, 128.1, 123.5, 121.2, 111.4, 108.8, 105.2, 94.6, 89.8, 86.6, 70.6, 51.7, 42.2, 40.1, 33.7, 33.2, 32.4, 26.4, 26.1, 25.9, 21.5, 20.9, -0.1; IR (neat): 2925, 2851, 2231(s), 1596, 1450, 1371, 1187, 1170, 1109, 816; HRESIMS Calcd for $[C_{40}H_{48}KN_2O_2SSi]^+$ (M + K^+) 687.2837, found 687.2834.

***N*-(1-cyclohexyl-4-(4-(dimethylamino)phenyl)but-3-yn-2-yl)-*N*-((2,6-dimethoxyphenyl)ethynyl)-4-methylbenzenesulfonamide (1q)**

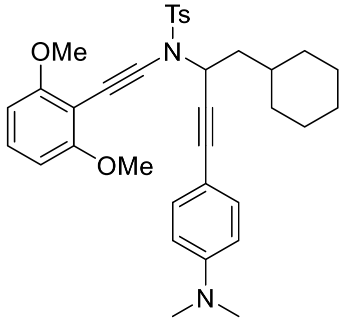

1q

Pale yellow oil (80%, 467.8 mg), (eluent: PE/EtOAc = 4/1). ^{1}H NMR (400 MHz, $CDCl_3$) δ 8.04 (d, *J* = 8.0 Hz, 2H), 7.20 (d, *J* = 8.0 Hz, 2H), 7.12 (t, *J* = 8.4 Hz, 1H), 6.88 (d, *J* = 8.8 Hz, 2H), 6.52 – 6.38 (m, 4H), 5.14 – 4.87 (m, 1H), 3.78 (s, 6H), 2.88 (s, 6H), 2.30 (s, 3H), 2.09 – 1.86 (m, 2H), 1.82 – 1.57 (m, 6H), 1.32 – 1.14 (m, 3H), 1.06 – 0.88 (m, 2H); ^{13}C NMR (100 MHz, $CDCl_3$) δ 161.0, 149.8, 143.9, 135.0, 132.5, 129.0, 128.6, 128.3, 111.2, 109.2, 103.6, 101.8, 87.5, 86.1, 83.0, 65.3, 55.8, 51.2, 41.6, 40.0, 33.5, 33.1, 32.3, 26.4, 26.0, 25.9, 21.4; IR (neat): 2923, 2850, 2242, 1608, 1582, 1474, 1359, 1255, 1112; HRESIMS Calcd for $[C_{35}H_{40}N_2NaO_4S]^+$ (M + Na^+) 607.2601, found 607.2607.

***N*-(1-cyclohexyl-4-(4-(dimethylamino)phenyl)but-3-yn-2-yl)-4-methyl-*N*-(*o*-tolylethynyl)benzenesulfonamide (1r)**

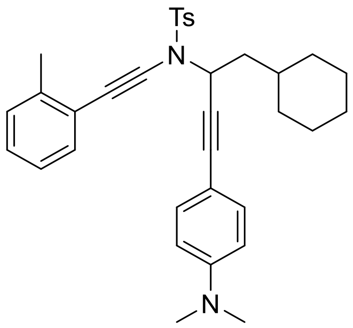

1r

Pale yellow oil (77%, 415.0 mg), (eluent: PE/EtOAc = 8/1). ^{1}H NMR (400 MHz, $CDCl_3$) δ 7.91 (d, *J* = 8.4 Hz, 2H), 7.39 (d, *J* = 7.6 Hz, 1H), 7.26 (d, *J* = 6.0 Hz, 2H), 7.20 – 7.06 (m, 3H), 6.92 (d, *J* = 8.8 Hz, 2H), 6.51 (d, *J* = 8.8 Hz, 2H), 5.01 (dd, *J* = 8.8, 6.4 Hz, 1H), 2.94 (s, 6H), 2.35 (s, 6H), 1.98 – 1.83 (m, 2H), 1.80 – 1.58 (m, 6H), 1.33 – 1.14 (m, 3H), 1.08 – 0.89 (m, 2H); ^{13}C NMR (100 MHz, $CDCl_3$) δ 150.0, 144.4, 140.0, 134.8, 132.6, 131.7, 129.4, 129.2, 128.3, 127.6, 125.4, 122.9, 111.4, 109.1, 86.5, 83.8, 82.9, 71.6, 51.6, 42.2, 40.2, 33.8, 33.2, 32.5, 26.5, 26.1, 26.0, 21.6, 20.9; IR (neat): 2923, 2850, 2229(s), 1608, 1522, 1149, 1367, 1174; HRESIMS Calcd for $[C_{34}H_{38}N_2NaO_2S]^+$ (M + Na^+) 561.2546, found 561.2541.

***N*-(1-cyclohexyl-4-(4-(dimethylamino)phenyl)but-3-yn-2-yl)-*N*-((2-fluorophenyl)ethynyl)-4-methylbenzenesulfonamide (1s)**

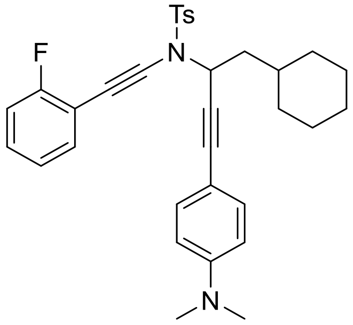

1s

Pale yellow oil (79%, 428.1 mg), (eluent: PE/EtOAc = 8/1). ^{1}H NMR (400 MHz, $CDCl_3$) δ 7.93 (d, *J* = 8.2 Hz, 2H), 7.43 – 7.35 (m, 1H), 7.27 – 7.21 (m, 3H), 7.09 – 7.00 (m, 2H), 6.95 (d, *J* = 8.8 Hz, 2H), 6.51 (d, *J* = 8.8 Hz, 2H), 5.15 – 4.81 (m, 1H), 2.94 (s, 6H), 2.34 (s, 3H), 1.99 – 1.82 (m, 2H), 1.82 – 1.58 (m, 7H), 1.25 – 1.09 (m, 2H), 1.05 – 0.91 (m, 2H); ^{13}C NMR (100 MHz, $CDCl_3$) δ 162.6 (d, *J* = 250.0 Hz), 150.0, 144.5,

134.7, 133.2, 132.7, 129.4, 129.2 (d, J = 8.0 Hz), 128.3, 123.7 (d, J = 4.0 Hz), 115.3 (d, J = 20.0 Hz), 111.8 (d, J = 16.0 Hz), 111.4, 109.1, 86.6, 84.9, 82.7, 66.5, 51.6, 42.0, 40.2, 33.8, 33.2, 32.5, 26.5, 26.1, 26.0, 21.6; ^{19}F NMR (376 MHz, $CDCl_3$) δ -109.5 – -109.9 (m); IR (neat): 2925, 2851, 2230(s), 1610, 1549, 1371, 1166, 817; HRESIMS Calcd for $[C_{33}H_{35}FN_2NaO_2S]^+$ (M + Na^+) 565.2295, found 565.2291.

***N*-((2-chlorophenyl)ethynyl)-*N*-(1-cyclohexyl-4-(4-(dimethylamino)phenyl)but-3-yn-2-yl)-4-methylbenzenesulfonamide (1t)**

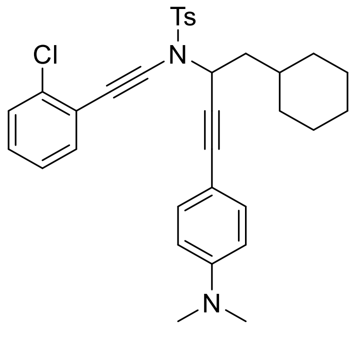

1t

Pale yellow oil (66%, 369.1 mg), (eluent: PE/EtOAc = 8/1). ^{1}H NMR (500 MHz, $CDCl_3$) δ 7.95 (d, J = 8.5 Hz, 2H), 7.47 – 7.39 (m, 1H), 7.36 – 7.31 (m, 1H), 7.25 (d, J = 8.0 Hz, 2H), 7.19 – 7.13 (m, 2H), 6.95 (d, J = 9.0 Hz, 2H), 6.50 (d, J = 9.0 Hz, 2H), 5.01 (dd, J = 9.0, 6.5 Hz, 1H), 2.92 (s, 6H), 2.33 (s, 3H), 2.05 – 1.94 (m, 1H), 1.92 – 1.84 (m, 1H), 1.82 – 1.57 (m, 7H), 1.25 – 1.10 (m, 2H), 1.05 – 0.93 (m, 2H); ^{13}C NMR (125 MHz, $CDCl_3$) δ 150.0, 144.5, 135.3, 134.7, 132.7, 129.4, 129.1, 128.4, 128.3, 126.2, 123.2, 111.4, 109.1, 86.6, 85.2, 82.7, 70.2, 51.7, 42.0, 40.1, 33.8, 33.2, 32.4, 26.5, 26.1, 26.0, 21.6; IR (neat): 2924, 2853, 2233(s), 1605, 1521, 1360, 1294, 1117, 1017; HRESIMS Calcd for $[C_{33}H_{35}ClN_2KO_2S]^+$ (M + K^+) 597.1739, found 597.1728.

***N*-((2-bromophenyl)ethynyl)-*N*-(1-cyclohexyl-4-(4-(dimethylamino)phenyl)but-3-yn-2-yl)-4-methylbenzenesulfonamide (1u)**

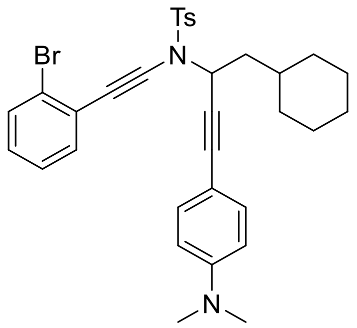

1u

Pale yellow oil (39%, 235.7 mg), (eluent: PE/EtOAc = 8/1). ^{1}H NMR (500 MHz, $CDCl_3$) δ 7.95 (d, *J* = 8.5 Hz, 2H), 7.52 (dd, *J* = 8.0, 1.0 Hz, 1H), 7.41 (dd, *J* = 7.5, 1.5 Hz, 1H), 7.25 (d, *J* = 8.0 Hz, 2H), 7.23 – 7.18 (m, 1H), 7.11 – 7.05 (m, 1H), 6.95 (d, *J* = 9.0 Hz, 2H), 6.50 (d, *J* = 9.0 Hz, 2H), 5.01 (dd, *J* = 9.0, 6.5 Hz, 1H), 2.92 (s, 6H), 2.33 (s, 3H), 2.03 – 1.96 (m, 1H), 1.92 – 1.85 (m, 1H), 1.84 – 1.60 (m, 6H), 1.28 – 1.22 (m, 2H), 1.21 – 1.11 (m, 1H), 1.05 – 0.90 (m, 2H); ^{13}C NMR (125 MHz, $CDCl_3$) δ 150.0, 144.5, 134.7, 132.8, 132.7, 132.2, 129.4, 128.5, 128.3, 126.8, 125.5, 124.8, 111.4, 109.1, 86.6, 84.6, 82.8, 71.9, 51.7, 42.0, 40.1, 33.7, 33.2, 32.4, 26.4, 26.1, 26.0, 21.6; IR (neat): 2923, 2850, 2230(s), 1611, 1519, 1368, 1319, 1190, 1013, 817; HRESIMS Calcd for $[C_{33}H_{35}BrN_2NaO_2S]^+$ (M + Na^+) 625.1495, found 625.1499.

***N*-(1-cyclohexyl-4-(4-(dimethylamino)phenyl)but-3-yn-2-yl)-4-methyl-*N*-(*m*-tolylethynyl)benzenesulfonamide (1v)**

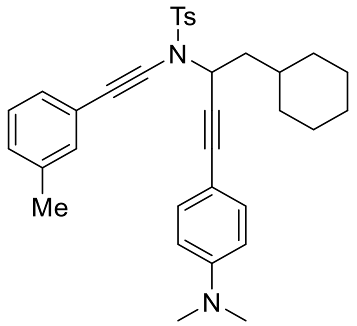

1v

Pale yellow oil (82%, 442.0 mg), (eluent: PE/EtOAc = 8/1). ^{1}H NMR (500 MHz, $CDCl_3$) δ 7.90 (d, *J* = 8.5 Hz, 2H), 7.24 (d, *J* = 8.0 Hz, 2H), 7.21 – 7.12 (m, 3H), 7.07 (d, *J* = 7.5 Hz, 1H), 6.95 (d, *J* = 9.0 Hz, 2H), 6.51 (d, *J* = 9.0 Hz, 2H), 4.98 (dd, *J* = 8.5, 6.5 Hz, 1H), 2.92 (s, 6H), 2.33 (s, 3H), 2.29 (s, 3H), 1.95 – 1.84 (m, 2H), 1.81 – 1.59 (m,

6H), 1.29 – 1.08 (m, 3H), 1.06 – 0.87 (m, 2H); ^{13}C NMR (125 MHz, $CDCl_3$) δ 150.0, 144.4, 137.7, 134.7, 132.6, 132.1, 129.3, 128.6, 128.5, 128.2, 128.0, 122.8, 111.4, 109.1, 86.5, 82.8, 79.7, 72.9, 51.6, 42.1, 40.1, 33.8, 33.1, 32.5, 26.4, 26.1, 26.0, 21.5, 21.1; IR (neat): 2920, 2849, 2228(s), 1605, 1525, 1457, 1366, 1168, 1013, 815; HRESIMS Calcd for $[C_{34}H_{38}N_2NaO_2S]^+$ (M + Na^+) 561.2546, found 561.2544.

***N*-(1-cyclohexyl-4-(4-(dimethylamino)phenyl)but-3-yn-2-yl)-*N*-((3-fluorophenyl)ethynyl)-4-methylbenzenesulfonamide (1w)**

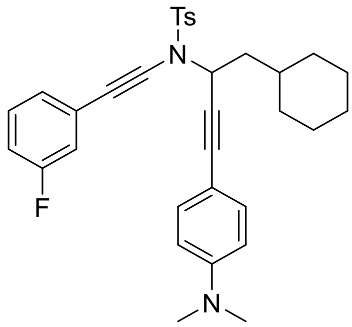

1w

Pale yellow oil (82%, 445.3 mg), (eluent: PE/EtOAc = 8/1). ^{1}H NMR (500 MHz, $CDCl_3$) δ 7.89 (d, *J* = 8.5 Hz, 2H), 7.27 (d, *J* = 8.0 Hz, 2H), 7.23 – 7.19 (m, 1H), 7.16 (d, *J* = 8.0 Hz, 1H), 7.09 – 7.03 (m, 1H), 6.97 – 6.93 (m, 3H), 6.52 (d, *J* = 9.0 Hz, 2H), 4.99 (dd, *J* = 8.5, 6.5 Hz, 1H), 2.93 (s, 6H), 2.35 (s, 3H), 1.92 – 1.84 (m, 2H), 1.80 – 1.50 (m, 7H), 1.26 – 1.10 (m, 2H), 1.05 – 0.89 (m, 2H); ^{13}C NMR (125 MHz, $CDCl_3$) δ 162.3 (d, *J* = 245.0 Hz), 150.1, 144.6, 134.6, 132.6, 129.7 (d, *J* = 8.8 Hz), 129.4, 128.1, 127.2 (d, *J* = 3.0 Hz), 124.9 (d, *J* = 9.7 Hz), 118.0 (d, *J* = 22.7 Hz), 114.8 (d, *J* = 21.2 Hz), 111.4, 108.8, 86.7, 82.6, 81.2, 71.9 (d, *J* = 3.4 Hz), 51.6, 42.2, 40.1, 33.8, 33.1, 32.5, 26.4, 26.1, 25.9, 21.5; ^{19}F NMR (376 MHz, $CDCl_3$) δ -113.2 – -113.4 (m); IR (neat): 2920, 2850, 2230(s), 1609, 1523, 1370, 1165, 1113, 815; HRESIMS Calcd for $[C_{33}H_{35}FN_2NaO_2S]^+$ (M + Na^+) 565.2295, found 565.2288.

***N*-((3-chlorophenyl)ethynyl)-*N*-(1-cyclohexyl-4-(4-(dimethylamino)phenyl)but-3-yn-2-yl)-4-methylbenzenesulfonamide (1x)**

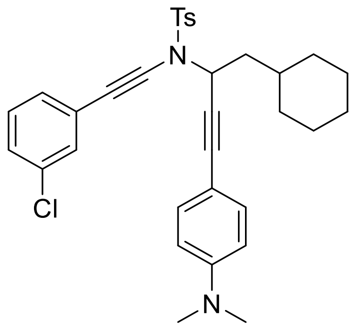

1x

Pale yellow oil (76%, 424.8 mg), (eluent: PE/EtOAc = 8/1). ^{1}H NMR (500 MHz, $CDCl_3$) δ 7.89 (d, *J* = 8.0 Hz, 2H), 7.34 (s, 1H), 7.30 – 7.18 (m, 5H), 6.95 (d, *J* = 9.0 Hz, 2H), 6.51 (d, *J* = 8.5 Hz, 2H), 4.99 (dd, *J* = 8.5, 7.0 Hz, 1H), 2.92 (s, 6H), 2.34 (s, 3H), 1.94 – 1.83 (m, 2H), 1.81 – 1.46 (m, 6H), 1.27 – 1.09 (m, 3H), 1.06 – 0.88 (m, 2H); ^{13}C NMR (125 MHz, $CDCl_3$) δ 150.0, 144.6, 134.6, 133.9, 132.6, 131.0, 129.4, 129.4, 129.4, 128.1, 127.8, 124.8, 111.4, 108.8, 86.7, 82.5, 81.5, 71.7, 51.7, 42.2, 40.0, 33.8, 33.1, 32.5, 26.4, 26.0, 25.9, 21.5; IR (neat): 2922, 2850, 2231(s), 1609, 1520, 1363, 1293, 1186, 1019, 815; HRESIMS Calcd for $[C_{33}H_{35}ClN_2KO_2S]^+$ (M + K^+) 597.1739, found 597.1732.

***N*-((3-bromophenyl)ethynyl)-*N*-(1-cyclohexyl-4-(4-(dimethylamino)phenyl)but-3-yn-2-yl)-4-methylbenzenesulfonamide (1y)**

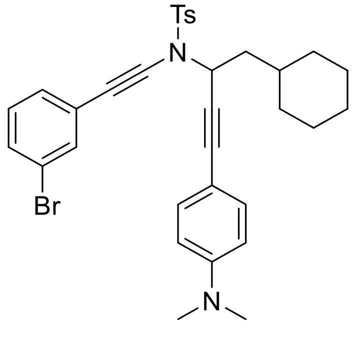

1y

Pale yellow oil (66%, 398.6 mg), (eluent: PE/EtOAc = 8/1). ^{1}H NMR (500 MHz, $CDCl_3$) δ 7.89 (d, *J* = 8.0 Hz, 2H), 7.51 – 7.49 (m, 1H), 7.38 (d, *J* = 8.0 Hz, 1H), 7.32 – 7.25 (m, 3H), 7.16 – 7.11 (m, 1H), 6.96 (d, *J* = 9.0 Hz, 2H), 6.52 (d, *J* = 9.0 Hz, 2H), 4.98 (dd, *J* = 8.5, 6.5 Hz, 1H), 2.93 (s, 6H), 2.35 (s, 3H), 1.93 – 1.83 (m, 2H), 1.80 – 1.58 (m, 6H), 1.27 – 1.10 (m, 3H), 1.07 – 0.90 (m, 2H); ^{13}C NMR (125 MHz, $CDCl_3$) δ

150.1, 144.6, 134.6, 134.0, 132.6, 130.7, 129.9, 129.6, 129.5, 128.1, 125.1, 122.0, 111.5, 108.9, 86.8, 82.6, 81.6, 71.6, 51.7, 42.2, 40.1, 33.8, 33.1, 32.5, 26.4, 26.1, 26.0, 21.6; IR (neat): 2925, 2850, 2230(s), 1608, 1519, 1364, 1315, 1188, 815; HRESIMS Calcd for $[C_{33}H_{35}BrN_2NaO_2S]^+$ (M + Na^+) 625.1495, found 625.1501.

_N_-((4-chlorophenyl)ethynyl)-_N_-(1-cyclohexyl-4-(4-(dimethylamino)phenyl)but-3-yn-2-yl)-4-methylbenzenesulfonamide (1z)

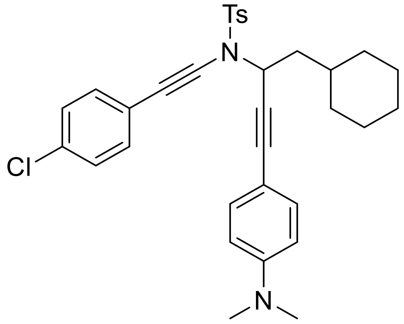

1z

Pale yellow oil (80%, 447.2 mg), (eluent: PE/EtOAc = 8/1). ^{1}H NMR (500 MHz, $CDCl_3$) δ 7.89 (d, J = 8.5 Hz, 2H), 7.31 – 7.22 (m, 6H), 6.94 (d, J = 9.0 Hz, 2H), 6.52 (d, J = 9.0 Hz, 2H), 4.98 (dd, J = 8.5, 6.5 Hz, 1H), 2.94 (s, 6H), 2.35 (s, 3H), 1.94 – 1.82 (m, 2H), 1.78 – 1.56 (m, 6H), 1.28 – 1.09 (m, 3H), 1.05 – 0.91 (m, 2H); ^{13}C NMR (125 MHz, $CDCl_3$) δ 150.1, 144.5, 134.6, 133.6, 132.7, 132.6, 129.4, 128.5, 128.2, 121.6, 111.4, 108.9, 86.7, 82.7, 81.1, 71.8, 51.7, 42.2, 40.1, 33.8, 33.1, 32.5, 26.4, 26.1, 26.0, 21.6; IR (neat): 2922, 2850, 2229(s), 1610, 1521, 1291, 1188, 1014, 817; HRESIMS Calcd for $[C_{33}H_{35}ClKN_2O_2S]^+$ (M + K^+) 597.1739, found 597.1731.

_N_-((4-bromophenyl)ethynyl)-_N_-(1-cyclohexyl-4-(4-(dimethylamino)phenyl)but-3-yn-2-yl)-4-methylbenzenesulfonamide (1aa)

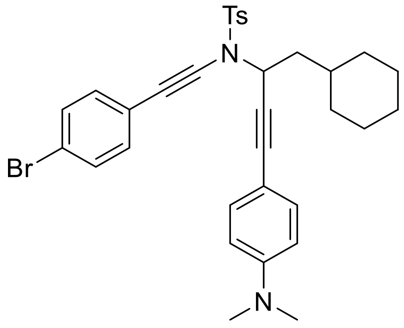

1aa

Pale yellow oil (75%, 453.2 mg), (eluent: PE/EtOAc = 8/1). ^{1}H NMR (500 MHz, $CDCl_3$) δ 7.88 (d, *J* = 8.0 Hz, 2H), 7.39 (d, *J* = 8.5 Hz, 2H), 7.28 – 7.21 (m, 4H), 6.94 (d, *J* = 8.5 Hz, 2H), 6.51 (d, *J* = 8.5 Hz, 2H), 4.98 (dd, *J* = 8.5, 6.5 Hz, 1H), 2.93 (s, 6H), 2.35 (s, 3H), 1.93 – 1.83 (m, 2H), 1.80 – 1.45 (m, 7H), 1.26 – 1.11 (m, 2H), 1.05 – 0.87 (m, 2H); ^{13}C NMR (125 MHz, $CDCl_3$) δ 150.1, 144.6, 134.6, 132.9, 132.6, 131.4, 129.4, 128.1, 122.2, 121.7, 111.4, 108.9, 86.7, 82.6, 81.3, 71.9, 51.7, 42.2, 40.1, 33.8, 33.1, 32.5, 26.4, 26.1, 26.0, 21.6; IR (neat): 2924, 2849, 2228(s), 1610, 1521, 1316, 1190, 815; HRESIMS Calcd for $[C_{33}H_{35}BrN_2NaO_2S]^+$ (M + Na^+) 625.1495, found 625.1499.

***N*-(1-cyclohexyl-4-(4-(dimethylamino)phenyl)but-3-yn-2-yl)-4-methyl-*N*-((4-(trifluoromethyl)phenyl)ethynyl)benzenesulfonamide (1ab)**

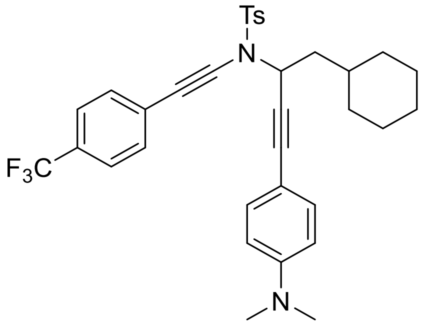

1ab

Pale yellow oil (84%, 498.3 mg), (eluent: PE/EtOAc = 8/1). ^{1}H NMR (500 MHz, $CDCl_3$) δ 7.90 (d, *J* = 8.0 Hz, 2H), 7.52 (d, *J* = 8.5 Hz, 2H), 7.46 (d, *J* = 8.5 Hz, 2H), 7.27 (d, *J* = 8.0 Hz, 2H), 6.95 (d, *J* = 9.0 Hz, 2H), 6.52 (d, *J* = 9.0 Hz, 2H), 5.00 (dd, *J* = 8.5, 6.5 Hz, 1H), 2.94 (s, 6H), 2.35 (s, 3H), 1.96 – 1.82 (m, 2H), 1.79 – 1.57 (m, 6H), 1.31 – 1.11 (m, 3H), 1.06 – 0.87 (m, 2H); ^{13}C NMR (125 MHz, $CDCl_3$) δ 150.1, 144.7, 134.6, 132.6, 131.2, 129.5, 128.2, 126.0 (q, *J* = 252.7 Hz), 125.1 (q, *J* = 3.6 Hz), 111.4, 108.8, 86.8, 82.9, 82.5, 72.2, 51.8, 42.3, 40.1, 33.8, 33.1, 32.5, 26.4, 26.1 26.0, 21.6; ^{19}F NMR (376 MHz, $CDCl_3$) δ -62.7 (s); IR (neat): 2924, 2851, 2224(s), 1609, 1522, 1405, 1187, 1168, 1104, 815; HRESIMS Calcd for $[C_{34}H_{35}F_3N_2NaO_2S]^+$ (M + Na^+) 615.2264, found 615.2261.

***N*-(1-cyclohexyl-4-(4-(dimethylamino)phenyl)but-3-yn-2-yl)-*N*-(hex-1-yn-1-yl)-4-methylbenzenesulfonamide (1ac)**

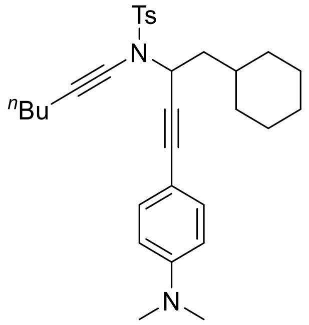

1ac

Pale yellow oil (53%, 267.6 mg), (eluent: PE/EtOAc = 10/1). ^{1}H NMR (500 MHz, $CDCl_3$) δ 7.86 (d, *J* = 8.5 Hz, 2H), 7.24 (d, *J* = 8.0 Hz, 2H), 6.92 (d, *J* = 9.0 Hz, 2H), 6.52 (d, *J* = 9.0 Hz, 2H), 4.89 (dd, *J* = 9.0, 6.5 Hz, 1H), 2.94 (s, 6H), 2.35 (s, 3H), 2.30 (t, *J* = 7.0 Hz, 2H), 1.92 – 1.78 (m, 2H), 1.76 – 1.62 (m, 5H), 1.58 – 1.50 (m, 1H), 1.48 – 1.34 (m, 4H), 1.31 – 1.22 (m, 2H), 1.20 – 1.11 (m, 1H), 1.04 – 0.88 (m, 2H), 0.84 (t, *J* = 7.0 Hz, 3H); ^{13}C NMR (125 MHz, $CDCl_3$) δ 145.0, 144.0, 134.8, 132.6, 129.1, 128.2, 111.4, 109.3, 86.1, 83.1, 72.3, 70.5, 51.0, 41.9, 40.1, 33.7, 33.2, 32.4, 31.0, 26.5, 26.1, 26.0, 21.6, 21.5, 18.2, 13.5; IR (neat): 2925, 2852, 2251, 2220(s), 1609, 1521, 1363, 1187, 1167, 1091, 814; HRESIMS Calcd for $[C_{31}H_{40}N_2NaO_2S]^+$ (M + Na^+) 527.2703, found 527.2692.

***N*-(1-cyclohexyl-4-(4-methoxyphenyl)but-3-yn-2-yl)-*N*-((2,6-dimethylphenyl)ethynyl)-4-methylbenzenesulfonamide (1ad)**

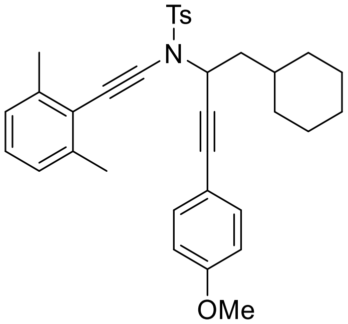

1ad

Pale yellow oil (85%, 459.2 mg), (eluent: PE/EtOAc = 5/1). ^{1}H NMR (500 MHz, $CDCl_3$) δ 7.92 (d, *J* = 8.0 Hz, 2H), 7.24 (d, *J* = 8.0 Hz, 2H), 7.09 – 7.03 (m, 1H), 7.03 – 6.99

(m, 2H), 6.97 (d, J = 8.5 Hz, 2H), 6.73 (d, J = 9.0 Hz, 2H), 5.03 (dd, J = 9.0, 6.5 Hz, 1H), 3.76 (s, 3H), 2.39 (s, 6H), 2.32 (s, 3H), 2.02 – 1.86 (m, 2H), 1.80 – 1.60 (m, 6H), 1.34 – 1.13 (m, 3H), 1.07 – 0.90 (m, 2H); ^{13}C NMR (125 MHz, $CDCl_3$) δ 159.5, 144.5, 139.8, 134.7, 132.9, 129.4, 128.2, 127.1, 126.5, 122.8, 114.2, 113.6, 87.9, 85.3, 83.9, 70.4, 55.2, 51.3, 41.9, 33.7, 33.2, 32.3, 26.4, 26.1, 25.9, 21.5, 21.2; IR (neat): 2930, 2855, 2230(s), 1609, 1520, 1366, 1189, 1165, 1087, 815; HRESIMS Calcd for $[C_{34}H_{37}NNaO_3S]^+$ (M + Na$^+$) 562.2386, found 562.2379.

***N*-(1-cyclohexyl-4-(4-(dimethylamino)phenyl)but-3-yn-2-yl)-4-methyl-*N*-((triisopropylsilyl)ethynyl)benzenesulfonamide (1ae)**

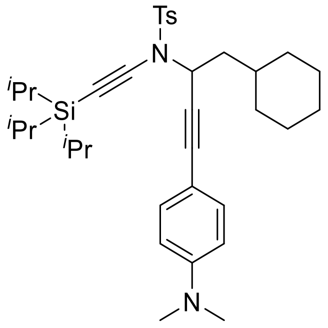

1ae

Pale yellow oil (50%, 302.5 mg), (eluent: PE/EtOAc = 10/1). ^{1}H NMR (600 MHz, $CDCl_3$) δ 7.87 (d, J = 7.8 Hz, 2H), 7.21 (d, J = 7.8 Hz, 2H), 6.90 (d, J = 8.4 Hz, 2H), 6.51 (d, J = 9.0 Hz, 2H), 4.90 (dd, J = 9.6, 6.0 Hz, 1H), 2.95 (s, 6H), 2.33 (s, 3H), 1.94 – 1.81 (m, 2H), 1.75 – 1.62 (m, 5H), 1.58 – 1.50 (m, 1H), 1.34 – 1.22 (m, 3H), 1.19 – 1.12 (m, 1H), 1.04 (s, 18H), 1.03 – 0.79 (m, 4H); ^{13}C NMR (150 MHz, $CDCl_3$) δ 149.9, 144.3, 134.6, 132.6, 129.2, 128.3, 111.4, 109.3, 93.8, 86.3, 82.8, 71.5, 51.1, 41.9, 40.2, 33.6, 33.3, 32.2, 26.5, 26.1, 25.9, 21.5, 18.6(0), 18.5(9), 11.3; IR (neat): 2930, 2855, 2229(s), 1609, 1520, 1365, 1130, 816, 583; HRESIMS Calcd for $[C_{36}H_{52}KN_2O_2SSi]^+$ (M + K$^+$) 643.3150, found 643.3158.

***N*-(1-cyclopentyl-4-(4-(dimethylamino)phenyl)but-3-yn-2-yl)-*N*-((2,6-dimethylphenyl)ethynyl)-4-methylbenzenesulfonamide (1af)**

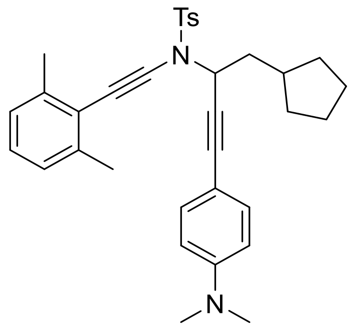

1af

Pale yellow oil (88%, 474.3 mg), (eluent: PE/EtOAc = 8/1). ^{1}H NMR (500 MHz, $CDCl_3$) δ 7.92 (d, *J* = 8.5 Hz, 2H), 7.24 (d, *J* = 8.0 Hz, 2H), 7.07 – 6.98 (m, 3H), 6.91 (d, *J* = 9.0 Hz, 2H), 6.50 (d, *J* = 9.0 Hz, 2H), 4.95 (dd, *J* = 8.5, 6.0 Hz, 1H), 2.93 (s, 6H), 2.39 (s, 6H), 2.34 (s, 3H), 2.16 – 2.05 (m, 2H), 1.99 – 1.83 (m, 3H), 1.67 – 1.54 (m, 4H), 1.23 – 1.13 (m, 2H); ^{13}C NMR (125 MHz, $CDCl_3$) δ 150.0, 144.4, 139.8, 134.8, 132.6, 129.4, 128.1, 127.0, 126.5, 122.9, 111.4, 109.0, 88.2, 86.5, 82.9, 70.4, 53.3, 41.0, 40.1, 36.5, 32.5, 32.1, 25.1, 25.0, 21.5, 21.2; IR (neat): 2948, 2864, 2229(s), 1609, 1521,1468, 1367, 1226, 1170, 1069; HRESIMS Calcd for $[C_{34}H_{38}N_2NaO_2S]^+$ (M + Na^+) 561.2546, found 561.2551.

***N*-(1-cycloheptyl-4-(4-(dimethylamino)phenyl)but-3-yn-2-yl)-*N*-((2,6-dimethylphenyl)ethynyl)-4-methylbenzenesulfonamide (1ag)**

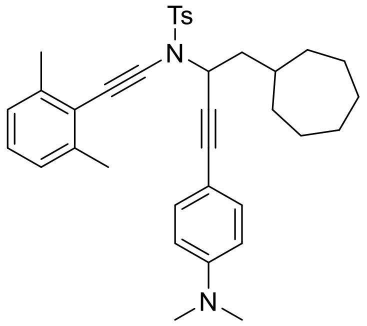

1ag

Pale yellow oil (80%, 453.4 mg), (eluent: PE/EtOAc = 8/1). ^{1}H NMR (500 MHz, $CDCl_3$) δ 7.92 (d, *J* = 8.5 Hz, 2H), 7.24 (d, *J* = 8.0 Hz, 2H), 7.10 – 6.96 (m, 3H), 6.91 (d, *J* = 9.0 Hz, 2H), 6.50 (d, *J* = 9.0 Hz, 2H), 5.00 (dd, *J* = 9.0, 6.0 Hz, 1H), 2.93 (s, 6H), 2.39 (s, 6H), 2.34 (s, 3H), 2.04 – 1.93 (m, 1H), 1.90 – 1.78 (m, 3H), 1.74 – 1.54 (m, 5H), 1.52 – 1.43 (m, 4H), 1.33 – 1.22 (m, 2H); ^{13}C NMR (125MHz, $CDCl_3$) δ 150.0, 144.4,

139.8, 134.8, 132.6, 129.4, 128.1, 127.0, 126.5, 122.9, 111.4, 109.0, 88.1, 86.5, 82.9, 70.3, 52.2, 42.6, 40.1, 35.1, 34.6, 33.6, 28.6, 28.5, 26.2, 26.0, 21.5, 21.2; IR (neat): 2921, 2853, 2231(s), 1609, 1522, 1461, 1445, 1364, 1187, 1168; HRESIMS Calcd for $[C_{36}H_{42}N_2NaO_2S]^+$ (M + Na^+) 589.2859, found 589.2851.

N-(1-cyclooctyl-4-(4-(dimethylamino)phenyl)but-3-yn-2-yl)-N-((2,6-dimethylphenyl)ethynyl)-4-methylbenzenesulfonamide (1ah)

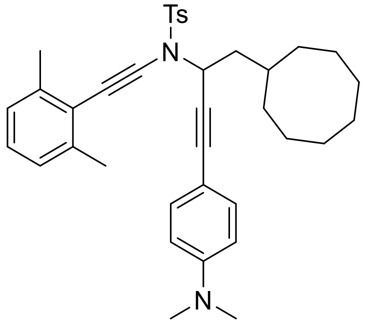

1ah

Pale yellow oil (82%, 476.3 mg), (eluent: PE/EtOAc = 8/1). ^1H NMR (500 MHz, $CDCl_3$) δ 7.93 (d, *J* = 8.5 Hz, 2H), 7.25 (d, *J* = 8.0 Hz, 2H), 7.08 – 7.02 (m, 1H), 6.99 (d, *J* = 7.5 Hz, 2H), 6.91 (d, *J* = 9.0 Hz, 2H), 6.50 (d, *J* = 8.5 Hz, 2H), 5.01 (dd, *J* = 9.0, 6.5 Hz, 1H), 2.93 (s, 6H), 2.38 (s, 6H), 2.34 (s, 3H), 1.99 – 1.74 (m, 4H), 1.71 – 1.47 (m, 11H), 1.41 – 1.31 (m, 2H); ^{13}C NMR (125 MHz, $CDCl_3$) δ 150.0, 144.4, 139.9, 134.9, 132.6, 129.4, 128.2, 127.0, 126.5, 122.9, 111.4, 109.1, 88.2, 86.5, 82.9, 70.3, 52.2, 42.4, 40.1, 33.3, 32.2, 31.1, 27.3(4), 27.2(5), 26.3, 25.1, 21.6, 21.2; IR (neat): 2918, 2853, 2229(s), 1608, 1522, 1468, 1445, 1364, 1187, 1168; HRESIMS Calcd for $[C_{37}H_{44}N_2NaO_2S]^+$ (M + Na^+) 603.3016, found 603.3024.

N-(1-cyclododecyl-4-(4-(dimethylamino)phenyl)but-3-yn-2-yl)-N-((2,6-dimethylphenyl)ethynyl)-4-methylbenzenesulfonamide (1ai)

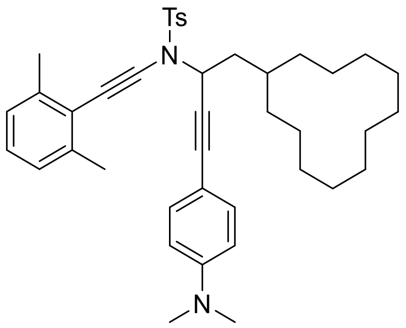

1ai

Pale yellow oil (73%, 465.3 mg), (eluent: PE/EtOAc = 8/1). ^{1}H NMR (400 MHz, $CDCl_3$) δ 7.93 (d, *J* = 8.0 Hz, 2H), 7.26 (d, *J* = 8.0 Hz, 2H), 7.08 – 6.97 (m, 3H), 6.92 (d, *J* = 8.8 Hz, 2H), 6.51 (d, *J* = 8.8 Hz, 2H), 5.02 (dd, *J* = 8.4, 6.4 Hz, 1H), 2.94 (s, 6H), 2.38 (s, 6H), 2.36 (s, 3H), 1.92 – 1.82 (m, 2H), 1.45 – 1.26 (m, 23H); ^{13}C NMR (100 MHz, $CDCl_3$) δ 150.0, 144.4, 139.9, 134.9, 132.6, 129.4, 128.2, 126.9, 126.5, 122.9, 111.4, 109.1, 88.2, 86.6, 82.9, 77.3, 70.4, 52.3, 40.2, 39.8, 30.4, 29.0, 28.5, 24.6(4), 24.6(2), 24.1, 23.4, 23.3(1), 23.2(7), 23.1, 21.7, 21.6, 21.4, 21.2; IR (neat): 2930, 2859, 2229(s), 1608, 1522, 1470, 1445, 1363, 1186, 1168; HRESIMS Calcd for $[C_{41}H_{52}N_2NaO_2S]^+$ (M + Na^+) 659.3642, found 659.3647.

***N*-(1-cyclopentadecyl-4-(4-(dimethylamino)phenyl)but-3-yn-2-yl)-*N*-((2,6-dimethylphenyl)ethynyl)-4-methylbenzenesulfonamide (1aj)**

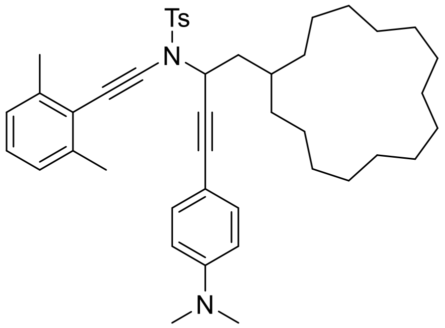

1aj

Pale yellow oil (79%, 536.4 mg), (eluent: PE/EtOAc = 8/1). ^{1}H NMR (400 MHz, $CDCl_3$) δ 7.92 (d, *J* = 8.4 Hz, 2H), 7.24 (d, *J* = 8.0 Hz, 2H), 7.09 – 6.95 (m, 3H), 6.92 (d, *J* = 9.2 Hz, 2H), 6.50 (d, *J* = 8.8 Hz, 2H), 5.01 (dd, *J* = 8.4, 7.2 Hz, 1H), 2.92 (s, 6H), 2.39 (s, 6H), 2.34 (s, 3H), 2.00 – 1.80 (m, 2H), 1.79 – 1.64 (m, 1H), 1.41 – 1.25 (m, 28H); ^{13}C NMR (100 MHz, $CDCl_3$) δ 150.0, 144.4, 139.8, 134.9, 132.6, 129.4, 128.2, 126.9,

126.4, 122.9, 111.4, 109.1, 88.2, 86.6, 82.9, 70.4, 52.3, 40.1, 32.9, 32.3, 31.6, 27.5(3), 27.4(8), 27.0, 26.9, 26.8, 26.7, 26.6, 26.5, 24.4, 24.2, 21.5, 21.2; IR (neat): 2931, 2860, 2230(s), 1610, 1521, 1474, 1450, 1362, 1181, 1166, 816; HRESIMS Calcd for $[C_{44}H_{58}N_2NaO_2S]^+$ (M + Na$^+$) 701.4111, found 701.4108.

***N*-(4-(4-(dimethylamino)phenyl)-1-(1,4-dioxaspiro[4.5]decan-8-yl)but-3-yn-2-yl)-*N*-((2,6-dimethylphenyl)ethynyl)-4-methylbenzenesulfonamide (1ak)**

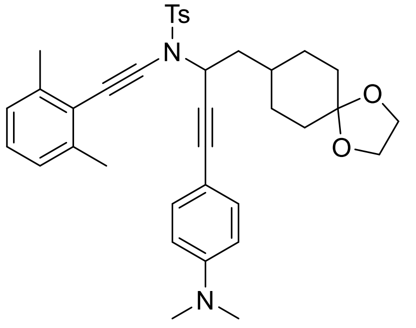

1ak

Pale yellow oil (75%, 458.2 mg), (eluent: PE/EtOAc = 8/1). ^{1}H NMR (500 MHz, $CDCl_3$) δ 7.92 (d, *J* = 8.0 Hz, 2H), 7.25 (d, *J* = 7.5 Hz, 2H), 7.11 – 7.03 (m, 1H), 7.00 (d, *J* = 7.5 Hz, 2H), 6.91 (d, *J* = 9.0 Hz, 2H), 6.50 (d, *J* = 9.0 Hz, 2H), 5.01 (dd, *J* = 9.0, 6.0 Hz, 1H), 3.93 (s, 4H), 2.93 (s, 6H), 2.39 (s, 6H), 2.34 (s, 3H), 2.09 – 1.91 (m, 2H), 1.89 – 1.48 (m, 7H), 1.42 – 1.27 (m, 2H); ^{13}C NMR (125 MHz, $CDCl_3$) δ 150.0, 144.5, 139.8, 134.7, 132.6, 129.4, 128.1, 127.0, 126.5, 122.8, 111.4, 108.8, 87.9, 86.6, 82.7, 70.5, 64.2, 51.9, 41.0, 40.1, 34.3, 34.2, 32.5, 30.2, 29.2, 21.5, 21.2; IR (neat): 2927, 2229(s), 1608, 1522, 1467, 1446, 1363, 1187, 1169, 816; HRESIMS Calcd for $[C_{37}H_{42}N_2NaO_4S]^+$ (M + Na$^+$) 633.2757, found 633.2761.

***N*-(4-(4-(dimethylamino)phenyl)-1-(4,4-dimethylcyclohexyl)but-3-yn-2-yl)-*N*-((2,6-dimethylphenyl)ethynyl)-4-methylbenzenesulfonamide (1al)**

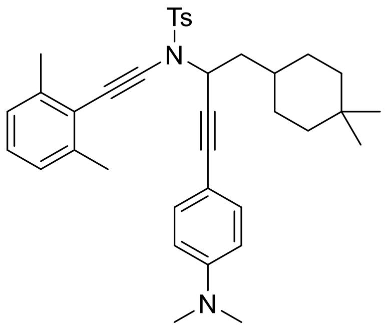

1al

Pale yellow oil (80%, 464.0 mg), (eluent: PE/EtOAc = 8/1). ^{1}H NMR (500 MHz, $CDCl_3$) δ 7.92 (d, *J* = 8.5 Hz, 2H), 7.24 (d, *J* = 9.0 Hz, 2H), 7.08 – 6.96 (m, 3H), 6.91 (d, *J* = 8.5 Hz, 2H), 6.50 (d, *J* = 9.0 Hz, 2H), 5.03 (dd, *J* = 9.0, 6.0 Hz, 1H), 2.93 (s, 6H), 2.39 (s, 6H), 2.34 (s, 3H), 2.04 – 1.96 (m, 1H), 1.84 – 1.68 (m, 2H), 1.60 – 1.09 (m, 10H), 0.90 (s, 3H), 0.87 (s, 3H); ^{13}C NMR (125 MHz, $CDCl_3$) δ 150.0, 144.4, 139.9, 134.8, 132.6, 129.4, 128.2, 127.0, 126.5, 122.9, 111.4, 109.0, 88.1, 86.4, 82.9, 70.4, 51.8, 41.8, 40.1, 38.9, 38.7, 33.7, 32.6, 30.0, 29.0, 28.1, 24.5, 21.6, 21.2; IR (neat): 2917, 2850, 2231(s), 1609, 1522, 1467, 1446, 1363, 1187, 1170; HRESIMS Calcd for $[C_{37}H_{45}N_2O_2S]^+$ (M + H$^+$) 581.3196, found 581.3199.

***N*-(1-((1*R*,3*S*,5*r*,7*r*)-adamantan-2-yl)-4-(4-(dimethylamino)phenyl)but-3-yn-2-yl)-*N*-((2,6-dimethylphenyl)ethynyl)-4-methylbenzenesulfonamide (1am)**

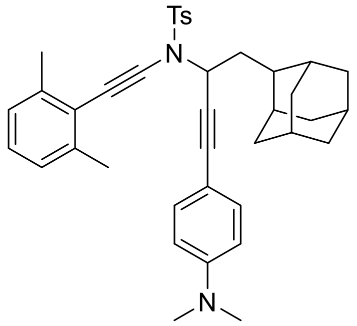

1am

Pale yellow oil (74%, 447.6 mg), (eluent: PE/EtOAc = 8/1). ^{1}H NMR (500 MHz, $CDCl_3$) δ 7.92 (d, *J* = 6.5 Hz, 2H), 7.25 (d, *J* = 7.5 Hz, 2H), 7.10 – 6.97 (m, 3H), 6.93 (d, *J* = 7.0 Hz, 2H), 6.51 (d, *J* = 7.5 Hz, 2H), 5.00 – 4.91 (m, 1H), 2.94 (s, 6H), 2.39 (s, 6H), 2.35 (s, 3H), 2.17 – 2.08 (m, 2H), 2.06 – 1.98 (m, 1H), 1.91 – 1.73 (m, 12H), 1.58 – 1.53 (m, 2H); ^{13}C NMR (125 MHz, $CDCl_3$) δ 150.0, 144.4, 139.9, 134.9, 132.6, 129.4,

128.2, 127.0, 126.5, 122.9, 111.4, 109.1, 88.2, 86.5, 83.1, 70.5, 52.4, 40.4, 40.1, 39.0, 38.9, 38.3, 37.8, 32.2, 31.9, 31.7, 31.0, 28.1, 27.9, 21.6, 21.2; IR (neat): 2906, 2851, 2230(s), 1608, 1522, 1470, 1445, 1366, 1187, 1167; HRESIMS Calcd for $[C_{39}H_{44}N_2NaO_2S]^+$ (M + Na^+) 627.3016, found 627.3020.

_N_-(1-(4-(dimethylamino)phenyl)-5-methylhex-1-yn-3-yl)-_N_-((2,6-dimethylphenyl)ethynyl)-4-methylbenzenesulfonamide (1an)

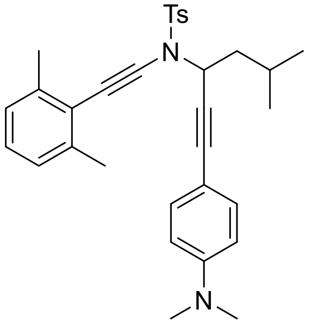

1an

Pale yellow oil (87%, 446.3 mg), (eluent: PE/EtOAc = 8/1). ^{1}H NMR (500 MHz, $CDCl_3$) δ 7.92 (d, *J* = 8.0 Hz, 2H), 7.26 – 7.22 (m, 2H), 7.09 – 7.02 (m, 1H), 6.99 (d, *J* = 7.5 Hz, 2H), 6.91 (d, *J* = 8.5 Hz, 2H), 6.49 (d, *J* = 9.0 Hz, 2H), 5.01 (dd, *J* = 9.0, 6.5 Hz, 1H), 2.92 (s, 6H), 2.39 (s, 6H), 2.33 (s, 3H), 2.06 – 1.88 (m, 2H), 1.80 – 1.70 (m, 1H), 1.03 (d, *J* = 6.0 Hz, 3H), 0.99 (d, *J* = 6.0 Hz, 3H); ^{13}C NMR (125 MHz, $CDCl_3$) δ 150.0, 144.4, 139.9, 134.7, 132.6, 129.4, 128.2, 127.0, 126.5, 122.8, 111.4, 108.9, 88.1, 86.5, 82.7, 70.3, 52.2, 43.5, 40.1, 24.5, 22.6, 21.7, 21.5, 21.2; IR (neat): 2923, 2850, 2230(s), 1608, 1522, 1446, 1372, 1170; HRESIMS Calcd for $[C_{32}H_{36}N_2NaO_2S]^+$ (M + Na^+) 535.2390, found 535.2385.

_N_-(1-(4-(dimethylamino)phenyl)-5-ethylhept-1-yn-3-yl)-_N_-((2,6-dimethylphenyl)ethynyl)-4-methylbenzenesulfonamide (1ao)

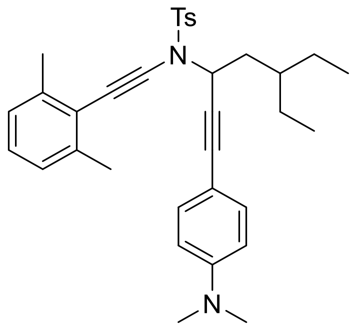

1ao

Pale yellow oil (87%, 470.6 mg), (eluent: PE/EtOAc = 8/1). ^{1}H NMR (400 MHz, $CDCl_3$) δ 7.92 (d, *J* = 8.0 Hz, 2H), 7.24 (d, *J* = 8.0 Hz, 2H), 7.09 – 6.95 (m, 3H), 6.92 (d, *J* = 8.4 Hz, 2H), 6.49 (d, *J* = 8.8 Hz, 2H), 5.11 – 4.89 (m, 1H), 2.92 (s, 6H), 2.39 (s, 6H), 2.34 (s, 3H), 2.04 – 1.79 (m, 2H), 1.64 – 1.55 (m, 1H), 1.53 – 1.31 (m, 4H), 1.00 – 0.78 (m, 6H); ^{13}C NMR (100 MHz, $CDCl_3$) δ 150.0, 144.4, 139.8, 134.9, 132.6, 129.4, 128.1, 126.9, 126.4, 122.9, 111.4, 109.0, 88.2, 86.6, 82.9, 70.4, 52.3, 40.1, 38.2, 36.5, 25.3, 24.7, 21.5, 21.2, 10.7, 10.3; IR (neat): 2922, 2850, 2230(s), 1609, 1522, 1445, 1372, 1170, 819; HRESIMS Calcd for $[C_{34}H_{40}N_2NaO_2S]^+$ (M + Na^+) 563.2703, found 563.2710.

***N*-(1-(4-(dimethylamino)phenyl)-5-propyloct-1-yn-3-yl)-*N*-((2,6-dimethylphenyl)ethynyl)-4-methylbenzenesulfonamide (1ap)**

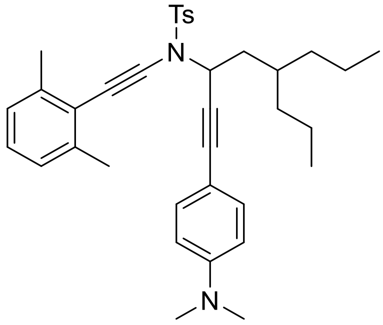

1ap

Pale yellow oil (85%, 482.3 mg), (eluent: PE/EtOAc = 8/1). ^{1}H NMR (400 MHz, $CDCl_3$) δ 7.92 (d, *J* = 8.0 Hz, 2H), 7.24 (d, *J* = 8.0 Hz, 2H), 7.08 – 6.96 (m, 3H), 6.92 (d, *J* = 9.2 Hz, 2H), 6.50 (d, *J* = 8.8 Hz, 2H), 5.08 – 4.93 (m, 1H), 2.92 (s, 6H), 2.38 (s, 6H), 2.34 (s, 3H), 2.02 – 1.81 (m, 2H), 1.76 – 1.67 (m, 1H), 1.40 – 1.29 (m, 8H), 0.98 – 0.86 (m, 6H); ^{13}C NMR (100 MHz, $CDCl_3$) δ 150.0, 144.4, 139.8, 134.9, 132.6, 129.4, 128.2,

126.9, 126.4, 122.9, 111.4, 109.0, 88.2, 86.6, 82.9, 70.5, 52.4, 40.1, 39.1, 35.8, 35.3, 33.5, 21.5, 21.2, 19.6, 19.3, 14.4; IR (neat): 2923, 2851, 2230(s), 1609, 1522, 1444, 1372, 1170, 815; HRESIMS Calcd for $[C_{36}H_{44}N_2NaO_2S]^+$ (M + Na^+) 591.3016, found 591.3021.

_N_-(1-(4-(dimethylamino)phenyl)-5,5-diphenylpent-1-yn-3-yl)-_N_-((2,6-dimethylphenyl)ethynyl)-4-methylbenzenesulfonamide (1aq)

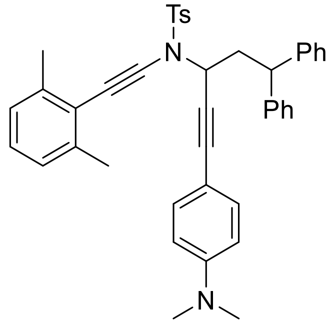

1aq

Pale yellow oil (81%, 515.6 mg), (eluent: PE/EtOAc = 8/1). ^{1}H NMR (500 MHz, $CDCl_3$) δ 7.83 (d, J = 8.5 Hz, 2H), 7.35 – 7.27 (m, 8H), 7.22 – 7.18 (m, 4H), 7.06 – 7.01 (m, 1H), 7.00 – 6.90 (m, 4H), 6.51 (d, J = 9.0 Hz, 2H), 4.77 (dd, J = 8.0, 6.5 Hz, 1H), 4.32 (t, J = 7.5 Hz, 1H), 2.93 (s, 6H), 2.83 – 2.59 (m, 2H), 2.34 (s, 6H), 2.31 (s, 3H); ^{13}C NMR (125 MHz, $CDCl_3$) δ 150.1, 144.5, 143.5, 143.2, 140.0, 134.6, 132.7, 129.4, 128.5(9), 128.5(7), 128.2(1), 128.1(8), 127.8, 127.1, 126.6, 126.4(7), 126.4(5), 122.7, 111.4, 108.8, 88.0, 87.4, 82.3, 70.6, 52.4, 47.2, 40.7, 40.1, 21.5, 21.2; IR (neat): 2923, 2850, 2230(s), 1609, 1523, 1444, 1362, 1113, 815; HRESIMS Calcd for $[C_{42}H_{40}N_2NaO_2S]^+$ (M + Na^+) 659.2703, found 659.2710.

_N_-(1-(4-(dimethylamino)phenyl)-5,7-dimethyloct-1-yn-3-yl)-_N_-((2,6-dimethylphenyl)ethynyl)-4-methylbenzenesulfonamide (1ar)

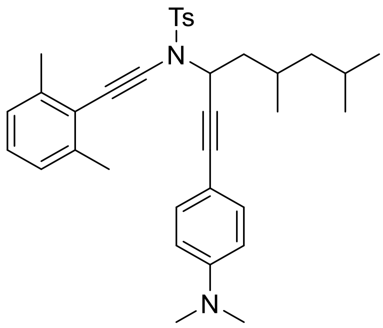

1ar

Prepared as a pale yellow oil (79%, 438.5 mg) with 1:1 dr (determined by ^{1}H NMR analysis), (eluent: PE/EtOAc = 8/1). ^{1}H NMR (500 MHz, CDCl$_3$) δ 7.92 (d, *J* = 8.5 Hz, 2H), 7.27 – 7.22 (m, 2H), 7.07 – 7.02 (m, 1H), 7.01 – 6.96 (m, 2H), 6.91 (t, *J* = 9.0 Hz, 2H), 6.54 – 6.46 (m, 2H), 5.08 – 4.97 (m, 1H), 2.92 (s, 6H), 2.39 (s, 6H), 2.36 – 2.30 (m, 3H), 2.20 – 1.92 (m, 1H), 1.85 – 1.58 (m, 2H), 1.56 – 1.22 (m, 3H), 1.06 – 0.93 (m, 3H), 0.91 – 0.87 (m, 3H), 0.87 – 0.80 (m, 3H); ^{13}C NMR (125 MHz, CDCl$_3$) δ 149.9(9), 149.9(6), 144.4, 139.9, 139.7, 134.8, 134.8, 132.5(9), 132.5(5), 129.4, 129.3, 128.2, 128.1, 127.0, 126.9, 126.5, 122.9, 122.8, 111.4, 108.9, 88.3, 87.9, 86.7, 86.4, 83.0, 82.6, 70.5, 70.2, 52.4, 51.9, 41.8 40.1 36.0, 35.8, 34.6, 34.0, 29.7, 29.2, 28.2, 28.1, 22.7(1), 22.6(7), 22.5(4), 22.4(6), 21.5, 21.2, 19.7, 19.0; IR (neat): 2925, 2850, 2231(s), 1609, 1522, 1447, 1372, 1168, 815; HRESIMS Calcd for $[C_{35}H_{42}N_2NaO_2S]^+$ (M + Na$^+$) 577.2859, found 577.2866.

***N*-(1-(4-(dimethylamino)phenyl)-5-phenylpent-1-yn-3-yl)-*N*-((2,6-dimethylphenyl)ethynyl)-4-methylbenzenesulfonamide (1as)**

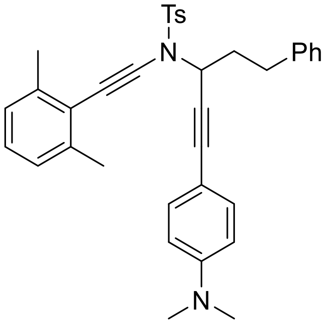

1as

Pale yellow oil (80%, 448.8 mg), (eluent: PE/EtOAc = 10/1). ^{1}H NMR (400 MHz, CDCl$_3$) δ 7.90 (d, *J* = 8.4 Hz, 2H), 7.31 – 7.17 (m, 7H), 7.09 – 7.02 (m, 1H), 7.01 –

6.96 (m, 2H), 6.93 (d, *J* = 8.8 Hz, 2H), 6.50 (d, *J* = 8.8 Hz, 2H), 4.93 (dd, *J* = 8.4, 6.8 Hz, 1H), 2.98 – 2.77 (m, 8H), 2.39 (s, 6H), 2.33 (s, 3H), 2.28 – 2.11 (m, 2H); ^{13}C NMR (100 MHz, $CDCl_3$) δ 150.1, 144.5, 140.6, 139.9, 134.7, 132.6, 129.4, 128.6, 128.4, 128.2, 127.1, 126.5, 126.1, 122.8, 111.4, 108.8, 87.9, 87.1, 82.4, 70.5, 53.4, 40.1, 36.5, 32.0, 21.5, 21.2; IR (neat): 2922, 2851, 2230(s), 1608, 1510, 1446, 1366, 1168, 815; HRESIMS Calcd for $[C_{36}H_{36}N_2NaO_2S]^+$ (M + Na^+) 583.2390, found 583.2379.

***N*-(1-(4-(dimethylamino)phenyl)hept-1-yn-3-yl)-*N*-((2,6-dimethylphenyl)ethynyl)-4-methylbenzenesulfonamide (1at)**

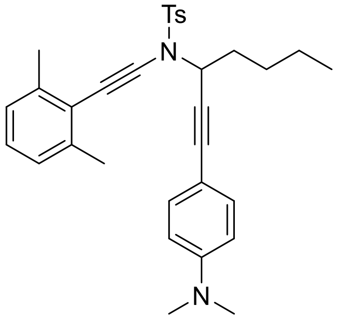

1at

Pale yellow oil (82%, 420.1 mg), (eluent: PE/EtOAc = 8/1). ^{1}H NMR (400 MHz, $CDCl_3$) δ 7.91 (d, *J* = 8.4 Hz, 2H), 7.24 (d, *J* = 8.0 Hz, 2H), 7.06 – 6.96 (m, 3H), 6.93 (d, *J* = 8.8 Hz, 2H), 6.49 (d, *J* = 8.8 Hz, 2H), 4.91 (dd, *J* = 8.0, 6.8 Hz, 1H), 2.91 (s, 6H), 2.39 (s, 6H), 2.33 (s, 3H), 2.08 – 1.85 (m, 2H), 1.59 – 1.47 (m, 2H), 1.44 – 1.32 (m, 2H), 0.93 (t, *J* = 7.2 Hz, 3H); ^{13}C NMR (100 MHz, $CDCl_3$) δ 150.0, 144.4, 139.8, 134.8, 132.6, 129.4, 128.1, 126.9, 126.4, 122.8, 111.4, 108.9, 88.1, 86.6, 82.7, 70.4, 53.8, 40.0, 34.5, 27.9, 22.0, 21.5, 21.1, 13.9; IR (neat): 2923, 2851, 2229(s), 1608, 1522, 1447, 1365, 1263, 1186, 815; HRESIMS Calcd for $[C_{32}H_{36}N_2NaO_2S]^+$ (M + Na^+) 535.2390, found 535.2395.

***N*-(1-cyclohexyl-3-(4-(dimethylamino)phenyl)prop-2-yn-1-yl)-*N*-((2,6-dimethylphenyl)ethynyl)-4-methylbenzenesulfonamide (1au)**

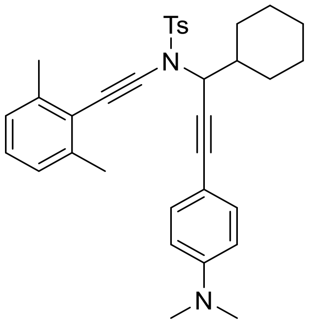

1au

Pale yellow oil (63%, 339.5 mg), (eluent: PE/EtOAc = 8/1). ^{1}H NMR (500 MHz, $CDCl_3$) δ 7.91 (d, *J* = 8.0 Hz, 2H), 7.25 (d, *J* = 8.0 Hz, 2H), 7.07 – 6.95 (m, 3H), 6.92 (d, *J* = 8.5 Hz, 2H), 6.49 (d, *J* = 8.5 Hz, 2H), 4.61 (d, *J* = 10.0 Hz, 1H), 2.92 (s, 6H), 2.37 (s, 6H), 2.34 (s, 3H), 2.18 – 2.07 (m, 2H), 1.96 – 1.62 (m, 4H), 1.32 – 1.08 (m, 5H); ^{13}C NMR (125 MHz, $CDCl_3$) δ 150.0, 144.3, 139.9, 134.9, 132.6, 129.4, 128.1, 127.0, 126.4, 122.9, 111.4, 109.1, 88.4, 87.4, 82.0, 70.1, 59.3, 41.4, 40.1, 29.9, 29.6, 26.2, 25.9, 25.6, 21.5, 21.2; IR (neat): 2923, 2851, 2236(s), 1608, 1521, 1447, 1363, 1229, 1169, 815; HRESIMS Calcd for $[C_{34}H_{38}N_2NaO_2S]^+$ (M + Na$^+$) 561.2546, found 561.2552.

***N*-(5-cyclohexyl-1-(4-(dimethylamino)phenyl)pent-1-yn-3-yl)-*N*-((2,6-dimethylphenyl)ethynyl)-4-methylbenzenesulfonamide (1av)**

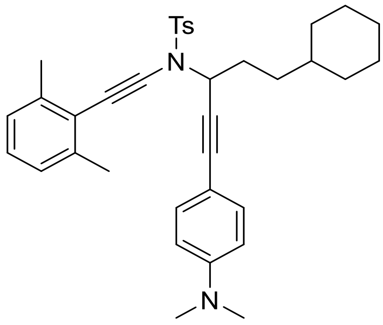

1av

Pale yellow oil (87%, 493.2 mg), (eluent: PE/EtOAc = 8/1). ^{1}H NMR (500 MHz, $CDCl_3$) δ 7.91 (d, *J* = 8.0 Hz, 2H), 7.25 (d, *J* = 8.5 Hz, 2H), 7.08 – 7.02 (m, 1H), 7.02 – 6.97 (m, 2H), 6.93 (d, *J* = 9.0 Hz, 2H), 6.51 (d, *J* = 8.5 Hz, 2H), 4.86 (dd, *J* = 8.0, 7.0 Hz, 1H), 2.94 (s, 6H), 2.38 (s, 6H), 2.35 (s, 3H), 2.07 – 1.90 (m, 2H), 1.74 – 1.61 (m, 5H), 1.48 – 1.36 (m, 2H), 1.25 – 1.08 (m, 4H), 0.95 – 0.85 (m, 2H); ^{13}C NMR (125 MHz, $CDCl_3$) δ 150.1, 144.4, 139.9, 134.9, 132.7, 129.4, 128.2, 127.0, 126.5, 123.0, 111.4,

109.1, 88.1, 86.6, 82.9, 70.4, 54.3, 40.1, 37.2, 33.4, 33.3, 33.2, 32.4, 26.6, 26.3, 21.6, 21.2; IR (neat): 2924, 2852, 2230(s), 1609, 1522, 1446, 1363, 1187, 1168, 815; HRESIMS Calcd for $[C_{36}H_{42}N_2NaO_2S]^+$ (M + Na^+) 589.2859, found 589.2868.

_N_-(5-(benzyloxy)-1-(4-(dimethylamino)phenyl)pent-1-yn-3-yl)-_N_-((2,6-dimethylphenyl)ethynyl)-4-methylbenzenesulfonamide (1aw)

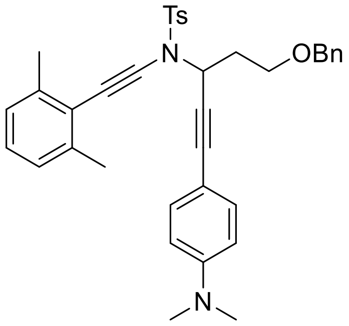

1aw

Pale yellow oil (82%, 484.4 mg), (eluent: PE/EtOAc = 8/1). ^{1}H NMR (500 MHz, $CDCl_3$) δ 7.92 (d, *J* = 8.5 Hz, 2H), 7.44 – 7.38 (m, 2H), 7.36 – 7.31 (m, 2H), 7.29 – 7.21 (m, 3H), 7.08 – 7.01 (m, 1H), 7.00 – 6.95 (m, 2H), 6.91 (d, *J* = 9.0 Hz, 2H), 6.49 (d, *J* = 9.0 Hz, 2H), 5.26 (dd, *J* = 8.5, 6.5 Hz, 1H), 4.60 (d, *J* = 12.0 Hz, 1H), 4.51 (d, *J* = 12.0 Hz, 1H), 3.68 (t, *J* = 5.5 Hz, 2H), 2.91 (s, 6H), 2.37 (s, 6H), 2.33 (s, 3H), 2.32 – 2.15 (m, 2H); ^{13}C NMR (125 MHz, $CDCl_3$) δ 150.0, 144.5, 139.8, 138.2, 134.7, 132.6, 129.4, 128.3, 128.1, 127.7, 127.5, 127.0, 126.5, 122.7, 111.3, 108.8, 88.0, 86.8, 82.2, 73.2, 70.5, 65.9, 51.1, 40.0, 35.1, 21.5, 21.2; IR (neat): 2930, 2851, 2230(s), 1609, 1521, 1447, 1368, 1229, 1165, 819; HRESIMS Calcd for $[C_{37}H_{38}N_2NaO_3S]^+$ (M + Na^+) 613.2495, found 613.2488.

_N_-(1-cyclohexyl-4-(4-(dimethylamino)phenyl)but-3-yn-2-yl-2-d)-_N_-((2,6-dimethylphenyl)ethynyl)-4-methylbenzenesulfonamide ((±)-[D]*-1a)

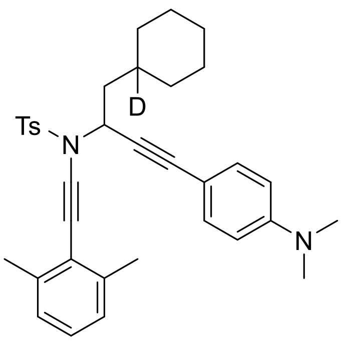

(±)-[D]*-**1a**

Compound (±)-[D]*-**1a** was prepared from 2-(cyclohexyl-1-*d*)ethan-1-ol[4] according to the general procedure. Pale yellow oil (78%, 431.4 mg), (eluent: PE/EtOAc = 8/1). ^{1}H NMR (500 MHz, $CDCl_3$) δ 7.92 (d, *J* = 8.0 Hz, 2H), 7.24 (d, *J* = 8.0 Hz, 2H), 7.09 – 6.95 (m, 3H), 6.91 (d, *J* = 9.0 Hz, 2H), 6.49 (d, *J* = 9.0 Hz, 2H), 5.03 (dd, *J* = 9.0, 6.5 Hz, 1H), 2.92 (s, 6H), 2.39 (s, 6H), 2.33 (s, 3H), 2.00 – 1.86 (m, 2H), 1.78 – 1.64 (m, 5H), 1.30 – 1.10 (m, 3H), 1.06 – 0.89 (m, 2H); ^{13}C NMR (125 MHz, $CDCl_3$) δ 150.0, 144.4, 139.8, 134.8, 132.6, 129.4, 128.2, 127.0, 126.5, 122.9, 111.4, 109.0, 88.1, 86.4, 82.9, 70.4, 51.6, 42.0, 40.1, 33.1, 32.2, 26.4, 26.1, 25.9, 21.5, 21.2; IR (neat): 2924, 2852, 2230(s), 1610, 1522, 1450, 1367, 1188, 1088; HRESIMS Calcd for $[C_{35}H_{39}DN_2NaO_2S]^+$ (M + Na^+) 576.2765, found 576.2773.

***N*-(1-cyclohexyl-4-(4-(dimethylamino)phenyl)but-3-yn-2-yl-2-d)-*N*-((2,6-dimethylphenyl)ethynyl)-4-methylbenzenesulfonamide ((±)-[D]-1a)**

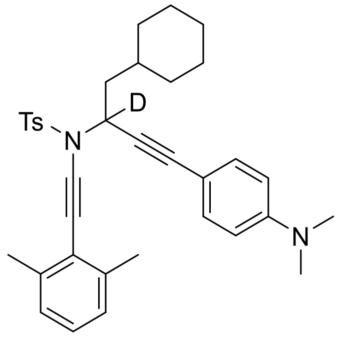

(±)-[D]-**1a**

Compound (±)-[D]-**1a** was prepared according to the known procedure[3]. Pale yellow oil (81%, 448.1 mg), (eluent: PE/EtOAc = 8/1). ^{1}H NMR (400 MHz, $CDCl_3$) δ 7.92 (d, *J* = 8.0 Hz, 2H), 7.23 (d, *J* = 8.0 Hz, 2H), 7.10 – 6.94 (m, 3H), 6.91 (d, *J* = 9.2 Hz, 2H),

6.49 (d, J = 9.2 Hz, 2H), 2.91 (s, 6H), 2.39 (s, 6H), 2.33 (s, 3H), 2.02 – 1.85 (m, 2H), 1.80 – 1.57 (m, 6H), 1.35 – 1.11 (m, 3H), 1.05 – 0.86 (m, 2H); ^{13}C NMR (100 MHz, $CDCl_3$) δ 149.9, 144.4, 139.8, 134.7, 132.6, 129.3, 128.1, 126.9, 126.4, 122.8, 111.3, 108.9, 88.1, 86.4, 82.8, 70.3, 42.0, 40.0, 33.7, 33.2, 32.3, 26.4, 26.1, 25.9, 21.5, 21.2; IR (neat): 2923, 2850, 2230(s), 1610, 1522, 1447, 1187, 1169, 1090, 816; HRESIMS Calcd for $[C_{35}H_{40}DN_2O_2S]^+$ (M + H^+) 554.2946, found 554.2951.

Synthesis of chiral substrate (-)-1an

To confirm the absolute configuration of (+)-**1** obtained through copper-catalyzed kinetic resolution, chiral substrate (-)-**1an** was prepared from a chiral starting material *L*-Valinol. We compared the optical rotation of (-)-**1an** and (+)-**1**, and assigned the absolute configuration of (+)-**1** to be *R*.

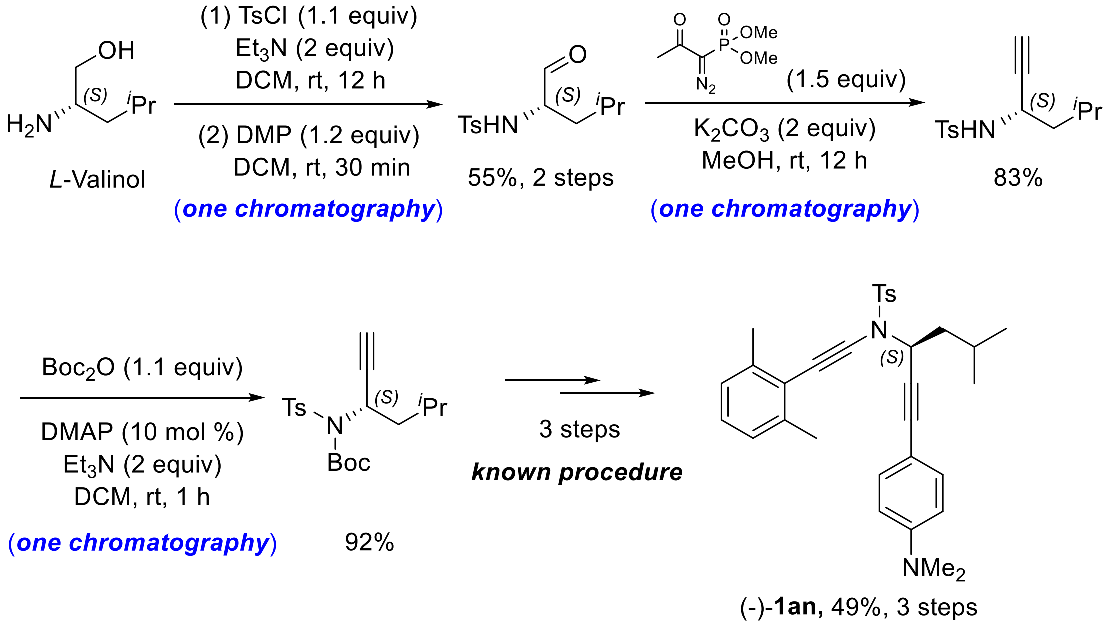

To the solution of *L*-Valinol (10 mmol, 1.1 mL) in DCM (50 mL) were added TsCl (11 mmol, 2.10 g) and Et_3N (20 mmol, 2.8 mL), and the reaction was stirred at room temperature for 12 h. The progress of the reaction was monitored by TLC. Upon completion, the reaction was diluted with water and extracted with DCM (3 x 50 mL). The combined organic layers was dried over anhydrous $MgSO_4$ and concentrated under

vacuum. The crude Ts-protected *L*-Valinol was directly used in the next step without further purification.

To the solution of the crude Ts-protected *L*-Valinol (10 mmol) in DCM (50 mL) was added Dess Martin Periodinane (12 mmol, 5.09 g), and the reaction was stirred at room temperature for 30 min. The progress of the reaction was monitored by TLC. Upon completion, the reaction was quenched with aqueous $NaHCO_3$ and extracted with DCM (3 x 50 mL). The combined organic layer was dried over anhydrous $MgSO_4$ and concentrated under vacuum. The residue was purified by column chromatography on silica gel (eluent: hexanes/EtOAc) to afford the desired aldehyde (55%, 2 steps).

To the solution of the above aldehyde (5.5 mmol, 1.48 g), K_2CO_3 (11 mmol, 1.52 g) in MeOH (30 mL) was added dimethyl (1-diazo-2-oxopropyl)phosphonate (8.5 mmol, 1.3 mL) dropwise at room temperature and stirred for additional 12 h. The progress of the reaction was monitored by TLC. Upon completion, the mixture was filtered through a pad of silica gel and the filtrate was concentrated under reduce pressure. The residue was purified by column chromatography on silica gel (eluent: hexanes/EtOAc) to give the desired alkyne (83% yield).

To the solution of the above alkyne (4.6 mmol, 1.21 g), DMAP (0.46 mmol, 55.8 mg) and Et_3N (9.2 mmol, 1.3 mL) in DCM (30 mL) was added Boc_2O (5 mmol, 1.2 mL) dropwise at room temperature and stirred for additional 1 h. The progress of the reaction was monitored by TLC. Upon completion, the reaction was quenched with HCl (1 M). The resulting mixture was extracted with DCM (3 x 50 mL). The combined organic layers was dried over anhydrous $MgSO_4$ and concentrated under vacuum. The residue was purified by column chromatography on silica gel (eluent: hexanes/EtOAc) to give the desired Boc-protected propargylamide (92% yield).

Fuether transformation of the Boc-protected propargylamide according to the general procedure for the preparation of ynamide **1** afforded (-)-**1an** in 49% yield (3 steps) as a pale yellow oil. $[\alpha]_D^{25}$ = -36.5º (c = 1.0, $CHCl_3$). The optical rotation of (-)-**1an** revealed the absolute configuration of (+)-**1** to be *R*.

4. General Procedure for the Copper-Catalyzed Kinetic Resolution

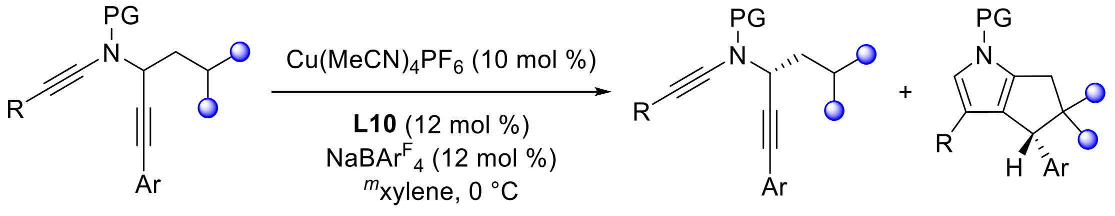

(±)-**1** (+)-**1** (-)-**2**

The powered Cu(MeCN)$_4$PF$_6$ (0.02 mmol, 7.5 mg), **L10** (0.024 mmol, 17.4 mg), and NaBAr$^F{}_4$ (0.024 mmol, 21.3 mg) were introduced into an oven-dried Schlenk tube under argon atmosphere. After ^{m}xylene (2 mL) was injected into the Schlenk tube, the solution was stirred at rt under the argon atmosphere for 2 h. Then the reaction was cooled to 0 °C, and *N*-propargyl ynamide **1** (0.2 mmol) in ^{m}xylene (2 mL) was introduced into the system dropwise. The resulting mixture was stirred at indicating temperature and the progress of the reaction was monitored by TLC or HPLC. After concentration in vacuo, the residue was purified by flash chromatography on silica gel (eluent: hexanes/EA or hexanes/DCM) to give the final product (+)-**1** and (-)-**2**.

(*R*)-*N*-(1-cyclohexyl-4-(4-(dimethylamino)phenyl)but-3-yn-2-yl)-*N*-((2,6-dimethylphenyl)ethynyl)-4-methylbenzenesulfonamide ((+)-1a)

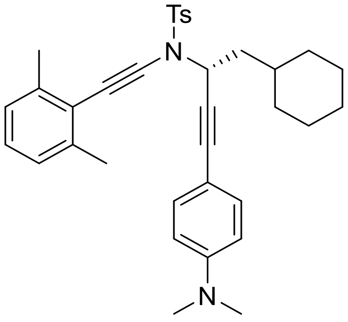

(+)-**1a**

Compound (+)-**1a** was prepared in 42% yield (46.4 mg) according to the general procedure. Pale yellow oil, (eluent: PE/DCM = 2/1). $[\alpha]_D^{25}$ = +106.3° (c = 1.0, $CHCl_3$). 95% ee (determined by HPLC: Chiralpak IE Column, 5/95 *i*-PrOH/hexane, 1.0 mL/min, 254 nm; TR = 16.73 min (minor), 18.42 min (major)).

(*R*)-4-(3'-(2,6-dimethylphenyl)-1'-tosyl-4',6'-dihydro-1'*H*-spiro[cyclohexane-1,5'-cyclopenta[*b*]pyrrol]-4'-yl)-*N*,*N*-dimethylaniline ((-)-2a)

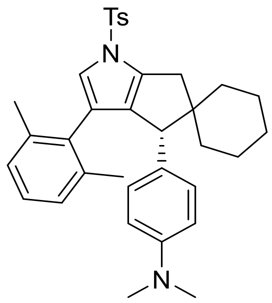

(-)-**2a**

Compound (-)-**2a** was prepared in 40% yield (44.2 mg) according to the general procedure. Pale yellow oil, (eluent: PE/DCM = 2/1). $[\alpha]_D^{25}$ = -46.5º (c = 1.0, $CHCl_3$). 94% ee (determined by HPLC: Chiralpak IE Column, 5/95 *i*-PrOH/hexane, 1.0 mL/min, 254 nm; TR = 10.23 min (minor), 11.65 min (major)). ^{1}H NMR (500 MHz, $CDCl_3$) δ 7.75 (d, *J* = 8.5 Hz, 2H), 7.32 (d, *J* = 8.0 Hz, 2H), 6.98 – 6.89 (m, 2H), 6.87 (s, 1H), 6.78 (d, *J* = 7.5 Hz, 1H), 6.45 (d, *J* = 8.0 Hz, 2H), 6.38 (d, *J* = 8.5 Hz, 2H), 3.22 (s, 1H), 2.90 (d, *J* = 16.0 Hz, 1H), 2.82 (s, 6H), 2.71 (d, *J* = 16.0 Hz, 1H), 2.45 (s, 3H), 1.95 (s, 3H), 1.64 (s, 3H), 1.59 – 1.33 (m, 6H), 1.23 – 1.12 (m, 2H), 1.09 – 1.01 (m, 1H), 0.96 – 0.82 (m, 1H); ^{13}C NMR (125 MHz, $CDCl_3$) δ 149.0, 144.5, 137.1, 136.9(8), 136.9(5), 136.4(0), 136.3(7), 133.0, 129.7, 129.0, 128.7, 126.8, 126.8, 126.5, 126.5, 125.3, 121.6, 111.9, 55.7, 53.9, 40.7, 38.9, 36.8, 35.1, 26.0, 23.3(0), 23.2(9), 21.6, 20.5, 20.3; IR (neat): 2924, 2851, 1614, 1519, 1449, 1370, 1186, 1175, 1124, 1102; HRESIMS Calcd for $[C_{35}H_{40}KN_2O_2S]^+$ (M + K^+) 591.2442, found 591.2444.

(*R*)-*N*-(1-cyclohexyl-4-(4-(dimethylamino)phenyl)but-3-yn-2-yl)-*N*-((2,6-dimethylphenyl)ethynyl)-4-methoxybenzenesulfonamide ((+)-1b)

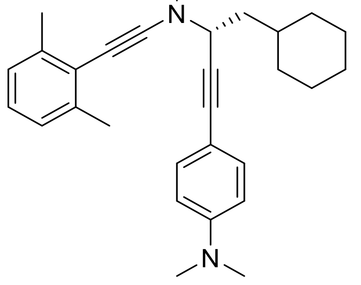

(+)-**1b**

Compound (+)-**1b** was prepared in 41% yield (46.7 mg) according to the general procedure. Pale yellow oil, (eluent: PE/DCM = 1/1). $[\alpha]_D^{25}$ = +84.3º (c = 1.0, $CHCl_3$). 99% ee (determined by HPLC: Chiralpak IE Column, 20/80 *i*-PrOH/hexane, 1.0 mL/min, 254 nm; TR = 10.72 min (minor), 11.49 min (major)).

(*R*)-4-(3'-(2,6-dimethylphenyl)-1'-((4-methoxyphenyl)sulfonyl)-4',6'-dihydro-1'*H*-spiro[cyclohexane-1,5'-cyclopenta[*b*]pyrrol]-4'-yl)-*N*,*N*-dimethylaniline ((-)-2b)

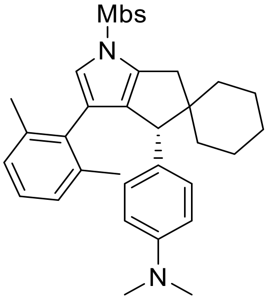

(-)-**2b**

Compound (-)-**2b** was prepared in 43% yield (48.8 mg) according to the general procedure. Pale yellow oil, (eluent: PE/DCM = 1/1). $[\alpha]_D^{25}$ = -27.6º (c = 1.0, $CHCl_3$). 95% ee (determined by HPLC: Chiralpak IE Column, 20/80 *i*-PrOH/hexane, 1.0 mL/min, 254 nm; TR = 7.29 min (minor), 8.24 min (major)). ^{1}H NMR (500 MHz, $CDCl_3$) δ 7.81 (d, *J* = 9.0 Hz, 2H), 7.02 – 6.89 (m, 4H), 6.86 (s, 1H), 6.78 (d, *J* = 7.0 Hz, 1H), 6.50 – 6.35 (m, 4H), 3.88 (s, 3H), 3.22 (s, 1H), 2.91 (d, *J* = 16.0 Hz, 1H), 2.82 (s, 6H), 2.71 (d, *J* = 16.0 Hz, 1H), 1.96 (s, 3H), 1.64 (s, 3H), 1.62 – 1.43 (m, 4H), 1.43 – 1.29 (m, 2H), 1.25 – 1.14 (m, 2H), 1.08 – 0.87 (m, 2H); ^{13}C NMR (125 MHz, $CDCl_3$) δ 163.6, 149.0, 137.1, 137.0, 136.4, 133.0, 130.9, 129.0, 128.8, 126.9, 126.8, 126.5, 125.2, 121.5, 114.2, 111.9, 55.7, 53.9, 40.7, 38.9, 36.9, 35.2, 26.0, 23.3, 20.6, 20.4; IR (neat): 2925, 2850, 1614, 1595, 1519, 1497, 1368, 1262, 1186, 1167; HRESIMS Calcd for $[C_{35}H_{40}KN_2O_3S]^+$ (M + K^+) 607.2391, found 607.2385.

(*R*)-*N*-(1-cyclohexyl-4-(4-(dimethylamino)phenyl)but-3-yn-2-yl)-*N*-((2,6-dimethylphenyl)ethynyl)benzenesulfonamide ((+)-1c)

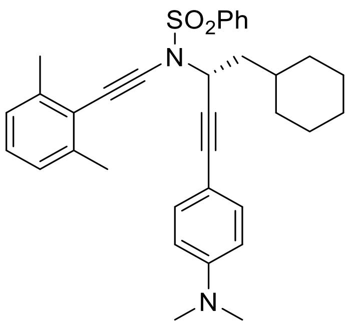

(+)-**1c**

Compound (+)-**1c** was prepared in 44% yield (47.3 mg) according to the general procedure. Pale yellow oil, (eluent: PE/DCM = 2/1). $[\alpha]_D^{25}$ = +56.2º (c = 1.0, $CHCl_3$). 95% ee (determined by HPLC: Chiralpak IE Column, 3/97 *i*-PrOH/hexane, 1.0 mL/min, 254 nm; TR = 17.08 min (minor), 18.53 min (major)).

(*R*)-4-(3'-(2,6-dimethylphenyl)-1'-(phenylsulfonyl)-4',6'-dihydro-1'*H*-spiro[cyclohexane-1,5'-cyclopenta[*b*]pyrrol]-4'-yl)-*N,N*-dimethylaniline ((-)-2c)

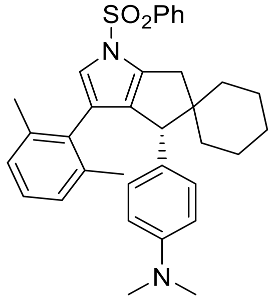

(-)-**2c**

Compound (-)-**2c** was prepared in 43% yield (46.3 mg) according to the general procedure. Pale yellow oil, (eluent: PE/DCM = 2/1). $[\alpha]_D^{25}$ = -18.7º (c = 1.0, $CHCl_3$). 96% ee (determined by HPLC: Chiralpak IE Column, 5/95 *i*-PrOH/hexane, 1.0 mL/min, 254 nm; TR = 8.98 min (minor), 10.42 min (major)). ^{1}H NMR (500 MHz, $CDCl_3$) δ 7.87 (d, *J* = 7.5 Hz, 2H), 7.63 (t, *J* = 7.5 Hz, 1H), 7.57 – 7.50 (m, 2H), 6.97 – 6.89 (m, 2H), 6.88 (s, 1H), 6.78 (d, *J* = 7.5 Hz, 1H), 6.47 (d, *J* = 8.5 Hz, 2H), 6.40 (d, *J* = 9.0 Hz, 2H), 3.22 (s, 1H), 2.92 (d, *J* = 16.0 Hz, 1H), 2.82 (s, 6H), 2.74 (d, *J* = 16.0 Hz, 1H), 1.94 (s, 3H), 1.63 (s, 3H), 1.59 – 1.35 (m, 6H), 1.22 – 1.12 (m, 2H), 1.10 – 1.02 (m, 1H), 0.96 – 0.88 (m, 1H); ^{13}C NMR (125 MHz, $CDCl_3$) δ 149.0, 139.3, 137.2, 137.0, 136.9, 136.6, 133.5, 132.9, 129.1, 129.0, 128.7, 126.9, 126.8, 126.5, 125.5, 121.6, 112.0,

55.7, 53.9, 40.7, 38.9, 36.9, 35.1, 26.0, 23.3, 20.5, 20.3; IR (neat): 2924, 2851, 1614, 1519, 1448, 1370, 1183, 1124, 1100, 735; HRESIMS Calcd for $[C_{34}H_{39}N_2O_2S]^+$ (M + H^+) 539.2727, found 539.2721.

(*R*)-4-bromo-*N*-(1-cyclohexyl-4-(4-(dimethylamino)phenyl)but-3-yn-2-yl)-*N*-((2,6-dimethylphenyl)ethynyl)benzenesulfonamide ((+)-1d)

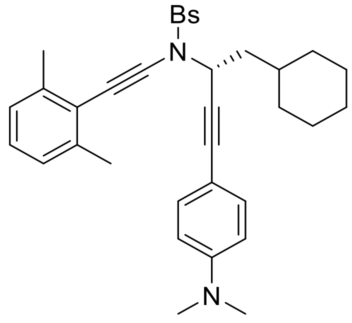

(+)-**1d**

Compound (+)-**1d** was prepared in 42% yield (52.0 mg) according to the general procedure. Pale yellow oil, (eluent: PE/DCM = 2/1). $[\alpha]_D^{25}$ = +128.4º (c = 1.0, $CHCl_3$). 98% ee (determined by HPLC: Chiralpak IE Column, 5/95 *i*-PrOH/hexane, 1.0 mL/min, 254 nm; TR = 9.44 min (minor), 10.41 min (major)).

(*R*)-4-(1'-((4-bromophenyl)sulfonyl)-3'-(2,6-dimethylphenyl)-4',6'-dihydro-1'*H*-spiro[cyclohexane-1,5'-cyclopenta[*b*]pyrrol]-4'-yl)-*N,N*-dimethylaniline ((-)-2d)

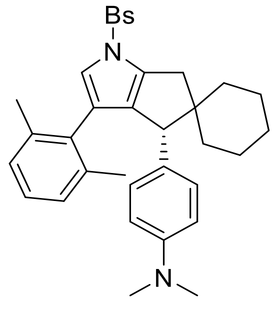

(-)-**2d**

Compound (-)-**2d** was prepared in 43% yield (53.0 mg) according to the general procedure. Pale yellow oil, (eluent: PE/DCM = 2/1). $[\alpha]_D^{25}$ = -33.4º (c = 1.0, $CHCl_3$). 96% ee (determined by HPLC: Chiralpak IE Column, 5/95 *i*-PrOH/hexane, 1.0 mL/min, 254 nm; TR = 7.26 min (minor), 8.27 min (major)). ^{1}H NMR (500 MHz, $CDCl_3$) δ 7.74

(d, J = 8.5 Hz, 2H), 7.68 (d, J = 9.0 Hz, 2H), 6.99 – 6.89 (m, 2H), 6.86 (s, 1H), 6.79 (d, J = 7.0 Hz, 1H), 6.44 – 6.31 (m, 4H), 3.22 (s, 1H), 2.90 (d, J = 16.0 Hz, 1H), 2.83 (s, 6H), 2.68 (d, J = 16.0 Hz, 1H), 1.97 (s, 3H), 1.61 (s, 3H), 1.59 – 1.34 (m, 6H), 1.22 – 1.11 (m, 2H), 1.10 – 1.01 (m, 1H), 0.97 – 0.89 (m, 1H); ^{13}C NMR (125 MHz, $CDCl_3$) δ 149.1, 138.2, 137.3, 137.2, 136.9, 132.6, 132.4, 128.9, 128.6, 128.3, 128.0, 127.0, 126.9, 126.6, 126.1, 121.6, 111.9, 55.6, 54.0, 40.7, 38.9, 37.0, 35.2, 26.0, 23.3(1), 23.2(8), 20.6, 20.3; IR (neat): 2924, 2851, 1614, 1574, 1519, 1471, 1448, 1374, 1184, 1122; HRESIMS Calcd for $[C_{34}H_{37}BrN_2O_2SK]^+$ (M + K^+) 655.1391, found 655.1399.

(*R*)-*N*-(1-cyclohexyl-4-(4-(dimethylamino)phenyl)but-3-yn-2-yl)-*N*-((2,6-dimethylphenyl)ethynyl)methanesulfonamide ((+)-1e)

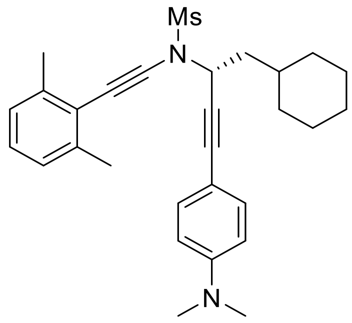

(+)-**1e**

Compound (+)-**1e** was prepared in 41% yield (39.0 mg) according to the general procedure. Pale yellow oil, (eluent: PE/DCM = 2/1). $[\alpha]_D^{25}$ = +23.3º (c = 1.0, $CHCl_3$). 87% ee (determined by HPLC: Chiralpak IE Column, 5/95 *i*-PrOH/hexane, 1.0 mL/min, 254 nm; TR = 11.05 min (minor), 12.90 min (major)).

(*R*)-4-(3'-(2,6-dimethylphenyl)-1'-(methylsulfonyl)-4',6'-dihydro-1'*H*-spiro[cyclohexane-1,5'-cyclopenta[*b*]pyrrol]-4'-yl)-*N*,*N*-dimethylaniline ((-)-2e)

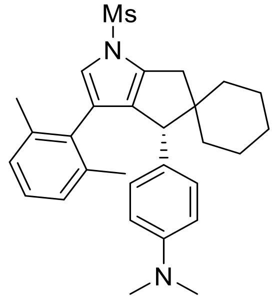

(-)-**2e**

Compound (-)-**2e** was prepared in 49% yield (46.6 mg) according to the general procedure. Pale yellow oil, (eluent: PE/DCM = 2/1). $[\alpha]_D^{25}$ = -16.7º (c = 1.0, $CHCl_3$). 91% ee (determined by HPLC: Chiralpak ODH Column, 5/95 *i*-PrOH/hexane, 1.0 mL/min, 254 nm; TR = 9.02 min (minor), 10.17 min (major)). ^{1}H NMR (500 MHz, $CDCl_3$) δ 7.01 – 6.91 (m, 2H), 6.81 (d, *J* = 7.0 Hz, 1H), 6.79 (s, 1H), 6.66 (d, *J* = 9.0 Hz, 2H), 6.48 (d, *J* = 8.5 Hz, 2H), 3.38 (s, 1H), 3.16 (s, 3H), 2.92 (d, *J* = 16.0 Hz, 1H), 2.87 – 2.79 (m, 7H), 2.10 (s, 3H), 1.86 – 1.78 (m, 1H), 1.76 (s, 3H), 1.68 – 1.49 (m, 5H), 1.25 – 1.13 (m, 3H), 1.03 – 0.93 (m, 1H); ^{13}C NMR (125 MHz, $CDCl_3$) δ 149.1, 137.0(1), 136.9(8), 136.4, 135.5, 132.9, 129.1, 128.8, 126.9(1), 126.8(7), 126.5, 124.6, 120.6, 112.1, 55.8, 54.0, 42.3, 40.7, 39.3, 37.0, 35.2, 26.0, 23.4, 23.3, 20.7, 20.5; IR (neat): 2925, 2852, 1614, 1519, 1447, 1365, 1176, 1130, 958, 766; HRESIMS Calcd for $[C_{29}H_{37}N_2O_2S]^+$ (M + H^+) 477.2570, found 477.2569.

(*R*)-*N*-(1-cyclohexyl-4-(4-(dibenzylamino)phenyl)but-3-yn-2-yl)-*N*-((2,6-dimethylphenyl)ethynyl)-4-methylbenzenesulfonamide ((+)-1f)

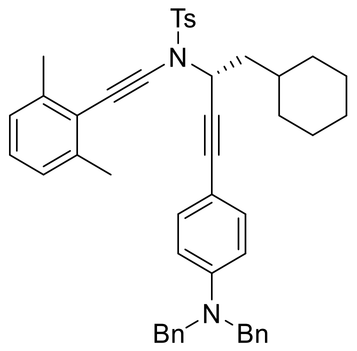

(+)-**1f**

Compound (+)-**1f** was prepared in 41% yield (57.8 mg) according to the general procedure at 30 °C. Pale yellow oil, (eluent: PE/DCM = 2/1). $[\alpha]_D^{25}$ = +71.7º (c = 1.0, $CHCl_3$). 98% ee (determined by HPLC: Chiralpak IE Column, 30/70 *i*-PrOH/hexane, 1.0 mL/min, 254 nm; TR = 7.59 min (minor), 13.35 min (major)).

(*R*)-*N,N*-dibenzyl-4-(3'-(2,6-dimethylphenyl)-1'-tosyl-4',6'-dihydro-1'*H*-spiro[cyclohexane-1,5'-cyclopenta[*b*]pyrrol]-4'-yl)aniline ((-)-2f)

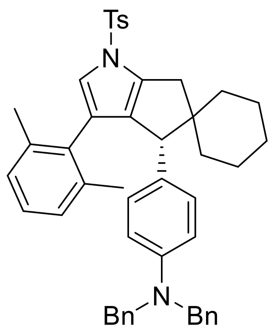

(-)-**2f**

Compound (-)-**2f** was prepared in 39% yield (55.1 mg) according to the general procedure at 30 °C. Pale yellow oil, (eluent: PE/DCM = 2/1). $[\alpha]_D^{25}$ = -123.5° (c = 1.0, $CHCl_3$). 98% ee (determined by HPLC: Chiralpak ODH Column, 3/97 *i*-PrOH/hexane, 1.0 mL/min, 254 nm; TR = 12.39 min (major), 14.61 min (minor)). ^{1}H NMR (500 MHz, $CDCl_3$) δ 7.70 (d, *J* = 8.5 Hz, 2H), 7.31 – 7.26 (m, 4H), 7.25 – 7.22 (m, 4H), 7.15 (d, *J* = 7.0 Hz, 4H), 6.99 – 6.95 (m, 1H), 6.90 (d, *J* = 7.5 Hz, 1H), 6.84 (s, 1H), 6.80 (d, *J* = 7.5 Hz, 1H), 6.41 – 6.24 (m, 4H), 4.49 (q, *J* = 17.0 Hz, 4H), 3.19 (s, 1H), 2.89 (d, *J* = 16.5 Hz, 1H), 2.69 (d, *J* = 16.5 Hz, 1H), 2.30 (s, 3H), 1.94 (s, 3H), 1.67 – 1.59 (m, 1H), 1.56 (s, 3H), 1.53 – 1.31 (m, 5H), 1.23 – 1.10 (m, 3H), 1.01 – 0.91 (m, 1H); ^{13}C NMR (125 MHz, $CDCl_3$) δ 147.4, 144.5, 138.8, 137.0, 137.0, 136.9, 136.8, 136.3, 132.9, 129.6, 129.2, 128.8, 128.4, 126.7(9), 126.7(6), 126.7(4), 126.5, 126.4, 125.2, 121.6, 111.9, 55.7, 54.1, 53.7, 39.0, 37.1, 35.0, 30.3, 26.0, 23.3(1), 23.2(9), 21.4, 20.5, 20.2; IR (neat): 2925, 2852, 1612, 1518, 1494, 1370, 1174, 1101, 730; HRESIMS Calcd for $[C_{47}H_{48}N_2O_2SNa]^+$ (M + Na^+) 727.3329, found 727.3320.

(*R*)-*N*-(1-cyclohexyl-4-(4-(diethylamino)phenyl)but-3-yn-2-yl)-*N*-((2,6-dimethylphenyl)ethynyl)-4-methylbenzenesulfonamide ((+)-1g)

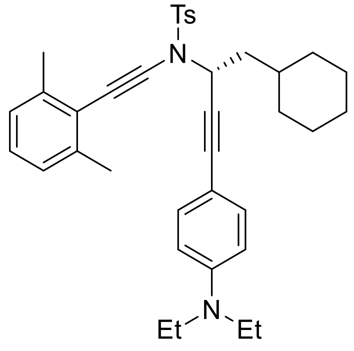

(+)-**1g**

Compound (+)-**1g** was prepared in 40% yield (46.4 mg) according to the general procedure. Pale yellow oil, (eluent: PE/DCM = 2/1). $[\alpha]_D^{25}$ = +41.3º (c = 1.0, $CHCl_3$). 79% ee (determined by HPLC: Chiralpak ADH Column, 5/95 *i*-PrOH/hexane, 1.0 mL/min, 254 nm; TR = 6.86 min (minor), 15.43 min (major)).

(*R*)-4-(3'-(2,6-dimethylphenyl)-1'-tosyl-4',6'-dihydro-1'*H*-spiro[cyclohexane-1,5'-cyclopenta[*b*]pyrrol]-4'-yl)-*N*,*N*-diethylaniline ((-)-2g)

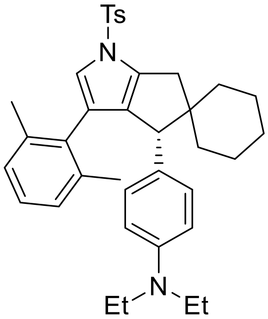

(-)-**2g**

Compound (-)-**2g** was prepared in 39% yield (45.3 mg) according to the general procedure. Pale yellow oil, (eluent: PE/DCM = 2/1). $[\alpha]_D^{25}$ = -56.3º (c = 1.0, $CHCl_3$). 82% ee (determined by HPLC: Chiralpak IE Column, 2/98 *i*-PrOH/hexane, 1.0 mL/min, 254 nm; TR = 8.31 min (minor), 9.13 min (major)). ^{1}H NMR (500 MHz, $CDCl_3$) δ 7.75 (d, *J* = 8.5 Hz, 2H), 7.32 (d, *J* = 8.0 Hz, 2H), 6.97 – 6.88 (m, 2H), 6.86 (s, 1H), 6.78 (d, *J* = 7.5 Hz, 1H), 6.40 (d, *J* = 8.0 Hz, 2H), 6.32 (d, *J* = 9.0 Hz, 2H), 3.30 – 3.13 (m, 5H), 2.90 (d, *J* = 16.0 Hz, 1H), 2.72 (d, *J* = 16.0 Hz, 1H), 2.45 (s, 3H), 1.95 (s, 3H), 1.63 (s, 3H), 1.62 – 1.38 (m, 6H), 1.21 – 1.09 (m, 3H), 1.06 (t, *J* = 7.0 Hz, 6H), 1.00 – 0.91 (m, 1H); ^{13}C NMR (125 MHz, $CDCl_3$) δ 146.2, 144.5, 137.1, 137.0, 136.7, 136.4, 133.0, 129.7, 129.2, 127.7, 126.8(0), 126.7(8), 126.6, 126.4, 125.3, 121.6, 111.8, 55.7, 53.8, 44.4, 38.9, 36.9, 35.1, 26.0, 23.3, 21.6, 20.6, 20.3, 12.4; IR (neat): 2926, 2851, 1612, 1558, 1371, 1175, 1121, 1003, 813; HRESIMS Calcd for $[C_{37}H_{45}N_2O_2S]^+$ (M + H$^+$) 581.3196, found 581.3186.

(*R*)-*N*-(1-cyclohexyl-4-(4-(piperidin-1-yl)phenyl)but-3-yn-2-yl)-*N*-((2,6-dimethylphenyl)ethynyl)-4-methylbenzenesulfonamide ((+)-1h)

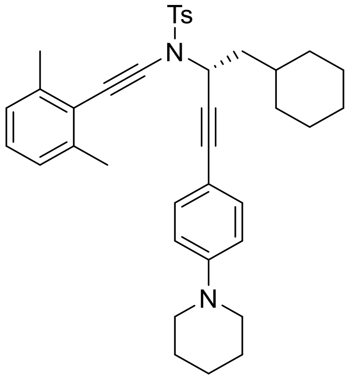

(+)-**1h**

Compound (+)-**1h** was prepared in 47% yield (55.8 mg) according to the general procedure. Pale yellow oil, (eluent: PE/DCM = 2/1). $[\alpha]_D^{25}$ = +68.8º (c = 1.0, $CHCl_3$). 90% ee (determined by HPLC: Chiralpak ADH Column, 5/95 *i*-PrOH/hexane, 1.0 mL/min, 254 nm; TR = 8.35 min (minor), 12.86 min (major)).

(*R*)-3'-(2,6-dimethylphenyl)-4'-(4-(piperidin-1-yl)phenyl)-1'-tosyl-4',6'-dihydro-1'*H*-spiro[cyclohexane-1,5'-cyclopenta[*b*]pyrrole] ((-)-2h)

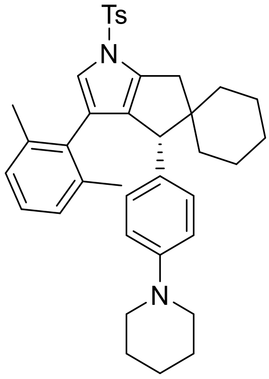

(-)-**2h**

Compound (-)-**2h** was prepared in 46% yield (54.6 mg) according to the general procedure. Pale yellow oil, (eluent: PE/DCM = 2/1). $[\alpha]_D^{25}$ = -63.8º (c = 1.0, $CHCl_3$). 90% ee (determined by HPLC: Chiralpak ADH Column, 5/95 *i*-PrOH/hexane, 1.0 mL/min, 254 nm; TR = 4.64 min (major), 6.04 min (minor)). ^{1}H NMR (500 MHz, $CDCl_3$) δ 7.74 (d, *J* = 8.0 Hz, 2H), 7.32 (d, *J* = 7.5 Hz, 2H), 7.06 – 6.82 (m, 3H), 6.77

(d, J = 7.0 Hz, 1H), 6.57 (d, J = 8.5 Hz, 2H), 6.46 (d, J = 8.0 Hz, 2H), 3.23 (s, 1H), 3.09 – 2.95 (m, 4H), 2.90 (d, J = 16.0 Hz, 1H), 2.72 (d, J = 16.0 Hz, 1H), 2.45 (s, 3H), 1.94 (s, 3H), 1.68 – 1.58 (m, 8H), 1.57 – 1.50 (m, 4H), 1.44 – 1.33 (m, 2H), 1.23 – 0.77 (m, 5H); ^{13}C NMR (125 MHz, $CDCl_3$) δ 150.5, 144.5, 137.2, 137.0, 136.9, 136.4, 136.3, 132.9, 131.4, 129.7, 128.9, 126.9, 126.8, 126.5(4), 126.4(7), 125.2, 121.6, 115.7, 55.8, 53.9, 50.9, 39.0, 36.9, 35.1, 26.0, 25.9, 24.2, 23.3, 21.6, 20.5, 20.3; IR (neat): 2930, 2852, 1610, 1594, 1512, 1371, 1174, 1102, 1051, 811; HRESIMS Calcd for $[C_{38}H_{45}N_2O_2S]^+$ (M + H^+) 593.3196, found 593.3192.

(*R*)-*N*-(1-cyclohexyl-4-(4-morpholinophenyl)but-3-yn-2-yl)-*N*-((2,6-dimethylphenyl)ethynyl)-4-methylbenzenesulfonamide ((+)-1i)

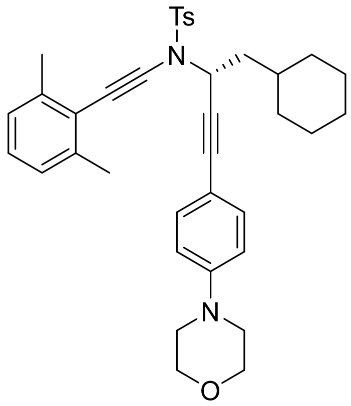

(+)-**1i**

Compound (+)-**1i** was prepared in 38% yield (45.2 mg) according to the general procedure. Pale yellow oil, (eluent: PE/DCM = 2/1). $[\alpha]_D^{25}$ = +23.1° (c = 1.0, $CHCl_3$). 98% ee (determined by HPLC: Chiralpak ADH Column, 20/80 *i*-PrOH/hexane, 1.0 mL/min, 254 nm; TR = 6.98 min (minor), 9.72 min (major)).

(*R*)-4-(4-(3'-(2,6-dimethylphenyl)-1'-tosyl-4',6'-dihydro-1'*H*-spiro[cyclohexane-1,5'-cyclopenta[*b*]pyrrol]-4'-yl)phenyl)morpholine ((-)-2i)

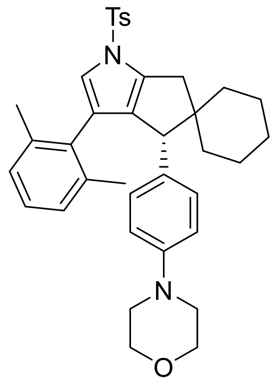

(-)-**2i**

Compound (-)-**2i** was prepared in 37% yield (44.0 mg) according to the general procedure. Pale yellow oil, (eluent: PE/DCM = 2/1). $[\alpha]_D^{25}$ = -29.6º (c = 1.0, $CHCl_3$). 97% ee (determined by HPLC: Chiralpak ADH Column, 5/95 *i*-PrOH/hexane, 1.0 mL/min, 254 nm; TR = 6.89 min (minor), 7.95 min (major)). ^{1}H NMR (500 MHz, $CDCl_3$) δ 7.75 (d, *J* = 8.0 Hz, 2H), 7.32 (d, *J* = 8.0 Hz, 2H), 6.96 – 6.92 (m, 1H), 6.90 (d, *J* = 7.0 Hz, 1H), 6.87 (s, 1H), 6.78 (d, *J* = 7.5 Hz, 1H), 6.61 – 6.46 (m, 4H), 3.89 – 3.72 (m, 4H), 3.25 (s, 1H), 3.08 – 2.95 (m, 4H), 2.91 (d, *J* = 16.0 Hz, 1H), 2.73 (d, *J* = 16.0 Hz, 1H), 2.45 (s, 3H), 1.94 (s, 3H), 1.64 (s, 3H), 1.60 – 1.30 (m, 6H), 1.22 – 1.10 (m, 2H), 1.09 – 1.01 (m, 1H), 0.95 – 0.81 (m, 1H); ^{13}C NMR (125 MHz, $CDCl_3$) δ 149.4 144.5, 137.2, 136.9(3), 136.8(6), 136.4, 136.0, 132.9, 132.2, 129.7, 129.1, 126.8, 126.5, 125.0, 121.6, 114.8, 66.9, 55.8, 53.9, 49.5, 38.9, 36.8, 35.1, 26.0, 23.3, 21.6, 20.5, 20.3; IR (neat): 2925, 2853, 1611, 1513, 1407, 1303, 1174, 1104, 814; HRESIMS Calcd for $[C_{37}H_{42}N_2O_3SNa]^+$ (M + Na^+) 633.2548, found 633.2554.

(*R*)-*N*-(1-cyclohexyl-4-(4-(dimethylamino)-2-methylphenyl)but-3-yn-2-yl)-*N*-((2,6-dimethylphenyl)ethynyl)-4-methylbenzenesulfonamide ((+)-1j)

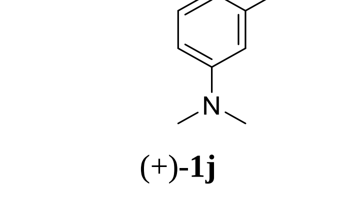

(+)-**1j**

Compound (+)-**1j** was prepared in 42% yield (47.7 mg) according to the general procedure. Pale yellow oil, (eluent: PE/DCM = 2/1). $[\alpha]_D^{25}$ = +53.3º (c = 1.0, $CHCl_3$). 97% ee (determined by HPLC: Chiralpak IE Column, 10/90 *i*-PrOH/hexane, 1.0 mL/min, 254 nm; TR = 9.64 min (minor), 10.58 min (major)).

(*R*)-4-(3'-(2,6-dimethylphenyl)-1'-tosyl-4',6'-dihydro-1'*H*-spiro[cyclohexane-1,5'-cyclopenta[*b*]pyrrol]-4'-yl)-*N*,*N*,3-trimethylaniline ((-)-2j)

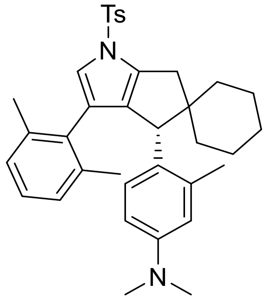

(-)-**2j**

Compound (-)-**2j** was prepared in 44% yield (49.7 mg) according to the general procedure. Pale yellow oil, (eluent: PE/DCM = 2/1). $[\alpha]_D^{25}$ = -41.5º (c = 1.0, $CHCl_3$). 95% ee (determined by HPLC: Chiralpak IE Column, 10/90 *i*-PrOH/hexane, 1.0 mL/min, 254 nm; TR = 7.53 min (minor), 8.89 min (major)). ^{1}H NMR (400 MHz, $CDCl_3$) δ 7.74 (d, *J* = 8.4 Hz, 2H), 7.32 (d, *J* = 8.0 Hz, 2H), 6.98 – 6.89 (m, 2H), 6.87 (s, 1H), 6.79 (d, *J* = 7.2 Hz, 1H), 6.42 (d, *J* = 8.0 Hz, 2H), 6.27 (d, *J* = 8.8 Hz, 2H), 3.21 (s, 1H), 2.90 (d, *J* = 16.4 Hz, 1H), 2.77 – 2.67 (m, 4H), 2.45 (s, 3H), 1.95 (s, 3H), 1.65 (s, 3H), 1.63 – 1.38 (m, 6H), 1.21 – 1.11 (m, 2H), 1.10 – 1.00 (m, 1H), 0.94 – 0.84 (m, 1H); ^{13}C NMR (100 MHz, $CDCl_3$) δ 147.5, 144.5, 137.1, 137.0, 136.5, 136.3, 133.0, 129.7, 129.4, 129.2, 126.9, 126.8, 126.6, 126.5, 125.2, 121.6, 111.6, 55.8, 53.9, 39.0, 36.9, 35.1, 30.8, 26.0, 23.3, 21.6, 20.5, 20.4; IR (neat): 2925, 2853, 1615, 1519, 1488, 1369, 1174, 1125, 1102, 1005, 673; HRESIMS Calcd for $[C_{36}H_{43}N_2O_2S]^+$ (M + H^+) 567.3040, found 567.3050.

(*R*)-*N*-(1-cyclohexyl-4-(4-(dimethylamino)phenyl)but-3-yn-2-yl)-*N*-(mesitylethynyl)-4-methylbenzenesulfonamide ((+)-1k)

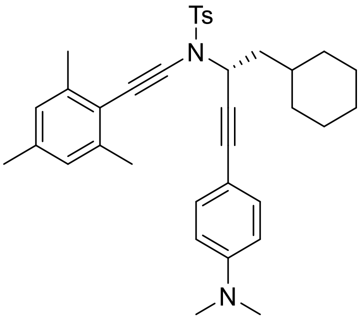

(+)-**1k**

Compound (+)-**1k** was prepared in 42% yield (47.6 mg) according to the general procedure. Pale yellow oil, (eluent: PE/DCM = 2/1). $[\alpha]_D^{25}$ = +32.8º (c = 1.0, $CHCl_3$). 93% ee (determined by HPLC: Chiralpak IE Column, 5/95 *i*-PrOH/hexane, 1.0 mL/min, 254 nm; TR = 17.47 min (minor), 18.78 min (major)).

(*R*)-4-(3'-mesityl-1'-tosyl-4',6'-dihydro-1'*H*-spiro[cyclohexane-1,5'-cyclopenta[*b*]pyrrol]-4'-yl)-*N,N*-dimethylaniline ((-)-2k)

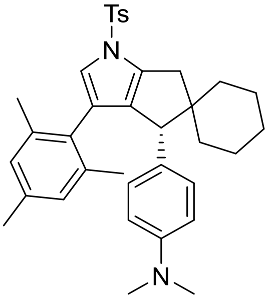

(-)-**2k**

Compound (-)-**2k** was prepared in 40% yield (45.3 mg) according to the general procedure. Pale yellow solid (mp 163–164 ºC) , (eluent: PE/DCM = 2/1). $[\alpha]_D^{25}$ = -58.3º (c = 1.0, $CHCl_3$). 95% ee (determined by HPLC: Chiralpak IE Column, 5/95 *i*-PrOH/hexane, 1.0 mL/min, 254 nm; TR = 10.34 min (minor), 11.63 min (major)). ^{1}H NMR (500 MHz, $CDCl_3$) δ 7.74 (d, *J* = 8.0 Hz, 2H), 7.32 (d, *J* = 8.0 Hz, 2H), 6.85 (s, 1H), 6.73 (s, 1H), 6.61 (s, 1H), 6.46 (d, *J* = 8.0 Hz, 2H), 6.39 (d, *J* = 8.5 Hz, 2H), 3.22 (s, 1H), 2.90 (d, *J* = 16.0 Hz, 1H), 2.83 (s, 6H), 2.69 (d, *J* = 16.0 Hz, 1H), 2.45 (s, 3H), 2.17 (s, 3H), 1.91 (s, 3H), 1.65 – 1.43 (m, 9H), 1.21 – 1.11 (m, 2H), 1.05 – 0.88 (m, 2H); ^{13}C NMR (125 MHz, $CDCl_3$) δ 149.0, 144.5, 137.2, 136.8, 136.5, 136.4, 136.2, 130.0, 129.7, 129.0, 128.8, 127.7, 127.3, 126.5, 125.3, 121.8, 111.9, 55.7, 53.9, 40.7,

38.9, 36.8, 35.2, 26.0, 23.3(3), 23.2(9), 21.6, 20.9, 20.5, 20.2; IR (neat): 2924, 2852, 1613, 1519, 1369, 1186, 1117, 1125, 1010; HRESIMS Calcd for $[C_{36}H_{42}N_2NaO_2S]^+$ (M + Na^+) 589.2859, found 589.2849.

(*R*)-*N*-(1-cyclohexyl-4-(4-(dimethylamino)phenyl)but-3-yn-2-yl)-*N*-((4-fluoro-2,6-dimethylphenyl)ethynyl)-4-methylbenzenesulfonamide ((+)-1l)

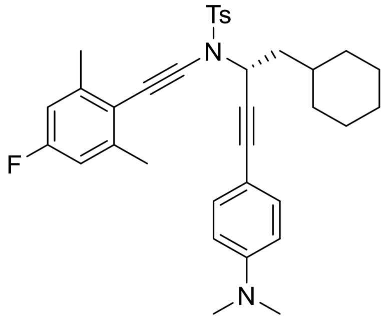

(+)-**1l**

Compound (+)-**1l** was prepared in 42% yield (48.0 mg) according to the general procedure. Pale yellow oil, (eluent: PE/DCM = 2/1). $[\alpha]_D^{25}$ = +43.2º (c = 1.0, $CHCl_3$). 94% ee (determined by HPLC: Chiralpak IE Column, 10/90 *i*-PrOH/hexane, 1.0 mL/min, 254 nm; TR = 10.10 min (minor), 11.41 min (major)).

(*R*)-4-(3'-(4-fluoro-2,6-dimethylphenyl)-1'-tosyl-4',6'-dihydro-1'*H*-spiro[cyclohexane-1,5'-cyclopenta[*b*]pyrrol]-4'-yl)-*N,N*-dimethylaniline ((-)-2l)

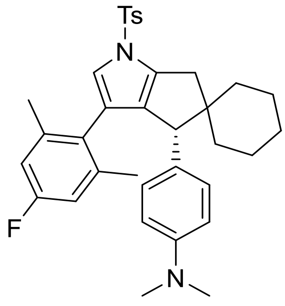

(-)-**2l**

Compound (-)-**2l** was prepared in 40% yield (45.6 mg) according to the general procedure. Pale yellow oli, (eluent: PE/DCM = 2/1). $[\alpha]_D^{25}$ = -14.2º (c = 1.0, $CHCl_3$). 96% ee (determined by HPLC: Chiralpak IE Column, 5/95 *i*-PrOH/hexane, 1.0 mL/min, 254 nm; TR = 9.47 min (minor), 10.30 min (major)). ^1H NMR (500 MHz, $CDCl_3$) δ

7.75 (d, J = 8.5 Hz, 2H), 7.32 (d, J = 8.0 Hz, 2H), 6.85 (s, 1H), 6.64 – 6.58 (m, 1H), 6.51 – 6.47 (m, 1H), 6.45 (d, J = 8.0 Hz, 2H), 6.39 (d, J = 8.5 Hz, 2H), 3.21 (s, 1H), 2.90 (d, J = 16.0 Hz, 1H), 2.83 (s, 6H), 2.73 (d, J = 16.0 Hz, 1H), 2.46 (s, 3H), 1.93 (s, 3H), 1.62 (s, 3H), 1.61 – 1.47 (m, 4H), 1.42 – 1.34 (m, 2H), 1.21 – 1.12 (m, 2H), 1.10 – 1.03 (m, 1H), 0.94 – 0.86 (m, 1H); ^{13}C NMR (150 MHz, $CDCl_3$) δ 161.4 (d, J = 243.9 Hz), 149.0, 144.6, 139.3 (d, J = 8.2 Hz), 139.2 (d, J = 8.1 Hz), 137.2, 136.3(4), 136.2(8), 129.7, 129.0, 128.7 (d, J = 2.8 Hz), 128.5, 126.5, 124.3, 121. 8, 113.3 (d, J = 20.7 Hz), 113.1 (d, J = 20.8 Hz), 111.9, 55.7, 53.9, 40.7, 38.9, 36.9, 35.1, 26.0, 23.3, 23.2, 21.6, 20.6 (d, J = 1.1 Hz), 20.5 (d, J = 0.9 Hz); ^{19}F NMR (376 MHz, $CDCl_3$) δ -117.1 (s); IR (neat): 2925, 2852, 1681, 1612, 1519, 1449, 1370, 1303, 1186, 1174; HRESIMS Calcd for $[C_{35}H_{39}FN_2NaO_2S]^+$ (M + Na^+) 593.2608, found 593.2615.

(*R*)-*N*-((4-chloro-2,6-dimethylphenyl)ethynyl)-*N*-(1-cyclohexyl-4-(4-(dimethylamino)phenyl)but-3-yn-2-yl)-4-methylbenzenesulfonamide ((+)-1m)

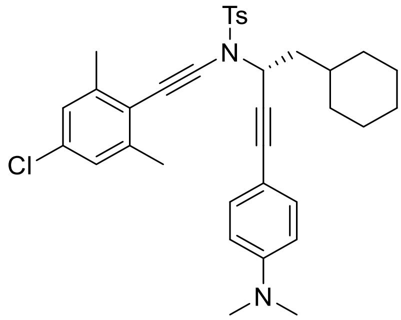

(+)-**1m**

Compound (+)-**1m** was prepared in 40% yield (47.0 mg) according to the general procedure. Pale yellow oil, (eluent: PE/DCM = 2/1). $[\alpha]_D^{25}$ = +22.3° (c = 1.0, $CHCl_3$). 98% ee (determined by HPLC: Chiralpak ADH Column, 5/95 *i*-PrOH/hexane, 1.0 mL/min, 254 nm; TR = 13.96 min (minor), 15.40 min (major)).

(*R*)-4-(3'-(4-chloro-2,6-dimethylphenyl)-1'-tosyl-4',6'-dihydro-1'*H*-spiro[cyclohexane-1,5'-cyclopenta[*b*]pyrrol]-4'-yl)-*N*,*N*-dimethylaniline ((-)-2m)

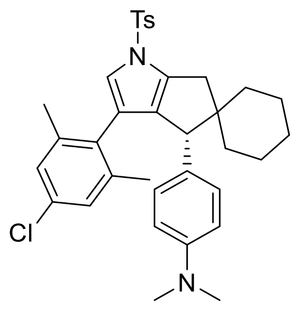

(-)-**2m**

Compound (-)-**2m** was prepared in 39% yield (45.8 mg) according to the general procedure. Pale yellow solid (mp 188–189 ^{o}C) , (eluent: PE/DCM = 2/1). $[\alpha]_D^{25}$ = -21.2^{o} (c = 1.0, $CHCl_3$). 97% ee (determined by HPLC: Chiralpak IE Column, 5/95 *i*-PrOH/hexane, 1.0 mL/min, 254 nm; TR = 9.12 min (minor), 10.04 min (major)). ^{1}H NMR (600 MHz, $CDCl_3$) δ 7.75 (d, *J* = 7.8 Hz, 2H), 7.33 (d, *J* = 8.4 Hz, 2H), 6.90 (s, 1H), 6.85 (s, 1H), 6.78 (s, 1H), 6.45 (d, *J* = 7.2 Hz, 2H), 6.39 (d, *J* = 8.4 Hz, 2H), 3.20 (s, 1H), 2.90 (d, *J* = 16.2 Hz, 1H), 2.83 (s, 6H), 2.72 (d, *J* = 16.2 Hz, 1H), 2.46 (s, 3H), 1.92 (s, 3H), 1.61 (s, 3H), 1.60 – 1.37 (m, 6H), 1.21 – 1.11 (m, 2H), 1.08 – 1.02 (m, 1H), 0.94 – 0.86 (m, 1H); ^{13}C NMR (150 MHz, $CDCl_3$) δ 149.0, 144.6, 138.8(9), 138.8(7), 137.3, 136.3, 136.1, 132.1, 131.6, 129.7, 129.0, 128.5, 126.7, 126.6, 126.4, 124.0, 121.6, 111.9, 55.7, 53.9, 40.7, 38.9, 36.8, 35.1, 26.0, 23.3, 21.6, 20.4, 20.2; IR (neat): 2926, 2854, 1611, 1518, 1447, 1369, 1303, 1173, 1101, 1006, 811; HRESIMS Calcd for $[C_{35}H_{39}ClN_2NaO_2S]^+$ (M + Na^+) 609.2313, found 609.2292.

(*R*)-*N*-((4-bromo-2,6-dimethylphenyl)ethynyl)-*N*-(1-cyclohexyl-4-(4-(dimethylamino)phenyl)but-3-yn-2-yl)-4-methylbenzenesulfonamide ((+)-1n)

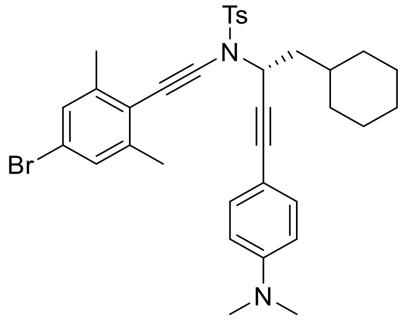

(+)-**1n**

Compound (+)-**1n** was prepared in 40% yield (50.6 mg) according to the general procedure. Pale yellow oil, (eluent: PE/DCM = 2/1). $[\alpha]_D^{25}$ = +43.2º (c = 1.0, $CHCl_3$). 98% ee (determined by HPLC: Chiralpak IE Column, 10/90 *i*-PrOH/hexane, 1.0 mL/min, 254 nm; TR = 10.16 min (minor), 11.15 min (major)).

(*R*)-4-(3'-(4-bromo-2,6-dimethylphenyl)-1'-tosyl-4',6'-dihydro-1'*H*-spiro[cyclohexane-1,5'-cyclopenta[*b*]pyrrol]-4'-yl)-*N*,*N*-dimethylaniline ((-)-2n)

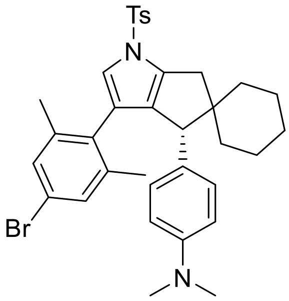

(-)-**2n**

Compound (-)-**2n** was prepared in 39% yield (49.3 mg) according to the general procedure. Pale yellow solid (mp 195–196 ºC) , (eluent: PE/DCM = 2/1). $[\alpha]_D^{25}$ = -32.3º (c = 1.0, $CHCl_3$). 95% ee (determined by HPLC: Chiralpak IC Column, 5/95 *i*-PrOH/hexane, 1.0 mL/min, 254 nm; TR = 8.78 min (minor), 10.08 min (major)). ^{1}H NMR (400 MHz, $CDCl_3$) δ 7.75 (d, *J* = 8.4 Hz, 2H), 7.32 (d, *J* = 8.0 Hz, 2H), 7.05 (s, 1H), 6.93 (s, 1H), 6.85 (s, 1H), 6.45 (d, *J* = 8.4 Hz, 2H), 6.39 (d, *J* = 8.8 Hz, 2H), 3.20 (s, 1H), 2.90 (d, *J* = 16.0 Hz, 1H), 2.83 (s, 6H), 2.71 (d, *J* = 16.0 Hz, 1H), 2.45 (s, 3H), 1.92 (s, 3H), 1.61 (s, 3H), 1.59 – 1.34 (m, 6H), 1.21 – 0.86 (m, 4H); ^{13}C NMR (100 MHz, $CDCl_3$) δ 149.1, 144.6, 139.2, 137.3, 136.4, 136.0, 132.1, 129.7, 129.6, 129.3, 129.0, 128.4, 126.6, 124.0, 121.5, 120.5, 111.9, 55.7, 53.9, 40.7, 39.0, 36.9, 35.1, 26.0, 23.3, 21.6, 20.3, 20.2; IR (neat): 2925, 2853, 1615, 1516, 1488, 1450, 1369, 1269, 1174, 1102, 673; HRESIMS Calcd for $[C_{35}H_{39}BrN_2NaO_2S]^+$ (M + Na^+) 653.1808, found 653.1800.

(*R*)-*N*-(1-cyclohexyl-4-(4-(dimethylamino)phenyl)but-3-yn-2-yl)-*N*-((3,5-dimethyl-[1,1'-biphenyl]-4-yl)ethynyl)-4-methylbenzenesulfonamide ((+)-1o)

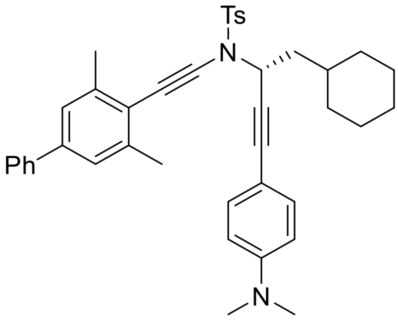

(+)-**1o**

Compound (+)-**1o** was prepared in 40% yield (50.3 mg) according to the general procedure. Pale yellow oil, (eluent: PE/DCM = 2/1). $[\alpha]_D^{25}$ = +100.8º (c = 1.0, $CHCl_3$). 97% ee (determined by HPLC: Chiralpak ADH Column, 10/90 *i*-PrOH/hexane, 1.0 mL/min, 254 nm; TR = 7.49 min (minor), 10.38 min (major)).

(*R*)-4-(3'-(3,5-dimethyl-[1,1'-biphenyl]-4-yl)-1'-tosyl-4',6'-dihydro-1'*H*-spiro[cyclohexane-1,5'-cyclopenta[*b*]pyrrol]-4'-yl)-*N,N*-dimethylaniline ((-)-2o)

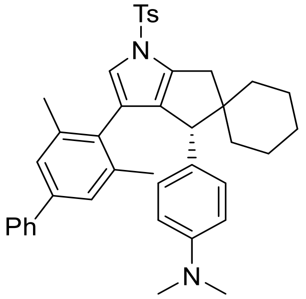

(-)-**2o**

Compound (-)-**2o** was prepared in 42% yield (52.8 mg) according to the general procedure. Pale yellow oil, (eluent: PE/DCM = 2/1). $[\alpha]_D^{25}$ = -100.6º (c = 1.0, $CHCl_3$). 95% ee (determined by HPLC: Chiralpak IC Column, 5/95 *i*-PrOH/hexane, 1.0 mL/min, 254 nm; TR = 4.05 min (major), 8.67 min (minor)). ^{1}H NMR (850 MHz, $CDCl_3$) δ 7.76 (d, *J* = 7.7 Hz, 2H), 7.51 (d, *J* = 7.7 Hz, 2H), 7.38 – 7.35 (m, 2H), 7.33 (d, *J* = 7.7 Hz, 2H), 7.30 – 7.29 (m, 1H), 7.15 (s, 1H), 7.03 (s, 1H), 6.91 (s, 1H), 6.48 (d, *J* = 6.8 Hz, 2H), 6.40 (d, *J* = 8.5 Hz, 2H), 3.27 (s, 1H), 2.92 (d, *J* = 16.2 Hz, 1H), 2.82 (s, 6H), 2.72 (d, *J* = 16.2 Hz, 1H), 2.47 (s, 3H), 2.02 (s, 3H), 1.72 (s, 3H), 1.66 – 1.47 (m, 5H), 1.45 – 1.37 (m, 2H), 1.19 – 1.16 (m, 1H), 1.10 – 1.02 (m, 1H), 0.95 – 0.90 (m, 1H); ^{13}C NMR (213 MHz, $CDCl_3$) δ 149.0, 144.6, 141.1, 139.5, 137.4, 137.3, 136.3(9), 136.3(5),

132.2, 129.7, 129.1, 128.6, 128.5, 126.9(3), 126.9(1), 126.6, 125.7, 125.3, 125.0, 121.7, 111.9, 55.8, 54.0, 40.7, 38.9, 36.7, 35.1, 26.0, 23.3, 21.6, 20.7, 20.6; IR (neat): 2930, 2852, 1610, 1506, 1464, 1371, 1265, 1128, 1102, 672, 588; HRESIMS Calcd for $[C_{41}H_{44}N_2NaO_2S]^+$ (M + Na^+) 651.3016, found 651.3020.

(*R*)-*N*-(1-cyclohexyl-4-(4-(dimethylamino)phenyl)but-3-yn-2-yl)-*N*-((2,6-dimethyl-4-((trimethylsilyl)ethynyl)phenyl)ethynyl)-4-methylbenzenesulfonamide ((+)-1p)

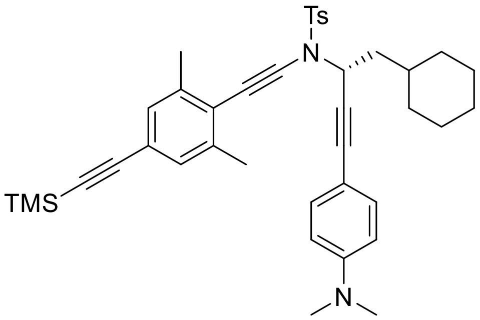

(+)-**1p**

Compound (+)-**1p** was prepared in 42% yield (54.5 mg) according to the general procedure. Pale yellow oil, (eluent: PE/DCM = 2/1). $[\alpha]_D^{25}$ = +27.5º (c = 1.0, $CHCl_3$). 97% ee (determined by HPLC: Chiralpak ADH Column, 5/95 *i*-PrOH/hexane, 1.0 mL/min, 254 nm; TR = 5.21 min (major), 6.66 min (minor)).

(*R*)-4-(3'-(2,6-dimethyl-4-((trimethylsilyl)ethynyl)phenyl)-1'-tosyl-4',6'-dihydro-1'*H*-spiro[cyclohexane-1,5'-cyclopenta[*b*]pyrrol]-4'-yl)-*N*,*N*-dimethylaniline ((-)-2p)

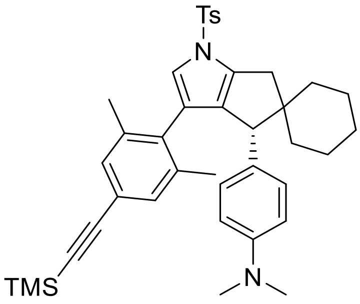

(-)-**2p**

Compound (-)-**2p** was prepared in 42% yield (54.5 mg) according to the general procedure. Pale yellow oil, (eluent: PE/DCM = 2/1). $[\alpha]_D^{25}$ = -16.5º (c = 1.0, $CHCl_3$). 95% ee (determined by HPLC: Chiralpak ADH Column, 5/95 *i*-PrOH/hexane, 1.0 mL/min, 254 nm; TR = 4.02 min (major), 9.18 min (minor)). ^{1}H NMR (500 MHz, $CDCl_3$) δ 7.75 (d, *J* = 8.5 Hz, 2H), 7.33 (d, *J* = 8.0 Hz, 2H), 7.04 (s, 1H), 6.92 (s, 1H), 6.86 (s, 1H), 6.42 (d, *J* = 8.0 Hz, 2H), 6.37 (d, *J* = 8.5 Hz, 2H), 3.19 (s, 1H), 2.89 (d, *J* = 16.5 Hz, 1H), 2.83 (s, 6H), 2.71 (d, *J* = 16.5 Hz, 1H), 2.46 (s, 3H), 1.92 (s, 3H), 1.61 (s, 3H), 1.60 – 1.38 (m, 6H), 1.21 – 1.01 (m, 3H), 0.92 – 0.81 (m, 1H), 0.21 (s, 9H); ^{13}C NMR (125 MHz, $CDCl_3$) δ 149.0, 144.6, 137.2, 137.1, 137.1, 136.4, 136.1, 133.9, 130.4, 130.0, 129.7, 129.0, 128.5, 126.6, 124.5, 121.5, 121.1, 111.9, 105.5, 93.2, 55.8, 53.9, 40.7, 39.0, 36.9, 35.1, 26.0, 23.3, 21.6, 20.3, 20.2, 0.0; IR (neat): 2927, 2851, 2150(s), 1614, 1519, 1372, 1174, 843, 813; HRESIMS Calcd for $[C_{40}H_{48}KN_2O_2SSi]^+$ (M + K^+) 687.2837, found 687.2820.

(*R*)-*N*-(1-cyclohexyl-4-(4-(dimethylamino)phenyl)but-3-yn-2-yl)-*N*-((2,6-dimethoxyphenyl)ethynyl)-4-methylbenzenesulfonamide ((+)-1q)

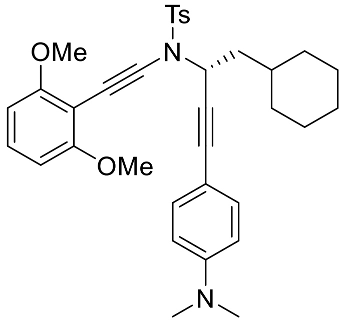

(+)-**1q**

Compound (+)-**1q** was prepared in 46% yield (53.8 mg) according to the general procedure except at 30 °C. Pale yellow oil, (eluent: PE/EtOAc = 10/1). $[\alpha]_D^{25}$ = +27.2º (c = 1.0, $CHCl_3$). 54% ee (determined by HPLC: Chiralpak IE Column, 20/80 *i*-PrOH/hexane, 1.0 mL/min, 254 nm; TR = 22.91 min (major), 28.79 min (minor)).

(*R*)-4-(3'-(2,6-dimethoxyphenyl)-1'-tosyl-4',6'-dihydro-1'*H*-spiro[cyclohexane-1,5'-cyclopenta[*b*]pyrrol]-4'-yl)-*N*,*N*-dimethylaniline ((-)-2q)

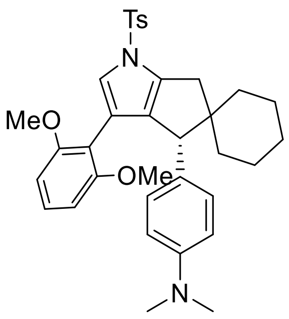

(-)-**2q**

Compound (-)-**2q** was prepared in 42% yield (49.0 mg) according to the general procedure except at 30 °C. Pale yellow oil, (eluent: PE/EtOAc = 10/1). $[\alpha]_D^{25}$ = -6.8° (c = 1.0, $CHCl_3$). 60% ee (determined by HPLC: Chiralpak IE Column, 30/70 *i*-PrOH/hexane, 1.0 mL/min, 254 nm; TR = 12.29 min (major), 17.84 min (minor)). ^{1}H NMR (850 MHz, $CDCl_3$) δ 7.79 (d, *J* = 8.5 Hz, 2H), 7.33 – 7.28 (m, 3H), 6.97 (t, *J* = 7.7 Hz, 1H), 6.67 – 6.44 (m, 2H), 6.42 – 6.33 (m, 2H), 6.32 (d, *J* = 8.5 Hz, 2H), 3.67 (s, 1H), 3.57 (s, 6H), 2.84 (d, *J* = 15.3 Hz, 1H), 2.79 (s, 6H), 2.75 (d, *J* = 15.3 Hz, 1H), 2.43 (s, 3H), 1.79 – 1.74 (m, 1H), 1.60 – 1.38 (m, 6H), 1.06 – 0.91 (m, 2H), 0.63 – 0.51 (m, 1H); ^{13}C NMR (214 MHz, $CDCl_3$) δ 157.5, 148.8, 144.2, 136.8, 135.8, 134.7, 129.7, 129.1, 128.6, 127.9, 126.7, 124.2, 117.0, 111.8, 111.6, 103.7, 57.6, 55.6, 53.9, 40.9, 38.4, 36.3, 33.8, 26.0, 23.7, 23.0, 21.6; IR (neat): 2926, 2851, 1597, 1519, 1473, 1366, 1246, 1173, 1112, 813; HRESIMS Calcd for $[C_{35}H_{40}N_2NaO_4S]^+$ (M + Na^+) 607.2601, found 607.2620.

(*R*)-*N*-(1-cyclohexyl-4-(4-(dimethylamino)phenyl)but-3-yn-2-yl)-4-methyl-*N*-(*o*-tolylethynyl)benzenesulfonamide ((+)-1r)

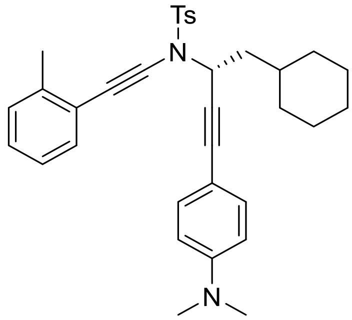

(+)-**1r**

Compound (+)-**1r** was prepared in 36% yield (38.7 mg) according to the general procedure. Pale yellow oil, (eluent: PE/DCM = 2/1). $[\alpha]_D^{25}$ = +105.6º (c = 1.0, $CHCl_3$). 97% ee (determined by HPLC: Chiralpak ODH Column, 3/97 *i*-PrOH/hexane, 1.0 mL/min, 254 nm; TR = 7.72 min (major), 8.74 min (minor)).

(*R*)-*N,N*-dimethyl-4-(3'-(*o*-tolyl)-1'-tosyl-4',6'-dihydro-1'*H*-spiro[cyclohexane-1,5'-cyclopenta[*b*]pyrrol]-4'-yl)aniline ((-)-2r)

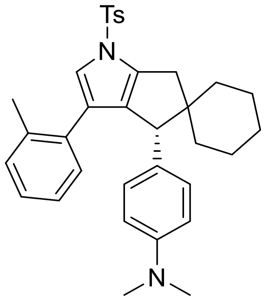

(-)-**2r**

Compound (-)-**2r** was prepared in 35% yield (37.7 mg) according to the general procedure. Pale yellow oil, (eluent: PE/DCM = 2/1). $[\alpha]_D^{25}$ = -78.3º (c = 1.0, $CHCl_3$). 92% ee (determined by HPLC: Chiralpak IE Column, 10/90 *i*-PrOH/hexane, 1.0 mL/min, 254 nm; TR = 10.04 min (minor), 12.59 min (major)). ^{1}H NMR (500 MHz, $CDCl_3$) δ 7.77 (d, *J* = 8.0 Hz, 2H), 7.32 (d, *J* = 8.0 Hz, 2H), 7.09 (s, 1H), 7.05 (d, *J* = 7.5 Hz, 1H), 7.03 – 6.96 (m, 1H), 6.93 – 6.86 (m, 1H), 6.86 – 6.80 (m, 1H), 6.56 (d, *J* = 7.0 Hz, 2H), 6.45 (d, *J* = 9.0 Hz, 2H), 3.53 (s, 1H), 2.88 (d, *J* = 16.0 Hz, 1H), 2.84 (s, 6H), 2.71 (d, *J* = 16.0 Hz, 1H), 2.44 (s, 3H), 2.21 (s, 3H), 1.69 – 1.39 (m, 7H), 1.20 – 1.15 (m, 1H), 1.06 – 0.97 (m, 1H), 0.90 – 0.85 (m, 1H); ^{13}C NMR (150 MHz, $CDCl_3$) δ 148.9, 144.6, 136.9, 136.3, 135.4, 134.6, 133.1, 130.1, 129.8, 129.5, 129.1, 128.9, 126.6(2), 126.5(7), 125.9, 125.3, 121.9, 112.1, 56.4, 53.9, 40.7, 38.9, 36.3, 35.3, 26.0, 23.4, 23.3, 21.6, 21.1; IR (neat): 2925, 2852, 1679, 1612, 1519, 1449, 1370, 1186, 1174, 1133; HRESIMS Calcd for $[C_{34}H_{38}N_2NaO_2S]^+$ (M + Na^+) 561.2546, found 561.2556.

(*R*)-*N*-(1-cyclohexyl-4-(4-(dimethylamino)phenyl)but-3-yn-2-yl)-*N*-((2-fluorophenyl)ethynyl)-4-methylbenzenesulfonamide ((+)-1s)

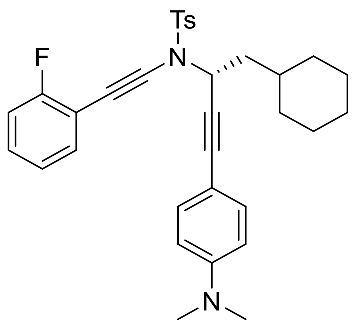

(+)-**1s**

Compound (+)-**1s** was prepared in 39% yield (42.4 mg) according to the general procedure employing **L8**. Pale yellow oil, (eluent: PE/DCM = 2/1). $[\alpha]_D^{25}$ = +44.1° (c = 1.0, $CHCl_3$). 86% ee (determined by HPLC: Chiralpak ADH Column, 5/95 *i*-PrOH/hexane, 1.0 mL/min, 254 nm; TR = 11.54 min (major), 17.18 min (minor)).

(*R*)-4-(3'-(2-fluorophenyl)-1'-tosyl-4',6'-dihydro-1'*H*-spiro[cyclohexane-1,5'-cyclopenta[*b*]pyrrol]-4'-yl)-*N,N*-dimethylaniline ((-)-2s)

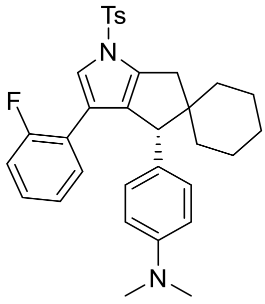

(-)-**2s**

Compound (-)-**2s** was prepared in 40% yield (43.4 mg) according to the general procedure employing **L8**. Pale yellow oil, (eluent: PE/DCM = 2/1). $[\alpha]_D^{25}$ = -66.5° (c = 1.0, $CHCl_3$). 84% ee (determined by HPLC: Chiralpak IE Column, 10/90 *i*-PrOH/hexane, 1.0 mL/min, 254 nm; TR = 11.99 min (major), 14.84 min (minor)). ^{1}H NMR (500 MHz, $CDCl_3$) δ 7.78 (d, *J* = 8.5 Hz, 2H), 7.60 (d, *J* = 3.0 Hz, 1H), 7.32 (d, *J* = 8.0 Hz, 2H), 7.16 – 7.14 (m, 1H), 7.05 – 6.98 (m, 1H), 6.98 – 6.93 (m, 1H), 7.86 – 7.80 (m, 1H), 6.79 – 6.60 (m, 2H), 6.57 – 6.49 (m, 2H), 3.71 (s, 1H), 2.90 (d, *J* = 16.0 Hz, 1H), 2.85 (s, 6H), 2.67 (d, *J* = 16.0 Hz, 1H), 2.42 (s, 3H), 1.65 – 1.43 (m, 6H), 1.22 – 1.14 (m, 2H), 0.99 – 0.81 (m, 2H); ^{13}C NMR (100 MHz, $CDCl_3$) δ 159.7 (d, *J* = 249.2 Hz), 149.1, 144.7, 137.3, 136.2, 132.7, 129.9, 129.1 (d, *J* = 3.9 Hz), 128.5, 127.4 (d, *J*

= 8.5 Hz), 126.7, 123.8 (d, *J* = 3.2 Hz), 123.2 (d, *J* = 15.4 Hz), 121.3 (d, *J* = 12.7 Hz), 119.4 (d, *J* = 1.5 Hz), 115.5 (d, *J* = 22.7 Hz), 112.2, 57.2, 53.7, 40.6, 39.1, 36.0, 35.8, 26.0, 23.4, 23.3, 21.6; ^{19}F NMR (376 MHz, $CDCl_3$) δ -111.9 – -112.2 (m); IR (neat): 2925, 2853, 1615, 1519, 1488, 1369, 1174, 1102, 1005, 673; HRESIMS Calcd for $[C_{33}H_{35}FN_2NaO_2S]^+$ (M + Na^+) 565.2295, found 565.2289.

(*R*)-*N*-((2-chlorophenyl)ethynyl)-*N*-(1-cyclohexyl-4-(4-(dimethylamino)phenyl)but-3-yn-2-yl)-4-methylbenzenesulfonamide ((+)-1t)

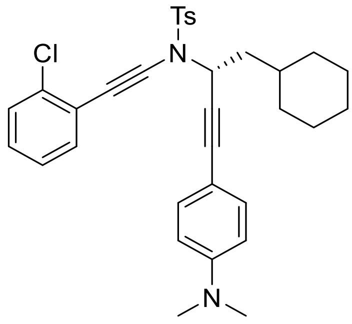

(+)-**1t**

Compound (+)-**1t** was prepared in 37% yield (41.4 mg) according to the general procedure employing **L8**. Pale yellow oil, (eluent: PE/DCM = 2/1). $[\alpha]_D^{25}$ = +24.1° (c = 1.0, $CHCl_3$). 95% ee (determined by HPLC: Chiralpak IE Column, 10/90 *i*-PrOH/hexane, 1.0 mL/min, 254 nm; TR = 15.44 min (major), 15.99 min (minor)).

(*R*)-4-(3'-(2-chlorophenyl)-1'-tosyl-4',6'-dihydro-1'*H*-spiro[cyclohexane-1,5'-cyclopenta[*b*]pyrrol]-4'-yl)-*N*,*N*-dimethylaniline ((-)-2t)

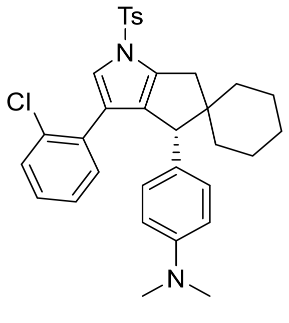

(-)-**2t**

Compound (-)-**2t** was prepared in 44% yield (49.2 mg) according to the general procedure employing **L8**. Pale yellow oil, (eluent: PE/DCM = 2/1). $[\alpha]_D^{25}$ = -19.5° (c =

1.0, $CHCl_3$). 86% ee (determined by HPLC: Chiralpak IE Column, 10/90 *i*-PrOH/hexane, 1.0 mL/min, 254 nm; TR = 10.27 min (minor), 11.77 min (major)). ^{1}H NMR (600 MHz, $CDCl_3$) δ 7.78 (d, *J* = 8.4 Hz, 2H), 7.45 (dd, *J* = 7.8, 0.6 Hz, 1H), 7.40 (s, 1H), 7.33 (d, *J* = 8.4 Hz, 2H), 6.99 – 6.96 (m, 1H), 6.96 – 6.90 (m, 1H), 6.88 (dd, *J* = 7.8, 1.8 Hz, 1H), 6.68 – 6.50 (m, 2H), 6.45 (d, *J* = 8.4 Hz, 2H), 3.63 (s, 1H), 2.86 – 2.81 (m, 7H), 2.73 (d, *J* = 15.6 Hz, 1H), 2.44 (s, 3H), 1.66 – 1.49 (m, 4H), 1.48 – 1.40 (m, 2H), 1.23 – 1.09 (m, 2H), 1.03 – 0.97 (m, 1H), 0.89 – 0.83 (m, 1H); ^{13}C NMR (150 MHz, $CDCl_3$) δ 149.0, 144.7, 136.8, 136.3, 134.5, 134.1, 133.0, 131.1, 129.9, 129.2, 128.7, 127.9, 126.8, 126.7, 124.9, 122.9, 122.5, 112.0, 56.4, 54.0, 40.6, 38.8, 36.4, 35.1, 26.0, 23.4, 23.3, 21.6; IR (neat): 2925, 2853, 1611, 1514, 1450, 1370, 1303, 1174, 1122, 931; HRESIMS Calcd for $[C_{33}H_{35}ClN_2KO_2S]^+$ ($M + K^+$) 597.1739, found 597.1733.

(*R*)-*N*-((2-bromophenyl)ethynyl)-*N*-(1-cyclohexyl-4-(4-(dimethylamino)phenyl)but-3-yn-2-yl)-4-methylbenzenesulfonamide ((+)-1u)

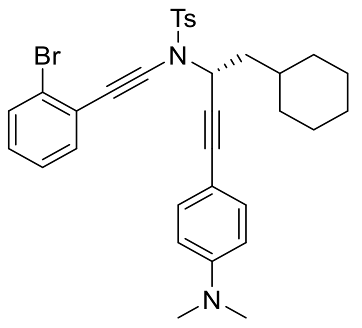

(+)-**1u**

Compound (+)-**1u** was prepared in 37% yield (44.7 mg) according to the general procedure employing **L8**. Pale yellow oil, (eluent: PE/DCM = 2/1). $[\alpha]_D^{25}$ = +152.3º (c = 1.0, $CHCl_3$). 98% ee (determined by HPLC: Chiralpak ADH Column, 5/95 *i*-PrOH/hexane, 1.0 mL/min, 254 nm; TR = 11.54 min (minor), 20.50 min (major)).

(*R*)-4-(3'-(2-bromophenyl)-1'-tosyl-4',6'-dihydro-1'*H*-spiro[cyclohexane-1,5'-cyclopenta[*b*]pyrrol]-4'-yl)-*N*,*N*-dimethylaniline ((-)-2u)

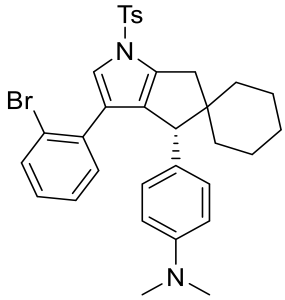

(-)-**2u**

Compound (-)-**2u** was prepared in 40% yield (48.3 mg) according to the general procedure employing **L8**. Pale yellow oil, (eluent: PE/DCM = 2/1). $[\alpha]_D^{25}$ = -55.3° (c = 1.0, $CHCl_3$). 90% ee (determined by HPLC: Chiralpak IE Column, 10/90 *i*-PrOH/hexane, 1.0 mL/min, 254 nm; TR = 10.35 min (minor), 12.24 min (major)). ^{1}H NMR (500 MHz, $CDCl_3$) δ 7.78 (d, *J* = 8.5 Hz, 2H), 7.44 (dd, *J* = 8.0, 1.0 Hz, 1H), 7.40 (s, 1H), 7.32 (d, *J* = 8.0 Hz, 2H), 6.97 (t, *J* = 9.0 Hz, 1H), 6.94 – 6.85 (m, 2H), 6.67 – 6.51 (m, 2H), 6.45 (d, *J* = 9.0 Hz, 2H), 3.63 (s, 1H), 2.91 – 2.85 (m, 1H), 2.84 (s, 6H), 2.73 (d, *J* = 16.0 Hz, 1H), 2.43 (s, 3H), 1.67 – 1.39 (m, 6H), 1.23 – 1.11 (m, 2H), 1.04 – 0.96 (m, 1H), 0.92 – 0.84 (m, 1H); ^{13}C NMR (125 MHz, $CDCl_3$) δ 149.0, 144.7, 136.8, 136.3, 134.5, 134.1, 133.0, 131.1, 129.9, 129.2, 128.7, 127.9, 126.8, 126.7, 124.9, 122.9, 122.5, 112.0, 56.4, 54.0, 40.6, 38.8, 36.4, 35.1, 26.0, 23.4, 23.3, 21.6; IR (neat): 2930, 2852, 1610, 1558, 1512, 1372, 1265, 1109, 1051, 811; HRESIMS Calcd for $[C_{33}H_{35}BrN_2NaO_2S]^+$ (M + Na^+) 625.1495, found 625.1488.

(*R*)-*N*-(1-cyclohexyl-4-(4-(dimethylamino)phenyl)but-3-yn-2-yl)-4-methyl-*N*-(*m*-tolylethynyl)benzenesulfonamide ((+)-1v)

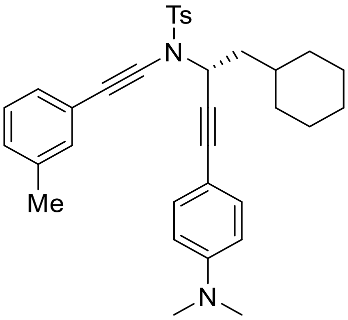

(+)-**1v**

Compound (+)-**1v** was prepared in 33% yield (35.6 mg) according to the general procedure. Pale yellow oil, (eluent: PE/DCM = 2/1). $[\alpha]_D^{25}$ = +76.6º (c = 1.0, $CHCl_3$). 91% ee (determined by HPLC: Chiralpak ADH Column, 10/90 *i*-PrOH/hexane, 1.0 mL/min, 254 nm; TR = 5.06 min (minor), 9.31 min (major)).

(*R*)-*N,N*-dimethyl-4-(3'-(*m*-tolyl)-1'-tosyl-4',6'-dihydro-1'*H*-spiro[cyclohexane-1,5'-cyclopenta[*b*]pyrrol]-4'-yl)aniline ((-)-2v)

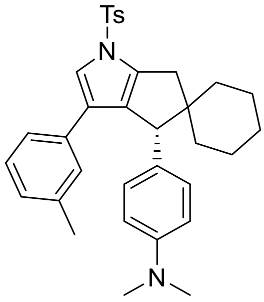

(-)-**2v**

Compound (-)-**2v** was prepared in 31% yield (33.4 mg) according to the general procedure. Pale yellow oil, (eluent: PE/DCM = 2/1). $[\alpha]_D^{25}$ = -11. 2º (c = 1.0, $CHCl_3$). 91% ee (determined by HPLC: Chiralpak IE Column, 10/90 *i*-PrOH/hexane, 1.0 mL/min, 254 nm; TR = 17.34 min (major), 21.14 min (minor)). ^{1}H NMR (500 MHz, $CDCl_3$) δ 7.76 (d, *J* = 8.5 Hz, 2H), 7.37 (s, 1H), 7.31 (d, *J* = 8.0 Hz, 2H), 7.04 – 6.98 (m, 3H), 6.91 – 6.86 (m, 1H), 6.84 – 6.39 (m, 4H), 3.68 (s, 1H), 2.89 (d, *J* = 16.5 Hz, 1H), 2.86 (s, 6H), 2.65 (d, *J* = 16.0 Hz, 1H), 2.43 (s, 3H), 2.16 (s, 3H), 1.63 – 1.43 (m, 6H), 1.22 – 1.15 (m, 2H), 1.08 – 0.94 (m, 2H); ^{13}C NMR (150 MHz, $CDCl_3$) δ 149.1, 144.7, 137.8, 137.8, 136.3, 133.3(1), 132.9(7), 129.9, 128.3, 127.1, 127.0, 126.7, 126.1, 123.0, 119.6, 112.3, 56.7, 53.7, 40.7, 39.2, 36.3, 35.9, 26.0, 23.5, 23.3, 21.6, 21.3; IR (neat): 2929, 2852, 1610, 1558, 1512, 1371, 1265, 1051, 1026, 768; HRESIMS Calcd for $[C_{34}H_{38}N_2NaO_2S]^+$ (M + Na^+) 561.2546, found 561.2546.

(*R*)-*N*-(1-cyclohexyl-4-(4-(dimethylamino)phenyl)but-3-yn-2-yl)-*N*-((3-fluorophenyl)ethynyl)-4-methylbenzenesulfonamide ((+)-1w)

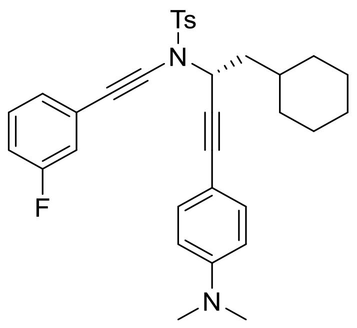

(+)-**1w**

Compound (+)-**1w** was prepared in 39% yield (42.4 mg) according to the general procedure. Pale yellow oil, (eluent: PE/DCM = 2/1). $[\alpha]_D^{25}$ = +17.5º (c = 1.0, $CHCl_3$). 82% ee (determined by HPLC: Chiralpak ADH Column, 10/90 *i*-PrOH/hexane, 1.0 mL/min, 254 nm; TR = 5.97 min (minor), 8.86 min (major)).

(*R*)-4-(3'-(3-fluorophenyl)-1'-tosyl-4',6'-dihydro-1'*H*-spiro[cyclohexane-1,5'-cyclopenta[*b*]pyrrol]-4'-yl)-*N,N*-dimethylaniline ((-)-2w)

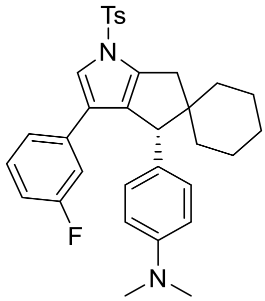

(-)-**2w**

Compound (-)-**2w** was prepared in 41% yield (44.5 mg) according to the general procedure. Pale yellow oil, (eluent: PE/DCM = 2/1). $[\alpha]_D^{25}$ = -11.7º (c = 1.0, $CHCl_3$). 82% ee (determined by HPLC: Chiralpak ADH Column, 10/90 *i*-PrOH/hexane, 1.0 mL/min, 254 nm; TR = 6.10 min (major), 10.73 min (minor)). ^{1}H NMR (500 MHz, $CDCl_3$) δ 7.78 (d, *J* = 8.5 Hz, 2H), 7.39 (s, 1H), 7.33 (d, *J* = 8.0 Hz, 2H), 7.11 – 7.05 (m, 1H), 7.00 (d, *J* = 8.0 Hz, 1H), 6.93 – 6.87 (m, 1H), 6.87 – 6.25 (m, 5H), 3.67 (s, 1H), 2.94 – 2.84 (m, 7H), 2.65 (d, *J* = 16.0 Hz, 1H), 2.43 (s, 3H), 1.69 – 1.40 (m, 7H), 1.19 – 0.96 (m, 3H); ^{13}C NMR (100 MHz, $CDCl_3$) δ 162.9 (d, *J* = 244.6 Hz), 149.2, 144.9, 138.1, 136.2, 135.8 (d, *J* = 8.3 Hz), 132.5, 130.0, 129.7 (d, *J* = 8.5 Hz), 129.2, 128.5, 126.7, 124.9 (d, *J* = 2.5 Hz), 121.6 (d, *J* = 2.7 Hz), 120.0, 113.1 (d, *J* = 19.9 Hz),

112.9 (d, J = 21.1 Hz), 112.3, 56.6, 53.8, 40.6, 39.2, 36.3, 35.8, 26.0, 23.5, 23.3, 21.6; ^{19}F NMR (376 MHz, $CDCl_3$) δ -113.3 – -113.5 (m); IR (neat): 2925, 2853, 1611, 1513, 1370, 1303, 1174, 1122, 1051, 672; HRESIMS Calcd for $[C_{33}H_{35}FN_2NaO_2S]^+$ (M + Na^+) 565.2295, found 565.2287.

(*R*)-*N*-((3-chlorophenyl)ethynyl)-*N*-(1-cyclohexyl-4-(4-(dimethylamino)phenyl)but-3-yn-2-yl)-4-methylbenzenesulfonamide ((+)-1x)

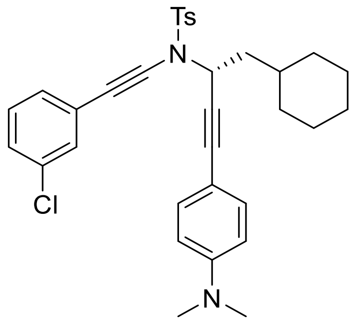

(+)-**1x**

Compound (+)-**1x** was prepared in 40% yield (44.7 mg) according to the general procedure. Pale yellow oil, (eluent: PE/DCM = 2/1). $[\alpha]_D^{25}$ = +8.3º (c = 1.0, $CHCl_3$). 95% ee (determined by HPLC: Chiralpak ADH Column, 10/90 *i*-PrOH/hexane, 1.0 mL/min, 254 nm; TR = 6.15 min (minor), 8.97 min (major)).

(*R*)-4-(3'-(3-chlorophenyl)-1'-tosyl-4',6'-dihydro-1'*H*-spiro[cyclohexane-1,5'-cyclopenta[*b*]pyrrol]-4'-yl)-*N,N*-dimethylaniline ((-)-2x)

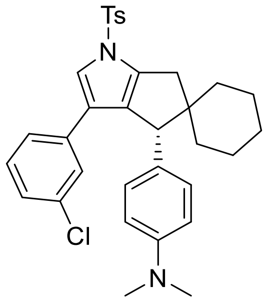

(-)-**2x**

Compound (-)-**2x** was prepared in 42% yield (47.0 mg) according to the general procedure. Pale yellow oil, (eluent: PE/DCM = 2/1). $[\alpha]_D^{25}$ = -19.3º (c = 1.0, $CHCl_3$). 90% ee (determined by HPLC: Chiralpak ADH Column, 10/90 *i*-PrOH/hexane, 1.0

mL/min, 254 nm; TR = 5.75 min (major), 9.71 min (minor)). ^{1}H NMR (400 MHz, $CDCl_3$) δ 7.77 (d, *J* = 8.4 Hz, 2H), 7.37 (s, 1H), 7.33 (d, *J* = 8.0 Hz, 2H), 7.21 – 7.15 (m, 1H), 7.12 – 6.95 (m, 3H), 6.94 – 6.35 (m, 4H), 3.67 (s, 1H), 2.90 (d, *J* = 16.4 Hz, 1H), 2.87 (s, 6H), 2.67 (d, *J* = 16.4 Hz, 1H), 2.44 (s, 3H), 1.64 – 1.44 (m, 6H), 1.24 – 1.15 (m, 2H), 1.06 – 0.90 (m, 2H); ^{13}C NMR (100 MHz, $CDCl_3$) δ 149.3, 144.9, 138.1, 136.2, 135.4, 134.2, 132.6, 130.0, 129.5, 129.3, 128.6, 126.8, 126.3, 126.2, 124.6, 124.1, 120.0, 112.3, 56.6, 53.8, 40.6, 39.2, 36.4, 35.8, 26.0, 23.5, 23.3, 21.6; IR (neat): 2925, 2854, 1615, 1519, 1488, 1369, 1174, 1124, 1005; HRESIMS Calcd for $[C_{33}H_{35}ClN_2KO_2S]^+$ (M + K^+) 597.1739, found 597.1742.

(*R*)-*N*-((3-bromophenyl)ethynyl)-*N*-(1-cyclohexyl-4-(4-(dimethylamino)phenyl)but-3-yn-2-yl)-4-methylbenzenesulfonamide ((+)-1y)

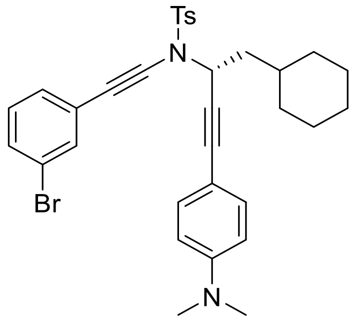

(+)-**1y**

Compound (+)-**1y** was prepared in 39% yield (47.1 mg) according to the general procedure. Pale yellow oil, (eluent: PE/DCM = 2/1). $[\alpha]_D^{25}$ = +19.6º (c = 1.0, $CHCl_3$). 96% ee (determined by HPLC: Chiralpak ADH Column, 10/90 *i*-PrOH/hexane, 1.0 mL/min, 254 nm; TR = 6.41 min (minor), 9.27 min (major)).

(*R*)-4-(3'-(3-bromophenyl)-1'-tosyl-4',6'-dihydro-1'*H*-spiro[cyclohexane-1,5'-cyclopenta[*b*]pyrrol]-4'-yl)-*N*,*N*-dimethylaniline ((-)-2y)

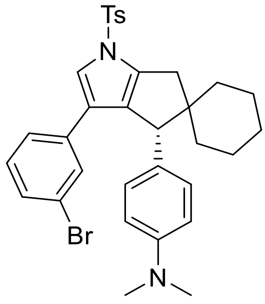

(-)-**2y**

Compound (-)-**2y** was prepared in 42% yield (50.7 mg) according to the general procedure. Pale yellow oil, (eluent: PE/DCM = 2/1). $[\alpha]_D^{25}$ = -24.7º (c = 1.0, $CHCl_3$). 88% ee (determined by HPLC: Chiralpak ADH Column, 10/90 *i*-PrOH/hexane, 1.0 mL/min, 254 nm; TR = 5.92 min (major), 9.60 min (minor)). ^{1}H NMR (600 MHz, $CDCl_3$) δ 7.77 (d, *J* = 8.4 Hz, 2H), 7.42 – 7.29 (m, 4H), 7.17 (dd, *J* = 7.8, 0.6 Hz, 1H), 7.12 (d, *J* = 7.8 Hz, 1H), 7.00 – 6.95 (m, 1H), 6.95 – 6.15 (m, 4H), 3.67 (s, 1H), 2.90 (d, *J* = 16.2 Hz, 1H), 2.87 (s, 6H), 2.67 (d, *J* = 16.2 Hz, 1H), 2.44 (s, 3H), 1.66 – 1.42 (m, 7H), 1.10 – 1.02 (m, 1H), 1.02 – 0.92 (m, 1H), 0.91 – 0.81 (m, 1H). ^{13}C NMR (150 MHz, $CDCl_3$) δ 149.2, 144.9, 138.0, 136.1, 135.6, 132.5, 130.0, 129.8, 129.2, 129.0, 128.5, 126.7, 124.5, 124.4, 122.5, 120.0, 112.3, 56.6, 53.7, 40.6, 39.2, 36.4, 35.8, 26.0, 23.4, 23.3, 21.6; IR (neat): 2924, 2852, 1614, 1519, 1369, 1187, 1125, 1100, 673; HRESIMS Calcd for $[C_{33}H_{35}BrN_2NaO_2S]^+$ (M + Na^+) 625.1495, found 625.1499.

(*R*)-*N*-((4-chlorophenyl)ethynyl)-*N*-(1-cyclohexyl-4-(4-(dimethylamino)phenyl)but-3-yn-2-yl)-4-methylbenzenesulfonamide ((+)-1z)

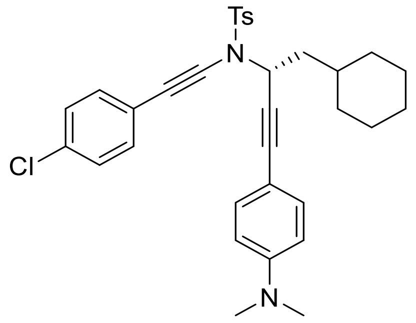

(+)-**1z**

Compound (+)-**1z** was prepared in 42% yield (47.0 mg) according to the general procedure. Pale yellow oil, (eluent: PE/DCM = 2/1). $[\alpha]_D^{25}$ = +16.4º (c = 1.0, $CHCl_3$).

82% ee (determined by HPLC: Chiralpak ADH Column, 10/90 *i*-PrOH/hexane, 1.0 mL/min, 254 nm; TR = 7.80 min (minor), 10.27 min (major)).

(*R*)-4-(3'-(4-chlorophenyl)-1'-tosyl-4',6'-dihydro-1'*H*-spiro[cyclohexane-1,5'-cyclopenta[*b*]pyrrol]-4'-yl)-*N,N*-dimethylaniline ((-)-2z)

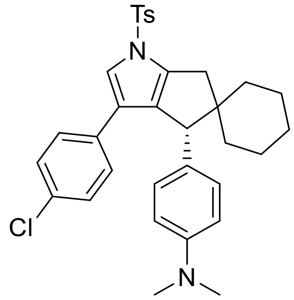

(-)-**2z**

Compound (-)-**2z** was prepared in 48% yield (53.4 mg) according to the general procedure. Pale yellow oil, (eluent: PE/DCM = 2/1). $[\alpha]_D^{25}$ = -11.3º (c = 1.0, $CHCl_3$). 75% ee (determined by HPLC: Chiralpak IG Column, 50/50 *i*-PrOH/hexane, 1.0 mL/min, 254 nm; TR = 6.69 min (major), 15.05 min (minor)). ^{1}H NMR (500 MHz, $CDCl_3$) δ 7.77 (d, *J* = 8.5 Hz, 2H), 7.36 (s, 1H), 7.33 (d, *J* = 8.0 Hz, 2H), 7.15 – 7.07 (m, 4H), 6.99 – 6.83 (m, 1H), 6.82 – 6.32 (m, 4H), 3.65 (d, *J* = 1.0 Hz, 1H), 2.90 (d, *J* = 16.0 Hz, 1H), 2.87 (s, 6H), 2.65 (d, *J* = 16.0 Hz, 1H), 2.43 (s, 3H), 1.69 – 1.44 (m, 6H), 1.23 – 1.16 (m, 2H), 1.07 – 0.92 (m, 2H); ^{13}C NMR (125 MHz, $CDCl_3$) δ 149.2, 144.8, 138.1, 136.2, 132.4, 132.0(3), 132.0(1), 130.0, 128.5(3), 128.4(7), 127.2, 126.7, 124.8, 119.6, 112.2, 56.7, 53.8, 40.5, 39.2, 36.3, 35.8, 26.0, 23.4, 23.3, 21.6; IR (neat): 2926, 2851, 1610, 1558, 1397, 1371, 1175, 1103, 1003, 813; HRESIMS Calcd for $[C_{33}H_{35}ClKN_2O_2S]^+$ (M + K^+) 597.1739, found 597.1731.

(*R*)-*N*-((4-bromophenyl)ethynyl)-*N*-(1-cyclohexyl-4-(4-(dimethylamino)phenyl)but-3-yn-2-yl)-4-methylbenzenesulfonamide ((+)-1aa)

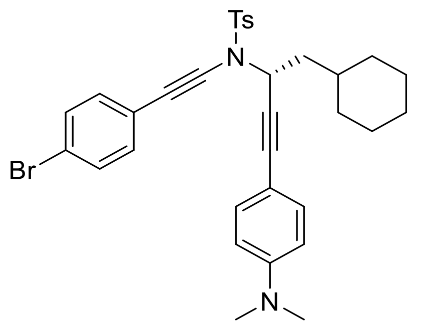

(+)-**1aa**

Compound (+)-**1aa** was prepared in 39% yield (47.1 mg) according to the general procedure. Pale yellow oil, (eluent: PE/DCM = 2/1). $[\alpha]_D^{25}$ = +5.9º (c = 1.0, $CHCl_3$). 78% ee (determined by HPLC: Chiralpak ADH Column, 10/90 *i*-PrOH/hexane, 1.0 mL/min, 254 nm; TR = 8.12 min (minor), 11.29 min (major)).

(*R*)-4-(3'-(4-bromophenyl)-1'-tosyl-4',6'-dihydro-1'*H*-spiro[cyclohexane-1,5'-cyclopenta[*b*]pyrrol]-4'-yl)-*N,N*-dimethylaniline ((-)-2aa)

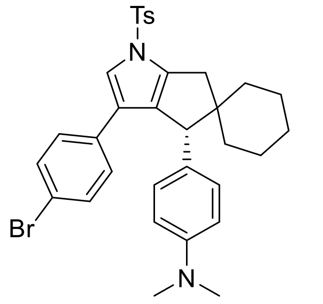

(-)-**2aa**

Compound (-)-**2aa** was prepared in 41% yield (49.5 mg) according to the general procedure. Pale yellow oil, (eluent: PE/DCM = 2/1). $[\alpha]_D^{25}$ = -41.2º (c = 1.0, $CHCl_3$). 80% ee (determined by HPLC: Chiralpak ADH Column, 40/60 *i*-PrOH/hexane, 1.0 mL/min, 254 nm; TR = 6.01 min (major), 19.19 min (minor)). ^{1}H NMR (500 MHz, $CDCl_3$) δ 7.77 (d, *J* = 8.5 Hz, 2H), 7.37 (s, 1H), 7.33 (d, *J* = 8.0 Hz, 2H), 7.24 (d, *J* = 9.0 Hz, 2H), 7.06 (d, *J* = 8.5 Hz, 2H), 6.98 – 6.16 (m, 4H), 3.65 (s, 1H), 2.90 (d, *J* = 16.0 Hz, 1H), 2.87 (s, 6H), 2.65 (d, *J* = 16.0 Hz, 1H), 2.43 (s, 3H), 1.65 – 1.41 (m, 6H), 1.23 – 1.15 (m, 2H), 1.03 – 0.86 (m, 2H); ^{13}C NMR (150 MHz, $CDCl_3$) δ 149.1, 144.9, 138.1, 136.1, 132.5, 132.3, 131.4, 130.0, 128.5, 127.6, 126.7, 124.8, 120.1, 119.6, 112.2, 56.7, 53.8, 40.5, 39.2, 36.2, 35.8, 26.0, 23.4, 23.3, 21.6; IR (neat): 2924, 2854, 1620,

1519, 1369, 1174, 1124, 1102, 1005, 816; HRESIMS Calcd for $[C_{33}H_{35}BrN_2NaO_2S]^+$ (M + Na^+) 625.1495, found 625.1489.

(*R*)-*N*-(1-cyclohexyl-4-(4-(dimethylamino)phenyl)but-3-yn-2-yl)-4-methyl-*N*-((4-(trifluoromethyl)phenyl)ethynyl)benzenesulfonamide ((+)-1ab)

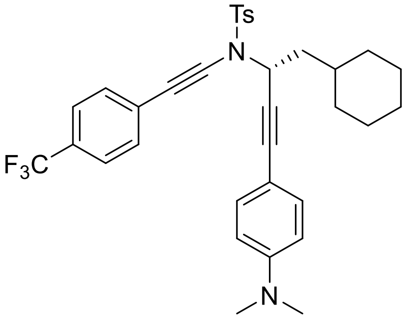

(+)-**1ab**

Compound (+)-**1ab** was prepared in 41% yield (48.6 mg) according to the general procedure. Pale yellow oil, (eluent: PE/DCM = 2/1). $[\alpha]_D^{25}$ = +21.7º (c = 1.0, $CHCl_3$).74% ee (determined by HPLC: Chiralpak ADH Column, 10/90 *i*-PrOH/hexane, 1.0 mL/min, 254 nm; TR = 6.39 min (minor), 9.16 min (major)).

(*R*)-*N*,*N*-dimethyl-4-(1'-tosyl-3'-(4-(trifluoromethyl)phenyl)-4',6'-dihydro-1'*H*-spiro[cyclohexane-1,5'-cyclopenta[*b*]pyrrol]-4'-yl)aniline ((-)-2ab)

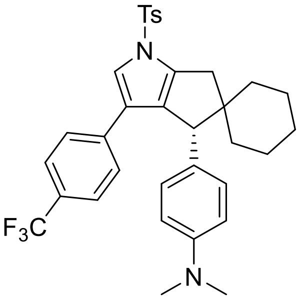

(-)-**2ab**

Compound (-)-**2ab** was prepared in 42% yield (49.7 mg) according to the general procedure. Pale yellow oil, (eluent: PE/DCM = 2/1). $[\alpha]_D^{25}$ = -16.4º (c = 1.0, $CHCl_3$). 74% ee (determined by HPLC: Chiralpak ADH Column, 40/60 *i*-PrOH/hexane, 1.0 mL/min, 254 nm; TR = 4.52 min (major), 19.14 min (minor)). ^1H NMR (500 MHz, $CDCl_3$) δ 7.79 (d, *J* = 8.5 Hz, 2H), 7.45 (s, 1H), 7.41 – 7.32 (m, 4H), 7.30 (d, *J* = 8.0

Hz, 2H), 7.11 – 6.17 (m, 4H), 3.68 (s, 1H), 2.91 (d, *J* = 16.0 Hz, 1H), 2.88 (s, 6H), 2.66 (d, *J* = 16.0 Hz, 1H), 2.44 (s, 3H), 1.65 – 1.43 (m, 6H), 1.24 – 1.16 (m, 2H), 1.05 – 0.91 (m, 2H); ^{13}C NMR (150 MHz, $CDCl_3$) δ 149.2, 145.0, 138.3, 137.1, 136.1, 132.4, 130.0, 128.1 (q, *J* = 32.2 Hz), 126.8, 126.0, 125.3 (q, *J* = 3.8 Hz), 125.1, 124.5, 123.3, 120.4, 112.2, 56.7, 53.9, 40.5, 39.2, 36.2, 35.8, 26.0, 23.4, 23.3, 21.7; ^{19}F NMR (376 MHz, $CDCl_3$) δ -62.5 (s); IR (neat): 2923, 2850, 1614, 1518, 1447, 1325, 1174, 1125, 1068, 814; HRESIMS Calcd for $[C_{34}H_{35}F_3N_2NaO_2S]^+$ (M + Na^+) 615.2264, found 615.2254.

(*R*)-*N*-(1-cyclohexyl-4-(4-(dimethylamino)phenyl)but-3-yn-2-yl)-*N*-(hex-1-yn-1-yl)-4-methylbenzenesulfonamide ((+)-1ac)

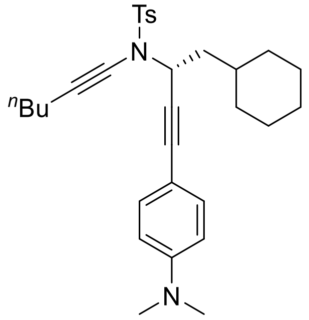

(+)-**1ac**

Compound (+)-**1ac** was prepared in 46% yield (46.5 mg) according to the general procedure at 15 °C. Pale yellow oil, (eluent: PE/DCM = 2/1). $[\alpha]_D^{25}$ = +28.8º (c = 1.0, $CHCl_3$). 97% ee (determined by HPLC: Chiralpak IE Column, 10/90 *i*-PrOH/hexane, 1.0 mL/min, 254 nm; TR = 5.25 min (minor), 8.12 min (major)).

(*R*)-4-(3'-butyl-1'-tosyl-4',6'-dihydro-1'*H*-spiro[cyclohexane-1,5'-cyclopenta[*b*]pyrrol]-4'-yl)-*N,N*-dimethylaniline ((-)-2ac)

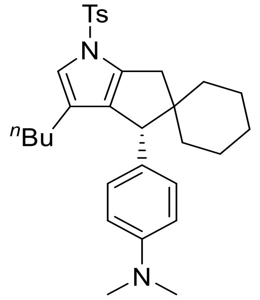

(-)-**2ac**

Compound (-)-**2ac** was prepared in 49% yield (49.5 mg) according to the general procedure at 15 °C. Pale yellow oil, (eluent: PE/DCM = 2/1). $[\alpha]_D^{25}$ = -90.6º (c = 1.0, $CHCl_3$). 90% ee (determined by HPLC: Chiralpak IE Column, 2/98 *i*-PrOH/hexane, 1.0 mL/min, 254 nm; TR = 7.18 min (minor), 9.15 min (major)). ^{1}H NMR (500 MHz, $CDCl_3$) δ 7.70 (d, *J* = 8.5 Hz, 2H), 7.30 (d, *J* = 8.0 Hz, 2H), 6.79 (s, 1H), 6.78 – 6.24 (m, 4H), 3.47 (s, 1H), 2.89 (s, 6H), 2.79 (d, *J* = 16.0 Hz, 1H), 2.69 (d, *J* = 16.5 Hz, 1H), 2.43 (s, 3H), 2.06 – 1.92 (m, 2H), 1.65 – 1.38 (m, 7H), 1.22 – 1.05 (m, 6H), 0.85 – 0.76 (m, 1H), 0.70 (t, *J* = 7.5 Hz, 3H); ^{13}C NMR (125 MHz, $CDCl_3$) δ 149.1, 144.2, 136.6(3), 136.6(2), 134.2, 129.7, 129.3, 128.9, 126.8, 126.5, 120.4, 112.1, 55.8, 53.5, 40.7, 39.2, 37.0, 35.1, 30.9, 25.9, 25.3, 23.5, 23.3, 22.0, 21.6, 13.7; IR (neat): 2927, 2853, 1615, 1519, 1447, 1368, 1187, 1173, 1126, 814; HRESIMS Calcd for $[C_{31}H_{40}N_2NaO_2S]^+$ (M + Na^+) 527.2703, found 527.2712.

(*R*)-*N*-(1-cyclopentyl-4-(4-(dimethylamino)phenyl)but-3-yn-2-yl)-*N*-((2,6-dimethylphenyl)ethynyl)-4-methylbenzenesulfonamide ((+)-1af)

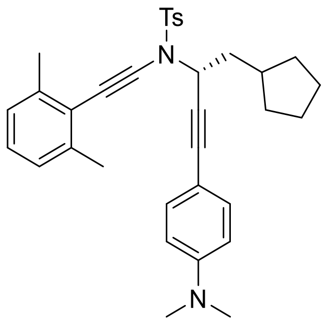

(+)-**1af**

Compound (+)-**1af** was prepared in 41% yield (44.1 mg) according to the general procedure. Pale yellow oil, (eluent: PE/DCM = 2/1). $[\alpha]_D^{25}$ = +129.9º (c = 1.0, $CHCl_3$). 95% ee (determined by HPLC: Chiralpak IE Column, 3/97 *i*-PrOH/hexane, 1.0 mL/min, 254 nm; TR = 22.62 min (minor), 24.46 min (major)).

(*R*)-4-(3'-(2,6-dimethylphenyl)-1'-tosyl-4',6'-dihydro-1'*H*-spiro[cyclopentane-1,5'-cyclopenta[*b*]pyrrol]-4'-yl)-*N*,*N*-dimethylaniline ((-)-2af)

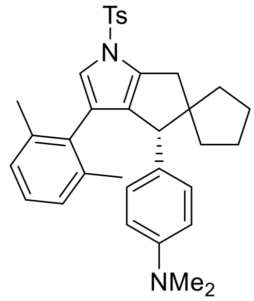

(-)-**2af**

Compound (-)-**2af** was prepared in 42% yield (45.2 mg) according to the general procedure. Pale yellow oil, (eluent: PE/DCM = 2/1). $[\alpha]_D^{25}$ = -144.0º (c = 1.0, $CHCl_3$). 93% ee (determined by HPLC: Chiralpak IE Column, 3/97 *i*-PrOH/hexane, 1.0 mL/min, 254 nm; TR = 13.88 min (minor), 15.98 min (major)). ^{1}H NMR (400 MHz, $CDCl_3$) δ 7.74 (d, *J* = 8.4 Hz, 2H), 7.33 (d, *J* = 8.0 Hz, 2H), 6.99 – 6.91 (m, 2H), 6.87 (s, 1H), 6.79 (d, *J* = 6.8 Hz, 1H), 6.45 (d, *J* = 8.8 Hz, 2H), 6.40 (d, *J* = 8.8 Hz, 2H), 3.27 (s, 1H), 2.91 (d, *J* = 16.0 Hz, 1H), 2.83 (s, 6H), 2.77 (d, *J* = 15.6 Hz, 1H), 2.46 (s, 3H), 1.96 (s, 3H), 1.72 – 1.62 (m, 4H), 1.61 (s, 3H), 1.55 – 1.46 (m, 2H), 1.20 – 1.11 (m, 2H); ^{13}C NMR (100 MHz, $CDCl_3$) δ 149.0, 144.5, 137.2, 137.1, 137.0(4), 136.9(8), 136.4, 133.0, 130.1, 129.7, 128.5, 126.9, 126.5(3), 126.4(7), 125.0, 121.6, 112.1, 62.4, 53.8, 42.0, 40.8, 39.3, 35.0, 23.6, 23.2, 21.6, 20.5, 20.3; IR (neat): 2950, 2857, 1614, 1519, 1445, 1406, 1369, 1186, 1174, 1098; HRESIMS Calcd for $[C_{34}H_{38}N_2NaO_2S]^+$ (M + Na^+) 561.2546, found 561.2555.

(*R*)-*N*-(1-cycloheptyl-4-(4-(dimethylamino)phenyl)but-3-yn-2-yl)-*N*-((2,6-dimethylphenyl)ethynyl)-4-methylbenzenesulfonamide ((+)-1ag)

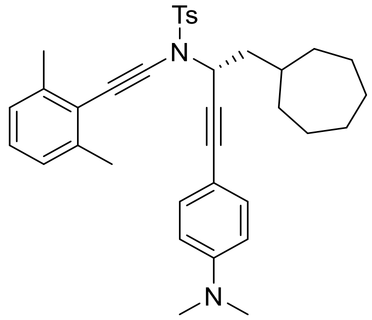

(+)-**1ag**

Compound (+)-**1ag** was prepared in 42% yield (47.5 mg) according to the general procedure. Pale yellow oil, (eluent: PE/DCM = 2/1). $[\alpha]_D^{25}$ = +59.4º (c = 1.0, $CHCl_3$). 96% ee (determined by HPLC: Chiralpak IE Column, 10/90 *i*-PrOH/hexane, 1.0 mL/min, 254 nm; TR = 11.44 min (minor), 12.43 min (major)).

(*R*)-4-(3'-(2,6-dimethylphenyl)-1'-tosyl-4',6'-dihydro-1'*H*-spiro[cycloheptane-1,5'-cyclopenta[*b*]pyrrol]-4'-yl)-*N,N*-dimethylaniline ((-)-2ag)

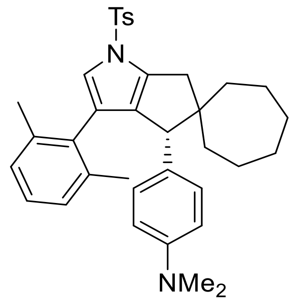

(-)-**2ag**

Compound (-)-**2ag** was prepared in 44% yield (49.8 mg) according to the general procedure. Pale yellow oil, (eluent: PE/DCM = 2/1). $[\alpha]_D^{25}$ = -53.5º (c = 1.0, $CHCl_3$). 91% ee (determined by HPLC: Chiralpak IE Column, 2/98 *i*-PrOH/hexane, 1.0 mL/min, 254 nm; TR = 7.96 min (minor), 9.34 min (major)). ^{1}H NMR (500 MHz, $CDCl_3$) δ 7.75 (d, *J* = 8.5 Hz, 2H), 7.32 (d, *J* = 8.0 Hz, 2H), 6.99 – 6.89 (m, 2H), 6.87 (s, 1H), 6.78 (d, *J* = 7.0 Hz, 1H), 6.51 – 6.34 (m, 4H), 3.25 (s, 1H), 2.83 (s, 6H), 2.81 – 2.77 (m, 1H), 2.72 (d, *J* = 16.0 Hz, 1H), 2.46 (s, 3H), 1.96 (s, 3H), 1.77 – 1.70 (m, 1H), 1.64 (s, 3H), 1.63 – 1.53 (m, 2H), 1.51 – 1.40 (m, 5H), 1.27 – 1.13 (m, 4H); ^{13}C NMR (125 MHz, $CDCl_3$) δ 148.9, 144.5, 137.1(2), 137.0(5), 137.0, 136.9, 136.4, 133.0, 129.7, 129.3, 129.0, 126.9, 126.8, 126.6, 126.5, 125.3, 121.5, 112.0, 57.6, 56.2, 42.9, 40.7, 39.9, 36.9, 30.2, 29.9, 23.5, 23.4, 21.6, 20.6, 20.4; IR (neat): 2921, 2852, 1614, 1519, 1462, 1370, 1265, 1186, 1175, 1101; HRESIMS Calcd for $[C_{36}H_{42}N_2NaO_2S]^+$ (M + Na^+) 589.2859, found 589.2848.

(*R*)-*N*-(1-cyclooctyl-4-(4-(dimethylamino)phenyl)but-3-yn-2-yl)-*N*-((2,6-dimethylphenyl)ethynyl)-4-methylbenzenesulfonamide ((+)-1ah)

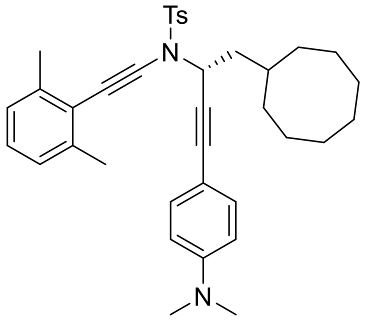

(+)-**1ah**

Compound (+)-**1ah** was prepared in 41% yield (47.6 mg) according to the general procedure. Pale yellow oil, (eluent: PE/DCM = 2/1). $[\alpha]_D^{25}$ = +24.8º (c = 1.0, $CHCl_3$). 94% ee (determined by HPLC: Chiralpak IE Column, 10/90 *i*-PrOH/hexane, 1.0 mL/min, 254 nm; TR = 11.28 min (minor), 12.41 min (major)).

(*R*)-4-(3'-(2,6-dimethylphenyl)-1'-tosyl-4',6'-dihydro-1'*H*-spiro[cyclooctane-1,5'-cyclopenta[*b*]pyrrol]-4'-yl)-*N,N*-dimethylaniline ((-)-2ah)

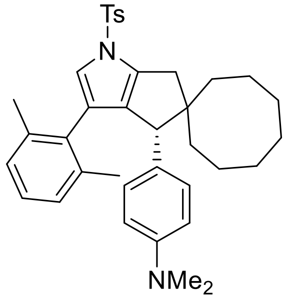

(-)-**2ah**

Compound (-)-**2ah** was prepared in 42% yield (48.8 mg) according to the general procedure. Pale yellow oil, (eluent: PE/DCM = 2/1). $[\alpha]_D^{25}$ = -87.2º (c = 1.0, $CHCl_3$). 90% ee (determined by HPLC: Chiralpak IE Column, 10/90 *i*-PrOH/hexane, 1.0 mL/min, 254 nm; TR = 7.84 min (minor), 9.21 min (major)). ^1H NMR (400 MHz, $CDCl_3$) δ 7.74 (d, *J* = 8.4 Hz, 2H), 7.32 (d, *J* = 8.0 Hz, 2H), 6.97 – 6.90 (m, 2H), 6.86 (s, 1H), 6.76 (d, *J* = 6.4 Hz, 1H), 6.46 – 6.36 (m, 4H), 3.28 (s, 1H), 2.83 (s, 6H), 2.79 (d, *J* = 16.0 Hz, 1H), 2.71 (d, *J* = 16.0 Hz, 1H), 2.46 (s, 3H), 1.96 (s, 3H), 1.79 – 1.62 (m, 3H), 1.61 (s, 3H), 1.60 – 1.39 (m, 9H), 1.36 – 1.26 (m, 2H); ^{13}C NMR (100 MHz, $CDCl_3$) δ 148.9, 144.5, 137.2, 137.1, 136.9, 136.7, 136.5, 133.0, 129.7, 129.4, 129.2, 126.9, 126.8, 126.5, 126.4, 125.4, 121.5, 112.0, 57.5, 54.3, 40.7, 39.3, 36.5, 32.3, 28.6,

28.5, 25.1, 23.6, 23.4, 21.6, 20.5, 20.3; IR (neat): 2919, 2851, 1614, 1519, 1464, 1445, 1370, 1266, 1186, 1174; HRESIMS Calcd for $[C_{37}H_{44}N_2NaO_2S]^+$ $(M + Na^+)$ 603.3016, found 603.3021.

(*R*)-*N*-(1-cyclododecyl-4-(4-(dimethylamino)phenyl)but-3-yn-2-yl)-*N*-((2,6-dimethylphenyl)ethynyl)-4-methylbenzenesulfonamide ((+)-1ai)

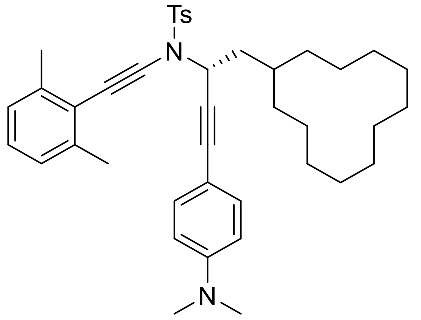

(+)-**1ai**

Compound (+)-**1ai** was prepared in 48% yield (61.0 mg) according to the general procedure. Pale yellow oil, (eluent: PE/DCM = 2/1). $[\alpha]_D^{25}$ = +30.4º (c = 1.0, $CHCl_3$). 92% ee (determined by HPLC: Chiralpak IE Column, 10/90 *i*-PrOH/hexane, 1.0 mL/min, 254 nm; TR = 9.39 min (minor), 10.11 min (major)).

(*R*)-4-(3'-(2,6-dimethylphenyl)-1'-tosyl-4',6'-dihydro-1'*H*-spiro[cyclododecane-1,5'-cyclopenta[*b*]pyrrol]-4'-yl)-*N,N*-dimethylaniline ((-)-2ai)

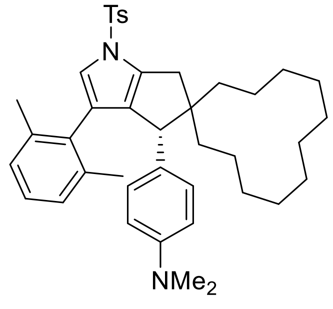

(-)-**2ai**

Compound (-)-**2ai** was prepared in 46% yield (58.5 mg) according to the general procedure. Pale yellow oil, (eluent: PE/DCM = 2/1). $[\alpha]_D^{25}$ = -66.5º (c = 1.0, $CHCl_3$). 96% ee (determined by HPLC: Chiralpak IE Column, 10/90 *i*-PrOH/hexane, 1.0 mL/min, 254 nm; TR = 7.94 min (minor), 9.18 min (major)). ^1H NMR (400 MHz,

$CDCl_3$) δ 7.74 (d, *J* = 8.0 Hz, 2H), 7.32 (d, *J* = 8.0 Hz, 2H), 7.01 – 6.90 (m, 2H), 6.86 (s, 1H), 6.77 (d, *J* = 6.8 Hz, 1H), 6.40 (s, 4H), 3.35 (s, 1H), 2.83 (s, 6H), 2.78 (d, *J* = 16.0 Hz, 1H), 2.60 (d, *J* = 16.0 Hz, 1H), 2.46 (s, 3H), 1.99 (s, 3H), 1.57 (s, 3H), 1.46 – 0.97 (m, 22H); ^{13}C NMR (100 MHz, $CDCl_3$) δ 148.9, 144.5, 137.3, 137.2, 137.1, 136.9, 136.4, 133.0, 129.7, 129.5, 129.1, 126.8(4), 126.8(1), 126.6, 126.5, 125.3, 121.5, 112.2, 56.8, 51.9, 40.8, 38.7, 34.7, 31.4, 26.9(1), 26.8(6), 26.1, 22.8(0), 22.7(8), 22.5, 22.4, 21.6, 20.5, 20.2, 20.1, 19.9; IR (neat): 2934, 2859, 1614, 1519, 1470, 1444, 1370, 1265, 1122, 1101; HRESIMS Calcd for $[C_{41}H_{52}N_2NaO_2S]]^+$ (M + Na^+) 659.3642, found 659.3644.

(*R*)-*N*-(1-cyclopentadecyl-4-(4-(dimethylamino)phenyl)but-3-yn-2-yl)-*N*-((2,6-dimethylphenyl)ethynyl)-4-methylbenzenesulfonamide ((+)-1aj)

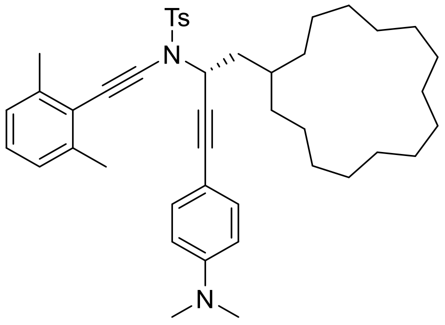

(+)-**1aj**

Compound (+)-**1aj** was prepared in 44% yield (59.8 mg) according to the general procedure. Pale yellow oil, (eluent: PE/DCM = 2/1). $[\alpha]_D^{25}$ = +52.4° (c = 1.0, $CHCl_3$). 94% ee (determined by HPLC: Chiralpak IE Column, 2/98 *i*-PrOH/hexane, 1.0 mL/min, 254 nm; TR = 18.59 min (minor), 20.16 min (major)).

(*R*)-4-(3'-(2,6-dimethylphenyl)-1'-tosyl-4',6'-dihydro-1'*H*-spiro[cyclopentadecane-1,5'-cyclopenta[*b*]pyrrol]-4'-yl)-*N*,*N*-dimethylaniline ((-)-2aj)

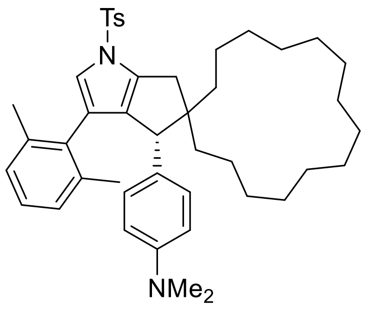

(-)-**2aj**

Compound (-)-**2aj** was prepared in 44% yield (59.8 mg) according to the general procedure. Pale yellow oil, (eluent: PE/DCM = 2/1). $[\alpha]_D^{25}$ = -79.2º (c = 1.0, $CHCl_3$). 93% ee (determined by HPLC: Chiralpak IE Column, 5/95 *i*-PrOH/hexane, 1.0 mL/min, 254 nm; TR = 8.47 min (minor), 9.82 min (major)). ^{1}H NMR (400 MHz, $CDCl_3$) δ 7.74 (d, *J* = 8.4 Hz, 2H), 7.32 (d, *J* = 8.0 Hz, 2H), 7.02 – 6.90 (m, 2H), 6.86 (s, 1H), 6.77 (d, *J* = 6.4 Hz, 1H), 6.53 – 6.30 (m, 4H), 3.29 (s, 1H), 2.83 (s, 6H), 2.78 – 2.64 (m, 2H), 2.46 (s, 3H), 1.97 (s, 3H), 1.58 (s, 3H), 1.38 – 1.02 (m, 28H); ^{13}C NMR (100 MHz, $CDCl_3$) δ 149.0, 144.5, 137.3, 137.2, 137.1, 136.9, 136.5, 133.0, 129.7, 129.3, 129.1, 126.9, 126.8, 126.6, 126.5, 125.4, 121.5, 112.2, 56.5, 53.0, 40.8, 39.2, 38.8, 34.8, 28.0, 27.9, 26.9(3), 26.8(5), 26.7(7), 26.6, 25.9, 22.6, 22.5, 21.6, 20.5, 20.3; IR (neat): 2931, 2860, 1614, 1515, 1471, 1445, 1370, 1266, 1122, 1103, 815; HRESIMS Calcd for $[C_{44}H_{58}N_2NaO_2S]^+$ (M + Na^+) 701.4111, found 701.4119.

(*R*)-*N*-(4-(4-(dimethylamino)phenyl)-1-(1,4-dioxaspiro[4.5]decan-8-yl)but-3-yn-2-yl)-*N*-((2,6-dimethylphenyl)ethynyl)-4-methylbenzenesulfonamide ((+)-1ak)

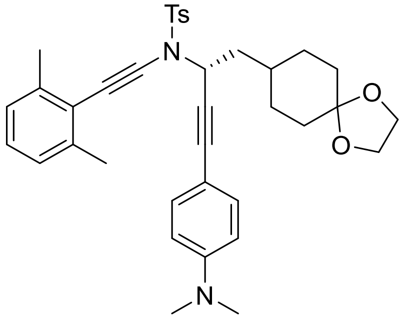

(+)-**1ak**

Compound (+)-**1ak** was prepared in 44% yield (53.7 mg) according to the general procedure. Pale yellow oil, (eluent: PE/DCM = 2/1). $[\alpha]_D^{25}$ = +66.4º (c = 1.0, $CHCl_3$).

94% ee (determined by HPLC: Chiralpak IE Column, 30/70 *i*-PrOH/hexane, 1.0 mL/min, 254 nm; TR = 15.71 min (minor), 18.91 min (major)).

(*R*)-4-(3-(2,6-dimethylphenyl)-1-tosyl-4,6-dihydro-1*H*-dispiro[cyclopenta[*b*]pyrrole-5,1'-cyclohexane-4',2''-[1,3]dioxolan]-4-yl)-*N*,*N*-dimethylaniline ((-)-2ak)

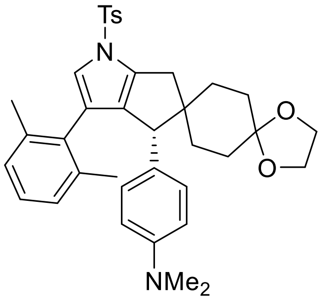

(-)-**2ak**

Compound (-)-**2ak** was prepared in 44% yield (53.7 mg) according to the general procedure. Pale yellow solid (mp 121–122 ºC) , (eluent: PE/DCM = 2/1). $[\alpha]_D^{25}$ = -53.8º (c = 1.0, $CHCl_3$). 94% ee (determined by HPLC: Chiralpak IE Column, 30/70 *i*-PrOH/hexane, 1.0 mL/min, 254 nm; TR = 8.41 min (minor), 9.11 min (major)). ^{1}H NMR (500 MHz, $CDCl_3$) δ 7.75 (d, *J* = 8.5 Hz, 2H), 7.32 (d, *J* = 8.0 Hz, 2H), 6.99 – 6.90 (m, 2H), 6.89 (s, 1H), 6.78 (d, *J* = 7.0 Hz, 1H), 6.47 (d, *J* = 8.0 Hz, 2H), 6.39 (d, *J* = 9.0 Hz, 2H), 3.96 – 3.84 (m, 4H), 3.26 (s, 1H), 2.92 (d, *J* = 16.0 Hz, 1H), 2.83 (s, 6H), 2.76 (d, *J* = 16.0 Hz, 1H), 2.46 (s, 3H), 1.95 (s, 3H), 1.78 – 1.65 (m, 4H), 1.63 (s, 3H), 1.62 – 1.56 (m, 1H), 1.53 – 1.40 (m, 2H), 1.27 – 1.19 (m, 2H), 1.16 – 1.09 (m, 1H); ^{13}C NMR (125 MHz, $CDCl_3$) δ 149.1, 144.6, 137.1, 136.9, 136.7, 136.4, 132.8, 129.7, 128.9, 128.6, 126.9(0), 126.8(8), 126.6, 126.5, 125.1, 121.8, 112.0, 108.7, 64.2, 64.1, 54.4, 52.9, 40.7, 36.3, 36.0, 32.2, 32.0, 31.9, 21.6, 20.5, 20.3; IR (neat): 2923, 1612, 1519, 1444, 1369, 1269, 1174, 1098, 814; HRESIMS Calcd for $[C_{37}H_{42}N_2NaO_4S]^+$ (M + Na^+) 633.2757, found 633.2766.

(*R*)-*N*-(4-(4-(dimethylamino)phenyl)-1-(4,4-dimethylcyclohexyl)but-3-yn-2-yl)-*N*-((2,6-dimethylphenyl)ethynyl)-4-methylbenzenesulfonamide ((+)-1al)

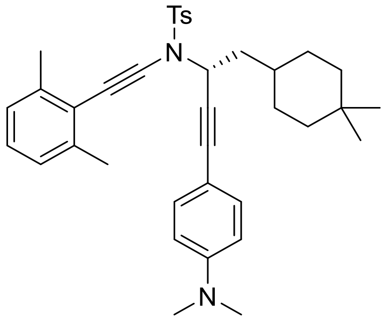

(+)-**1al**

Compound (+)-**1al** was prepared in 45% yield (52.5 mg) according to the general procedure. Pale yellow oil, (eluent: PE/DCM = 2/1). $[\alpha]_D^{25}$ = +40.9º (c = 1.0, $CHCl_3$). 96% ee (determined by HPLC: Chiralpak IE Column, 5/95 *i*-PrOH/hexane, 1.0 mL/min, 254 nm; TR = 14.95 min (minor), 16.39 min (major)).

(*R*)-4-(3'-(2,6-dimethylphenyl)-4,4-dimethyl-1'-tosyl-4',6'-dihydro-1'*H*-spiro[cyclohexane-1,5'-cyclopenta[*b*]pyrrol]-4'-yl)-*N,N*-dimethylaniline ((-)-2al)

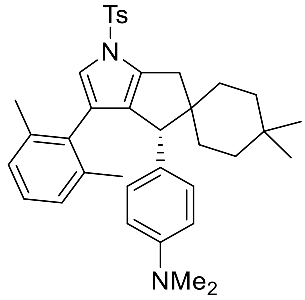

(-)-**2al**

Compound (-)-**2al** was prepared in 45% yield (52.2 mg) according to the general procedure. Pale yellow oil, (eluent: PE/DCM = 2/1). $[\alpha]_D^{25}$ = -84.2º (c = 1.0, $CHCl_3$). 96% ee (determined by HPLC: Chiralpak IE Column, 5/95 *i*-PrOH/hexane, 1.0 mL/min, 254 nm; TR = 8.63 min (minor), 9.63 min (major)). ^{1}H NMR (500 MHz, $CDCl_3$) δ 7.75 (d, *J* = 8.5 Hz, 2H), 7.32 (d, *J* = 8.0 Hz, 2H), 6.98 – 6.88 (m, 2H), 6.86 (s, 1H), 6.78 (d, *J* = 7.5 Hz, 1H), 6.45 (d, *J* = 8.0 Hz, 2H), 6.39 (d, *J* = 8.5 Hz, 2H), 3.23 (s, 1H), 2.92 (d, *J* = 16.0 Hz, 1H), 2.82 (s, 6H), 2.68 (d, *J* = 16.0 Hz, 1H), 2.46 (s, 3H), 1.95 (s, 3H), 1.63 (s, 3H), 1.57 – 1.52 (m, 2H), 1.43 – 1.37 (m, 1H), 1.31 – 1.23 (m, 2H), 1.14 – 0.99 (m, 3H), 0.89 (s, 3H), 0.81 (s, 3H); ^{13}C NMR (125 MHz, $CDCl_3$) δ 149.0, 144.5, 137.1, 137.0, 136.9, 136.4, 132.9, 129.7, 129.0, 126.8(5), 126.8(2), 126.5(3), 126.4(7), 125.3,

121.6, 112.0, 53.7, 40.7, 36.2, 36.1, 34.9, 31.0, 29.6, 21.6, 20.5, 20.3; IR (neat): 2920, 2851, 1614, 1519, 1463, 1370, 1187, 1175, 1128, 1101; HRESIMS Calcd for $[C_{37}H_{45}N_2O_2S]^+$ (M + H^+) 581.3196, found 581.3201.

(*R*)-*N*-(1-((1*R*,3*S*,5*r*,7*r*)-adamantan-2-yl)-4-(4-(dimethylamino)phenyl)but-3-yn-2-yl)-*N*-((2,6-dimethylphenyl)ethynyl)-4-methylbenzenesulfonamide ((+)-1am)

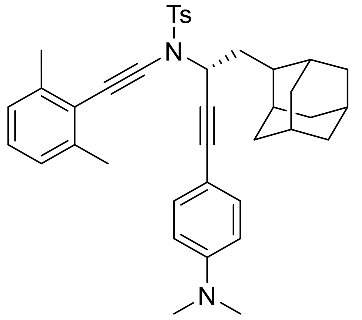

(+)-**1am**

Compound (+)-**1am** was prepared in 45% yield (54.4 mg) according to the general procedure. Pale yellow oil, (eluent: PE/DCM = 2/1). $[\alpha]_D^{25}$ = +72.3º (c = 1.0, $CHCl_3$). 99.9% ee (determined by HPLC: Chiralpak IE Column, 3/97 *i*-PrOH/hexane, 1.0 mL/min, 254 nm; TR = 20.93 min (minor), 21.67 min (major)).

4-((1*R*,2*S*,4'*S*,5*S*)-3'-(2,6-dimethylphenyl)-1'-tosyl-4',6'-dihydro-1'*H*-spiro[adamantane-2,5'-cyclopenta[*b*]pyrrol]-4'-yl)-*N*,*N*-dimethylaniline ((-)-2am)

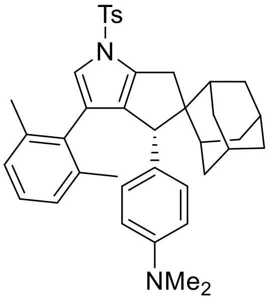

(-)-**2am**

Compound (-)-**2am** was prepared in 45% yield (54.4 mg) according to the general procedure. Pale yellow oil, (eluent: PE/DCM = 2/1). $[\alpha]_D^{25}$ = -144.3º (c = 1.0, $CHCl_3$). 98% ee (determined by HPLC: Chiralpak IE Column, 10/90 *i*-PrOH/hexane, 1.0 mL/min, 254 nm; TR = 8.24 min (minor), 10.37 min (major)). ^{1}H NMR (500 MHz,

$CDCl_3$) δ 7.73 (d, *J* = 8.0 Hz, 2H), 7.30 (d, *J* = 8.0 Hz, 2H), 7.02 – 6.93 (m, 2H), 6.78 (s, 1H), 6.75 (d, *J* = 7.0 Hz, 1H), 6.51 (d, *J* = 8.5 Hz, 2H), 6.37 (d, *J* = 8.5 Hz, 2H), 3.67 (s, 1H), 3.39 (d, *J* = 16.5 Hz, 1H), 2.87 – 2.79 (m, 7H), 2.45 (s, 3H), 2.20 – 2.08 (m, 2H), 1.93 (s, 3H), 1.86 – 1.81 (m, 2H), 1.74 – 1.66 (m, 3H), 1.63 – 1.48 (m, 6H), 1.28 (s, 3H), 1.16 – 1.08 (m, 1H); ^{13}C NMR (125 MHz, $CDCl_3$) δ 148.8, 144.4, 138.6, 137.6, 136.8, 136.4, 136.1, 132.9, 129.6, 129.5, 126.8, 126.6, 126.5, 126.3, 125.1, 121.1, 112.0, 59.9, 51.0, 40.8, 38.7, 37.4, 35.0, 34.8, 34.7, 34.5, 33.9, 33.0, 27.3(2), 27.2(7), 21.6, 20.5, 19.6; IR (neat):2911, 2856, 1614, 1519, 1457, 1369, 1266, 1187, 1174, 1093; HRESIMS Calcd for $[C_{39}H_{44}N_2NaO_2S]^+$ (M + Na^+) 627.3016, found 627.3020.

(*R*)-*N*-(1-(4-(dimethylamino)phenyl)-5-methylhex-1-yn-3-yl)-*N*-((2,6-dimethylphenyl)ethynyl)-4-methylbenzenesulfonamide ((+)-1an)

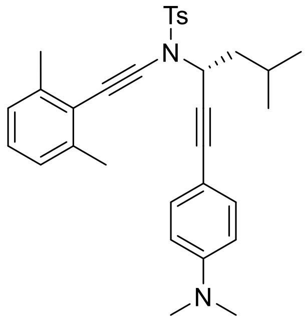

(+)-**1an**

Compound (+)-**1an** was prepared in 38% yield (38.9 mg) according to the general procedure. Pale yellow oil, (eluent: PE/DCM = 2/1). $[\alpha]_D^{25}$ = +40.4º (c = 1.0, $CHCl_3$). 90% ee (determined by HPLC: Chiralpak ODH Column, 1/99 *i*-PrOH/hexane, 1.0 mL/min, 254 nm; TR = 12.54 min (major), 15.36 min (minor)).

(*R*)-4-(3-(2,6-dimethylphenyl)-5,5-dimethyl-1-tosyl-1,4,5,6-tetrahydrocyclopenta[*b*]pyrrol-4-yl)-*N*,*N*-dimethylaniline ((-)-2an)

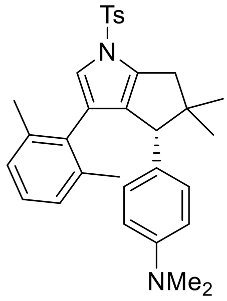

(-)-**2an**

Compound (-)-**2an** was prepared in 37% yield (37.9 mg) according to the general procedure. Pale yellow oil, (eluent: PE/DCM = 2/1). $[\alpha]_D^{25}$ = -13.2º (c = 1.0, $CHCl_3$). 90% ee (determined by HPLC: Chiralpak IC Column, 10/90 *i*-PrOH/hexane, 1.0 mL/min, 254 nm; TR = 6.39 min (minor), 7.50 min (major)). ^{1}H NMR (500 MHz, $CDCl_3$) δ 7.75 (d, *J* = 8.5 Hz, 2H), 7.32 (d, *J* = 8.0 Hz, 2H), 6.98 – 6.87 (m, 3H), 6.80 (d, *J* = 7.5 Hz, 1H), 6.46 (d, *J* = 8.5 Hz, 2H), 6.40 (d, *J* = 9.0 Hz, 2H), 3.24 (s, 1H), 2.82 (s, 6H), 2.80 (d, *J* = 16.0 Hz, 1H), 2.68 (d, *J* = 16.0 Hz, 1H), 2.46 (s, 3H), 1.96 (s, 3H), 1.74 (s, 3H), 1.20 (s, 3H), 0.66 (s, 3H); ^{13}C NMR (125 MHz, $CDCl_3$) δ 144.5, 137.0(4), 137.0(2), 136.85, 136.5, 136.0, 133.0, 129.7, 128.7, 126.9, 126.8, 126.6, 126.5, 125.2, 121.7, 112.1, 55.7, 50.1, 41.2, 40.8, 31.2, 25.9, 21.6, 20.5; IR (neat): 2961, 2919, 1613, 1519, 1458, 1369, 1265, 1186, 1174, 1097; HRESIMS Calcd for $[C_{32}H_{36}N_2NaO_2S]^+$ (M + Na^+) 535.2390, found 535.2388.

(*R*)-*N*-(1-(4-(dimethylamino)phenyl)-5-ethylhept-1-yn-3-yl)-*N*-((2,6-dimethylphenyl)ethynyl)-4-methylbenzenesulfonamide ((+)-1ao)

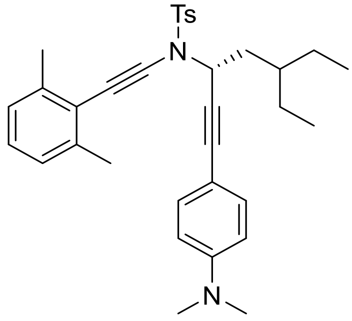

(+)-**1ao**

Compound (+)-**1ao** was prepared in 39% yield (42.1 mg) according to the general procedure. Pale yellow oil, (eluent: PE/DCM = 2/1). $[\alpha]_D^{25}$ = +33.5º (c = 1.0, $CHCl_3$).

91% ee (determined by HPLC: Chiralpak ODH Column, 1/99 *i*-PrOH/hexane, 1.0 mL/min, 254 nm; TR = 9.95 min (major), 12.65 min (minor)).

(*R*)-4-(3-(2,6-dimethylphenyl)-5,5-diethyl-1-tosyl-1,4,5,6-tetrahydrocyclopenta[*b*]pyrrol-4-yl)-*N,N*-dimethylaniline ((-)-2ao)

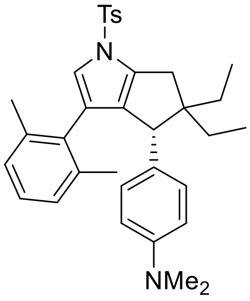

(-)-**2ao**

Compound (-)-**2ao** was prepared in 40% yield (43.2 mg) according to the general procedure. Pale yellow oil, (eluent: PE/DCM = 2/1). $[\alpha]_D^{25}$ = -11.3º (c = 1.0, $CHCl_3$). 93% ee (determined by HPLC: Chiralpak IC Column, 2/98 *i*-PrOH/hexane, 1.0 mL/min, 254 nm; TR = 13.56 min (minor), 17.01 min (major)). ^{1}H NMR (400 MHz, $CDCl_3$) δ 7.74 (d, *J* = 7.2 Hz, 2H), 7.32 (d, *J* = 7.6 Hz, 2H), 6.98 – 6.89 (m, 2H), 6.86 (s, 1H), 6.76 (d, *J* = 6.8 Hz, 1H), 6.45 (d, *J* = 7.6 Hz, 2H), 6.39 (d, *J* = 8.0 Hz, 2H), 3.35 (s, 1H), 2.83 (s, 6H), 2.72 (q, *J* = 16.0 Hz, 2H), 2.45 (s, 3H), 1.97 (s, 3H), 1.61 (s, 3H), 1.55 – 1.46 (m, 2H), 1.11 – 0.89 (m, 2H), 0.82 (t, *J* = 7.2 Hz, 3H), 0.64 (t, *J* = 7.2 Hz, 3H); ^{13}C NMR (100 MHz, $CDCl_3$) δ 148.9, 144.5, 137.2, 137.0, 136.9, 136.7, 136.4, 133.0, 129.7, 129.3, 129.2, 126.9, 126.8, 126.6, 126.5, 125.1, 121.6, 112.0, 56.6, 52.1, 40.7, 38.4, 30.6, 27.7, 21.6, 20.6, 20.3, 8.9, 8.7; IR (neat): 2961, 2920, 1610, 1519, 1460, 1366, 1188, 1175, 1005; HRESIMS Calcd for $[C_{34}H_{40}N_2NaO_2S]^+$ (M + Na$^+$) 563.2703, found 563.2708.

(*R*)-*N*-(1-(4-(dimethylamino)phenyl)-5-propyloct-1-yn-3-yl)-*N*-((2,6-dimethylphenyl)ethynyl)-4-methylbenzenesulfonamide ((+)-1ap)

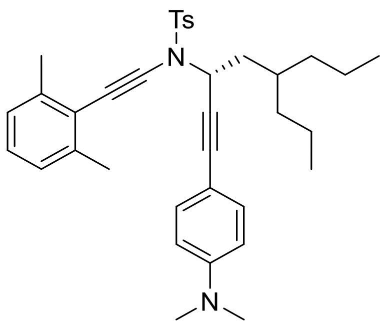

(+)-**1ap**

Compound (+)-**1ap** was prepared in 43% yield (48.9 mg) according to the general procedure. Pale yellow oil, (eluent: PE/DCM = 2/1). $[\alpha]_D^{25}$ = +54.9º (c = 1.0, $CHCl_3$). 91% ee (determined by HPLC: Chiralpak ADH Column, 10/90 *i*-PrOH/hexane, 1.0 mL/min, 254 nm; TR = 8.21 min (minor), 9.37 min (major)).

(*R*)-4-(3-(2,6-dimethylphenyl)-5,5-dipropyl-1-tosyl-1,4,5,6-tetrahydrocyclopenta[*b*]pyrrol-4-yl)-*N*,*N*-dimethylaniline ((-)-2ap)

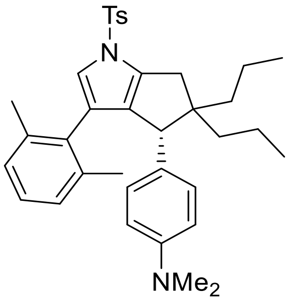

(-)-**2ap**

Compound (-)-**2ap** was prepared in 41% yield (46.6 mg) according to the general procedure. Pale yellow oil, (eluent: PE/DCM = 2/1). $[\alpha]_D^{25}$ = -9.6º (c = 1.0, $CHCl_3$). 91% ee (determined by HPLC: Chiralpak IE Column, 2/98 *i*-PrOH/hexane, 1.0 mL/min, 254 nm; TR = 8.13 min (minor), 9.73 min (major)). ^{1}H NMR (500 MHz, $CDCl_3$) δ 7.74 (d, *J* = 8.5 Hz, 2H), 7.32 (d, *J* = 8.0 Hz, 2H), 6.98 – 6.90 (m, 2H), 6.87 (s, 1H), 6.77 (d, *J* = 7.0 Hz, 1H), 6.44 (d, *J* = 9.0 Hz, 2H), 6.40 (d, *J* = 9.0 Hz, 2H), 3.31 (d, *J* = 1.0 Hz, 1H), 2.83 (s, 6H), 2.75 (d, *J* = 16.5 Hz, 1H), 2.67 (d, *J* = 16.5 Hz, 1H), 2.46 (s, 3H), 1.98 (s, 3H), 1.59 (s, 3H), 1.42 – 1.36 (m, 2H), 1.22 – 1.01 (m, 4H), 0.98 – 0.87 (m, 2H), 0.81 (t, *J* = 7.0 Hz, 3H), 0.62 (t, *J* = 7.5 Hz, 3H); ^{13}C NMR (125 MHz, $CDCl_3$) δ 149.0, 144.5, 137.2, 137.1, 136.9, 136.4, 133.0, 129.7, 129.3, 129.1, 126.9, 126.8, 126.6,

126.5, 125.1, 121.6, 112.2, 56.4, 52.9, 41.8, 40.8, 38.9, 38.1, 21.6, 20.5, 20.3, 17.8, 17.7, 14.9, 14.8; IR (neat): 2925, 2853, 1610, 1522, 1369, 1277, 1185, 1105, 1097, 815; HRESIMS Calcd for $[C_{36}H_{44}N_2NaO_2S]^+$ (M + Na$^+$) 591.3016, found 591.3028.

(*R*)-*N*-(1-(4-(dimethylamino)phenyl)-5,5-diphenylpent-1-yn-3-yl)-*N*-((2,6-dimethylphenyl)ethynyl)-4-methylbenzenesulfonamide ((+)-1aq)

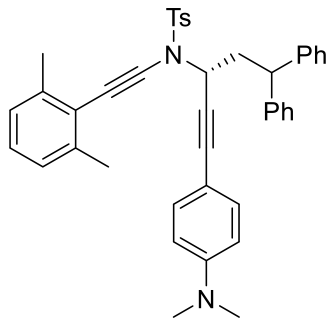

(+)-**1aq**

Compound (+)-**1aq** was prepared in 40% yield (50.9 mg) according to the general procedure at -20 °C. Pale yellow oil, (eluent: PE/DCM = 2/1). $[\alpha]_D^{25}$ = +21.3º (c = 1.0, $CHCl_3$). 66% ee (determined by HPLC: Chiralpak ADH Column, 20/80 *i*-PrOH/hexane, 1.0 mL/min, 254 nm; TR = 4.95 min (major), 11.43 min (minor)).

(*S*)-4-(3-(2,6-dimethylphenyl)-5,5-diphenyl-1-tosyl-1,4,5,6-tetrahydrocyclopenta[*b*]pyrrol-4-yl)-*N*,*N*-dimethylaniline ((-)-2aq)

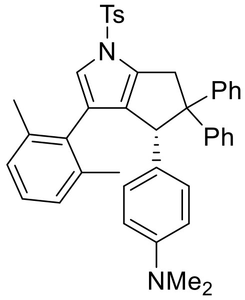

(-)-**2aq**

Compound (-)-**2aq** was prepared in 38% yield (48.2 mg) according to the general procedure at -20 °C. Pale yellow solid (mp 174–175 °C), (eluent: PE/DCM = 2/1). $[\alpha]_D^{25}$ = -45.7º (c = 1.0, $CHCl_3$). 71% ee (determined by HPLC: Chiralpak IE Column, 20/80 *i*-PrOH/hexane, 1.0 mL/min, 254 nm; TR = 7.37 min (major), 14.40 min (minor)).

^{1}H NMR (500 MHz, $CDCl_3$) δ 7.46 (d, *J* = 8.5 Hz, 2H), 7.19 – 7.15 (m, 2H), 7.13 – 7.06 (m, 5H), 7.01 – 6.92 (m, 5H), 6.91 – 6.87 (m, 2H), 6.85 (s, 1H), 6.77 (d, *J* = 7.0 Hz, 1H), 6.34 (d, *J* = 9.0 Hz, 2H), 6.20 (d, *J* = 9.0 Hz, 2H), 4.44 (s, 1H), 4.24 (d, *J* = 16.0 Hz, 1H), 3.45 (d, *J* = 16.0 Hz, 1H), 2.72 (s, 6H), 2.39 (s, 3H), 1.97 (s, 3H), 1.38 (s, 3H); ^{13}C NMR (125 MHz, $CDCl_3$) δ 150.9, 149.0, 145.0, 144.3, 138.1, 137.7, 136.9, 136.2, 135.2, 132.6, 129.7, 129.4, 128.9, 127.6, 127.5, 127.3, 127.1, 126.8, 126.5, 126.3, 125.5, 125.4, 124.7, 122.1, 112.2, 66.6, 54.0, 40.8, 40.5, 21.6, 20.6, 20.0; IR (neat): 2925, 2851, 1610, 1593, 1527, 1371, 1175, 1115, 818; HRESIMS Calcd for $[C_{42}H_{40}N_2NaO_2S]^+$ (M + Na^+) 659.2703, found 659.2713.

(*R*)-*N*-(1-(4-(dimethylamino)phenyl)-5,7-dimethyloct-1-yn-3-yl)-*N*-((2,6-dimethylphenyl)ethynyl)-4-methylbenzenesulfonamide ((+)-1ar)

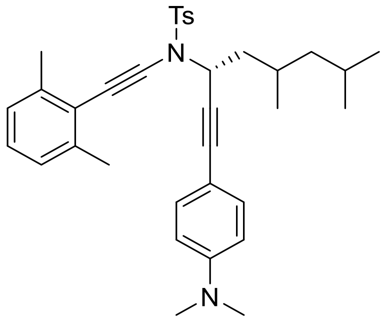

(+)-**1ar**

Compound (+)-**1ar** was prepared in 41% yield (45.5 mg, 1:1 dr) according to the general procedure. Pale yellow oil, (eluent: PE/DCM = 2/1). $[\alpha]_D^{25}$ = +106.3º (c = 1.0, $CHCl_3$). 99% ee (determined by HPLC: Chiralpak IE Column, 10/90 *i*-PrOH/hexane, 1.0 mL/min, 254 nm; TR = 15.27 min (major), 16.85 min (minor)). 99% ee (determined by HPLC: Chiralpak IE Column, 10/90 *i*-PrOH/hexane, 1.0 mL/min, 254 nm; TR = 18.00 min (minor), 19.46 min (major)).

4-((4*R*)-3-(2,6-dimethylphenyl)-5-isobutyl-5-methyl-1-tosyl-1,4,5,6-tetrahydrocyclopenta[*b*]pyrrol-4-yl)-*N*,*N*-dimethylaniline ((-)-2ar)

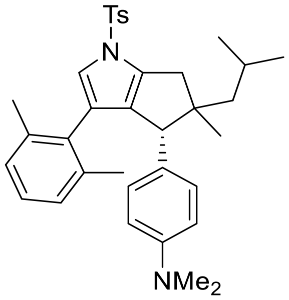

(-)-**2ar**

Compound (-)-**2ar** was prepared in 45% yield (50.0 mg) according to the general procedure. Pale yellow oil, (eluent: PE/DCM = 2/1). dr = 1:1.2 (determined by ^{1}H NMR analysis of the reaction mixture). $[\alpha]_D^{25}$ = -74.9° (c = 1.0, $CHCl_3$). 90%, 92% ee (determined by HPLC: Chiralpak IE Column, 5/95 *i*-PrOH/hexane, 1.0 mL/min, 254 nm; TR_1 =7.97 min (minor), 9.06 min (major); TR_2 =8.39 min (minor), 9.07 min (major)). ^{1}H NMR (600 MHz, $CDCl_3$) δ 7.79 – 7.70 (m, 2H), 7.34 – 7.29 (m, 2H), 6.98 – 6.84 (m, 3H), 6.83 – 6.74 (m, 1H), 6.51 (d, *J* = 8.4 Hz, 1H), 6.45 – 6.34 (m, 3H), 3.37 (s, 0.5H), 3.17 (s, 0.5H), 2.82 (s, 6H), 2.81 – 2.78 (m, 0.5H), 2.73 (s, 1H), 2.62 (d, *J* = 15.6 Hz, 0.5H), 2.45 (d, *J* = 7.8 Hz, 3H), 1.96 (d, *J* = 4.2 Hz, 3H), 1.77 (s, 1.5H), 1.57 (s, 1.5H), 1.46 – 1.40 (m, 1H), 1.14 (s, 1.5H), 1.04 – 0.96 (m, 2H), 0.84 – 0.79 (m, 3H), 0.69 (d, *J* = 6.6 Hz, 1.5H), 0.62 (d, *J* = 6.6 Hz, 1.5H), 0.60 (s, 1.5H); ^{13}C NMR (150 MHz, $CDCl_3$) δ 149.1, 149.0, 144.6, 144.5, 137.2, 136.9(9), 136.9(5), 136.9(3), 136.9(1), 136.8(5), 136.4, 135.7, 132.9(8), 132.9(6), 129.7(4), 129.7(0), 128.9, 126.9, 126.8(3), 126.8(0), 126.6, 126.5(3), 126.5(0), 126.4, 125.3, 124.9, 121.7, 121.5, 112.3, 112.0, 55.6, 53.7, 53.2, 53.1, 41.2, 40.9, 40.7, 39.9, 39.5, 36.2, 34.0, 33.9, 28.7, 28.5 28.1 24.1, 22.7(1), 22.6(7), 22.5, 22.3, 21.6, 21.6, 20.6(0), 20.5(7), 20.2; IR (neat): 2926, 2850, 1611, 1556, 1527, 1377, 1175, 1111, 815; HRESIMS Calcd for $[C_{35}H_{42}N_2NaO_2S]^+$ (M + Na^+) 577.2859, found 577.2851.

(*R*)-*N*-(1-(4-(dimethylamino)phenyl)-5-phenylpent-1-yn-3-yl)-*N*-((2,6-dimethylphenyl)ethynyl)-4-methylbenzenesulfonamide ((+)-1as)

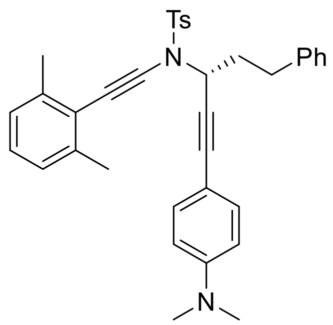

(+)-**1as**

Compound (+)-**1as** was prepared in 35% yield (39.3 mg) according to the general procedure. Pale yellow oil, (eluent: PE/DCM = 2/1). $[\alpha]_D^{25}$ = +88.1° (c = 1.0, $CHCl_3$). 94% ee (determined by HPLC: Chiralpak IE Column, 10/90 *i*-PrOH/hexane, 1.0 mL/min, 254 nm; TR = 10.66 min (minor), 12.42 min (major)).

4-((4*R*)-3-(2,6-dimethylphenyl)-5-phenyl-1-tosyl-1,4,5,6-tetrahydrocyclopenta[*b*]pyrrol-4-yl)-*N*,*N*-dimethylaniline ((-)-2as)

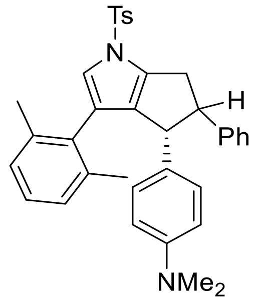

(-)-**2as**

Compound (-)-**2as** was prepared in 41% yield (45.9 mg) according to the general procedure. Pale yellow oil, (eluent: PE/DCM = 2/1). dr = 1:1.3 (determined by ^{1}H NMR analysis of the reaction mixture). $[\alpha]_D^{25}$ = -65.3° (c = 1.0, $CHCl_3$). 88% ee, 81% ee (determined by HPLC: Chiralpak IG Column, 3/97 *i*-PrOH/hexane, 1.0 mL/min, 254 nm; TR = 16.84 min, 18.96 min, 21.01 min, 23.09 min). ^{1}H NMR (600 MHz, $CDCl_3$) δ 7.86 – 7.70 (m, 2H), 7.41 – 7.29 (m, 2H), 7.19 – 6.76 (m, 9H), 6.65 – 6.37 (m, 2H), 6.27 – 6.08 (m, 2H), 4.31 – 4.22 (m, 0.5H), 3.93 (d, *J* = 7.2 Hz, 0.5H), 3.79 – 3.68 (m, 1H), 3.63 – 3.54 (m, 0.5H), 3.33 – 3.15 (m, 1H), 3.07 – 2.95 (m, 0.5H), 2.86 – 2.68 (m, 6H), 2.48 (s, 3H), 2.07 – 1.93 (m, 3H), 1.78 – 1.69 (m, 3H); ^{13}C NMR (150 MHz, $CDCl_3$) δ 149.3, 149.0, 146.5, 144.7, 140.4, 137.1(2), 137.0(5), 136.9, 136.3(3),

136.2(5), 135.9, 135.8, 132.9, 132.6, 132.1, 129.9, 128.8, 128.5, 128.4, 128.0, 127.5, 127.4, 127.1, 126.9, 126.8, 126.7(1), 126.7(0), 126.6, 126.5, 126.4, 126.3, 125.9, 124.5, 124.3, 122.4, 121.8, 112.6, 112.1, 58.9, 56.0, 53.7, 49.6, 40.8, 40.8, 35.0, 31.3, 21.7, 20.6, 20.4; IR (neat): 2930, 2853, 1615, 1590, 1375, 1160, 1015, 815; HRESIMS Calcd for $[C_{36}H_{36}N_2NaO_2S]^+$ (M + Na^+) 583.2390, found 583.2382.

5. Synthetic Transformations

(*S*)-4-(3'-(2,6-dimethylphenyl)-1'-tosyl-4',6'-dihydro-1'*H*-spiro[cyclohexane-1,5'-cyclopenta[*b*]pyrrol]-4'-yl)-*N*,*N*-dimethylaniline ((+)-2a)

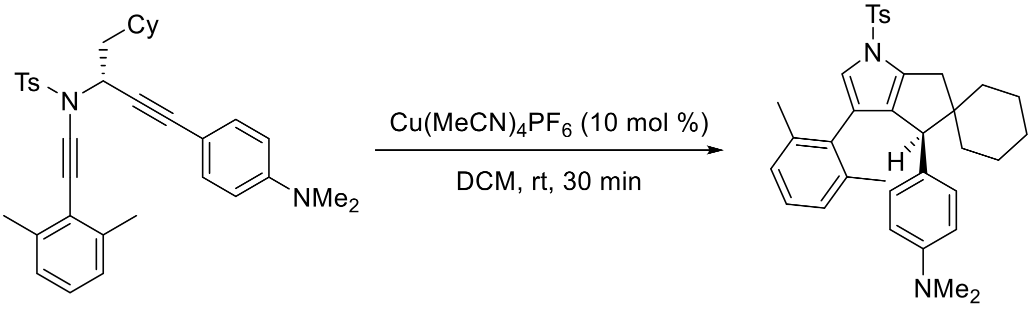

(+)-**1a**, 96% ee (+)-**2a**, 81%, 96% ee

The powered Cu(MeCN)$_4$PF$_6$ (0.01 mmol, 3.8 mg) was introduced into an oven-dried Schlenk tube under N_2 atmosphere. After DCM (1 mL) was injected into the Schlenk tube, *N*-propargyl ynamide (+)-**1a** (0.1 mmol, 55.3 mg, 96% ee) in DCM (1 mL) was added into the system. The resulting mixture was stirred at room temperature and the progress of the reaction was monitored by TLC. After concentration in vacuo, the residue was purified by column chromatography on silica gel (eluent: hexanes/EtOAc) to give the final product (+)-**2a** in 81% yield (44.7 mg). Pale yellow oil, (eluent: PE/DCM = 2/1). $[\alpha]_D^{25}$ = +47.3° (c = 1.0, $CHCl_3$). 96% ee (determined by HPLC: Chiralpak IE Column, 2/98 *i*-PrOH/hexane, 1.0 mL/min, 254 nm; TR = 9.93 min (major), 11.36 min (minor)).

(*R*)-4-(3'-(2,6-dimethylphenyl)-4',6'-dihydro-1'*H*-spiro[cyclohexane-1,5'-cyclopenta[*b*]pyrrol]-4'-yl)-*N*,*N*-dimethylaniline (3aa)

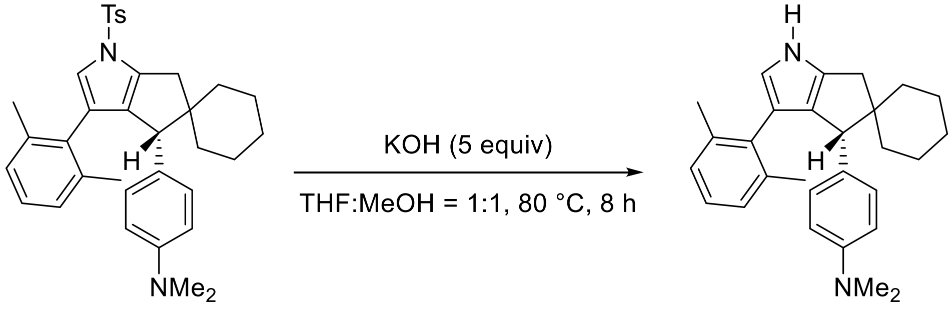

(-)-**2a**, 94% ee **3aa**, 78%, 94% ee

Compound **3aa** was prepared in 78% yield (0.1 mmol scale, 31.1 mg) according to the known procedure[2]. To a solution of the compound (-)-**2a** (0.1 mmol, 55.3 mg) in THF

(1 mL) and MeOH (1 mL) was added KOH (0.5 mmol, 28.1 mg), and the reaction was stirred at 80 °C for 8 hours. The progress of the reaction was monitored by TLC. Upon completion, the reaction was quenched with water and extracted with DCM (3 x 5 mL). The combined organic layers were dried over anhydrous $MgSO_4$ and concentrated under vacuum. The crude product was purified by column chromatography on silica gel (eluent: PE/EtOAc = 5/1) to afford the desired product **3aa**. Colourless oil. $[\alpha]_D^{25}$ = -33.6° (c = 1.0, $CHCl_3$). 94% ee (determined by HPLC: Chiralpak IE Column, 5/95 *i*-PrOH/hexane, 1.0 mL/min, 254 nm; TR = 10.11 min (minor), 11.64 min (major)). ^{1}H NMR (400 MHz, $CDCl_3$) δ 7.85 (s, 1H), 6.92 (d, *J* = 4.0 Hz, 2H), 6.85 – 6.80 (m, 1H), 6.71 (d, *J* = 8.4 Hz, 2H), 6.48 (d, *J* = 8.8 Hz, 2H), 6.42 (d, *J* = 2.4 Hz, 1H), 3.41 (s, 1H), 2.82 (s, 6H), 2.67 (s, 2H), 2.11 (s, 3H), 1.84 (s, 3H), 1.63 – 1.39 (m, 6H), 1.21 – 1.11 (m, 3H), 0.99 – 0.90 (m, 1H); ^{13}C NMR (100 MHz, $CDCl_3$) δ 148.8, 137.8, 137.5, 136.0, 134.3, 131.3, 129.2, 129.1, 126.6, 126.3, 125.8, 118.9, 117.4, 112.0, 56.3, 54.5, 40.9, 39.2, 35.7, 35.5, 26.2, 23.7, 23.6, 20.9; IR (neat): 2920, 2850, 1616, 1595, 1515, 1255, 1149, 1118, 815, 617; HRESIMS Calcd for $[C_{28}H_{35}N_2]^+$ (M + H^+) 399.2795, found 399.2795.

4-((4'*R*,6a'*R*)-3'-(2,6-dimethylphenyl)-1'-tosyl-2',4',6',6a'-tetrahydro-1'*H*-spiro[cyclohexane-1,5'-cyclopenta[*b*]pyrrol]-4'-yl)-*N,N*-dimethylaniline (3ab)

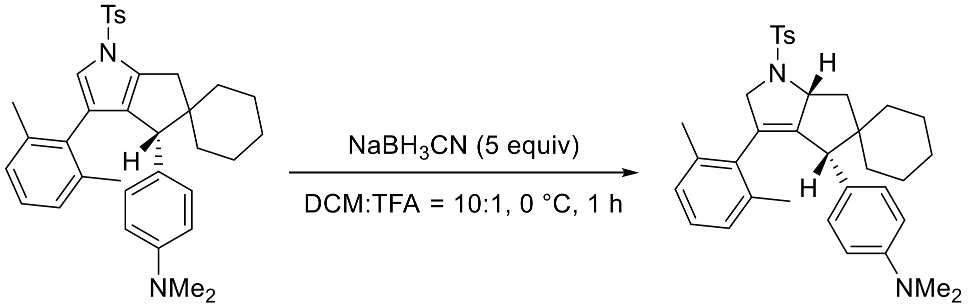

(-)-**2a**, 94% ee

3ab, 95%, 95% ee
>20:1 dr

Compound **3ab** was prepared in 95% yield (0.1 mmol scale, 52.7 mg) with >20:1 dr according to the known procedure[3]. To a solution of the compound (-)-**2a** (0.1 mmol, 55.3 mg) in DCM (1 mL) and TFA (0.1 mL) was added $NaBH_3CN$ (0.5 mmol, 31.4 mg), and the reaction was stirred at 0 °C for 1 hours. The progress of the reaction was

monitored by TLC. Upon completion, the reaction was quenched with aqueous $NaHCO_3$ and extracted with DCM (3 x 5 mL). The combined organic layers were dried over anhydrous $MgSO_4$ and concentrated under vacuum. The crude product was purified by column chromatography on silica gel (eluent: PE/EtOAc = 5/1) to afford the desired product **3ab**. Colourless oil. $[\alpha]_D^{25}$ = -86.4° (c = 1.0, $CHCl_3$). 95% ee (determined by HPLC: Chiralpak ADH Column, 5/95 *i*-PrOH/hexane, 1.0 mL/min, 254 nm; TR = 5.82 min (minor), 7.36 min (major)). ^{1}H NMR (500 MHz, $CDCl_3$) δ 7.76 (d, *J* = 8.0 Hz, 2H), 7.36 (d, *J* = 8.0 Hz, 2H), 6.82 – 6.73 (m, 2H), 6.64 – 6.52 (m, 3H), 6.21 (d, *J* = 8.5 Hz, 2H), 4.55 – 4.37 (m, 2H), 3.98 (dt, *J* = 12.0, 3.5 Hz, 1H), 2.99 (d, *J* = 3.5 Hz, 1H), 2.73 (s, 6H), 2.45 (s, 3H), 2.43 – 2.37 (m, 1H), 2.32 (s, 3H), 1.92 – 1.82 (m, 1H), 1.74 (s, 3H), 1.57 – 1.26 (m, 8H), 1.06 – 0.83 (m, 2H); ^{13}C NMR (125 MHz, $CDCl_3$) δ 148.9, 145.6, 143.5, 136.4, 135.6, 132.9, 131.8, 130.3, 129.7, 128.1, 127.2, 127.1, 126.6, 125.1, 124.7, 111.4, 68.6, 61.8, 55.1, 50.1, 42.0, 40.8, 39.3, 36.5, 25.6, 23.4, 22.3, 21.5, 20.4, 20.3; IR (neat): 2926, 2851, 1617, 1521, 1446, 1348, 1164, 1069, 812, 763; HRESIMS Calcd for $[C_{35}H_{42}N_2NaO_2S]^+$ (M + Na^+) 577.2859, found 577.2868.

dimethyl (1'*R*,6'*S*)-1'-(4-(dimethylamino)phenyl)-7'-(2,6-dimethylphenyl)-8'-tosyl-1',6'-dihydro-3'*H*-spiro[cyclohexane-1,2'-[3a,6]epiminoindene]-4',5'-dicarboxylate (3ac)

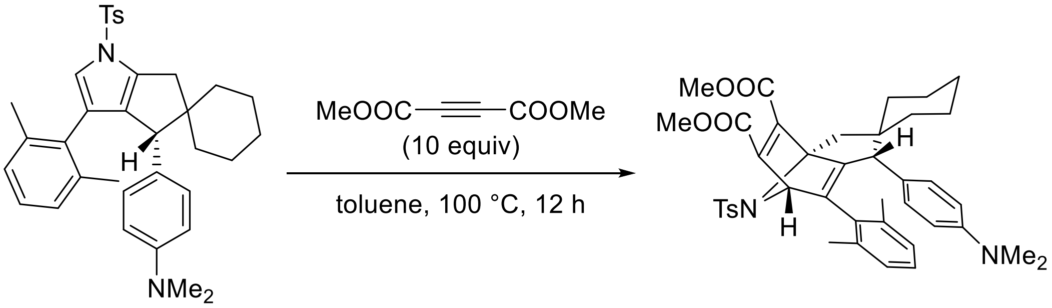

(-)-**2a**, 94% ee

3ac, 83%, 95% ee
>20:1 dr

Compound **3ac** was prepared in 83% yield (0.1 mmol scale, 57.7 mg) with >20:1 dr according to the known procedure[5]. To a solution of the compound (-)-**2a** (0.1 mmol, 55.3 mg) in toluene (1 mL) was added dimethyl acetylenedicarboxylate (1 mmol, 123

μL), and the reaction was stirred at 100 °C for 12 hours. The progress of the reaction was monitored by TLC. Upon completion, the reaction was concentrated under vacuum. The crude product was purified by column chromatography on silica gel (eluent: PE/EtOAc = 3/1) to afford the desired product **3ac**. Colourless solid (mp 188–189 °C). $[\alpha]_D^{25}$ = -44.9° (c = 1.0, $CHCl_3$). 95% ee (determined by HPLC: Chiralpak IE Column, 30/70 *i*-PrOH/hexane, 1.0 mL/min, 254 nm; TR = 8.29 min (minor), 9.71 min (major)). ^1H NMR (500 MHz, $CDCl_3$) δ 7.63 (d, *J* = 8.0 Hz, 2H), 7.29 – 7.26 (m, 2H), 7.00 (d, *J* = 7.5 Hz, 1H), 6.90 – 6.86 (m, 1H), 6.61 – 6.43 (m, 3H), 6.22 (d, *J* = 8.5 Hz, 2H), 5.52 (s, 1H), 3.72 (s, 3H), 3.56 (s, 3H), 3.39 (s, 1H), 3.23 (d, *J* = 15.5 Hz, 1H), 2.76 (s, 6H), 2.50 (s, 3H), 2.42 (s, 3H), 2.04 (dd, *J* = 15.5, 1.5 Hz, 1H), 1.73 – 1.66 (m, 1H), 1.59 – 1.47 (m, 6H), 1.45 (s, 3H), 0.92 – 0.79 (m, 3H); ^{13}C NMR (100 MHz, $CDCl_3$) δ 164.9, 162.0, 161.5, 154.1, 149.2, 146.7, 143.6, 141.8, 136.4, 135.8, 135.5, 132.2, 130.6, 129.8, 128.5, 127.4, 127.2, 126.9, 121.7, 111.0, 86.8, 76.2, 57.9, 52.9, 51.9(3), 51.8(8), 40.6, 37.3, 30.8, 30.1, 25.6, 23.5, 21.9, 21.5, 20.6, 19.7; IR (neat): 2925, 2850, 1636, 1517, 1453, 1349, 1159, 1106, 650; HRESIMS Calcd for $[C_{41}H_{46}N_2NaO_6S]^+$ (M + Na^+) 717.2969, found 717.2960.

(*R*)-4'-([1,1'-biphenyl]-4-yl)-3'-(2,6-dimethylphenyl)-1'-tosyl-4',6'-dihydro-1'*H*-spiro[cyclohexane-1,5'-cyclopenta[*b*]pyrrole] (3ad)

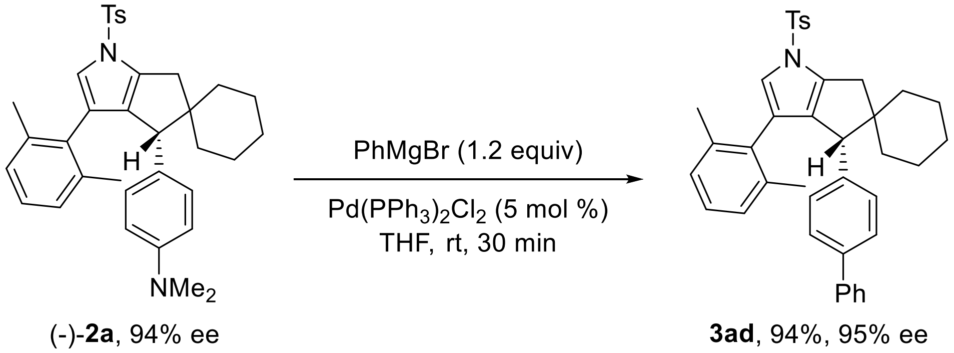

Compound **3ad** was prepared in 94% yield (0.1 mmol scale, 55.1 mg) according to the known procedure[3]. To a dry Schlenk tube were added the compound (-)-**2a** (0.1 mmol, 55.3 mg), $Pd(PPh_3)_2Cl_2$ (0.005 mmol, 3.5 mg) and freshly distilled THF (1 mL) under nitrogen atmosphere. Next, phenyl magnesium bromide (1 M, 0.12 mmol, 120 μL) was added slowly at room temperature and stirred at this temperature for 30 min. The

progress of the reaction was monitored by TLC. Upon completion, the reaction was quenched with aqueous NH_4Cl. The resulting mixture was extracted with DCM (3 x 5 mL). The combined organic layers were dried over anhydrous $MgSO_4$ and concentrated under vacuum. The crude product was purified by column chromatography on silica gel (eluent: PE/EtOAc = 8/1) to afford the desired product **3ad**. Colourless oil. $[\alpha]_D^{25}$ = -54.6° (c = 1.0, $CHCl_3$). 95% ee (determined by HPLC: Chiralpak IE Column, 5/95 *i*-PrOH/hexane, 1.0 mL/min, 254 nm; TR = 8.50 min (major), 9.58 min (minor)). ^{1}H NMR (400 MHz, $CDCl_3$) δ 7.77 (d, *J* = 8.4 Hz, 2H), 7.53 – 7.45 (m, 2H), 7.39 – 7.23 (m, 7H), 6.99 – 6.86 (m, 3H), 6.76 (d, *J* = 6.8 Hz, 1H), 6.67 (d, *J* = 8.0 Hz, 2H), 3.36 (s, 1H), 2.97 (d, *J* = 16.4 Hz, 1H), 2.79 (d, *J* = 16.4 Hz, 1H), 2.46 (s, 3H), 1.97 (s, 3H), 1.74 – 1.64 (m, 1H), 1.61 (s, 3H), 1.59 – 1.42 (m, 4H), 1.30 – 1.08 (m, 4H), 1.02 – 0.90 (m, 1H); ^{13}C NMR (100 MHz, $CDCl_3$) δ 144.7, 140.9, 139.7, 138.7, 137.3, 136.9, 136.8, 136.4, 135.9, 132.7, 129.7, 128.9, 128.6, 126.9(3), 126.8(8), 126.8, 126.7, 126.0, 125.0, 121.7, 56.2, 54.0, 39.1, 37.1, 35.1, 25.9, 23.3, 21.6, 20.6, 20.2; IR (neat): 2925, 2852, 1646, 1597, 1487, 1415, 1304, 1269, 1051, 812; HRESIMS Calcd for $[C_{39}H_{39}NNaO_2S]^+$ (M + Na^+) 608.2594, found 608.2598.

(*R*)-4-(3'-(2,6-dimethylphenyl)-1'-tosyl-4',6'-dihydro-1'*H*-spiro[cyclohexane-1,5'-cyclopenta[*b*]pyrrol]-4'-yl)aniline (3fa)

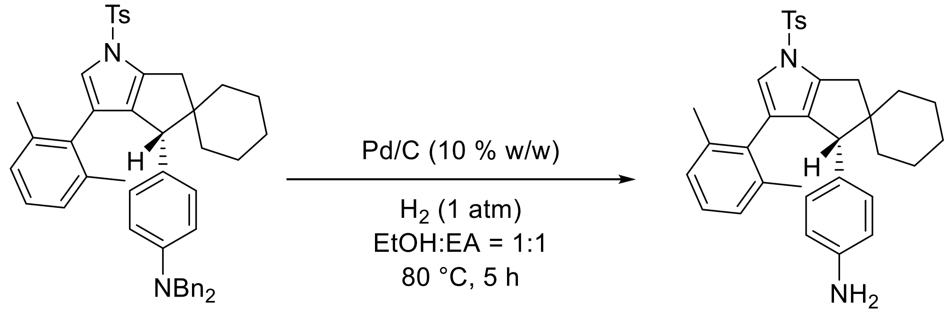

(-)-**2f**, 95% ee   **3fa**, 90%, 95% ee

Compound **3fa** was prepared in 90% yield (0.1 mmol scale, 47.3 mg) according to the known procedure[3]. To a solution of the compound (-)-**2f** (0.1 mmol, 70.5 mg) in MeOH (1 mL) and EtOAc (1 mL) was added Pd/C (10% w/w, 7.1 mg), and the reaction was stirred at 80 °C under H_2 (1 atm) for 5 hours. The progress of the reaction was monitored by TLC. Upon completion, the reaction was concentrated under vacuum. The crude

product was purified by column chromatography on silica gel (eluent: PE/EtOAc = 4/1) to afford the desired product **3fa**. Colourless oil. $[\alpha]_D^{25}$ = -88.1° (c = 1.0, $CHCl_3$). 95% ee (determined by HPLC: Chiralpak ADH Column, 10/90 *i*-PrOH/hexane, 1.0 mL/min, 254 nm; TR = 14.33 min (major), 16.44 min (minor)). ^1H NMR (600 MHz, $CDCl_3$) δ 7.74 (d, *J* = 8.4 Hz, 2H), 7.31 (d, *J* = 8.4 Hz, 2H), 6.95 (t, *J* = 7.2 Hz, 1H), 6.90 (d, *J* = 7.2 Hz, 1H), 6.87 (s, 1H), 6.79 (d, *J* = 7.2 Hz, 1H), 6.39 (d, *J* = 7.8 Hz, 2H), 6.34 (d, *J* = 9.0 Hz, 2H), 3.43 (s, 2H), 3.21 (s, 1H), 2.90 (d, *J* = 16.2 Hz, 1H), 2.71 (d, *J* = 16.2 Hz, 1H), 2.45 (s, 3H), 1.94 (s, 3H), 1.65 (s, 3H), 1.63 – 1.36 (m, 6H), 1.22 – 1.11 (m, 2H), 1.09 – 1.02 (m, 1H), 0.95 – 0.86 (m, 1H); ^{13}C NMR (150 MHz, $CDCl_3$) δ 144.5, 144.2, 137.1, 136.9, 136.9, 136.4, 136.2, 132.9, 130.7, 129.7, 129.2, 126.8(4), 126.8(3), 126.5(0), 126.4(7), 125.1, 121.6, 114.4, 55.8, 53.8, 38.9, 36.9, 35.1, 26.0, 23.3, 21.6, 20.5, 20.3; IR (neat): 3440, 2925, 2851, 1620, 1610, 1596, 1530, 1343, 1267, 1101, 815; HRESIMS Calcd for $[C_{33}H_{37}N_2O_2S]^+$ (M + H^+) 525.2570, found 525.2577.

(*R*)-4-bromo-*N*-(4-(3'-(2,6-dimethylphenyl)-1'-tosyl-4',6'-dihydro-1'*H*-spiro[cyclohexane-1,5'-cyclopenta[*b*]pyrrol]-4'-yl)phenyl)benzamide (3fb)

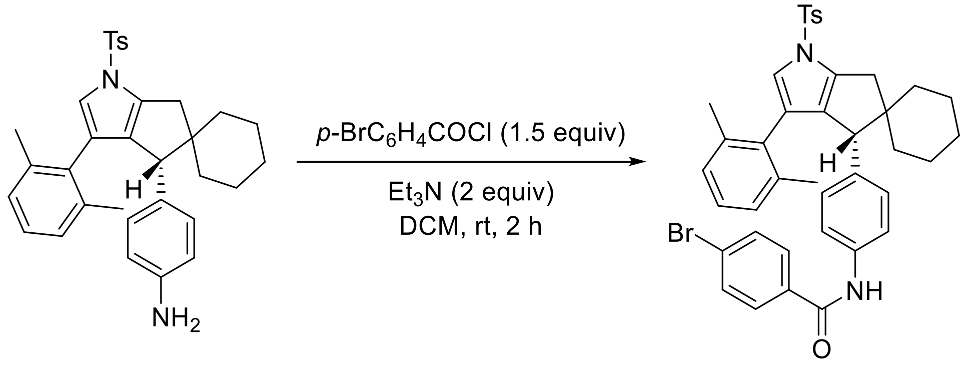

3fa, 95% ee **3fb**, 81%, 96% ee

To the solution of the **3fa** (0.1 mmol, 52.5 mg) and Et_3N (0.2 mmol, 28 μL) in DCM (3 mL) was added *p*-BrC_6H_4COCl (0.15 mmol, 38.3 mg) at room temperature and stirred for additional 2 h. The progress of the reaction was monitored by TLC. Upon completion, the reaction was quenched with water. The resulting mixture was extracted with DCM (3 x 5 mL). The combined organic layers was dried over anhydrous $MgSO_4$ and concentrated under vacuum. The residue was purified by column chromatography on silica gel (eluent: PE/EtOAc = 8/1) to give the desired product **3fb** in 81% yield

(57.3 mg). Colourless solid (mp 144–145 °C). $[\alpha]_D^{25}$ = -62.2º (c = 1.0, $CHCl_3$). 96% ee (determined by HPLC: Chiralpak ODH Column, 5/95 *i*-PrOH/hexane, 1.0 mL/min, 254 nm; TR = 14.35 min (major), 15.05 min (minor)). ^{1}H NMR (600 MHz, $CDCl_3$) δ 7.86 (s, 1H), 7.74 (d, *J* = 8.4 Hz, 2H), 7.65 (d, *J* = 8.4 Hz, 2H), 7.53 (d, *J* = 8.4 Hz, 2H), 7.33 (d, *J* = 8.4 Hz, 2H), 7.30 (d, *J* = 8.4 Hz, 2H), 6.97 – 6.86 (m, 3H), 6.77 (d, *J* = 7.2 Hz, 1H), 6.61 (d, *J* = 7.8 Hz, 2H), 3.33 (s, 1H), 2.93 (d, *J* = 16.2 Hz, 1H), 2.74 (d, *J* = 16.2 Hz, 1H), 2.47 (s, 3H), 1.96 (s, 3H), 1.73 – 1.61 (m, 5H), 1.60 – 1.31 (m, 6H), 1.06 – 1.01 (m, 1H), 0.90 – 0.86 (m, 1H); ^{13}C NMR (150 MHz, $CDCl_3$) δ 164.6, 144.9, 137.3, 137.0, 136.9, 136.6, 136.2, 135.8, 135.4, 133.8, 132.7, 131.8, 129.8, 129.0, 128.6, 126.9(4), 126.9(1), 126.6, 126.5, 126.3, 124.9, 121.7, 119.2, 56.1, 54.1, 38.9, 36.8, 35.1, 25.9, 23.2(1), 23.1(9), 21.6, 20.5, 20.4; IR (neat): 2924, 2853, 1615, 1519, 1488, 1370, 1124, 1102, 816; HRESIMS Calcd for $[C_{40}H_{40}BrN_2O_3S]^+$ (M + H^+) 707.1938, found 707.1946.

dimethyl (1'*R*,6'*S*)-1'-(4-(dibenzylamino)phenyl)-7'-(2,6-dimethylphenyl)-8'-tosyl-1',6'-dihydro-3'*H*-spiro[cyclohexane-1,2'-[3a,6]epiminoindene]-4',5'-dicarboxylate (3fc)

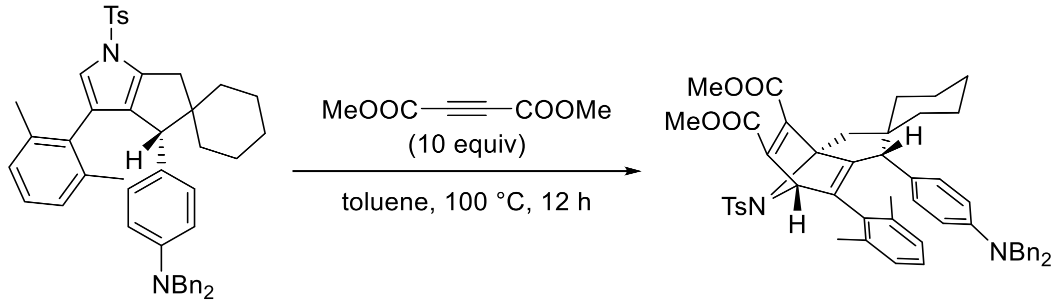

(-)-**2f**, 95% ee

3fc, 87%, 96% ee
>20:1 dr

Compound **3fc** was prepared in 87% yield (0.1 mmol scale, 73.6 mg) with >20/1 dr according to the known procedure[5]. To a solution of the compound (-)-**2f** (0.1 mmol, 70.5 mg) in toluene (1 mL) was added dimethyl acetylenedicarboxylate (1 mmol, 123 μL), and the reaction was stirred at 100 °C for 12 hours. The progress of the reaction was monitored by TLC. Upon completion, the reaction was concentrated under vacuum. The crude product was purified by column chromatography on silica gel (eluent:

PE/EtOAc = 3/1) to afford the desired product **3ac**. Colourless solid (mp 177–178 °C). $[\alpha]_D^{25}$ = -120.5º (c = 1.0, $CHCl_3$). 96% ee (determined by HPLC: Chiralpak IE Column, 20/80 *i*-PrOH/hexane, 1.0 mL/min, 254 nm; TR = 12.37 min (minor), 15.20 min (major)). ^{1}H NMR (400 MHz, $CDCl_3$) δ 7.63 (d, *J* = 8.4 Hz, 2H), 7.32 – 7.22 (m, 8H), 7.09 (d, *J* = 7.2 Hz, 4H), 6.94 – 6.77 (m, 2H), 6.58 (d, *J* = 7.2 Hz, 1H), 6.49 (d, *J* = 8.0 Hz, 2H), 6.21 (d, *J* = 8.4 Hz, 2H), 5.52 (s, 1H), 4.41 (q, *J* = 16.8 Hz, 4H), 3.71 (s, 3H), 3.56 (s, 3H), 3.34 (s, 1H), 3.22 (d, *J* = 14.8 Hz, 1H), 2.43 (s, 3H), 2.42 (s, 3H), 2.04 (d, *J* = 15.2 Hz, 1H), 1.72 – 1.49 (m, 6H), 1.45 (s, 3H), 1.39 – 1.31 (m, 2H), 1.01 – 0.86 (m, 2H); ^{13}C NMR (150 MHz, $CDCl_3$) δ 171.1, 164.9, 162.0, 161.9, 154.1, 147.7, 146.7, 143.6, 141.7, 138.7, 136.5, 135.9, 135.4, 132.1, 130.9, 129.8, 128.5, 128.4, 127.4, 127.2, 126.8, 126.7, 126.5, 121.7, 110.7, 86.7, 76.1, 60.4, 57.9, 53.7, 52.8, 52.0, 51.9, 37.3, 30.8, 30.2, 25.6, 23.5, 21.9, 21.5, 21.0, 20.5, 19.8, 14.2; IR (neat): 2924, 2851, 1626, 1518, 1453, 1350, 1243, 1157, 1106, 815; HRESIMS Calcd for $[C_{53}H_{55}N_2O_6S]^+$ (M + Na^+) 847.3775, found 847.3784.

6. Crystal Data

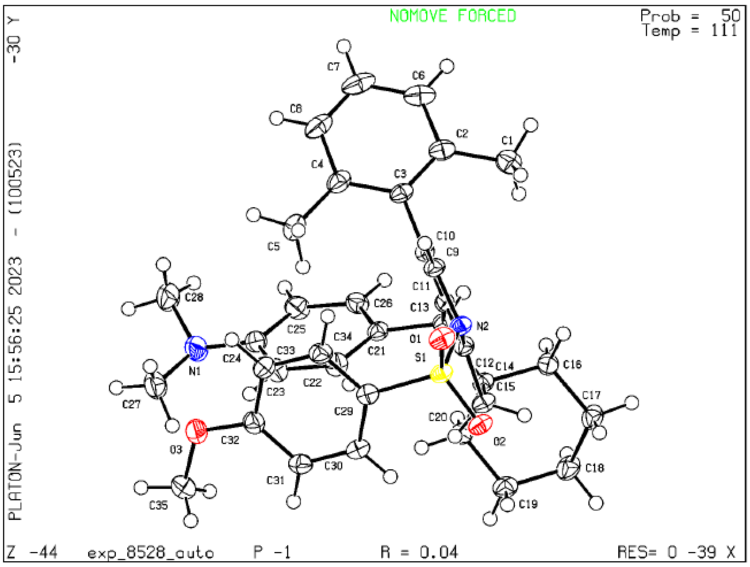

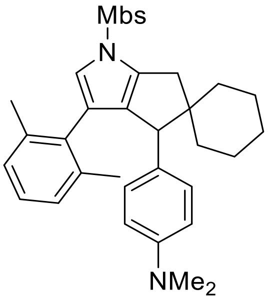

Bond precision: C-C = 0.0018 A Wavelength=1.54184

Cell:	a=10.9798(2)	b=11.0505(2)	c=12.7508(1)
	alpha=86.375(1)	beta=83.639(1)	gamma=82.282(2)
Temperature:	111 K		

	Calculated	Reported
Volume	1521.79(4)	1521.79(4)
Space group	P -1	P -1
Hall group	-P 1	-P 1
Moiety formula	C35 H40 N2 O3 S	C35 H40 N2 O3 S
Sum formula	C35 H40 N2 O3 S	C35 H40 N2 O3 S
Mr	568.75	568.75
Dx,g cm-3	1.241	1.241
Z	2	2
Mu (mm-1)	1.235	1.235
F000	608.0	608.0
F000'	610.27	
h,k,lmax	13,13,15	13,13,15
Nref	6275	5965
Tmin,Tmax	0.976,0.976	0.886,1.000
Tmin'	0.976	

Correction method= # Reported T Limits: Tmin=0.886 Tmax=1.000
AbsCorr = MULTI-SCAN

Data completeness= 0.951 Theta(max)= 75.148

R(reflections)= 0.0352(5575) wR2(reflections)= 0.0951(5965)

S = 1.066 Npar= 375

Supplementary Figure 9. Crystal data and structure refinement for (±)-**2b**.

CCDC Number = 2270341.

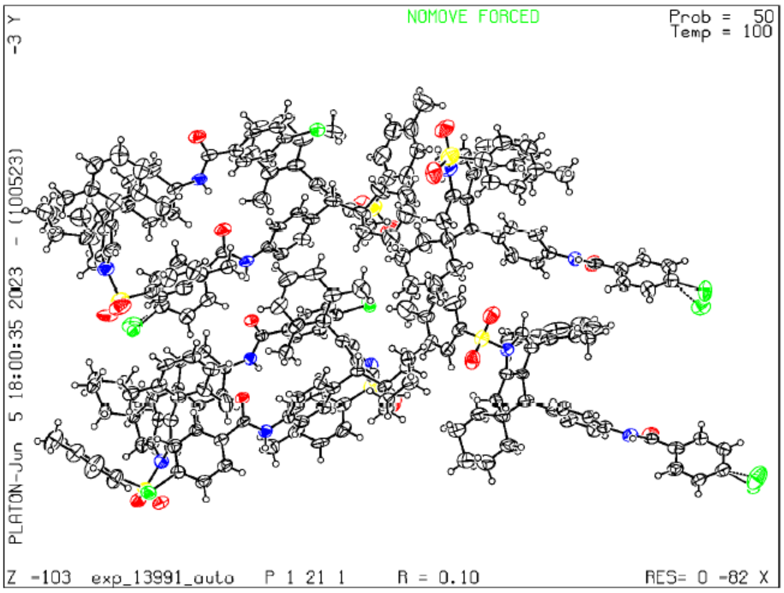

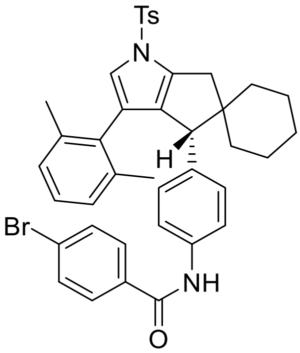

Bond precision: C-C = 0.0136 A Wavelength=1.54184

Cell: a=18.2120(2) b=10.4169(1) c=54.5817(5)
alpha=90 beta=91.514(1) gamma=90

Temperature: 100 K

	Calculated	Reported
Volume	10351.22(18)	10351.22(18)
Space group	P 21	P 1 21 1
Hall group	P 2yb	P 2yb
Moiety formula	C40 H39 Br N2 O3 S	C40 H39 Br N2 O3 S
Sum formula	C40 H39 Br N2 O3 S	C40 H39 Br N2 O3 S
Mr	707.69	707.70
Dx,g cm-3	1.362	1.362
Z	12	12
Mu (mm-1)	2.510	2.510
F000	4416.0	4416.0
F000'	4422.53	
h,k,lmax	21,12,65	21,12,65
Nref	36976[19604]	36953
Tmin,Tmax	0.817,0.778	0.725,1.000
Tmin'	0.741	

Correction method= # Reported T Limits: Tmin=0.725 Tmax=1.000
AbsCorr = MULTI-SCAN

Data completeness= 1.88/1.00 Theta(max)= 67.079

R(reflections)= 0.0955(33929) wR2(reflections)= 0.2990(36953)

S = 1.123 Npar= 2309

Supplementary Figure 10. Crystal data and structure refinement for (-)-**3fb**.

CCDC Number =2270342.

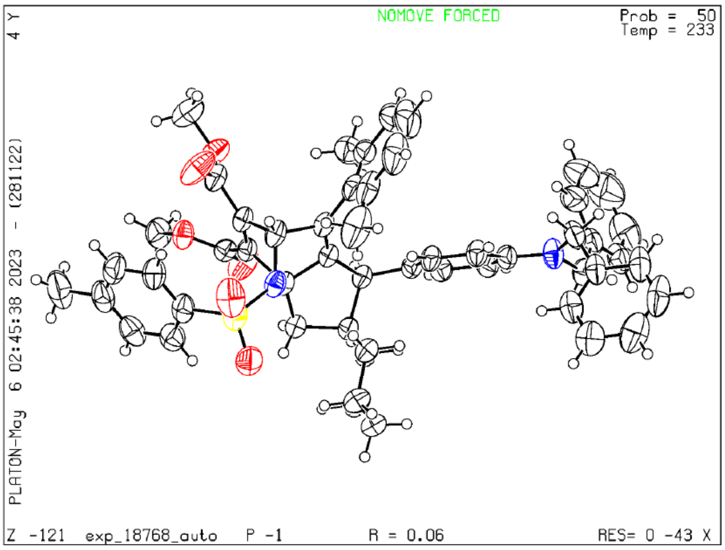

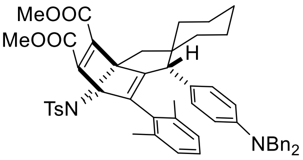

Bond precision:	C-C = 0.0050 A	Wavelength=1.54184

Cell:	a=14.0153(3)	b=14.7720(3)	c=14.8716(3)
	alpha=104.309(2)	beta=104.247(2)	gamma=112.795(2)
Temperature:	233 K		

	Calculated	Reported
Volume	2541.03(12)	2541.02(10)
Space group	P -1	P -1
Hall group	-P 1	-P 1
Moiety formula	C53 H54 N2 O6 S [+ solvent]	C53 H54 N2 O6 S
Sum formula	C53 H54 N2 O6 S [+ solvent]	C53 H54 N2 O6 S
Mr	847.04	847.04
Dx,g cm-3	1.107	1.107
Z	2	2
Mu (mm-1)	0.940	0.940
F000	900.0	900.0
F000'	903.18	
h,k,lmax	17,18,18	17,18,18
Nref	10667	10427
Tmin,Tmax		0.819,1.000
Tmin'		

Correction method= # Reported T Limits: Tmin=0.819 Tmax=1.000
AbsCorr = MULTI-SCAN

Data completeness= 0.978 Theta(max)= 76.394

R(reflections)= 0.0627(8056) wR2(reflections)= 0.1844(10427)

S = 1.067 Npar= 564

Supplementary Figure 11. Crystal data and structure refinement for (±)-**3fc**.

CCDC Number = 2270343.

7. Computational Studies

All calculations were performed using **Gaussian16** package[6]. Geometry optimizations and vibration frequencies were calculated by using **B3LYP-D3** level of theory[7,8] with the **LANL2DZ** basis set[9,10] and pseudopotential for the Cu atom, and the **6-31G(d,p)** basis set[11] for C, H, O, N and S atom. All the electronic energies were recomputed at **PBE0-D3**[12]**/Def2-TZVP**[13] level of theory. All local minimums were confirmed with no imaginary frequency and all transition states had only one imaginary frequency. And every transition state was checked by intrinsic reaction coordinate (IRC). The SMD solvation mode[14] with ^{m}xylene was used for all calculations. The ball stick models of molecules were drawn by VMD[15].

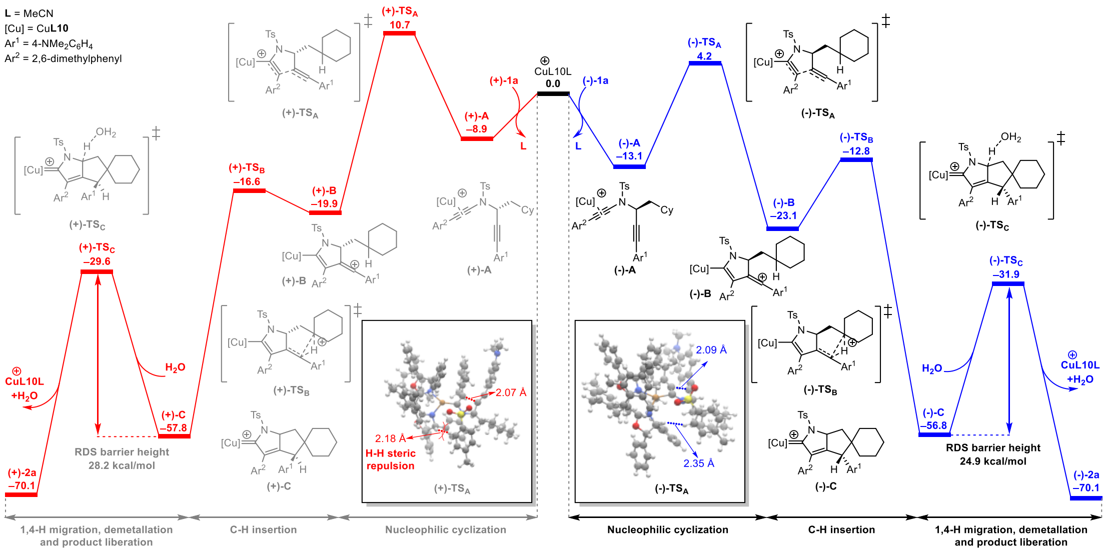

Supplementary Figure 12. Plausible reaction mechanism. Relative free energies (ΔG, in kcal/mol) of all the transition states and intermediates were computed at the SMD(solvent = ^{m}xylene)-PBE0-D3/Def2-TZVP//SMD(solvent = ^{m}xylene)-B3LYP-D3/6-31G(d,p) level of theory

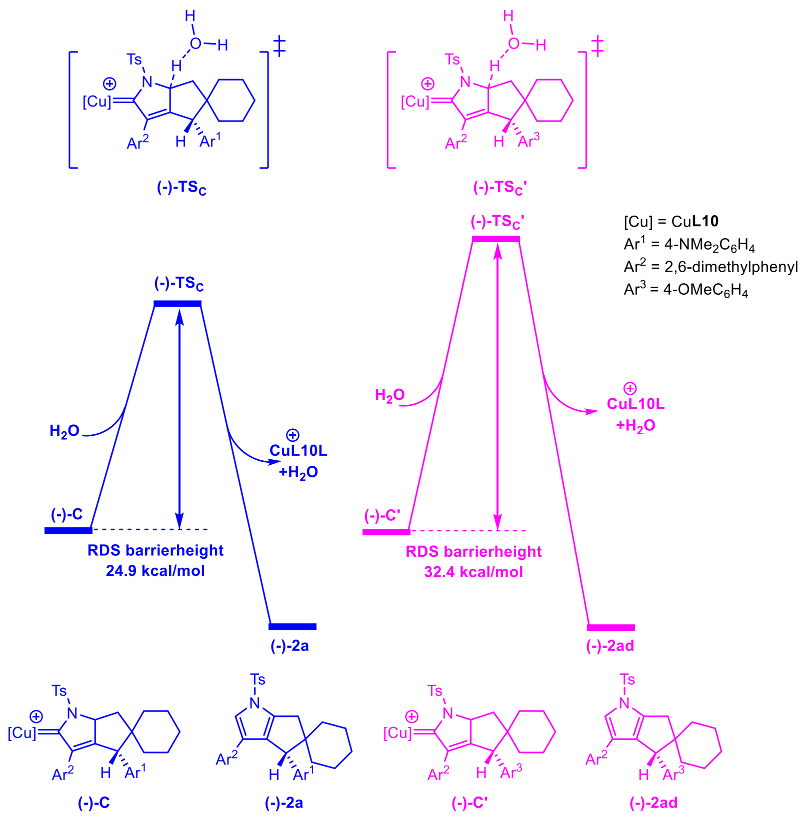

Supplementary Figure 13. Barrier-heights of the rate-determining step affording (-)-**2a** and (-)-**2ad**. Relative free energies (ΔG, in kcal/mol) of all the transition states and intermediates were computed at the SMD(solvent = ^{m}xylene)-PBE0-D3/Def2-TZVP//SMD(solvent = ^{m}xylene)-B3LYP-D3/6-31G(d,p) level of theory.

8. HPLC Chromatograms

(+)-1a: IE, *i*-PrOH/hexane = 5/95, v = 1.0 mL/min, λ = 254 nm

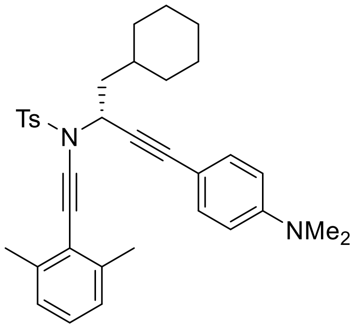

Chromatogram

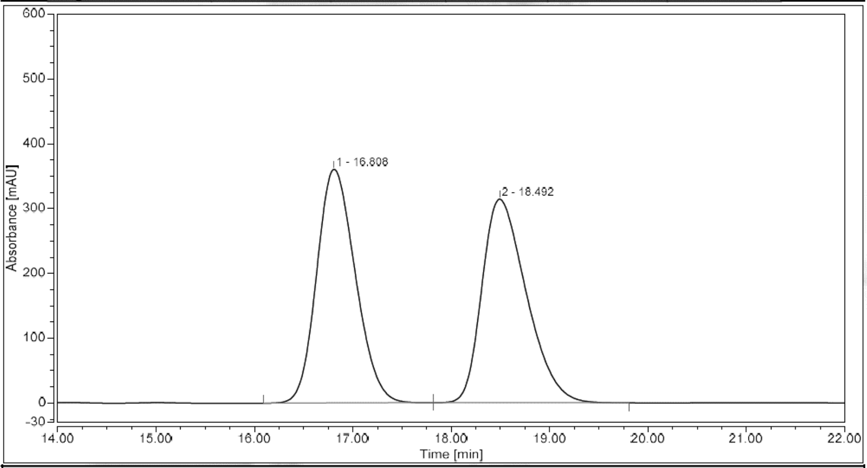

Integration Results

No.	Peak Name	Retention Time min	Area mAU*min	Height mAU	Relative Area %	Relative Height %	Amount n.a.
1		16.808	164.035	360.612	49.97	53.45	n.a.
2		18.492	164.247	314.045	50.03	46.55	n.a.
**Total:**			**328.282**	**674.657**	**100.00**	**100.00**	

Chromatogram

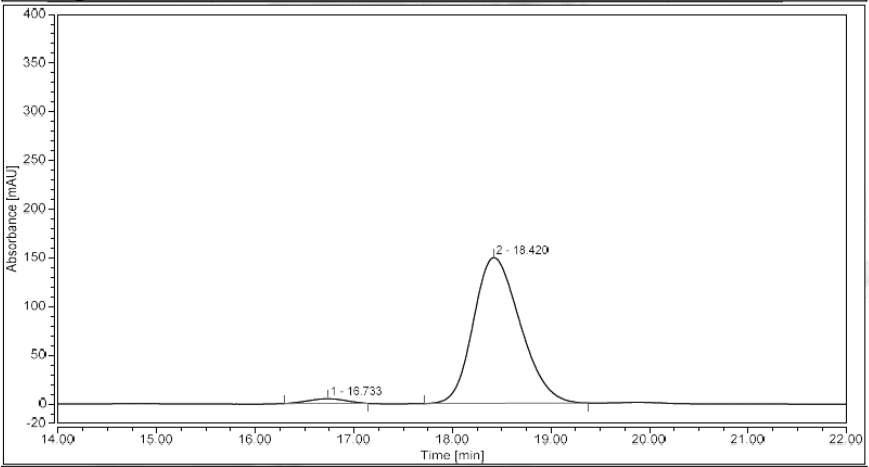

Integration Results

No.	Peak Name	Retention Time min	Area mAU*min	Height mAU	Relative Area %	Relative Height %	Amount n.a.
1		16.733	2.092	4.783	2.48	3.10	n.a.
2		18.420	82.248	149.589	97.52	96.90	n.a.
**Total:**			**84.339**	**154.372**	**100.00**	**100.00**	

Supplementary Figure 14. HPLC spectrum for compound **(+)-1a**

(-)-2a: IE, *i*-PrOH/hexane = 5/95, v = 1.0 mL/min, λ = 254 nm

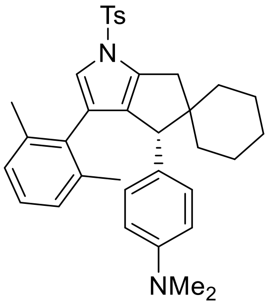

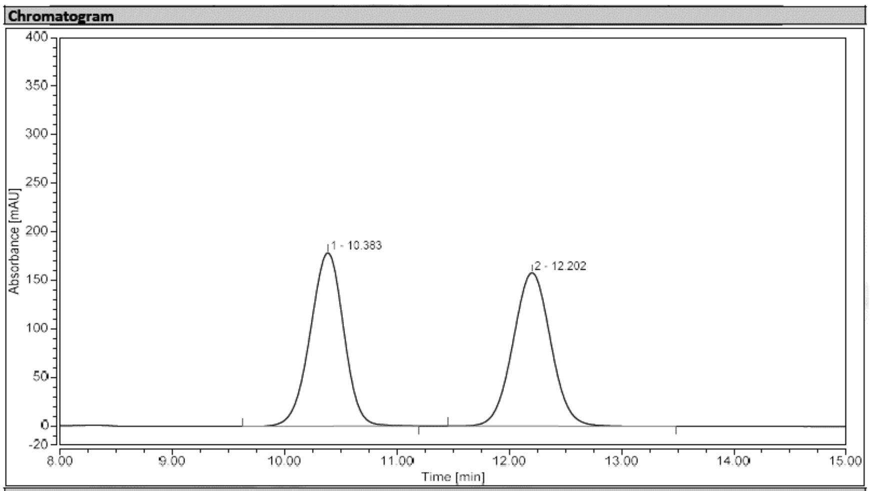

Integration Results

No.	Peak Name	Retention Time min	Area mAU*min	Height mAU	Relative Area %	Relative Height %	Amount n.a.
1		10.383	61.722	178.498	50.13	53.07	n.a.
2		12.202	61.410	157.843	49.87	46.93	n.a.
Total:			123.133	336.341	100.00	100.00	

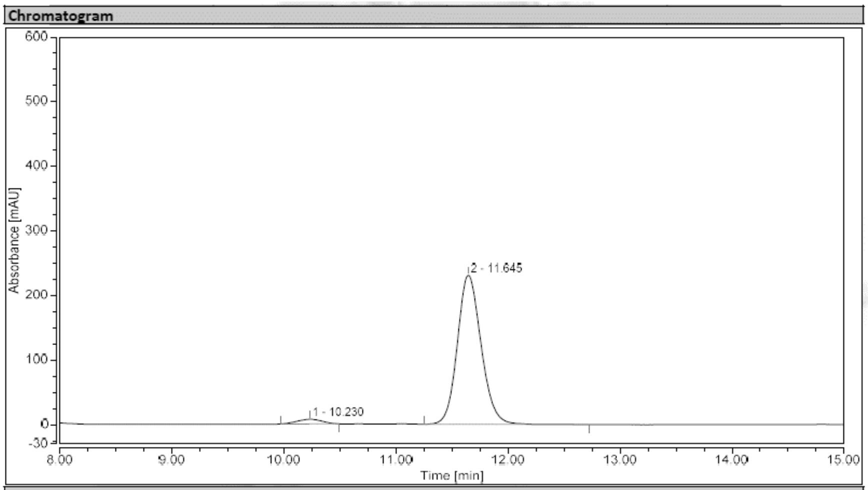

Integration Results

No.	Peak Name	Retention Time min	Area mAU*min	Height mAU	Relative Area %	Relative Height %	Amount n.a.
1		10.230	1.832	7.160	3.13	3.01	n.a.
2		11.645	56.797	230.858	96.87	96.99	n.a.
Total:			58.629	238.018	100.00	100.00	

Supplementary Figure 15. HPLC spectrum for compound **(-)-2a**

(+)-1b: IE, *i*-PrOH/hexane = 20/80, v = 1.0 mL/min, λ = 254 nm

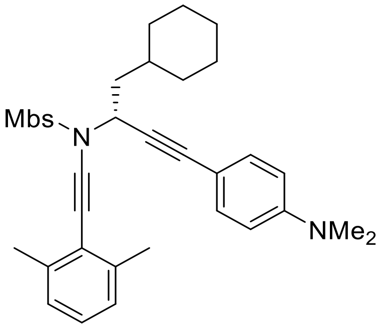

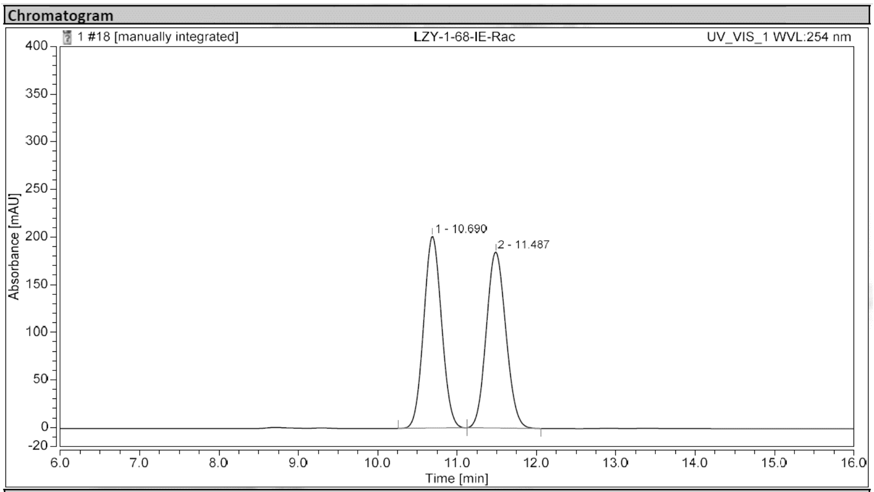

Integration Results

No.	Peak Name	Retention Time min	Area mAU*min	Height mAU	Relative Area %	Relative Height %	Amount n.a.
1		10.690	52.776	201.365	50.02	52.14	n.a.
2		11.487	52.731	184.812	49.98	47.86	n.a.
Total:			105.507	386.177	100.00	100.00	

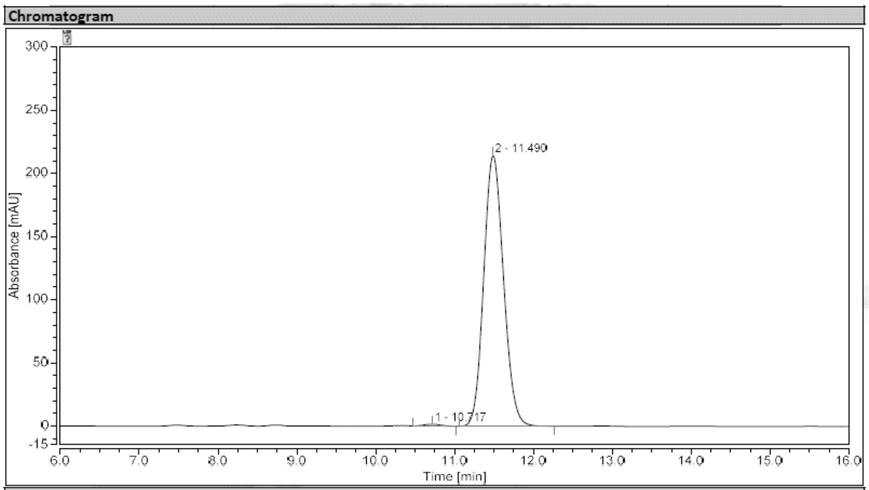

Integration Results

No.	Peak Name	Retention Time min	Area mAU*min	Height mAU	Relative Area %	Relative Height %	Amount n.a.
1		10.717	0.337	1.423	0.54	0.66	n.a.
2		11.490	62.206	214.446	99.46	99.34	n.a.
Total:			62.543	215.870	100.00	100.00	

Supplementary Figure 16. HPLC spectrum for compound **(+)-1b**

(-)-2b: IE, *i*-PrOH/hexane = 20/80, v = 1.0 mL/min, λ = 254 nm

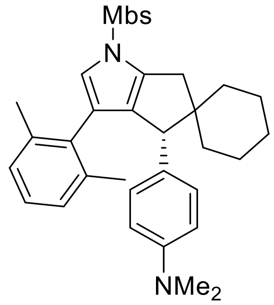

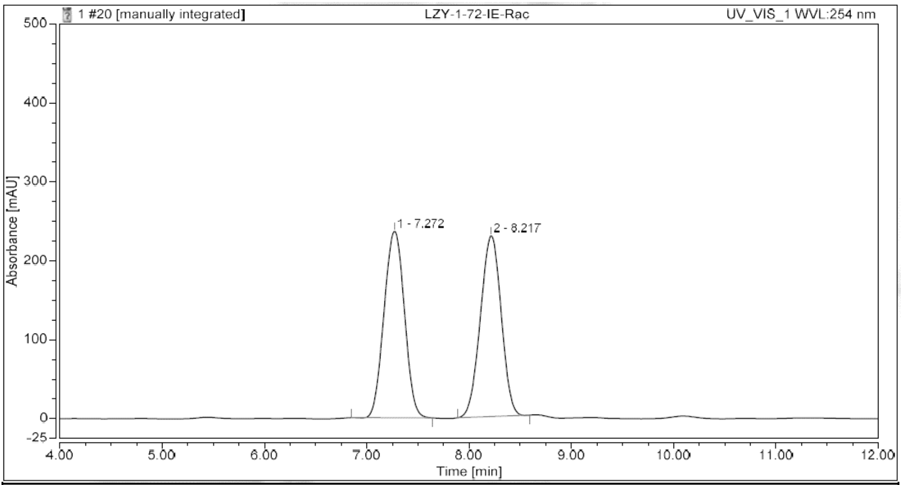

Integration Results

No.	Peak Name	Retention Time min	Area mAU*min	Height mAU	Relative Area %	Relative Height %	Amount n.a.
1		7.272	54.539	236.289	50.27	50.77	n.a.
2		8.217	53.963	229.118	49.73	49.23	n.a.
Total:			108.502	465.407	100.00	100.00	

Chromatogram

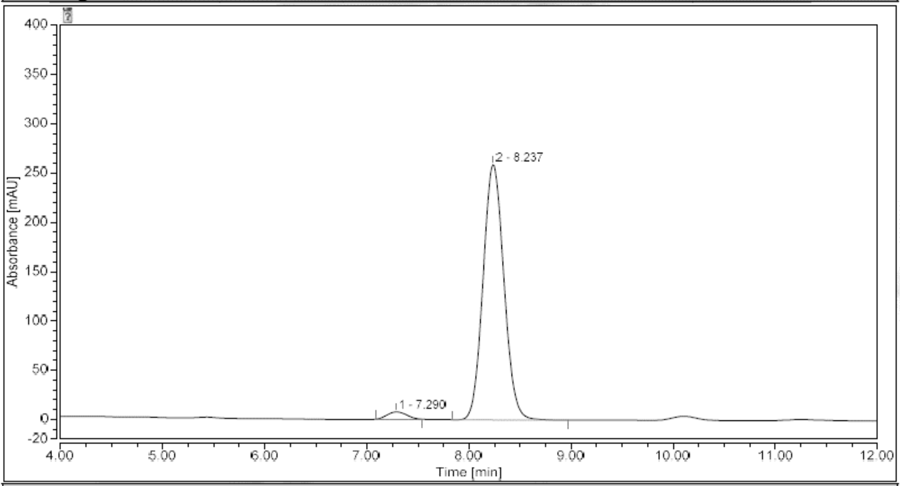

Integration Results

No.	Peak Name	Retention Time min	Area mAU*min	Height mAU	Relative Area %	Relative Height %	Amount n.a.
1		7.290	1.600	7.365	2.54	2.76	n.a.
2		8.237	61.383	259.033	97.46	97.24	n.a.
Total:			62.984	266.398	100.00	100.00	

Supplementary Figure 17. HPLC spectrum for compound **(-)-2b**

(+)-1c: IE, *i*-PrOH/hexane = 3/97, v = 1.0 mL/min, λ = 254 nm

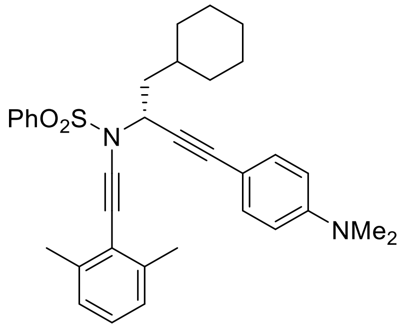

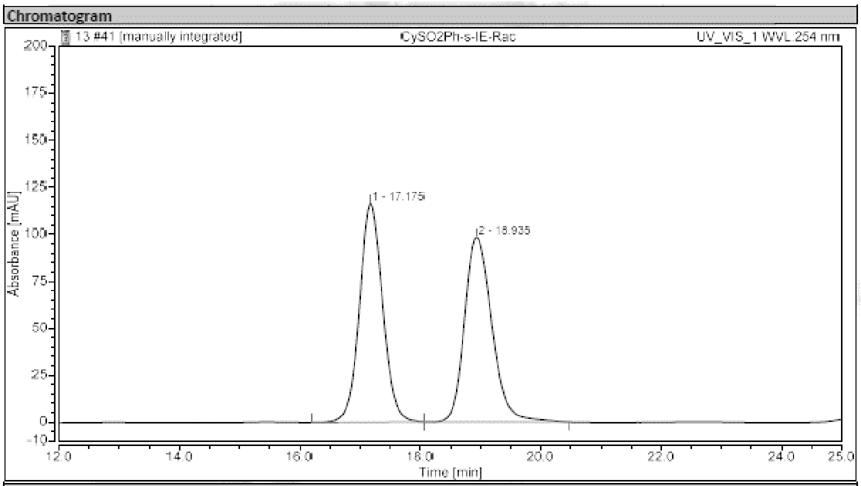

Integration Results

No.	Peak Name	Retention Time min	Area mAU*min	Height mAU	Relative Area %	Relative Height %	Amount n.a.
1		17.175	51.734	116.200	50.06	54.19	n.a.
2		18.935	51.619	98.234	49.94	45.81	n.a.
**Total:**			**103.353**	**214.433**	**100.00**	**100.00**	

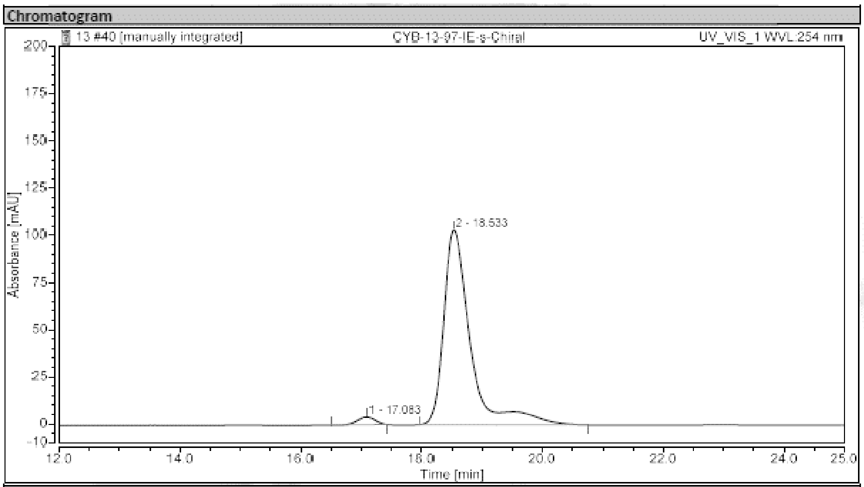

Integration Results

No.	Peak Name	Retention Time min	Area mAU*min	Height mAU	Relative Area %	Relative Height %	Amount n.a.
1		17.083	1.392	4.056	2.57	3.78	n.a.
2		18.533	52.678	103.278	97.43	96.22	n.a.
**Total:**			**54.070**	**107.333**	**100.00**	**100.00**	

Supplementary Figure 18. HPLC spectrum for compound **(+)-1c**

(-)-2c: IE, *i*-PrOH/hexane = 5/95, v = 1.0 mL/min, λ = 254 nm

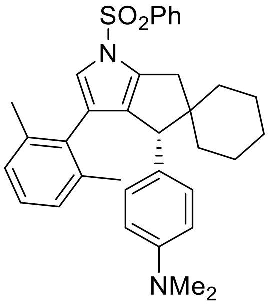

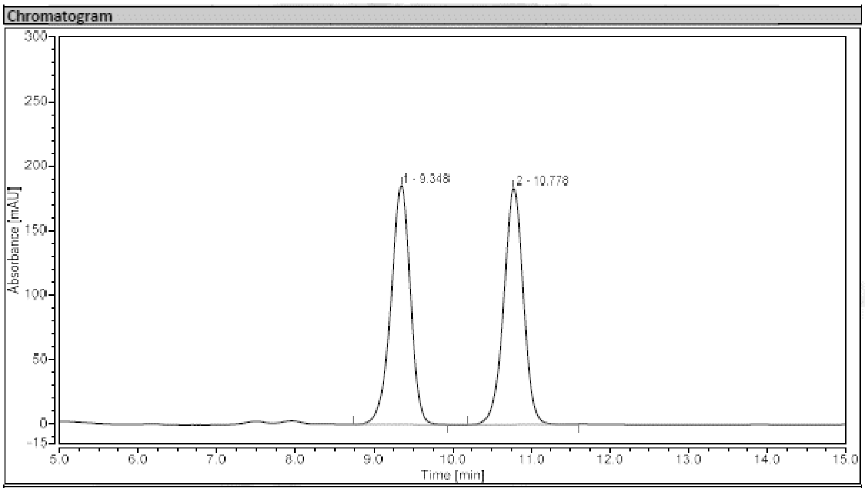

Integration Results

No.	Peak Name	Retention Time min	Area mAU*min	Height mAU	Relative Area %	Relative Height %	Amount n.a.
1		9.348	52.809	185.257	50.04	50.30	n.a.
2		10.778	52.734	183.050	49.96	49.70	n.a.
Total:			105.543	368.307	100.00	100.00	

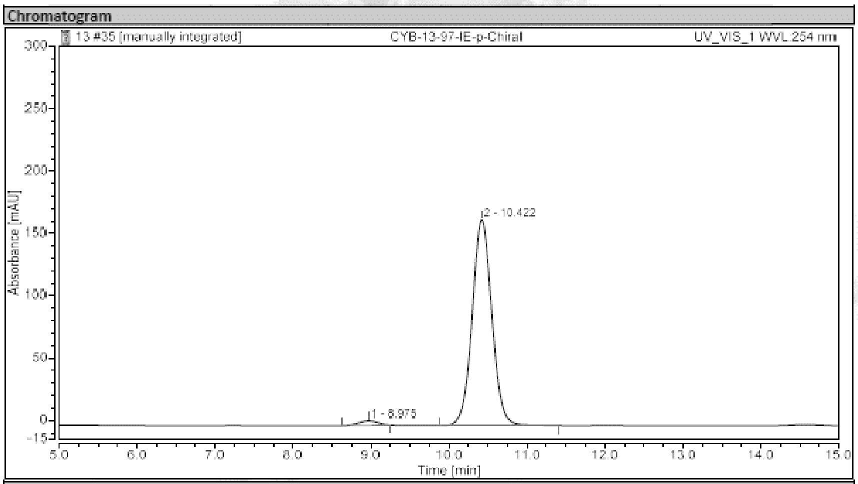

Integration Results

No.	Peak Name	Retention Time min	Area mAU*min	Height mAU	Relative Area %	Relative Height %	Amount n.a.
1		8.975	1.000	3.543	2.03	2.10	n.a.
2		10.422	48.250	165.218	97.97	97.90	n.a.
Total:			49.250	168.760	100.00	100.00	

Supplementary Figure 19. HPLC spectrum for compound **(-)-2c**

(+)-1d: IE, *i*-PrOH/hexane = 5/95, v = 1.0 mL/min, λ = 254 nm

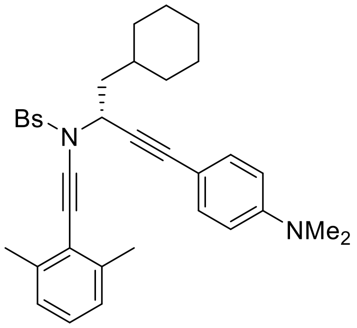

Chromatogram

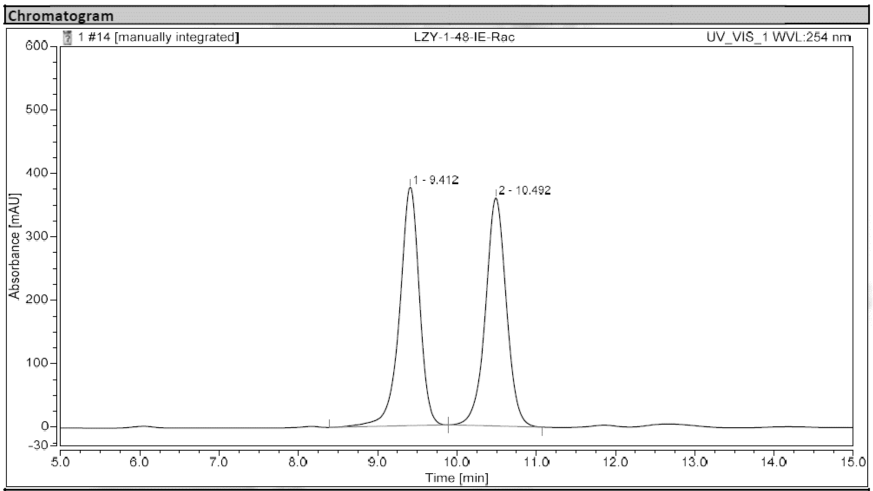

Integration Results

No.	Peak Name	Retention Time min	Area mAU*min	Height mAU	Relative Area %	Relative Height %	Amount n.a.
1		9.412	108.548	375.989	49.78	51.10	n.a.
2		10.492	109.495	359.772	50.22	48.90	n.a.
**Total:**			**218.043**	**735.762**	**100.00**	**100.00**	

Chromatogram

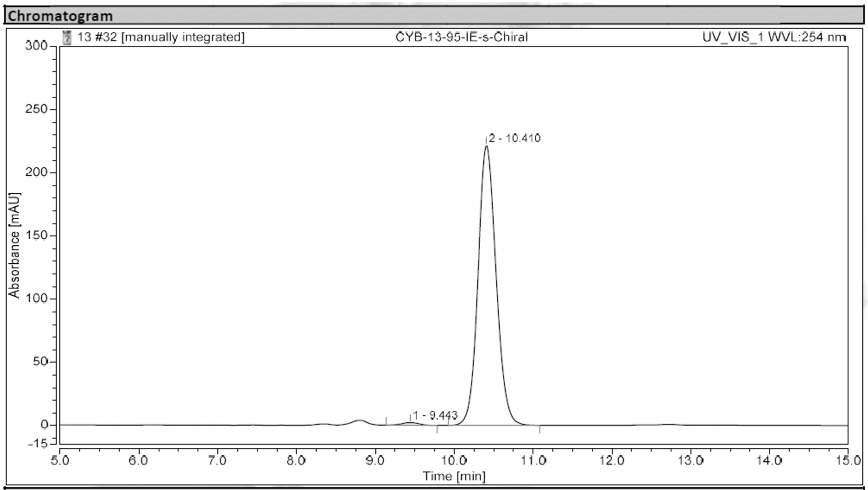

Integration Results

No.	Peak Name	Retention Time min	Area mAU*min	Height mAU	Relative Area %	Relative Height %	Amount n.a.
1		9.443	0.535	2.129	0.90	0.95	n.a.
2		10.410	59.000	221.542	99.10	99.05	n.a.
**Total:**			**59.535**	**223.671**	**100.00**	**100.00**	

Supplementary Figure 20. HPLC spectrum for compound **(+)-1d**

(-)-2d: IE, *i*-PrOH/hexane = 5/95, v = 1.0 mL/min, λ = 254 nm

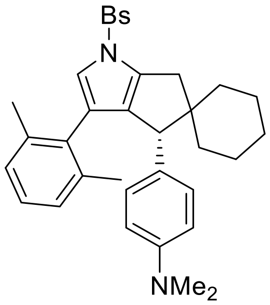

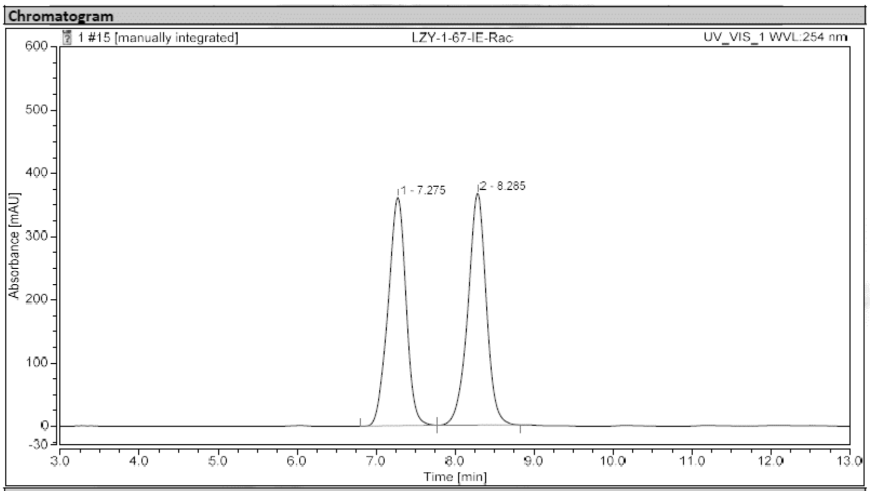

Integration Results

No.	Peak Name	Retention Time min	Area mAU*min	Height mAU	Relative Area %	Relative Height %	Amount n.a.
1		7.275	94.869	361.295	48.87	49.62	n.a.
2		8.285	99.260	366.876	51.13	50.38	n.a.
Total:			194.128	728.171	100.00	100.00	

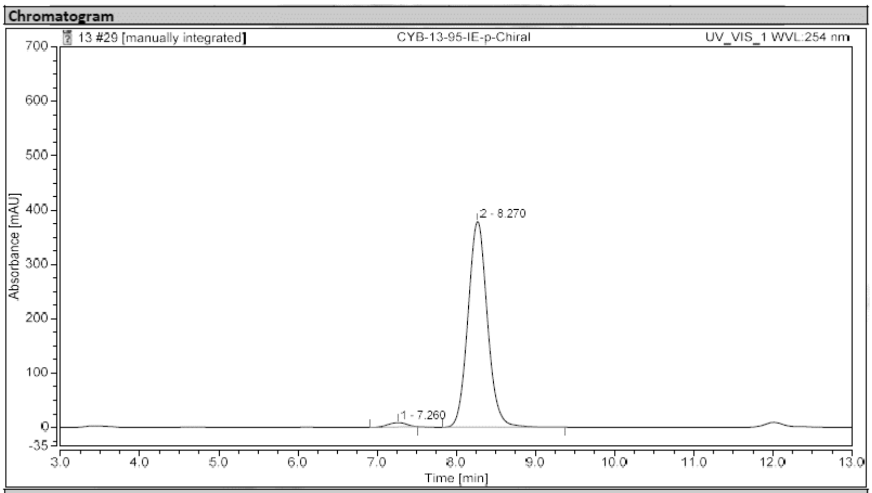

Integration Results

No.	Peak Name	Retention Time min	Area mAU*min	Height mAU	Relative Area %	Relative Height %	Amount n.a.
1		7.260	2.116	7.873	1.92	2.03	n.a.
2		8.270	107.985	379.134	98.08	97.97	n.a.
Total:			110.101	387.007	100.00	100.00	

Supplementary Figure 21. HPLC spectrum for compound **(-)-2d**

(+)-1e: IE, *i*-PrOH/hexane = 5/95, v = 1.0 mL/min, λ = 254 nm

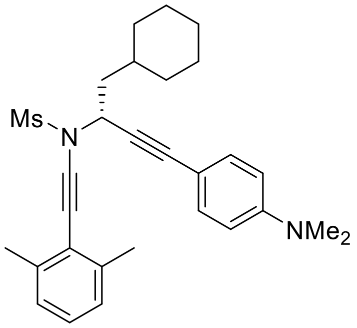

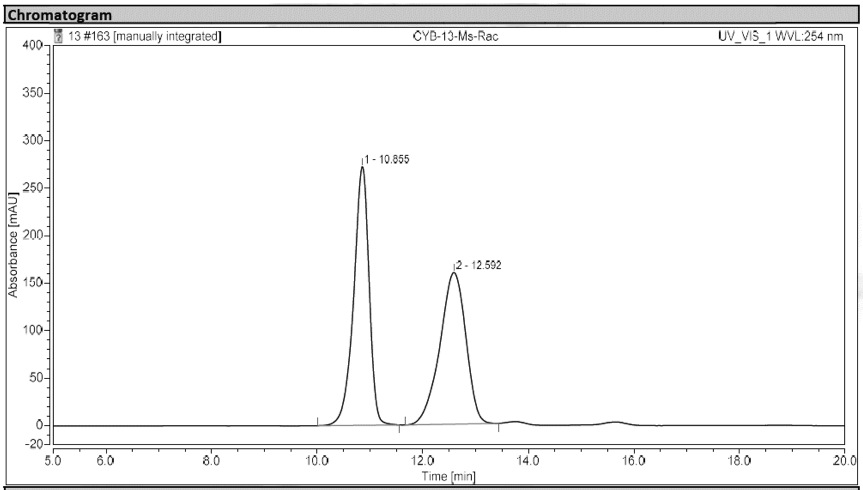

Integration Results

No.	Peak Name	Retention Time min	Area mAU*min	Height mAU	Relative Area %	Relative Height %	Amount n.a.
1		10.855	92.395	272.624	50.39	63.07	n.a.
2		12.592	90.971	159.632	49.61	36.93	n.a.
Total:			183.365	432.256	100.00	100.00	

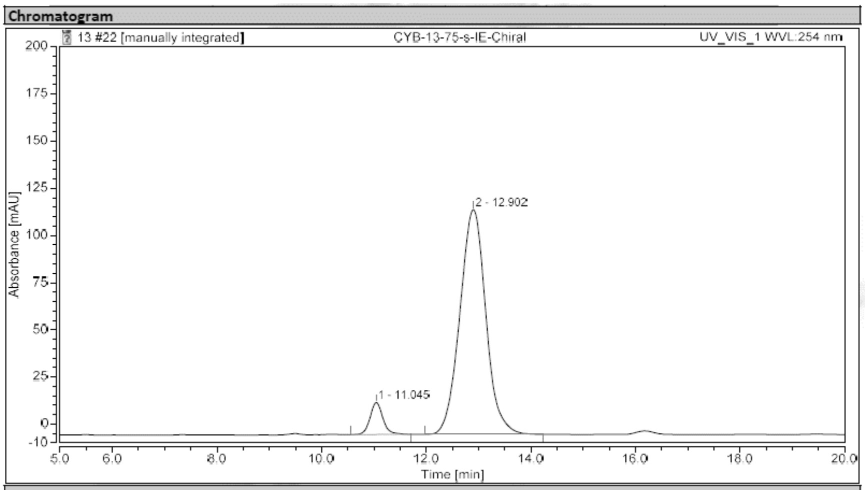

Integration Results

No.	Peak Name	Retention Time min	Area mAU*min	Height mAU	Relative Area %	Relative Height %	Amount n.a.
1		11.045	4.804	16.985	6.56	12.47	n.a.
2		12.902	68.415	119.168	93.44	87.53	n.a.
Total:			73.219	136.153	100.00	100.00	

Supplementary Figure 22. HPLC spectrum for compound **(+)-1e**

(-)-2e: ODH, *i*-PrOH/hexane = 5/95, v = 1.0 mL/min, λ = 254 nm

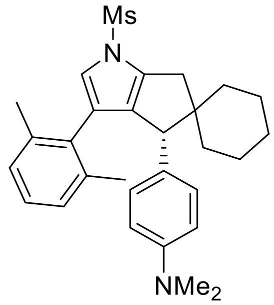

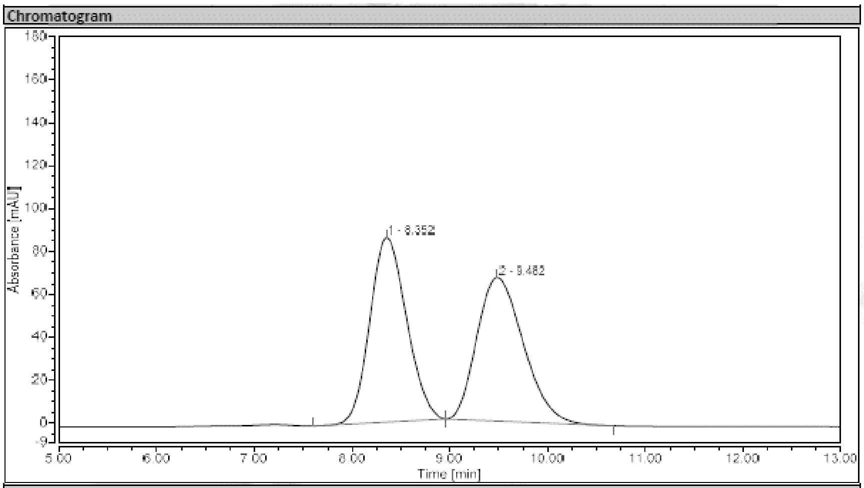

Integration Results

No.	Peak Name	Retention Time min	Area mAU*min	Height mAU	Relative Area %	Relative Height %	Amount n.a.
1		8.352	37.431	86.011	50.40	56.26	n.a.
2		9.482	36.835	66.865	49.60	43.74	n.a.
Total:			74.266	152.876	100.00	100.00	

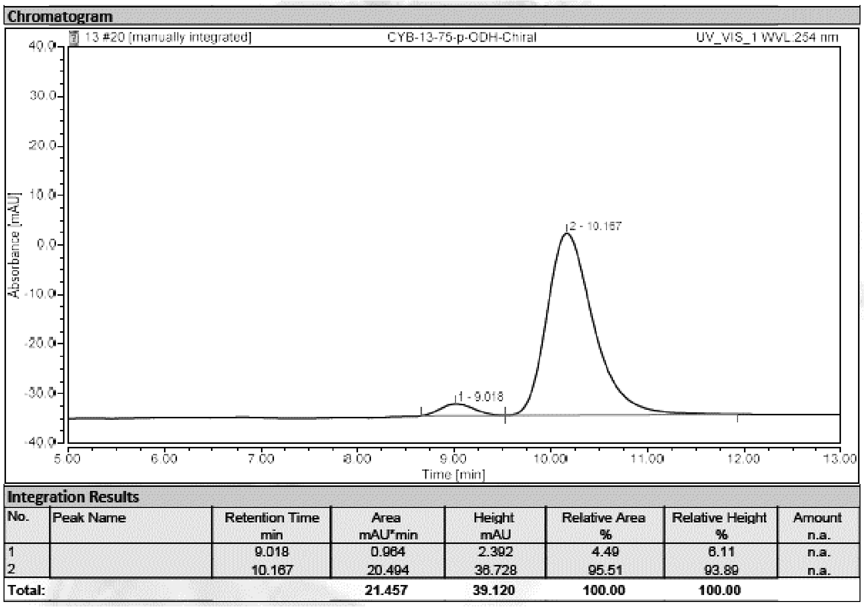

Integration Results

No.	Peak Name	Retention Time min	Area mAU*min	Height mAU	Relative Area %	Relative Height %	Amount n.a.
1		9.018	0.964	2.392	4.49	6.11	n.a.
2		10.167	20.494	36.728	95.51	93.89	n.a.
Total:			21.457	39.120	100.00	100.00	

Supplementary Figure 23. HPLC spectrum for compound **(-)-2e**

(+)-1f: IE, *i*-PrOH/hexane = 30/70, v = 1.0 mL/min, λ = 254 nm

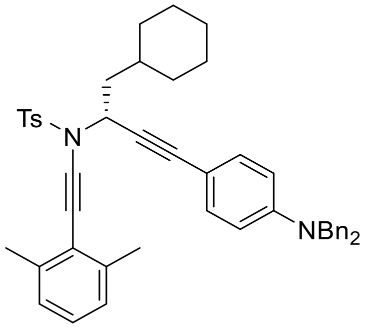

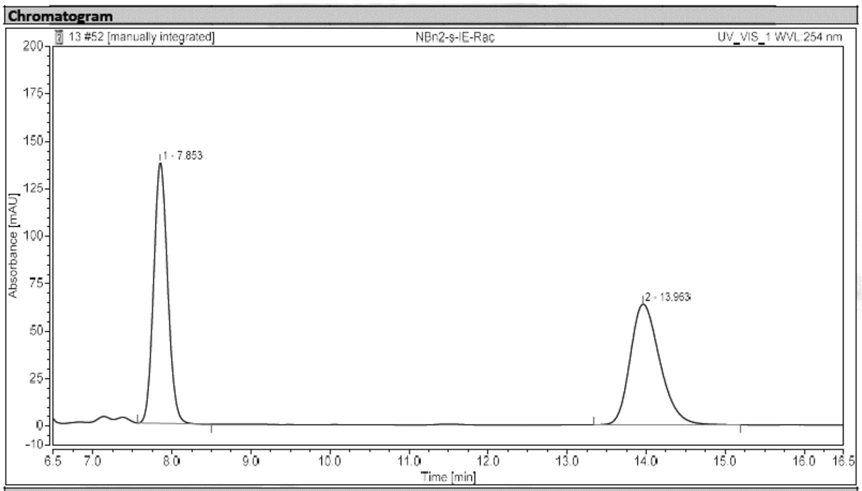

Integration Results

No.	Peak Name	Retention Time min	Area mAU*min	Height mAU	Relative Area %	Relative Height %	Amount n.a.
1		7.853	28.158	137.506	50.53	68.39	n.a.
2		13.963	27.565	63.546	49.47	31.61	n.a.
**Total:**			**55.723**	**201.052**	**100.00**	**100.00**	

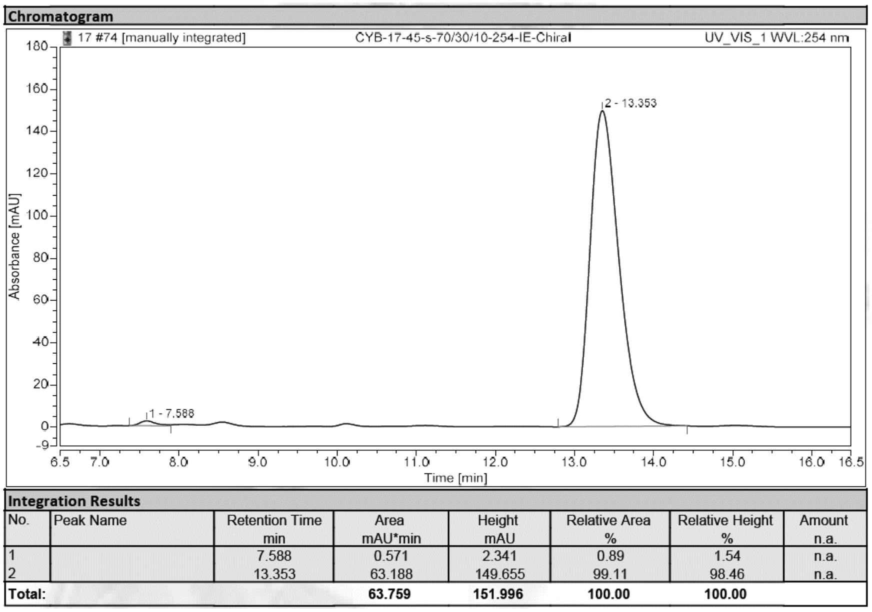

Integration Results

No.	Peak Name	Retention Time min	Area mAU*min	Height mAU	Relative Area %	Relative Height %	Amount n.a.
1		7.588	0.571	2.341	0.89	1.54	n.a.
2		13.353	63.188	149.655	99.11	98.46	n.a.
**Total:**			**63.759**	**151.996**	**100.00**	**100.00**	

Supplementary Figure 24. HPLC spectrum for compound **(+)-1f**

(-)-2f: ODH, *i*-PrOH/hexane = 3/97, v = 1.0 mL/min, λ = 254 nm

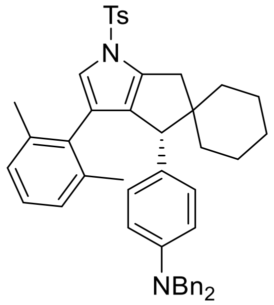

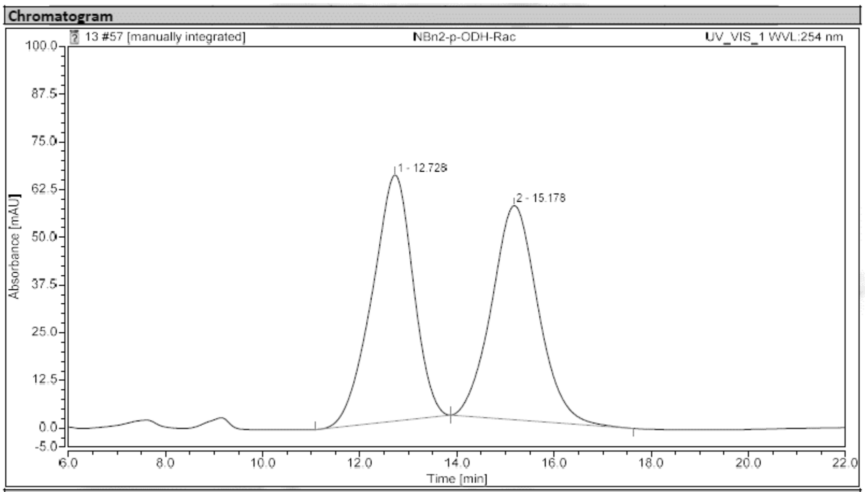

Integration Results

No.	Peak Name	Retention Time min	Area mAU*min	Height mAU	Relative Area %	Relative Height %	Amount n.a.
1		12.728	63.026	64.499	50.33	53.46	n.a.
2		15.178	62.210	56.156	49.67	46.54	n.a.
**Total:**			**125.236**	**120.654**	**100.00**	**100.00**	

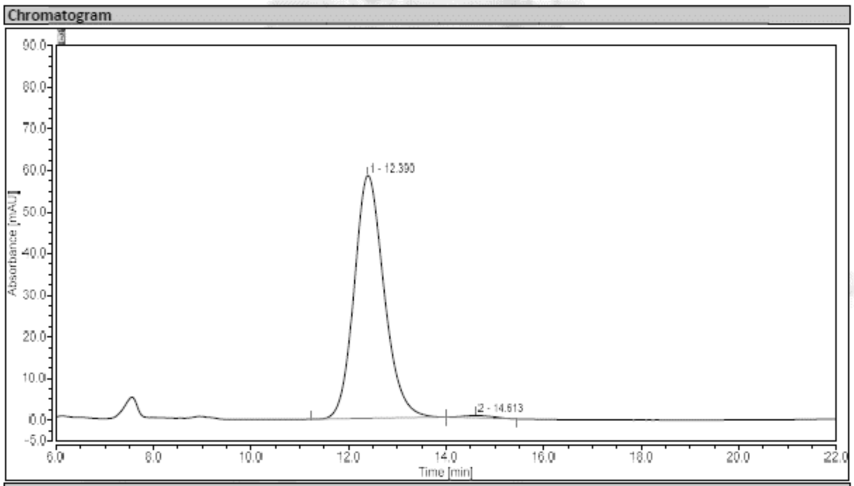

Integration Results

No.	Peak Name	Retention Time min	Area mAU*min	Height mAU	Relative Area %	Relative Height %	Amount n.a.
1		12.390	41.505	58.430	99.03	99.04	n.a.
2		14.613	0.407	0.563	0.97	0.96	n.a.
**Total:**			**41.912**	**58.993**	**100.00**	**100.00**	

Supplementary Figure 25. HPLC spectrum for compound **(-)-2f**

(+)-1g: ADH, *i*-PrOH/hexane = 5/95, v = 1.0 mL/min, λ = 254 nm

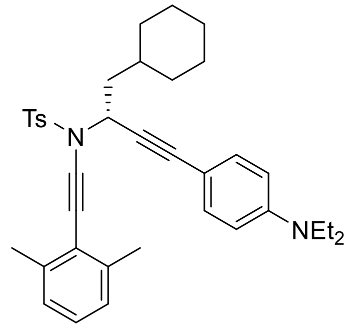

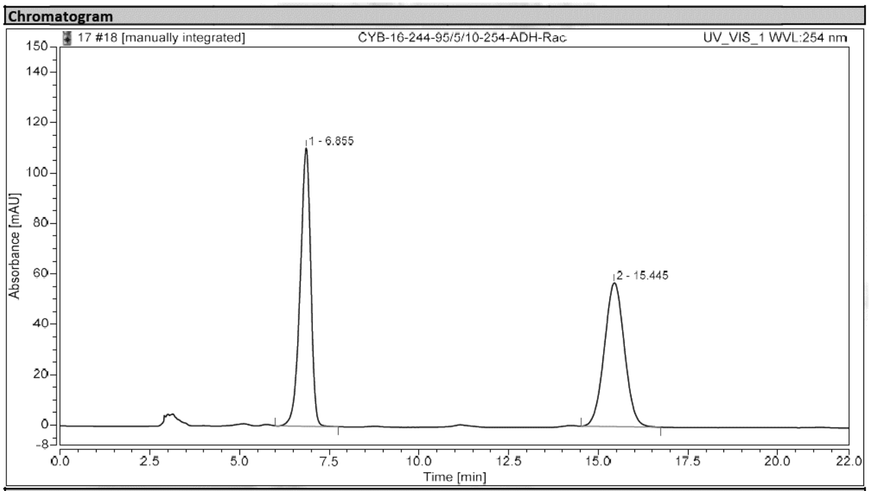

Integration Results

No.	Peak Name	Retention Time min	Area mAU*min	Height mAU	Relative Area %	Relative Height %	Amount n.a.
1		6.855	37.578	110.403	50.41	65.93	n.a.
2		15.445	36.970	57.042	49.59	34.07	n.a.
**Total:**			**74.547**	**167.445**	**100.00**	**100.00**	

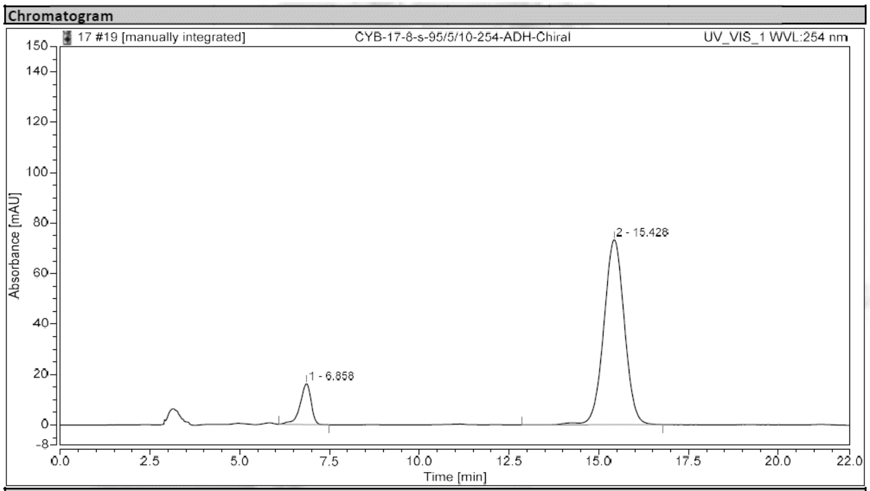

Integration Results

No.	Peak Name	Retention Time min	Area mAU*min	Height mAU	Relative Area %	Relative Height %	Amount n.a.
1		6.858	5.766	16.236	10.58	18.12	n.a.
2		15.428	48.717	73.350	89.42	81.88	n.a.
**Total:**			**54.483**	**89.586**	**100.00**	**100.00**	

Supplementary Figure 26. HPLC spectrum for compound **(+)-1g**

(-)-2g: IE, *i*-PrOH/hexane = 2/98, v = 1.0 mL/min, λ = 254 nm

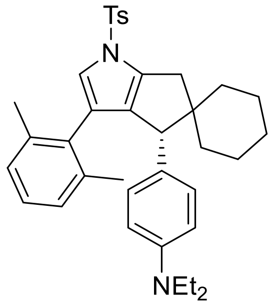

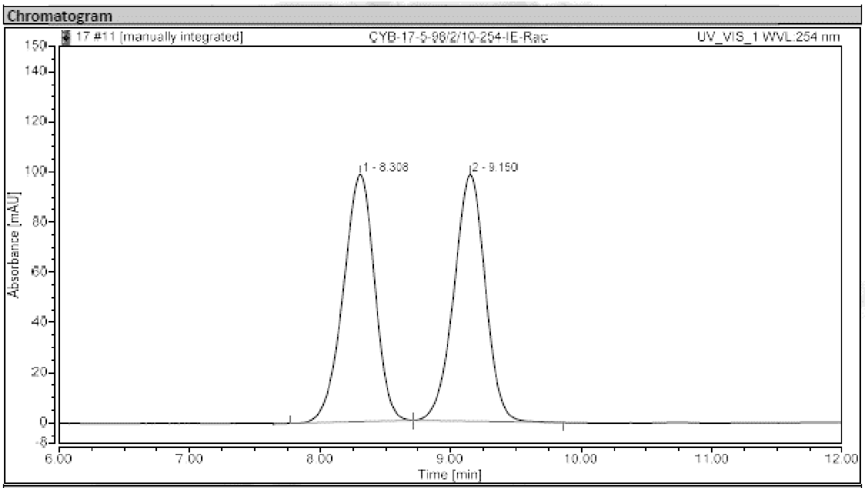

Integration Results

No.	Peak Name	Retention Time min	Area mAU*min	Height mAU	Relative Area %	Relative Height %	Amount n.a.
1		8.308	27.444	98.512	50.04	50.08	n.a.
2		9.150	27.395	98.196	49.96	49.92	n.a.
Total:			54.839	196.709	100.00	100.00	

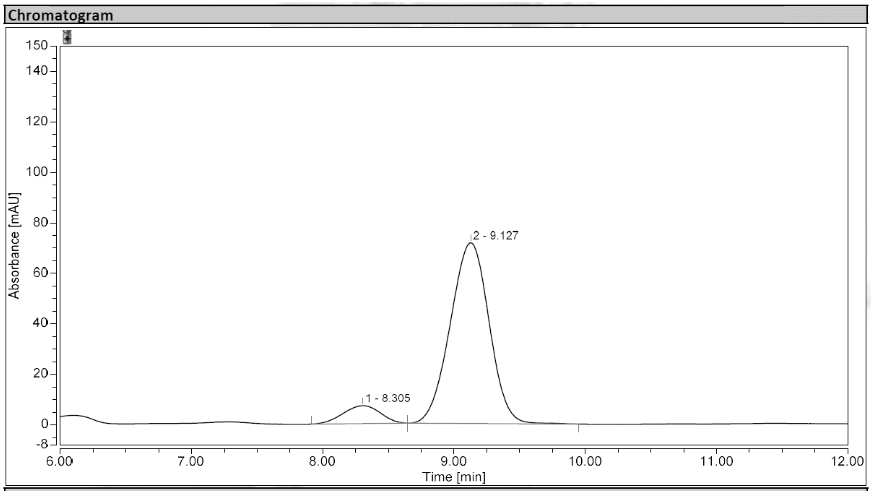

Integration Results

No.	Peak Name	Retention Time min	Area mAU*min	Height mAU	Relative Area %	Relative Height %	Amount n.a.
1		8.305	2.331	7.090	8.68	9.02	n.a.
2		9.127	24.526	71.541	91.32	90.98	n.a.
Total:			26.857	78.631	100.00	100.00	

Supplementary Figure 27. HPLC spectrum for compound **(-)-2g**

(+)-1h: ADH, *i*-PrOH/hexane = 5/95, v = 1.0 mL/min, λ = 254 nm

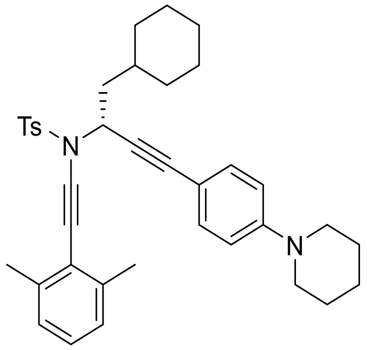

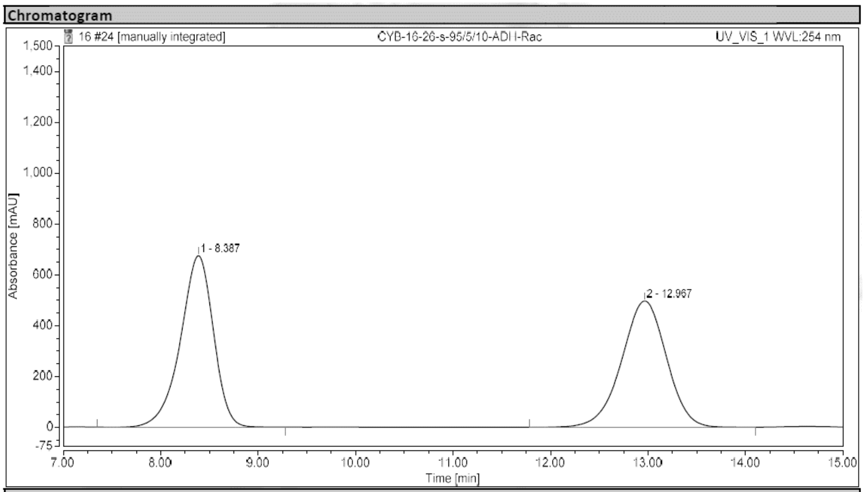

Integration Results

No.	Peak Name	Retention Time min	Area mAU*min	Height mAU	Relative Area %	Relative Height %	Amount n.a.
1		8.387	264.687	675.029	49.94	57.59	n.a.
2		12.967	265.311	497.060	50.06	42.41	n.a.
**Total:**			**529.998**	**1172.089**	**100.00**	**100.00**	

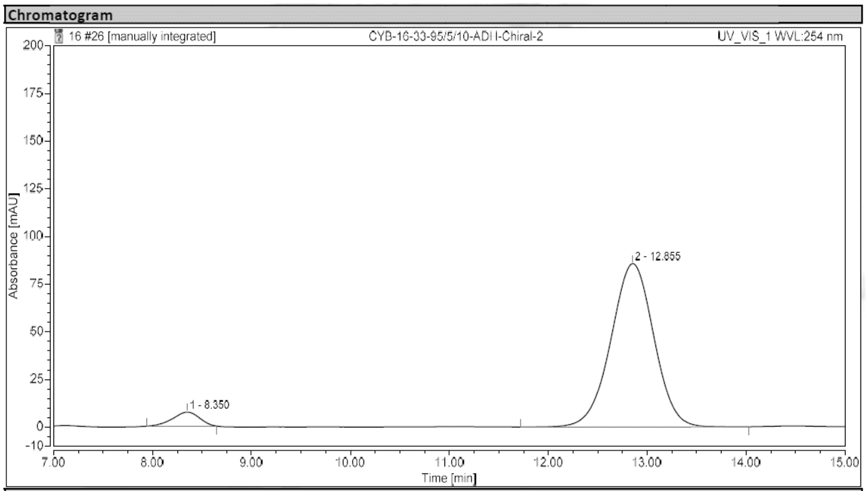

Integration Results

No.	Peak Name	Retention Time min	Area mAU*min	Height mAU	Relative Area %	Relative Height %	Amount n.a.
1		8.350	2.307	7.331	5.08	7.89	n.a.
2		12.855	43.124	85.632	94.92	92.11	n.a.
**Total:**			**45.430**	**92.963**	**100.00**	**100.00**	

Supplementary Figure 28. HPLC spectrum for compound **(+)-1h**

(-)-2h: ADH, *i*-PrOH/hexane = 5/95, v = 1.0 mL/min, λ = 254 nm

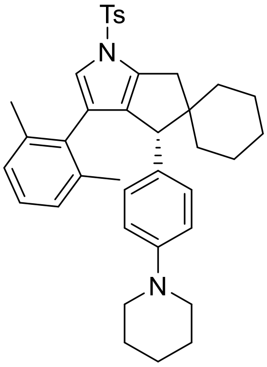

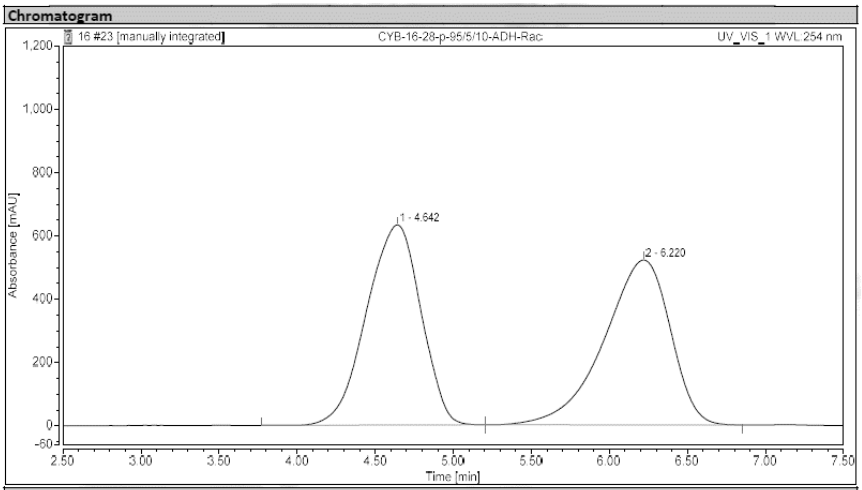

Integration Results

No.	Peak Name	Retention Time min	Area mAU*min	Height mAU	Relative Area %	Relative Height %	Amount n.a.
1		4.642	255.623	632.699	49.91	54.83	n.a.
2		6.220	256.545	521.308	50.09	45.17	n.a.
Total:			512.168	1154.007	100.00	100.00	

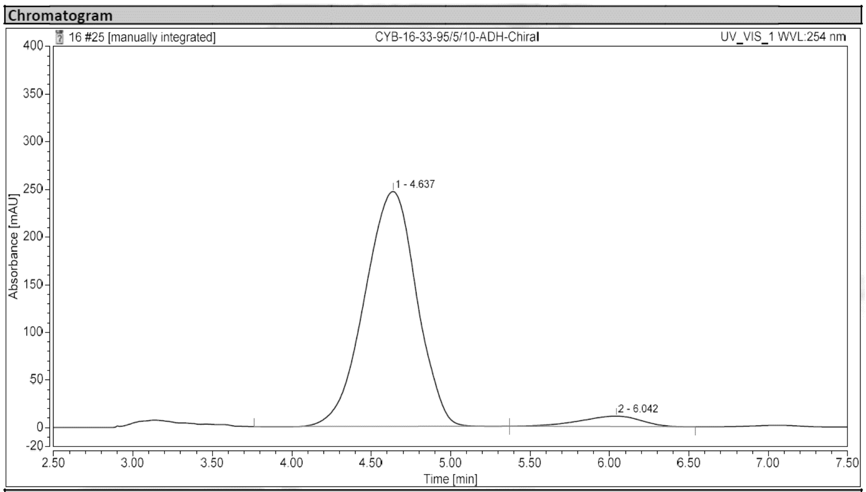

Integration Results

No.	Peak Name	Retention Time min	Area mAU*min	Height mAU	Relative Area %	Relative Height %	Amount n.a.
1		4.637	93.570	246.495	95.04	95.75	n.a.
2		6.042	4.879	10.954	4.96	4.25	n.a.
Total:			98.449	257.449	100.00	100.00	

Supplementary Figure 29. HPLC spectrum for compound **(-)-2h**

(+)-1i: ADH, *i*-PrOH/hexane = 20/80, v = 1.0 mL/min, λ = 254 nm

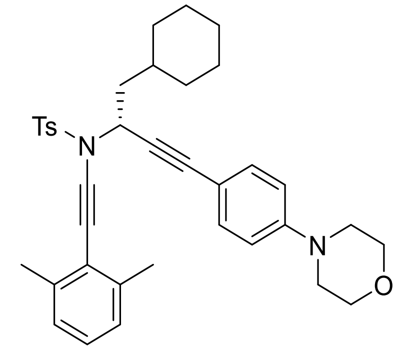

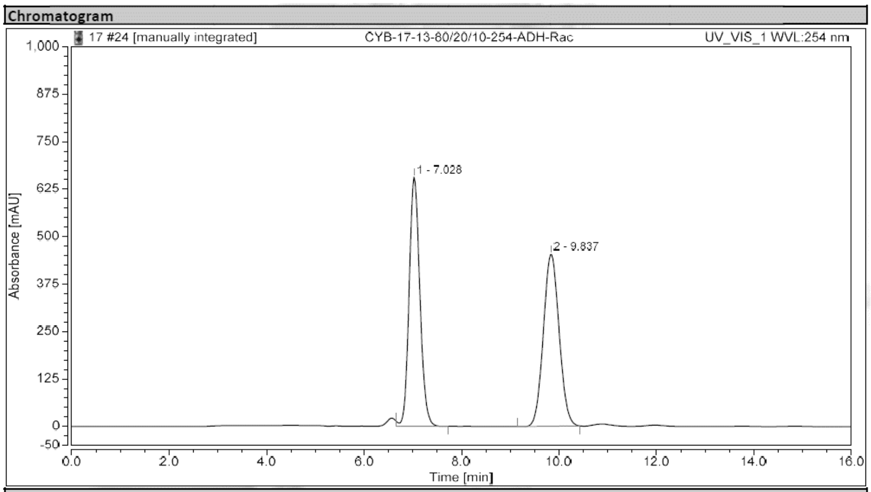

Integration Results

No.	Peak Name	Retention Time min	Area mAU*min	Height mAU	Relative Area %	Relative Height %	Amount n.a.
1		7.028	167.051	656.326	49.64	59.14	n.a.
2		9.837	169.450	453.514	50.36	40.86	n.a.
**Total:**			**336.501**	**1109.841**	**100.00**	**100.00**	

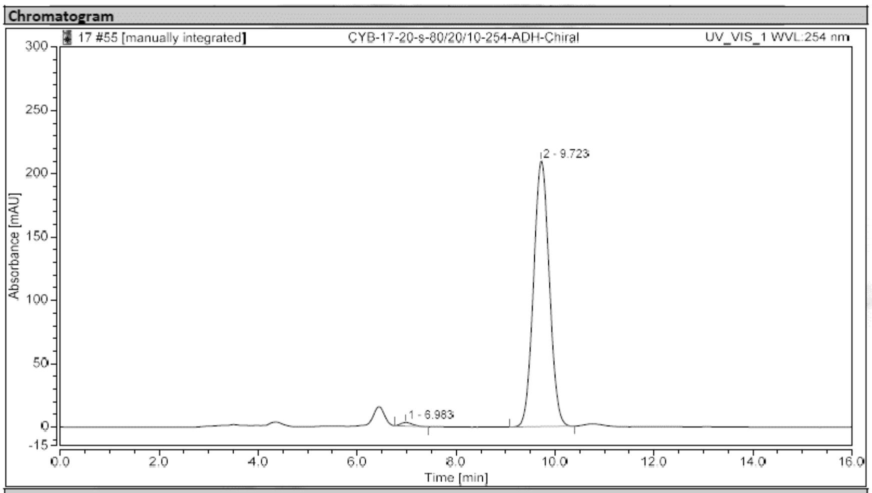

Integration Results

No.	Peak Name	Retention Time min	Area mAU*min	Height mAU	Relative Area %	Relative Height %	Amount n.a.
1		6.983	0.744	2.745	0.95	1.29	n.a.
2		9.723	77.508	209.891	99.05	98.71	n.a.
**Total:**			**78.252**	**212.636**	**100.00**	**100.00**	

Supplementary Figure 30. HPLC spectrum for compound **(+)-1i**

(-)-2i: ADH, *i*-PrOH/hexane = 5/95, v = 1.0 mL/min, λ = 254 nm

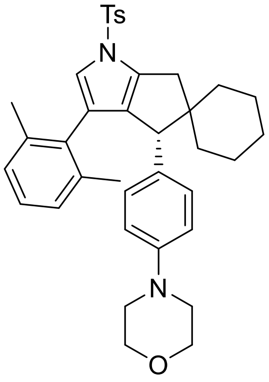

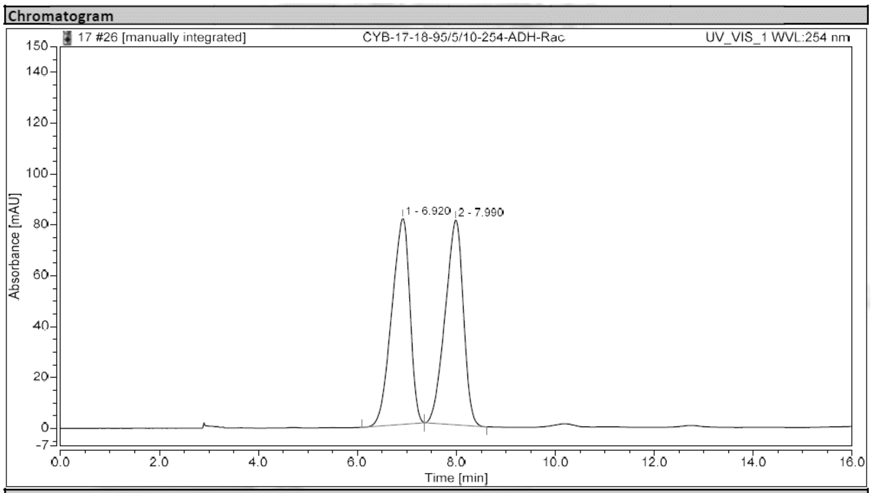

Integration Results

No.	Peak Name	Retention Time<br>min	Area<br>mAU*min	Height<br>mAU	Relative Area<br>%	Relative Height<br>%	Amount<br>n.a.
1		6.920	34.359	81.042	50.08	50.13	n.a.
2		7.990	34.245	80.605	49.92	49.87	n.a.
**Total:**			**68.605**	**161.647**	**100.00**	**100.00**	

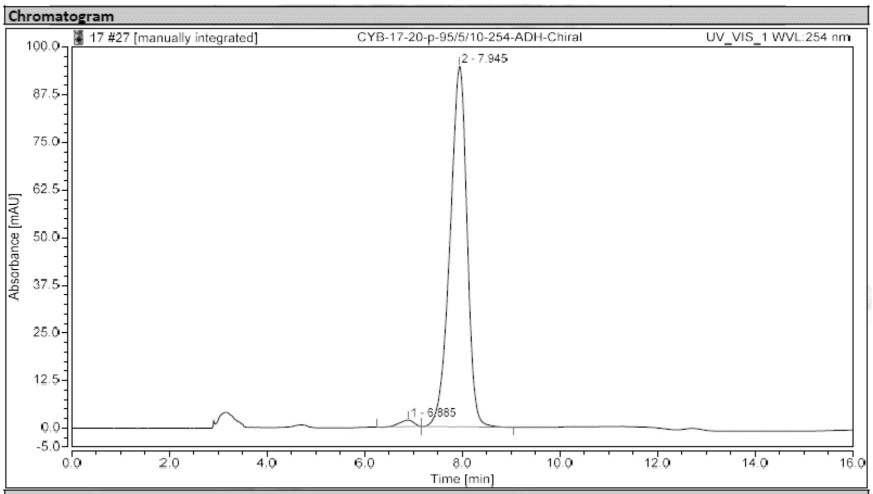

Integration Results

No.	Peak Name	Retention Time<br>min	Area<br>mAU*min	Height<br>mAU	Relative Area<br>%	Relative Height<br>%	Amount<br>n.a.
1		6.885	0.575	1.728	1.48	1.79	n.a.
2		7.945	38.269	94.713	98.52	98.21	n.a.
**Total:**			**38.844**	**96.441**	**100.00**	**100.00**	

Supplementary Figure 31. HPLC spectrum for compound **(-)-2i**

(+)-1j: IE, *i*-PrOH/hexane = 10/90, v = 1.0 mL/min, λ = 254 nm

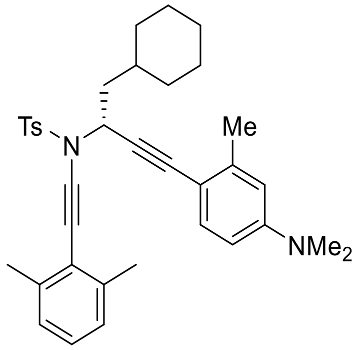

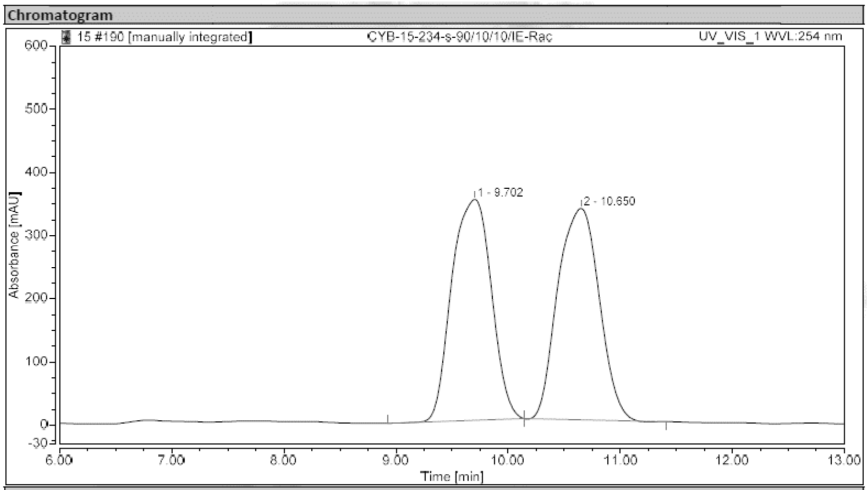

Integration Results

No.	Peak Name	Retention Time min	Area mAU*min	Height mAU	Relative Area %	Relative Height %	Amount n.a.
1		9.702	143.148	349.601	49.97	51.08	n.a.
2		10.650	143.339	334.797	50.03	48.92	n.a.
Total:			286.487	684.398	100.00	100.00	

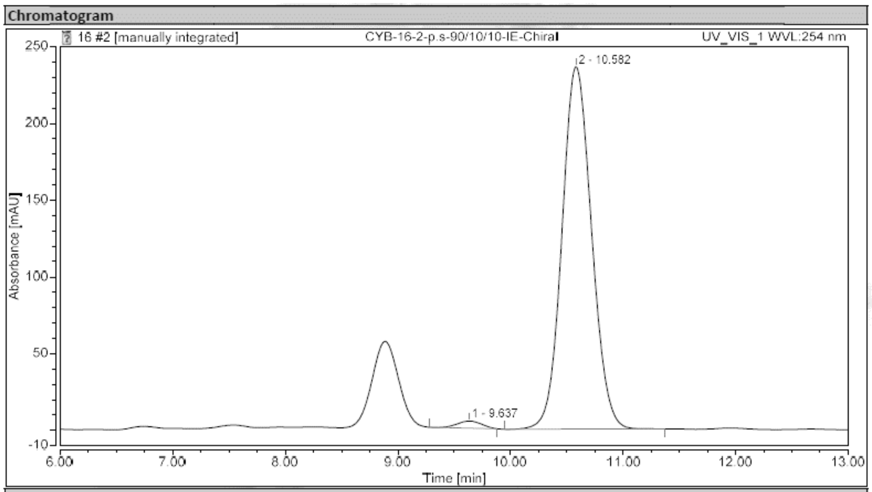

Integration Results

No.	Peak Name	Retention Time min	Area mAU*min	Height mAU	Relative Area %	Relative Height %	Amount n.a.
1		9.637	1.150	4.617	1.57	1.92	n.a.
2		10.582	72.036	236.232	98.43	98.08	n.a.
Total:			73.186	240.850	100.00	100.00	

Supplementary Figure 32. HPLC spectrum for compound **(+)-1j**

(-)-2j: IE, *i*-PrOH/hexane = 10/90, v = 1.0 mL/min, λ = 254 nm

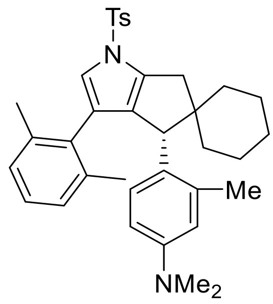

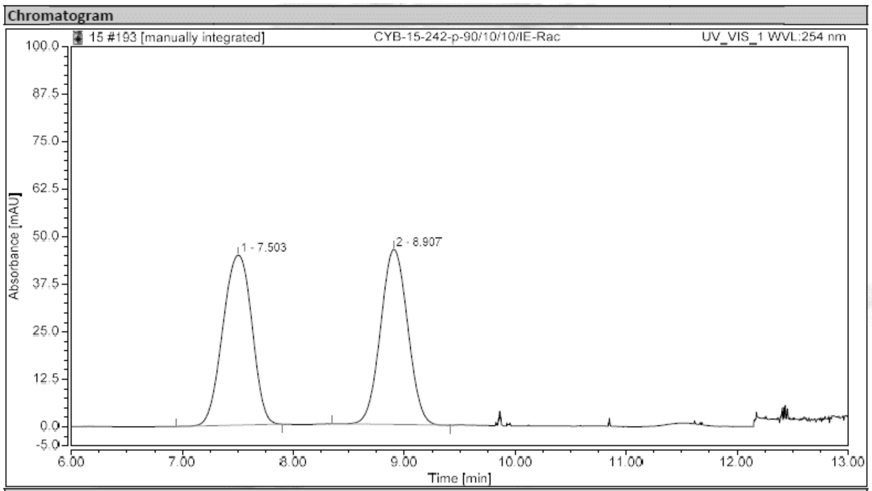

Integration Results

No.	Peak Name	Retention Time min	Area mAU*min	Height mAU	Relative Area %	Relative Height %	Amount n.a.
1		7.503	13.590	44.721	51.05	49.25	n.a.
2		8.907	13.031	46.087	48.95	50.75	n.a.
**Total:**			**26.621**	**90.808**	**100.00**	**100.00**	

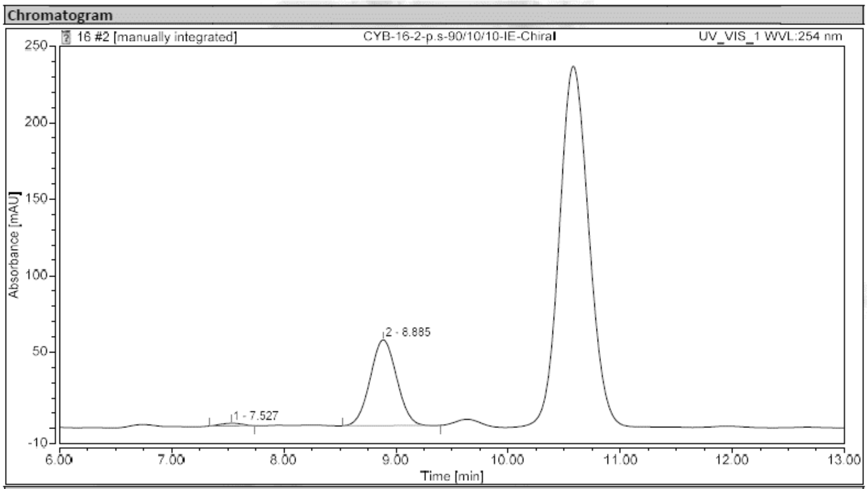

Integration Results

No.	Peak Name	Retention Time min	Area mAU*min	Height mAU	Relative Area %	Relative Height %	Amount n.a.
1		7.527	0.386	1.752	2.45	3.03	n.a.
2		8.885	15.342	56.079	97.55	96.97	n.a.
**Total:**			**15.728**	**57.831**	**100.00**	**100.00**	

Supplementary Figure 33. HPLC spectrum for compound **(-)-2j**

(+)-1k: IE, *i*-PrOH/hexane = 5/95, v = 1.0 mL/min, λ = 254 nm

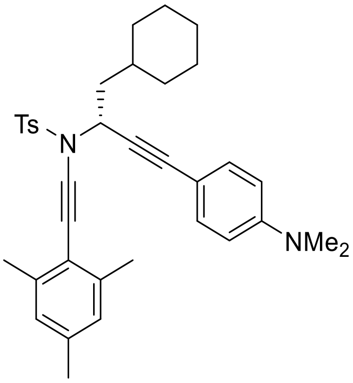

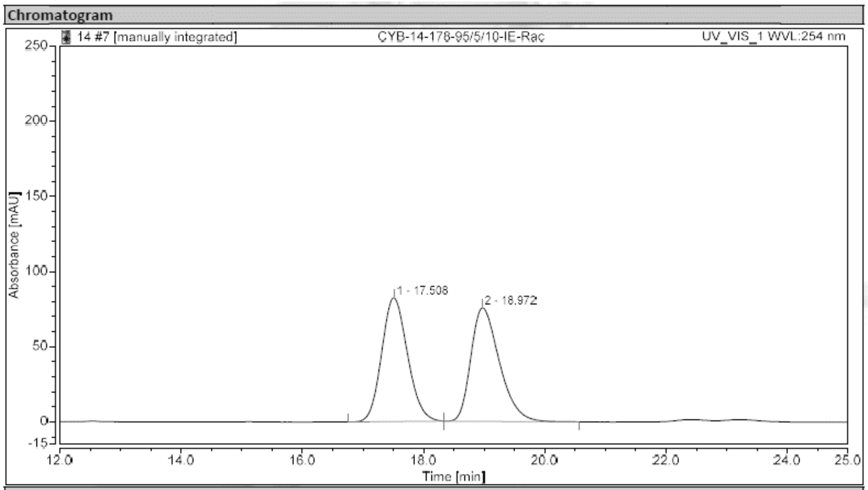

Integration Results

No.	Peak Name	Retention Time min	Area mAU*min	Height mAU	Relative Area %	Relative Height %	Amount n.a.
1		17.508	40.695	82.427	49.73	52.16	n.a.
2		18.972	41.138	75.603	50.27	47.84	n.a.
**Total:**			**81.833**	**158.031**	**100.00**	**100.00**	

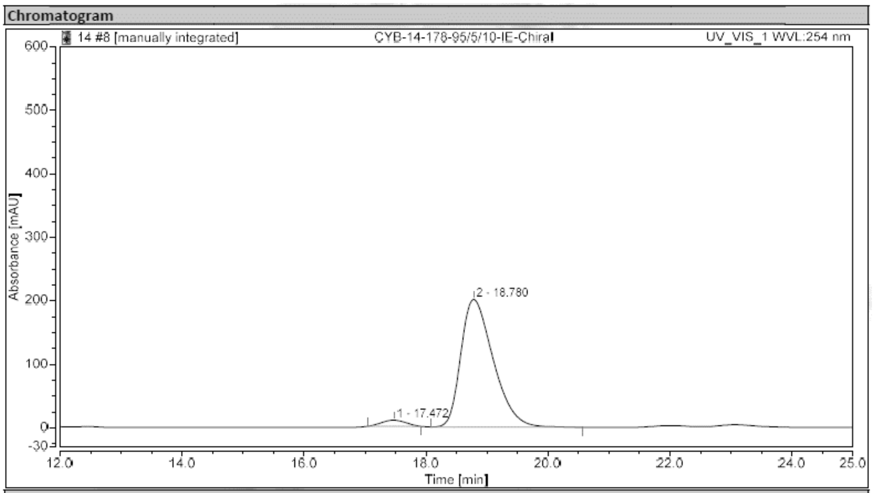

Integration Results

No.	Peak Name	Retention Time min	Area mAU*min	Height mAU	Relative Area %	Relative Height %	Amount n.a.
1		17.472	4.481	9.687	3.50	4.60	n.a.
2		18.780	123.664	200.985	96.50	95.40	n.a.
**Total:**			**128.145**	**210.672**	**100.00**	**100.00**	

Supplementary Figure 34. HPLC spectrum for compound **(+)-1k**

(-)-2k: IE, *i*-PrOH/hexane = 5/95, v = 1.0 mL/min, λ = 254 nm

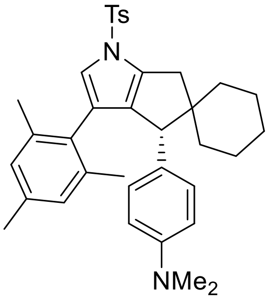

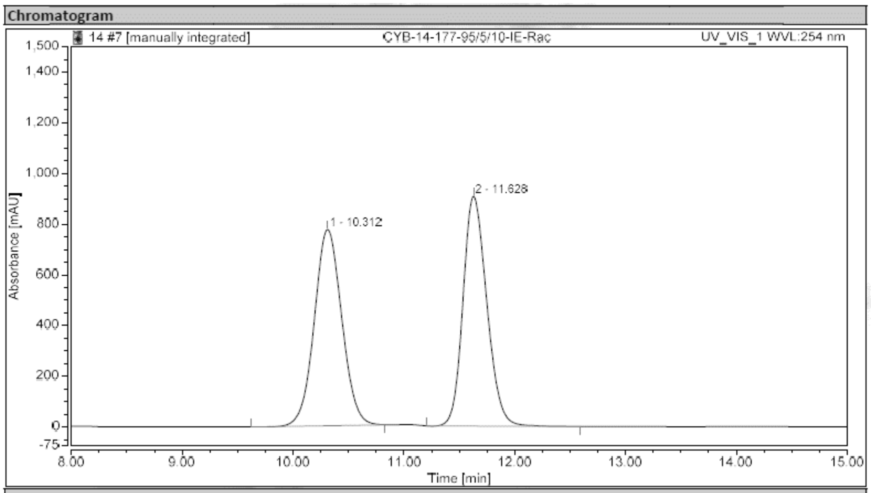

Integration Results

No.	Peak Name	Retention Time min	Area mAU*min	Height mAU	Relative Area %	Relative Height %	Amount n.a.
1		10.312	222.077	774.858	49.64	46.06	n.a.
2		11.628	225.261	907.470	50.36	53.94	n.a.
Total:			447.338	1682.328	100.00	100.00	

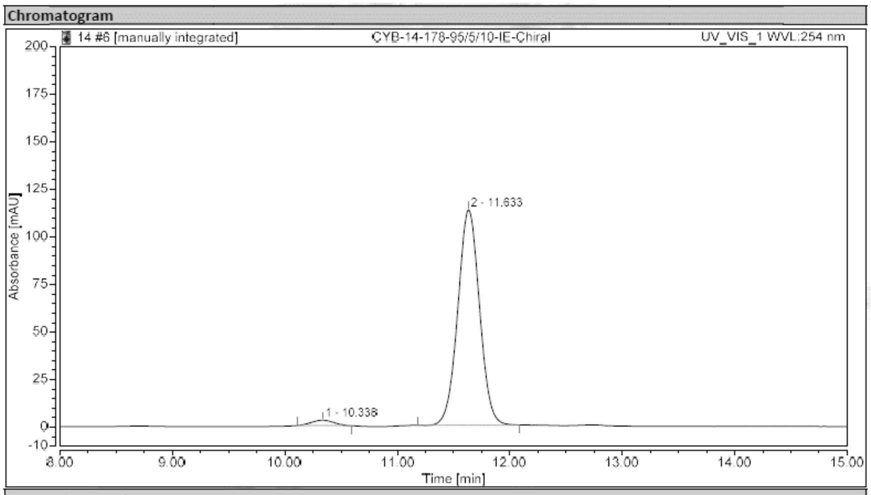

Integration Results

No.	Peak Name	Retention Time min	Area mAU*min	Height mAU	Relative Area %	Relative Height %	Amount n.a.
1		10.338	0.660	2.839	2.54	2.45	n.a.
2		11.633	25.307	113.200	97.46	97.55	n.a.
Total:			25.967	116.040	100.00	100.00	

Supplementary Figure 35. HPLC spectrum for compound **(-)-2k**

(+)-1l: IE, *i*-PrOH/hexane = 10/90, v = 1.0 mL/min, λ = 254 nm

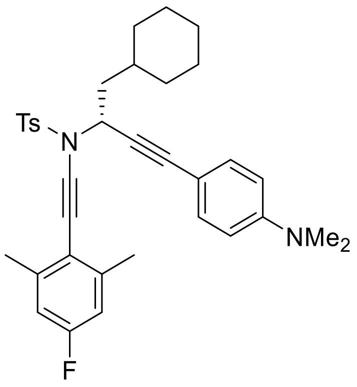

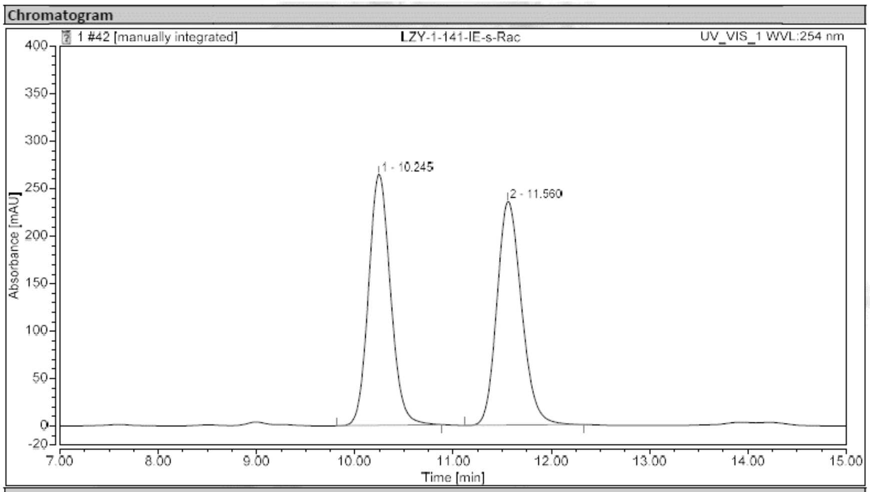

Integration Results

No.	Peak Name	Retention Time min	Area mAU*min	Height mAU	Relative Area %	Relative Height %	Amount n.a.
1		10.245	68.377	264.670	49.97	52.91	n.a.
2		11.560	68.465	235.598	50.03	47.09	n.a.
Total:			136.843	500.268	100.00	100.00	

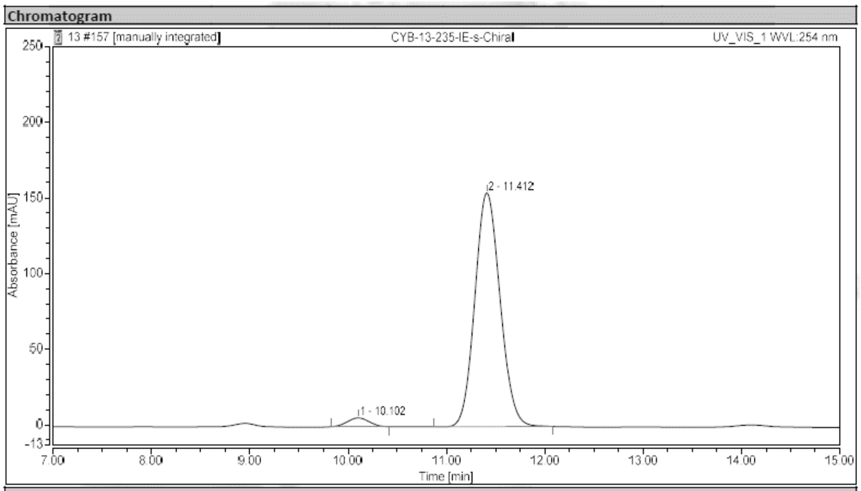

Integration Results

No.	Peak Name	Retention Time min	Area mAU*min	Height mAU	Relative Area %	Relative Height %	Amount n.a.
1		10.102	1.485	5.797	3.14	3.62	n.a.
2		11.412	45.825	154.448	96.86	96.38	n.a.
Total:			47.309	160.245	100.00	100.00	

Supplementary Figure 36. HPLC spectrum for compound **(+)-1l**

(-)-2l: IE, *i*-PrOH/hexane = 5/95, v = 1.0 mL/min, λ = 254 nm

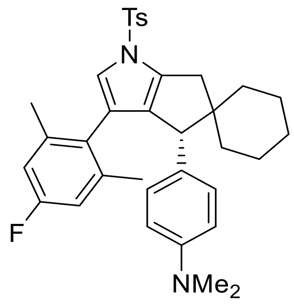

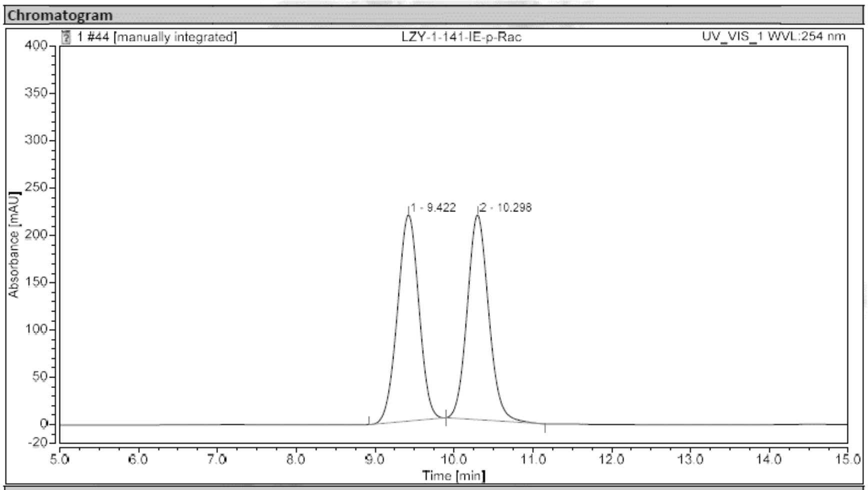

Integration Results

No.	Peak Name	Retention Time min	Area mAU*min	Height mAU	Relative Area %	Relative Height %	Amount n.a.
1		9.422	68.214	218.201	49.97	50.18	n.a.
2		10.298	68.300	216.652	50.03	49.82	n.a.
**Total:**			**136.513**	**434.853**	**100.00**	**100.00**	

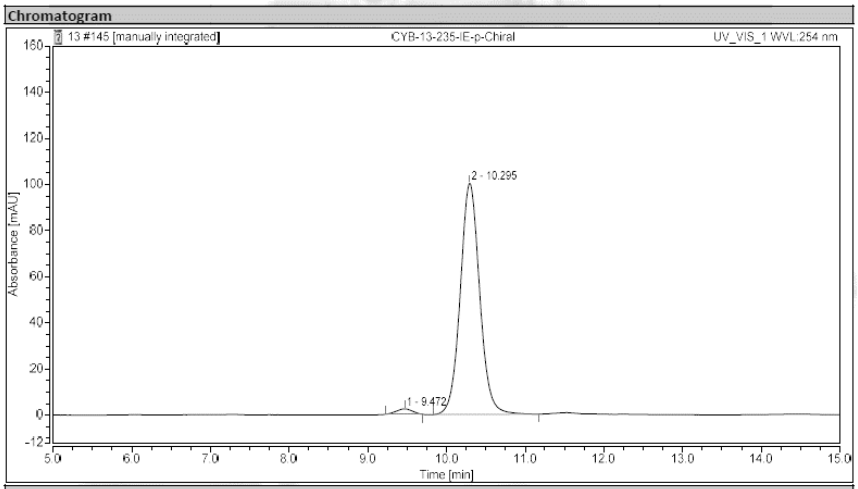

Integration Results

No.	Peak Name	Retention Time min	Area mAU*min	Height mAU	Relative Area %	Relative Height %	Amount n.a.
1		9.472	0.559	2.362	1.91	2.30	n.a.
2		10.295	28.643	100.284	98.09	97.70	n.a.
**Total:**			**29.202**	**102.647**	**100.00**	**100.00**	

Supplementary Figure 37. HPLC spectrum for compound **(-)-2l**

(+)-1m: ADH, *i*-PrOH/hexane = 5/95, v = 1.0 mL/min, λ = 254 nm

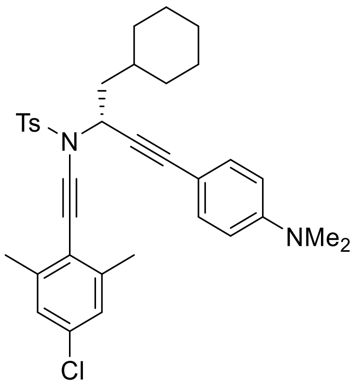

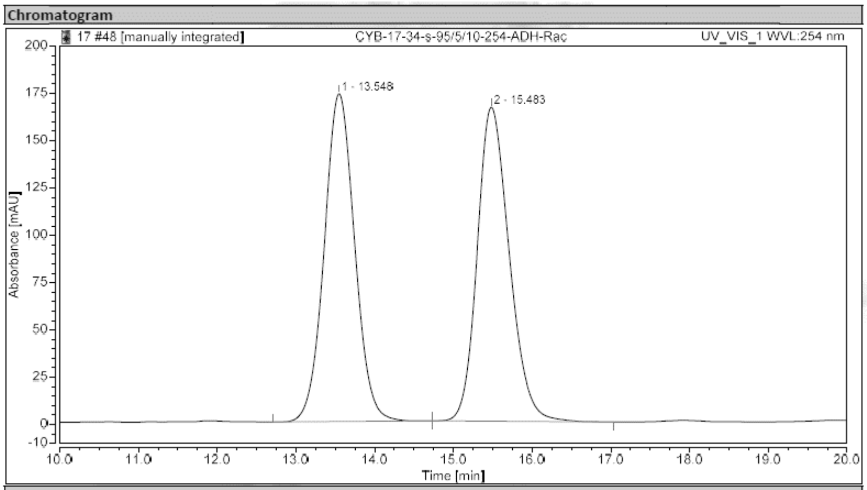

Integration Results

No.	Peak Name	Retention Time min	Area mAU*min	Height mAU	Relative Area %	Relative Height %	Amount n.a.
1		13.548	77.475	173.317	49.77	51.07	n.a.
2		15.483	78.197	166.021	50.23	48.93	n.a.
Total:			155.673	339.338	100.00	100.00	

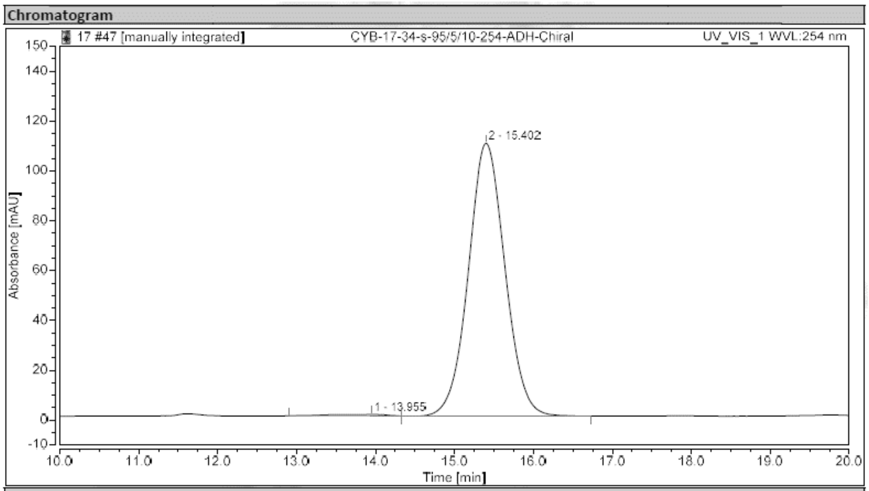

Integration Results

No.	Peak Name	Retention Time min	Area mAU*min	Height mAU	Relative Area %	Relative Height %	Amount n.a.
1		13.955	0.491	0.605	0.83	0.55	n.a.
2		15.402	58.788	109.479	99.17	99.45	n.a.
Total:			59.279	110.084	100.00	100.00	

Supplementary Figure 38. HPLC spectrum for compound **(+)-1m**

(-)-2m: IE, *i*-PrOH/hexane = 5/95, v = 1.0 mL/min, λ = 254 nm

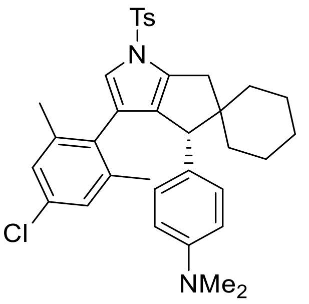

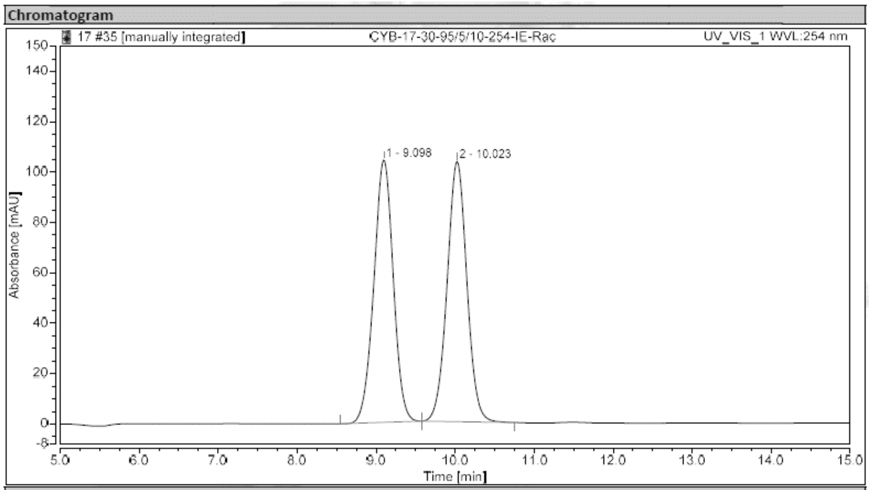

Integration Results

No.	Peak Name	Retention Time min	Area mAU*min	Height mAU	Relative Area %	Relative Height %	Amount n.a.
1		9.098	29.743	104.225	49.97	50.21	n.a.
2		10.023	29.781	103.343	50.03	49.79	n.a.
**Total:**			**59.524**	**207.568**	**100.00**	**100.00**	

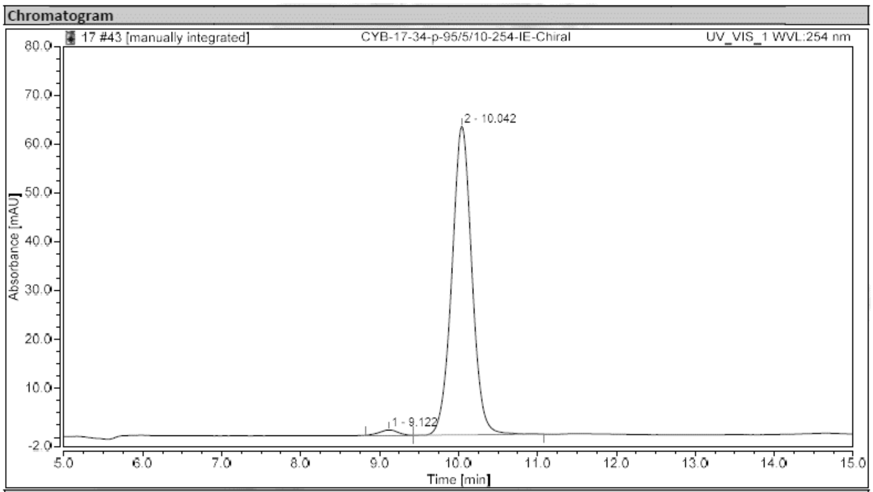

Integration Results

No.	Peak Name	Retention Time min	Area mAU*min	Height mAU	Relative Area %	Relative Height %	Amount n.a.
1		9.122	0.292	1.102	1.55	1.71	n.a.
2		10.042	18.579	63.264	98.45	98.29	n.a.
**Total:**			**18.871**	**64.365**	**100.00**	**100.00**	

Supplementary Figure 39. HPLC spectrum for compound **(-)-2m**

(+)-1n: IE, *i*-PrOH/hexane = 10/90, v = 1.0 mL/min, λ = 254 nm

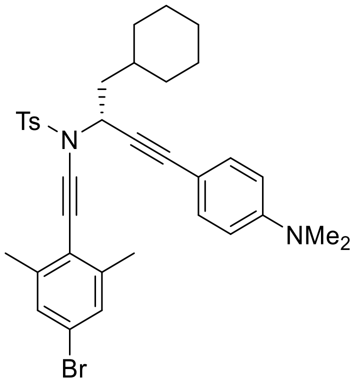

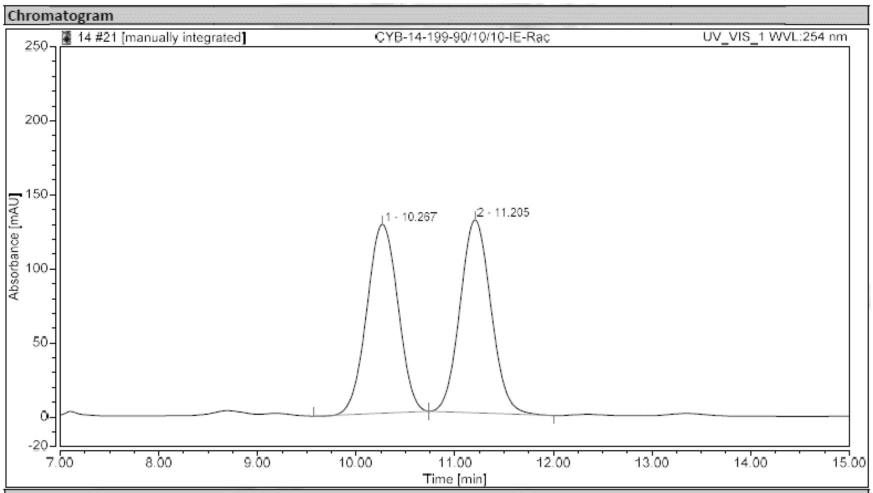

Integration Results

No.	Peak Name	Retention Time<br>min	Area<br>mAU*min	Height<br>mAU	Relative Area<br>%	Relative Height<br>%	Amount<br>n.a.
1		10.267	46.555	127.612	49.59	49.47	n.a.
2		11.205	47.324	130.364	50.41	50.53	n.a.
Total:			93.879	257.977	100.00	100.00	

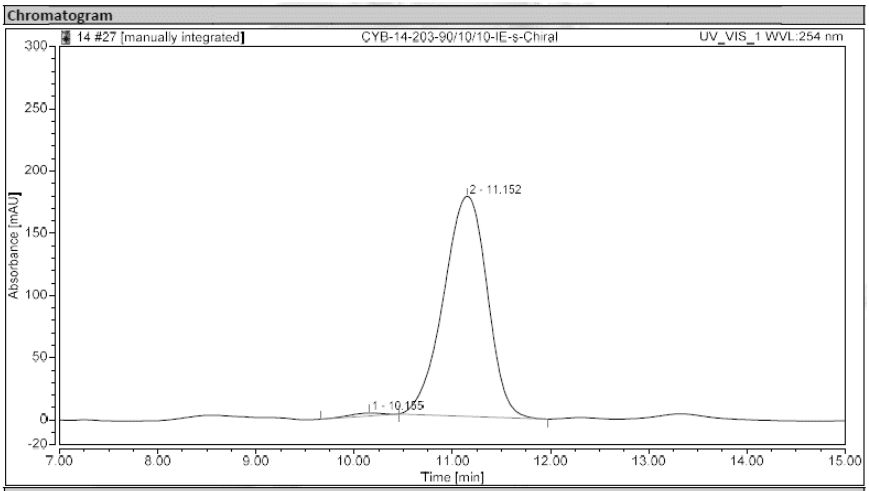

Integration Results

No.	Peak Name	Retention Time<br>min	Area<br>mAU*min	Height<br>mAU	Relative Area<br>%	Relative Height<br>%	Amount<br>n.a.
1		10.155	0.901	2.287	0.98	1.28	n.a.
2		11.152	91.092	176.773	99.02	98.72	n.a.
Total:			91.993	179.060	100.00	100.00	

Supplementary Figure 40. HPLC spectrum for compound **(+)-1n**

(-)-2n: IC, *i*-PrOH/hexane = 5/95, v = 1.0 mL/min, λ = 254 nm

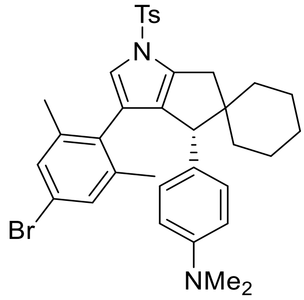

Chromatogram

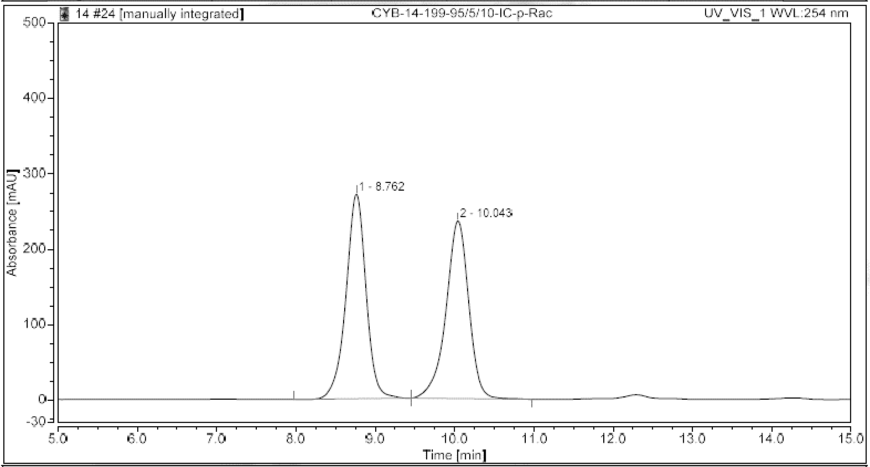

Integration Results

No.	Peak Name	Retention Time min	Area mAU*min	Height mAU	Relative Area %	Relative Height %	Amount n.a.
1		8.762	79.406	271.731	50.37	53.53	n.a.
2		10.043	78.252	235.920	49.63	46.47	n.a.
Total:			157.658	507.651	100.00	100.00	

Chromatogram

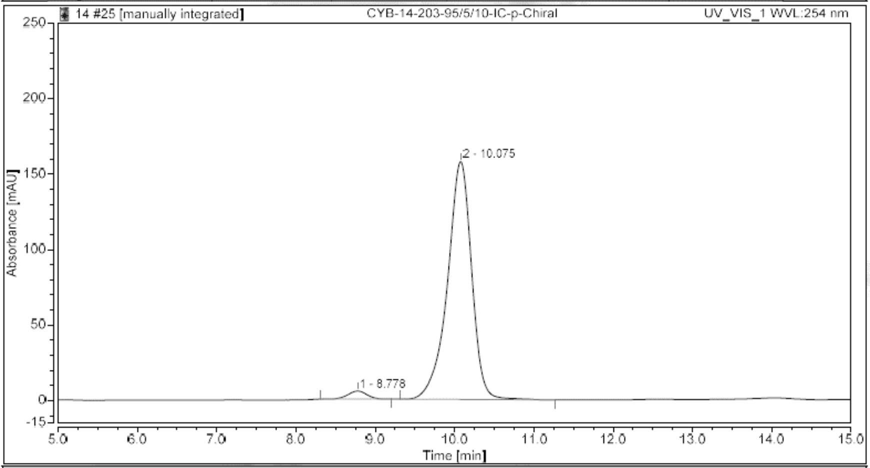

Integration Results

No.	Peak Name	Retention Time min	Area mAU*min	Height mAU	Relative Area %	Relative Height %	Amount n.a.
1		8.778	1.459	5.345	2.56	3.28	n.a.
2		10.075	55.521	157.805	97.44	96.72	n.a.
Total:			56.980	163.150	100.00	100.00	

Supplementary Figure 41. HPLC spectrum for compound **(-)-2n**

(+)-1o: ADH, *i*-PrOH/hexane = 10/90, v = 1.0 mL/min, λ = 254 nm

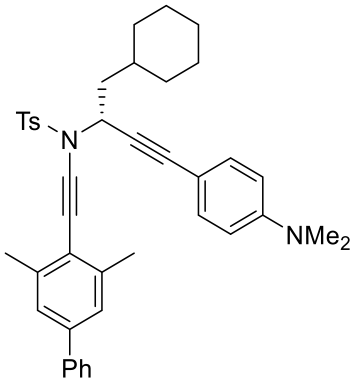

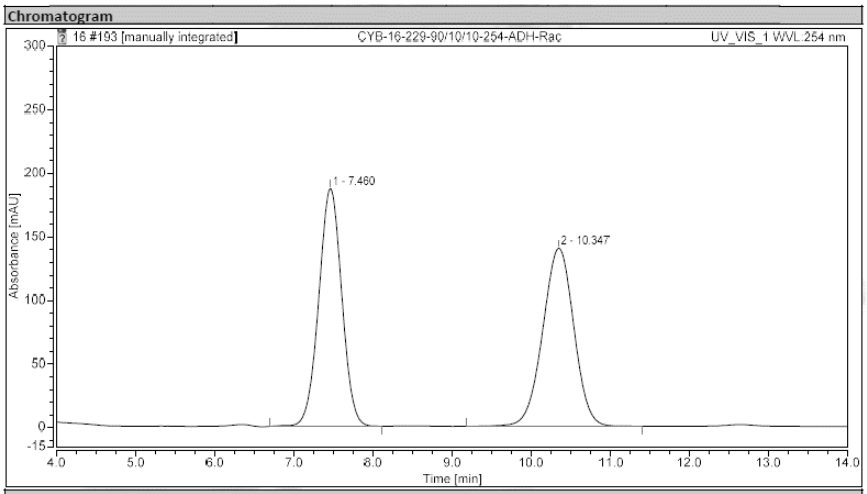

Integration Results

No.	Peak Name	Retention Time min	Area mAU*min	Height mAU	Relative Area %	Relative Height %	Amount n.a.
1		7.460	63.173	187.106	49.66	57.17	n.a.
2		10.347	64.029	140.182	50.34	42.83	n.a.
Total:			127.203	327.287	100.00	100.00	

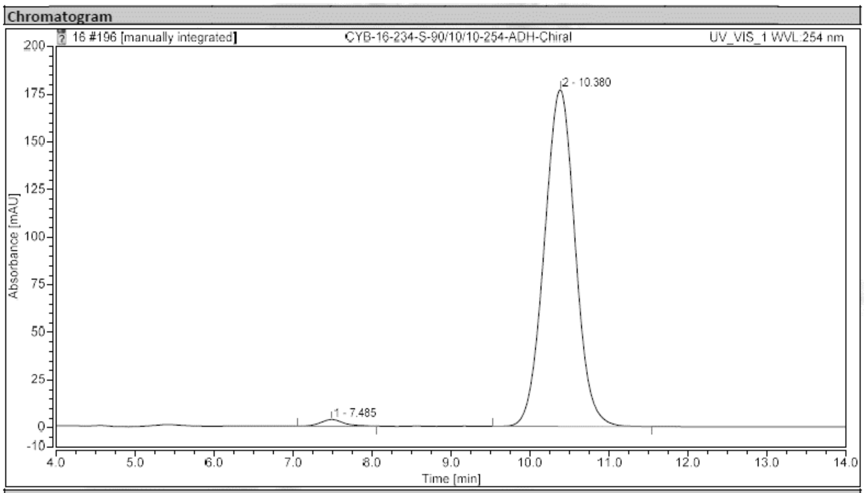

Integration Results

No.	Peak Name	Retention Time min	Area mAU*min	Height mAU	Relative Area %	Relative Height %	Amount n.a.
1		7.485	1.139	3.465	1.41	1.92	n.a.
2		10.380	79.753	176.767	98.59	98.08	n.a.
Total:			80.892	180.232	100.00	100.00	

Supplementary Figure 42. HPLC spectrum for compound **(+)-1o**

(-)-2o: IC, *i*-PrOH/hexane = 5/95, v = 1.0 mL/min, λ = 254 nm

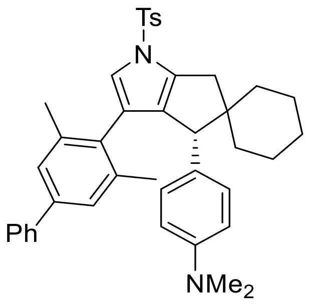

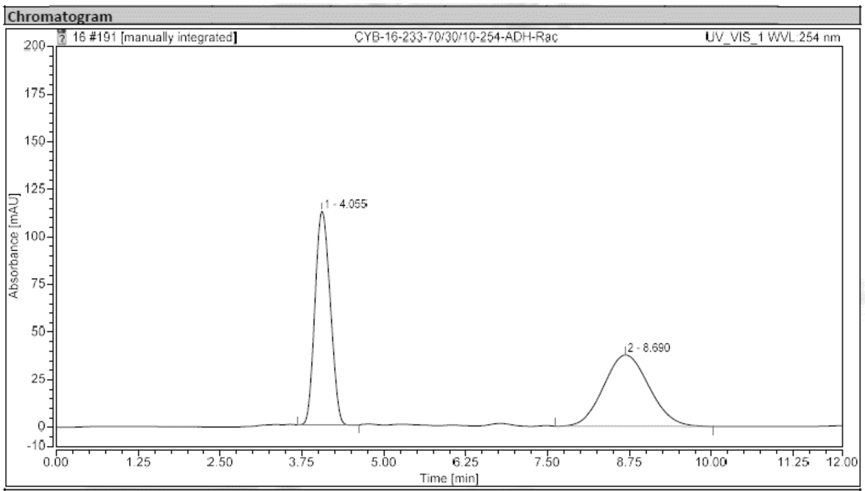

Integration Results

No.	Peak Name	Retention Time min	Area mAU*min	Height mAU	Relative Area %	Relative Height %	Amount n.a.
1		4.055	30.288	112.220	50.19	74.99	n.a.
2		8.690	30.065	37.421	49.81	25.01	n.a.
**Total:**			**60.353**	**149.642**	**100.00**	**100.00**	

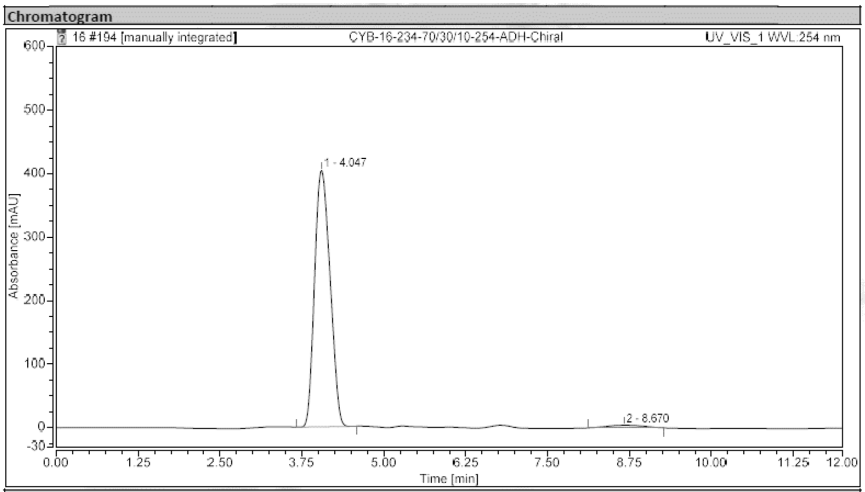

Integration Results

No.	Peak Name	Retention Time min	Area mAU*min	Height mAU	Relative Area %	Relative Height %	Amount n.a.
1		4.047	110.731	404.027	97.56	98.95	n.a.
2		8.670	2.766	4.291	2.44	1.05	n.a.
**Total:**			**113.498**	**408.318**	**100.00**	**100.00**	

Supplementary Figure 43. HPLC spectrum for compound **(-)-2o**

(+)-1p: ADH, *i*-PrOH/hexane = 5/95, v = 1.0 mL/min, λ = 254 nm

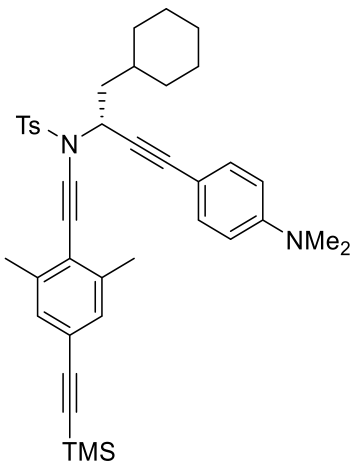

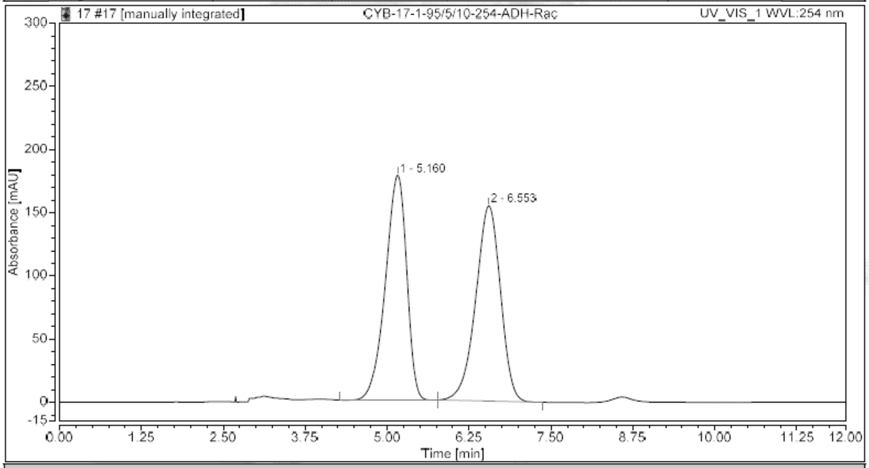

Integration Results

No.	Peak Name	Retention Time min	Area mAU*min	Height mAU	Relative Area %	Relative Height %	Amount n.a.
1		5.160	66.899	178.065	49.69	53.52	n.a.
2		6.553	67.726	154.626	50.31	46.48	n.a.
Total:			134.624	332.690	100.00	100.00	

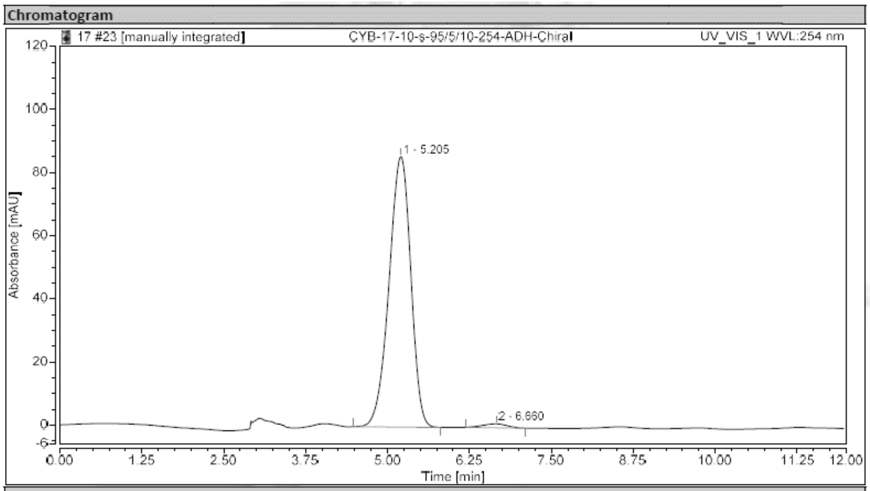

Integration Results

No.	Peak Name	Retention Time min	Area mAU*min	Height mAU	Relative Area %	Relative Height %	Amount n.a.
1		5.205	32.964	85.705	98.47	98.60	n.a.
2		6.660	0.511	1.221	1.53	1.40	n.a.
Total:			33.475	86.926	100.00	100.00	

Supplementary Figure 44. HPLC spectrum for compound **(+)-1p**

(-)-2p: ADH, *i*-PrOH/hexane = 5/95, v = 1.0 mL/min, λ = 254 nm

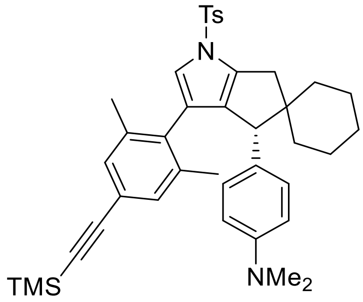

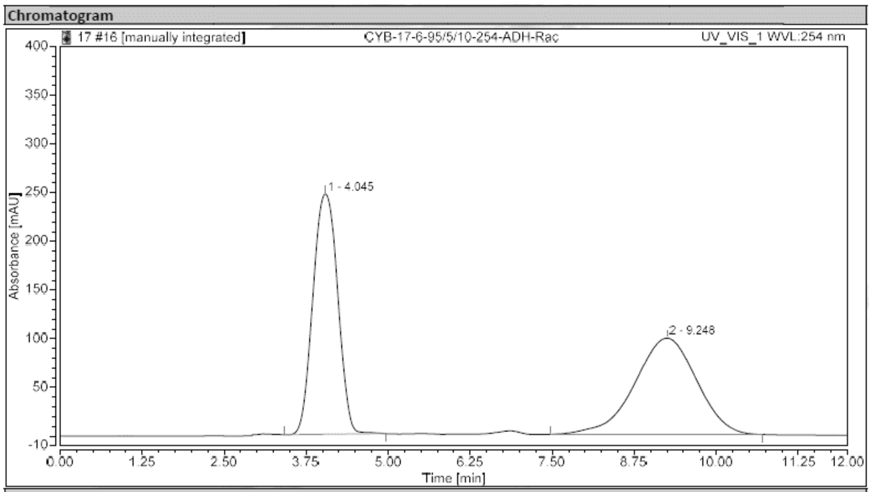

Integration Results

No.	Peak Name	Retention Time min	Area mAU*min	Height mAU	Relative Area %	Relative Height %	Amount n.a.
1		4.045	108.101	247.050	49.96	71.42	n.a.
2		9.248	108.253	98.872	50.04	28.58	n.a.
**Total:**			**216.354**	**345.922**	**100.00**	**100.00**	

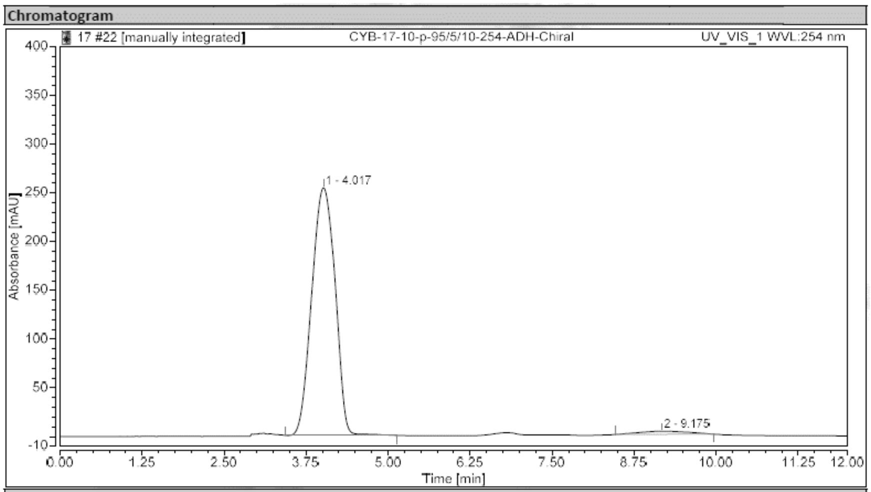

Integration Results

No.	Peak Name	Retention Time min	Area mAU*min	Height mAU	Relative Area %	Relative Height %	Amount n.a.
1		4.017	105.033	254.373	97.50	98.83	n.a.
2		9.175	2.696	3.020	2.50	1.17	n.a.
**Total:**			**107.729**	**257.393**	**100.00**	**100.00**	

Supplementary Figure 45. HPLC spectrum for compound **(-)-2p**

(+)-1q: IE, *i*-PrOH/hexane = 20/80, v = 1.0 mL/min, λ = 254 nm

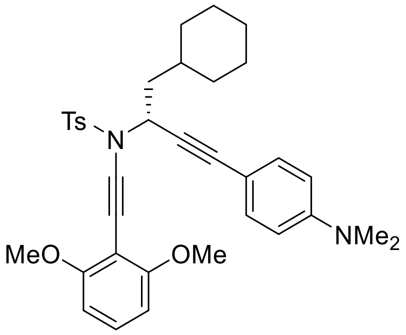

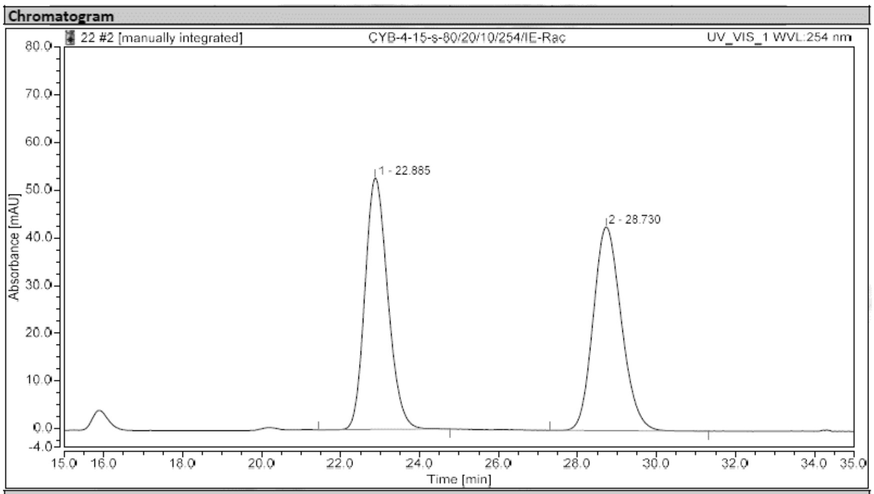

Integration Results

No.	Peak Name	Retention Time min	Area mAU*min	Height mAU	Relative Area %	Relative Height %	Amount n.a.
1		22.885	35.376	52.786	49.97	55.24	n.a.
2		28.730	35.417	42.776	50.03	44.76	n.a.
Total:			70.792	95.562	100.00	100.00	

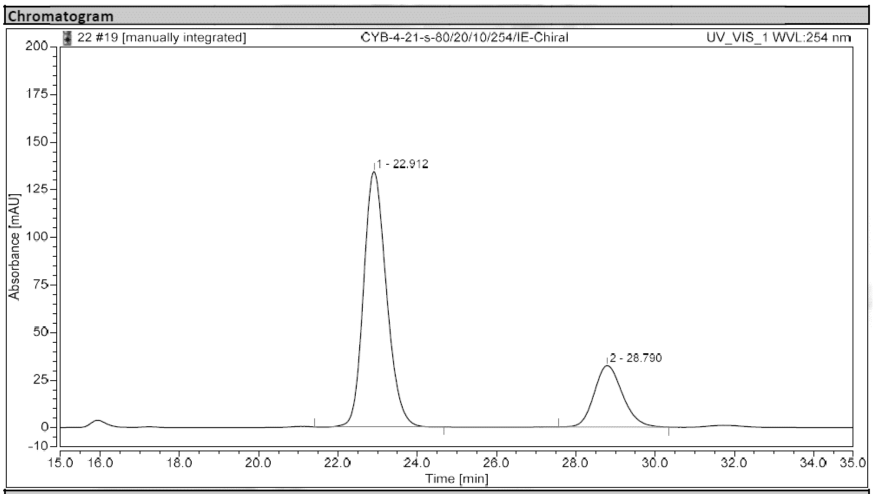

Integration Results

No.	Peak Name	Retention Time min	Area mAU*min	Height mAU	Relative Area %	Relative Height %	Amount n.a.
1		22.912	89.759	134.176	77.14	80.64	n.a.
2		28.790	26.593	32.210	22.86	19.36	n.a.
Total:			116.352	166.386	100.00	100.00	

Supplementary Figure 46. HPLC spectrum for compound **(+)-1q**

(-)-2q: IE, *i*-PrOH/hexane = 30/70, v = 1.0 mL/min, λ = 254 nm

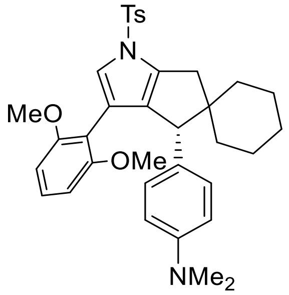

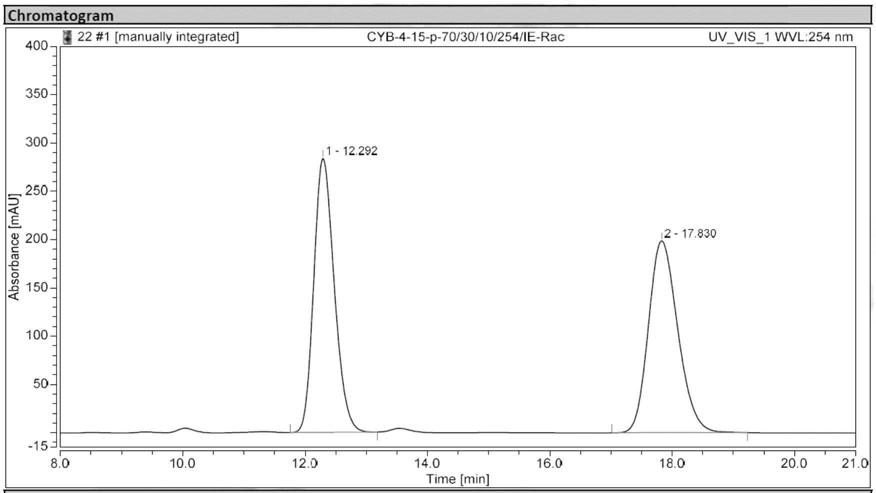

Integration Results

No.	Peak Name	Retention Time min	Area mAU*min	Height mAU	Relative Area %	Relative Height %	Amount n.a.
1		12.292	109.978	283.782	49.88	58.81	n.a.
2		17.830	110.505	198.726	50.12	41.19	n.a.
**Total:**			**220.483**	**482.507**	**100.00**	**100.00**	

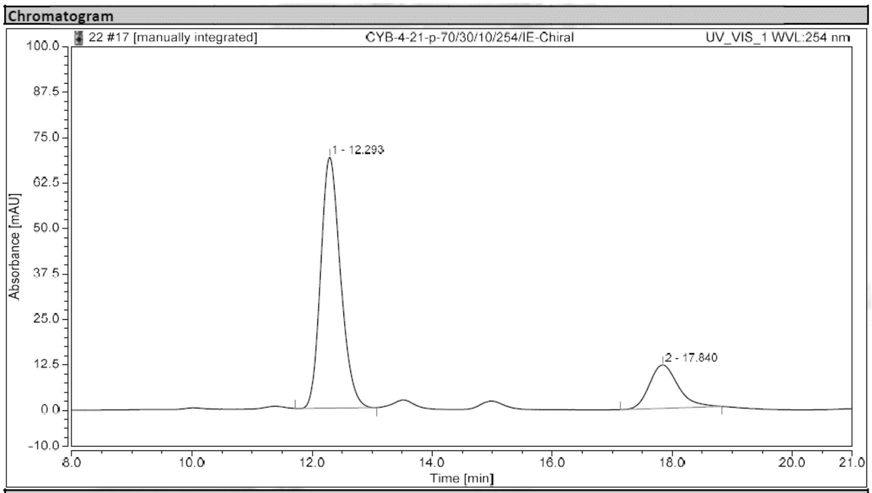

Integration Results

No.	Peak Name	Retention Time min	Area mAU*min	Height mAU	Relative Area %	Relative Height %	Amount n.a.
1		12.293	26.520	69.098	80.01	85.27	n.a.
2		17.840	6.627	11.941	19.99	14.73	n.a.
**Total:**			**33.147**	**81.039**	**100.00**	**100.00**	

Supplementary Figure 47. HPLC spectrum for compound **(-)-2q**

(+)-1r: ODH, *i*-PrOH/hexane = 3/97, v = 1.0 mL/min, λ = 254 nm

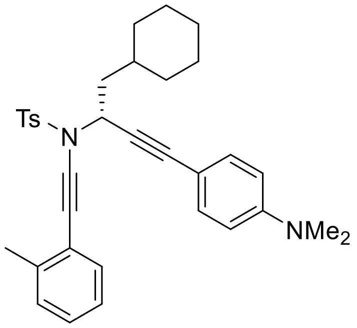

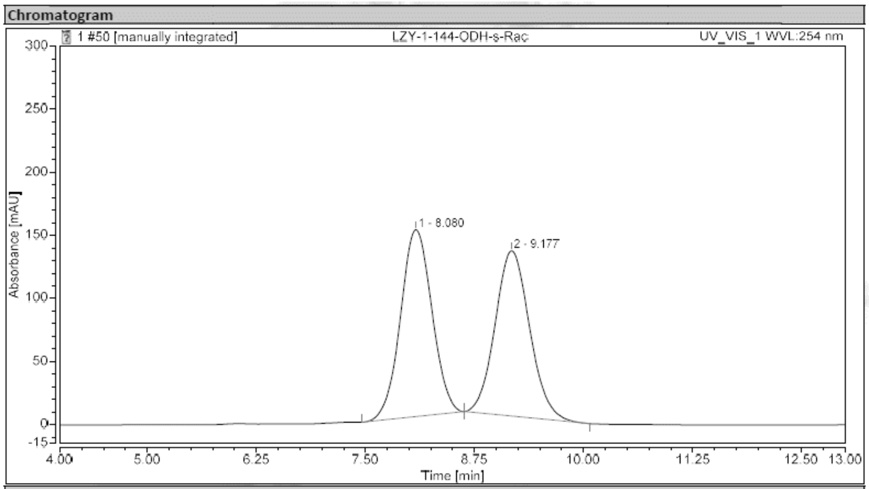

Integration Results

No.	Peak Name	Retention Time min	Area mAU*min	Height mAU	Relative Area %	Relative Height %	Amount n.a.
1		8.080	61.856	148.198	50.72	53.07	n.a.
2		9.177	60.104	131.043	49.28	46.93	n.a.
**Total:**			**121.960**	**279.241**	**100.00**	**100.00**	

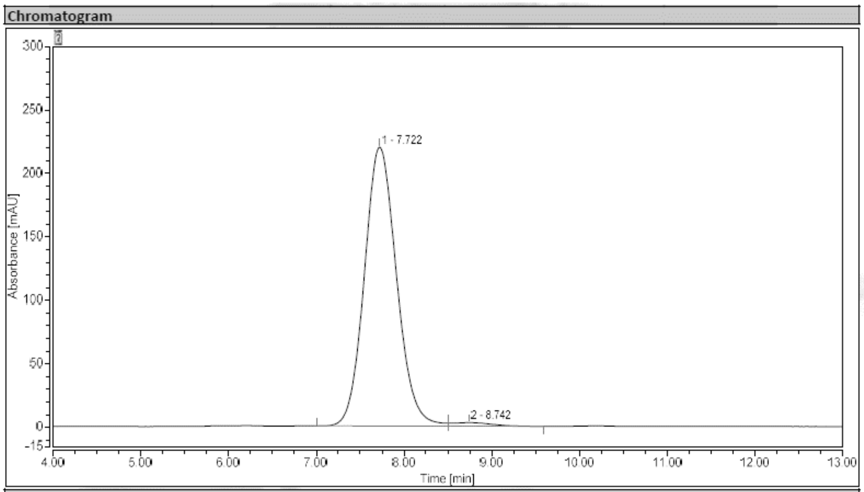

Integration Results

No.	Peak Name	Retention Time min	Area mAU*min	Height mAU	Relative Area %	Relative Height %	Amount n.a.
1		7.722	93.416	219.773	98.46	98.73	n.a.
2		8.742	1.460	2.834	1.54	1.27	n.a.
**Total:**			**94.875**	**222.607**	**100.00**	**100.00**	

Supplementary Figure 48. HPLC spectrum for compound **(+)-1r**

(-)-2r: IE, *i*-PrOH/hexane = 10/90, v = 1.0 mL/min, λ = 254 nm

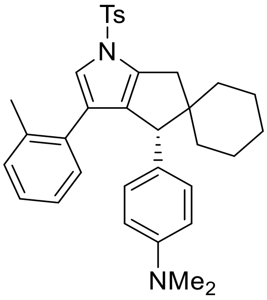

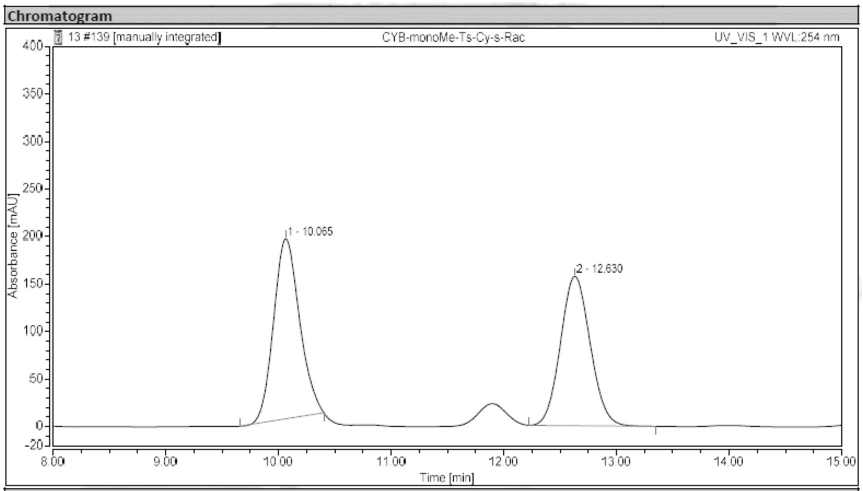

Integration Results

No.	Peak Name	Retention Time min	Area mAU*min	Height mAU	Relative Area %	Relative Height %	Amount n.a.
1		10.065	50.459	189.539	51.22	54.67	n.a.
2		12.630	48.048	157.131	48.78	45.33	n.a.
**Total:**			**98.506**	**346.669**	**100.00**	**100.00**	

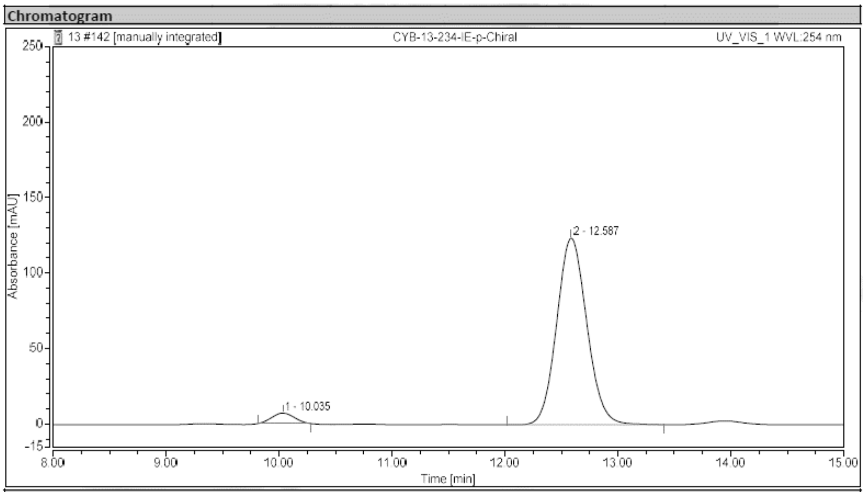

Integration Results

No.	Peak Name	Retention Time min	Area mAU*min	Height mAU	Relative Area %	Relative Height %	Amount n.a.
1		10.035	1.602	6.944	4.03	5.32	n.a.
2		12.587	38.140	123.489	95.97	94.68	n.a.
**Total:**			**39.742**	**130.433**	**100.00**	**100.00**	

Supplementary Figure 49. HPLC spectrum for compound **(-)-2r**

(+)-1s: ADH, *i*-PrOH/hexane = 5/95, v = 1.0 mL/min, λ = 254 nm

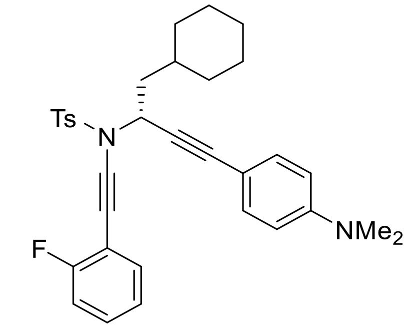

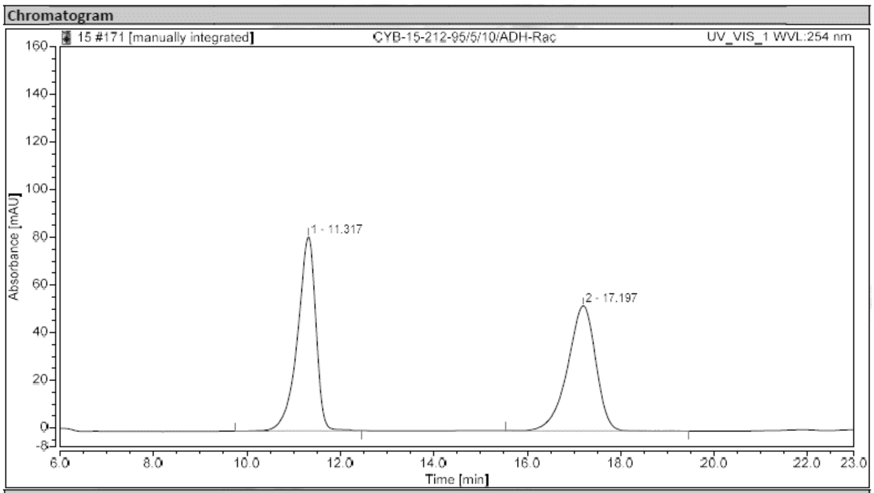

Integration Results

No.	Peak Name	Retention Time min	Area mAU*min	Height mAU	Relative Area %	Relative Height %	Amount n.a.
1		11.317	37.083	81.472	50.32	60.80	n.a.
2		17.197	36.615	52.526	49.68	39.20	n.a.
Total:			73.697	133.998	100.00	100.00	

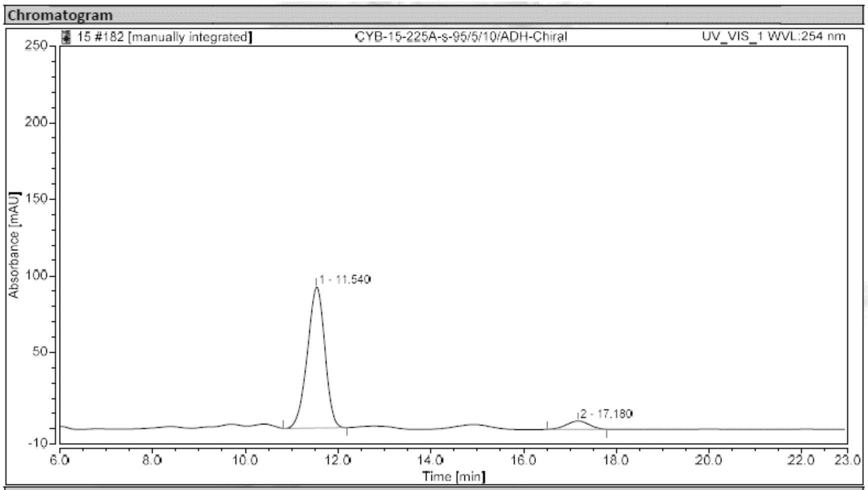

Integration Results

No.	Peak Name	Retention Time min	Area mAU*min	Height mAU	Relative Area %	Relative Height %	Amount n.a.
1		11.540	40.245	92.091	92.91	94.39	n.a.
2		17.180	3.072	5.475	7.09	5.61	n.a.
Total:			43.318	97.566	100.00	100.00	

Supplementary Figure 50. HPLC spectrum for compound **(+)-1s**

(-)-2s: IE, *i*-PrOH/hexane = 10/90, v = 1.0 mL/min, λ = 254 nm

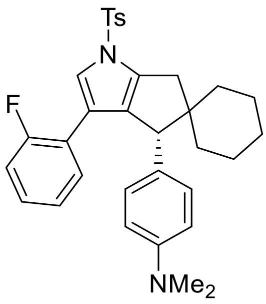

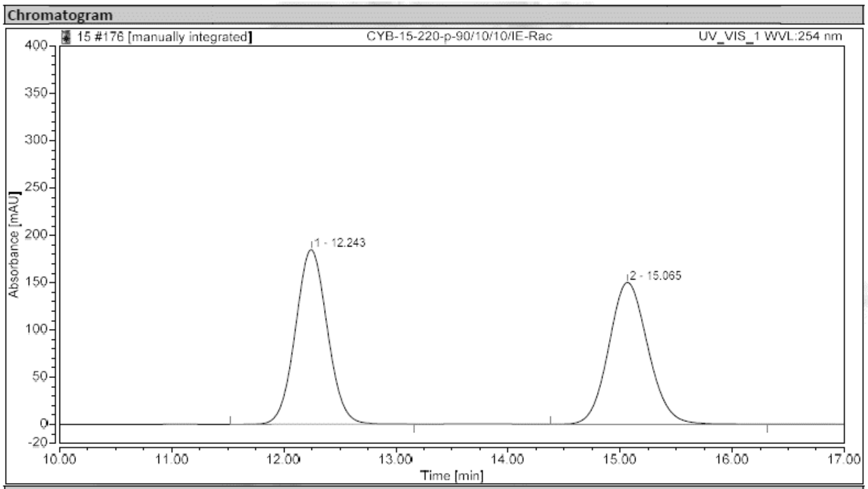

Integration Results

No.	Peak Name	Retention Time min	Area mAU*min	Height mAU	Relative Area %	Relative Height %	Amount n.a.
1		12.243	60.850	184.510	50.05	55.17	n.a.
2		15.065	60.730	149.951	49.95	44.83	n.a.
Total:			121.580	334.461	100.00	100.00	

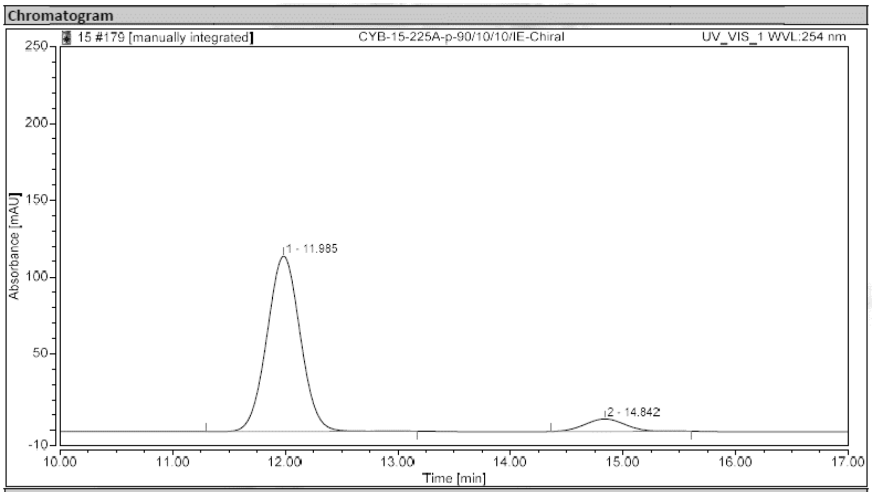

Integration Results

No.	Peak Name	Retention Time min	Area mAU*min	Height mAU	Relative Area %	Relative Height %	Amount n.a.
1		11.985	39.083	114.174	92.10	93.39	n.a.
2		14.842	3.354	8.077	7.90	6.61	n.a.
Total:			42.437	122.251	100.00	100.00	

Supplementary Figure 51. HPLC spectrum for compound **(-)-2s**

(+)-1t: IE, *i*-PrOH/hexane = 10/90, v = 1.0 mL/min, λ = 254 nm

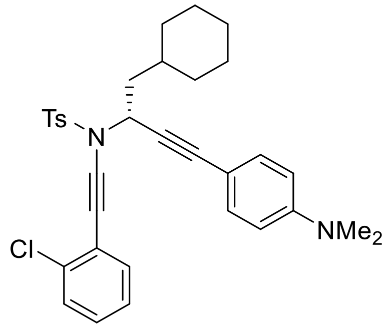

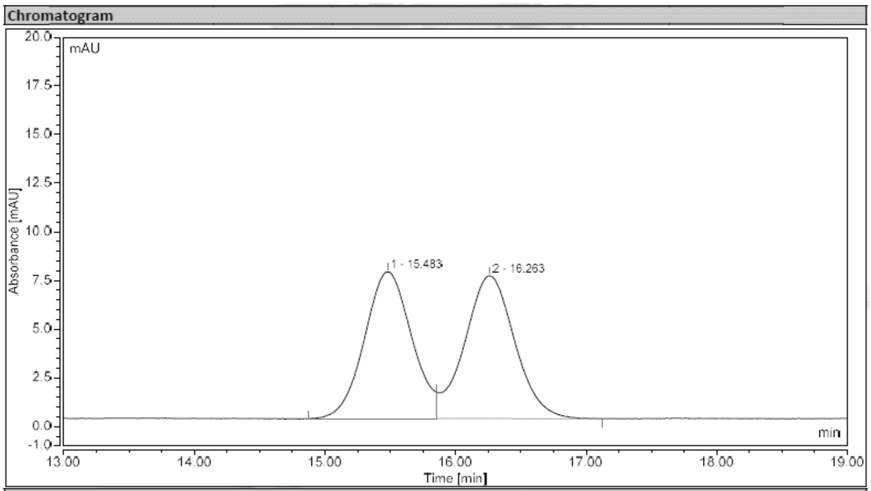

Integration Results

No.	Peak Name	Retention Time min	Area mAU*min	Height mAU	Relative Area %	Relative Height %	Amount n.a.
1		15.483	3.151	7.542	49.14	50.68	n.a.
2		16.263	3.261	7.339	50.86	49.32	n.a.
Total:			6.412	14.881	100.00	100.00	

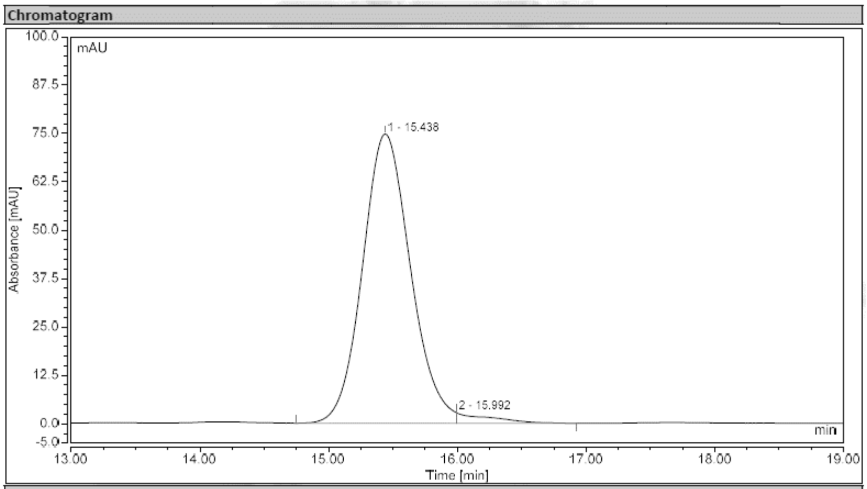

Integration Results

No.	Peak Name	Retention Time min	Area mAU*min	Height mAU	Relative Area %	Relative Height %	Amount n.a.
1		15.438	32.258	74.744	97.56	96.44	n.a.
2		15.992	0.806	2.762	2.44	3.56	n.a.
Total:			33.063	77.507	100.00	100.00	

Supplementary Figure 52. HPLC spectrum for compound **(+)-1t**

(-)-2t: IE, *i*-PrOH/hexane = 10/90, v = 1.0 mL/min, λ = 254 nm

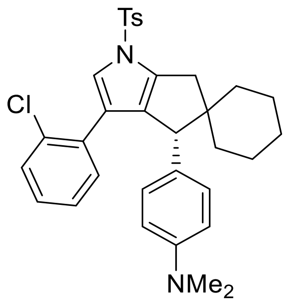

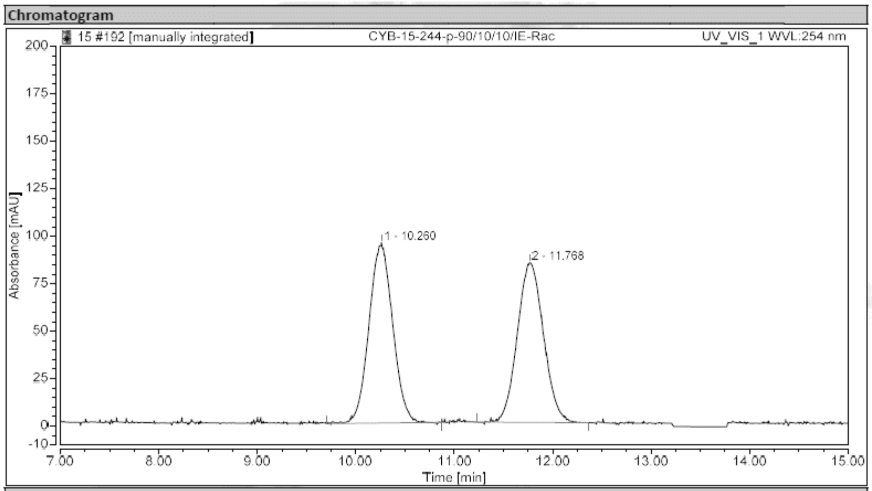

Integration Results

No.	Peak Name	Retention Time min	Area mAU*min	Height mAU	Relative Area %	Relative Height %	Amount n.a.
1		10.260	26.316	95.062	50.42	53.04	n.a.
2		11.768	25.873	84.149	49.58	46.96	n.a.
Total:			52.189	179.210	100.00	100.00	

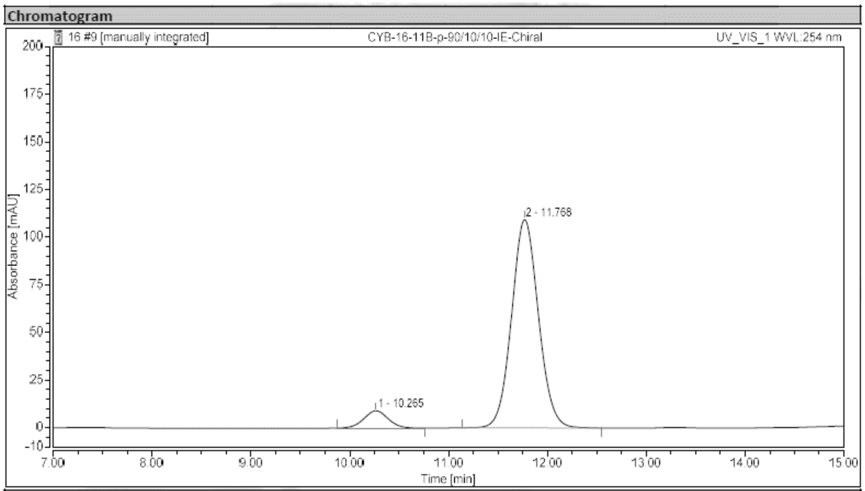

Integration Results

No.	Peak Name	Retention Time min	Area mAU*min	Height mAU	Relative Area %	Relative Height %	Amount n.a.
1		10.265	2.599	9.099	7.04	7.68	n.a.
2		11.768	34.336	109.385	92.96	92.32	n.a.
Total:			36.934	118.484	100.00	100.00	

Supplementary Figure 53. HPLC spectrum for compound **(-)-2t**

(+)-1u: ADH, *i*-PrOH/hexane = 5/95, v = 1.0 mL/min, λ = 254 nm

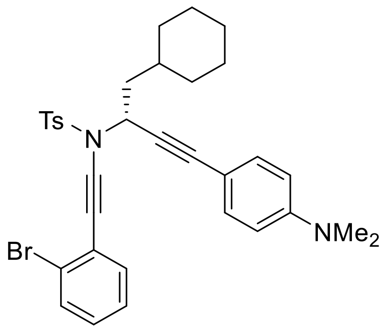

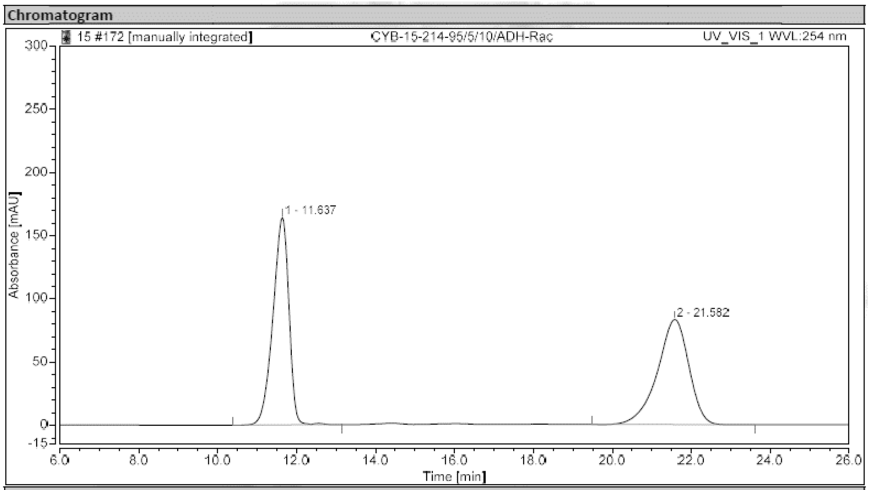

Integration Results

No.	Peak Name	Retention Time min	Area mAU*min	Height mAU	Relative Area %	Relative Height %	Amount n.a.
1		11.637	77.045	164.203	50.17	66.41	n.a.
2		21.582	76.526	83.056	49.83	33.59	n.a.
Total:			153.571	247.259	100.00	100.00	

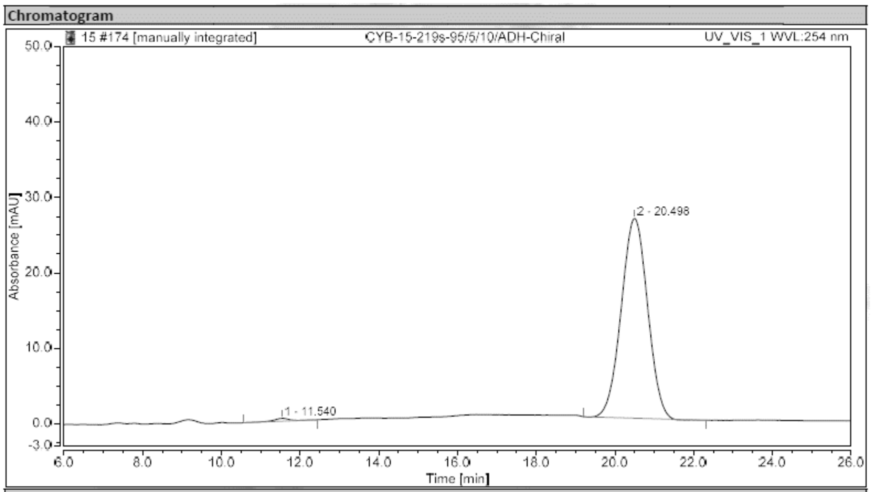

Integration Results

No.	Peak Name	Retention Time min	Area mAU*min	Height mAU	Relative Area %	Relative Height %	Amount n.a.
1		11.540	0.165	0.382	0.80	1.42	n.a.
2		20.498	20.539	26.523	99.20	98.58	n.a.
Total:			20.704	26.905	100.00	100.00	

Supplementary Figure 54. HPLC spectrum for compound **(+)-1u**

(-)-2u: IE, *i*-PrOH/hexane = 10/90, v = 1.0 mL/min, λ = 254 nm

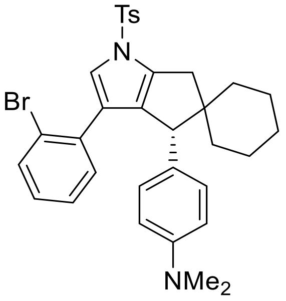

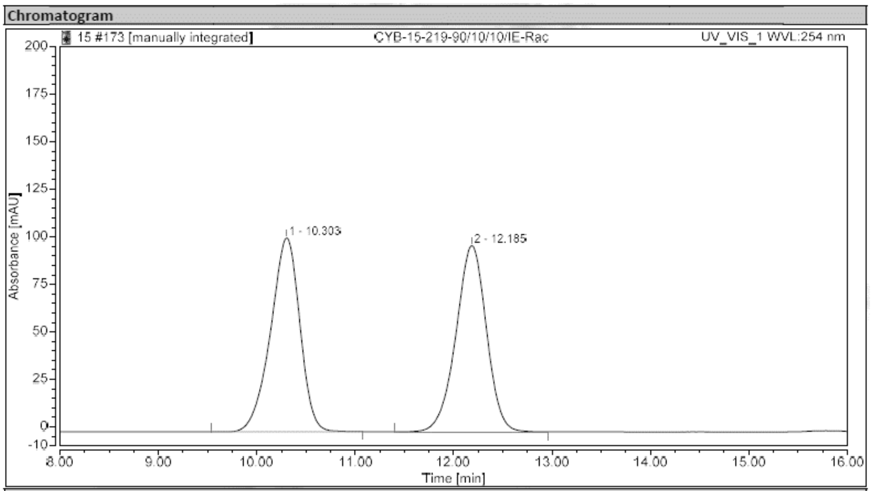

Integration Results

No.	Peak Name	Retention Time min	Area mAU*min	Height mAU	Relative Area %	Relative Height %	Amount n.a.
1		10.303	35.151	101.960	50.00	51.00	n.a.
2		12.185	35.152	97.980	50.00	49.00	n.a.
**Total:**			**70.303**	**199.940**	**100.00**	**100.00**	

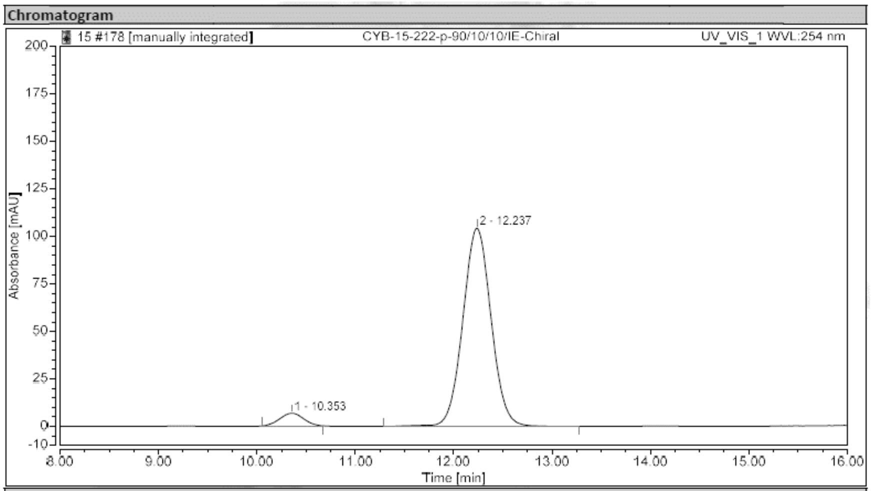

Integration Results

No.	Peak Name	Retention Time min	Area mAU*min	Height mAU	Relative Area %	Relative Height %	Amount n.a.
1		10.353	1.837	6.627	5.03	5.98	n.a.
2		12.237	34.649	104.280	94.97	94.02	n.a.
**Total:**			**36.485**	**110.906**	**100.00**	**100.00**	

Supplementary Figure 55. HPLC spectrum for compound **(-)-2u**

(+)-1v: ADH, *i*-PrOH/hexane = 10/90, v = 1.0 mL/min, λ = 254 nm

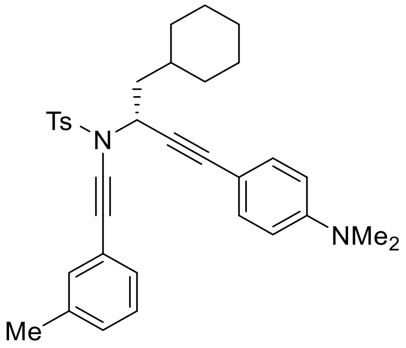

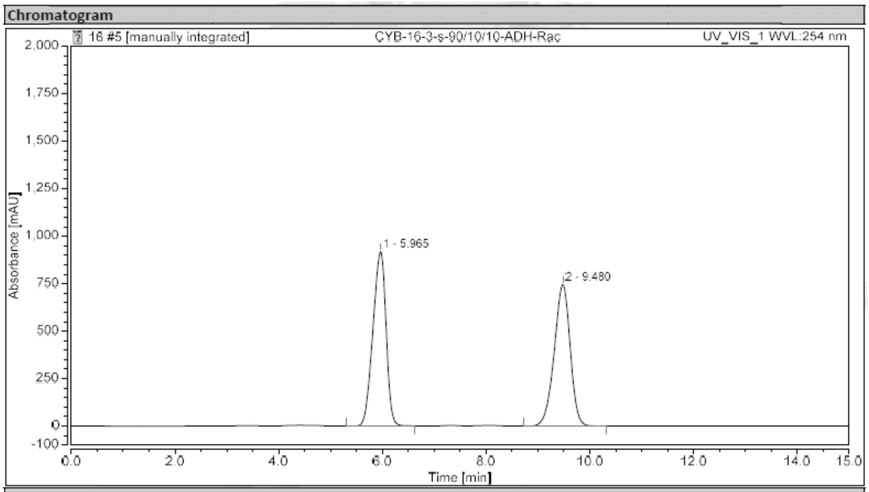

Integration Results

No.	Peak Name	Retention Time min	Area mAU*min	Height mAU	Relative Area %	Relative Height %	Amount n.a.
1		5.965	267.845	919.168	50.17	55.21	n.a.
2		9.480	266.077	745.779	49.83	44.79	n.a.
Total:			533.921	1664.946	100.00	100.00	

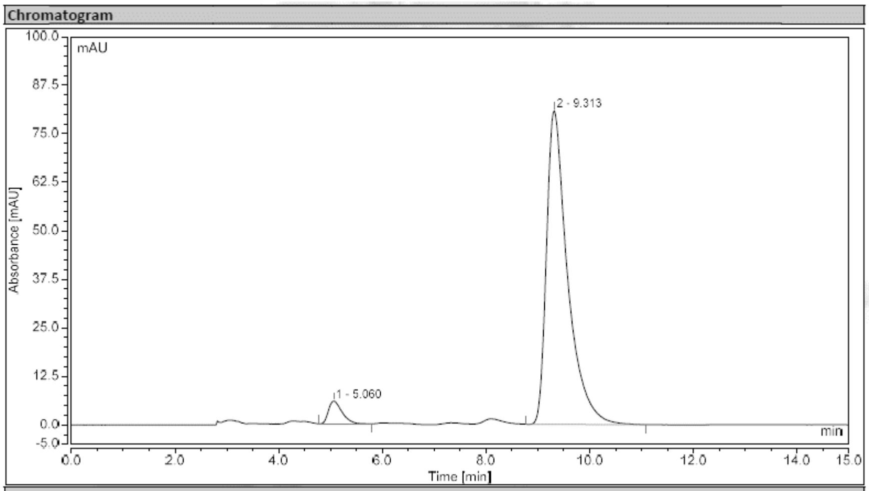

Integration Results

No.	Peak Name	Retention Time min	Area mAU*min	Height mAU	Relative Area %	Relative Height %	Amount n.a.
1		5.060	1.774	5.845	4.45	6.74	n.a.
2		9.313	38.056	80.860	95.55	93.26	n.a.
Total:			39.830	86.705	100.00	100.00	

Supplementary Figure 56. HPLC spectrum for compound **(+)-1v**

(-)-2v: IE, *i*-PrOH/hexane = 10/90, v = 1.0 mL/min, λ = 254 nm

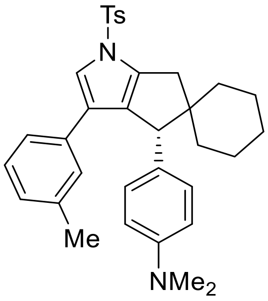

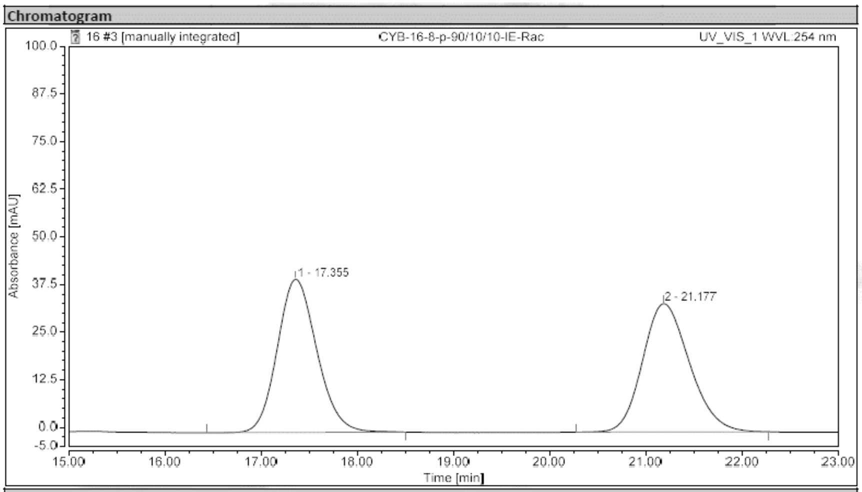

Integration Results

No.	Peak Name	Retention Time min	Area mAU*min	Height mAU	Relative Area %	Relative Height %	Amount n.a.
1		17.355	19.139	40.174	50.13	54.41	n.a.
2		21.177	19.039	33.661	49.87	45.59	n.a.
**Total:**			**38.179**	**73.835**	**100.00**	**100.00**	

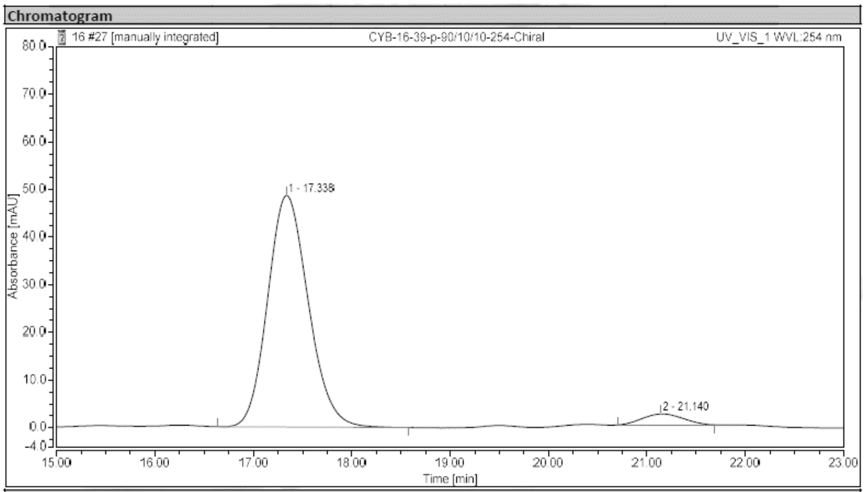

Integration Results

No.	Peak Name	Retention Time min	Area mAU*min	Height mAU	Relative Area %	Relative Height %	Amount n.a.
1		17.338	23.268	48.618	95.49	95.48	n.a.
2		21.140	1.100	2.300	4.51	4.52	n.a.
**Total:**			**24.368**	**50.918**	**100.00**	**100.00**	

Supplementary Figure 57. HPLC spectrum for compound **(-)-2v**

(+)-1w: ADH, *i*-PrOH/hexane = 10/90, v = 1.0 mL/min, λ = 254 nm

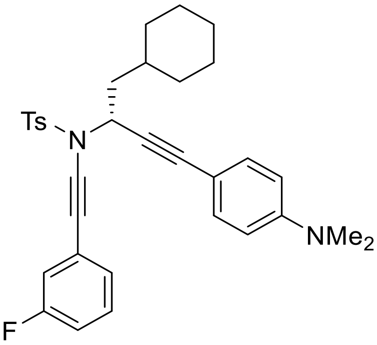

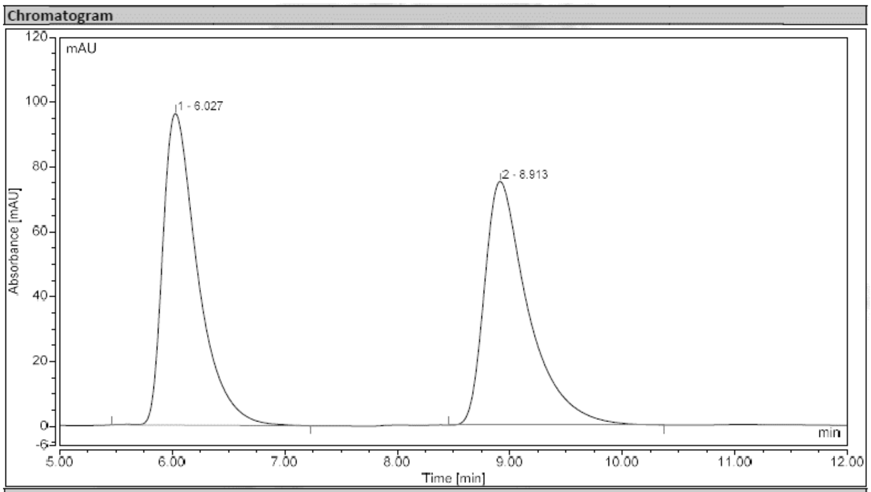

Integration Results

No.	Peak Name	Retention Time min	Area mAU*min	Height mAU	Relative Area %	Relative Height %	Amount n.a.
1		6.027	34.520	96.119	50.49	56.13	n.a.
2		8.913	33.843	75.129	49.51	43.87	n.a.
Total:			68.363	171.247	100.00	100.00	

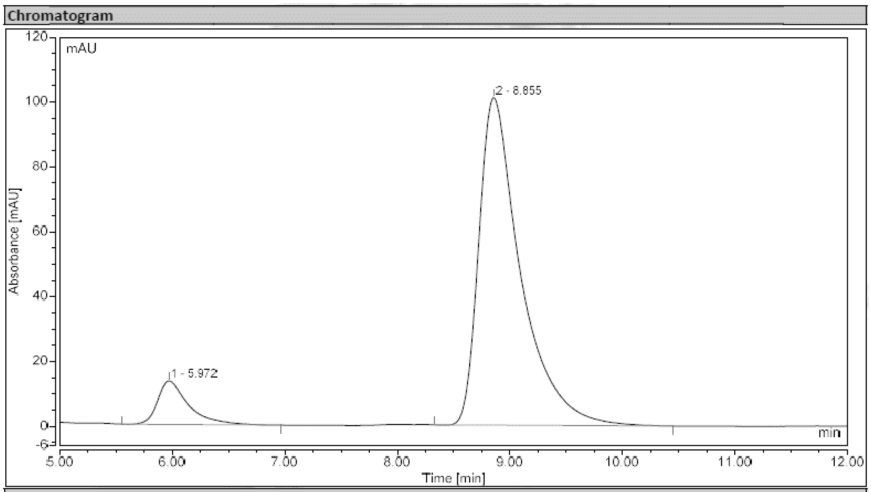

Integration Results

No.	Peak Name	Retention Time min	Area mAU*min	Height mAU	Relative Area %	Relative Height %	Amount n.a.
1		5.972	4.249	13.424	8.93	11.74	n.a.
2		8.855	43.357	100.962	91.07	88.26	n.a.
Total:			47.607	114.386	100.00	100.00	

Supplementary Figure 58. HPLC spectrum for compound **(+)-1w**

(-)-2w: ADH, *i*-PrOH/hexane = 10/90, v = 1.0 mL/min, λ = 254 nm

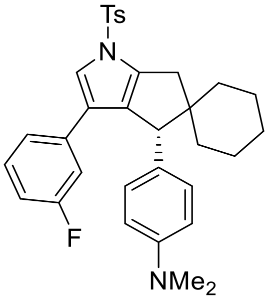

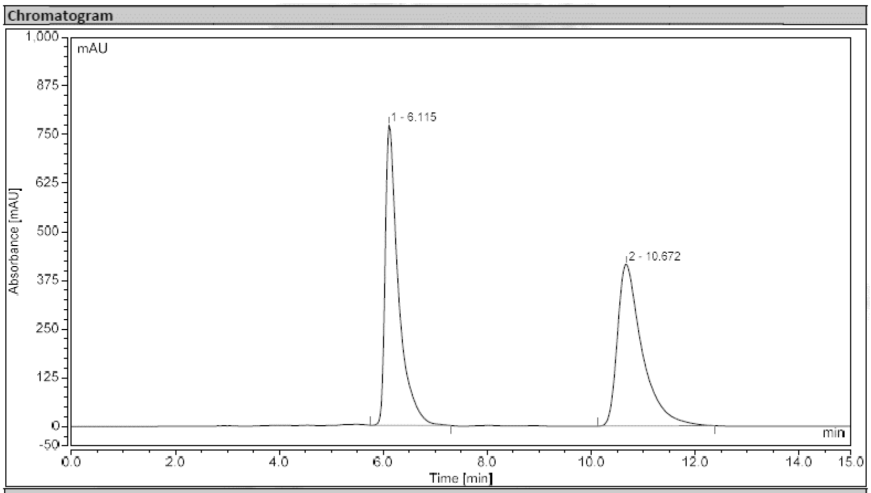

Integration Results

No.	Peak Name	Retention Time min	Area mAU*min	Height mAU	Relative Area %	Relative Height %	Amount n.a.
1		6.115	228.628	772.023	50.01	64.99	n.a.
2		10.672	228.564	415.953	49.99	35.01	n.a.
**Total:**			**457.192**	**1187.976**	**100.00**	**100.00**	

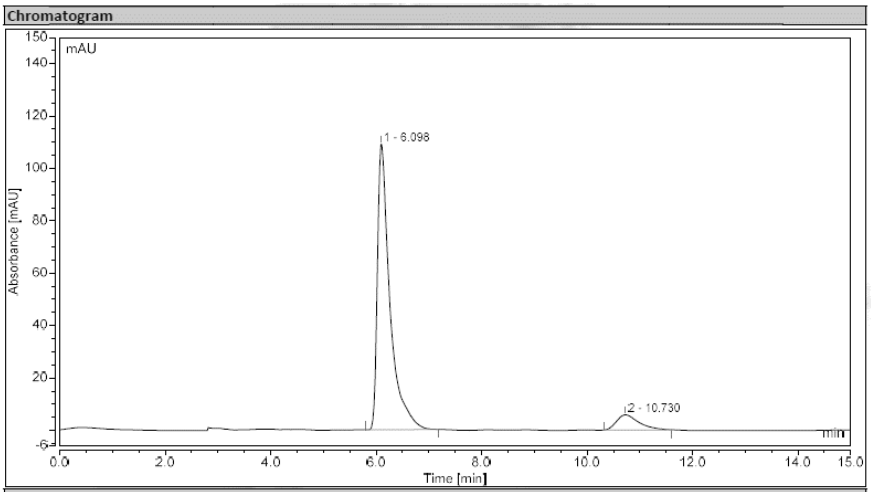

Integration Results

No.	Peak Name	Retention Time min	Area mAU*min	Height mAU	Relative Area %	Relative Height %	Amount n.a.
1		6.098	29.978	109.099	91.39	94.97	n.a.
2		10.730	2.823	5.779	8.61	5.03	n.a.
**Total:**			**32.801**	**114.879**	**100.00**	**100.00**	

Supplementary Figure 59. HPLC spectrum for compound **(-)-2w**

(+)-1x: ADH, *i*-PrOH/hexane = 10/90, v = 1.0 mL/min, λ = 254 nm

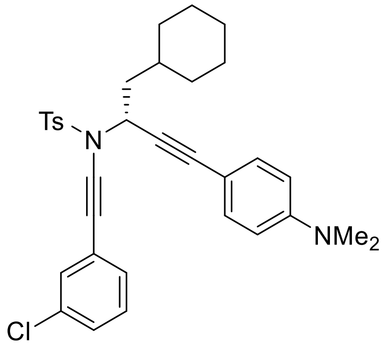

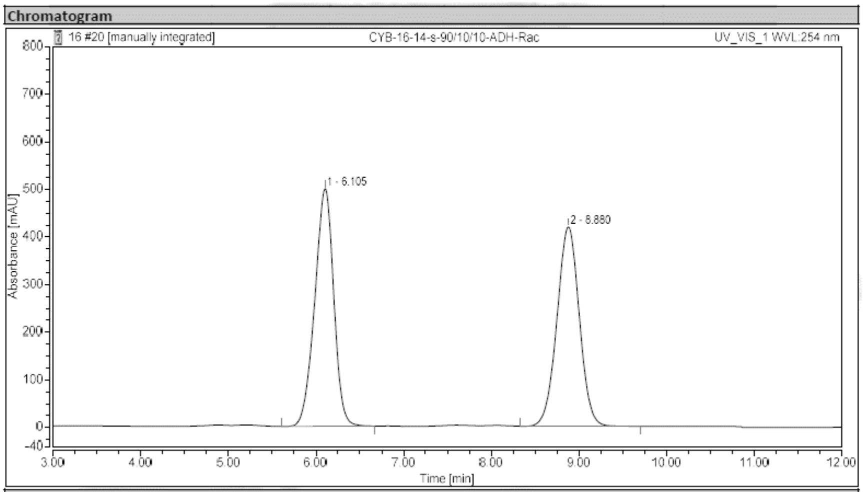

Integration Results

No.	Peak Name	Retention Time min	Area mAU*min	Height mAU	Relative Area %	Relative Height %	Amount n.a.
1		6.105	125.081	499.138	50.24	54.38	n.a.
2		8.880	123.899	418.798	49.76	45.62	n.a.
Total:			248.980	917.936	100.00	100.00	

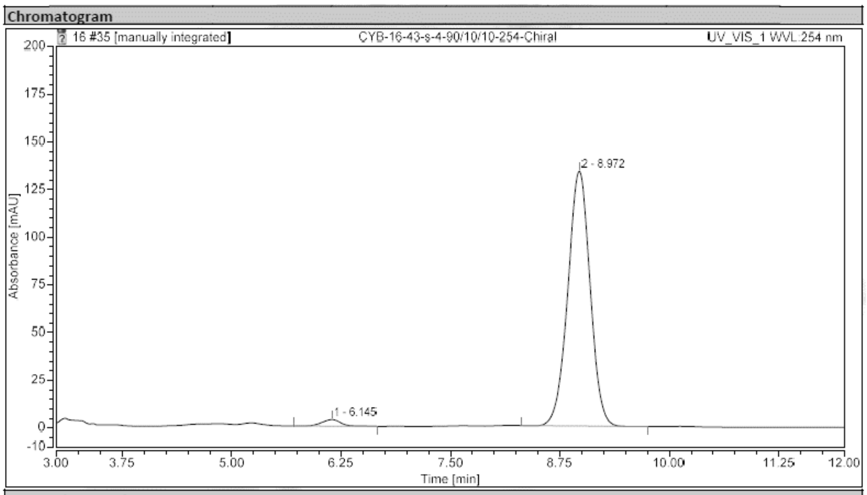

Integration Results

No.	Peak Name	Retention Time min	Area mAU*min	Height mAU	Relative Area %	Relative Height %	Amount n.a.
1		6.145	0.898	3.503	2.23	2.55	n.a.
2		8.972	39.359	133.791	97.77	97.45	n.a.
Total:			40.258	137.294	100.00	100.00	

Supplementary Figure 60. HPLC spectrum for compound **(+)-1x**

(-)-2x: ADH, *i*-PrOH/hexane = 10/90, v = 1.0 mL/min, λ = 254 nm

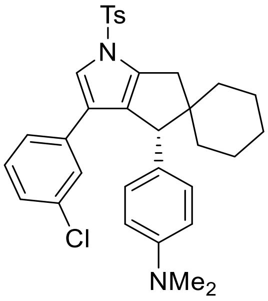

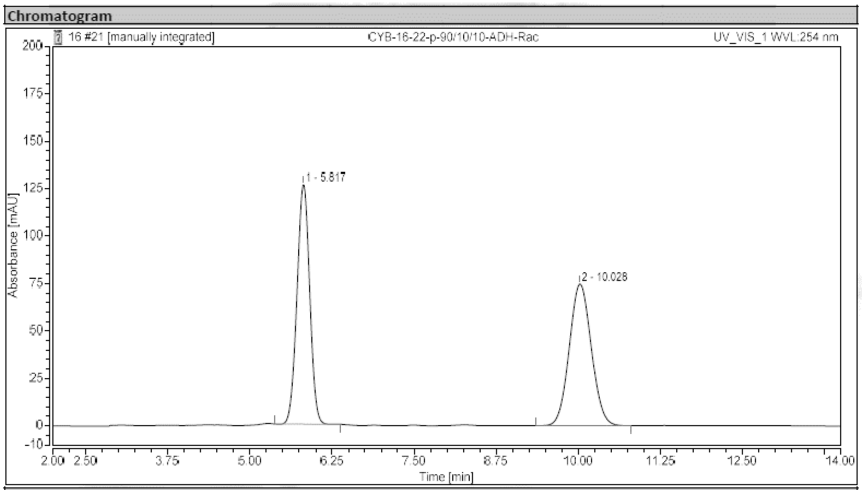

Integration Results

No.	Peak Name	Retention Time min	Area mAU*min	Height mAU	Relative Area %	Relative Height %	Amount n.a.
1		5.817	28.346	126.478	49.49	62.89	n.a.
2		10.028	28.934	74.619	50.51	37.11	n.a.
**Total:**			**57.280**	**201.098**	**100.00**	**100.00**	

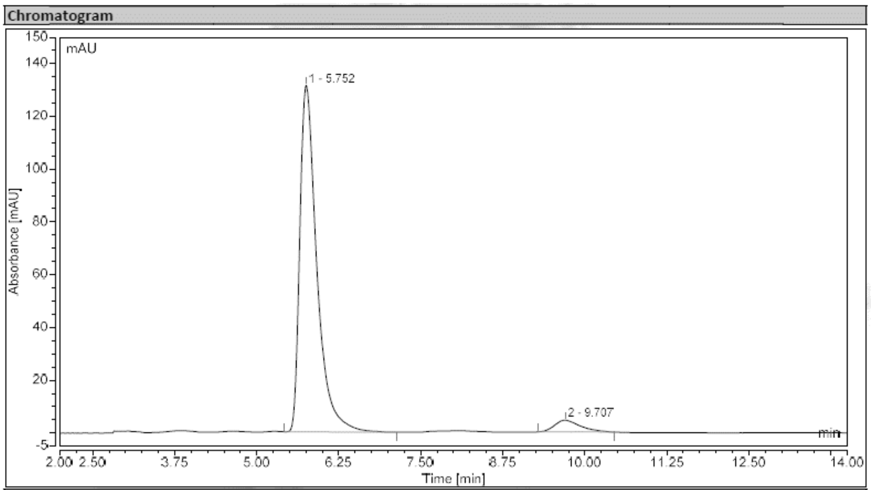

Integration Results

No.	Peak Name	Retention Time min	Area mAU*min	Height mAU	Relative Area %	Relative Height %	Amount n.a.
1		5.752	39.366	131.309	95.15	96.74	n.a.
2		9.707	2.006	4.427	4.85	3.26	n.a.
**Total:**			**41.372**	**135.736**	**100.00**	**100.00**	

Supplementary Figure 61. HPLC spectrum for compound **(-)-2x**

(+)-1y: ADH, *i*-PrOH/hexane = 10/90, v = 1.0 mL/min, λ = 254 nm

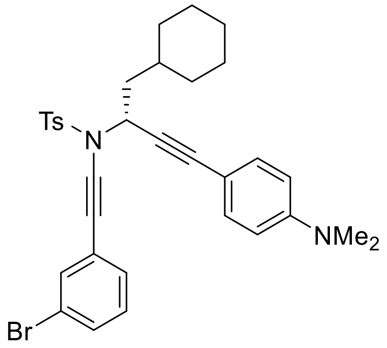

Chromatogram

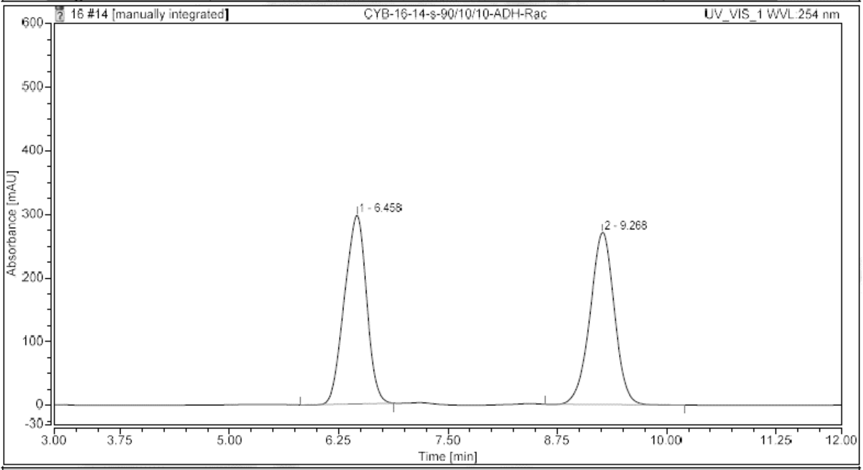

Integration Results

No.	Peak Name	Retention Time min	Area mAU*min	Height mAU	Relative Area %	Relative Height %	Amount n.a.
1		6.458	86.792	296.529	49.91	52.27	n.a.
2		9.268	87.105	270.724	50.09	47.73	n.a.
Total:			173.897	567.253	100.00	100.00	

Chromatogram

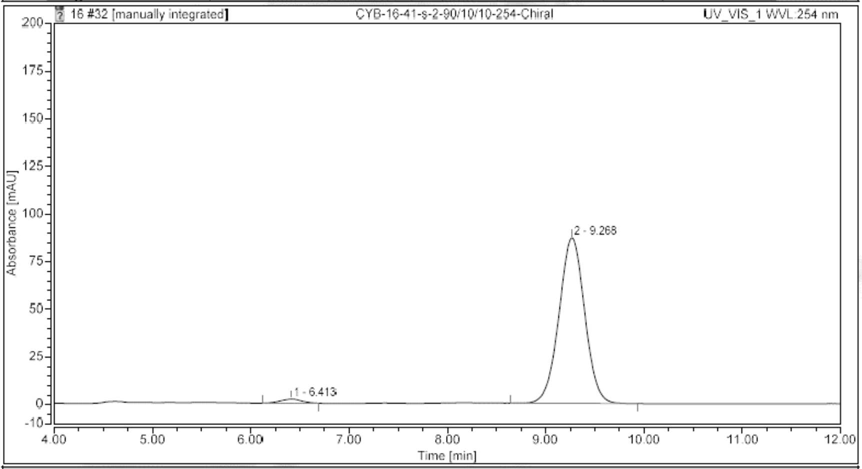

Integration Results

No.	Peak Name	Retention Time min	Area mAU*min	Height mAU	Relative Area %	Relative Height %	Amount n.a.
1		6.413	0.555	2.208	2.06	2.48	n.a.
2		9.268	26.356	86.918	97.94	97.52	n.a.
Total:			26.911	89.126	100.00	100.00	

Supplementary Figure 62. HPLC spectrum for compound **(+)-1y**

(-)-2y: ADH, *i*-PrOH/hexane = 10/90, v = 1.0 mL/min, λ = 254 nm

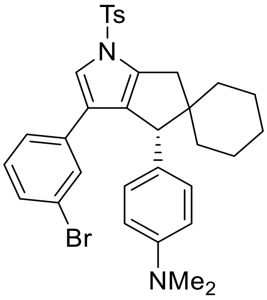

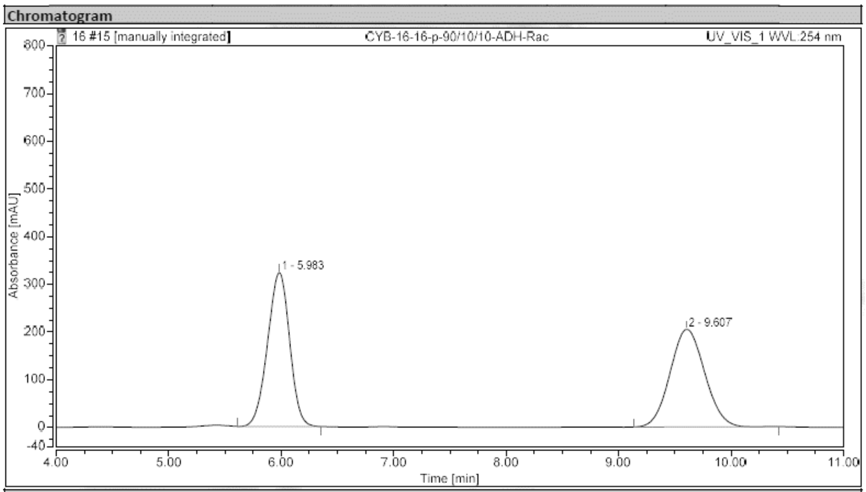

Integration Results

No.	Peak Name	Retention Time min	Area mAU*min	Height mAU	Relative Area %	Relative Height %	Amount n.a.
1		5.983	73.277	322.909	50.15	61.23	n.a.
2		9.607	72.827	204.476	49.85	38.77	n.a.
Total:			146.103	527.385	100.00	100.00	

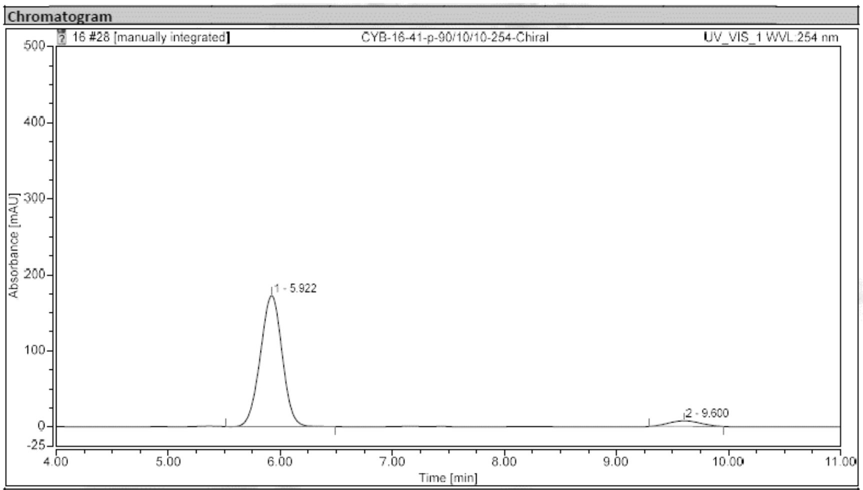

Integration Results

No.	Peak Name	Retention Time min	Area mAU*min	Height mAU	Relative Area %	Relative Height %	Amount n.a.
1		5.922	38.902	172.271	94.08	95.85	n.a.
2		9.600	2.448	7.458	5.92	4.15	n.a.
Total:			41.350	179.729	100.00	100.00	

Supplementary Figure 63. HPLC spectrum for compound **(-)-2y**

(+)-1z: ADH, *i*-PrOH/hexane = 10/90, v = 1.0 mL/min, λ = 254 nm

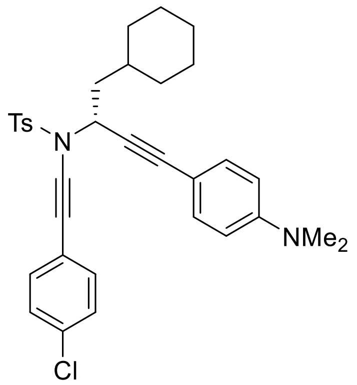

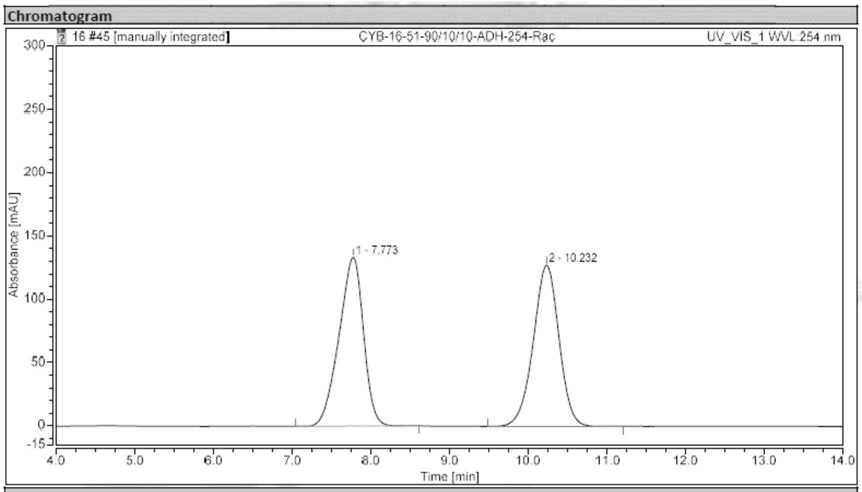

Integration Results							
No.	Peak Name	Retention Time min	Area mAU*min	Height mAU	Relative Area %	Relative Height %	Amount n.a.
1		7.773	48.036	133.387	49.88	51.14	n.a.
2		10.232	48.274	127.449	50.12	48.86	n.a.
Total:			96.310	260.836	100.00	100.00	

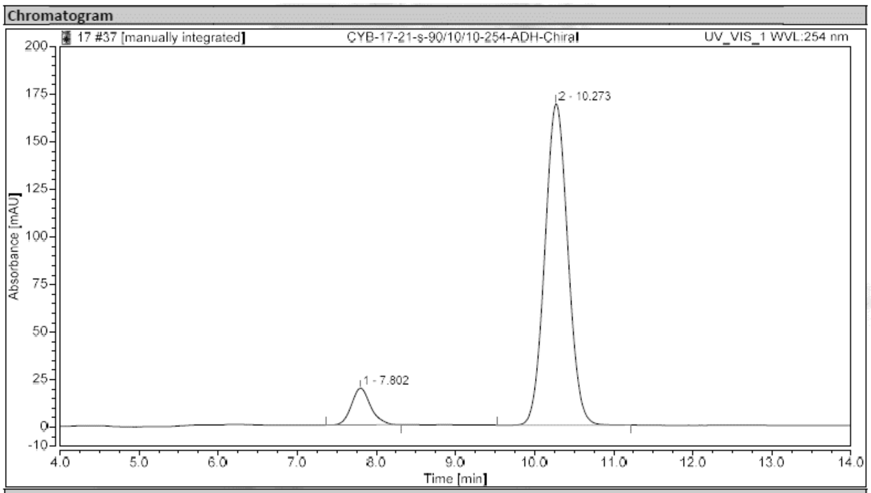

Integration Results							
No.	Peak Name	Retention Time min	Area mAU*min	Height mAU	Relative Area %	Relative Height %	Amount n.a.
1		7.802	5.433	19.327	8.75	10.26	n.a.
2		10.273	56.685	169.060	91.25	89.74	n.a.
Total:			62.118	188.387	100.00	100.00	

Supplementary Figure 64. HPLC spectrum for compound **(+)-1z**

(-)-2z: IG, *i*-PrOH/hexane = 50/50, v = 1.0 mL/min, λ = 254 nm

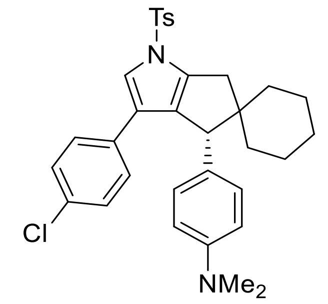

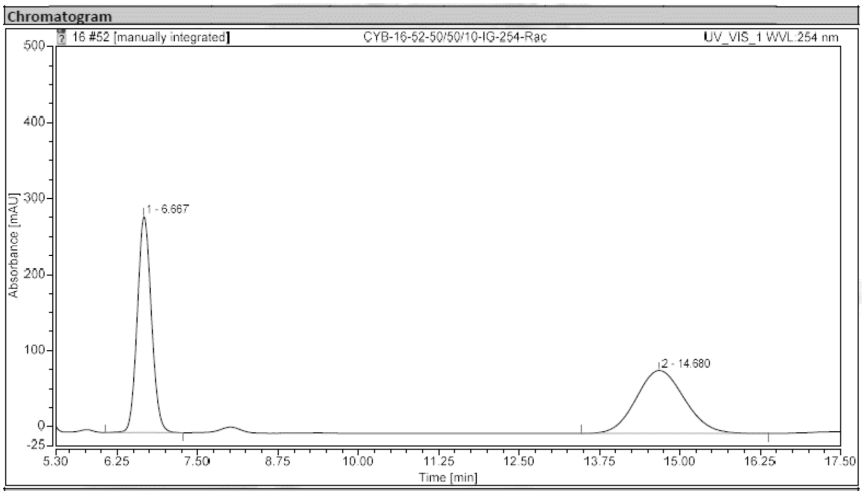

Integration Results

No.	Peak Name	Retention Time min	Area mAU*min	Height mAU	Relative Area %	Relative Height %	Amount n.a.
1		6.667	72.664	284.336	50.23	77.46	n.a.
2		14.680	71.998	82.734	49.77	22.54	n.a.
**Total:**			**144.661**	**367.070**	**100.00**	**100.00**	

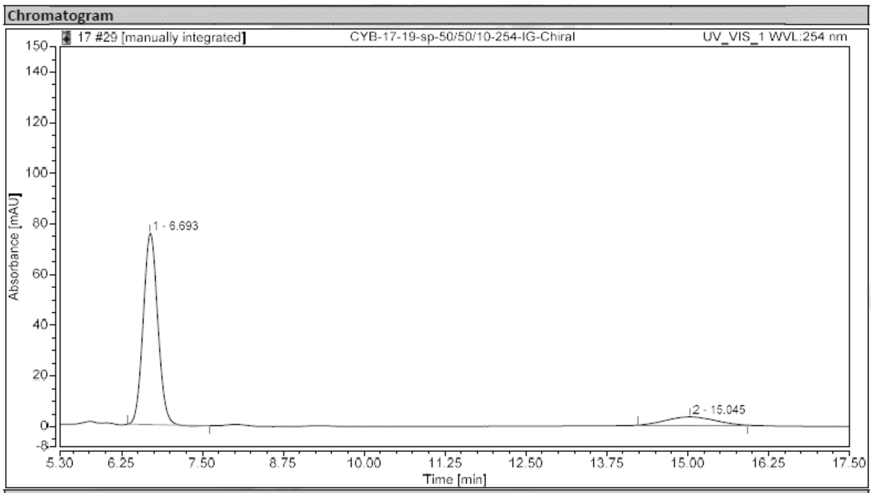

Integration Results

No.	Peak Name	Retention Time min	Area mAU*min	Height mAU	Relative Area %	Relative Height %	Amount n.a.
1		6.693	19.638	75.629	87.59	95.84	n.a.
2		15.045	2.781	3.280	12.41	4.16	n.a.
**Total:**			**22.419**	**78.910**	**100.00**	**100.00**	

Supplementary Figure 65. HPLC spectrum for compound **(-)-2z**

(+)-1aa: ADH, *i*-PrOH/hexane = 10/90, v = 1.0 mL/min, λ = 254 nm

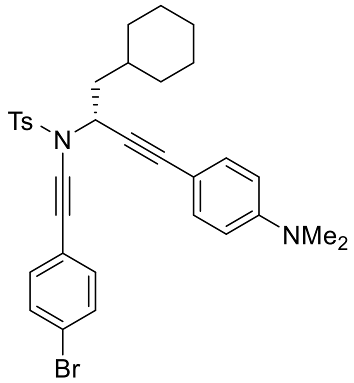

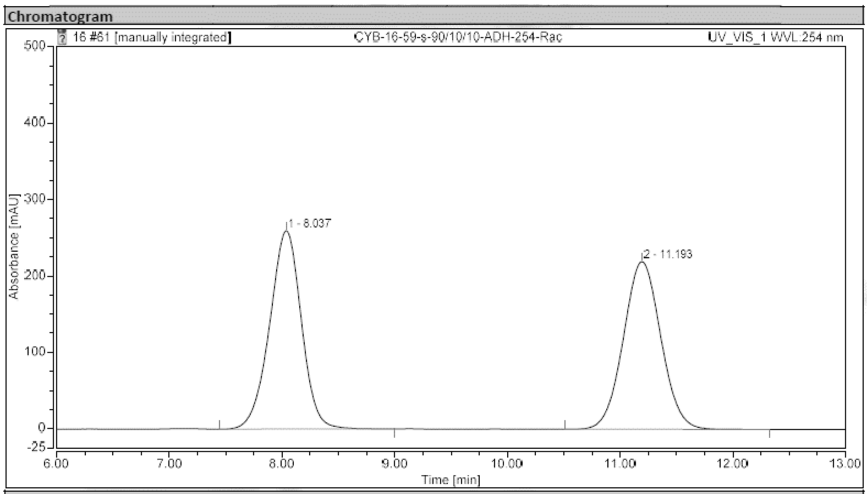

Integration Results

No.	Peak Name	Retention Time min	Area mAU*min	Height mAU	Relative Area %	Relative Height %	Amount n.a.
1		8.037	84.857	259.687	50.06	54.19	n.a.
2		11.193	84.639	219.512	49.94	45.81	n.a.
Total:			**169.495**	**479.199**	**100.00**	**100.00**	

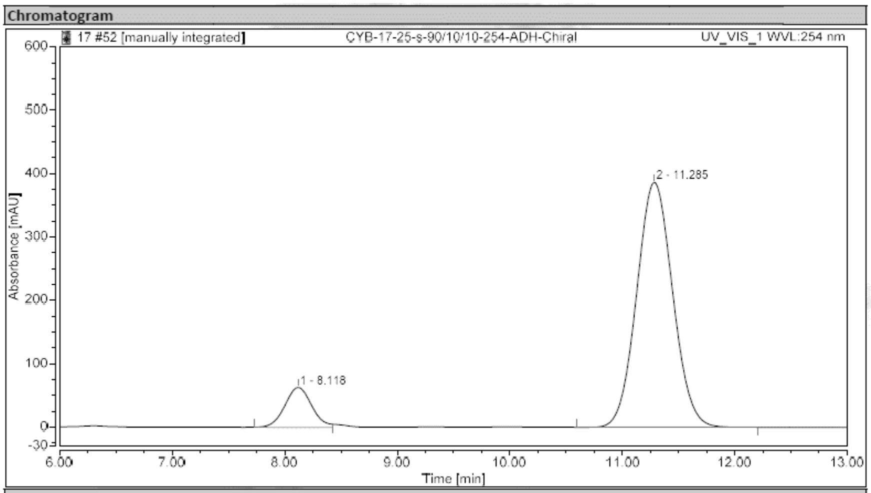

Integration Results

No.	Peak Name	Retention Time min	Area mAU*min	Height mAU	Relative Area %	Relative Height %	Amount n.a.
1		8.118	17.377	62.870	10.82	13.99	n.a.
2		11.285	143.165	386.422	89.18	86.01	n.a.
Total:			**160.543**	**449.293**	**100.00**	**100.00**	

Supplementary Figure 66. HPLC spectrum for compound **(+)-1aa**

(-)-2aa: ADH, *i*-PrOH/hexane = 40/60, v = 1.0 mL/min, λ = 254 nm

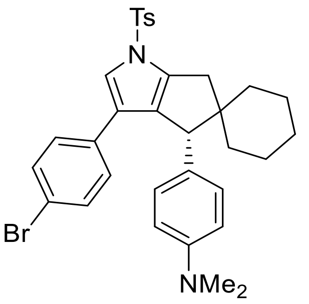

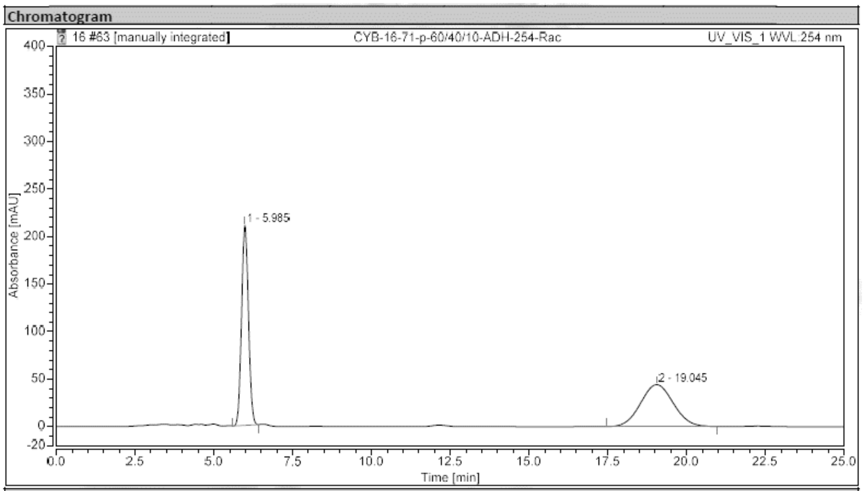

Integration Results

No.	Peak Name	Retention Time min	Area mAU*min	Height mAU	Relative Area %	Relative Height %	Amount n.a.
1		5.985	53.292	210.543	49.97	82.67	n.a.
2		19.045	53.353	44.133	50.03	17.33	n.a.
Total:			106.645	254.676	100.00	100.00	

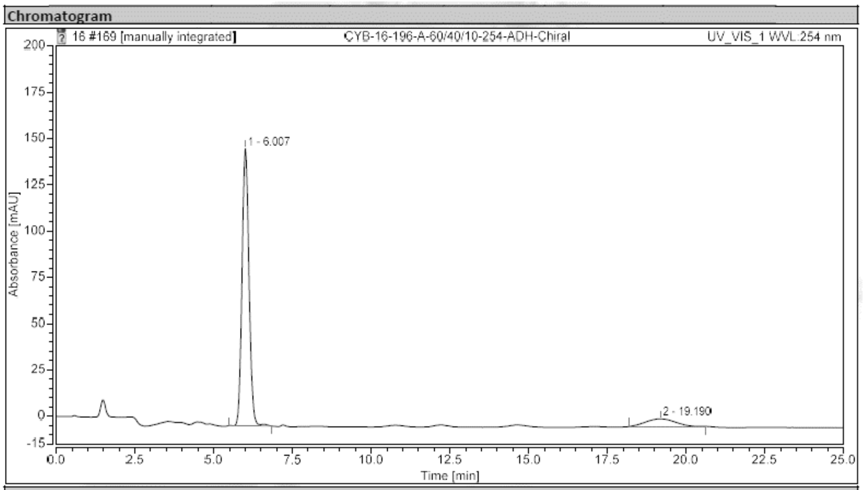

Integration Results

No.	Peak Name	Retention Time min	Area mAU*min	Height mAU	Relative Area %	Relative Height %	Amount n.a.
1		6.007	38.374	149.865	89.83	97.35	n.a.
2		19.190	4.343	4.080	10.17	2.65	n.a.
Total:			42.717	153.944	100.00	100.00	

Supplementary Figure 67. HPLC spectrum for compound **(-)-2aa**

(+)-1ab: ADH, *i*-PrOH/hexane = 10/90, v = 1.0 mL/min, λ = 254 nm

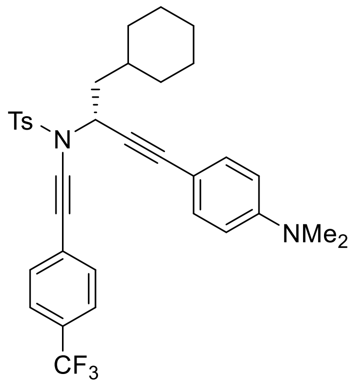

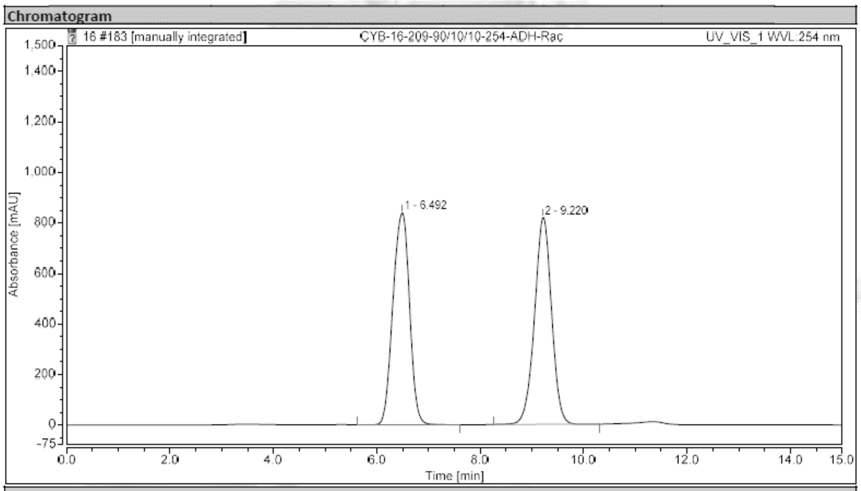

Integration Results

No.	Peak Name	Retention Time min	Area mAU*min	Height mAU	Relative Area %	Relative Height %	Amount n.a.
1		6.492	311.066	839.723	49.91	50.61	n.a.
2		9.220	312.241	819.338	50.09	49.39	n.a.
Total:			623.307	1659.061	100.00	100.00	

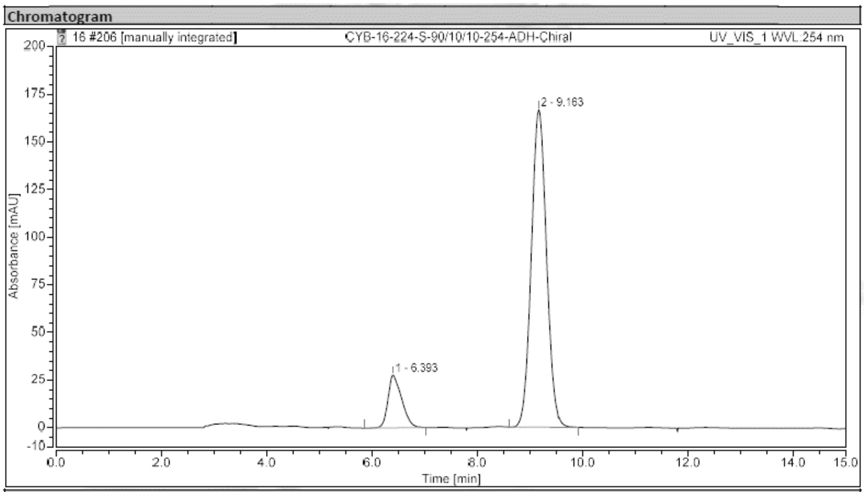

Integration Results

No.	Peak Name	Retention Time min	Area mAU*min	Height mAU	Relative Area %	Relative Height %	Amount n.a.
1		6.393	8.276	27.763	12.92	14.28	n.a.
2		9.163	55.782	166.669	87.08	85.72	n.a.
Total:			64.058	194.433	100.00	100.00	

Supplementary Figure 68. HPLC spectrum for compound **(+)-1ab**

(-)-2ab: ADH, *i*-PrOH/hexane = 40/60, v = 1.0 mL/min, λ = 254 nm

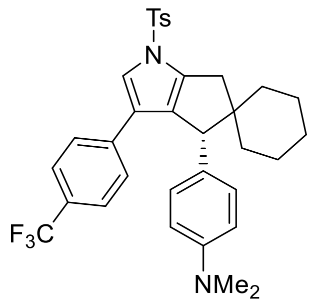

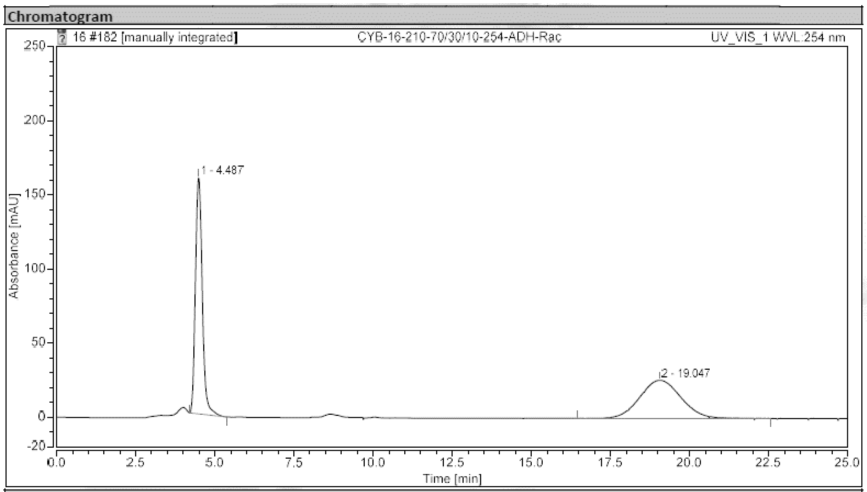

Integration Results

No.	Peak Name	Retention Time min	Area mAU*min	Height mAU	Relative Area %	Relative Height %	Amount n.a.
1		4.487	39.766	159.393	50.46	86.06	n.a.
2		19.047	39.044	25.810	49.54	13.94	n.a.
**Total:**			**78.810**	**185.203**	**100.00**	**100.00**	

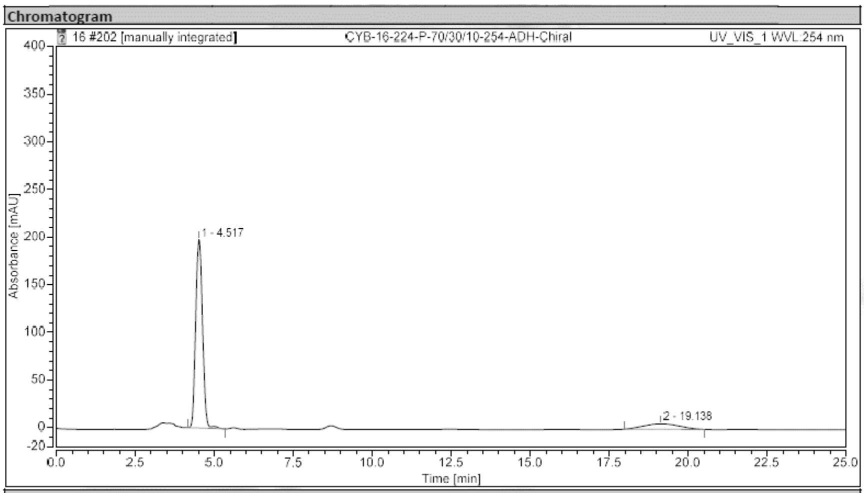

Integration Results

No.	Peak Name	Retention Time min	Area mAU*min	Height mAU	Relative Area %	Relative Height %	Amount n.a.
1		4.517	49.330	197.880	87.02	97.21	n.a.
2		19.138	7.357	5.674	12.98	2.79	n.a.
**Total:**			**56.687**	**203.554**	**100.00**	**100.00**	

Supplementary Figure 69. HPLC spectrum for compound **(-)-2ab**

(+)-1ac: IE, *i*-PrOH/hexane = 10/90, v = 1.0 mL/min, λ = 254 nm

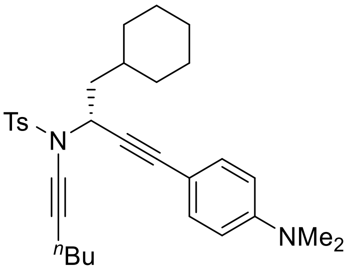

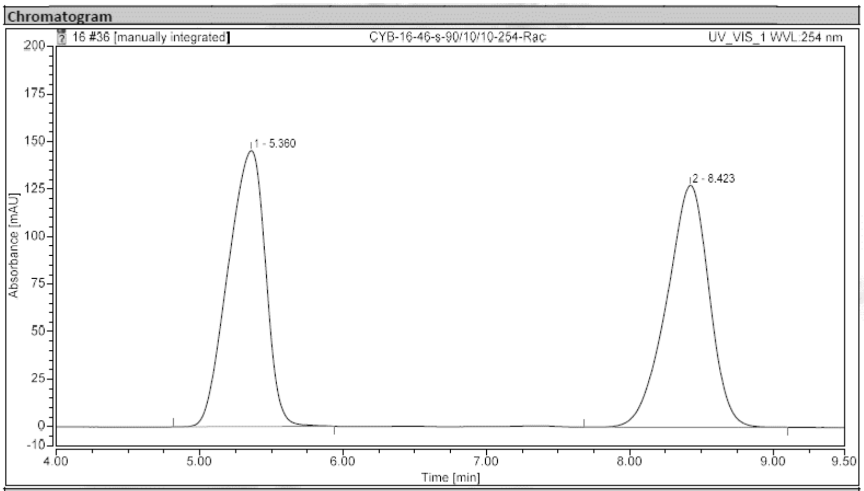

Integration Results

No.	Peak Name	Retention Time min	Area mAU*min	Height mAU	Relative Area %	Relative Height %	Amount n.a.
1		5.360	43.913	145.273	50.03	53.29	n.a.
2		8.423	43.864	127.348	49.97	46.71	n.a.
Total:			87.777	272.622	100.00	100.00	

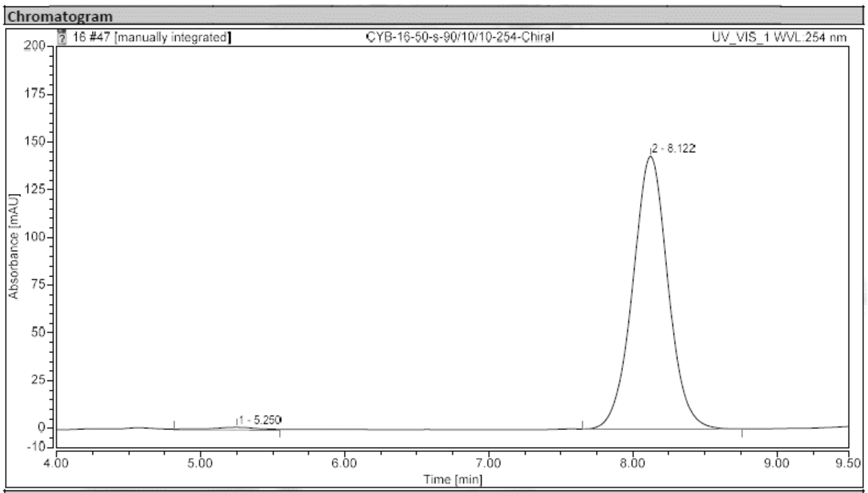

Integration Results

No.	Peak Name	Retention Time min	Area mAU*min	Height mAU	Relative Area %	Relative Height %	Amount n.a.
1		5.250	0.631	1.492	1.53	1.03	n.a.
2		8.122	40.586	142.993	98.47	98.97	n.a.
Total:			41.217	144.485	100.00	100.00	

Supplementary Figure 70. HPLC spectrum for compound **(+)-1ac**

(-)-2ac: IE, *i*-PrOH/hexane = 2/98, v = 1.0 mL/min, λ = 254 nm

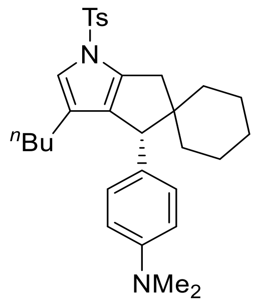

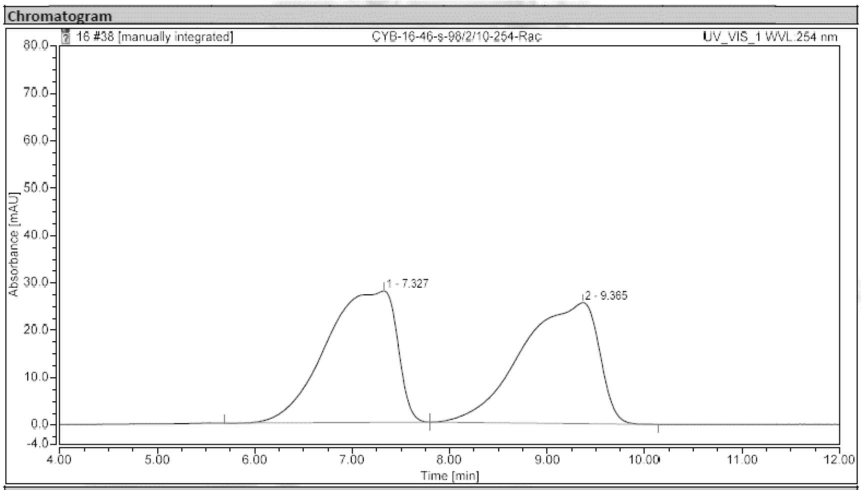

Integration Results

No.	Peak Name	Retention Time min	Area mAU*min	Height mAU	Relative Area %	Relative Height %	Amount n.a.
1		7.327	23.331	27.752	50.05	52.06	n.a.
2		9.365	23.283	25.554	49.95	47.94	n.a.
**Total:**			**46.614**	**53.307**	**100.00**	**100.00**	

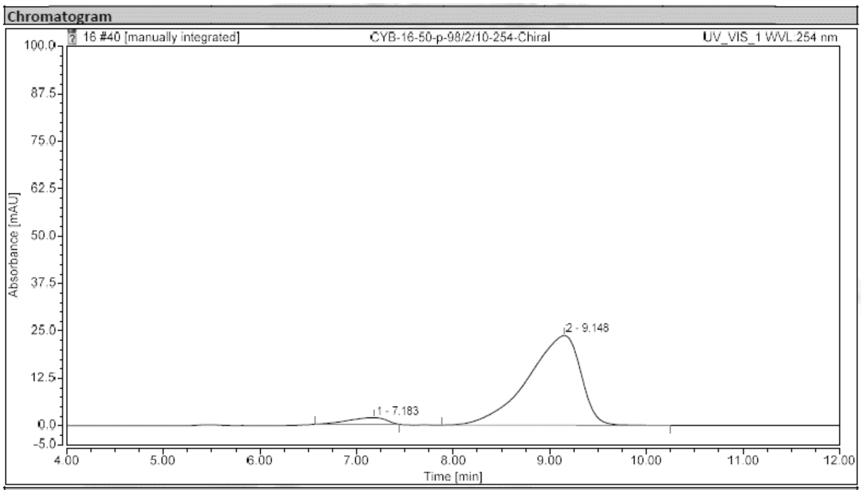

Integration Results

No.	Peak Name	Retention Time min	Area mAU*min	Height mAU	Relative Area %	Relative Height %	Amount n.a.
1		7.183	0.794	1.756	5.02	6.91	n.a.
2		9.148	15.023	23.654	94.98	93.09	n.a.
**Total:**			**15.817**	**25.410**	**100.00**	**100.00**	

Supplementary Figure 71. HPLC spectrum for compound **(-)-2ac**

(+)-1af: IE, *i*-PrOH/hexane = 3/97, v = 1.0 mL/min, λ = 254 nm

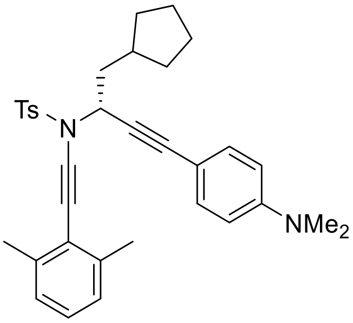

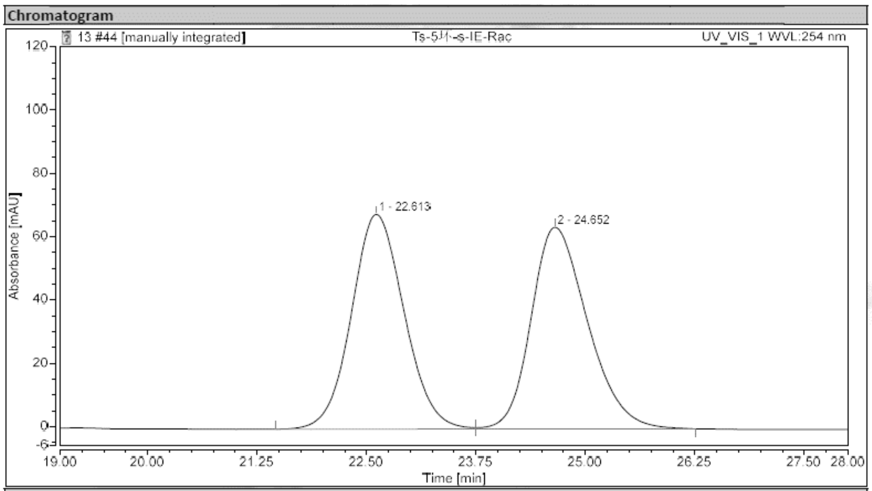

Integration Results

No.	Peak Name	Retention Time min	Area mAU*min	Height mAU	Relative Area %	Relative Height %	Amount n.a.
1		22.613	47.268	67.782	49.90	51.57	n.a.
2		24.652	47.467	63.650	50.10	48.43	n.a.
Total:			94.736	131.432	100.00	100.00	

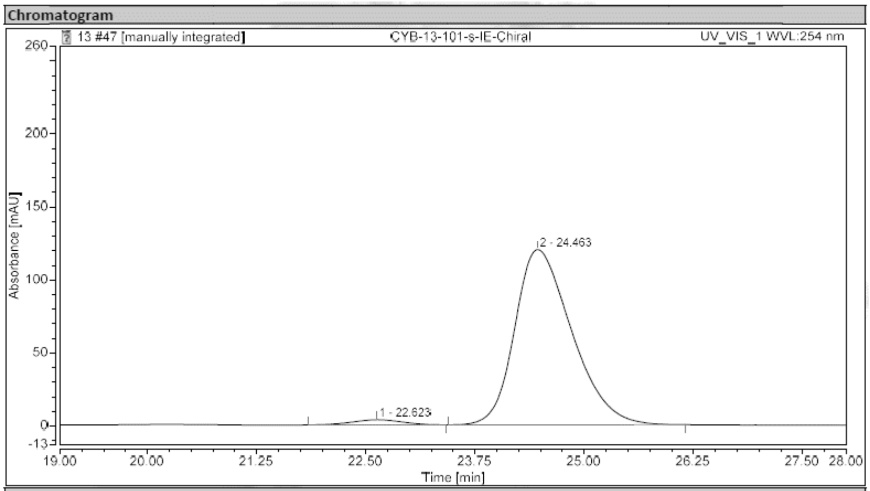

Integration Results

No.	Peak Name	Retention Time min	Area mAU*min	Height mAU	Relative Area %	Relative Height %	Amount n.a.
1		22.623	2.302	3.476	2.47	2.81	n.a.
2		24.463	90.876	120.025	97.53	97.19	n.a.
Total:			93.178	123.500	100.00	100.00	

Supplementary Figure 72. HPLC spectrum for compound **(+)-1af**

(-)-2af: IE, *i*-PrOH/hexane = 3/97, v = 1.0 mL/min, λ = 254 nm

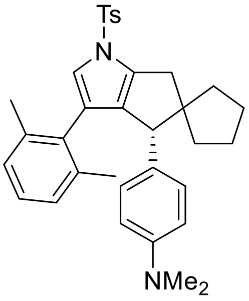

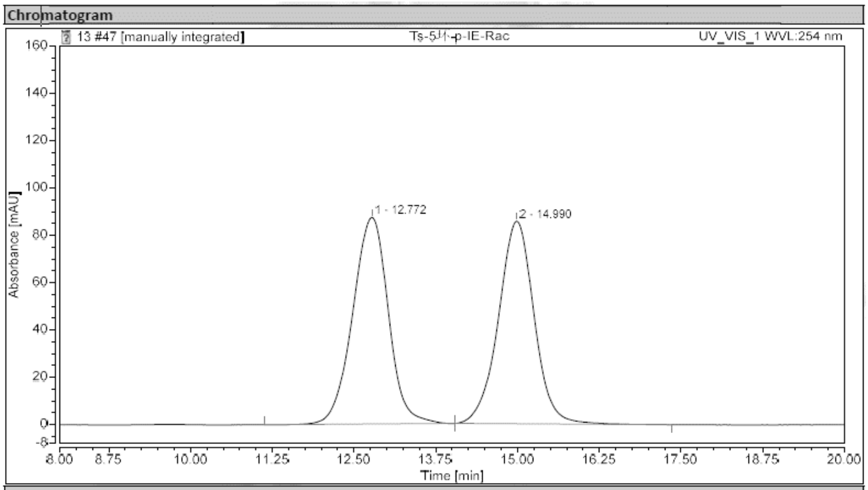

Integration Results

No.	Peak Name	Retention Time min	Area mAU*min	Height mAU	Relative Area %	Relative Height %	Amount n.a.
1		12.772	55.210	87.343	50.51	50.48	n.a.
2		14.990	54.087	85.672	49.49	49.52	n.a.
Total:			109.297	173.015	100.00	100.00	

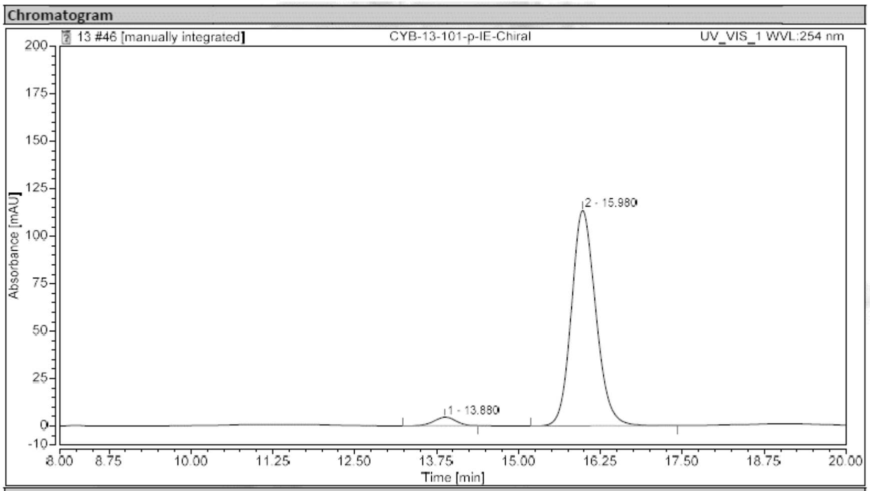

Integration Results

No.	Peak Name	Retention Time min	Area mAU*min	Height mAU	Relative Area %	Relative Height %	Amount n.a.
1		13.880	1.766	4.517	3.48	3.83	n.a.
2		15.980	49.008	113.500	96.52	96.17	n.a.
Total:			50.773	118.017	100.00	100.00	

Supplementary Figure 73. HPLC spectrum for compound **(-)-2af**

(+)-1ag: IE, *i*-PrOH/hexane = 10/90, v = 1.0 mL/min, λ = 254 nm

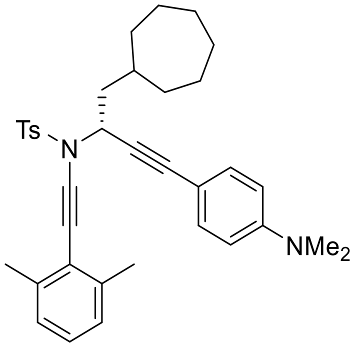

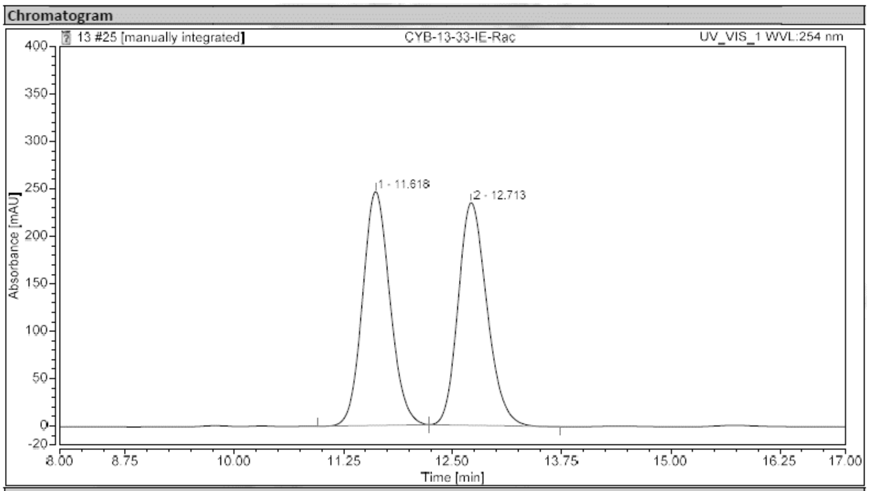

Integration Results

No.	Peak Name	Retention Time min	Area mAU*min	Height mAU	Relative Area %	Relative Height %	Amount n.a.
1		11.618	89.441	246.480	49.93	51.19	n.a.
2		12.713	89.680	235.021	50.07	48.81	n.a.
Total:			179.121	481.501	100.00	100.00	

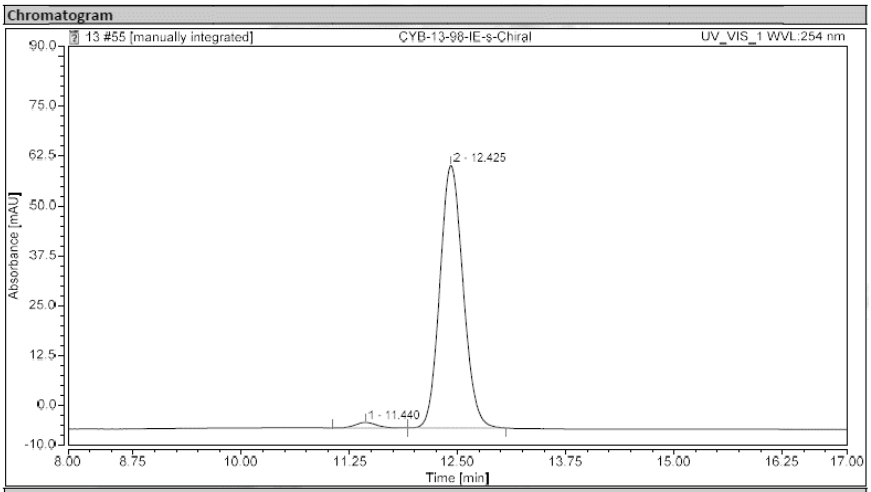

Integration Results

No.	Peak Name	Retention Time min	Area mAU*min	Height mAU	Relative Area %	Relative Height %	Amount n.a.
1		11.440	0.396	1.387	1.90	2.06	n.a.
2		12.425	20.448	65.847	98.10	97.94	n.a.
Total:			20.844	67.234	100.00	100.00	

Supplementary Figure 74. HPLC spectrum for compound **(+)-1ag**

(-)-2ag: IE, *i*-PrOH/hexane = 2/98, v = 1.0 mL/min, λ = 254 nm

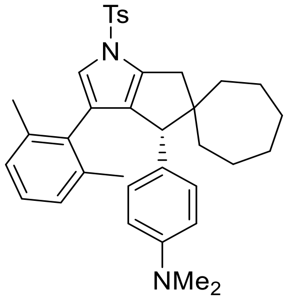

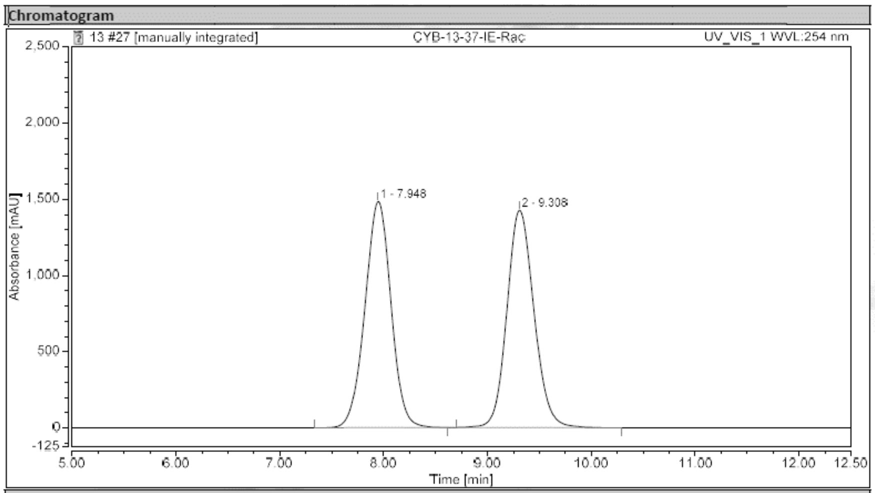

Integration Results

No.	Peak Name	Retention Time min	Area mAU*min	Height mAU	Relative Area %	Relative Height %	Amount n.a.
1		7.948	431.316	1484.606	50.06	51.01	n.a.
2		9.308	430.362	1425.697	49.94	48.99	n.a.
Total:			861.678	2910.303	100.00	100.00	

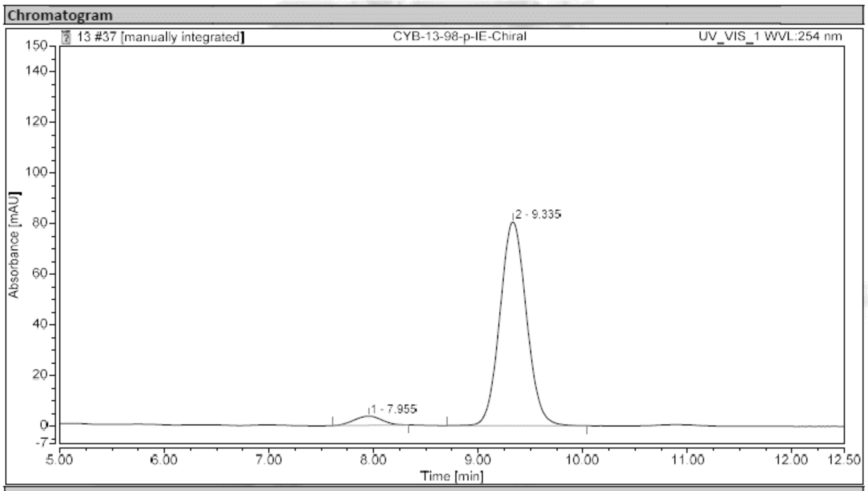

Integration Results

No.	Peak Name	Retention Time min	Area mAU*min	Height mAU	Relative Area %	Relative Height %	Amount n.a.
1		7.955	1.116	3.603	4.51	4.29	n.a.
2		9.335	23.611	80.430	95.49	95.71	n.a.
Total:			24.727	84.033	100.00	100.00	

Supplementary Figure 75. HPLC spectrum for compound **(-)-2ag**

(+)-1ah: IE, *i*-PrOH/hexane = 10/90, v = 1.0 mL/min, λ = 254 nm

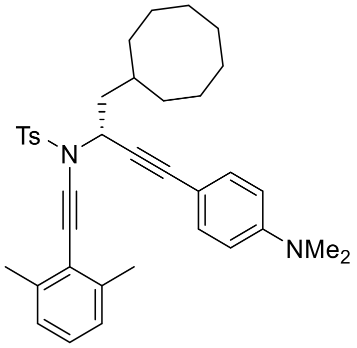

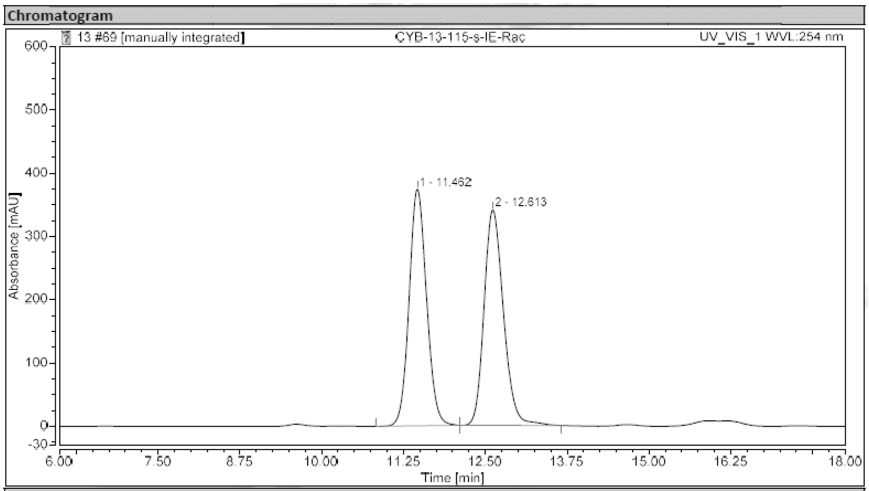

Integration Results

No.	Peak Name	Retention Time min	Area mAU*min	Height mAU	Relative Area %	Relative Height %	Amount n.a.
1		11.462	115.638	374.184	49.74	52.34	n.a.
2		12.613	116.849	340.746	50.26	47.66	n.a.
Total:			232.488	714.929	100.00	100.00	

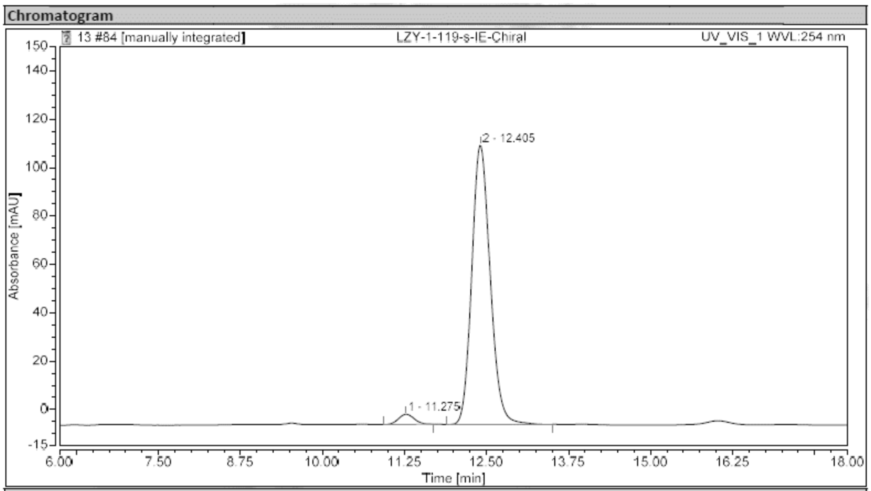

Integration Results

No.	Peak Name	Retention Time min	Area mAU*min	Height mAU	Relative Area %	Relative Height %	Amount n.a.
1		11.275	1.163	4.201	3.00	3.51	n.a.
2		12.405	37.588	115.530	97.00	96.49	n.a.
Total:			38.750	119.732	100.00	100.00	

Supplementary Figure 76. HPLC spectrum for compound **(+)-1ah**

(-)-2ah: IE, *i*-PrOH/hexane = 10/90, v = 1.0 mL/min, λ = 254 nm

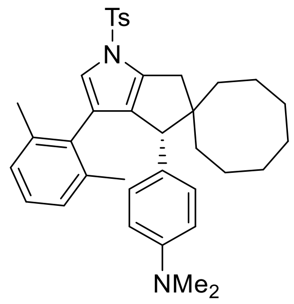

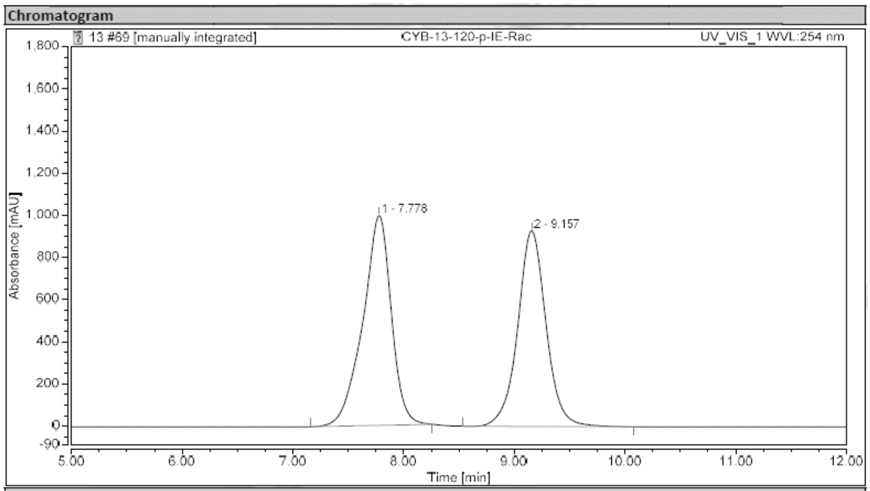

Integration Results

No.	Peak Name	Retention Time min	Area mAU*min	Height mAU	Relative Area %	Relative Height %	Amount n.a.
1		7.778	296.000	995.940	51.83	51.75	n.a.
2		9.157	275.108	928.572	48.17	48.25	n.a.
**Total:**			**571.108**	**1924.512**	**100.00**	**100.00**	

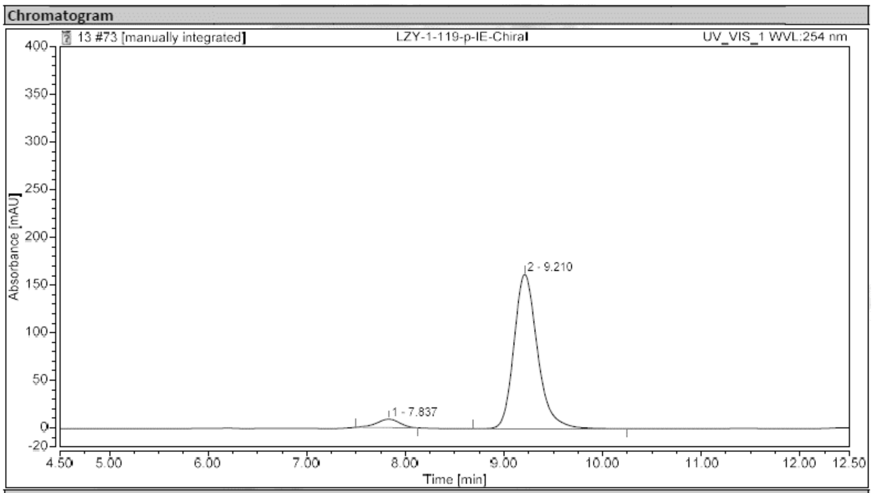

Integration Results

No.	Peak Name	Retention Time min	Area mAU*min	Height mAU	Relative Area %	Relative Height %	Amount n.a.
1		7.837	2.386	8.837	5.08	5.18	n.a.
2		9.210	44.576	161.918	94.92	94.82	n.a.
**Total:**			**46.962**	**170.754**	**100.00**	**100.00**	

Supplementary Figure 77. HPLC spectrum for compound **(-)-2ah**

(+)-1ai: IE, *i*-PrOH/hexane = 10/90, v = 1.0 mL/min, λ = 254 nm

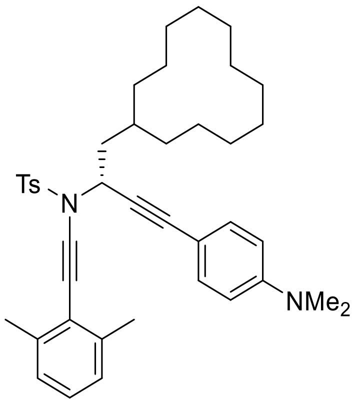

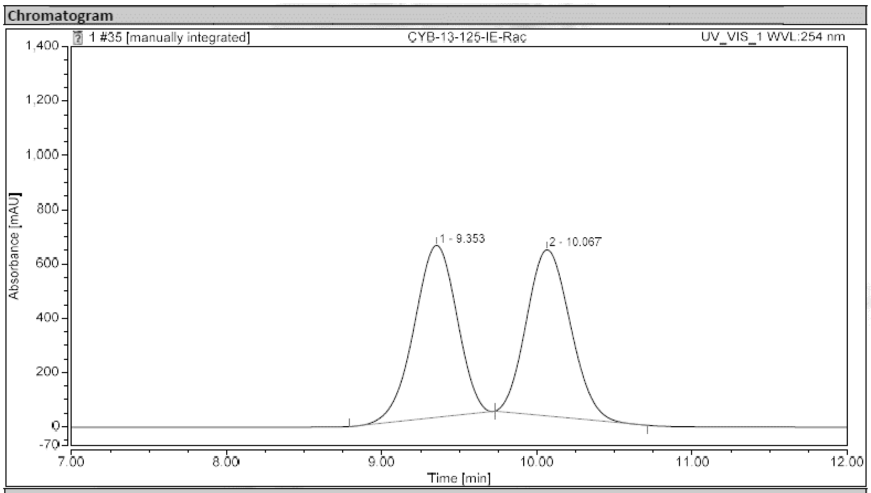

Integration Results

No.	Peak Name	Retention Time min	Area mAU*min	Height mAU	Relative Area %	Relative Height %	Amount n.a.
1		9.353	202.332	633.917	50.18	50.86	n.a.
2		10.067	200.858	612.601	49.82	49.14	n.a.
Total:			403.190	1246.517	100.00	100.00	

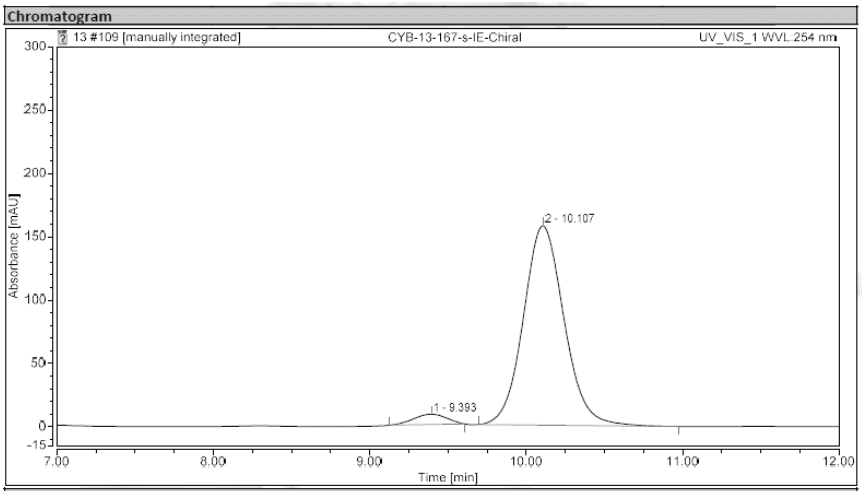

Integration Results

No.	Peak Name	Retention Time min	Area mAU*min	Height mAU	Relative Area %	Relative Height %	Amount n.a.
1		9.393	1.996	8.160	4.04	4.93	n.a.
2		10.107	47.420	157.368	95.96	95.07	n.a.
Total:			49.416	165.528	100.00	100.00	

Supplementary Figure 78. HPLC spectrum for compound **(+)-1ai**

(-)-2ai: IE, *i*-PrOH/hexane = 10/90, v = 1.0 mL/min, λ = 254 nm

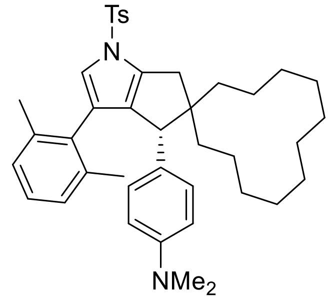

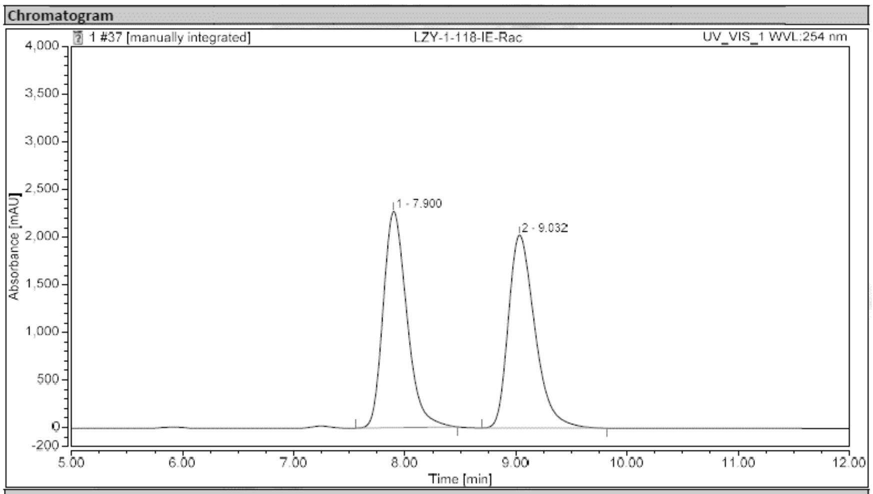

Integration Results

No.	Peak Name	Retention Time min	Area mAU*min	Height mAU	Relative Area %	Relative Height %	Amount n.a.
1		7.900	561.545	2273.685	50.03	52.87	n.a.
2		9.032	560.923	2026.530	49.97	47.13	n.a.
Total:			**1122.469**	**4300.215**	**100.00**	**100.00**	

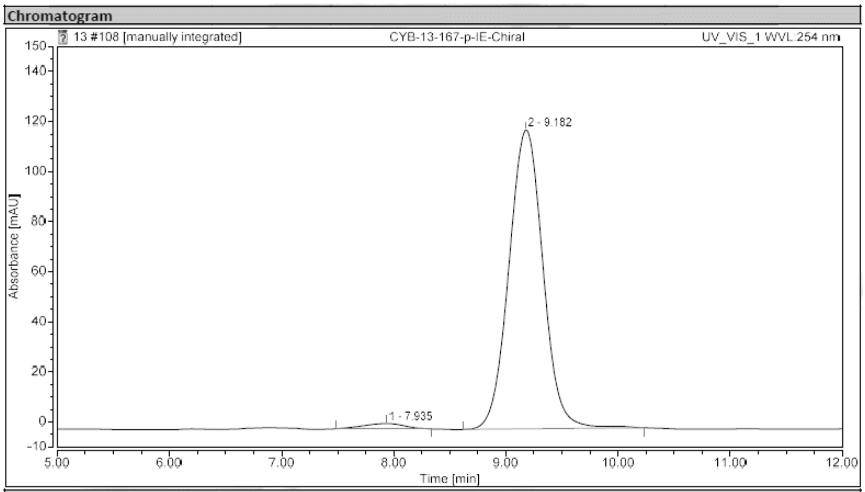

Integration Results

No.	Peak Name	Retention Time min	Area mAU*min	Height mAU	Relative Area %	Relative Height %	Amount n.a.
1		7.935	0.870	2.086	2.03	1.72	n.a.
2		9.182	42.070	119.416	97.97	98.28	n.a.
Total:			**42.940**	**121.502**	**100.00**	**100.00**	

Supplementary Figure 79. HPLC spectrum for compound **(-)-2ai**

(+)-1aj: IE, *i*-PrOH/hexane = 2/98, v = 1.0 mL/min, λ = 254 nm

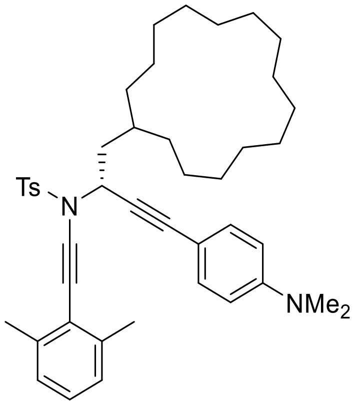

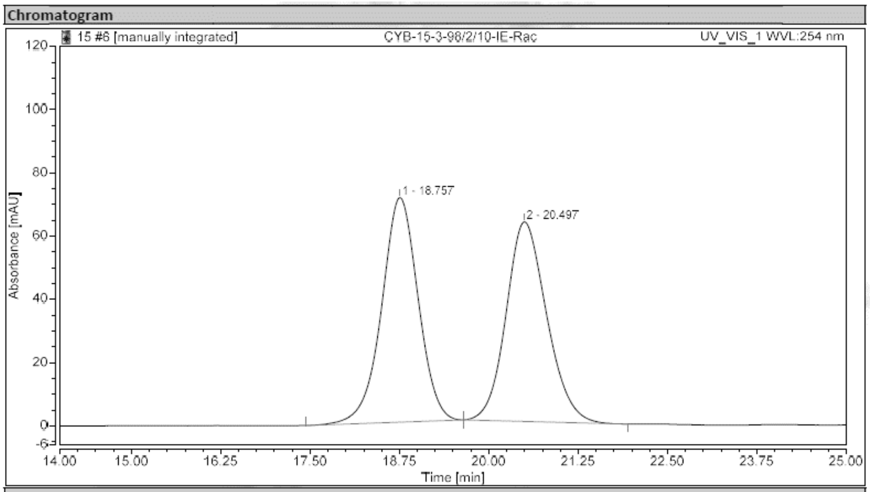

Integration Results

No.	Peak Name	Retention Time min	Area mAU*min	Height mAU	Relative Area %	Relative Height %	Amount n.a.
1		18.757	42.049	70.979	50.34	52.95	n.a.
2		20.497	41.486	63.082	49.66	47.05	n.a.
Total:			83.535	134.061	100.00	100.00	

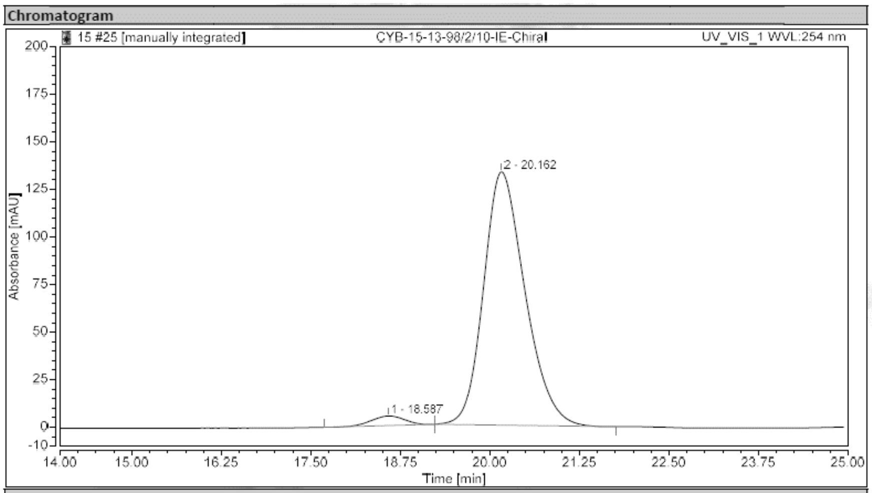

Integration Results

No.	Peak Name	Retention Time min	Area mAU*min	Height mAU	Relative Area %	Relative Height %	Amount n.a.
1		18.587	2.655	4.990	2.88	3.61	n.a.
2		20.162	89.395	133.180	97.12	96.39	n.a.
Total:			92.050	138.170	100.00	100.00	

Supplementary Figure 80. HPLC spectrum for compound **(+)-1aj**

(-)-2aj: IE, *i*-PrOH/hexane = 5/95, v = 1.0 mL/min, λ = 254 nm

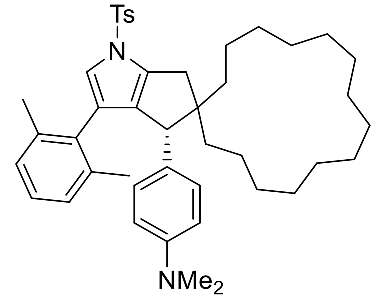

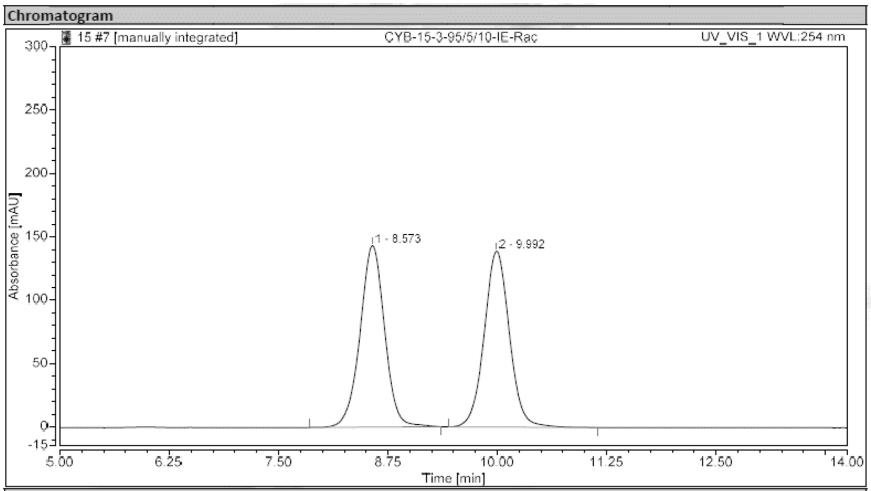

Integration Results

No.	Peak Name	Retention Time min	Area mAU*min	Height mAU	Relative Area %	Relative Height %	Amount n.a.
1		8.573	46.727	143.412	50.24	50.80	n.a.
2		9.992	46.285	138.892	49.76	49.20	n.a.
Total:			93.012	282.305	100.00	100.00	

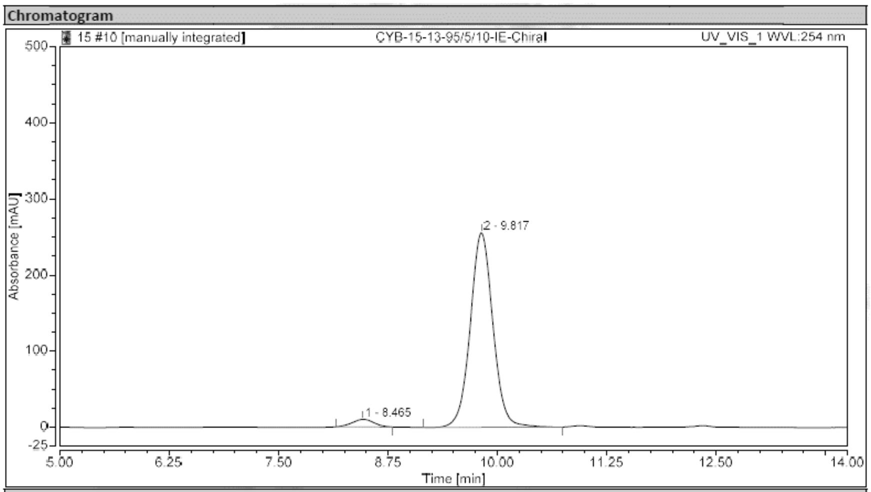

Integration Results

No.	Peak Name	Retention Time min	Area mAU*min	Height mAU	Relative Area %	Relative Height %	Amount n.a.
1		8.465	2.808	9.866	3.53	3.71	n.a.
2		9.817	76.619	255.728	96.47	96.29	n.a.
Total:			79.427	265.595	100.00	100.00	

Supplementary Figure 81. HPLC spectrum for compound **(-)-2aj**

(+)-1ak: IE, *i*-PrOH/hexane = 30/70, v = 1.0 mL/min, λ = 254 nm

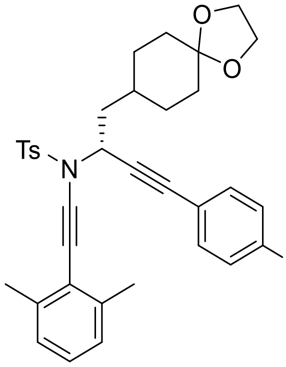

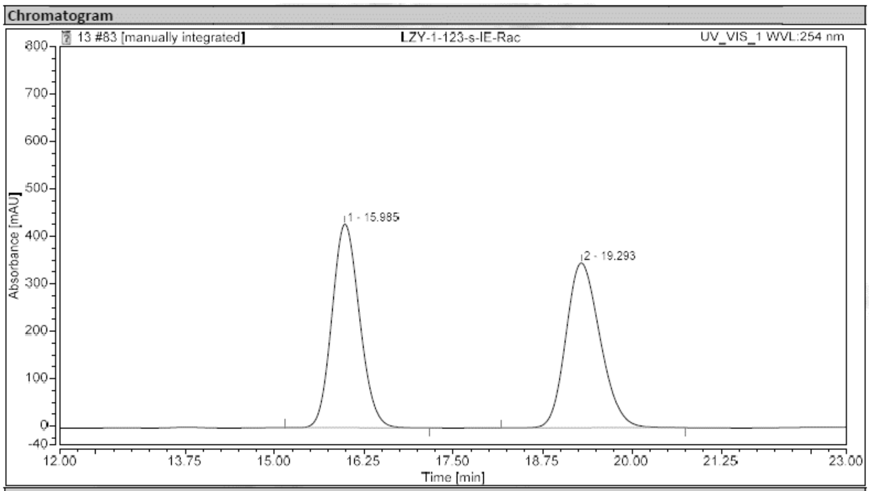

Integration Results

No.	Peak Name	Retention Time min	Area mAU*min	Height mAU	Relative Area %	Relative Height %	Amount n.a.
1		15.985	192.194	429.545	49.88	55.28	n.a.
2		19.293	193.083	347.535	50.12	44.72	n.a.
Total:			385.277	777.080	100.00	100.00	

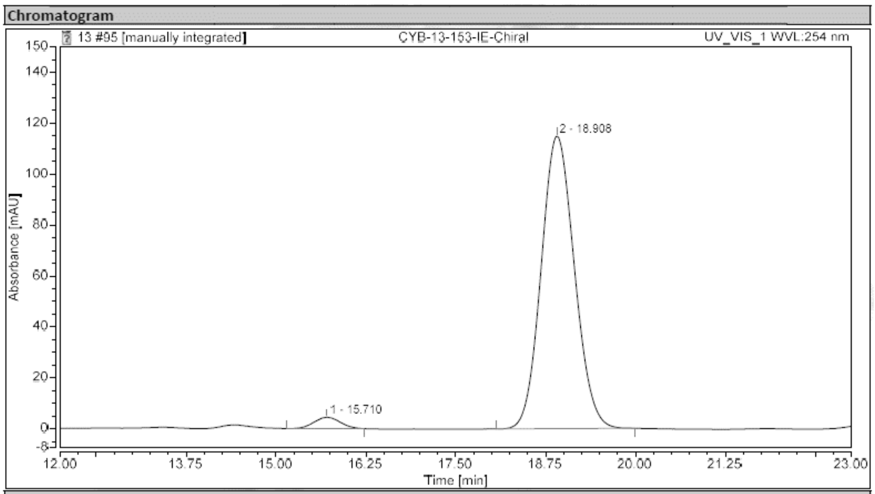

Integration Results

No.	Peak Name	Retention Time min	Area mAU*min	Height mAU	Relative Area %	Relative Height %	Amount n.a.
1		15.710	1.865	4.431	2.94	3.71	n.a.
2		18.908	61.599	114.960	97.06	96.29	n.a.
Total:			63.464	119.391	100.00	100.00	

Supplementary Figure 82. HPLC spectrum for compound **(+)-1ak**

(-)-2ak: IE, *i*-PrOH/hexane = 30/70, v = 1.0 mL/min, λ = 254 nm

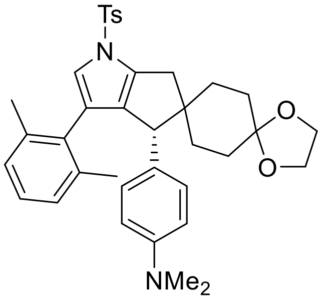

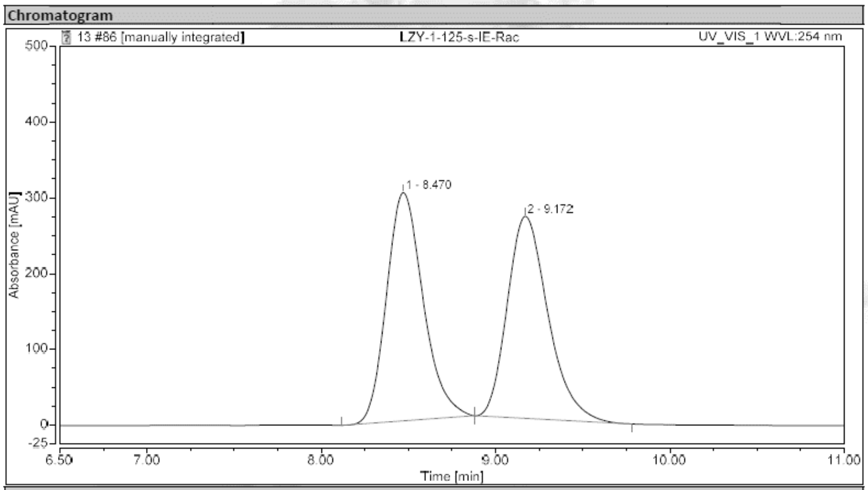

Integration Results

No.	Peak Name	Retention Time<br>min	Area<br>mAU*min	Height<br>mAU	Relative Area<br>%	Relative Height<br>%	Amount<br>n.a.
1		8.470	73.394	301.368	50.37	53.02	n.a.
2		9.172	72.324	266.998	49.63	46.98	n.a.
**Total:**			**145.718**	**568.366**	**100.00**	**100.00**	

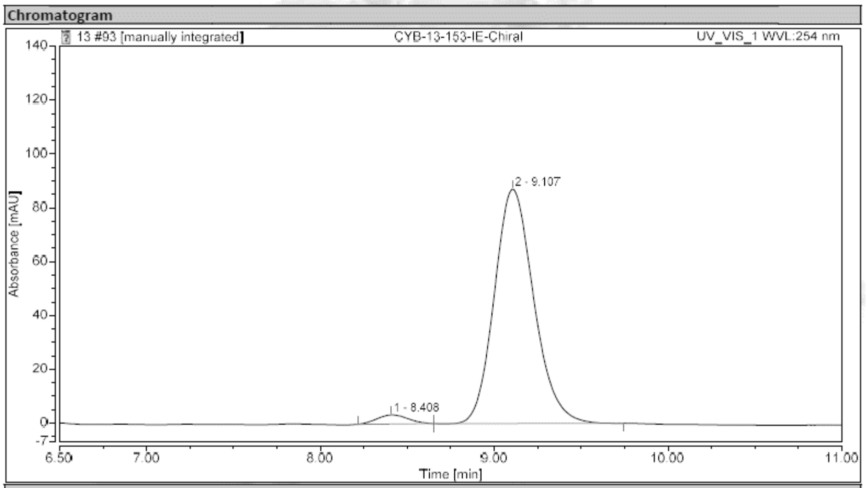

Integration Results

No.	Peak Name	Retention Time<br>min	Area<br>mAU*min	Height<br>mAU	Relative Area<br>%	Relative Height<br>%	Amount<br>n.a.
1		8.408	0.699	3.367	2.96	3.73	n.a.
2		9.107	22.886	87.011	97.04	96.27	n.a.
**Total:**			**23.586**	**90.378**	**100.00**	**100.00**	

Supplementary Figure 83. HPLC spectrum for compound **(-)-2ak**

(+)-1al: IE, *i*-PrOH/hexane = 5/95, v = 1.0 mL/min, λ = 254 nm

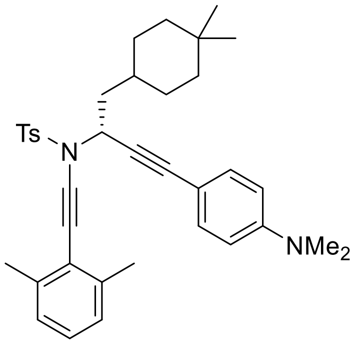

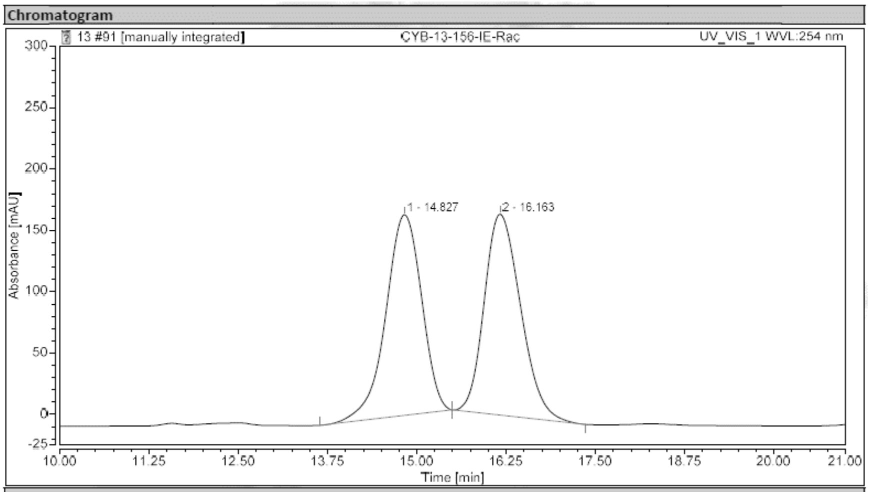

Integration Results

No.	Peak Name	Retention Time min	Area mAU*min	Height mAU	Relative Area %	Relative Height %	Amount n.a.
1		14.827	95.322	163.538	49.64	49.96	n.a.
2		16.163	96.721	163.816	50.36	50.04	n.a.
Total:			192.044	327.354	100.00	100.00	

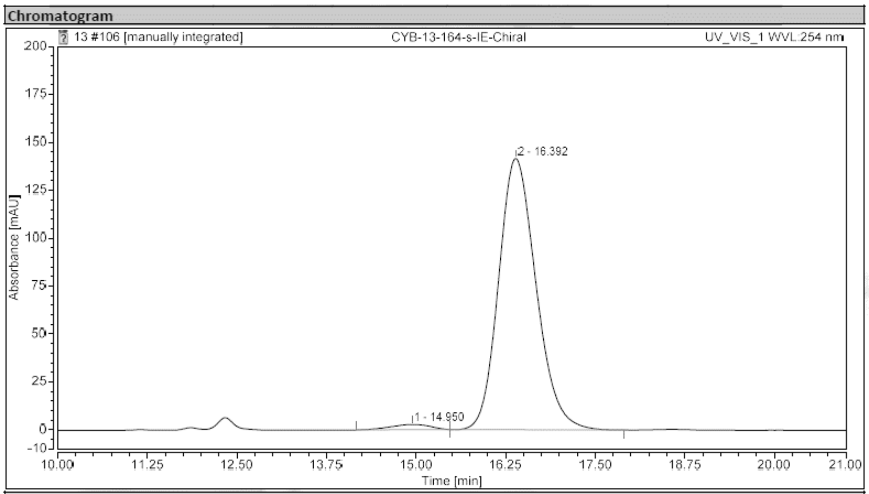

Integration Results

No.	Peak Name	Retention Time min	Area mAU*min	Height mAU	Relative Area %	Relative Height %	Amount n.a.
1		14.950	1.743	2.725	1.99	1.89	n.a.
2		16.392	85.745	141.536	98.01	98.11	n.a.
Total:			87.488	144.261	100.00	100.00	

Supplementary Figure 84. HPLC spectrum for compound **(+)-1al**

(-)-2al: IE, *i*-PrOH/hexane = 5/95, v = 1.0 mL/min, λ = 254 nm

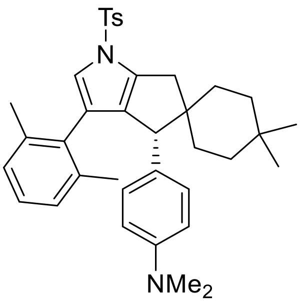

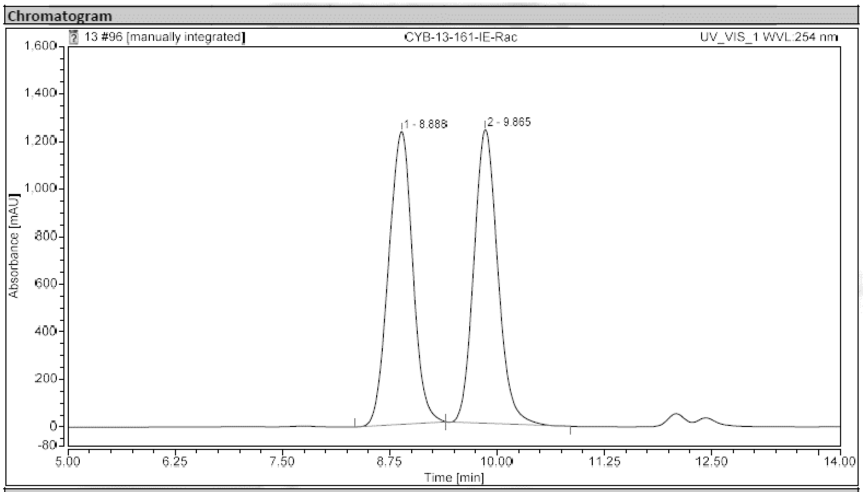

Integration Results

No.	Peak Name	Retention Time min	Area mAU*min	Height mAU	Relative Area %	Relative Height %	Amount n.a.
1		8.888	399.996	1232.310	50.14	49.94	n.a.
2		9.865	397.784	1235.250	49.86	50.06	n.a.
**Total:**			**797.780**	**2467.560**	**100.00**	**100.00**	

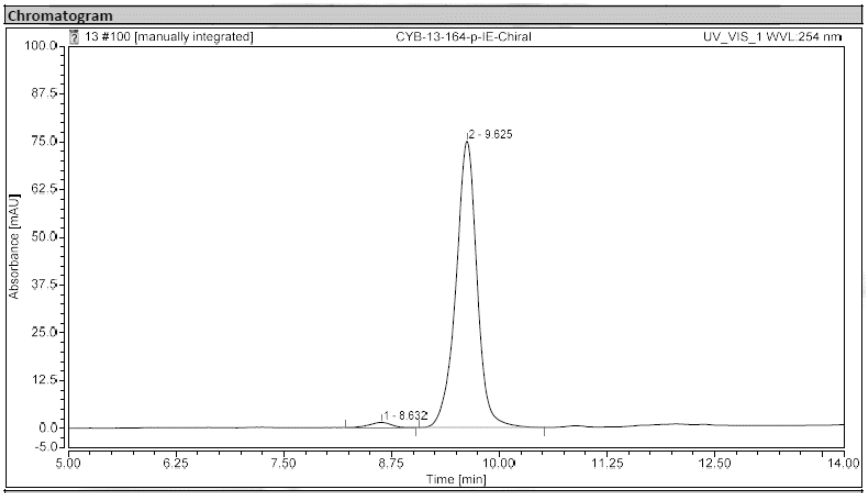

Integration Results

No.	Peak Name	Retention Time min	Area mAU*min	Height mAU	Relative Area %	Relative Height %	Amount n.a.
1		8.632	0.397	1.356	1.83	1.78	n.a.
2		9.625	21.302	74.954	98.17	98.22	n.a.
**Total:**			**21.699**	**76.310**	**100.00**	**100.00**	

Supplementary Figure 85. HPLC spectrum for compound **(-)-2al**

(+)-1am: IE, *i*-PrOH/hexane = 3/97, v = 1.0 mL/min, λ = 254 nm

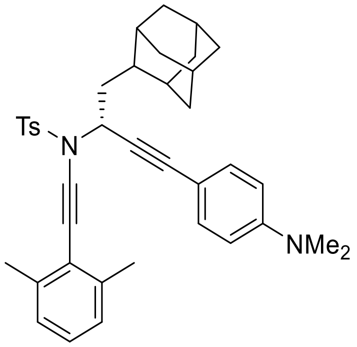

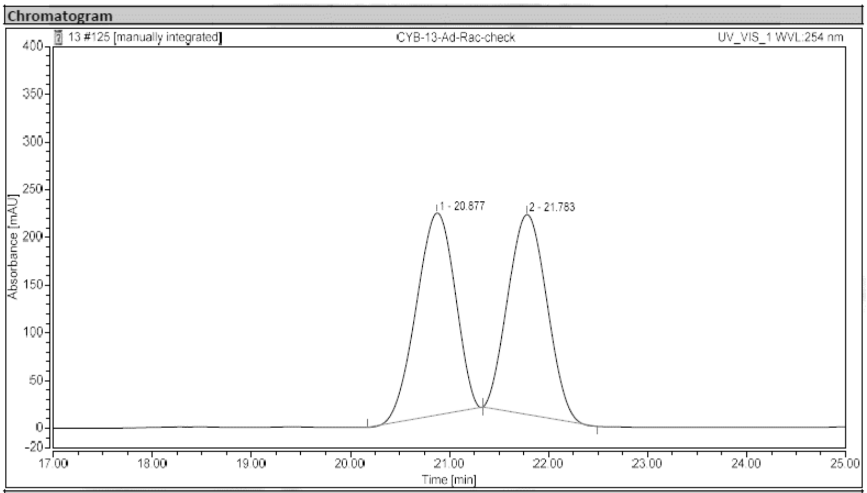

Integration Results

No.	Peak Name	Retention Time min	Area mAU*min	Height mAU	Relative Area %	Relative Height %	Amount n.a.
1		20.877	95.488	211.672	50.08	50.26	n.a.
2		21.783	95.185	209.481	49.92	49.74	n.a.
Total:			190.673	421.153	100.00	100.00	

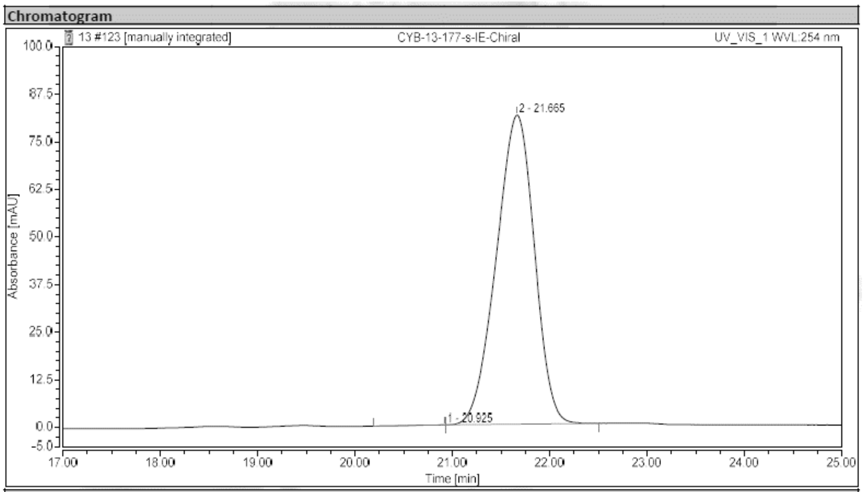

Integration Results

No.	Peak Name	Retention Time min	Area mAU*min	Height mAU	Relative Area %	Relative Height %	Amount n.a.
1		20.925	0.010	0.008	0.03	0.01	n.a.
2		21.665	36.849	81.166	99.97	99.99	n.a.
Total:			36.859	81.173	100.00	100.00	

Supplementary Figure 86. HPLC spectrum for compound **(+)-1am**

(-)-2am: IE, *i*-PrOH/hexane = 10/90, v = 1.0 mL/min, λ = 254 nm

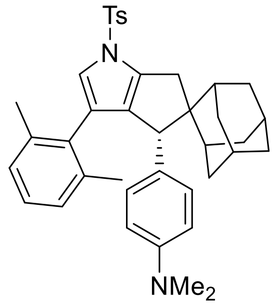

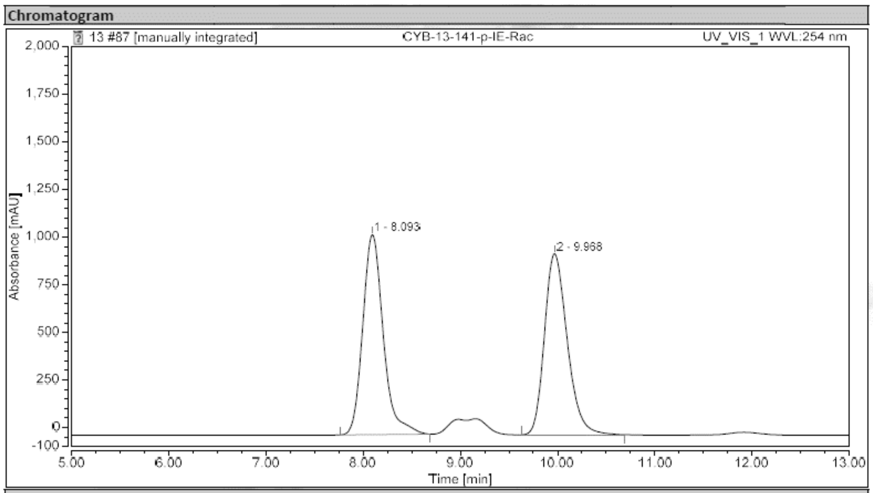

Integration Results

No.	Peak Name	Retention Time<br>min	Area<br>mAU*min	Height<br>mAU	Relative Area<br>%	Relative Height<br>%	Amount<br>n.a.
1		8.093	256.835	1050.530	49.88	52.47	n.a.
2		9.968	258.071	951.576	50.12	47.53	n.a.
**Total:**			**514.905**	**2002.106**	**100.00**	**100.00**	

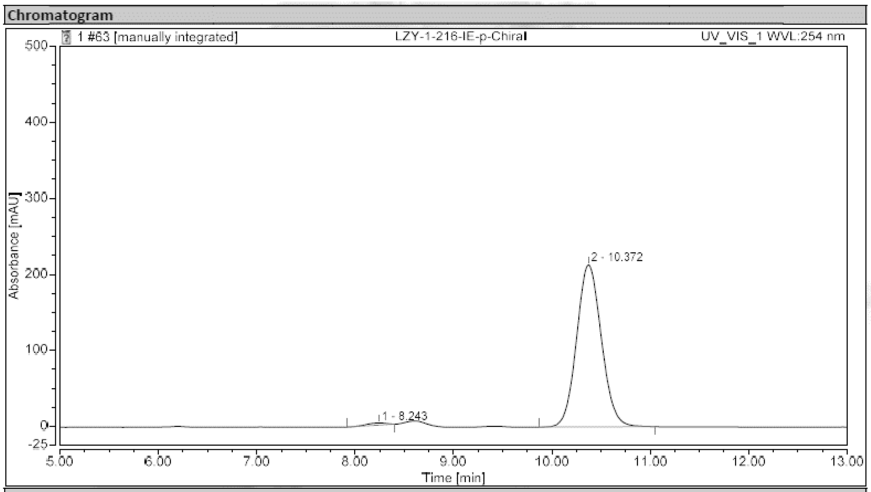

Integration Results

No.	Peak Name	Retention Time<br>min	Area<br>mAU*min	Height<br>mAU	Relative Area<br>%	Relative Height<br>%	Amount<br>n.a.
1		8.243	0.653	2.779	1.04	1.29	n.a.
2		10.372	62.302	213.087	98.96	98.71	n.a.
**Total:**			**62.955**	**215.866**	**100.00**	**100.00**	

Supplementary Figure 87. HPLC spectrum for compound **(-)-2am**

(+)-1an: ODH, *i*-PrOH/hexane = 1/99, v = 1.0 mL/min, λ = 254 nm

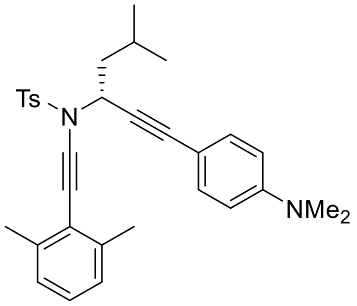

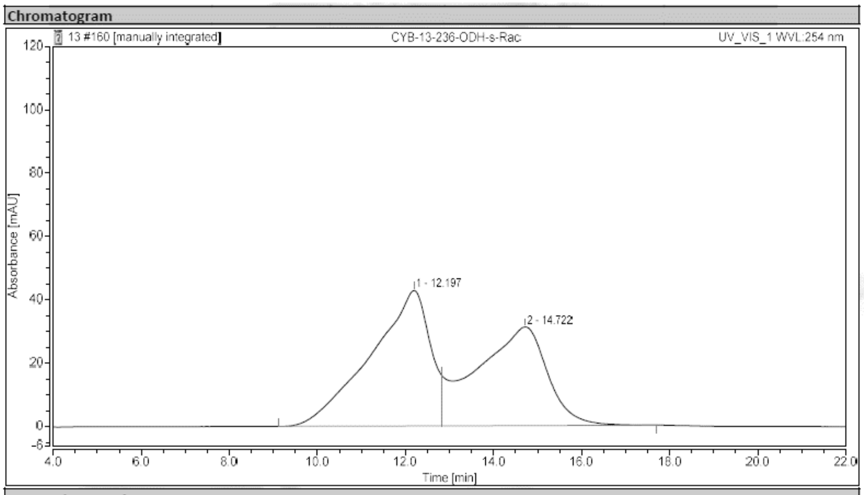

Integration Results

No.	Peak Name	Retention Time min	Area mAU*min	Height mAU	Relative Area %	Relative Height %	Amount n.a.
1		12.197	66.631	42.766	53.38	57.85	n.a.
2		14.722	58.194	31.156	46.62	42.15	n.a.
**Total:**			**124.825**	**73.922**	**100.00**	**100.00**	

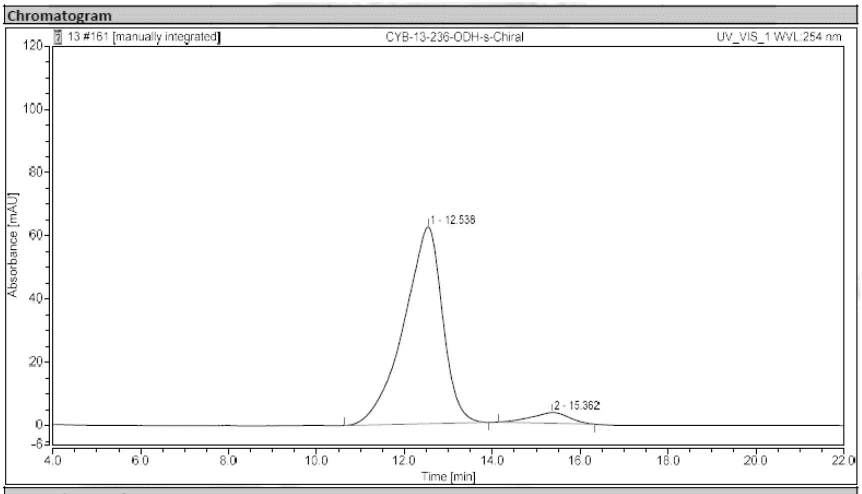

Integration Results

No.	Peak Name	Retention Time min	Area mAU*min	Height mAU	Relative Area %	Relative Height %	Amount n.a.
1		12.538	63.691	62.223	95.01	94.83	n.a.
2		15.362	3.342	3.389	4.99	5.17	n.a.
**Total:**			**67.033**	**65.612**	**100.00**	**100.00**	

Supplementary Figure 88. HPLC spectrum for compound **(+)-1an**

(-)-2an: IC, *i*-PrOH/hexane = 10/90, v = 1.0 mL/min, λ = 254 nm

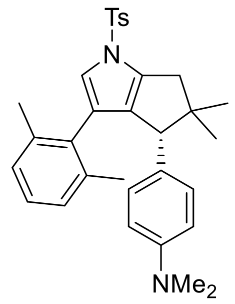

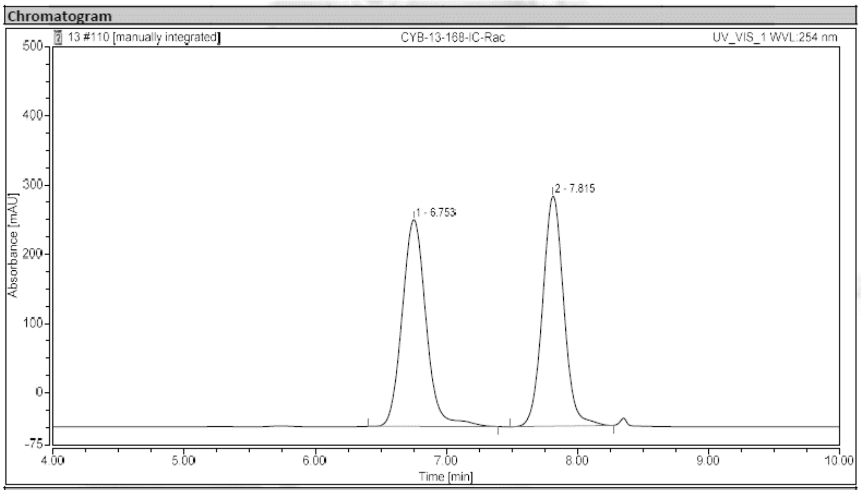

No.	Peak Name	Retention Time min	Area mAU*min	Height mAU	Relative Area %	Relative Height %	Amount n.a.
1		6.753	63.266	298.969	50.45	47.34	n.a.
2		7.815	62.149	332.606	49.55	52.66	n.a.
Total:			125.415	631.574	100.00	100.00	

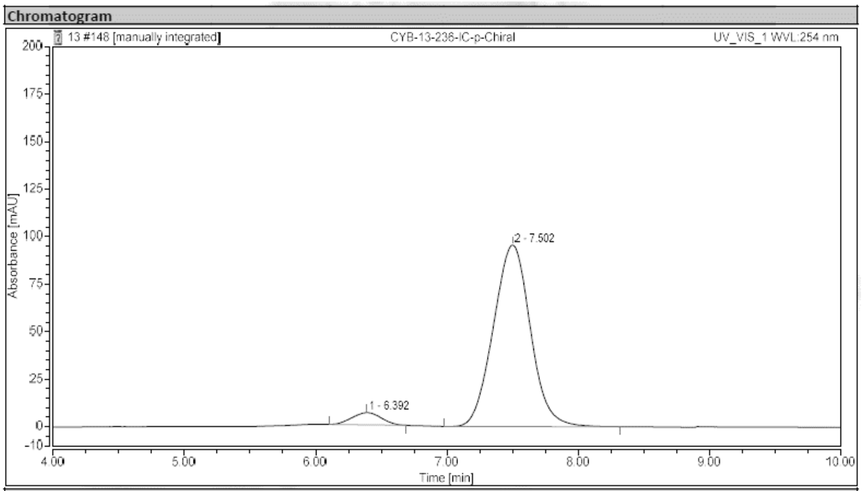

No.	Peak Name	Retention Time min	Area mAU*min	Height mAU	Relative Area %	Relative Height %	Amount n.a.
1		6.392	1.655	6.409	5.04	6.29	n.a.
2		7.502	31.205	95.483	94.96	93.71	n.a.
Total:			32.860	101.892	100.00	100.00	

Supplementary Figure 89. HPLC spectrum for compound **(-)-2an**

(+)-1ao: ODH, *i*-PrOH/hexane = 1/99, v = 1.0 mL/min, λ = 254 nm

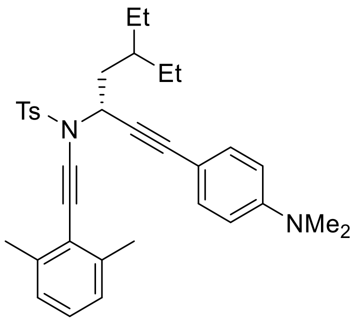

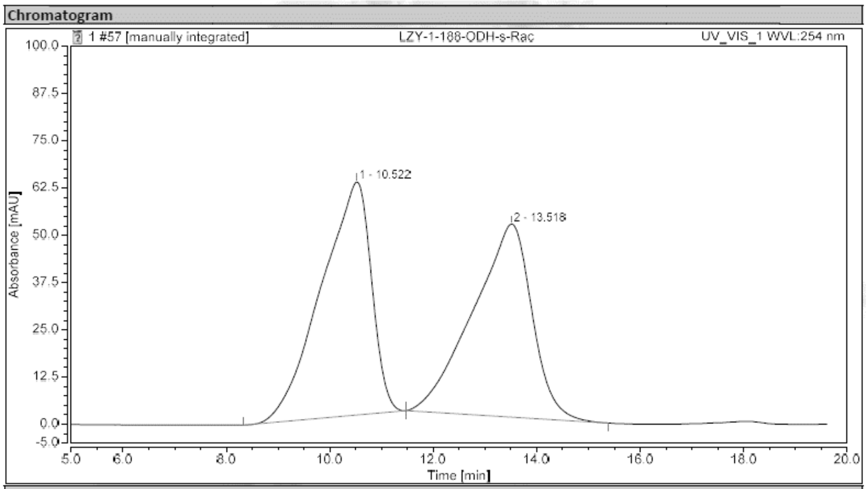

Integration Results

No.	Peak Name	Retention Time min	Area mAU*min	Height mAU	Relative Area %	Relative Height %	Amount n.a.
1		10.522	69.465	61.637	50.70	54.68	n.a.
2		13.518	67.542	51.083	49.30	45.32	n.a.
**Total:**			**137.007**	**112.720**	**100.00**	**100.00**	

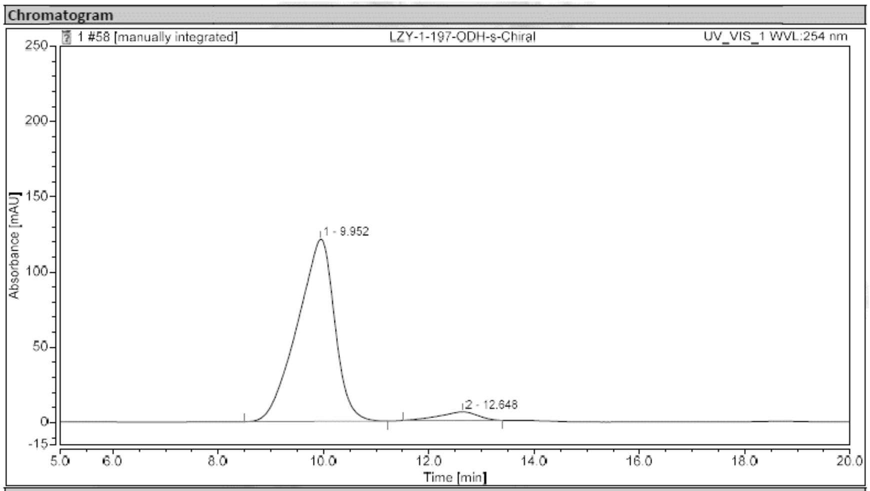

Integration Results

No.	Peak Name	Retention Time min	Area mAU*min	Height mAU	Relative Area %	Relative Height %	Amount n.a.
1		9.952	99.315	121.134	95.44	95.52	n.a.
2		12.648	4.750	5.677	4.56	4.48	n.a.
**Total:**			**104.065**	**126.811**	**100.00**	**100.00**	

Supplementary Figure 90. HPLC spectrum for compound **(+)-1ao**

(-)-2ao: IC, *i*-PrOH/hexane = 2/98, v = 1.0 mL/min, λ = 254 nm

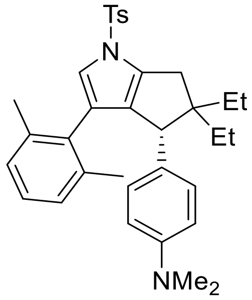

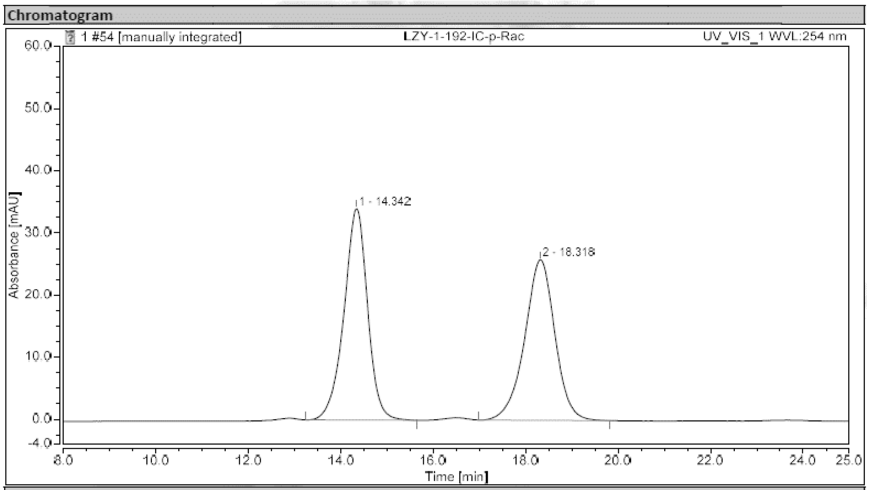

Integration Results

No.	Peak Name	Retention Time min	Area mAU*min	Height mAU	Relative Area %	Relative Height %	Amount n.a.
1		14.342	20.135	34.031	50.74	56.85	n.a.
2		18.318	19.551	25.830	49.26	43.15	n.a.
Total:			39.686	59.861	100.00	100.00	

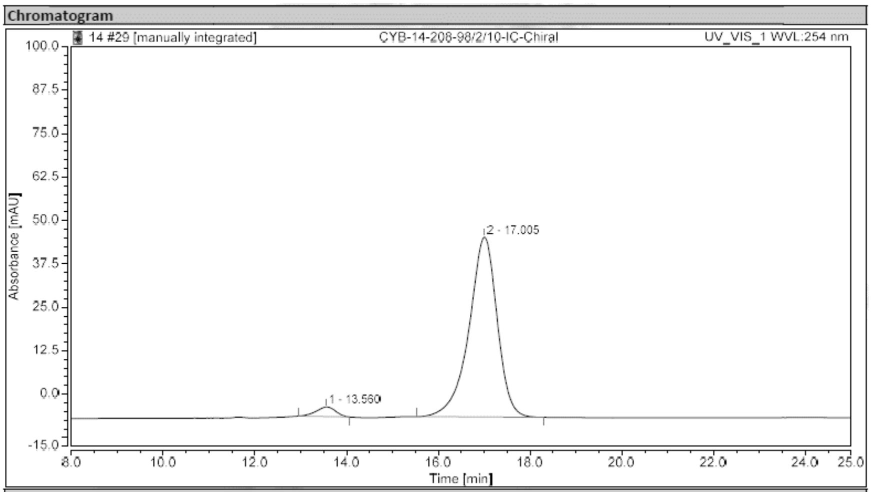

Integration Results

No.	Peak Name	Retention Time min	Area mAU*min	Height mAU	Relative Area %	Relative Height %	Amount n.a.
1		13.560	1.355	2.764	3.55	5.07	n.a.
2		17.005	36.810	51.779	96.45	94.93	n.a.
Total:			38.165	54.543	100.00	100.00	

Supplementary Figure 91. HPLC spectrum for compound **(-)-2ao**

(+)-1ap: ADH, *i*-PrOH/hexane = 10/90, v = 1.0 mL/min, λ = 254 nm

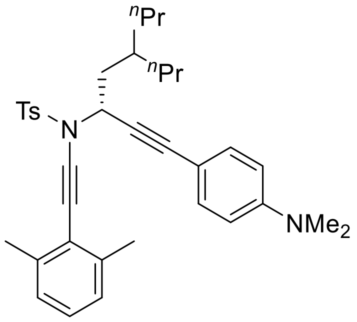

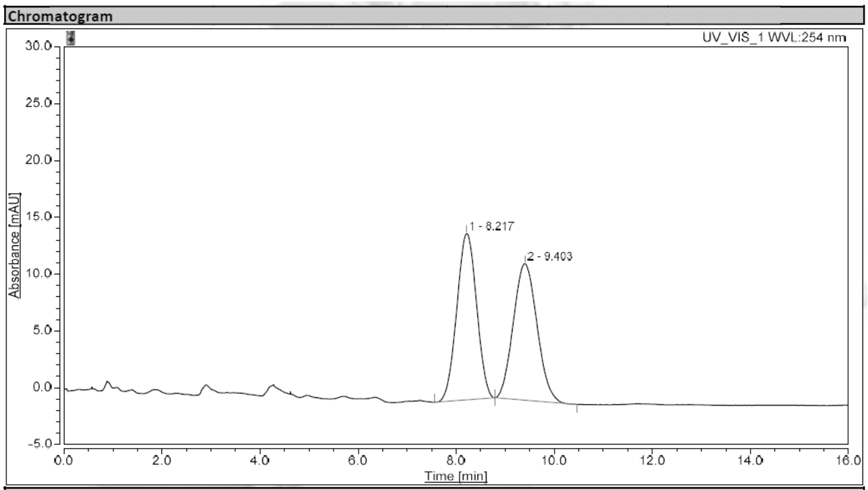

Integration Results

No.	Peak Name	Retention Time min	Area mAU*min	Height mAU	Relative Area %	Relative Height %	Amount n.a.
1		8.217	6.820	14.657	50.33	54.95	n.a.
2		9.403	6.731	12.016	49.67	45.05	n.a.
Total:			13.552	26.673	100.00	100.00	

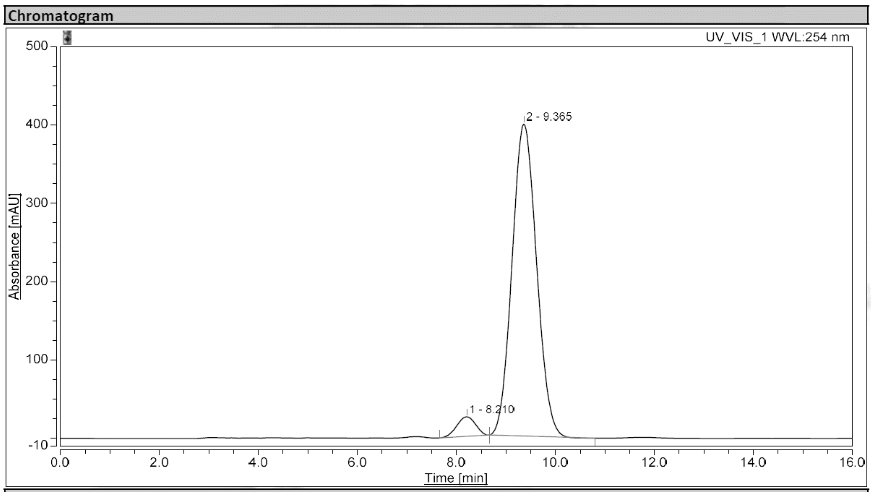

Integration Results

No.	Peak Name	Retention Time min	Area mAU*min	Height mAU	Relative Area %	Relative Height %	Amount n.a.
1		8.210	10.860	24.933	4.54	5.89	n.a.
2		9.365	228.105	398.100	95.46	94.11	n.a.
Total:			238.965	423.033	100.00	100.00	

Supplementary Figure 92. HPLC spectrum for compound **(+)-1ap**

(-)-2ap: IE, *i*-PrOH/hexane = 2/98, v = 1.0 mL/min, λ = 254 nm

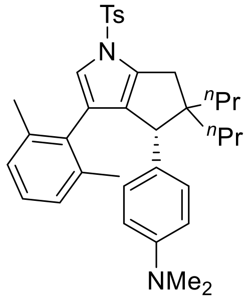

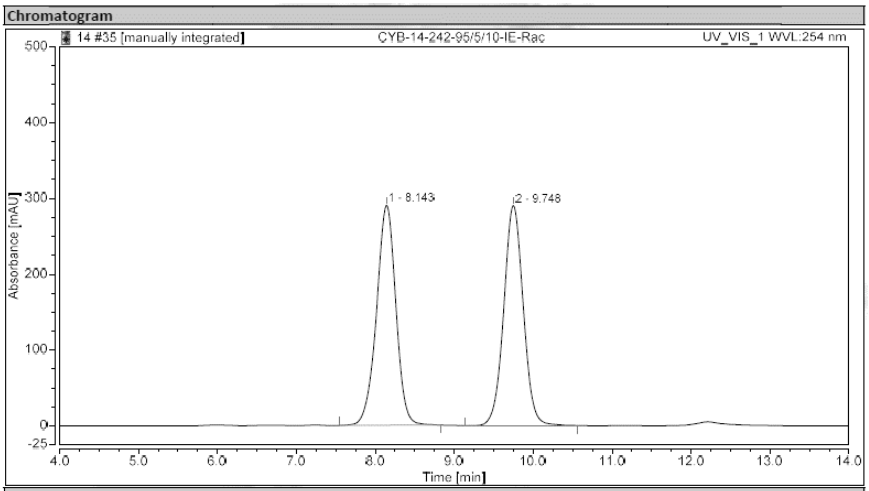

Integration Results

No.	Peak Name	Retention Time min	Area mAU*min	Height mAU	Relative Area %	Relative Height %	Amount n.a.
1		8.143	82.492	290.366	49.91	49.98	n.a.
2		9.748	82.804	290.577	50.09	50.02	n.a.
Total:			165.296	580.943	100.00	100.00	

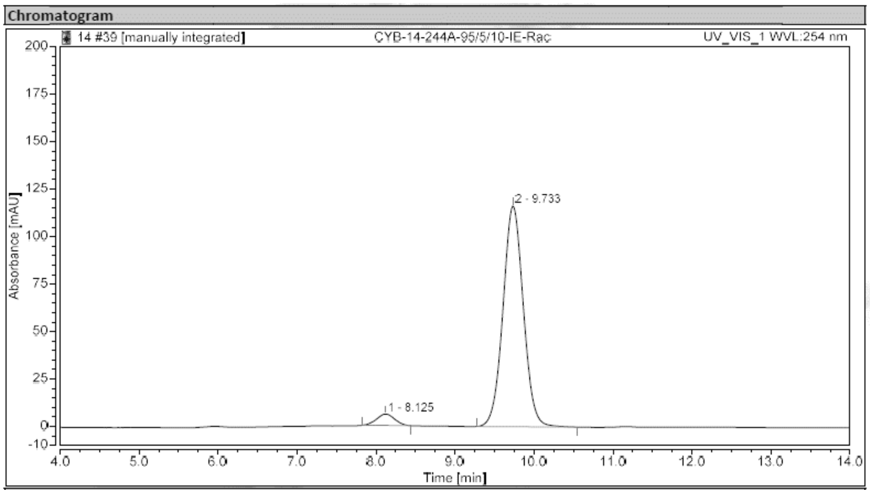

Integration Results

No.	Peak Name	Retention Time min	Area mAU*min	Height mAU	Relative Area %	Relative Height %	Amount n.a.
1		8.125	1.583	5.971	4.37	4.88	n.a.
2		9.733	34.639	116.282	95.63	95.12	n.a.
Total:			36.222	122.253	100.00	100.00	

Supplementary Figure 93. HPLC spectrum for compound **(-)-2ap**

(+)-1aq: ADH, *i*-PrOH/hexane = 20/80, v = 1.0 mL/min, λ = 254 nm

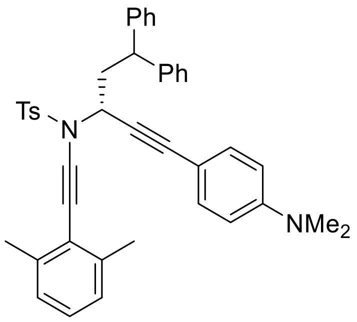

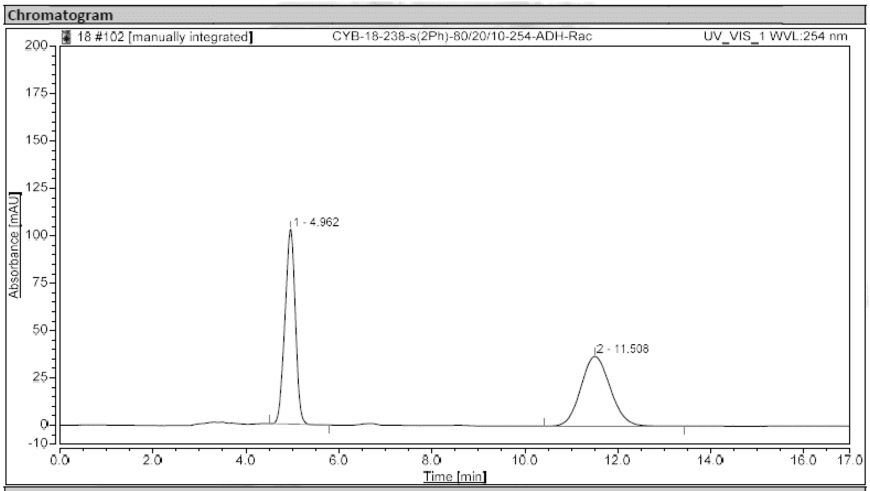

Integration Results

No.	Peak Name	Retention Time min	Area mAU*min	Height mAU	Relative Area %	Relative Height %	Amount n.a.
1		4.962	26.485	102.801	49.62	73.66	n.a.
2		11.508	26.891	36.755	50.38	26.34	n.a.
Total:			53.376	139.556	100.00	100.00	

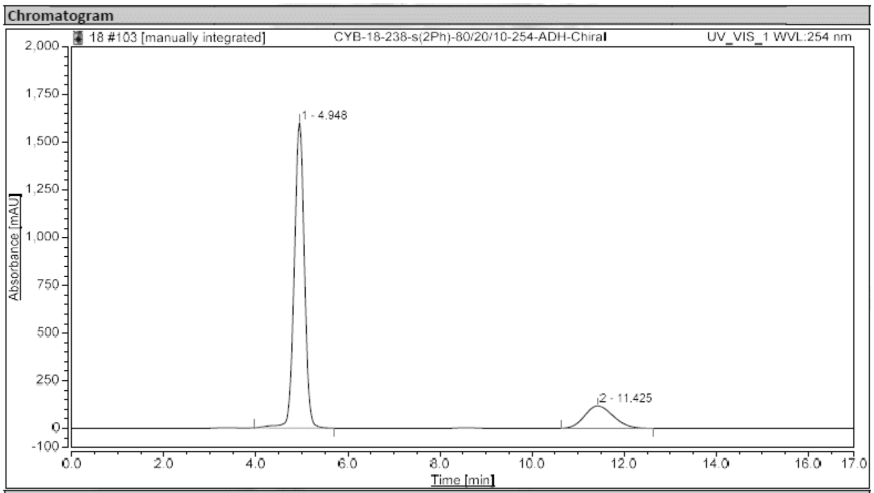

Integration Results

No.	Peak Name	Retention Time min	Area mAU*min	Height mAU	Relative Area %	Relative Height %	Amount n.a.
1		4.948	407.349	1600.188	82.92	93.17	n.a.
2		11.425	83.882	117.250	17.08	6.83	n.a.
Total:			491.231	1717.438	100.00	100.00	

Supplementary Figure 94. HPLC spectrum for compound **(+)-1aq**

(-)-2aq: IE, *i*-PrOH/hexane = 20/80, v = 1.0 mL/min, λ = 254 nm

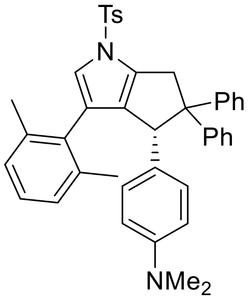

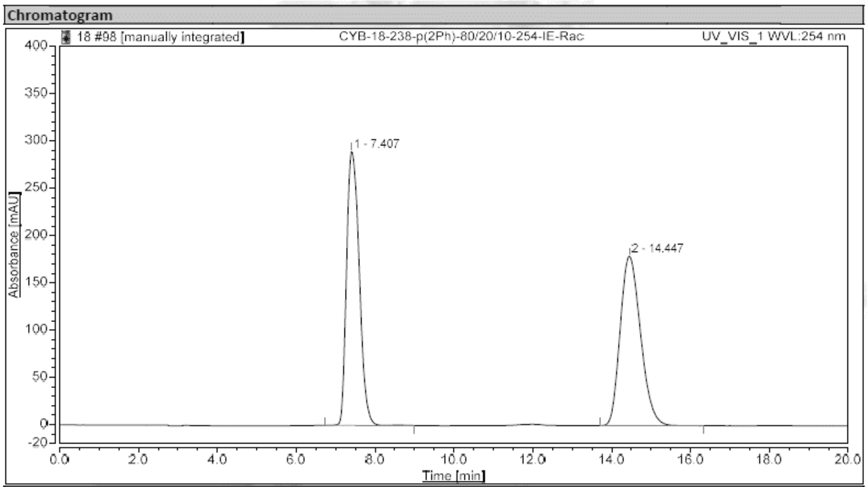

Integration Results

No.	Peak Name	Retention Time min	Area mAU*min	Height mAU	Relative Area %	Relative Height %	Amount n.a.
1		7.407	107.830	290.198	50.13	61.82	n.a.
2		14.447	107.291	179.228	49.87	38.18	n.a.
Total:			215.121	469.426	100.00	100.00	

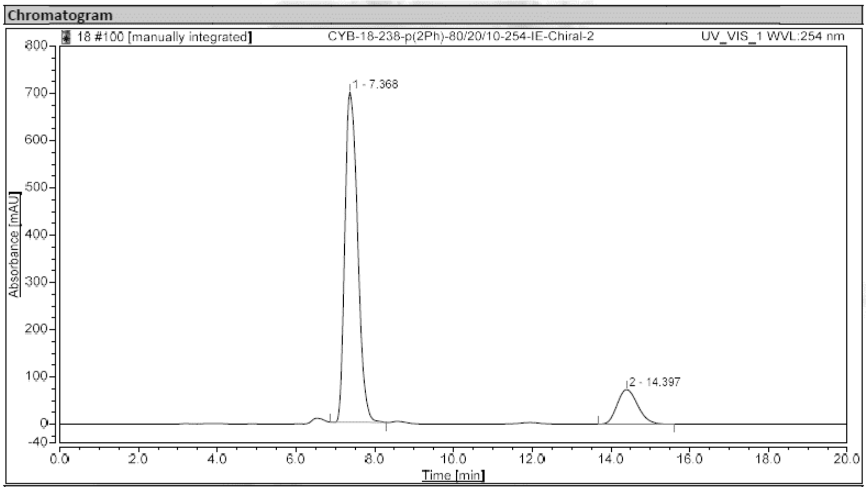

Integration Results

No.	Peak Name	Retention Time min	Area mAU*min	Height mAU	Relative Area %	Relative Height %	Amount n.a.
1		7.368	261.530	698.795	85.59	90.53	n.a.
2		14.397	44.034	73.082	14.41	9.47	n.a.
Total:			305.563	771.877	100.00	100.00	

Supplementary Figure 95. HPLC spectrum for compound **(-)-2aq**

(+)-1ar (1:1 dr)**: IE**, *i*-PrOH/hexane = 10/90, v = 1.0 mL/min, λ = 254 nm

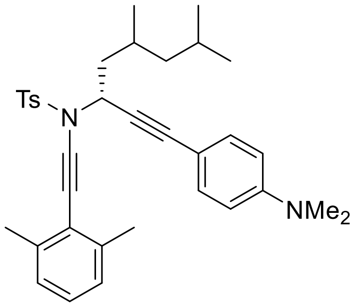

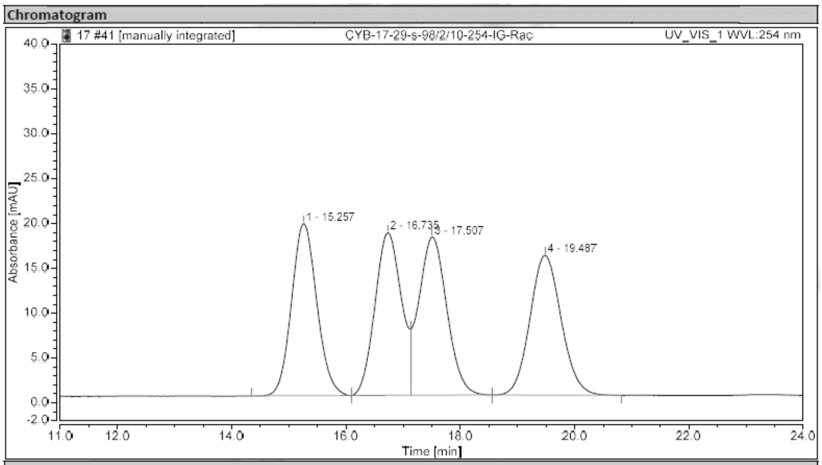

Integration Results

No.	Peak Name	Retention Time min	Area mAU*min	Height mAU	Relative Area %	Relative Height %	Amount n.a.
1		15.257	9.841	19.215	24.93	27.21	n.a.
2		16.735	9.717	18.151	24.61	25.70	n.a.
3		17.507	10.032	17.652	25.41	25.00	n.a.
4		19.487	9.888	15.599	25.05	22.09	n.a.
Total:			39.478	70.616	100.00	100.00	

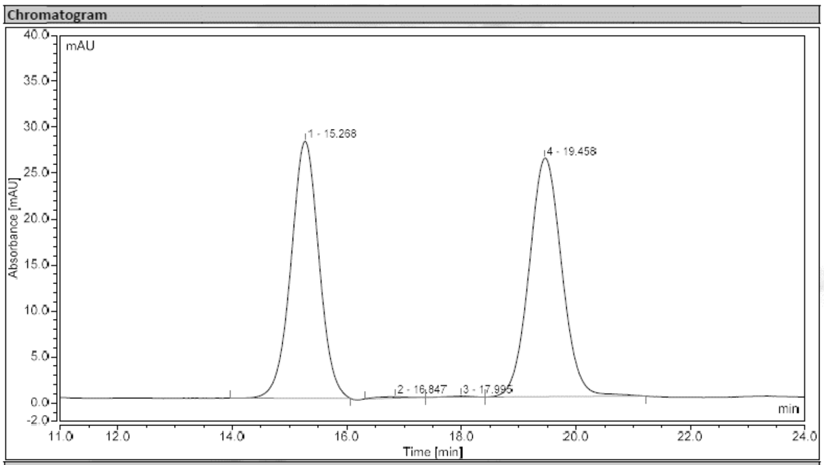

Integration Results

No.	Peak Name	Retention Time min	Area mAU*min	Height mAU	Relative Area %	Relative Height %	Amount n.a.
1		15.268	15.836	27.981	47.73	51.61	n.a.
2		16.847	0.097	0.147	0.29	0.27	n.a.
3		17.995	0.053	0.118	0.16	0.22	n.a.
4		19.458	17.192	25.968	51.82	47.90	n.a.
Total:			33.178	54.213	100.00	100.00	

Supplementary Figure 96. HPLC spectrum for compound **(+)-1ar**

(-)-2ar: IE, *i*-PrOH/hexane = 5/95, v = 1.0 mL/min, λ = 254 nm

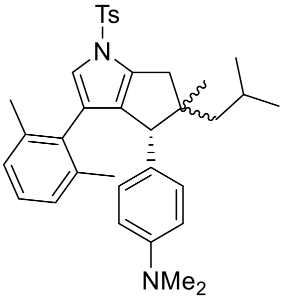

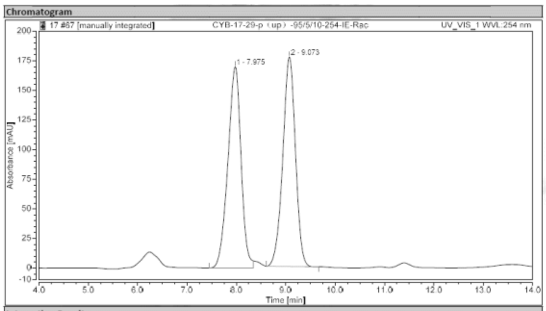

Integration Results

No.	Peak Name	Retention Time min	Area mAU*min	Height mAU	Relative Area %	Relative Height %	Amount n.a.
1		7.975	52.679	170.076	50.15	49.00	n.a.
2		9.073	52.356	177.016	49.85	51.00	n.a.
Total:			105.035	347.093	100.00	100.00	

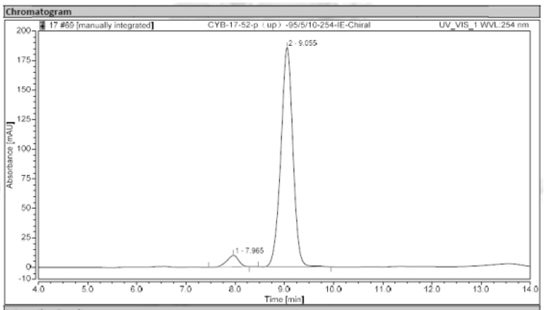

Integration Results

No.	Peak Name	Retention Time min	Area mAU*min	Height mAU	Relative Area %	Relative Height %	Amount n.a.
1		7.965	2.658	9.638	4.83	4.94	n.a.
2		9.055	52.379	185.529	95.17	95.06	n.a.
Total:			55.037	195.167	100.00	100.00	

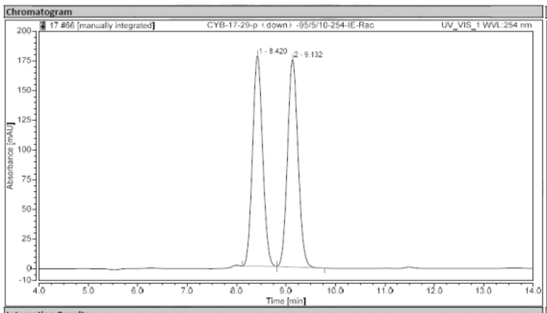

Integration Results

No.	Peak Name	Retention Time min	Area mAU*min	Height mAU	Relative Area %	Relative Height %	Amount n.a.
1		8.420	41.795	177.317	49.26	50.35	n.a.
2		9.132	43.049	174.843	50.74	49.65	n.a.
Total:			84.845	352.160	100.00	100.00	

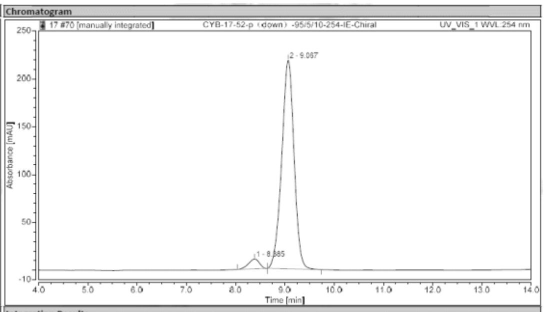

Integration Results

No.	Peak Name	Retention Time min	Area mAU*min	Height mAU	Relative Area %	Relative Height %	Amount n.a.
1		8.385	2.614	10.357	3.98	4.53	n.a.
2		9.067	63.110	218.118	96.02	95.47	n.a.
Total:			65.724	228.475	100.00	100.00	

Supplementary Figure 97. HPLC spectrum for compound **(-)-2ar**

(+)-1as: IE, *i*-PrOH/hexane = 10/90, v = 1.0 mL/min, λ = 254 nm

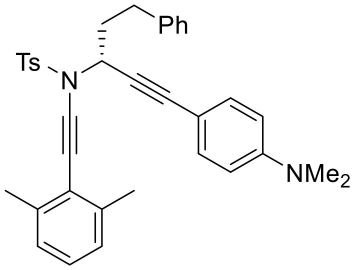

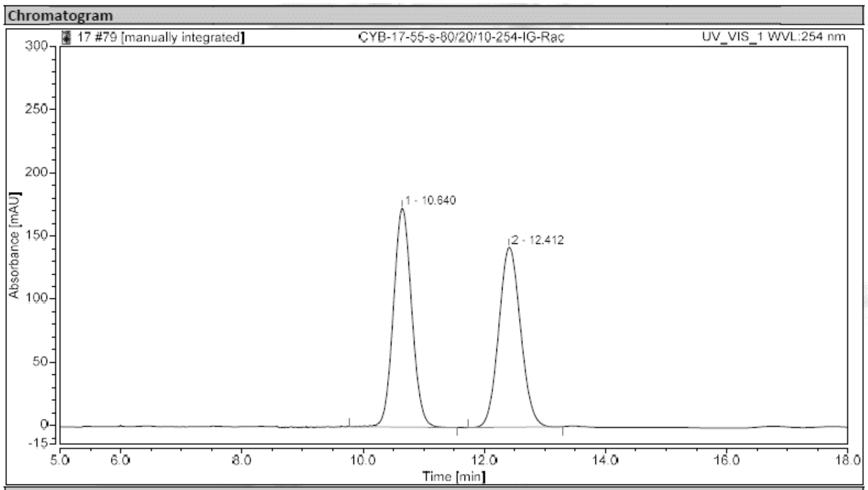

Integration Results

No.	Peak Name	Retention Time min	Area mAU*min	Height mAU	Relative Area %	Relative Height %	Amount n.a.
1		10.640	60.605	173.534	50.43	54.88	n.a.
2		12.412	59.568	142.686	49.57	45.12	n.a.
Total:			**120.173**	**316.220**	**100.00**	**100.00**	

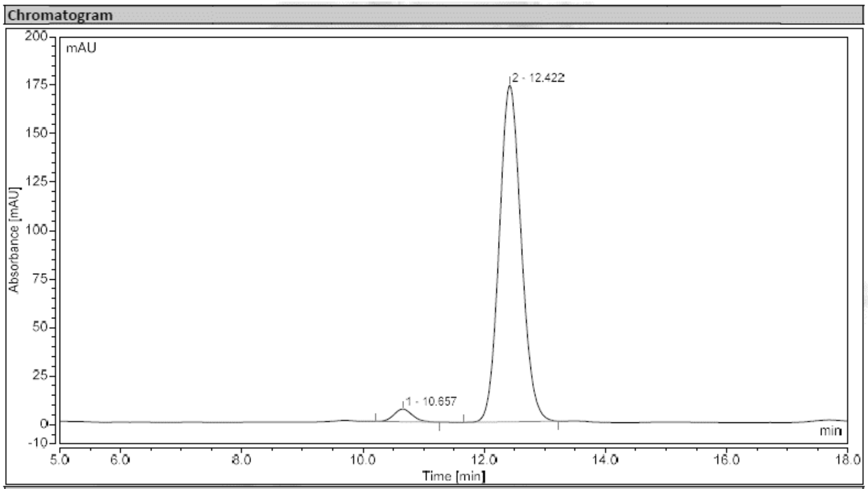

Integration Results

No.	Peak Name	Retention Time min	Area mAU*min	Height mAU	Relative Area %	Relative Height %	Amount n.a.
1		10.657	2.297	6.522	3.06	3.62	n.a.
2		12.422	72.825	173.570	96.94	96.38	n.a.
Total:			**75.122**	**180.092**	**100.00**	**100.00**	

Supplementary Figure 98. HPLC spectrum for compound **(+)-1as**

(-)-2as: IE, *i*-PrOH/hexane = 2/98, v = 1.0 mL/min, λ = 254 nm

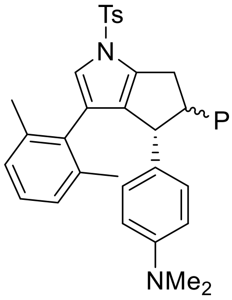

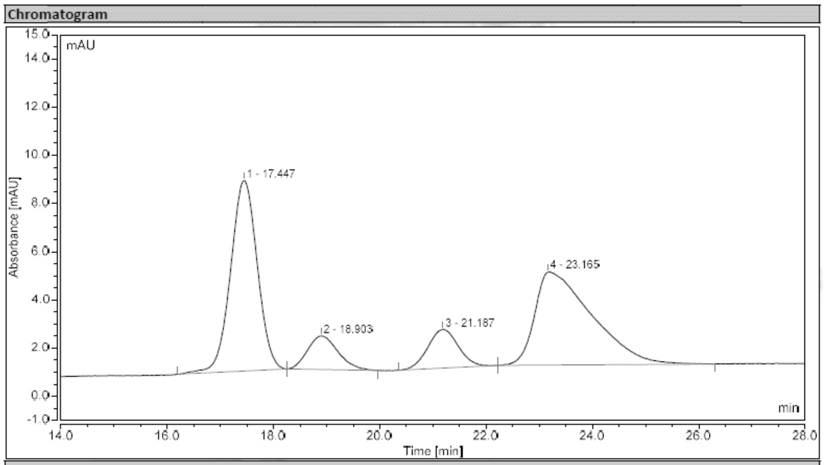

Integration Results

No.	Peak Name	Retention Time min	Area mAU*min	Height mAU	Relative Area %	Relative Height %	Amount n.a.
1		17.447	4.593	7.909	40.28	53.45	n.a.
2		18.903	0.923	1.400	8.10	9.46	n.a.
3		21.187	1.066	1.613	9.35	10.90	n.a.
4		23.165	4.819	3.874	42.27	26.18	n.a.
Total:			11.401	14.796	100.00	100.00	

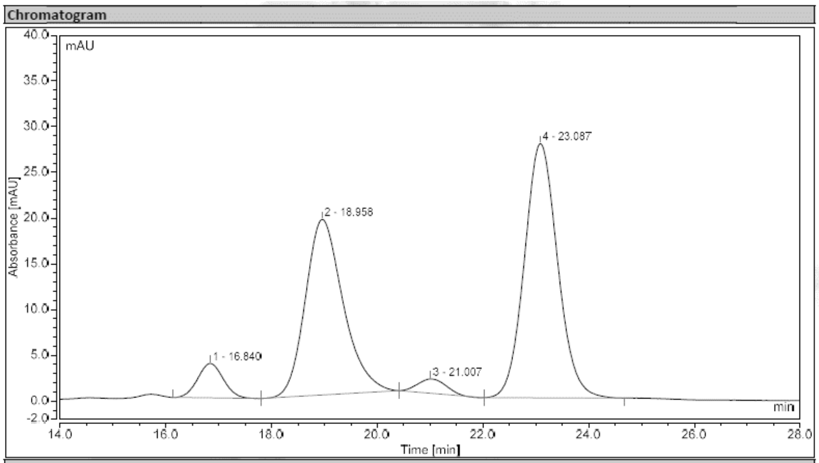

Integration Results

No.	Peak Name	Retention Time min	Area mAU*min	Height mAU	Relative Area %	Relative Height %	Amount n.a.
1		16.840	2.115	3.768	5.39	7.20	n.a.
2		18.958	15.907	19.210	40.54	36.70	n.a.
3		21.007	0.956	1.569	2.44	3.00	n.a.
4		23.087	20.259	27.803	51.63	53.11	n.a.
Total:			39.236	52.349	100.00	100.00	

Supplementary Figure 99. HPLC spectrum for compound **(-)-2as**

(+)-2a: IE, *i*-PrOH/hexane = 2/98, v = 1.0 mL/min, λ = 254 nm

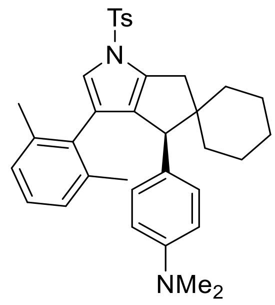

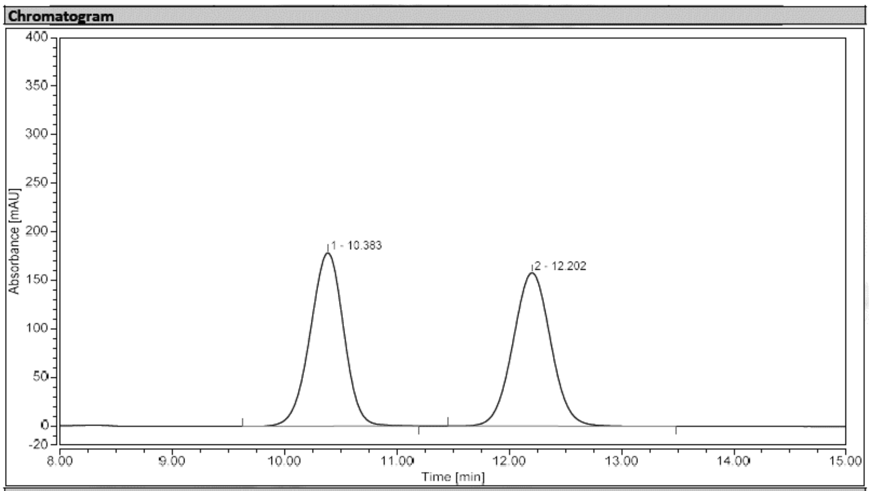

Integration Results							
No.	Peak Name	Retention Time min	Area mAU*min	Height mAU	Relative Area %	Relative Height %	Amount n.a.
1		10.383	61.722	178.498	50.13	53.07	n.a.
2		12.202	61.410	157.843	49.87	46.93	n.a.
Total:			123.133	336.341	100.00	100.00	

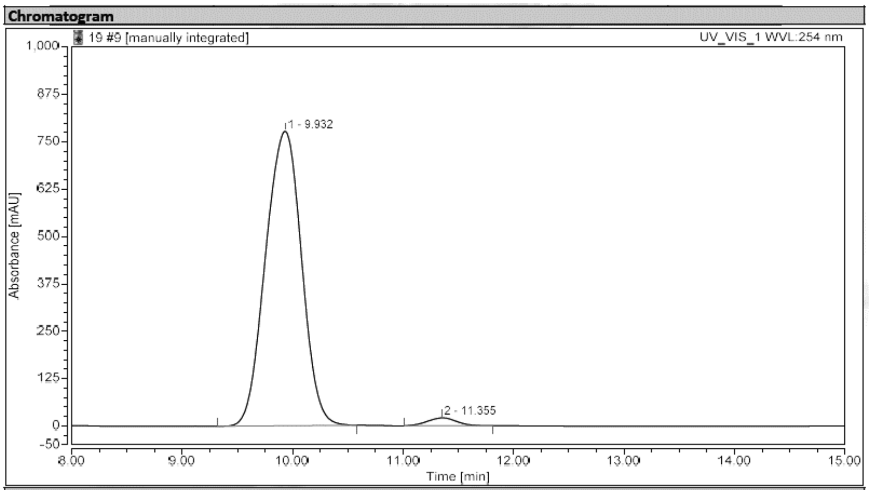

Integration Results							
No.	Peak Name	Retention Time min	Area mAU*min	Height mAU	Relative Area %	Relative Height %	Amount n.a.
1		9.932	293.581	777.060	97.93	97.42	n.a.
2		11.355	6.219	20.572	2.07	2.58	n.a.
Total:			299.800	797.633	100.00	100.00	

Supplementary Figure 100. HPLC spectrum for compound **(+)-2a**

3aa: IE, *i*-PrOH/hexane = 5/95, v = 1.0 mL/min, λ = 254 nm

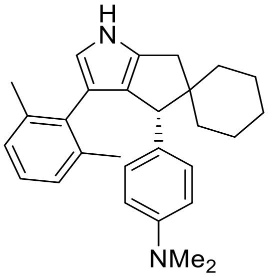

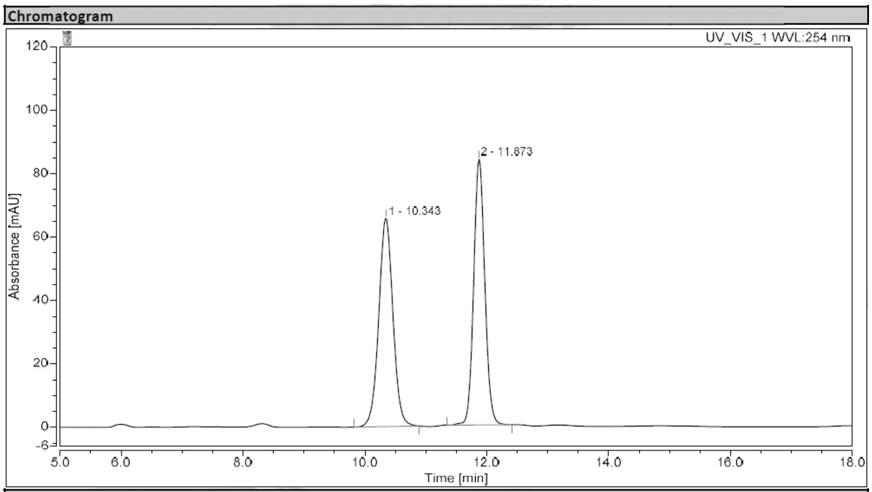

Integration Results

No.	Peak Name	Retention Time min	Area mAU*min	Height mAU	Relative Area %	Relative Height %	Amount n.a.
1		10.343	17.713	65.784	49.99	43.95	n.a.
2		11.873	17.718	83.885	50.01	56.05	n.a.
**Total:**			**35.431**	**149.669**	**100.00**	**100.00**	

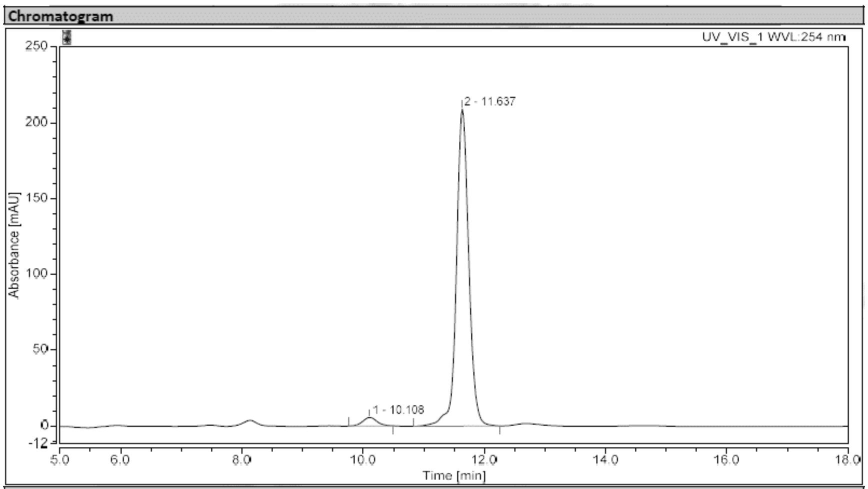

Integration Results

No.	Peak Name	Retention Time min	Area mAU*min	Height mAU	Relative Area %	Relative Height %	Amount n.a.
1		10.108	1.532	5.615	3.01	2.61	n.a.
2		11.637	49.417	209.319	96.99	97.39	n.a.
**Total:**			**50.948**	**214.934**	**100.00**	**100.00**	

Supplementary Figure 101. HPLC spectrum for compound **3aa**

3ab: ADH, *i*-PrOH/hexane = 5/95, v = 1.0 mL/min, λ = 254 nm

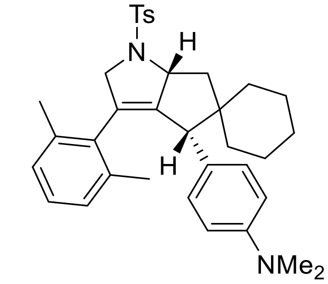

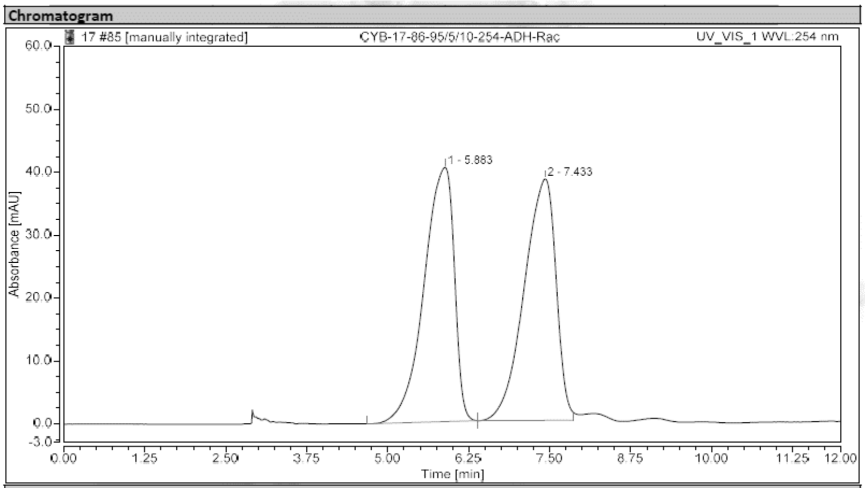

Integration Results

No.	Peak Name	Retention Time min	Area mAU*min	Height mAU	Relative Area %	Relative Height %	Amount n.a.
1		5.883	21.834	40.455	49.97	51.28	n.a.
2		7.433	21.860	38.430	50.03	48.72	n.a.
**Total:**			**43.695**	**78.885**	**100.00**	**100.00**	

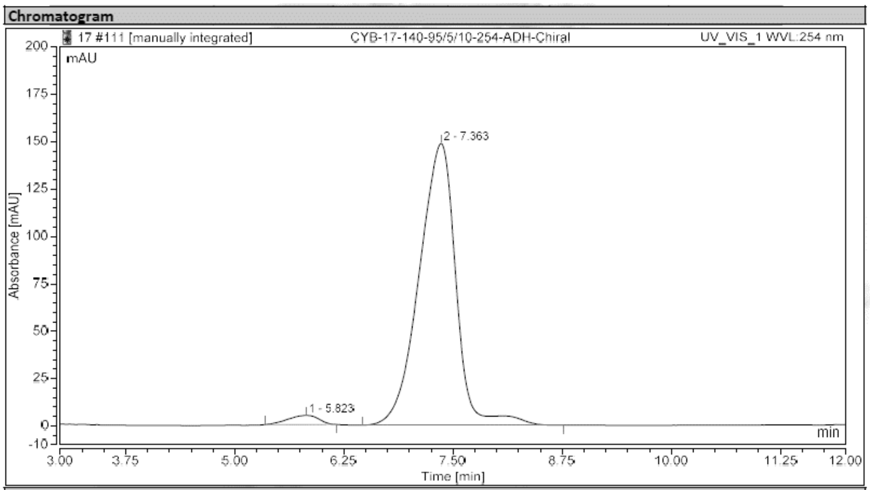

Integration Results

No.	Peak Name	Retention Time min	Area mAU*min	Height mAU	Relative Area %	Relative Height %	Amount n.a.
1		5.823	1.946	4.777	2.61	3.11	n.a.
2		7.363	72.621	148.757	97.39	96.89	n.a.
**Total:**			**74.567**	**153.534**	**100.00**	**100.00**	

Supplementary Figure 102. HPLC spectrum for compound **3ab**

3ac: IE, *i*-PrOH/hexane = 30/70, v = 1.0 mL/min, λ = 254 nm

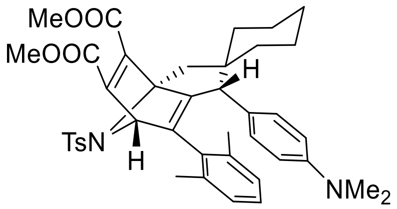

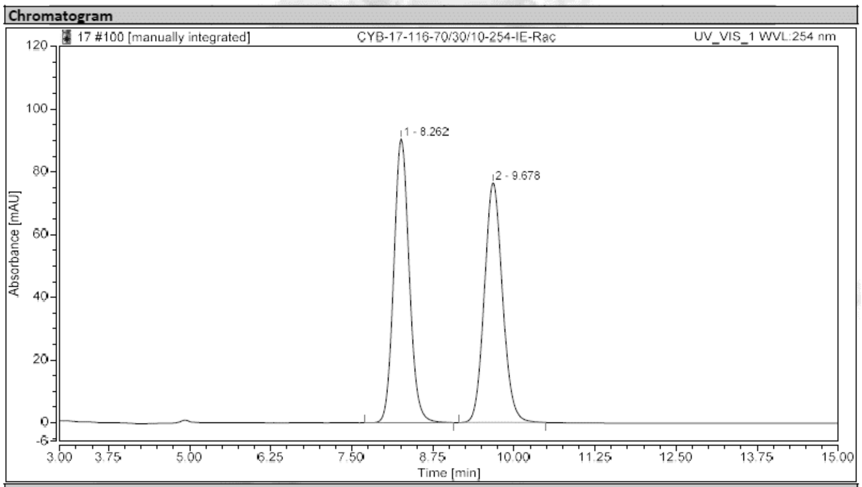

Integration Results							
No.	Peak Name	Retention Time min	Area mAU*min	Height mAU	Relative Area %	Relative Height %	Amount n.a.
1		8.262	24.890	90.552	50.08	54.19	n.a.
2		9.678	24.806	76.536	49.92	45.81	n.a.
Total:			49.696	167.089	100.00	100.00	

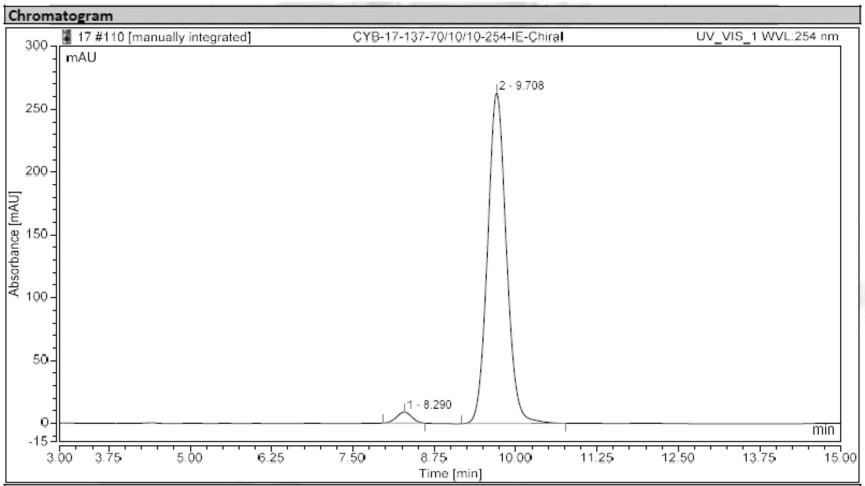

Integration Results							
No.	Peak Name	Retention Time min	Area mAU*min	Height mAU	Relative Area %	Relative Height %	Amount n.a.
1		8.290	2.332	8.715	2.58	3.20	n.a.
2		9.708	88.050	263.360	97.42	96.80	n.a.
Total:			90.382	272.074	100.00	100.00	

Supplementary Figure 103. HPLC spectrum for compound **3ac**

3ad: IE, *i*-PrOH/hexane = 5/95, v = 1.0 mL/min, λ = 254 nm

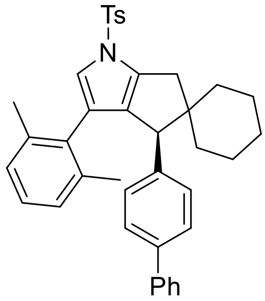

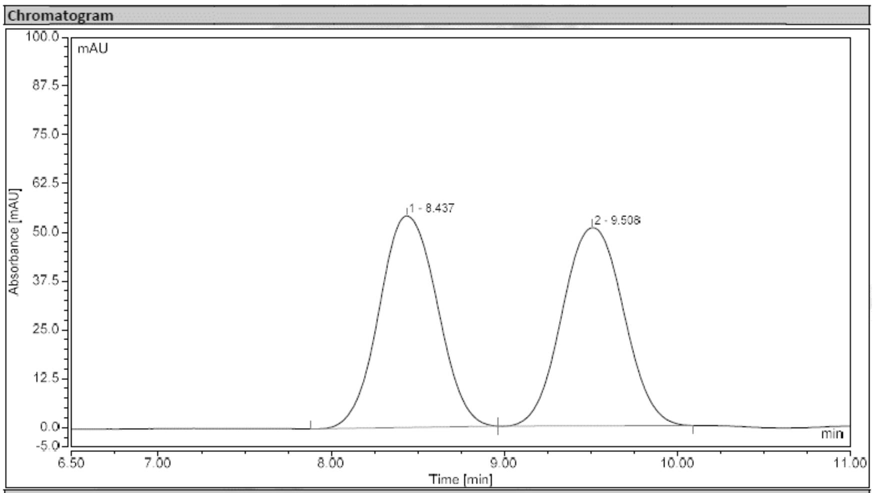

Integration Results

No.	Peak Name	Retention Time min	Area mAU*min	Height mAU	Relative Area %	Relative Height %	Amount n.a.
1		8.437	21.091	54.219	50.23	51.64	n.a.
2		9.508	20.900	50.773	49.77	48.36	n.a.
Total:			**41.991**	**104.992**	**100.00**	**100.00**	

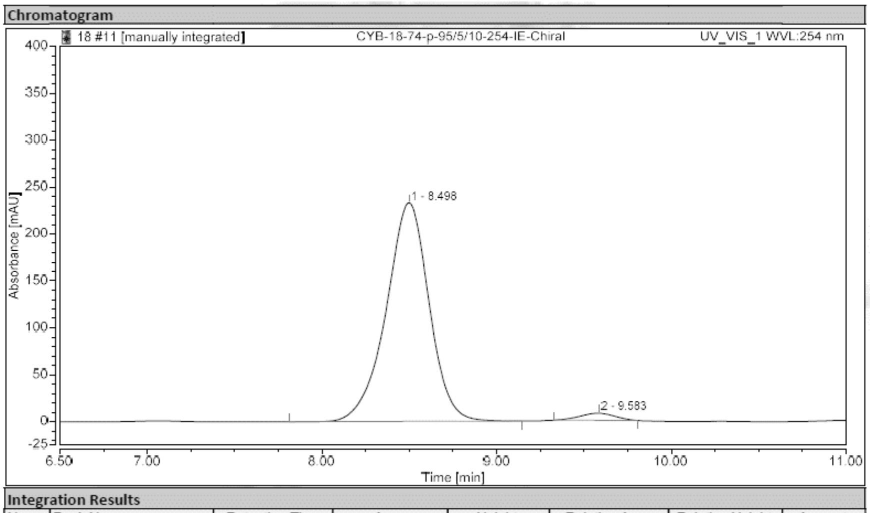

Integration Results

No.	Peak Name	Retention Time min	Area mAU*min	Height mAU	Relative Area %	Relative Height %	Amount n.a.
1		8.498	66.352	233.054	97.44	96.85	n.a.
2		9.583	1.746	7.573	2.56	3.15	n.a.
Total:			**68.098**	**240.627**	**100.00**	**100.00**	

Supplementary Figure 104. HPLC spectrum for compound **3ad**

3fa: ADH, *i*-PrOH/hexane = 10/90, v = 1.0 mL/min, λ = 254 nm

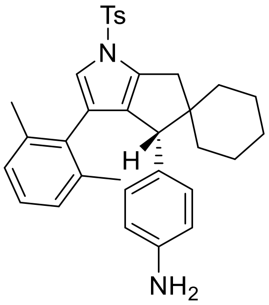

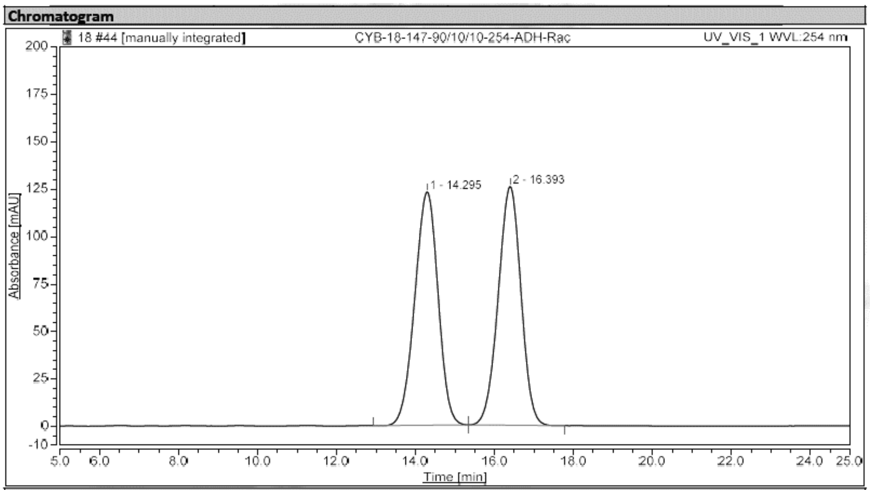

Integration Results

No.	Peak Name	Retention Time min	Area mAU*min	Height mAU	Relative Area %	Relative Height %	Amount n.a.
1		14.295	83.927	123.070	49.98	49.44	n.a.
2		16.393	83.978	125.861	50.02	50.56	n.a.
**Total:**			**167.905**	**248.931**	**100.00**	**100.00**	

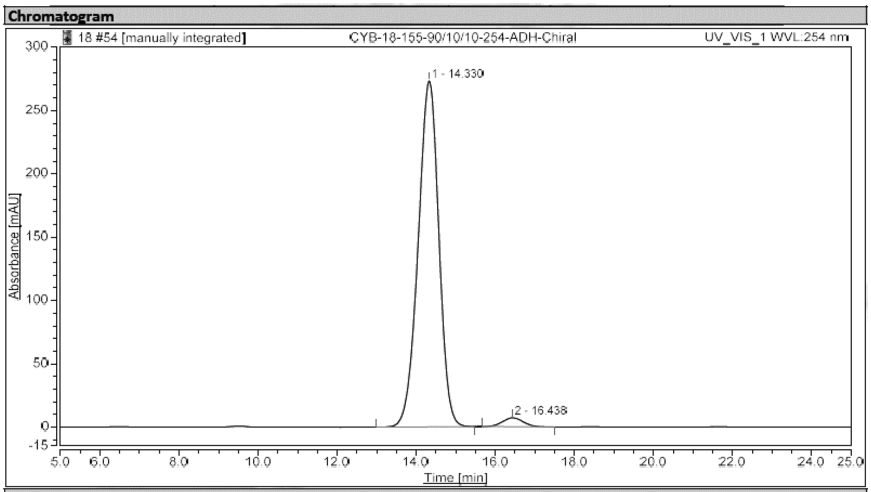

Integration Results

No.	Peak Name	Retention Time min	Area mAU*min	Height mAU	Relative Area %	Relative Height %	Amount n.a.
1		14.330	165.591	273.320	97.47	97.48	n.a.
2		16.438	4.297	7.064	2.53	2.52	n.a.
**Total:**			**169.888**	**280.384**	**100.00**	**100.00**	

Supplementary Figure 105. HPLC spectrum for compound **3fa**

3fb: ODH, *i*-PrOH/hexane = 5/95, v = 1.0 mL/min, λ = 254 nm

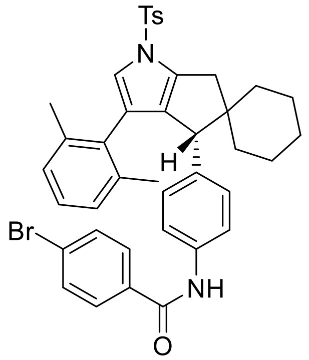

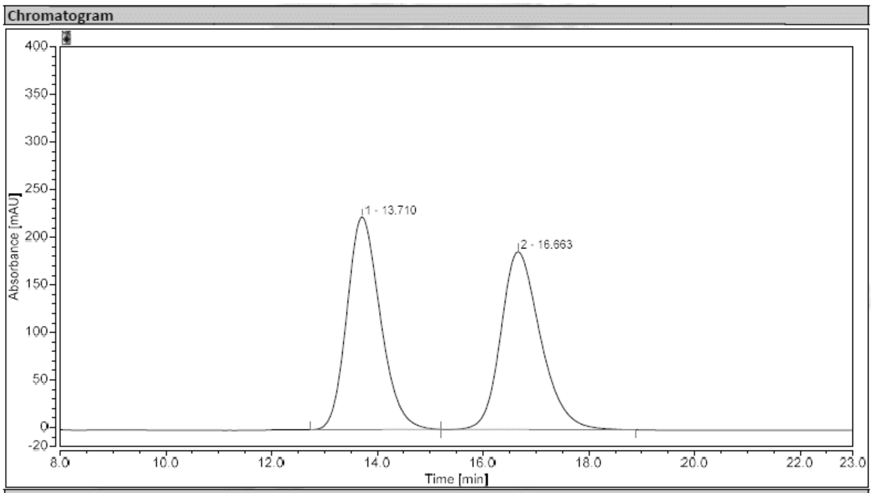

Integration Results

No.	Peak Name	Retention Time min	Area mAU*min	Height mAU	Relative Area %	Relative Height %	Amount n.a.
1		13.710	163.721	223.291	50.19	54.45	n.a.
2		16.663	162.502	186.798	49.81	45.55	n.a.
Total:			326.223	410.089	100.00	100.00	

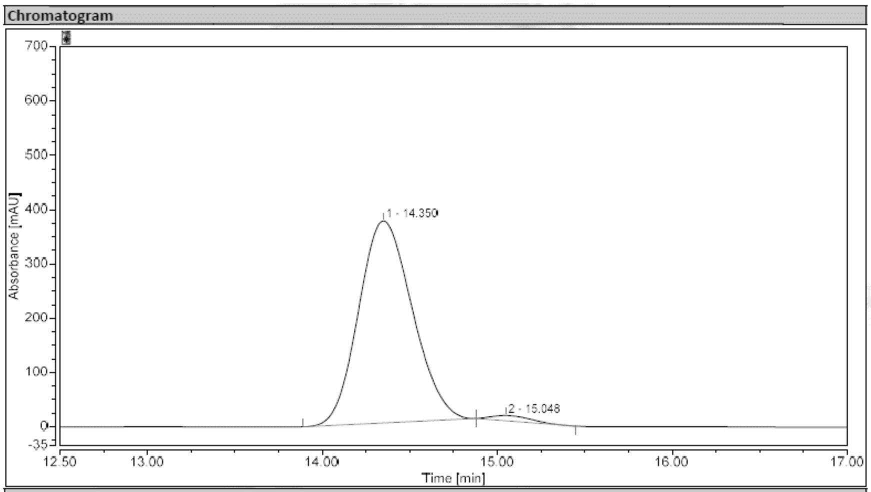

Integration Results

No.	Peak Name	Retention Time min	Area mAU*min	Height mAU	Relative Area %	Relative Height %	Amount n.a.
1		14.350	132.238	372.220	98.08	97.45	n.a.
2		15.048	2.592	9.729	1.92	2.55	n.a.
Total:			134.830	381.949	100.00	100.00	

Supplementary Figure 106. HPLC spectrum for compound **3fb**

3fc: IE, *i*-PrOH/hexane = 20/80, v = 1.0 mL/min, λ = 254 nm

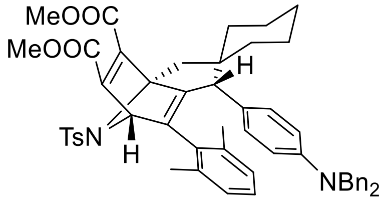

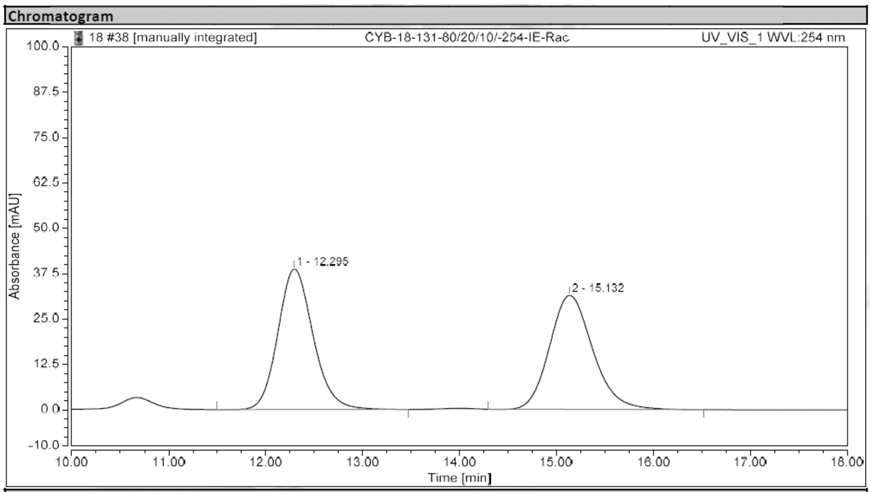

Integration Results

No.	Peak Name	Retention Time min	Area mAU*min	Height mAU	Relative Area %	Relative Height %	Amount n.a.
1		12.295	16.291	38.715	50.09	55.19	n.a.
2		15.132	16.231	31.437	49.91	44.81	n.a.
**Total:**			**32.521**	**70.153**	**100.00**	**100.00**	

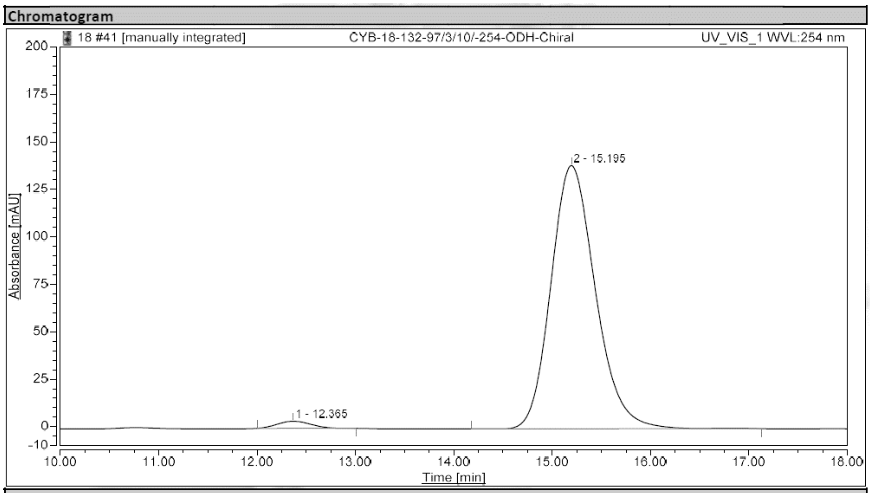

Integration Results

No.	Peak Name	Retention Time min	Area mAU*min	Height mAU	Relative Area %	Relative Height %	Amount n.a.
1		12.365	1.460	3.741	1.97	2.63	n.a.
2		15.195	72.650	138.661	98.03	97.37	n.a.
**Total:**			**74.110**	**142.402**	**100.00**	**100.00**	

Supplementary Figure 107. HPLC spectrum for compound **3fc**

9. NMR Spectra

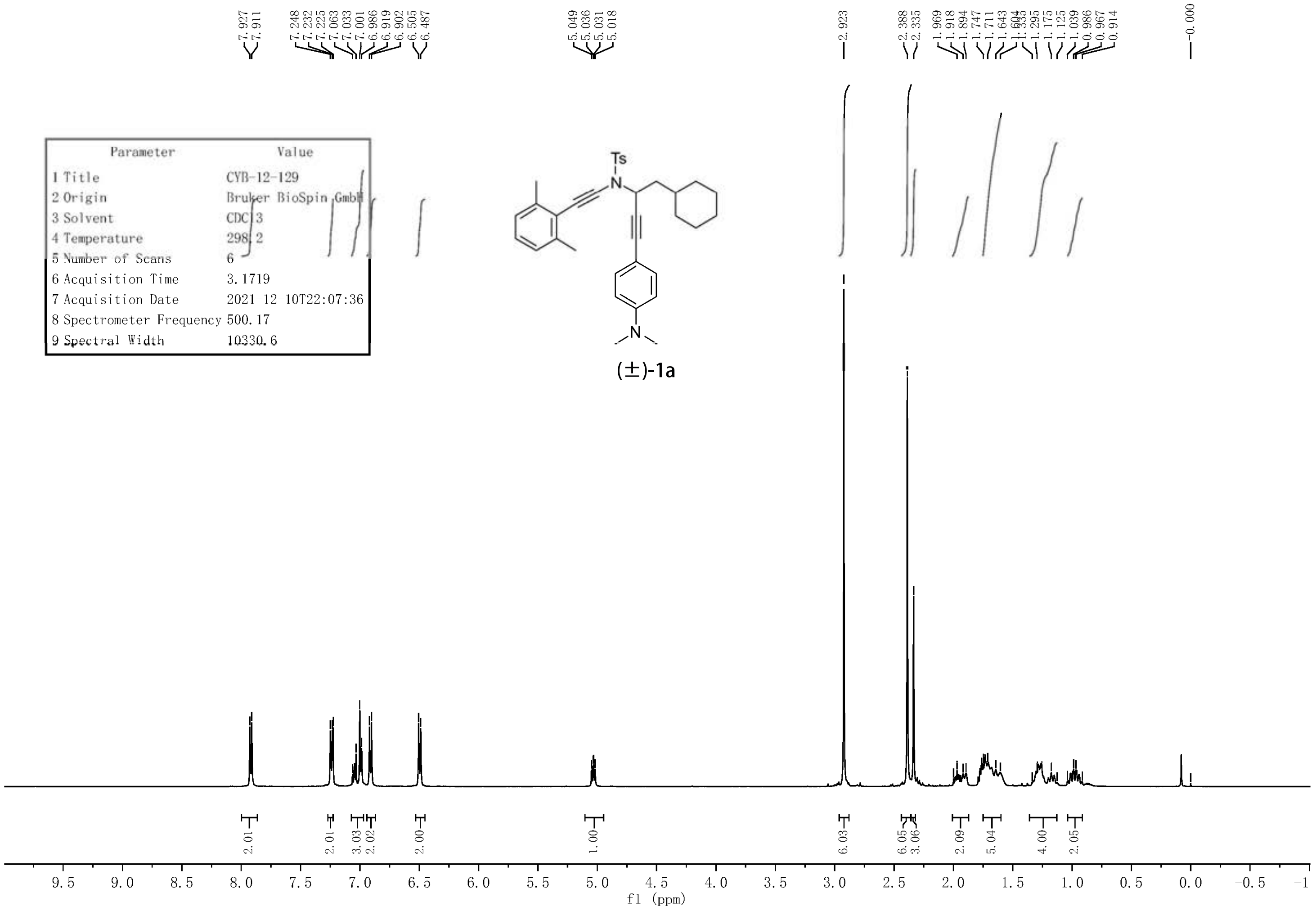

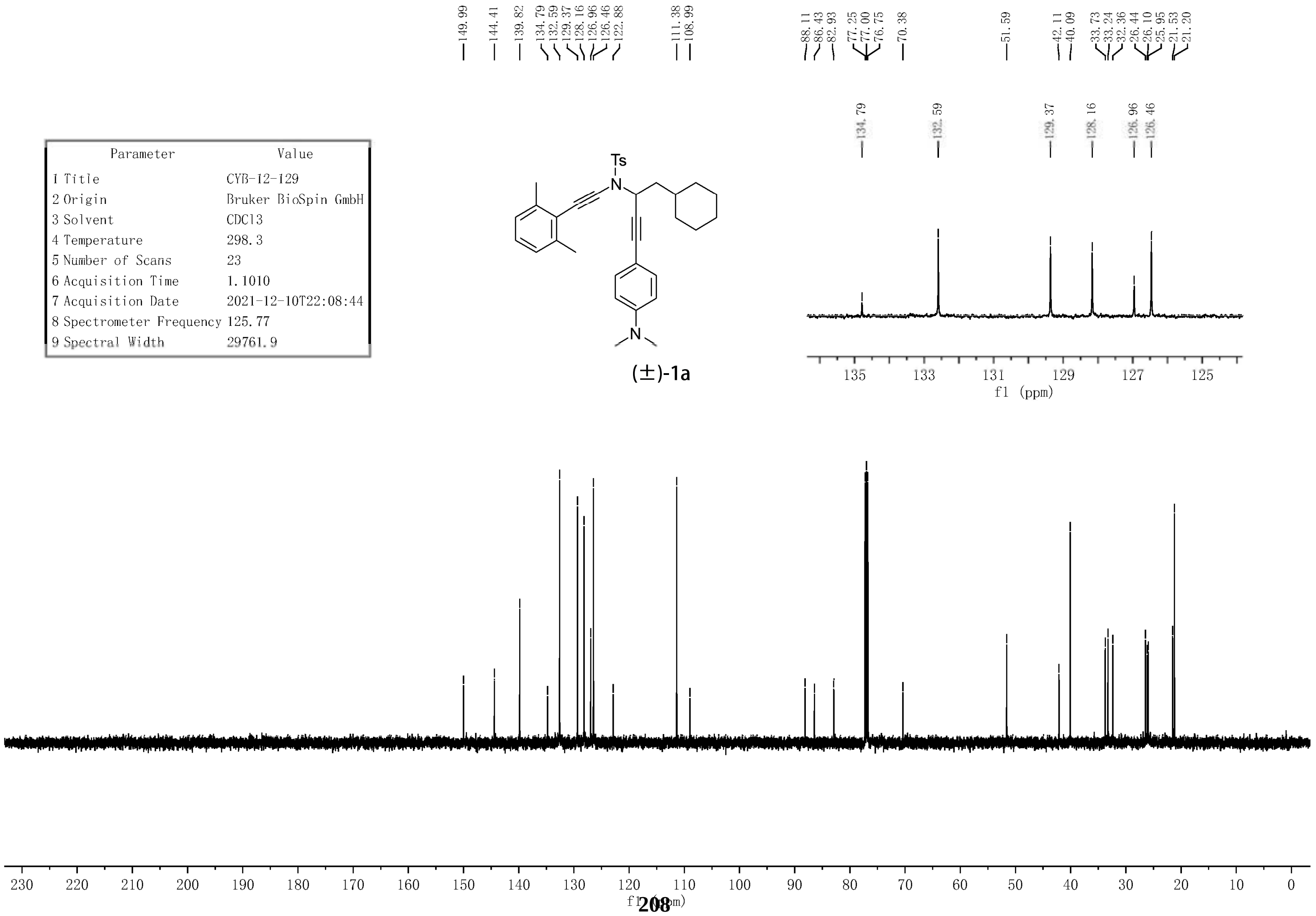

Supplementary Figure 108. ^{1}H and ^{13}C NMR spectra for (±)-1a

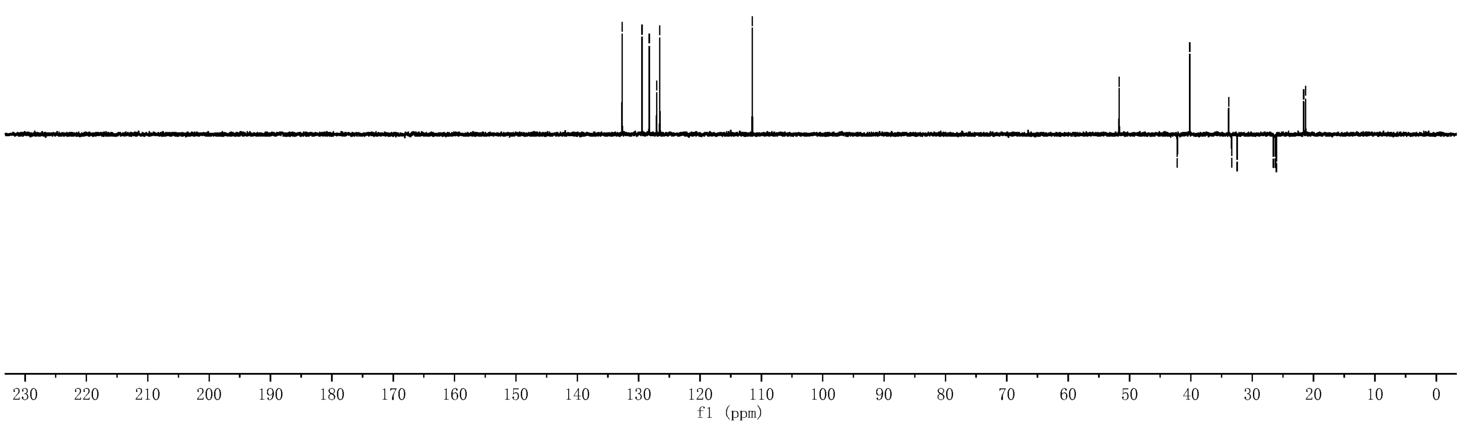

Parameter	Value
1 Title	CYB-12-129
2 Origin	Bruker BioSpin GmbH
3 Solvent	CDCl3
4 Temperature	298.6
5 Number of Scans	8
6 Acquisition Time	1.1010
7 Acquisition Date	2021-12-10T22:10:56
8 Spectrometer Frequency	125.77
9 Spectral Width	29761.9

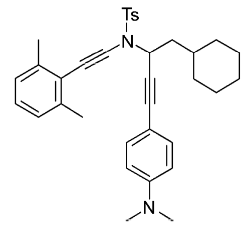

(±)-1a

Supplementary Figure 109. DEPT-135 NMR spectra for (±)-1a

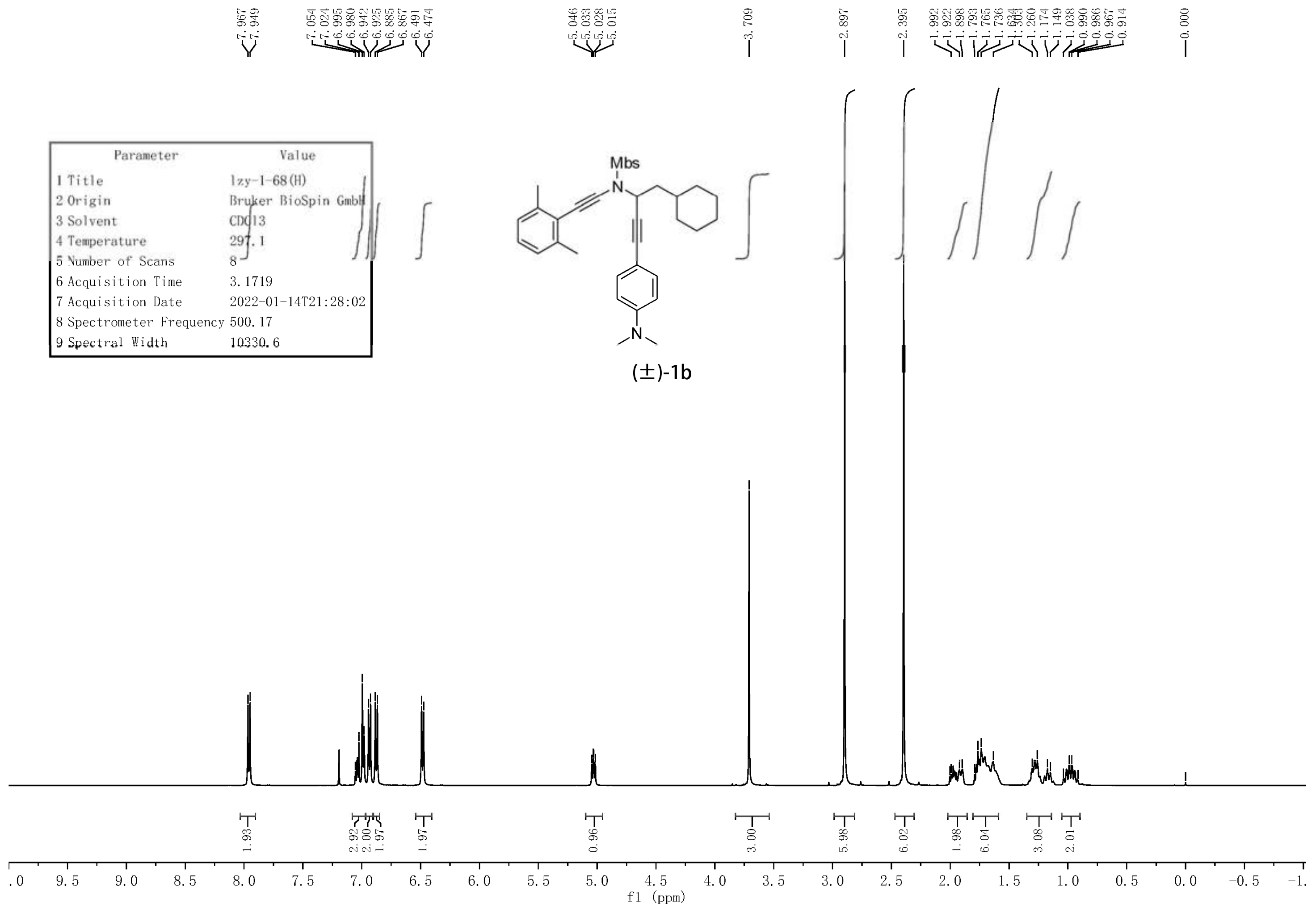

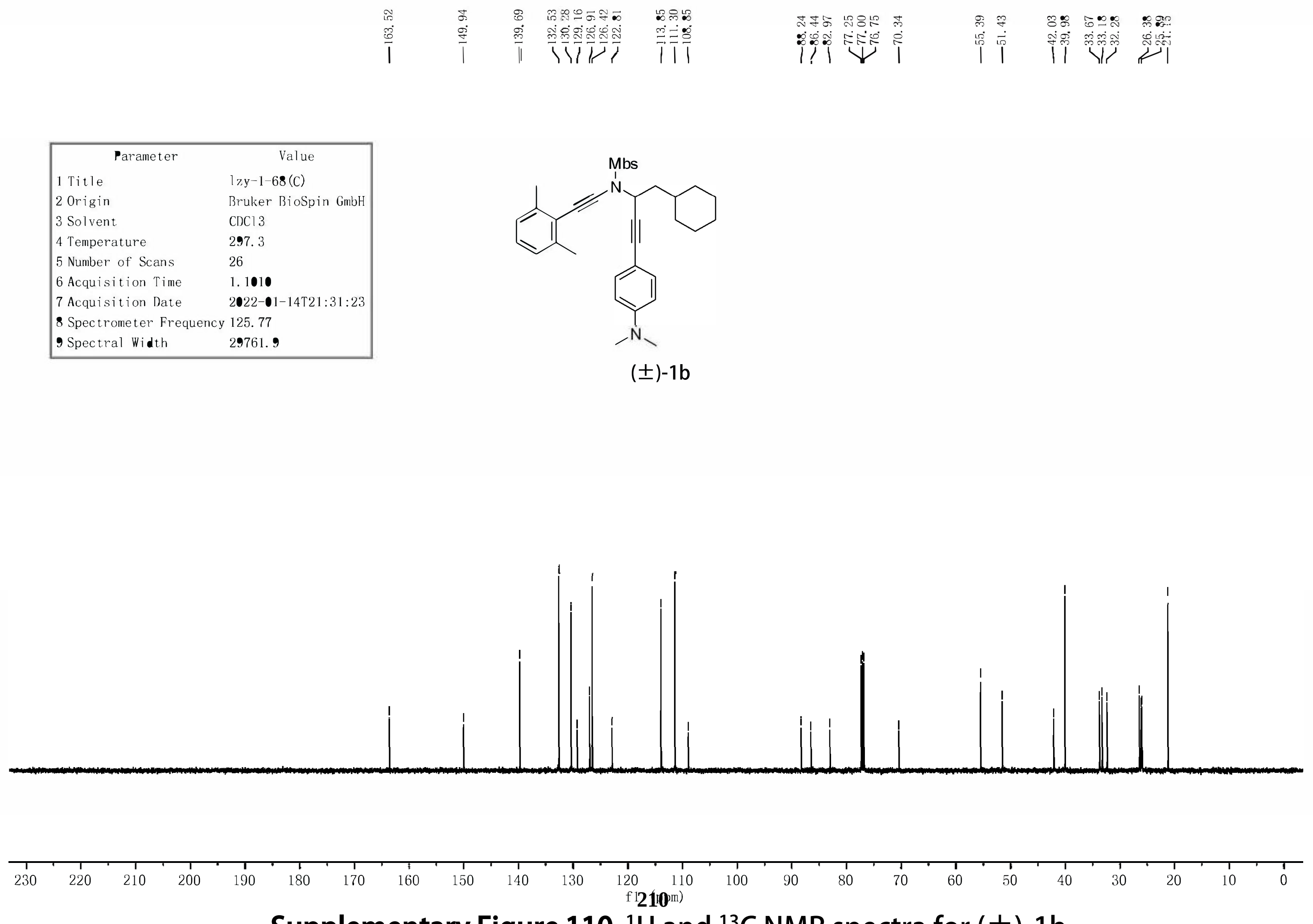

Supplementary Figure 110. ^{1}H and ^{13}C NMR spectra for (±)-1b

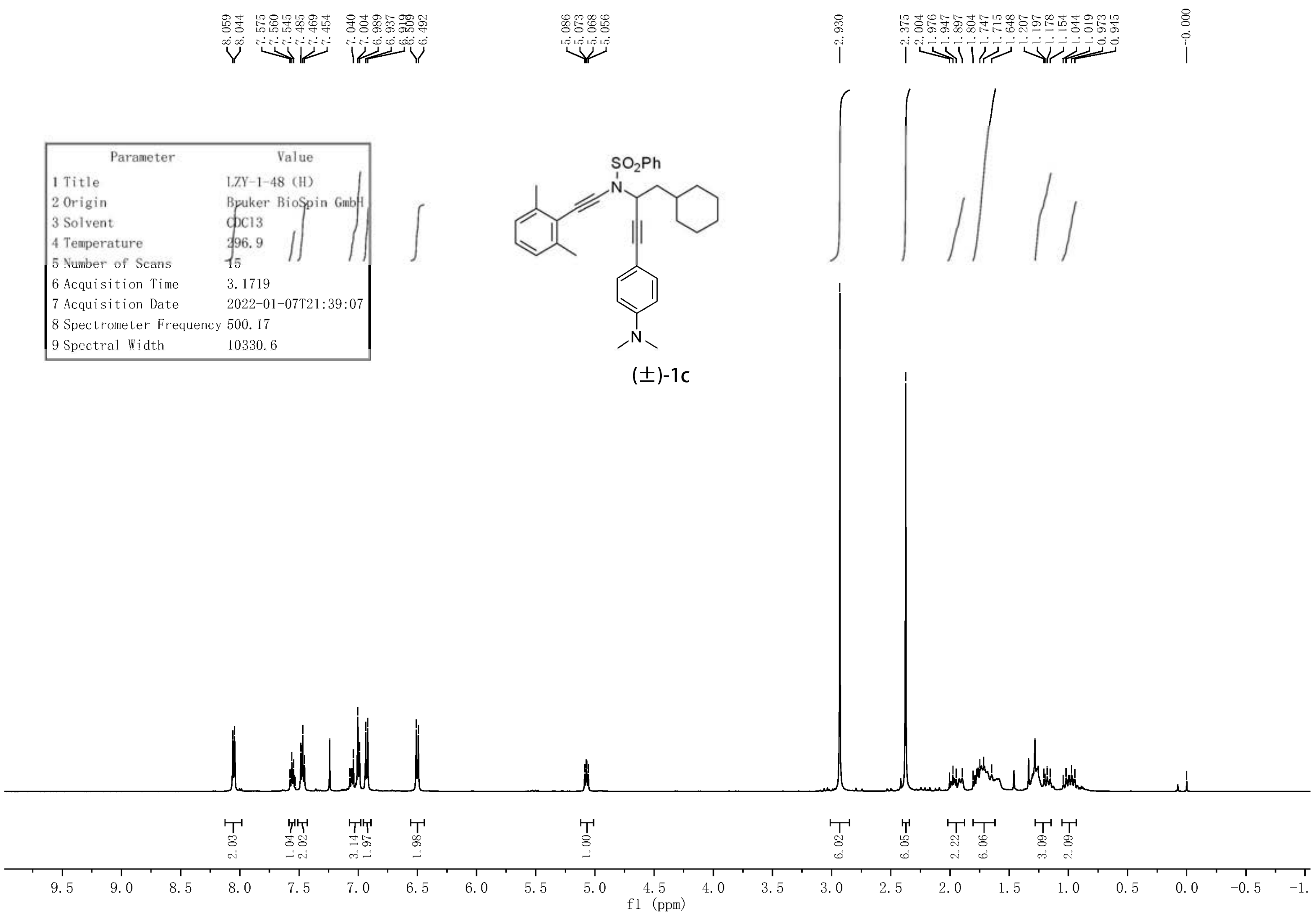

Parameter	Value
1 Title	LZY-1-48 (H)
2 Origin	Bruker BioSpin GmbH
3 Solvent	CDCl3
4 Temperature	296.9
5 Number of Scans	15
6 Acquisition Time	3.1719
7 Acquisition Date	2022-01-07T21:39:07
8 Spectrometer Frequency	500.17
9 Spectral Width	10330.6

(±)-1c

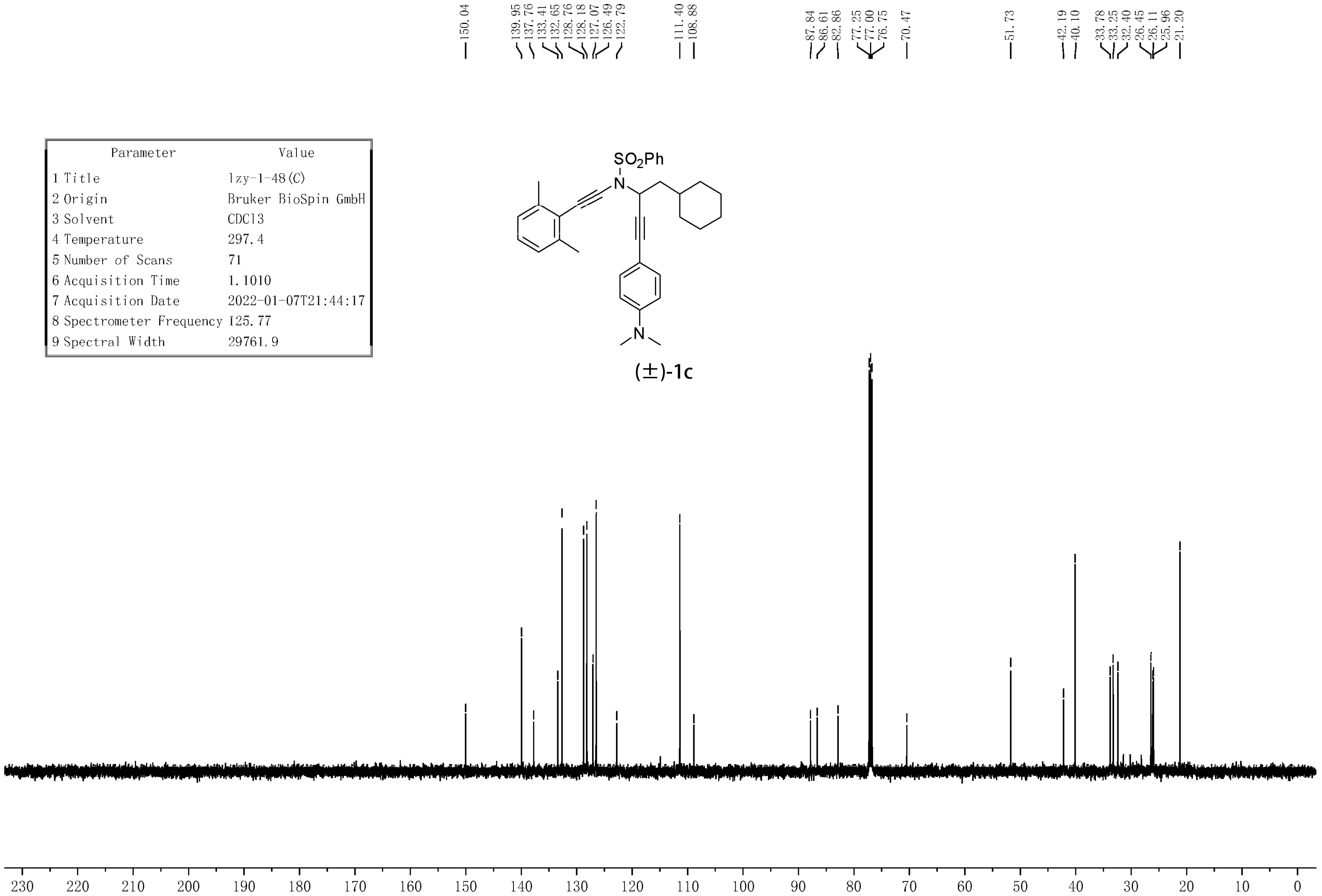

Parameter	Value
1 Title	lzy-1-48(C)
2 Origin	Bruker BioSpin GmbH
3 Solvent	CDCl3
4 Temperature	297.4
5 Number of Scans	71
6 Acquisition Time	1.1010
7 Acquisition Date	2022-01-07T21:44:17
8 Spectrometer Frequency	125.77
9 Spectral Width	29761.9

(±)-1c

Supplementary Figure 111. ^{1}H and ^{13}C NMR spectra for (±)-1c

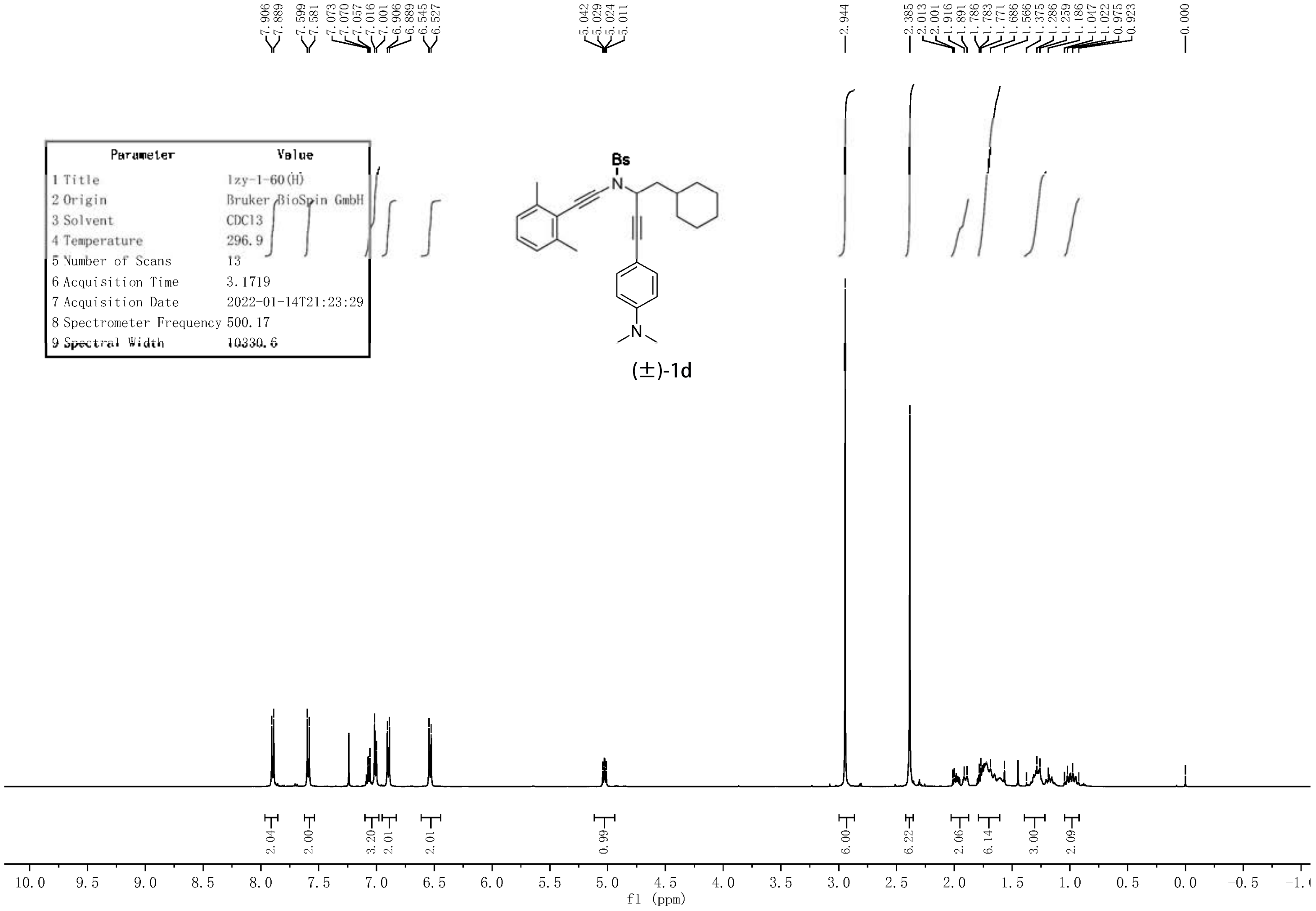

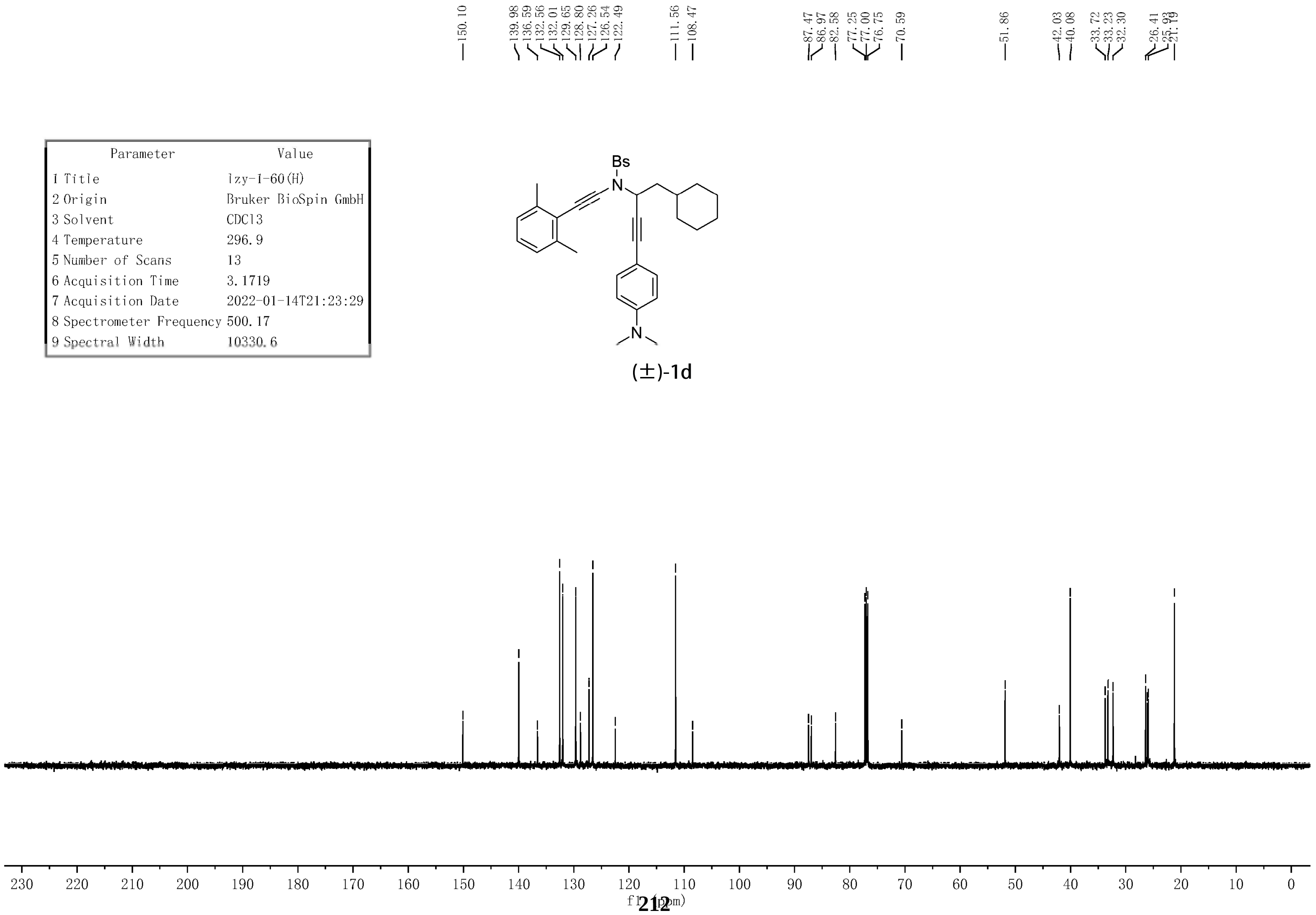

Supplementary Figure 112. ^{1}H and ^{13}C NMR spectra for (±)-1d

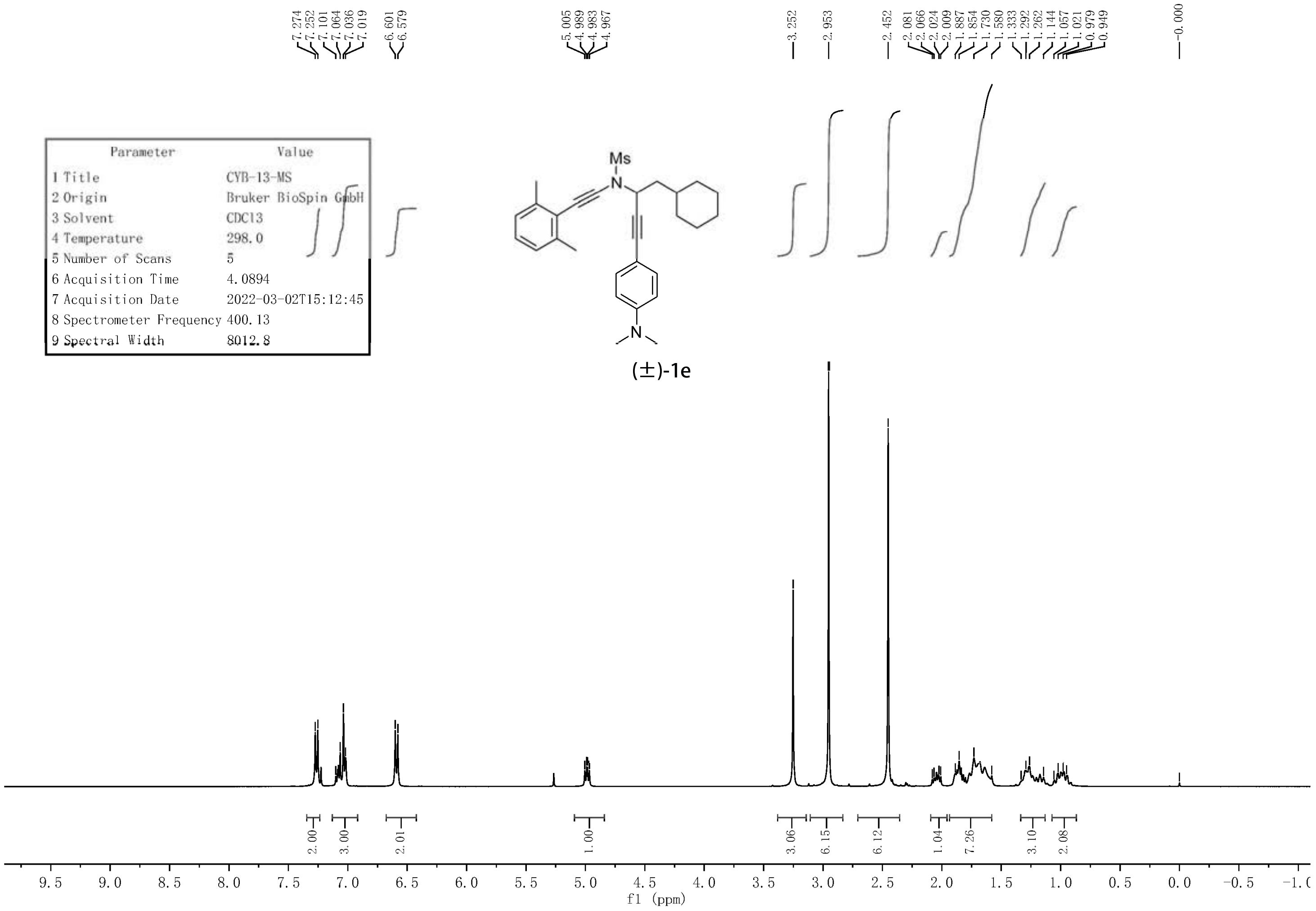

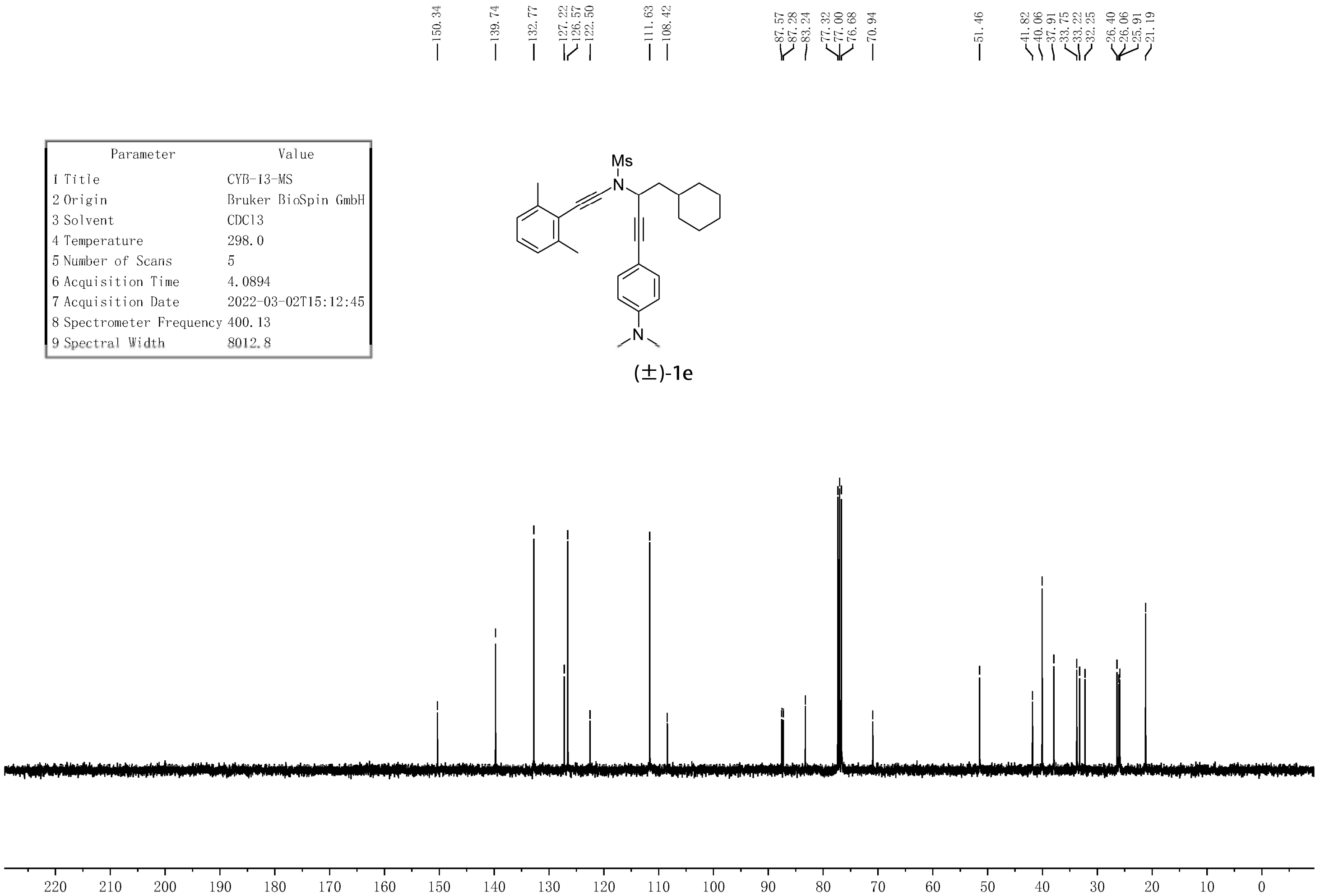

Supplementary Figure 113. ^{1}H and ^{13}C NMR spectra for (±)-1e

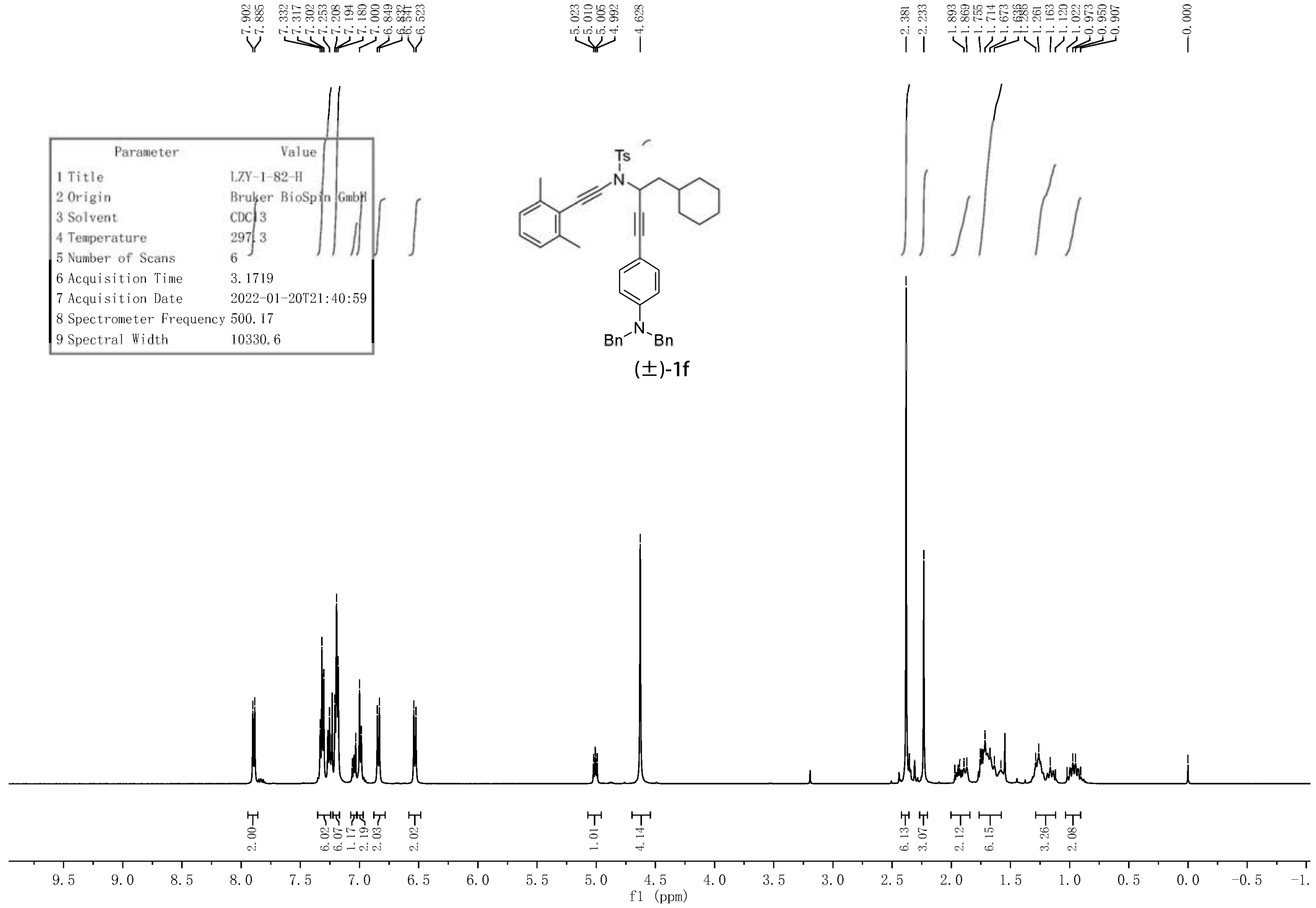

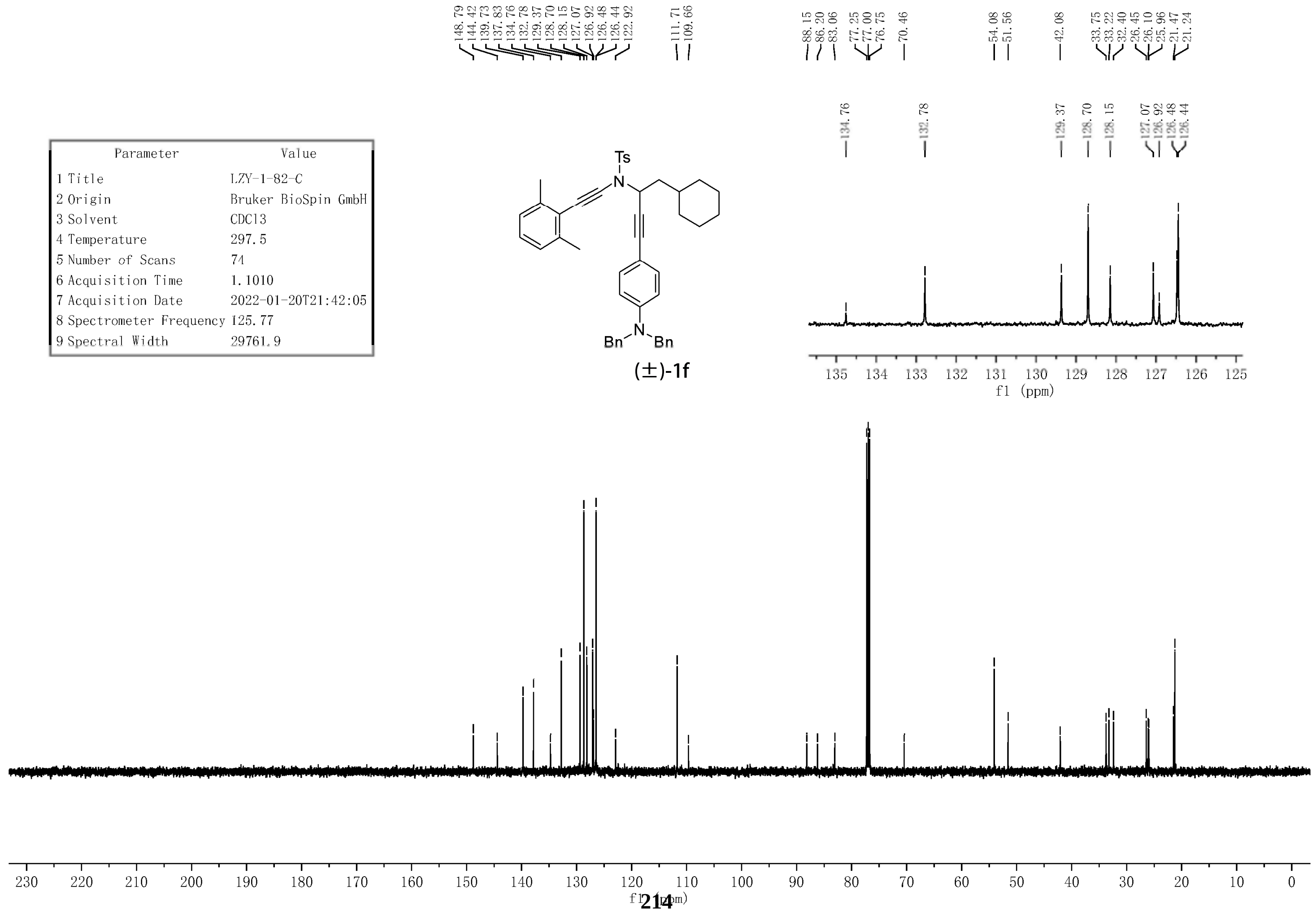

Supplementary Figure 114. ^{1}H and ^{13}C NMR spectra for (±)-1f

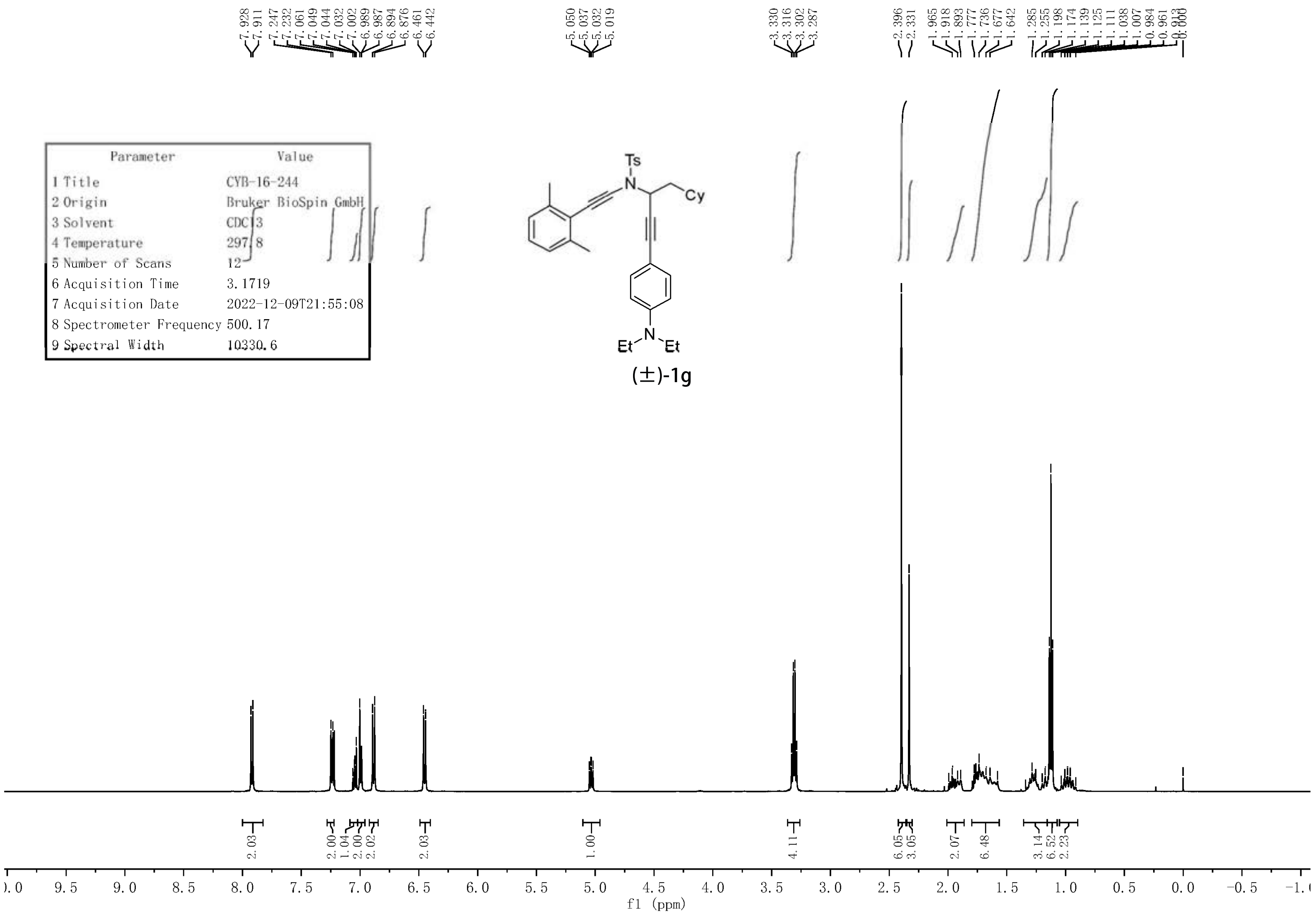

Parameter	Value
1 Title	CYB-16-244
2 Origin	Bruker BioSpin GmbH
3 Solvent	CDCl3
4 Temperature	297.8
5 Number of Scans	12
6 Acquisition Time	3.1719
7 Acquisition Date	2022-12-09T21:55:08
8 Spectrometer Frequency	500.17
9 Spectral Width	10330.6

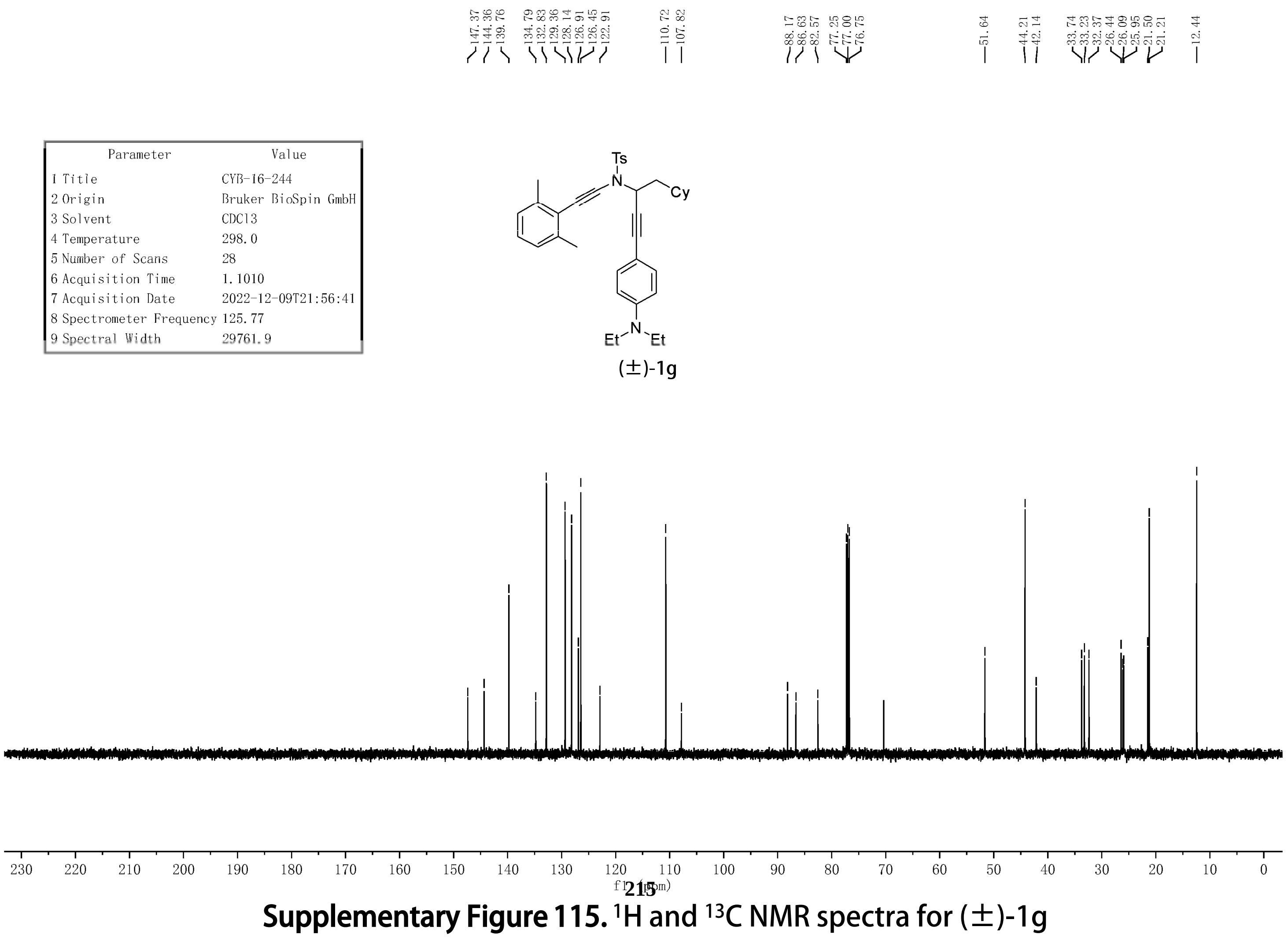

Parameter	Value
1 Title	CYB-16-244
2 Origin	Bruker BioSpin GmbH
3 Solvent	CDCl3
4 Temperature	298.0
5 Number of Scans	28
6 Acquisition Time	1.1010
7 Acquisition Date	2022-12-09T21:56:41
8 Spectrometer Frequency	125.77
9 Spectral Width	29761.9

Supplementary Figure 115. ^{1}H and ^{13}C NMR spectra for (±)-1g

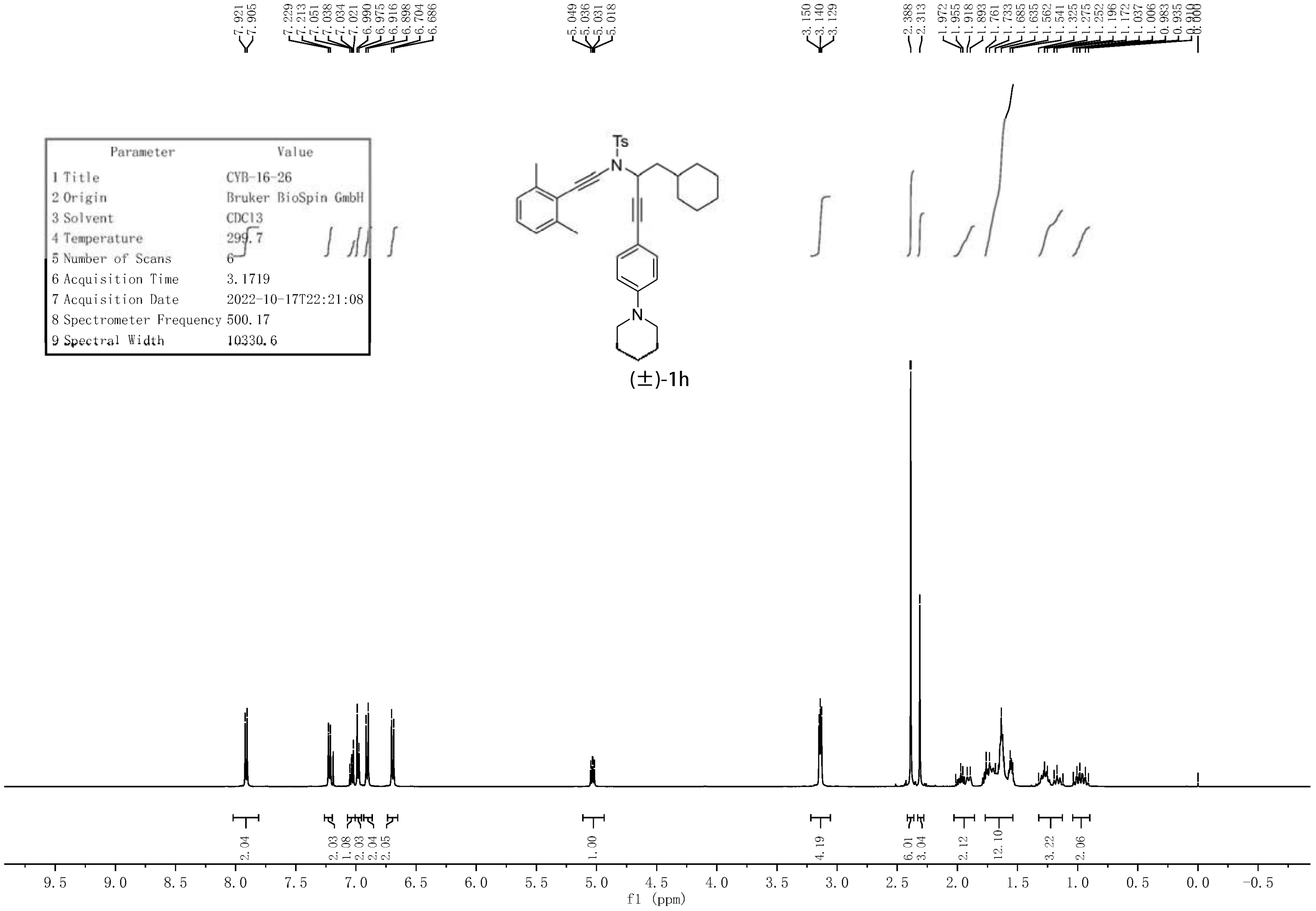

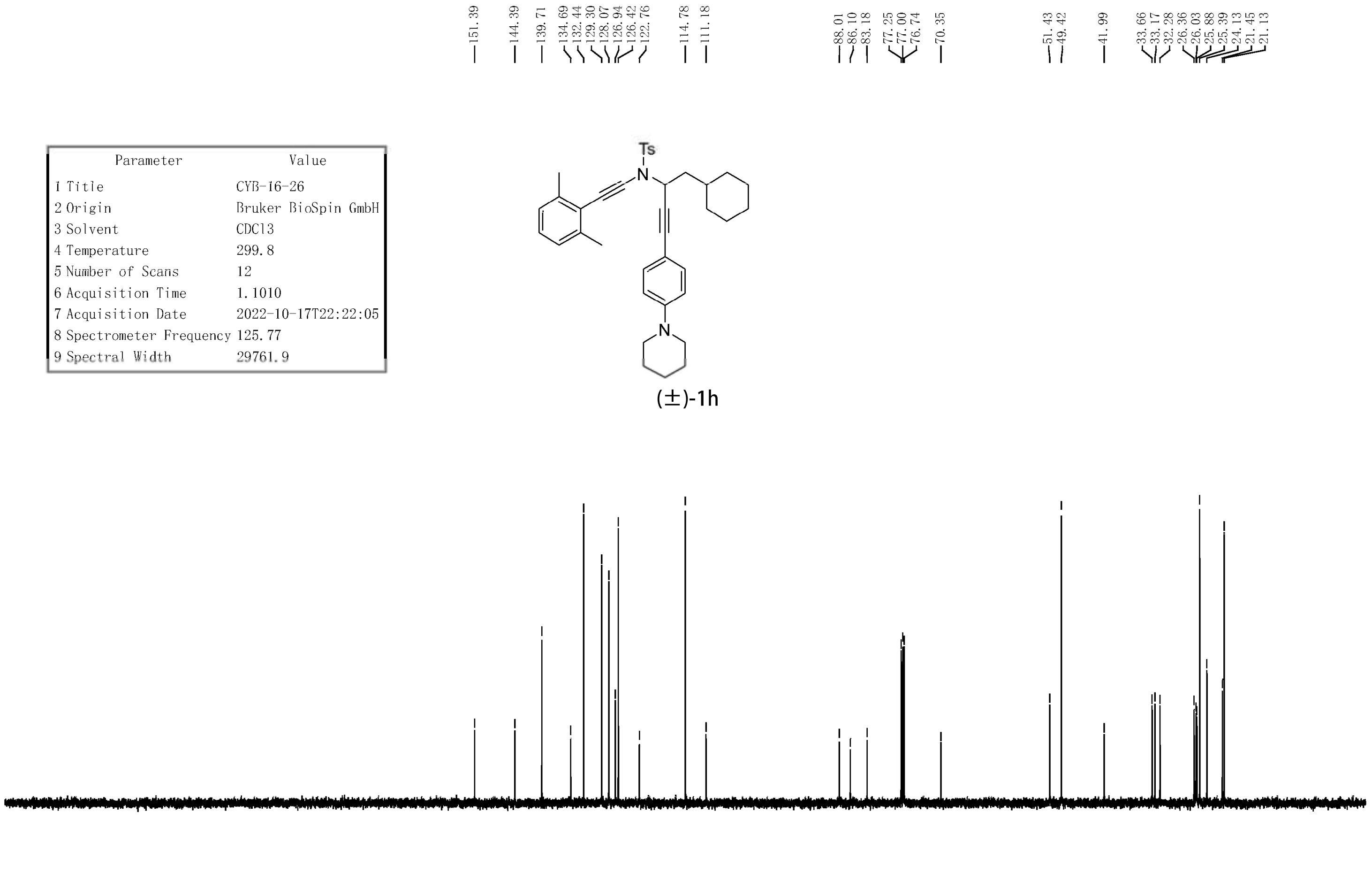

Supplementary Figure 116. ^{1}H and ^{13}C NMR spectra for (±)-1h

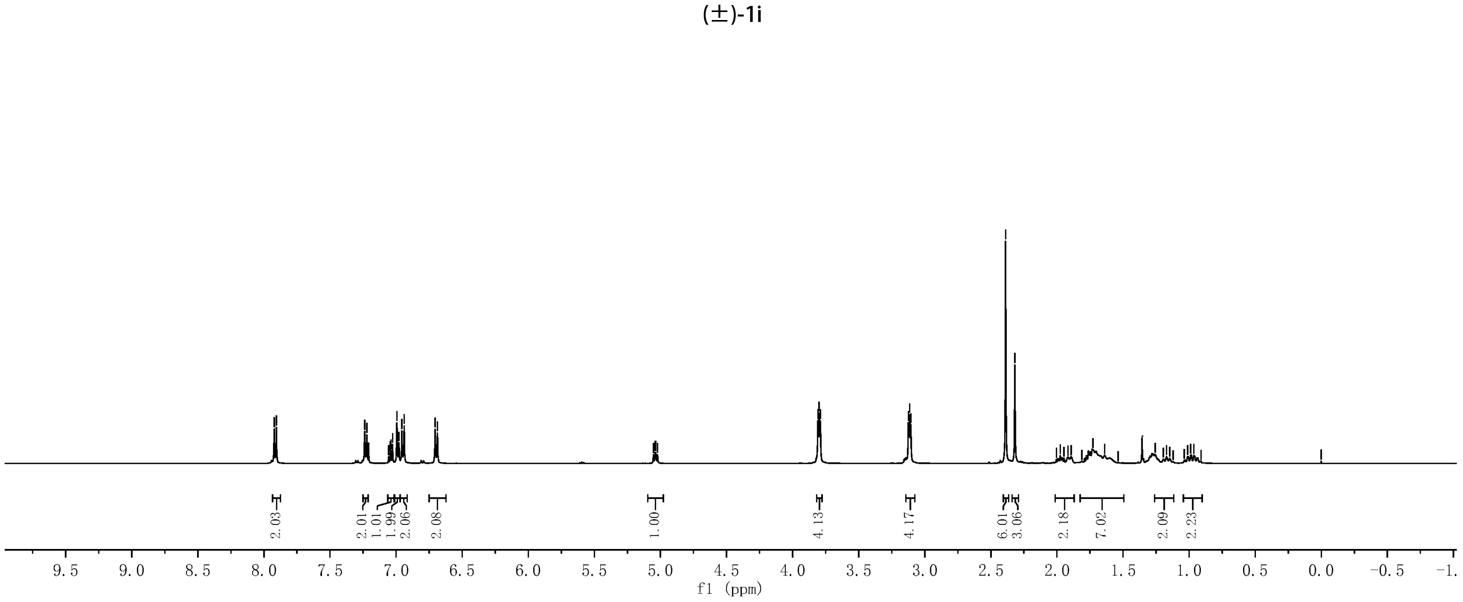

Parameter	Value
1 Title	CYB-17-13-500M
2 Origin	Bruker BioSpin GmbH
3 Solvent	CDCl3
4 Temperature	296.9
5 Number of Scans	11
6 Acquisition Time	3.1719
7 Acquisition Date	2022-12-13T22:09:53
8 Spectrometer Frequency	500.17
9 Spectral Width	10330.6

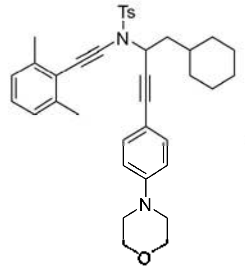

(±)-1i

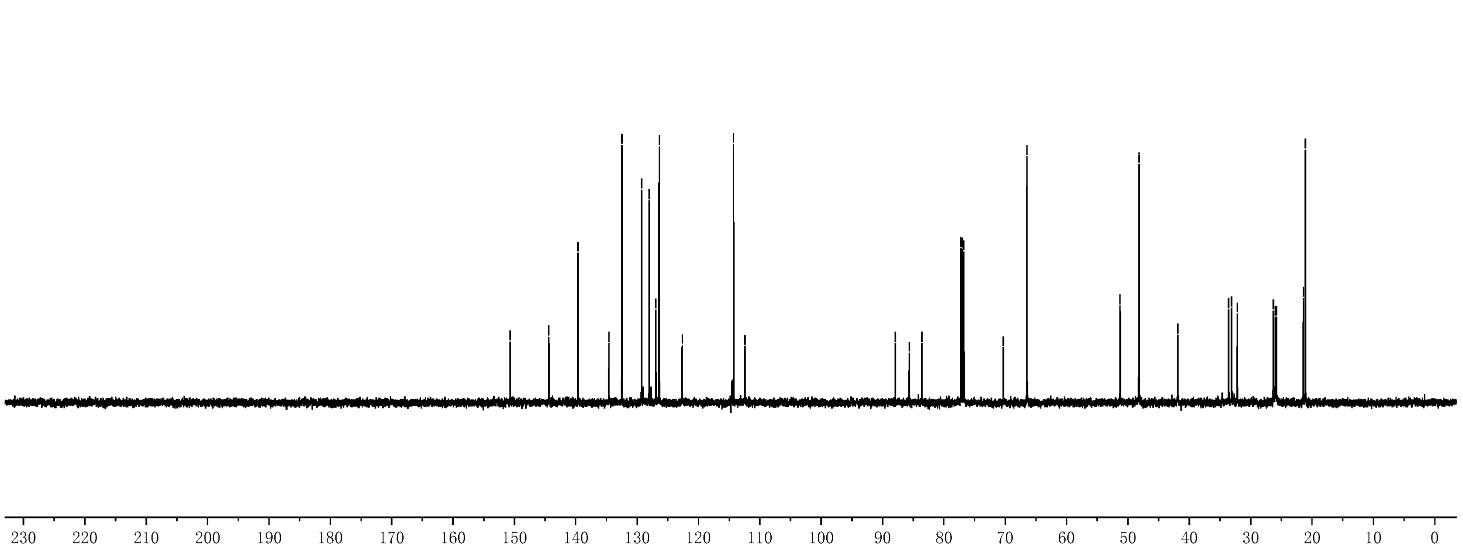

Parameter	Value
1 Title	CYB-17-13-500M
2 Origin	Bruker BioSpin GmbH
3 Solvent	CDCl3
4 Temperature	297.1
5 Number of Scans	16
6 Acquisition Time	1.1010
7 Acquisition Date	2022-12-13T22:11:09
8 Spectrometer Frequency	125.77
9 Spectral Width	29761.9

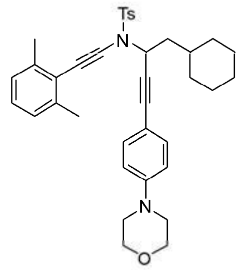

(±)-1i

Supplementary Figure 117. ^{1}H and ^{13}C NMR spectra for (±)-1i

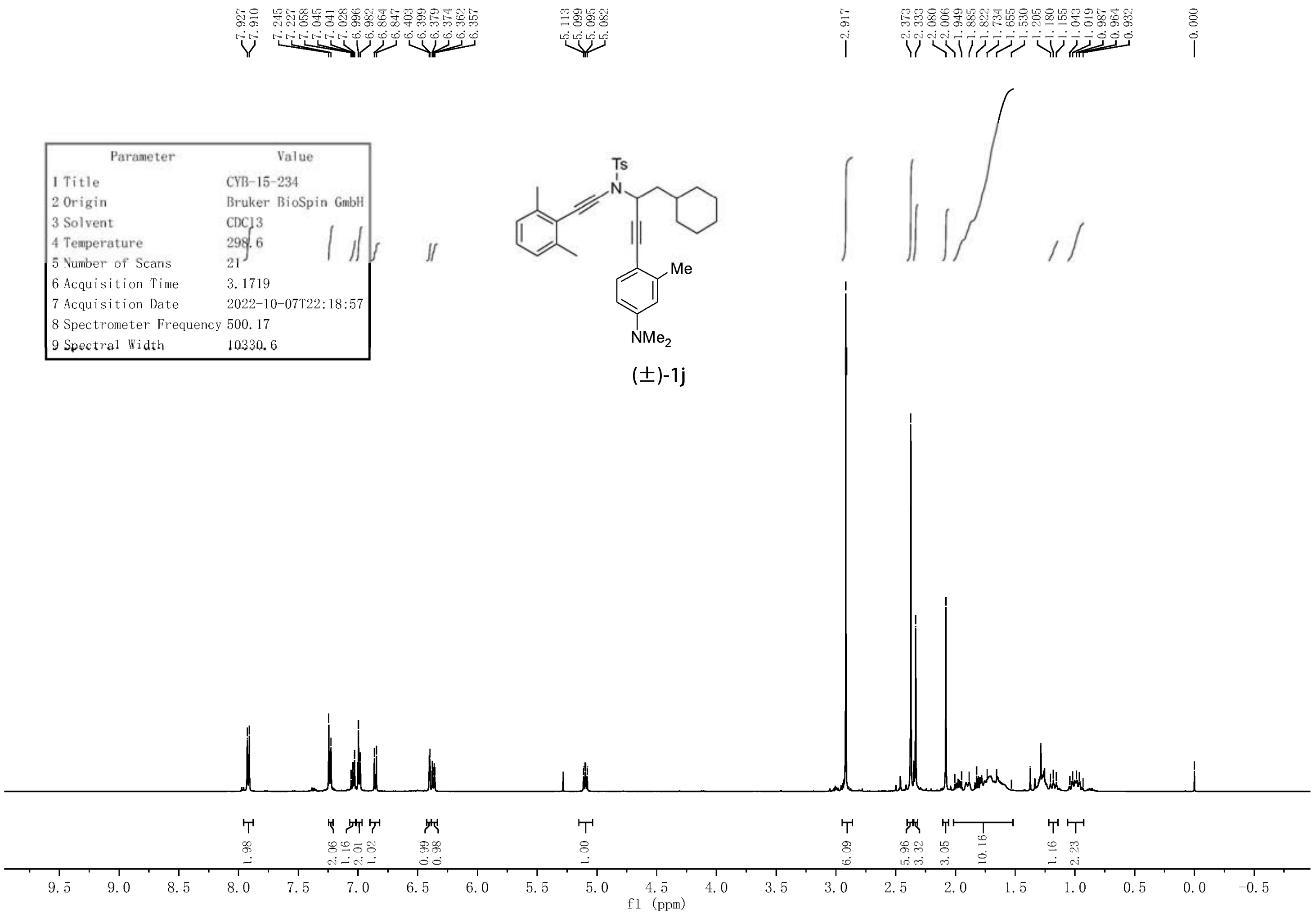

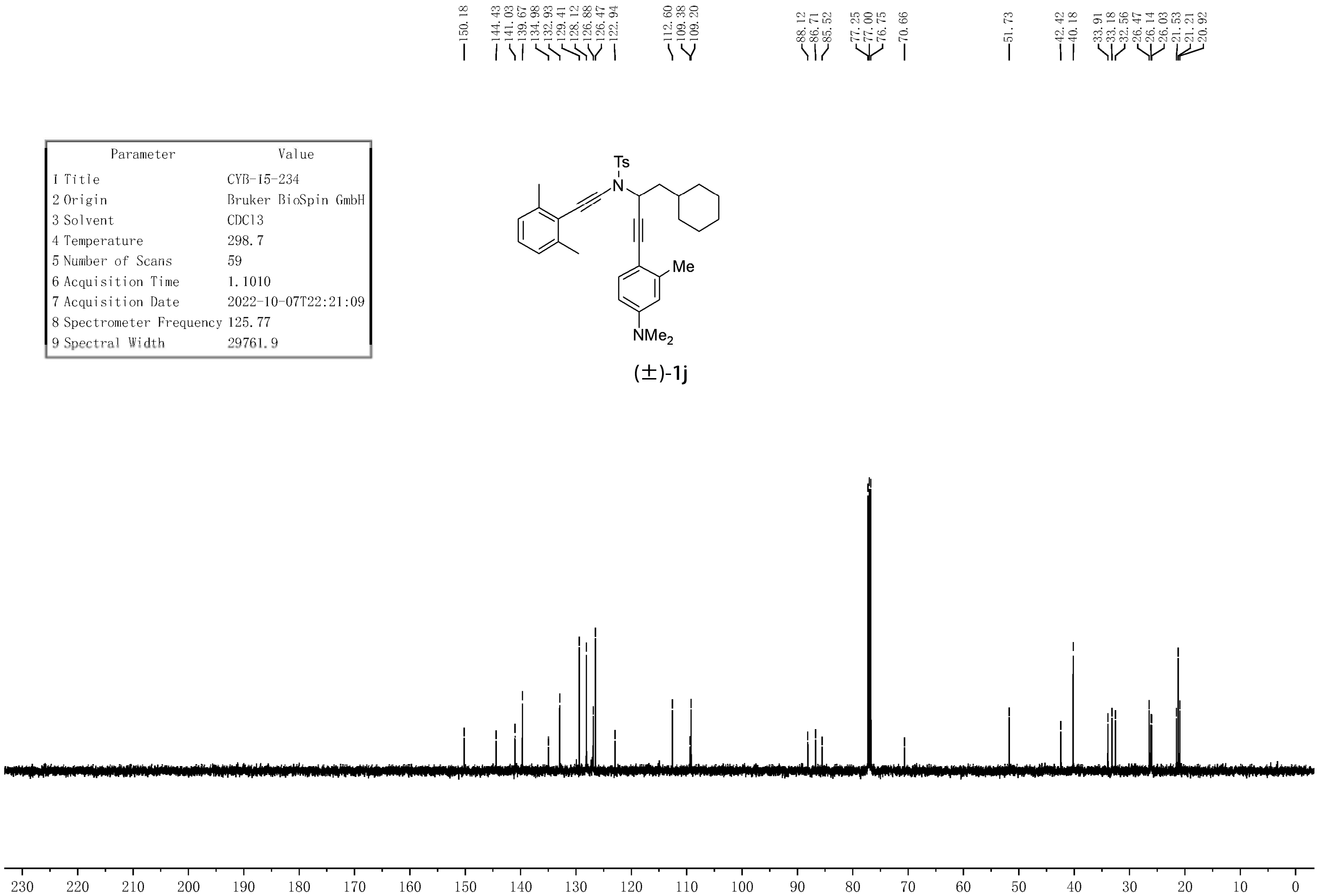

Supplementary Figure 118. ^{1}H and ^{13}C NMR spectra for (±)-1j

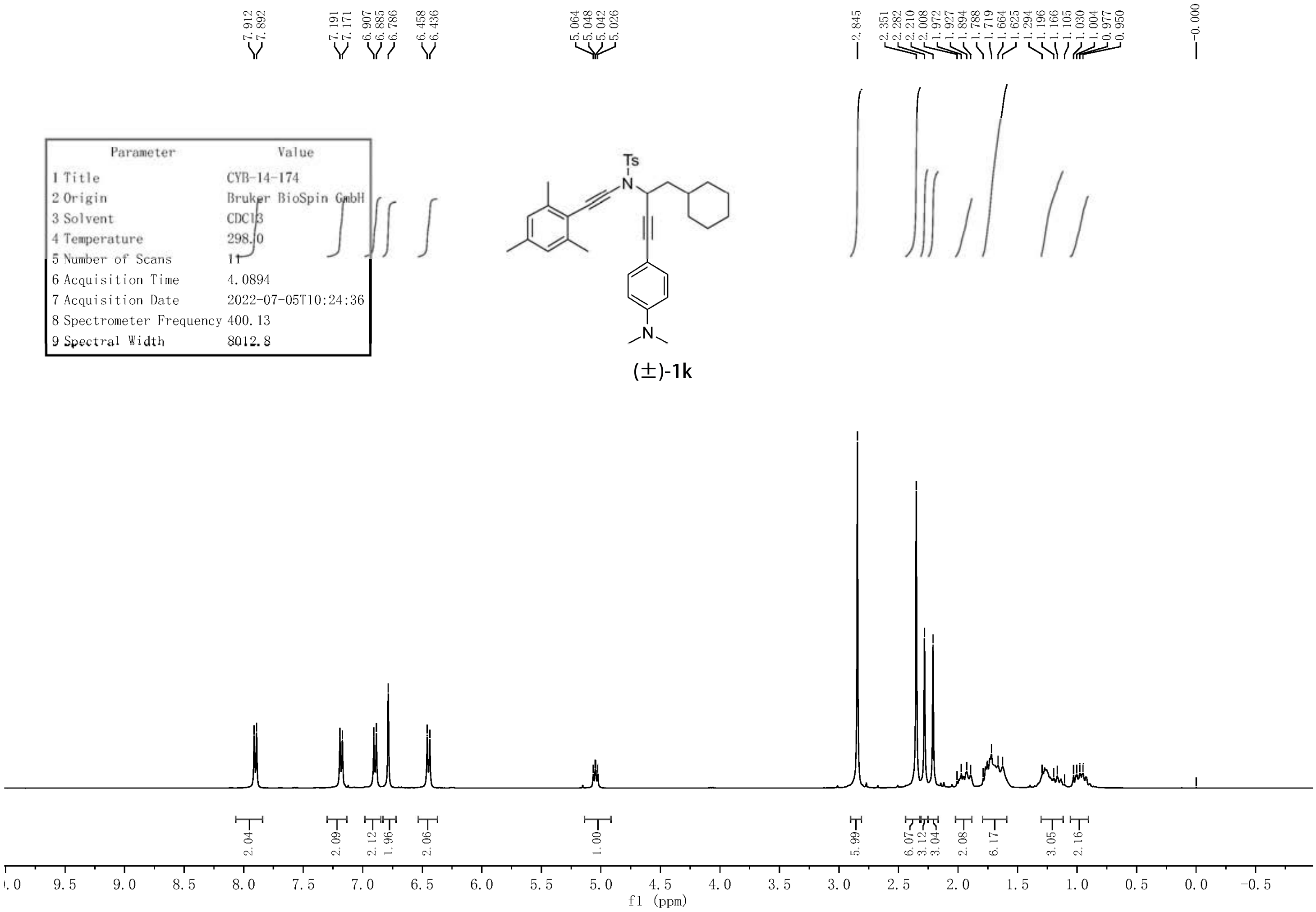

Parameter	Value
1 Title	CYB-14-174
2 Origin	Bruker BioSpin GmbH
3 Solvent	CDCl3
4 Temperature	298.0
5 Number of Scans	11
6 Acquisition Time	4.0894
7 Acquisition Date	2022-07-05T10:24:36
8 Spectrometer Frequency	400.13
9 Spectral Width	8012.8

(±)-1k

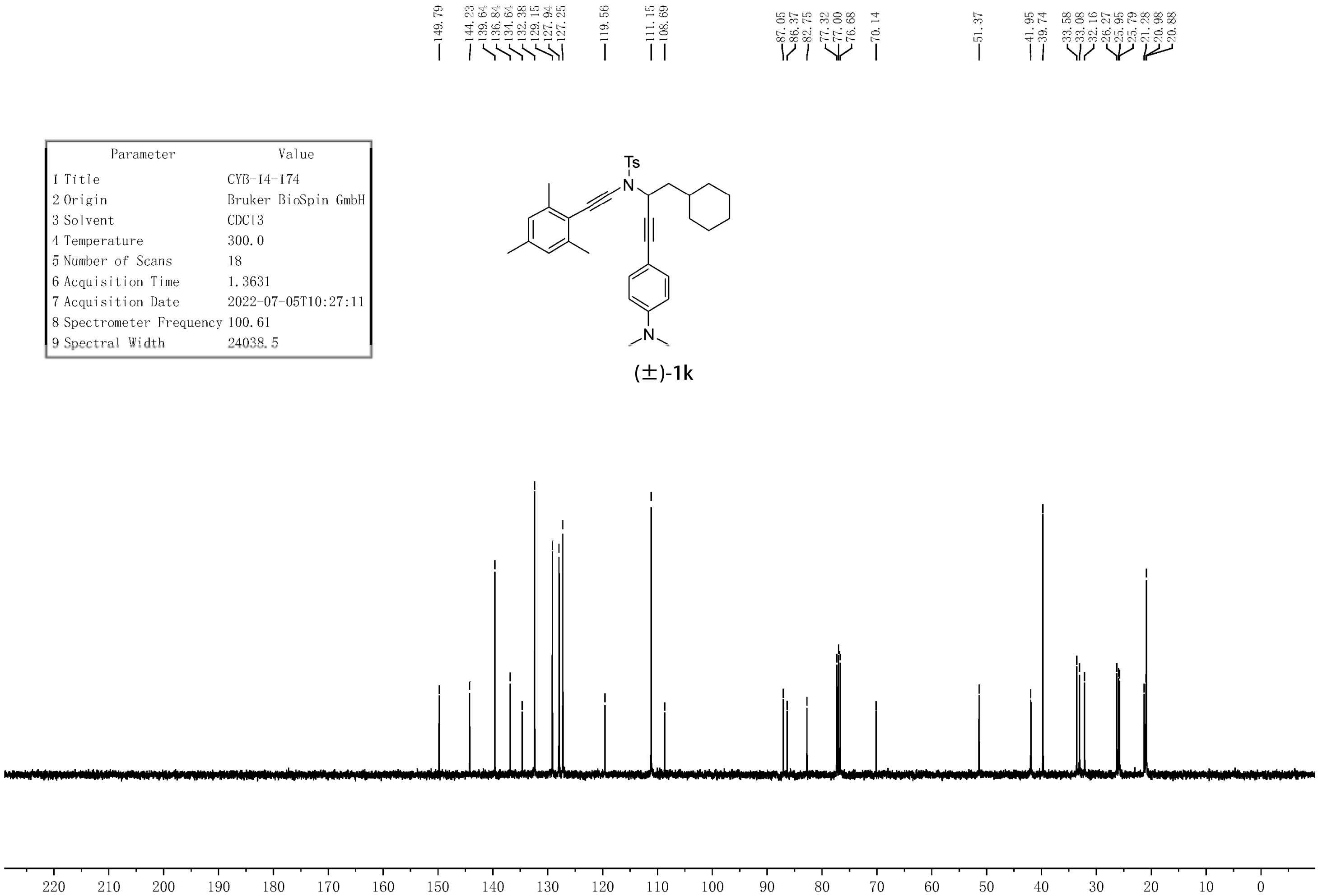

Parameter	Value
1 Title	CYB-14-174
2 Origin	Bruker BioSpin GmbH
3 Solvent	CDCl3
4 Temperature	300.0
5 Number of Scans	18
6 Acquisition Time	1.3631
7 Acquisition Date	2022-07-05T10:27:11
8 Spectrometer Frequency	100.61
9 Spectral Width	24038.5

(±)-1k

Supplementary Figure 119. ^{1}H and ^{13}C NMR spectra for (±)-1k

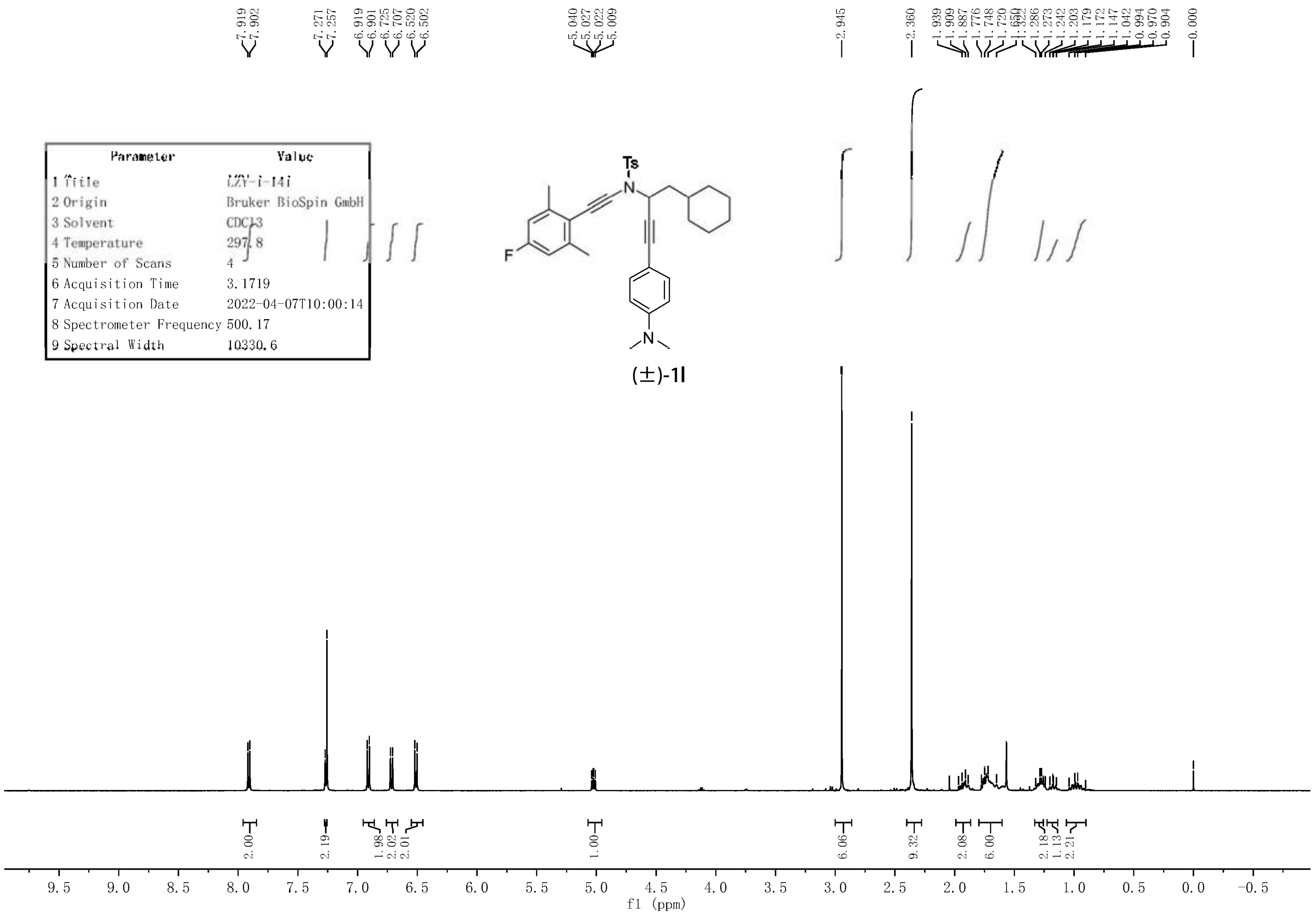

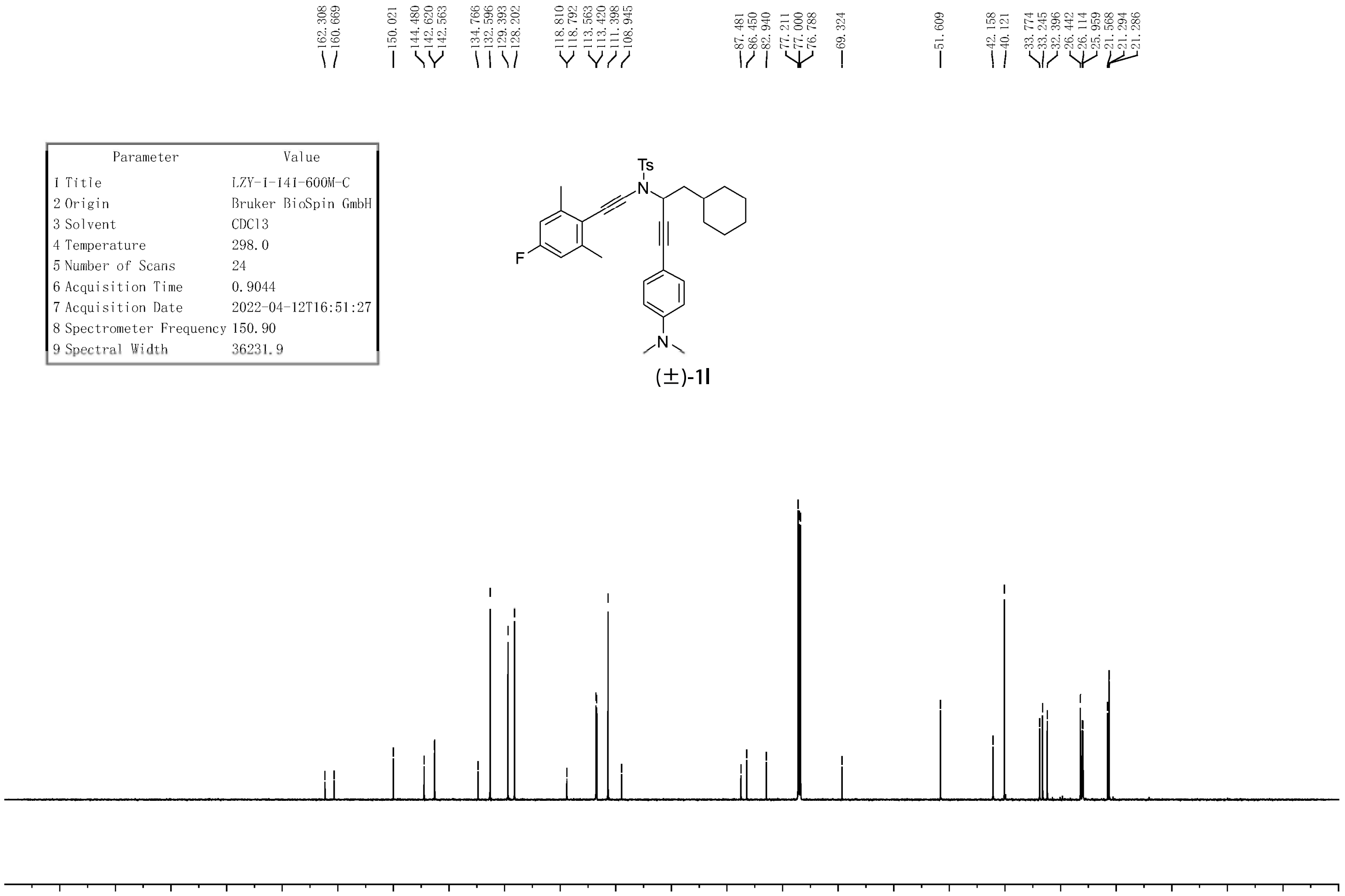

Supplementary Figure 120. ^{1}H and ^{13}C NMR spectra for (±)-1l

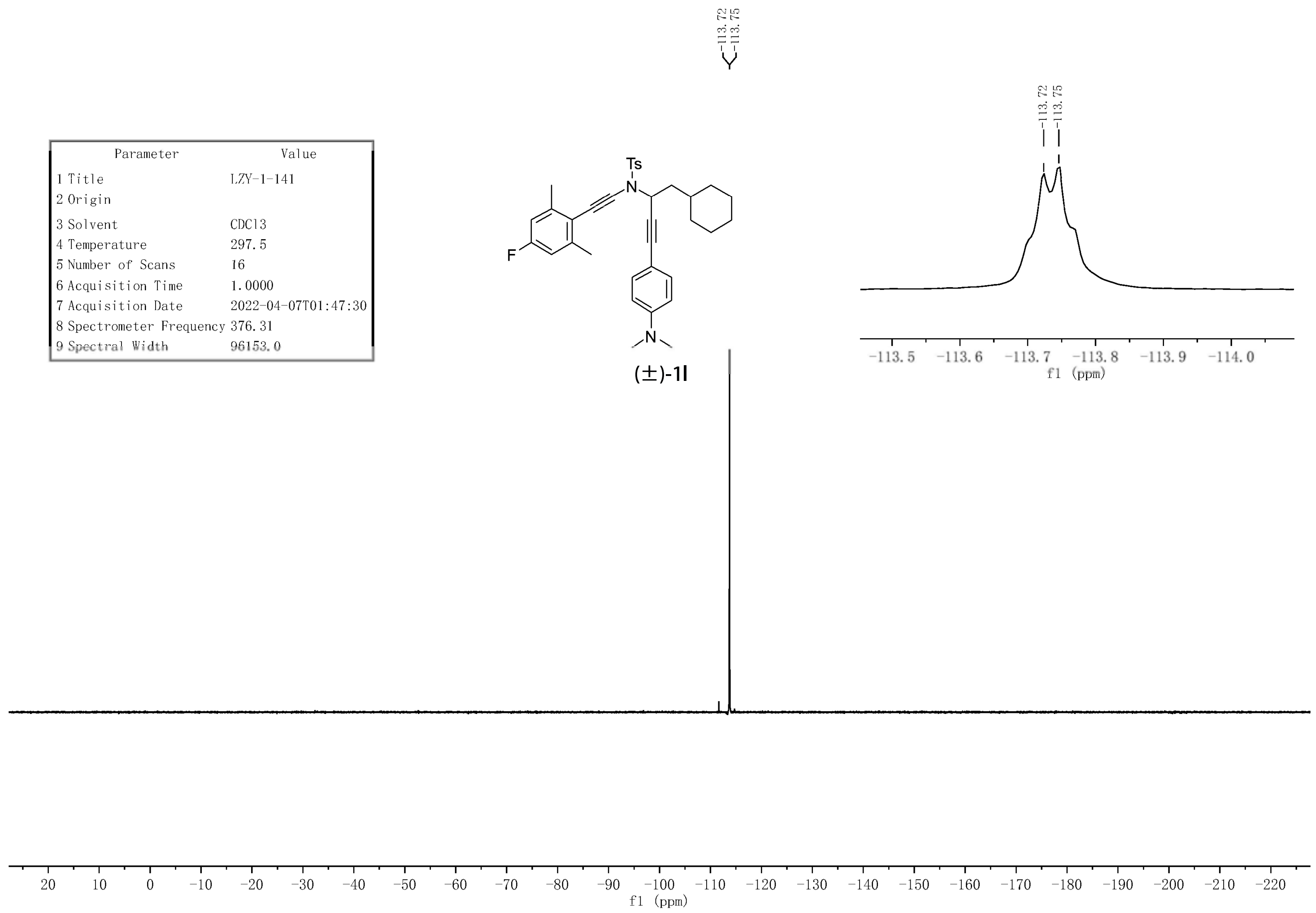

Supplementary Figure 121. ^{19}F NMR spectra for (±)-1l

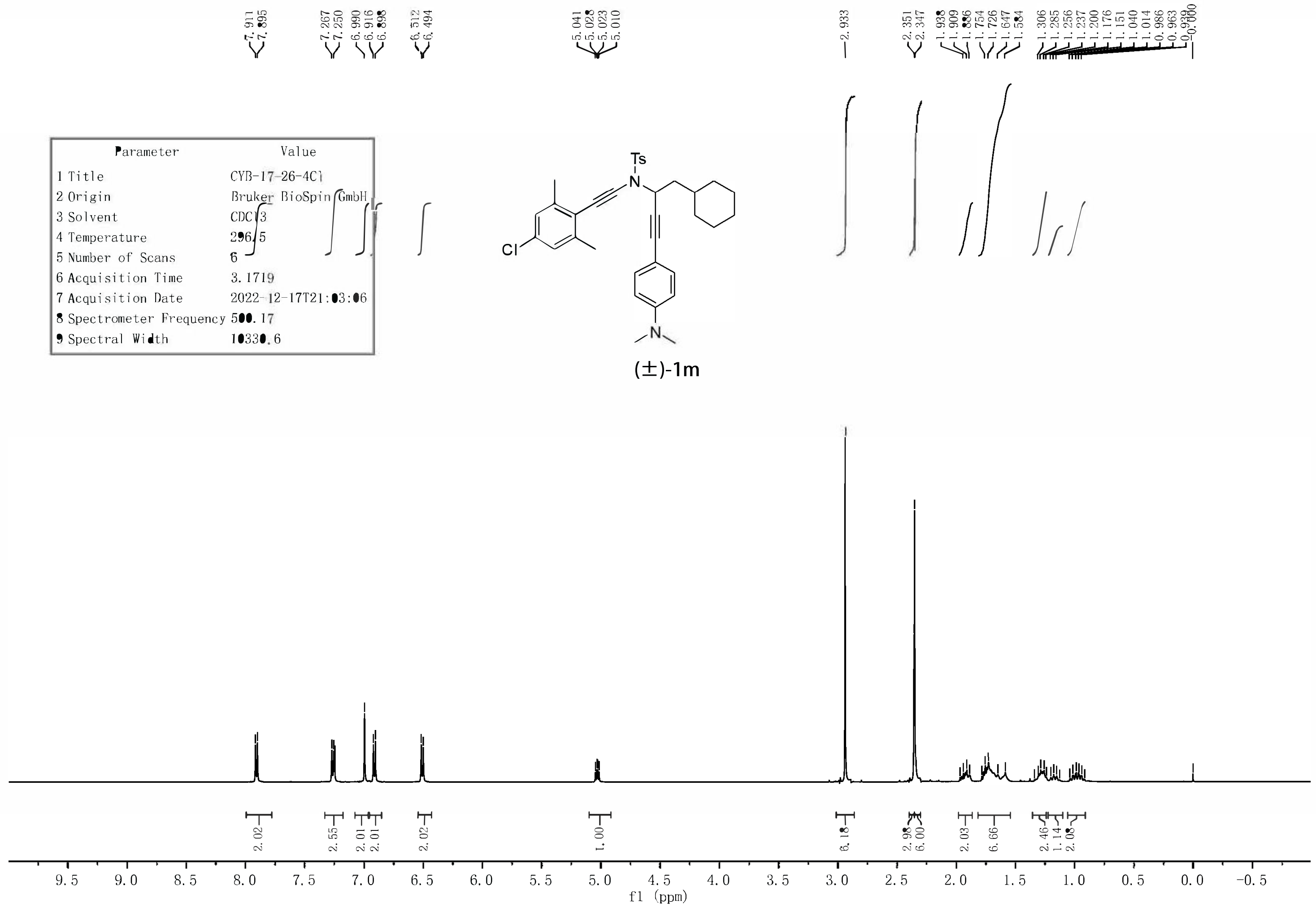

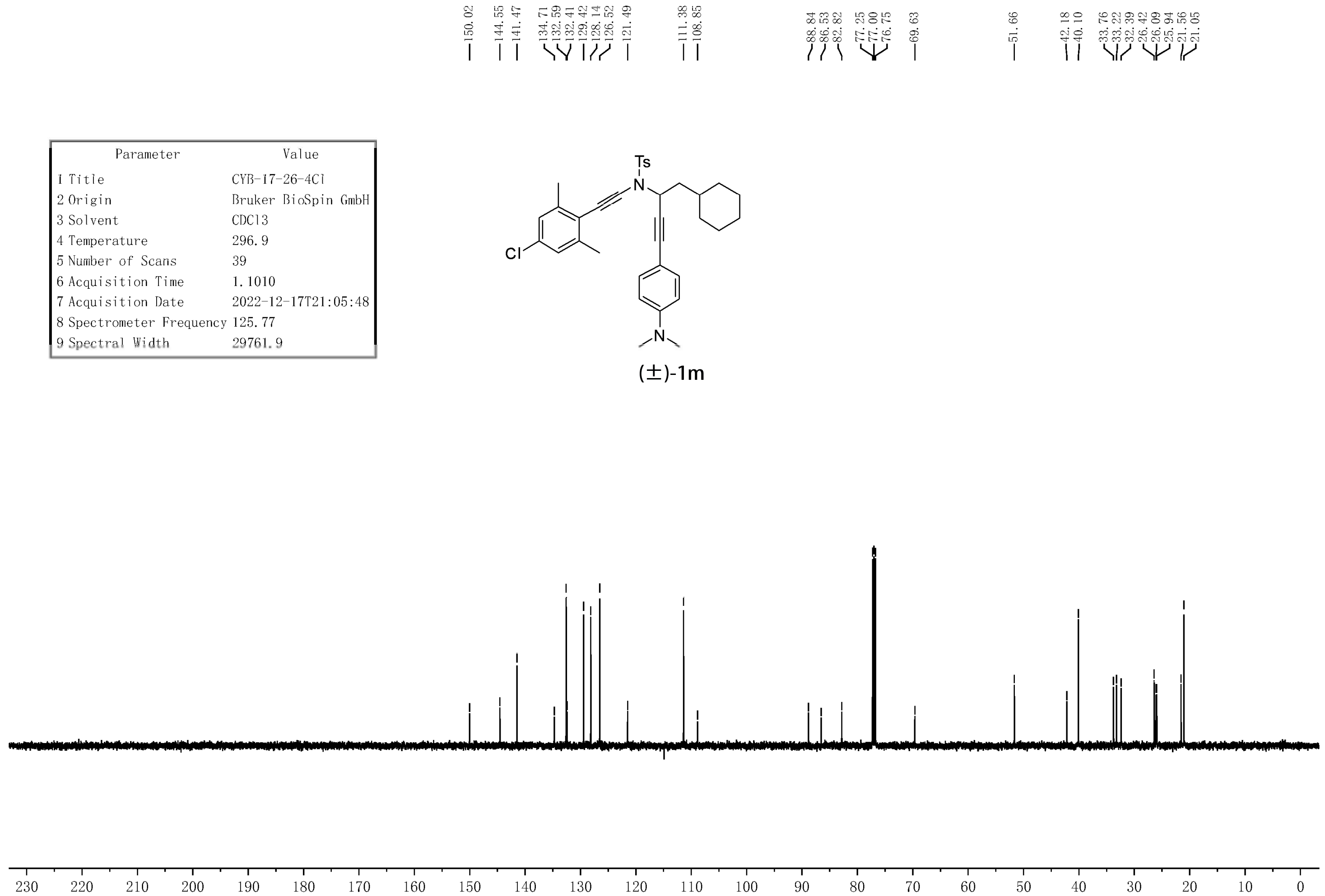

Supplementary Figure 122. ^{1}H and ^{13}C NMR spectra for (±)-1m

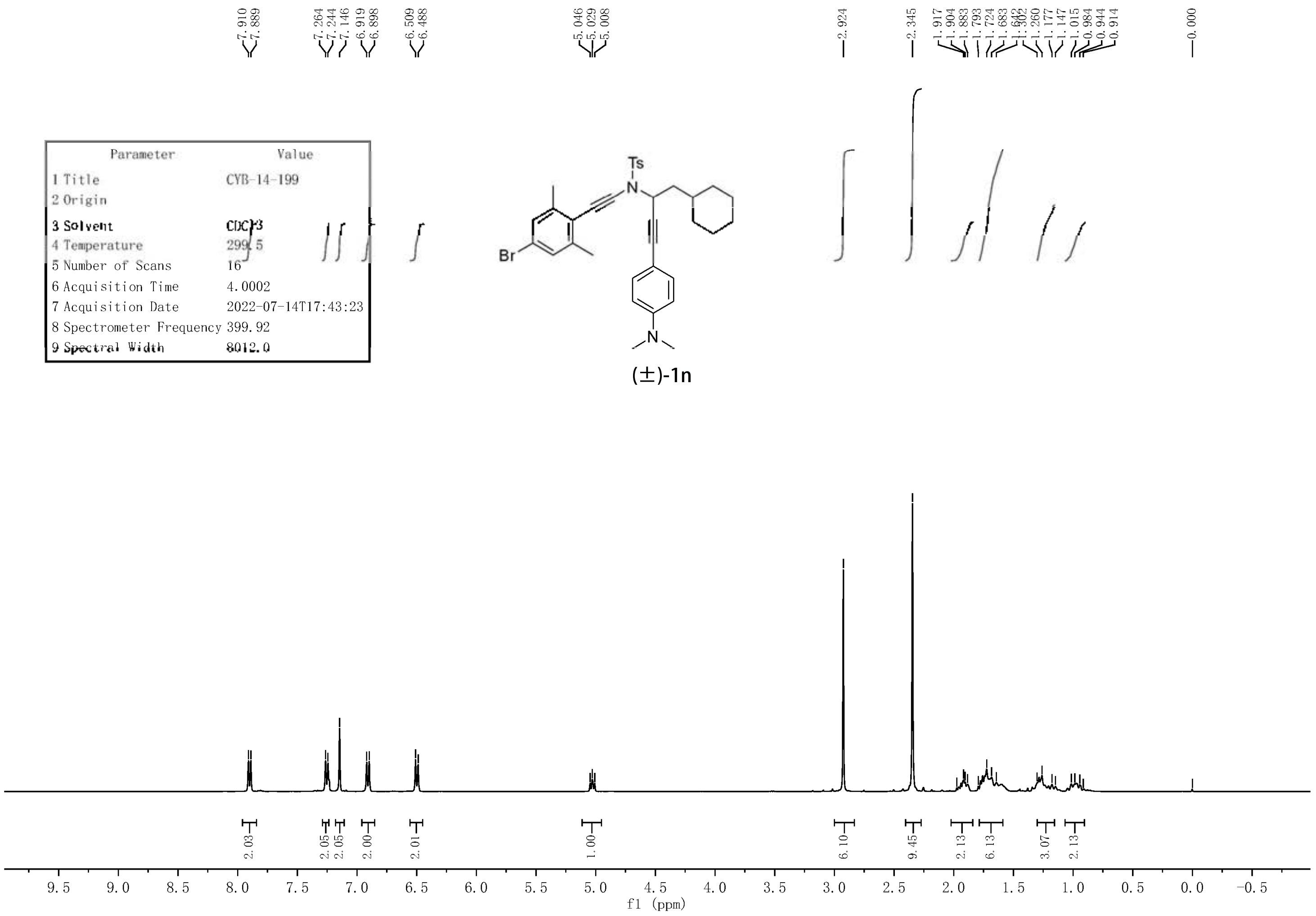

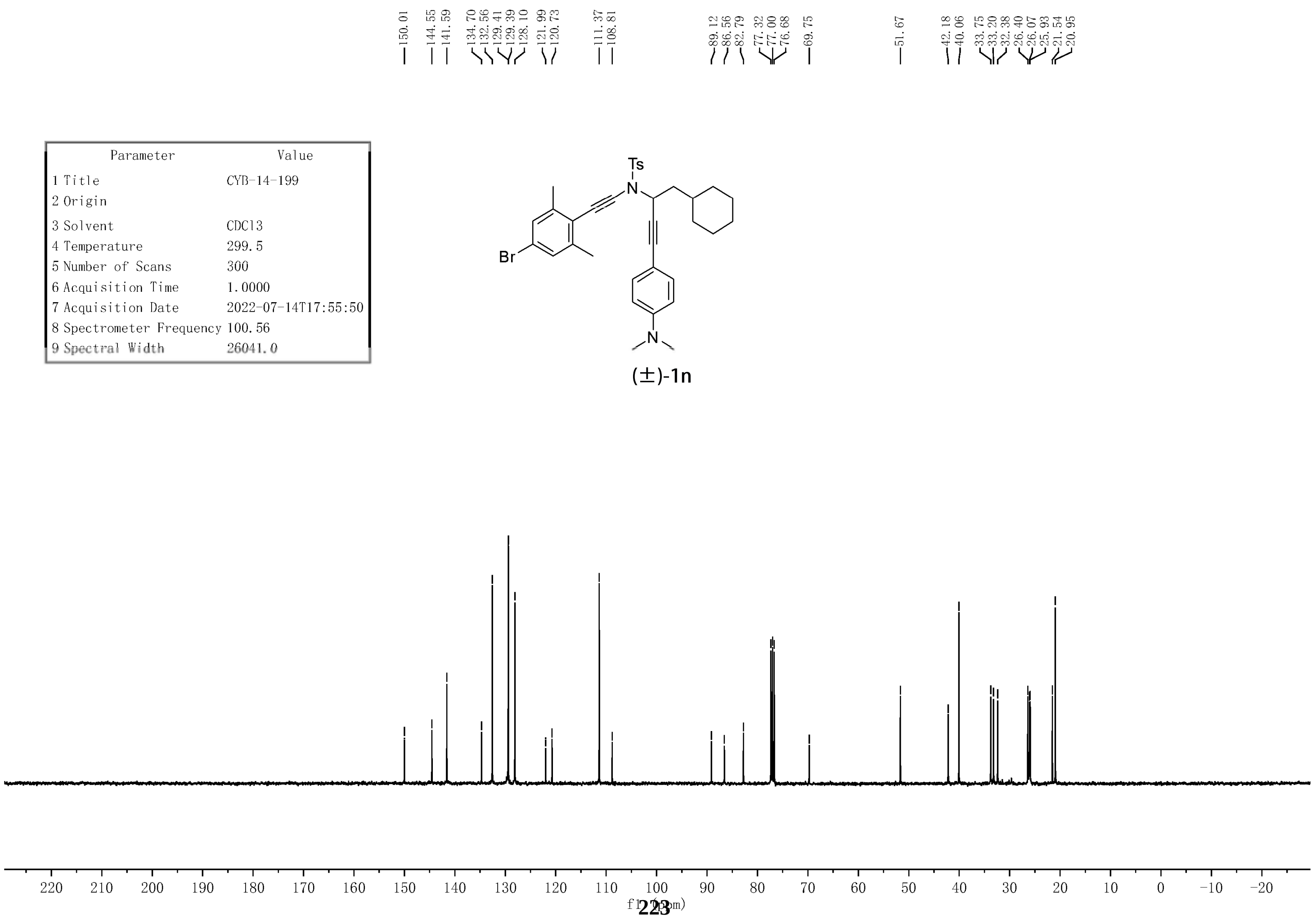

Supplementary Figure 123. ^{1}H and ^{13}C NMR spectra for (±)-1n

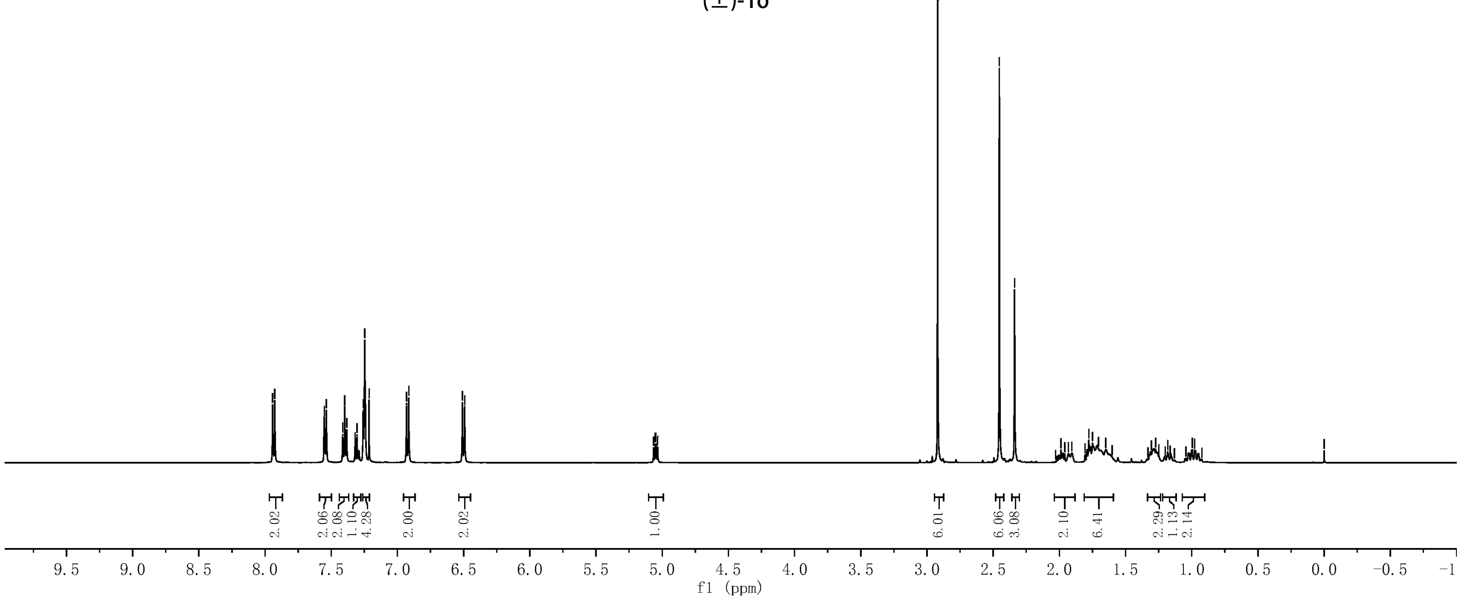

Parameter	Value
1 Title	CYB-16-229
2 Origin	Bruker BioSpin GmbH
3 Solvent	CDCl3
4 Temperature	297.8
5 Number of Scans	4
6 Acquisition Time	3.1719
7 Acquisition Date	2022-12-07T22:10:40
8 Spectrometer Frequency	500.17
9 Spectral Width	10330.6

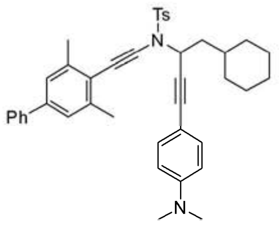

(±)-1o

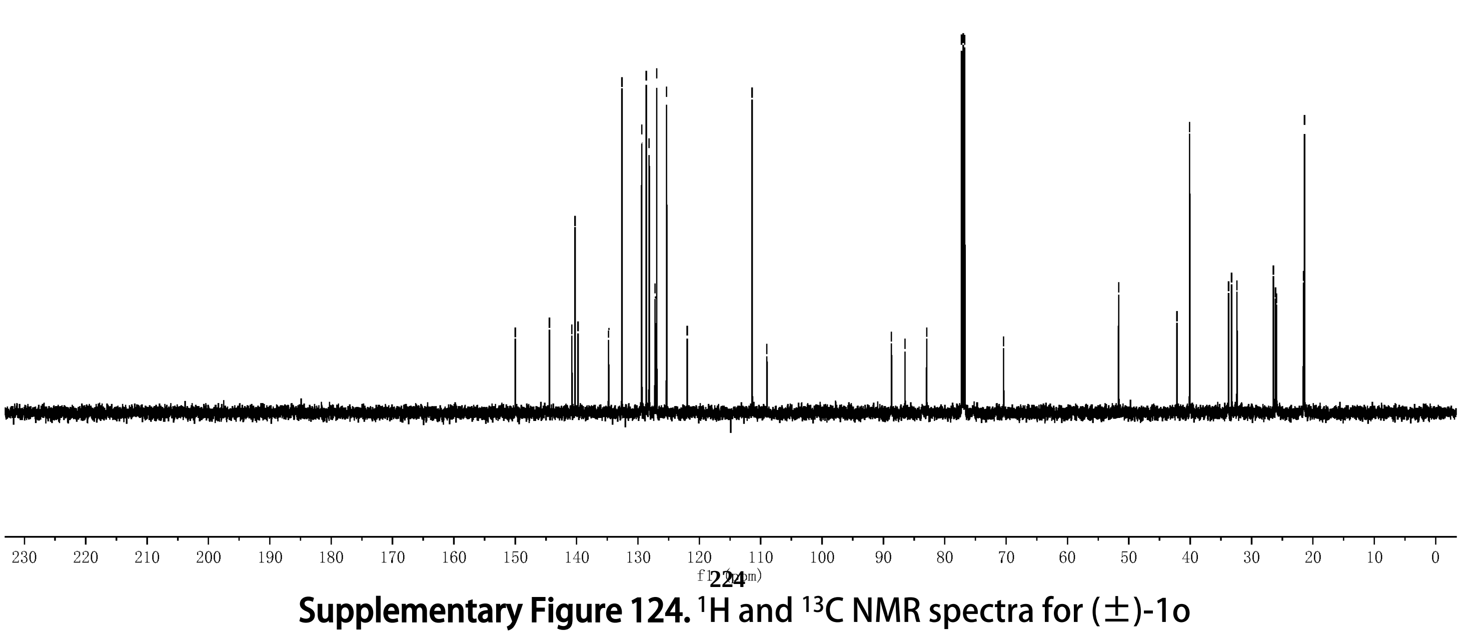

Parameter	Value
1 Title	CYB-16-229
2 Origin	Bruker BioSpin GmbH
3 Solvent	CDCl3
4 Temperature	298.1
5 Number of Scans	43
6 Acquisition Time	1.1010
7 Acquisition Date	2022-12-07T22:12:49
8 Spectrometer Frequency	125.77
9 Spectral Width	29761.9

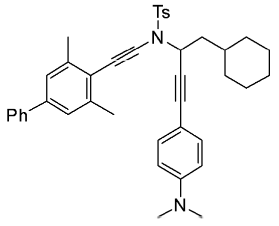

(±)-1o

Supplementary Figure 124. ^{1}H and ^{13}C NMR spectra for (±)-1o

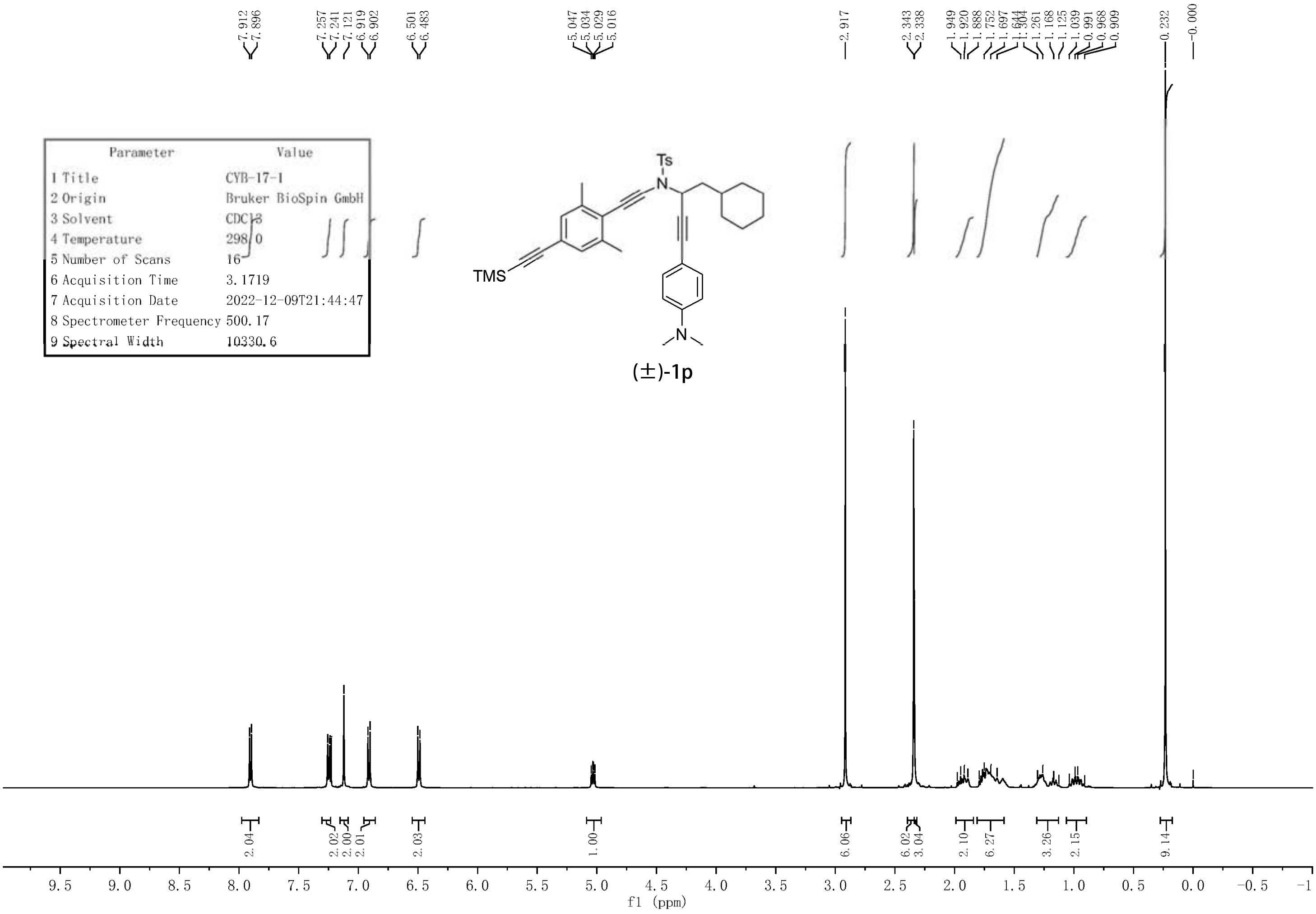

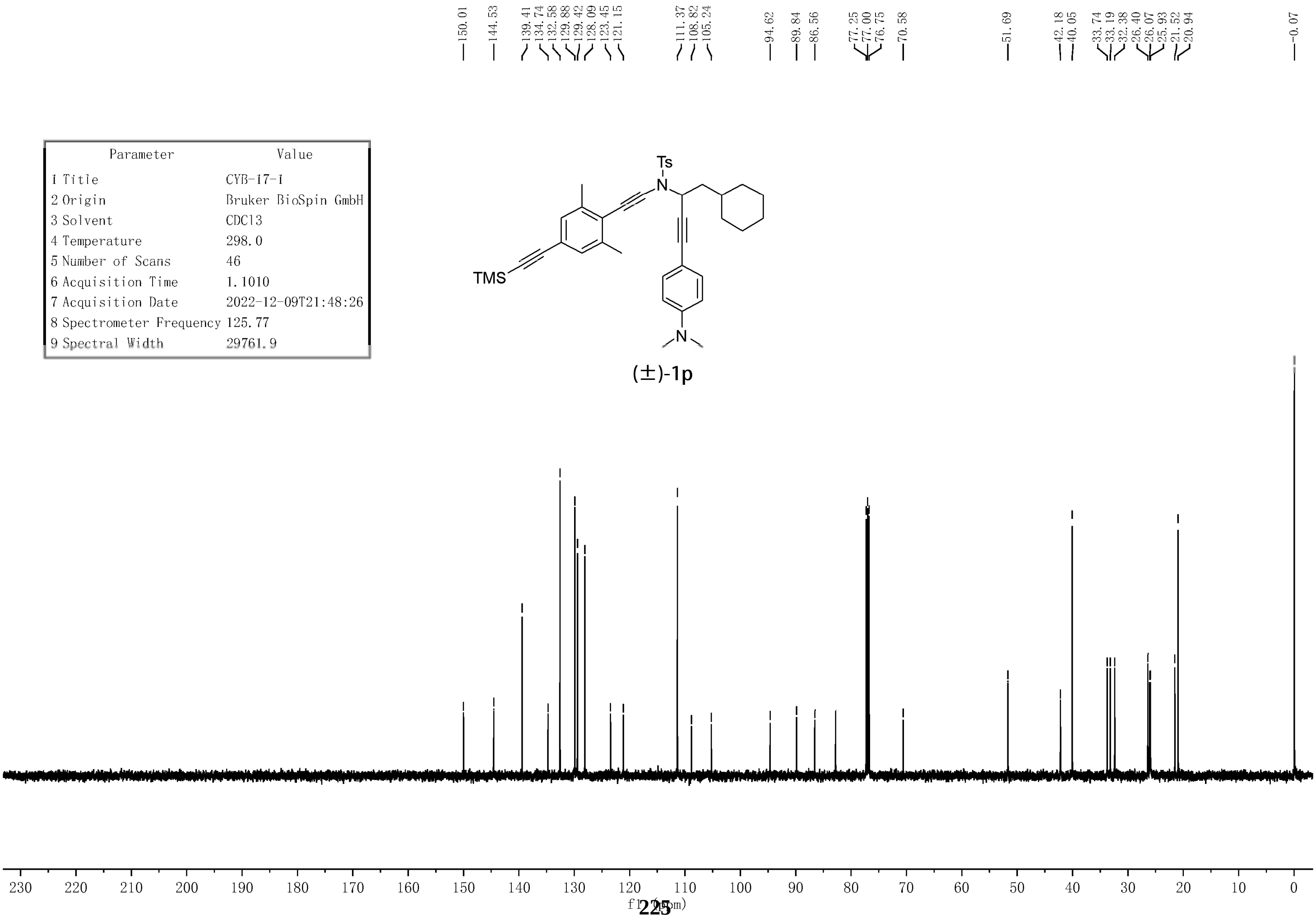

Supplementary Figure 125. ^{1}H and ^{13}C NMR spectra for (±)-1p

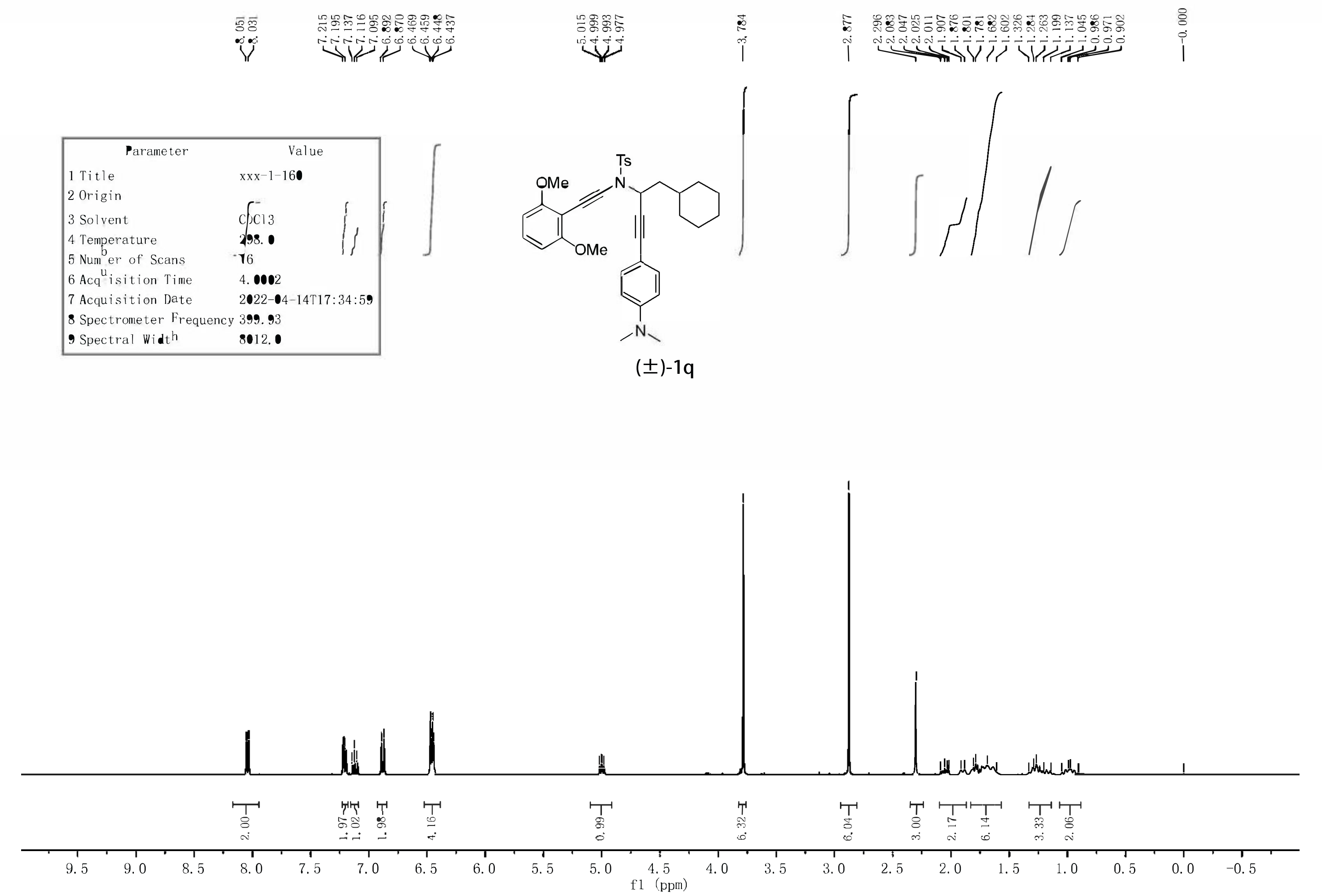

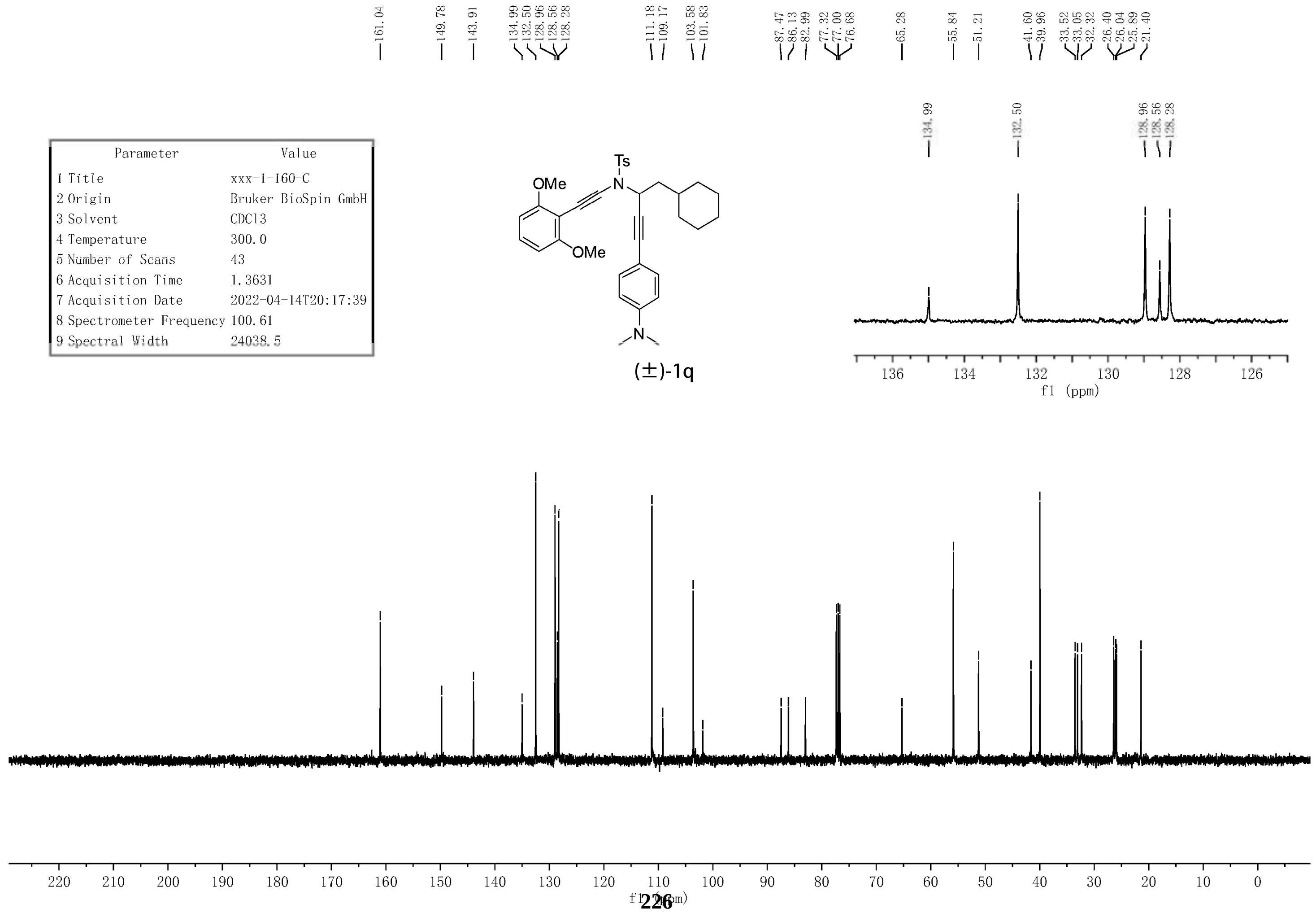

Supplementary Figure 126. ^{1}H and ^{13}C NMR spectra for (±)-1q

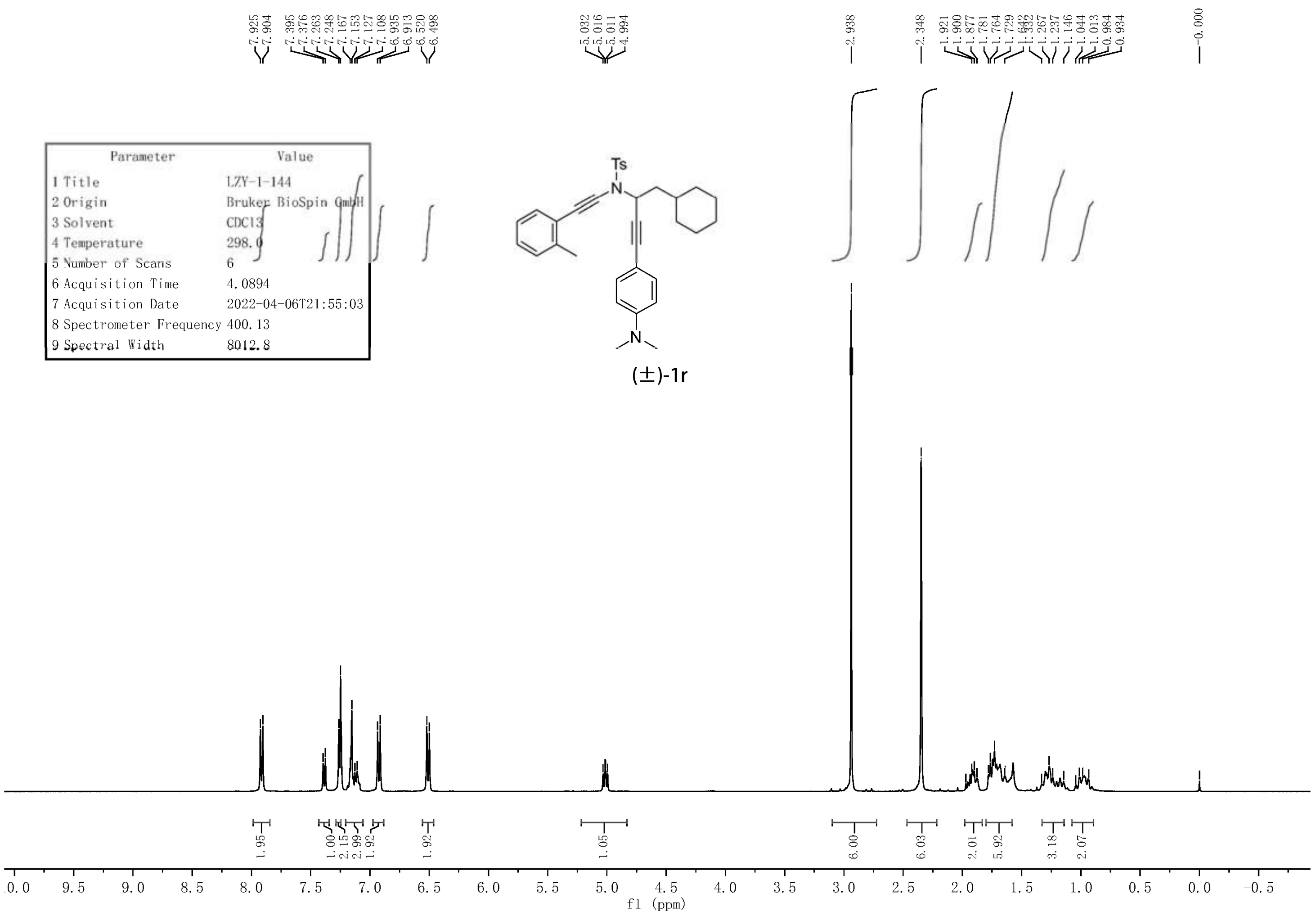

Parameter	Value
1 Title	LZY-1-144
2 Origin	Bruker BioSpin GmbH
3 Solvent	CDCl3
4 Temperature	298.0
5 Number of Scans	6
6 Acquisition Time	4.0894
7 Acquisition Date	2022-04-06T21:55:03
8 Spectrometer Frequency	400.13
9 Spectral Width	8012.8

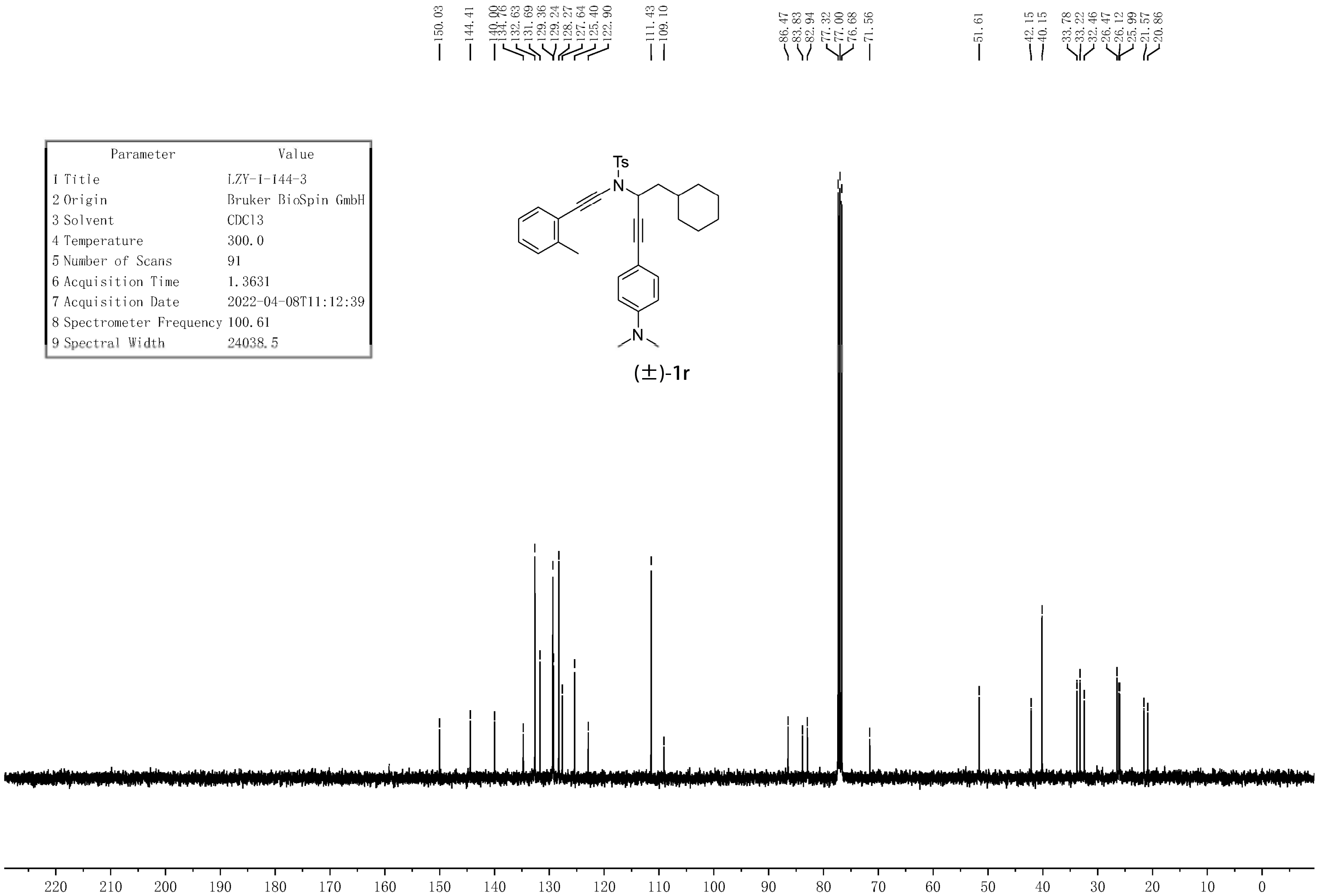

Parameter	Value
1 Title	LZY-1-144-3
2 Origin	Bruker BioSpin GmbH
3 Solvent	CDCl3
4 Temperature	300.0
5 Number of Scans	91
6 Acquisition Time	1.3631
7 Acquisition Date	2022-04-08T11:12:39
8 Spectrometer Frequency	100.61
9 Spectral Width	24038.5

Supplementary Figure 127. ^{1}H and ^{13}C NMR spectra for (±)-1r

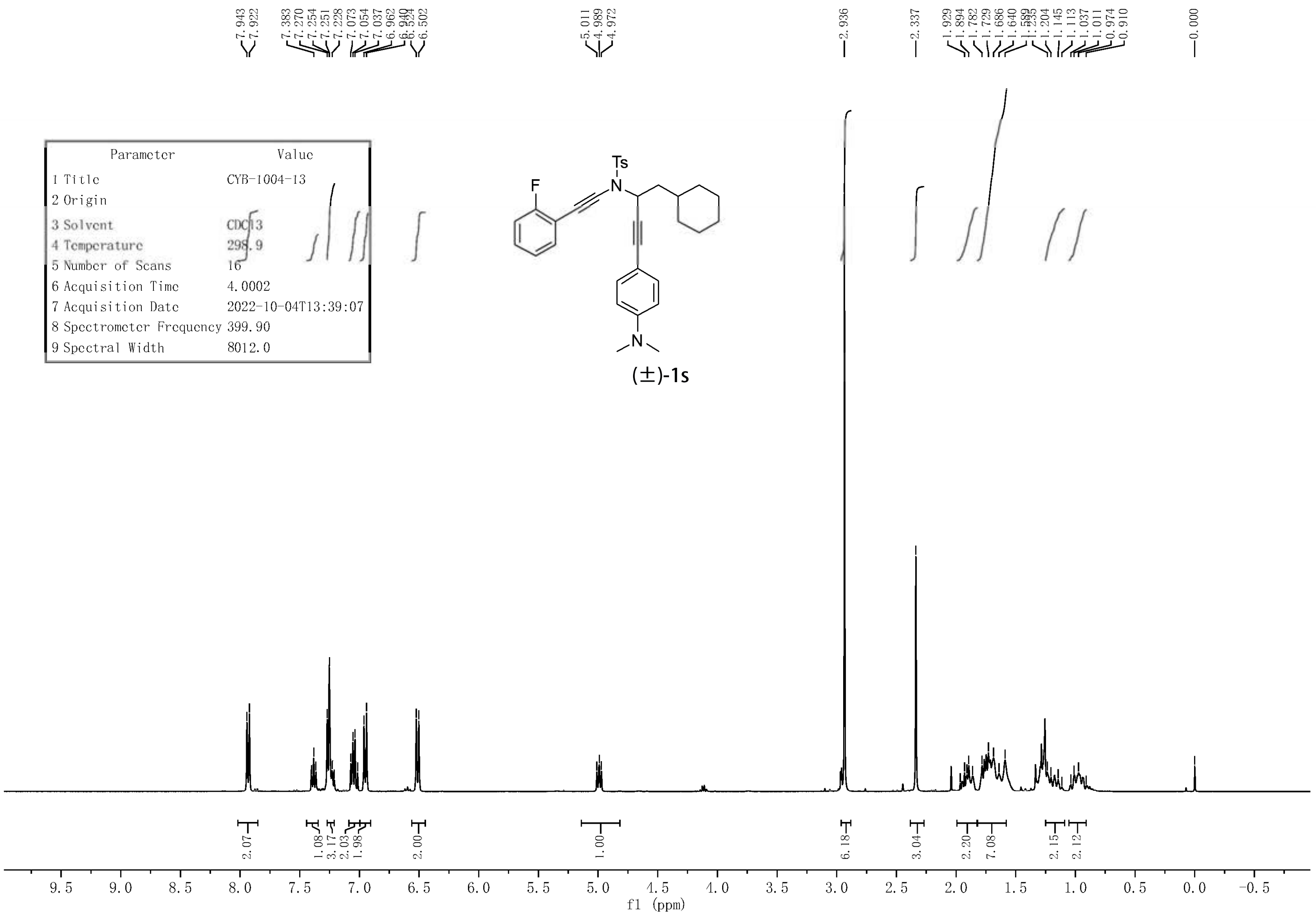

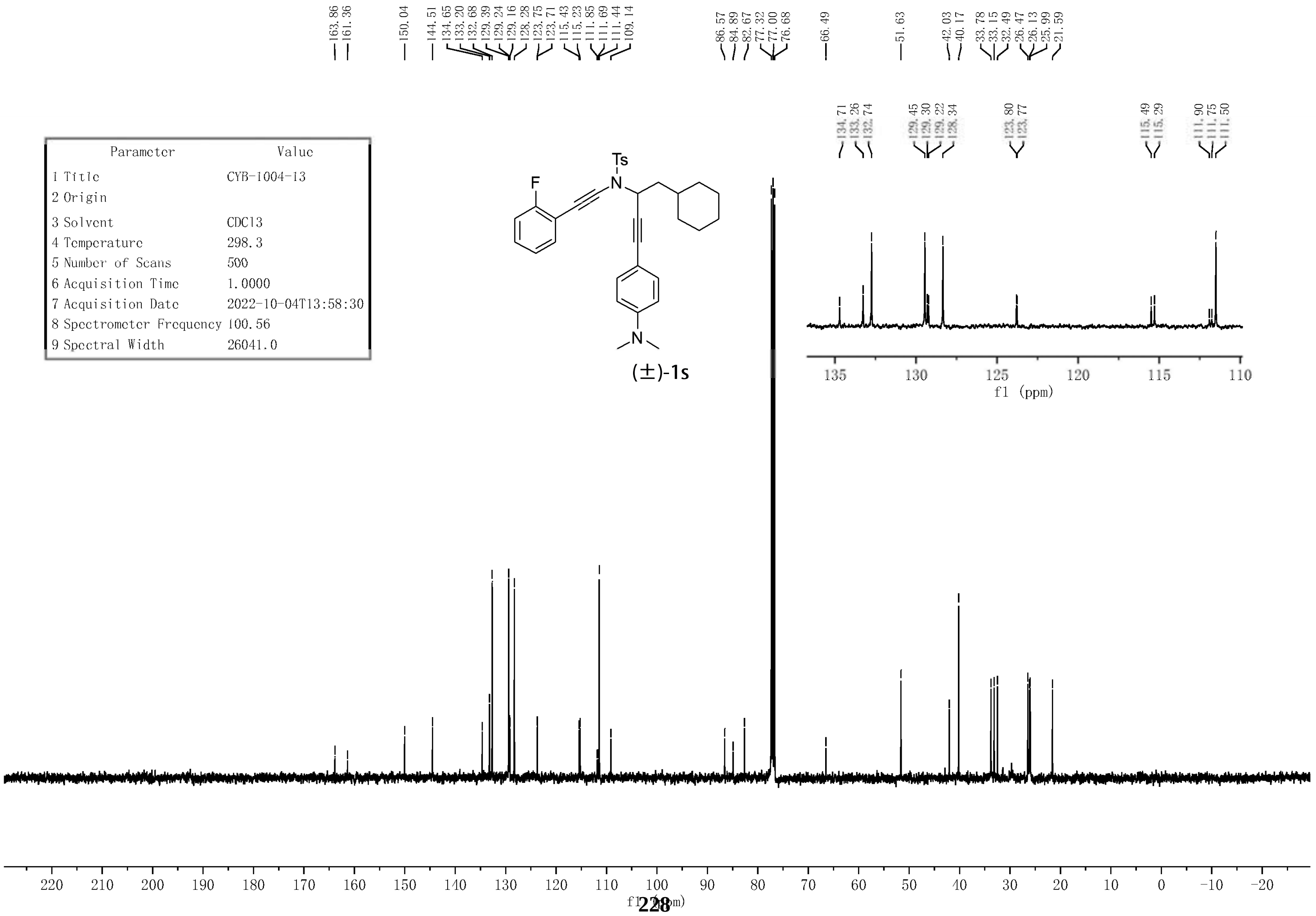

Supplementary Figure 128. ^{1}H and ^{13}C NMR spectra for (±)-1s

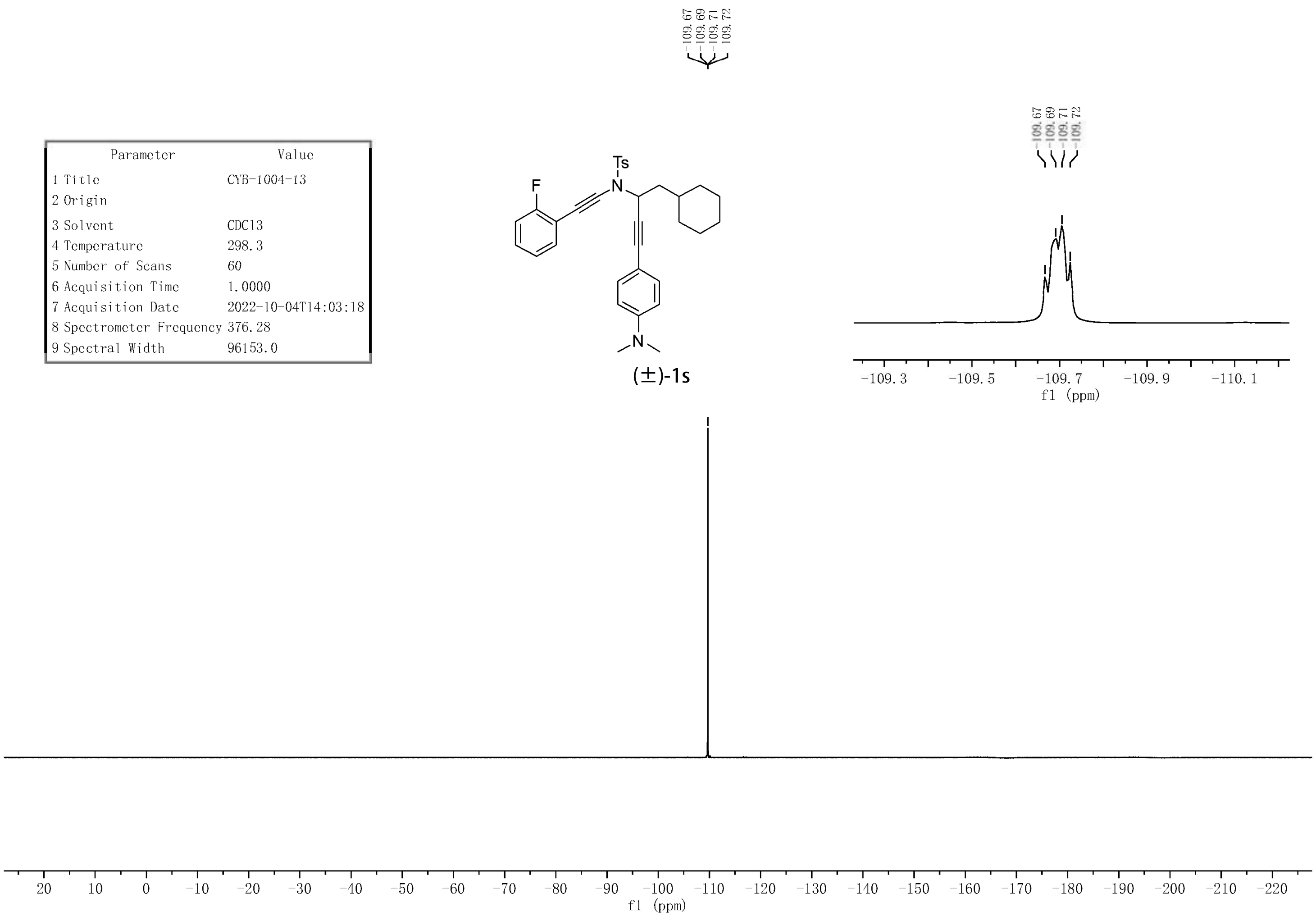

Supplementary Figure 129. ^{19}F NMR spectra for (±)-1s

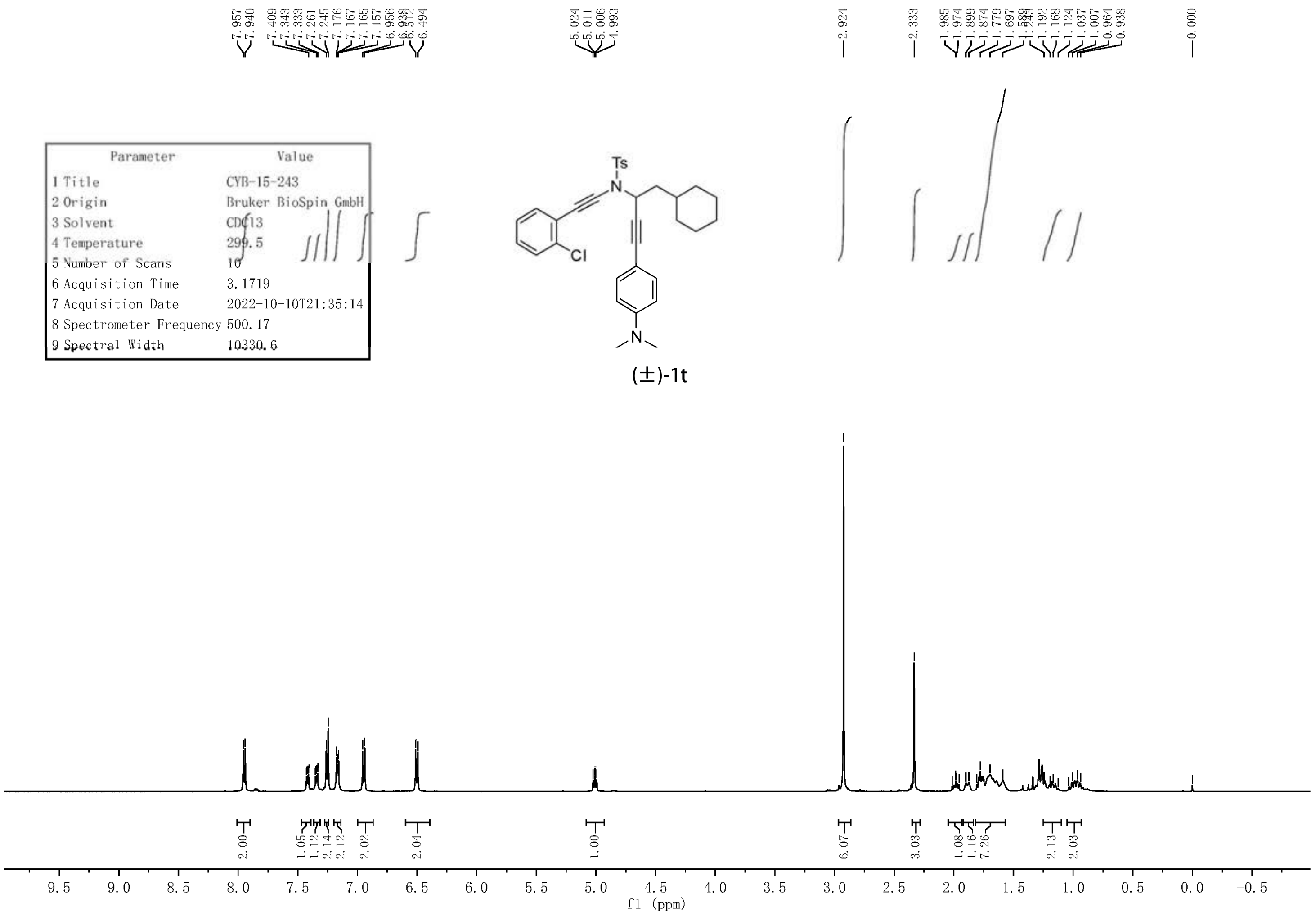

Parameter	Value
1 Title	CYB-15-243
2 Origin	Bruker BioSpin GmbH
3 Solvent	CDCl3
4 Temperature	299.5
5 Number of Scans	10
6 Acquisition Time	3.1719
7 Acquisition Date	2022-10-10T21:35:14
8 Spectrometer Frequency	500.17
9 Spectral Width	10330.6

(±)-1t

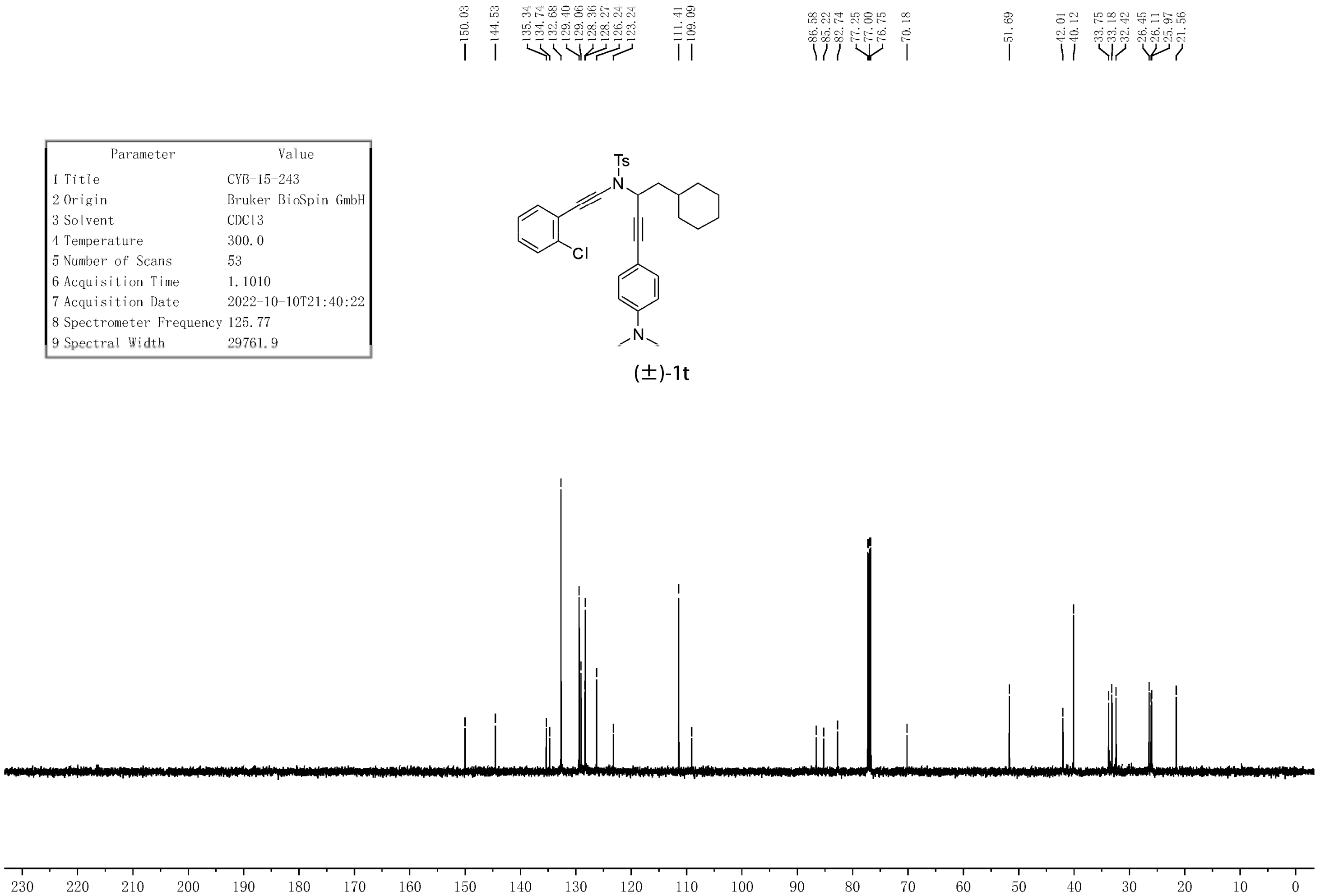

Parameter	Value
1 Title	CYB-15-243
2 Origin	Bruker BioSpin GmbH
3 Solvent	CDCl3
4 Temperature	300.0
5 Number of Scans	53
6 Acquisition Time	1.1010
7 Acquisition Date	2022-10-10T21:40:22
8 Spectrometer Frequency	125.77
9 Spectral Width	29761.9

(±)-1t

Supplementary Figure 130. ^{1}H and ^{13}C NMR spectra for (±)-1t

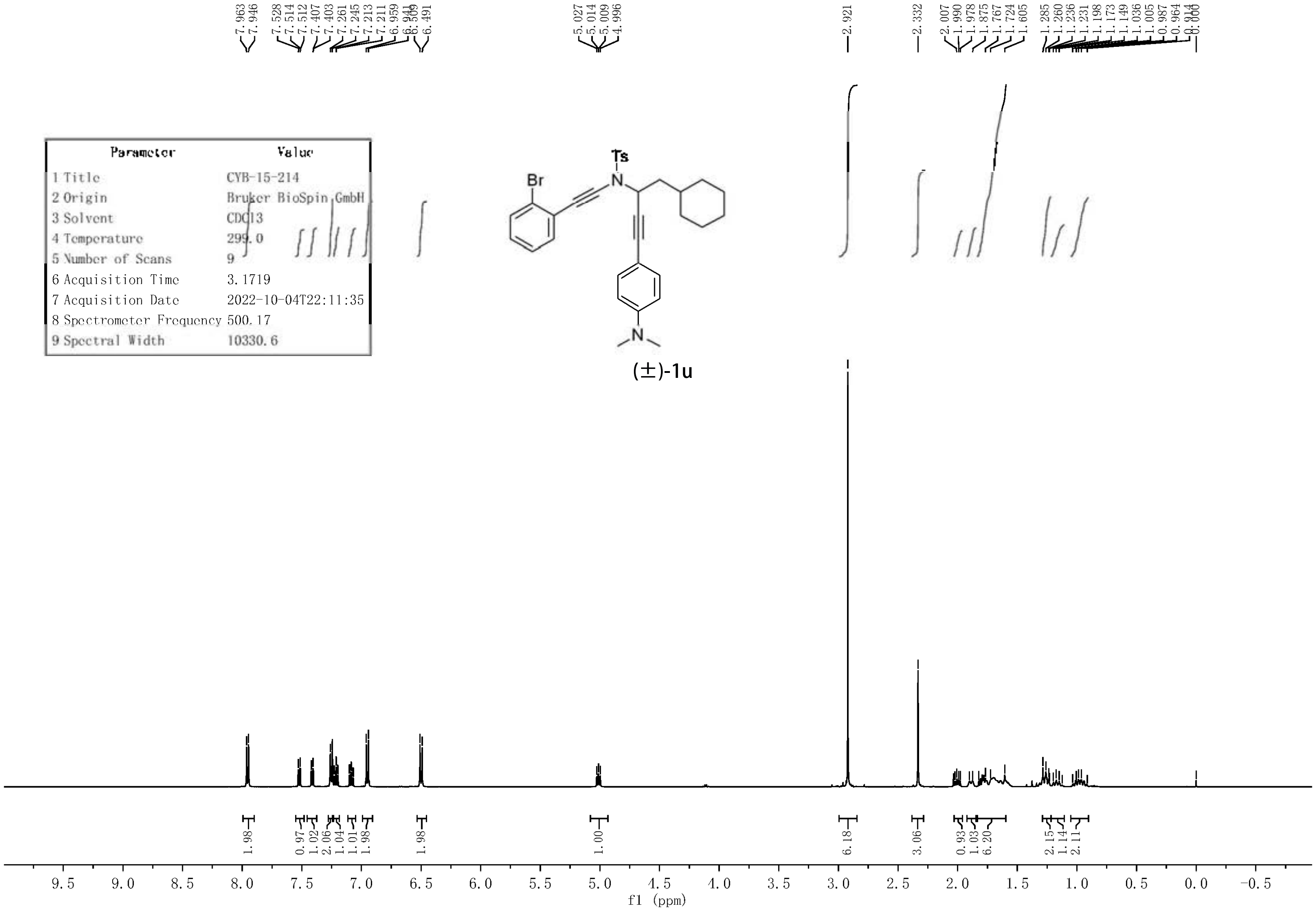

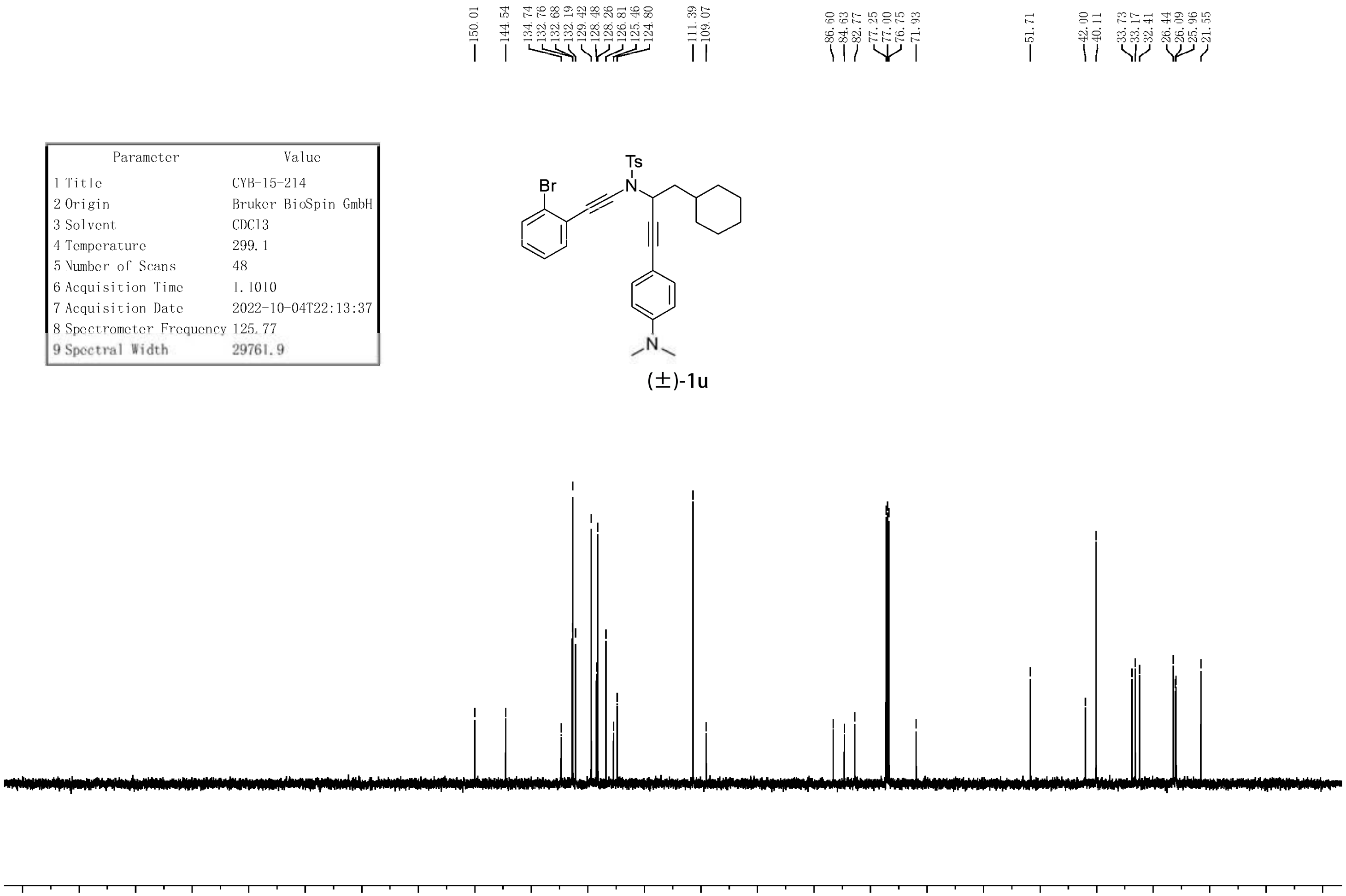

Supplementary Figure 131. ^{1}H and ^{13}C NMR spectra for (±)-1u

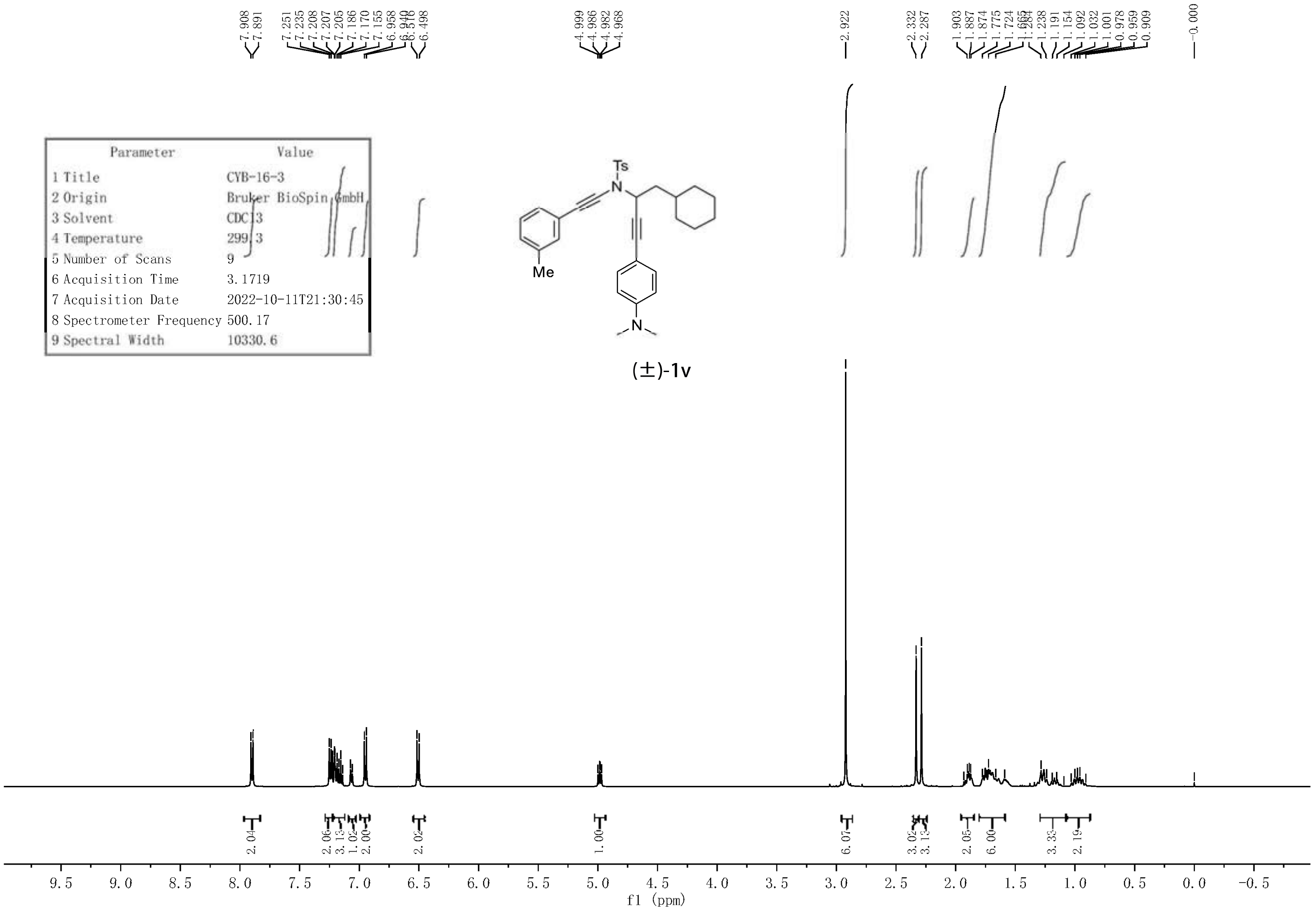

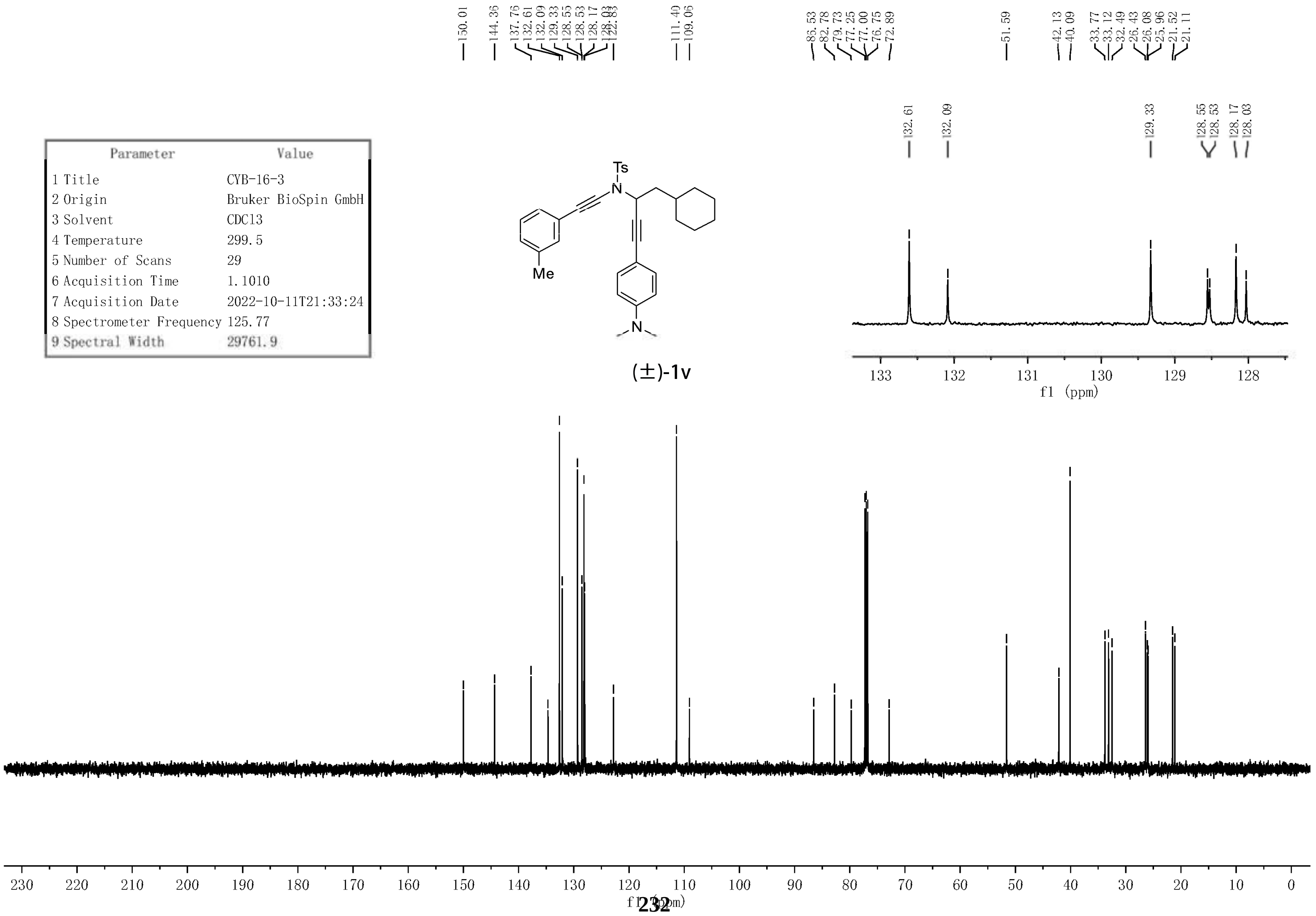

Supplementary Figure 132. ^{1}H and ^{13}C NMR spectra for (±)-1v

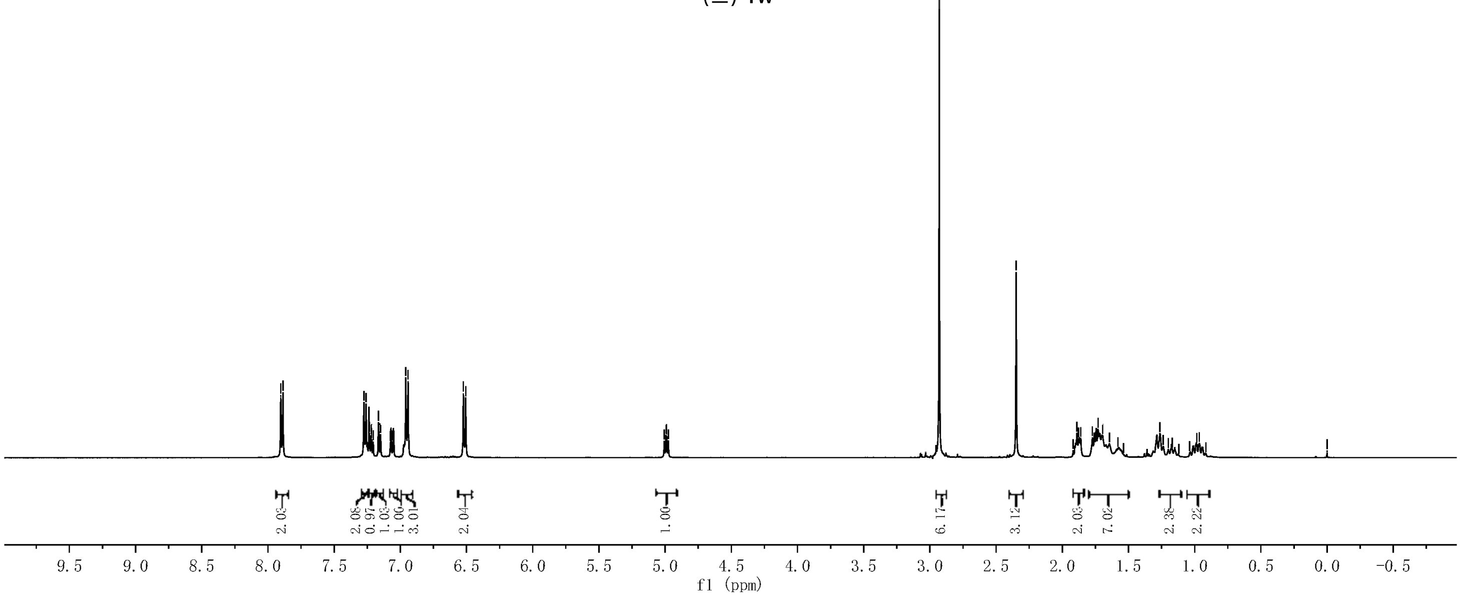

Parameter	Value
1 Title	CYB-17-208
2 Origin	Bruker BioSpin GmbH
3 Solvent	CDCl3
4 Temperature	296.8
5 Number of Scans	3
6 Acquisition Time	3.1719
7 Acquisition Date	2023-03-09T22:09:48
8 Spectrometer Frequency	500.17
9 Spectral Width	10330.6

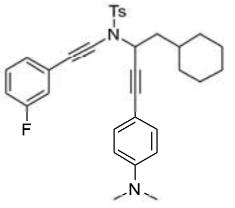

(±)-1w

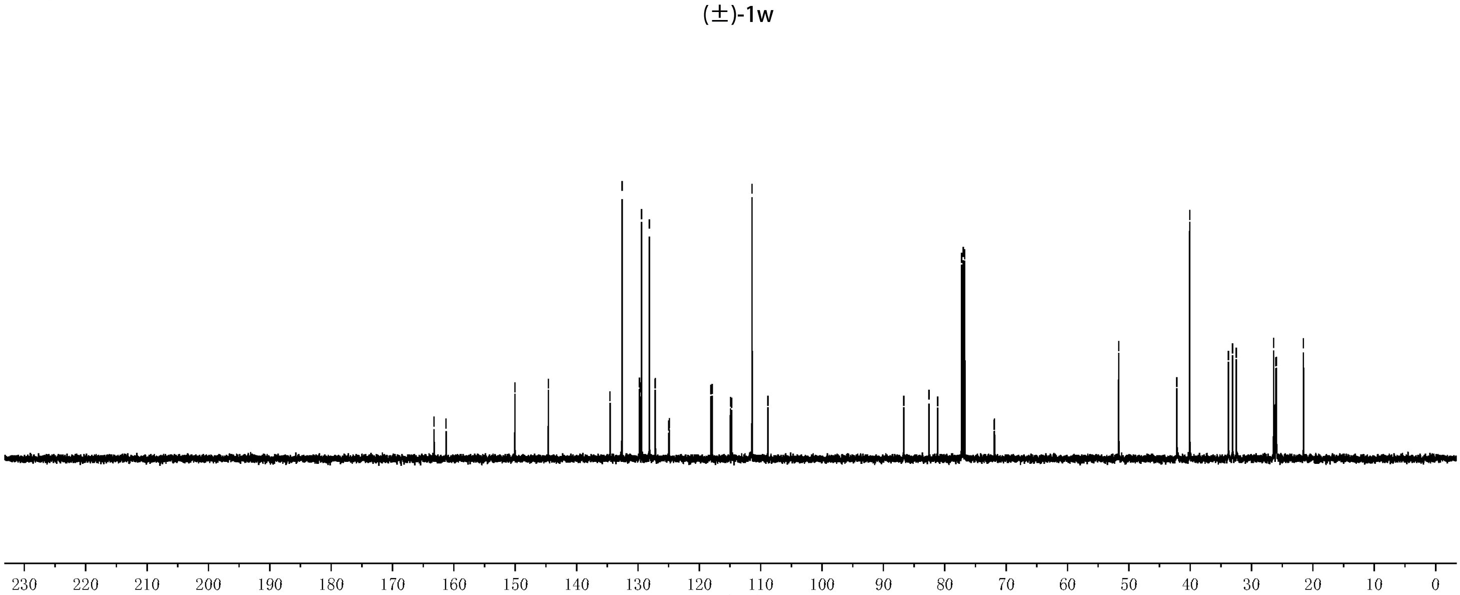

Parameter	Value
1 Title	CYB-17-208
2 Origin	Bruker BioSpin GmbH
3 Solvent	CDCl3
4 Temperature	297.2
5 Number of Scans	44
6 Acquisition Time	1.1010
7 Acquisition Date	2023-03-09T22:12:55
8 Spectrometer Frequency	125.77
9 Spectral Width	29761.9

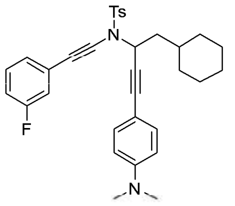

(±)-1w

Supplementary Figure 133. ^{1}H and ^{13}C NMR spectra for (±)-1w

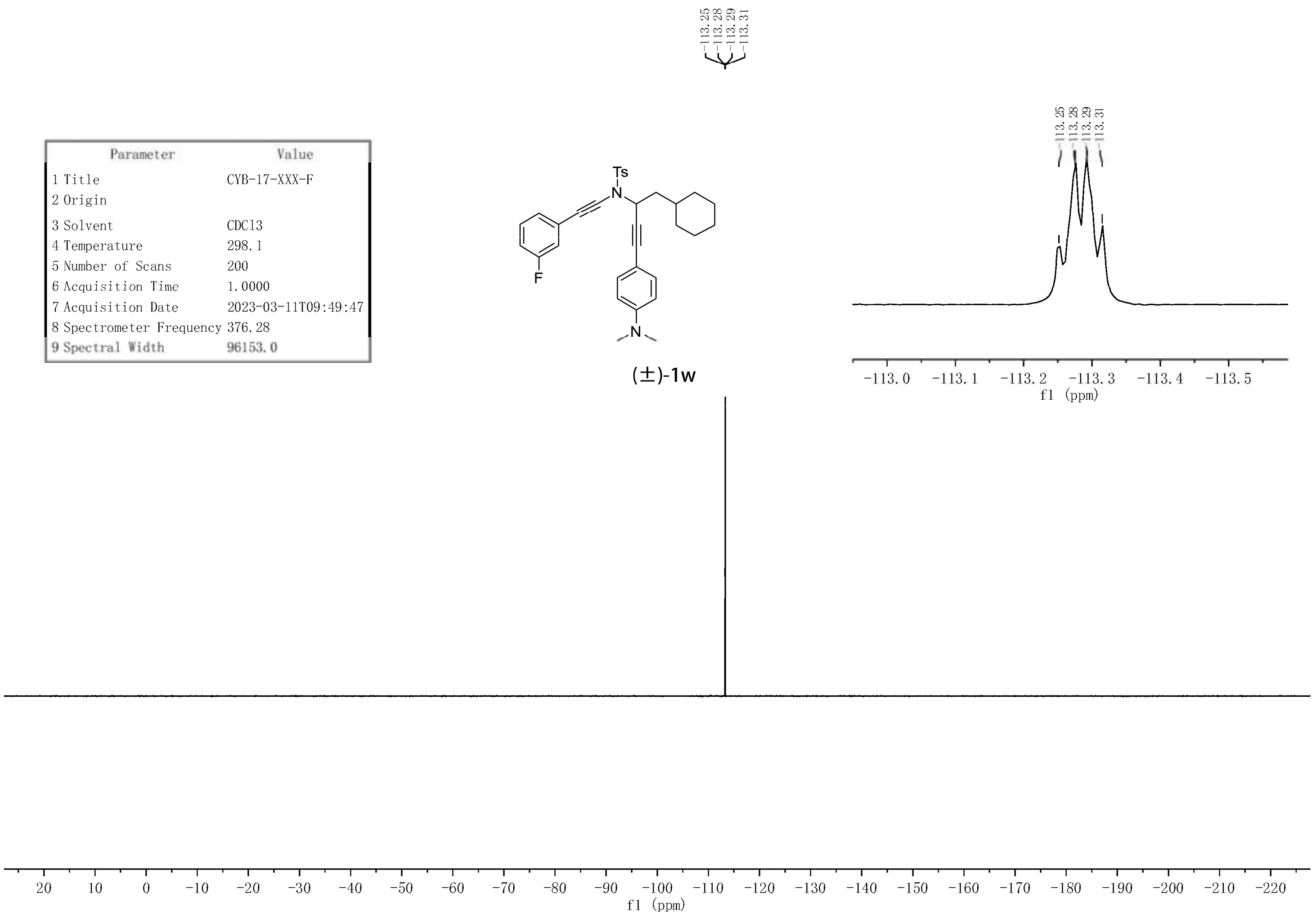

Supplementary Figure 134. ^{19}F NMR spectra for (±)-1w

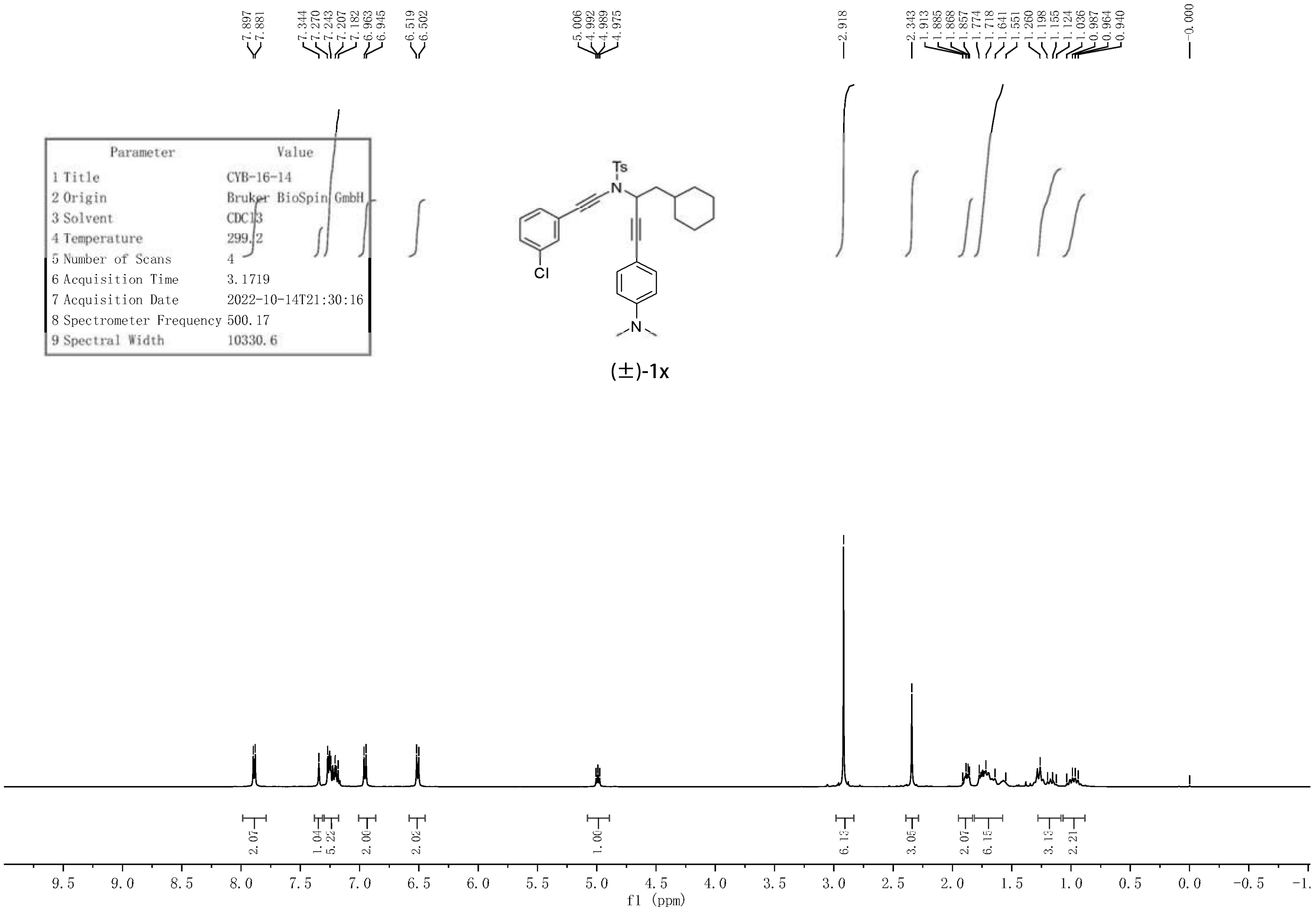

Parameter	Value
1 Title	CYB-16-14
2 Origin	Bruker BioSpin GmbH
3 Solvent	CDCl3
4 Temperature	299.2
5 Number of Scans	4
6 Acquisition Time	3.1719
7 Acquisition Date	2022-10-14T21:30:16
8 Spectrometer Frequency	500.17
9 Spectral Width	10330.6

(±)-1x

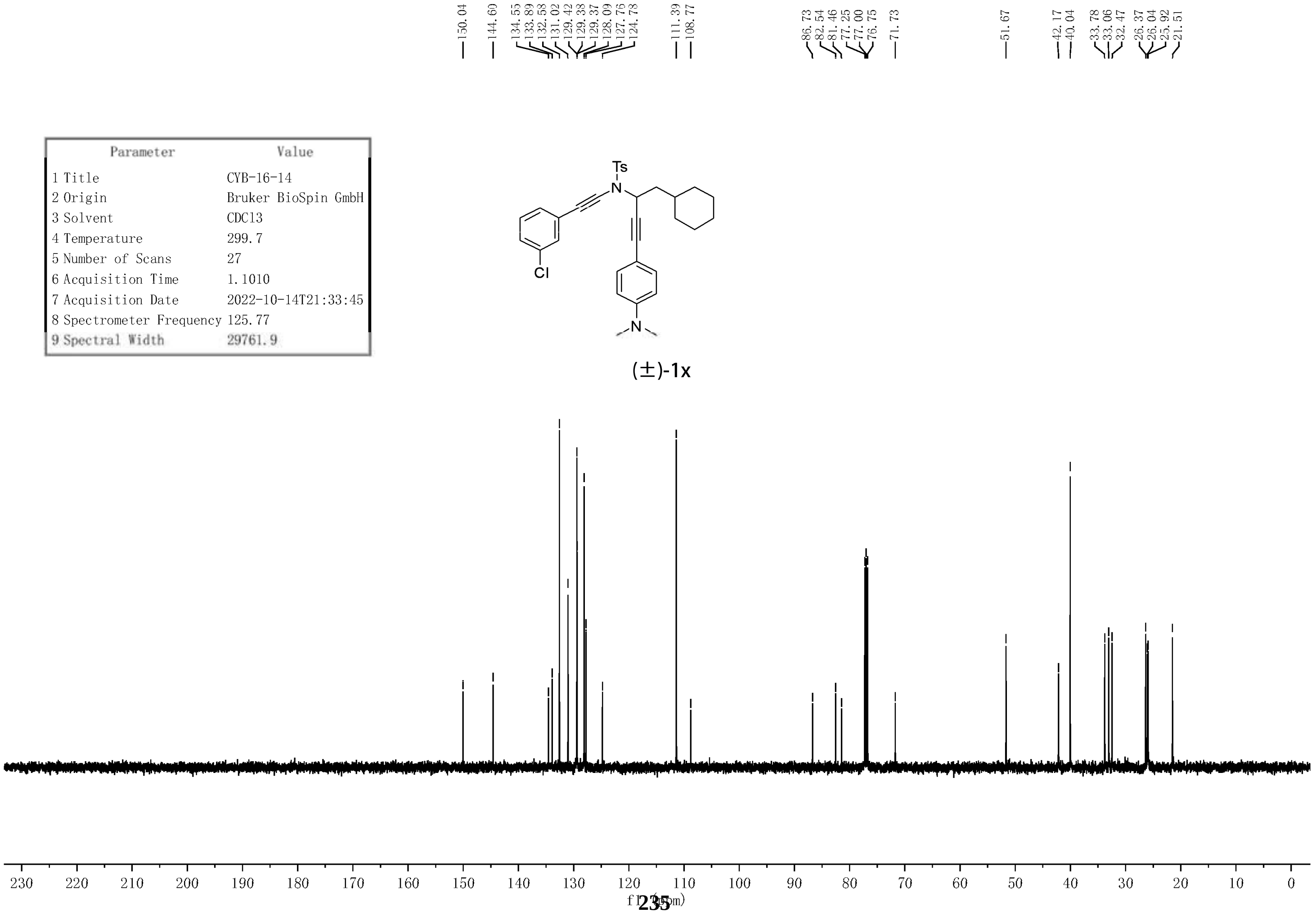

Parameter	Value
1 Title	CYB-16-14
2 Origin	Bruker BioSpin GmbH
3 Solvent	CDCl3
4 Temperature	299.7
5 Number of Scans	27
6 Acquisition Time	1.1010
7 Acquisition Date	2022-10-14T21:33:45
8 Spectrometer Frequency	125.77
9 Spectral Width	29761.9

(±)-1x

Supplementary Figure 135. ^{1}H and ^{13}C NMR spectra for (±)-1x

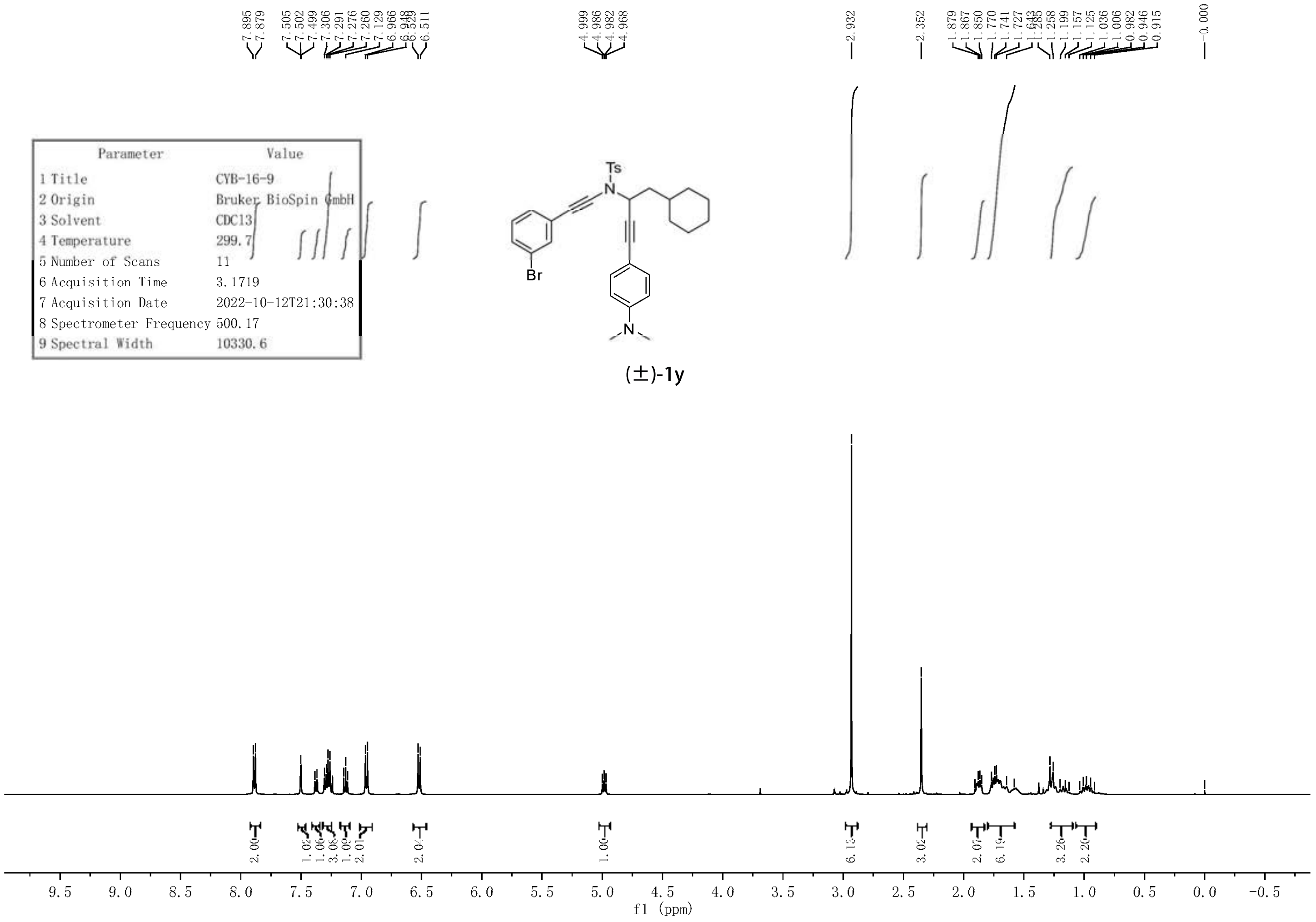

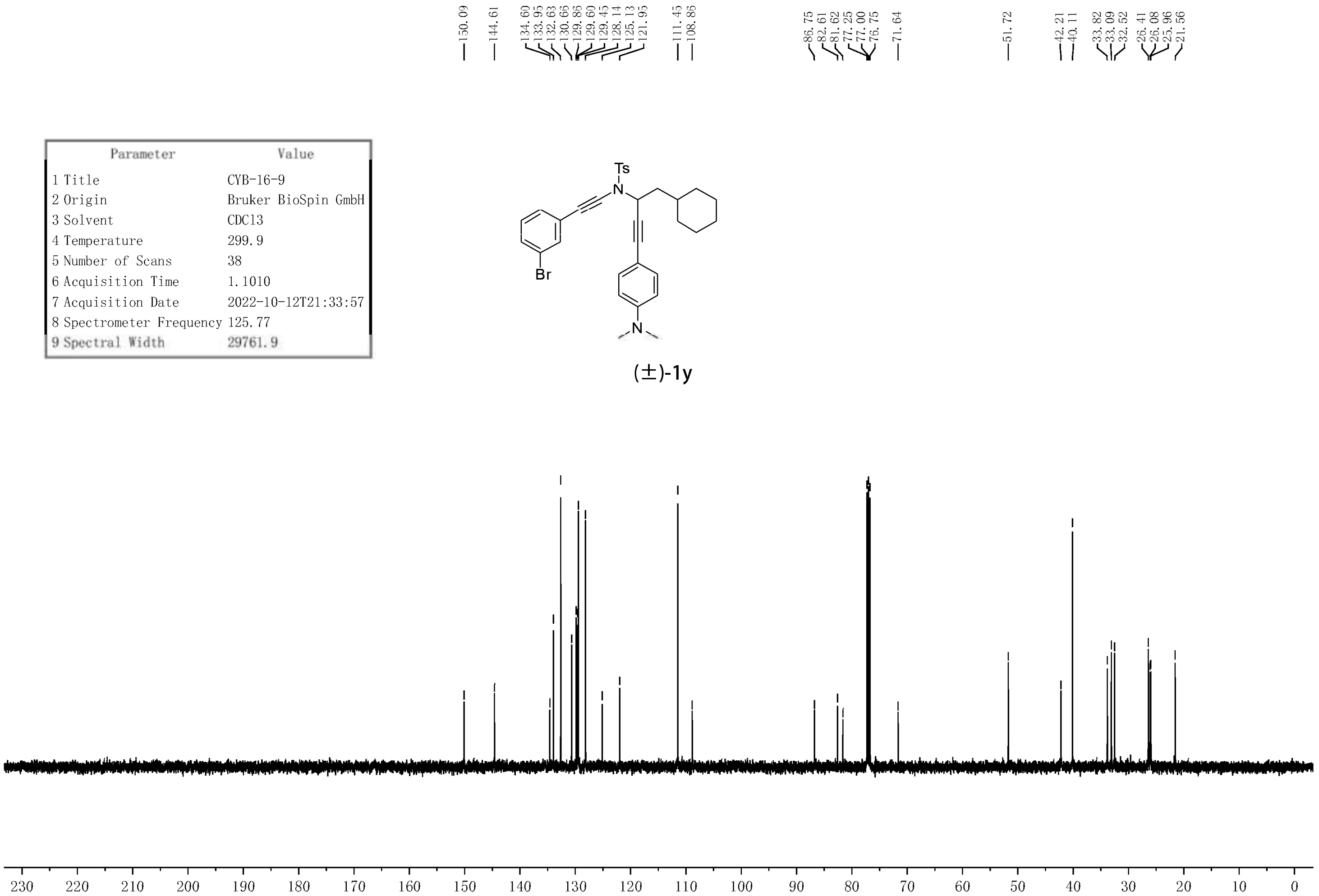

Supplementary Figure 136. ^{1}H and ^{13}C NMR spectra for (±)-1y

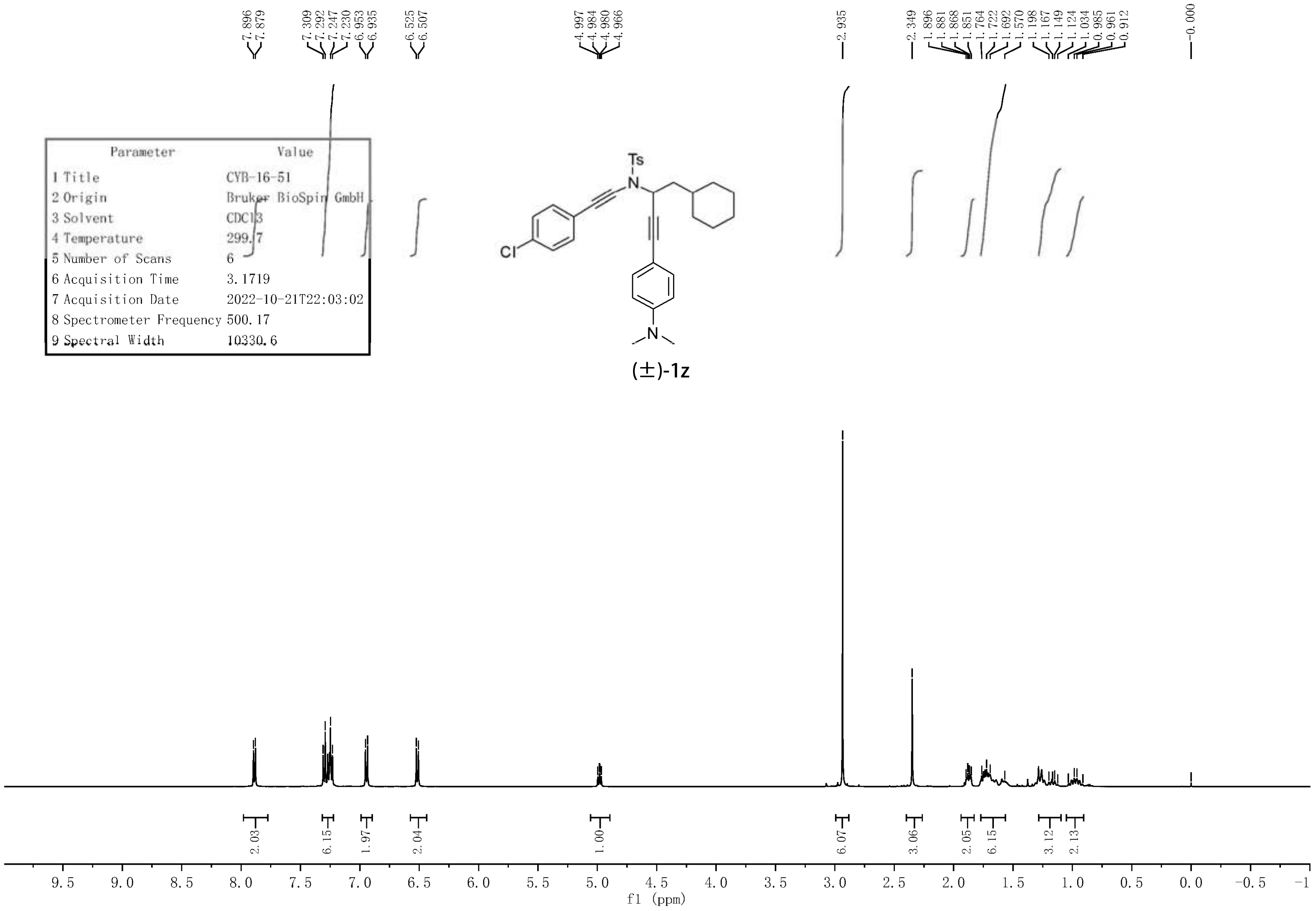

Parameter	Value
1 Title	CYB-16-51
2 Origin	Bruker BioSpin GmbH
3 Solvent	CDCl3
4 Temperature	299.7
5 Number of Scans	6
6 Acquisition Time	3.1719
7 Acquisition Date	2022-10-21T22:03:02
8 Spectrometer Frequency	500.17
9 Spectral Width	10330.6

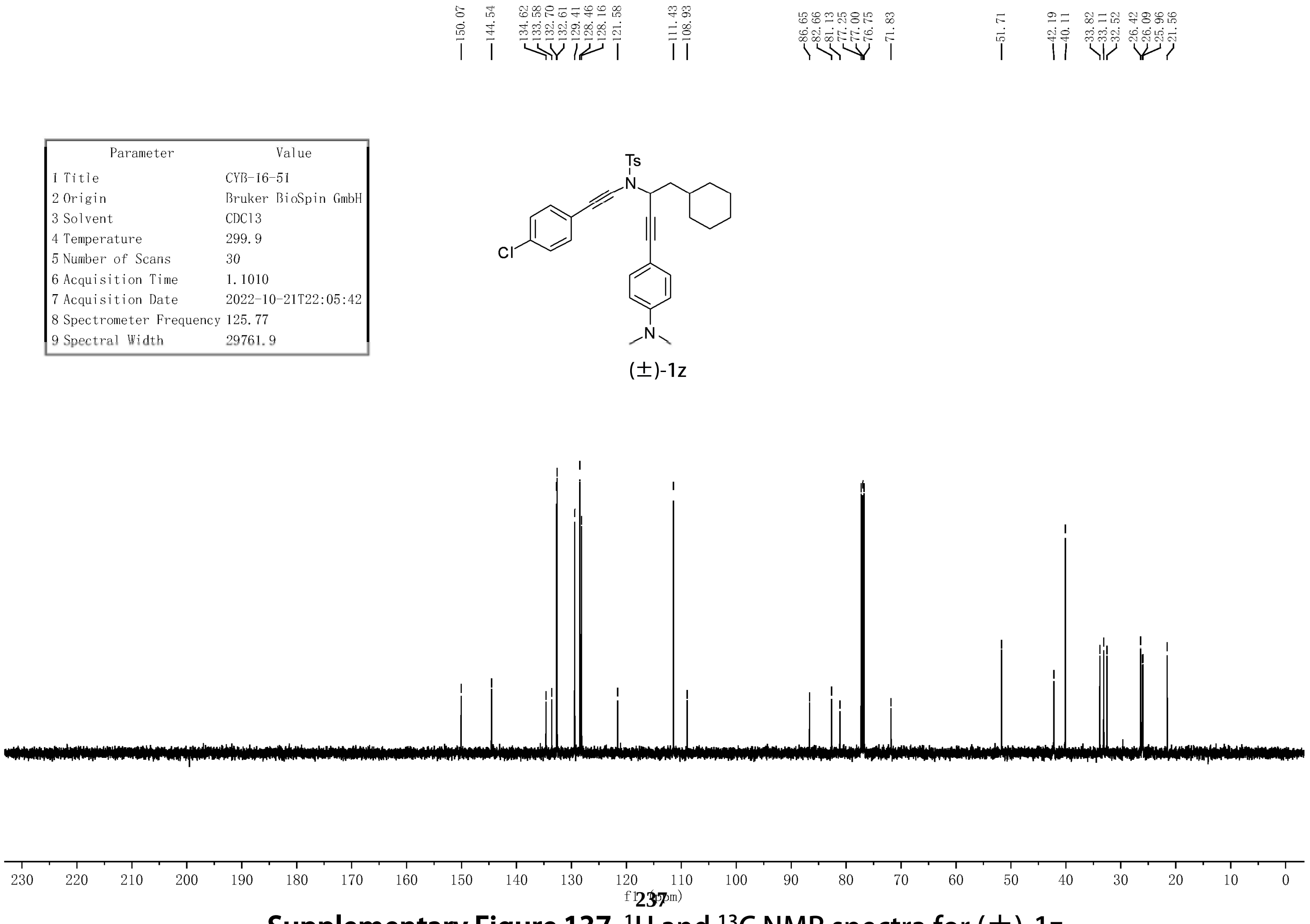

Parameter	Value
1 Title	CYB-16-51
2 Origin	Bruker BioSpin GmbH
3 Solvent	CDCl3
4 Temperature	299.9
5 Number of Scans	30
6 Acquisition Time	1.1010
7 Acquisition Date	2022-10-21T22:05:42
8 Spectrometer Frequency	125.77
9 Spectral Width	29761.9

Supplementary Figure 137. ^{1}H and ^{13}C NMR spectra for (±)-1z

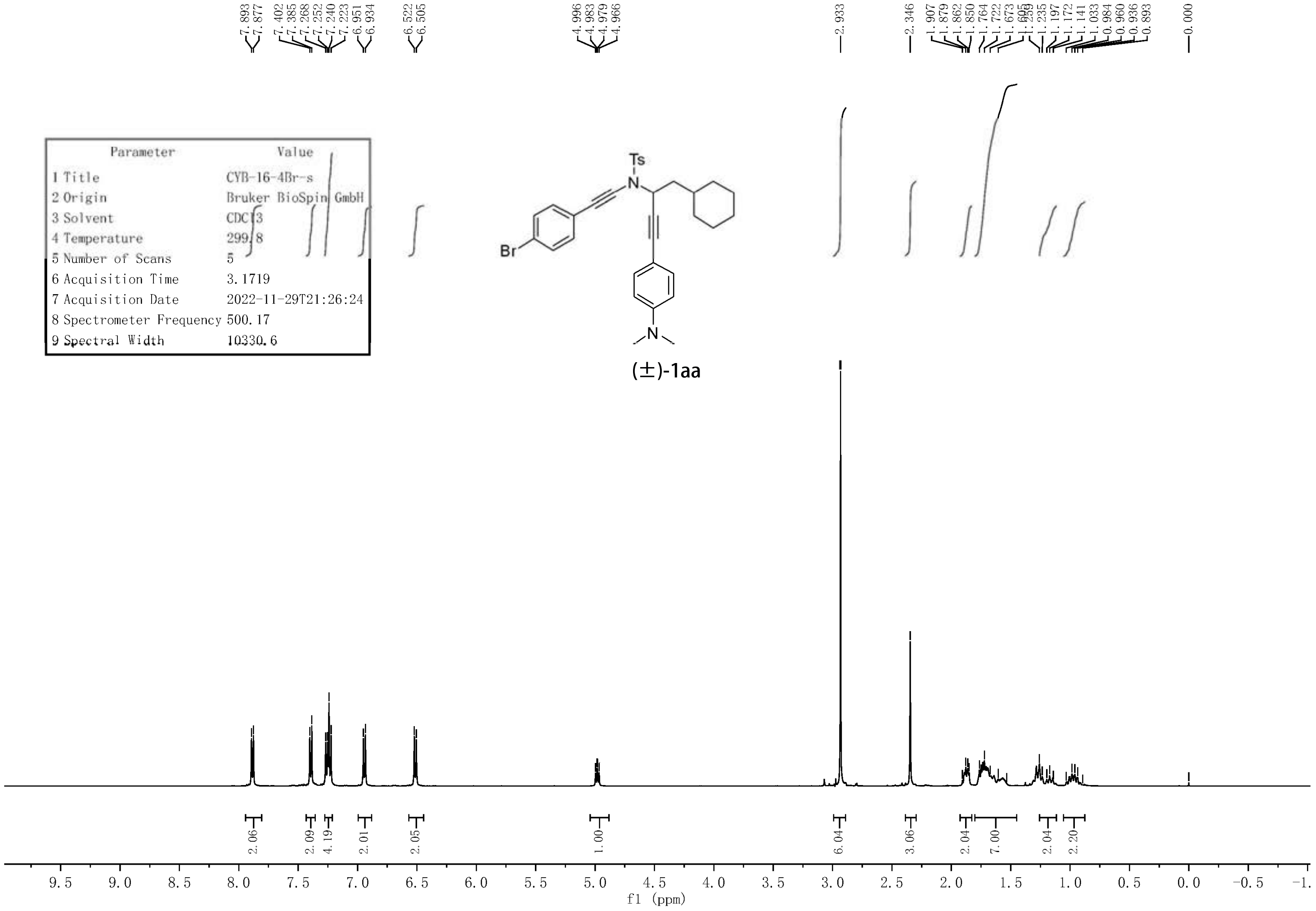

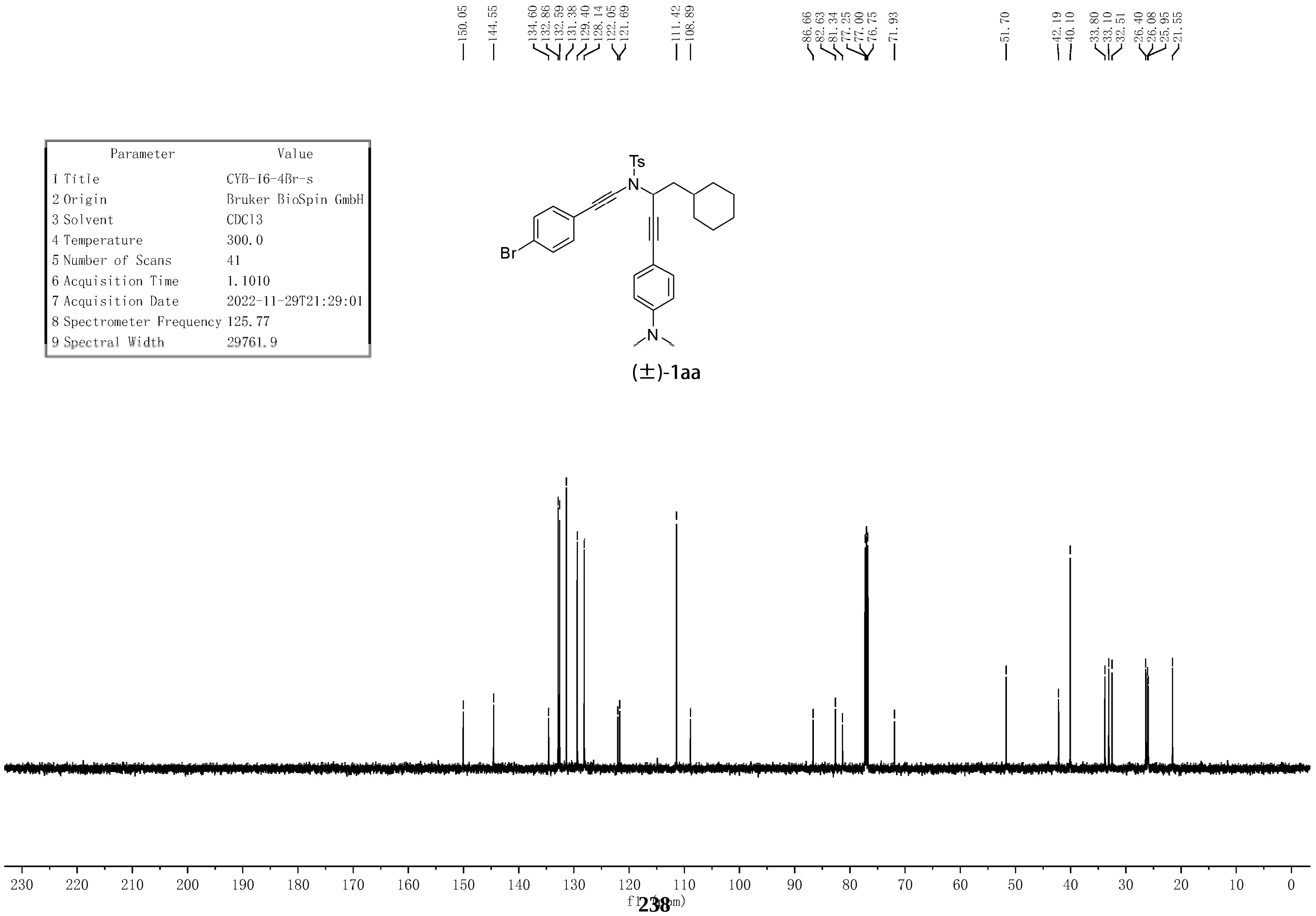

Supplementary Figure 138. ^{1}H and ^{13}C NMR spectra for (±)-1aa

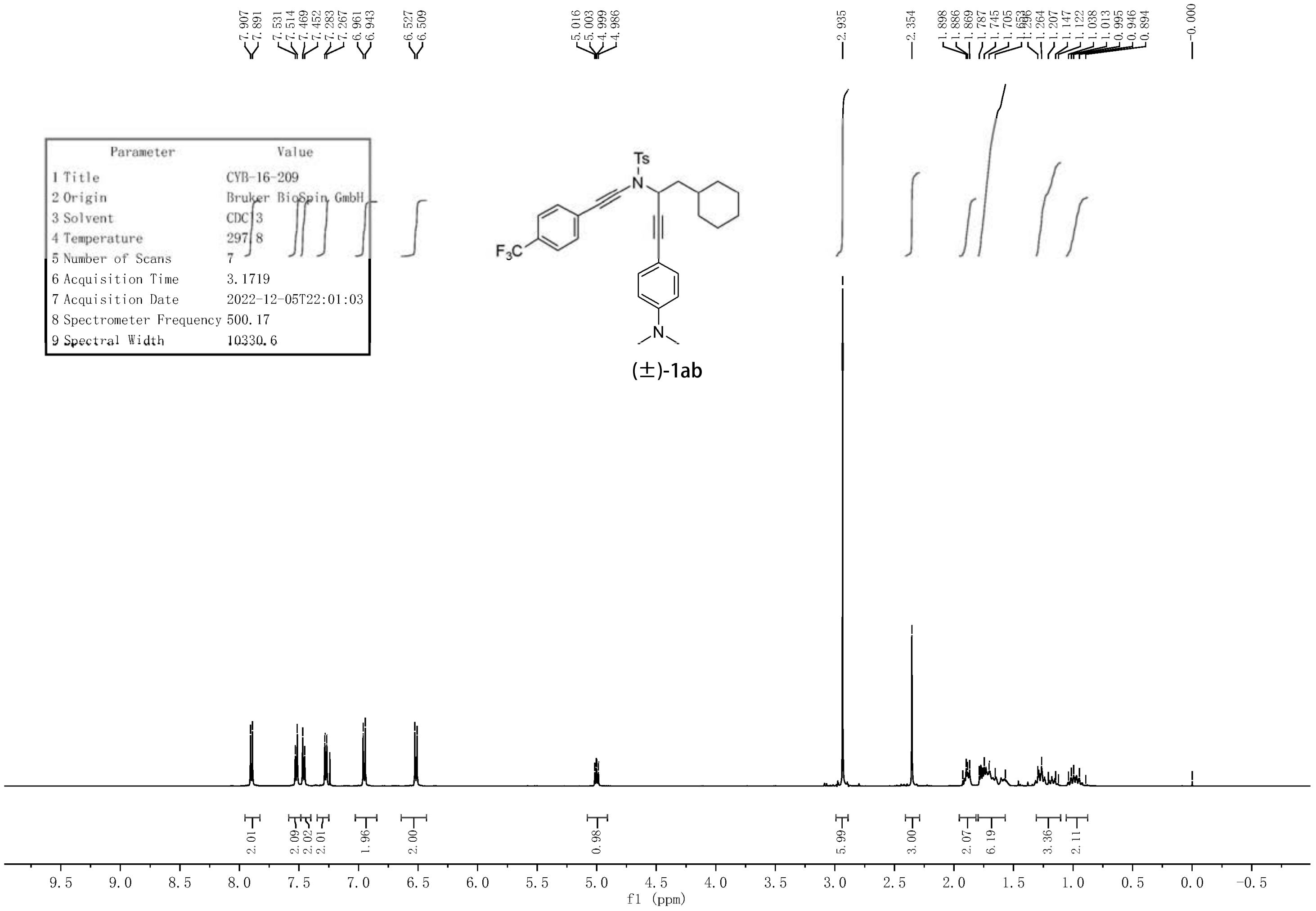

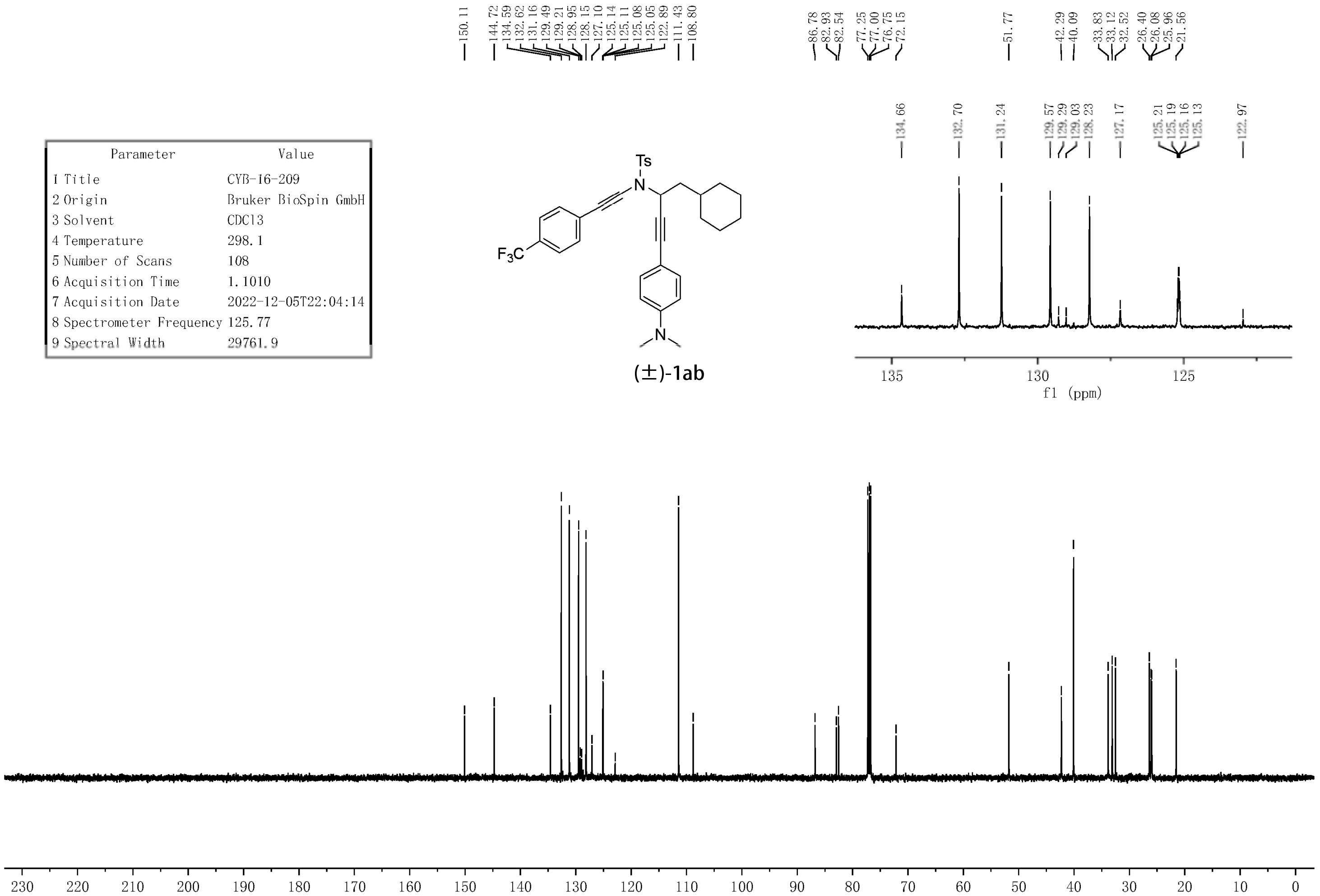

Supplementary Figure 139. ^{1}H and ^{13}C NMR spectra for (±)-1ab

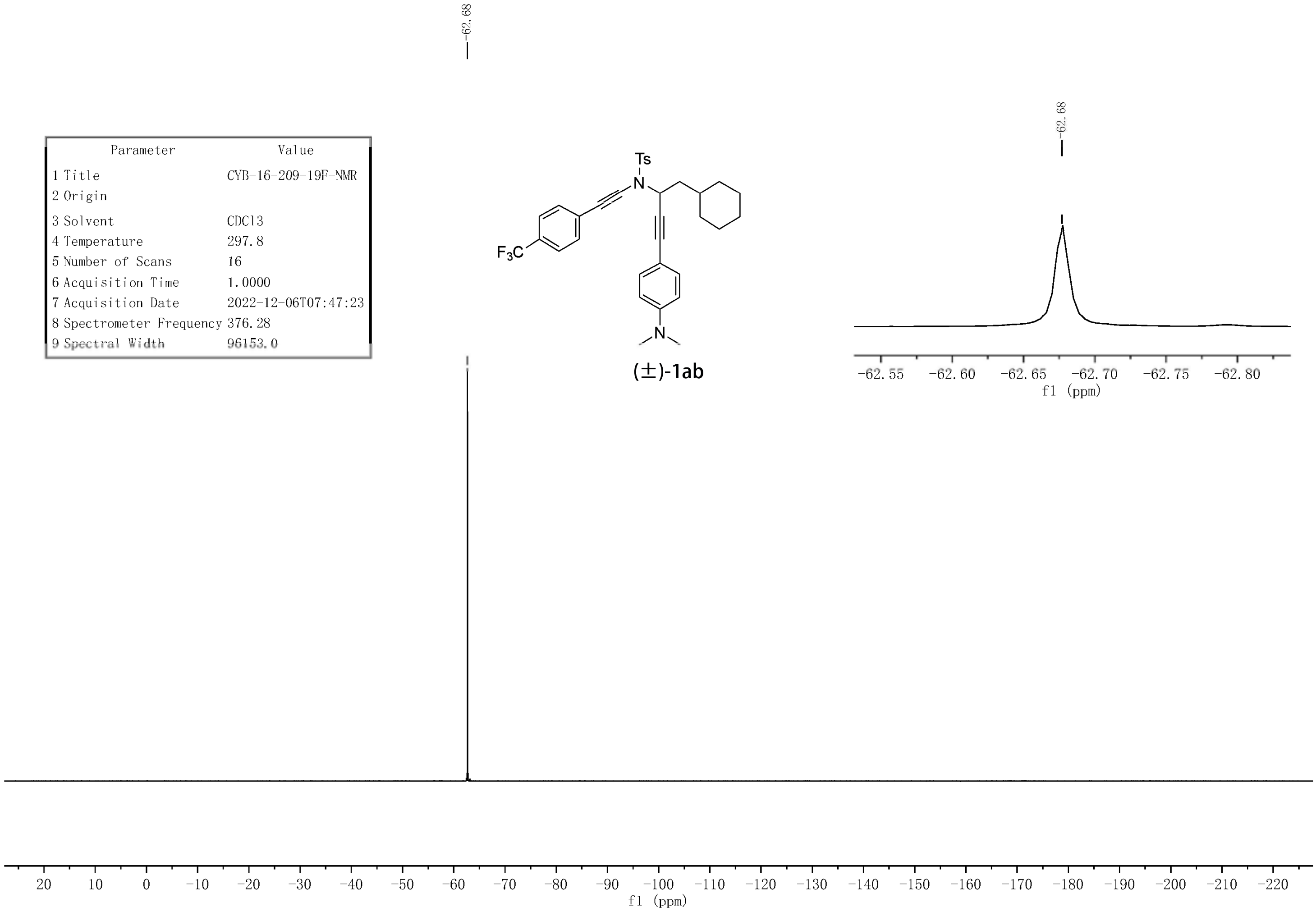

Supplementary Figure 140. ^{19}F NMR spectra for (±)-1ab

Parameter	Value
1 Title	CYB-16-46
2 Origin	Bruker BioSpin GmbH
3 Solvent	CDCl3
4 Temperature	299.5
5 Number of Scans	11
6 Acquisition Time	3.1719
7 Acquisition Date	2022-10-20T22:01:31
8 Spectrometer Frequency	500.17
9 Spectral Width	10330.6

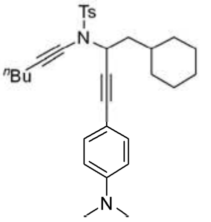

(±)-1ac

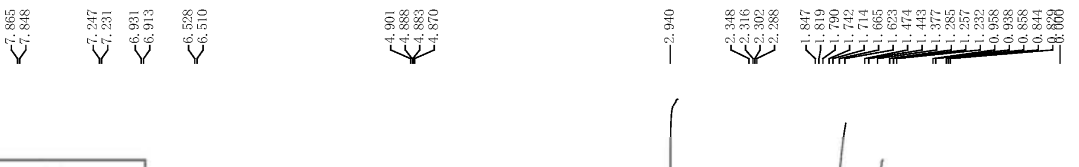
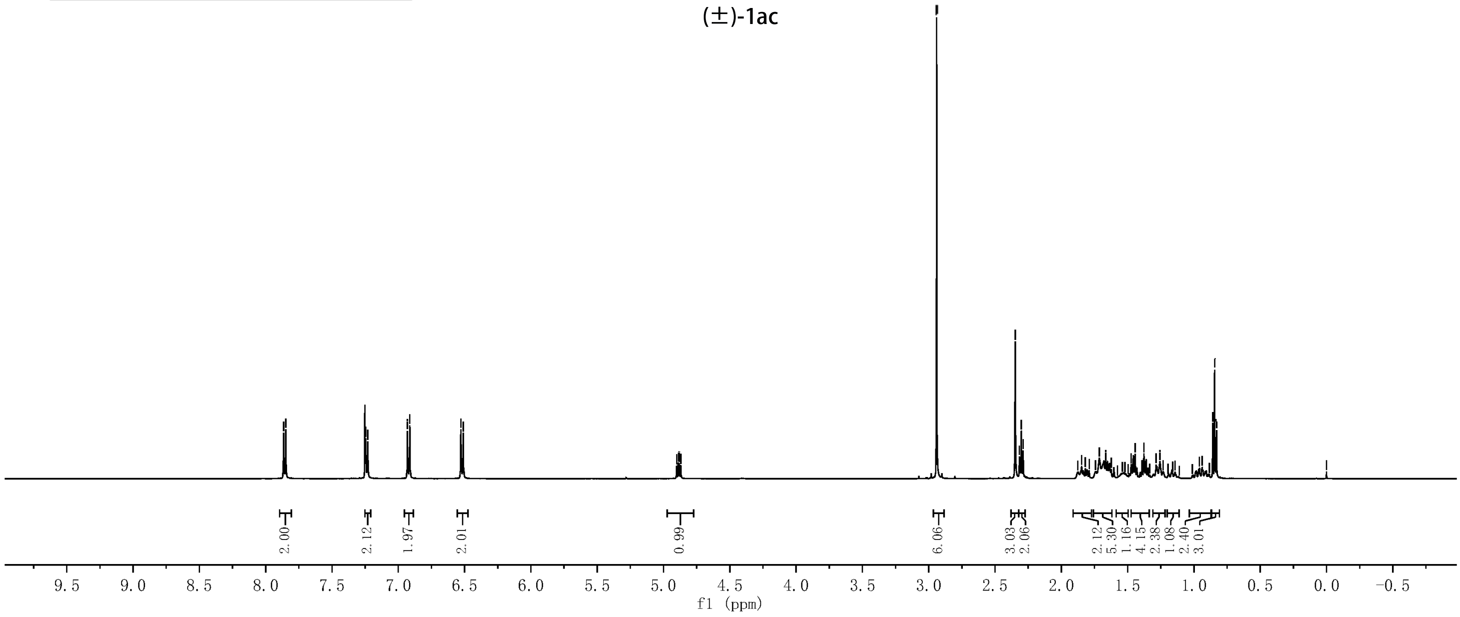

Parameter	Value
1 Title	CYB-16-46
2 Origin	Bruker BioSpin GmbH
3 Solvent	CDCl3
4 Temperature	299.7
5 Number of Scans	42
6 Acquisition Time	1.1010
7 Acquisition Date	2022-10-20T22:04:37
8 Spectrometer Frequency	125.77
9 Spectral Width	29761.9

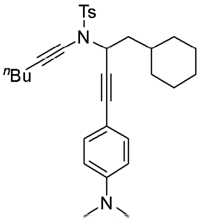

(±)-1ac

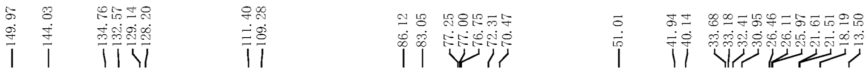
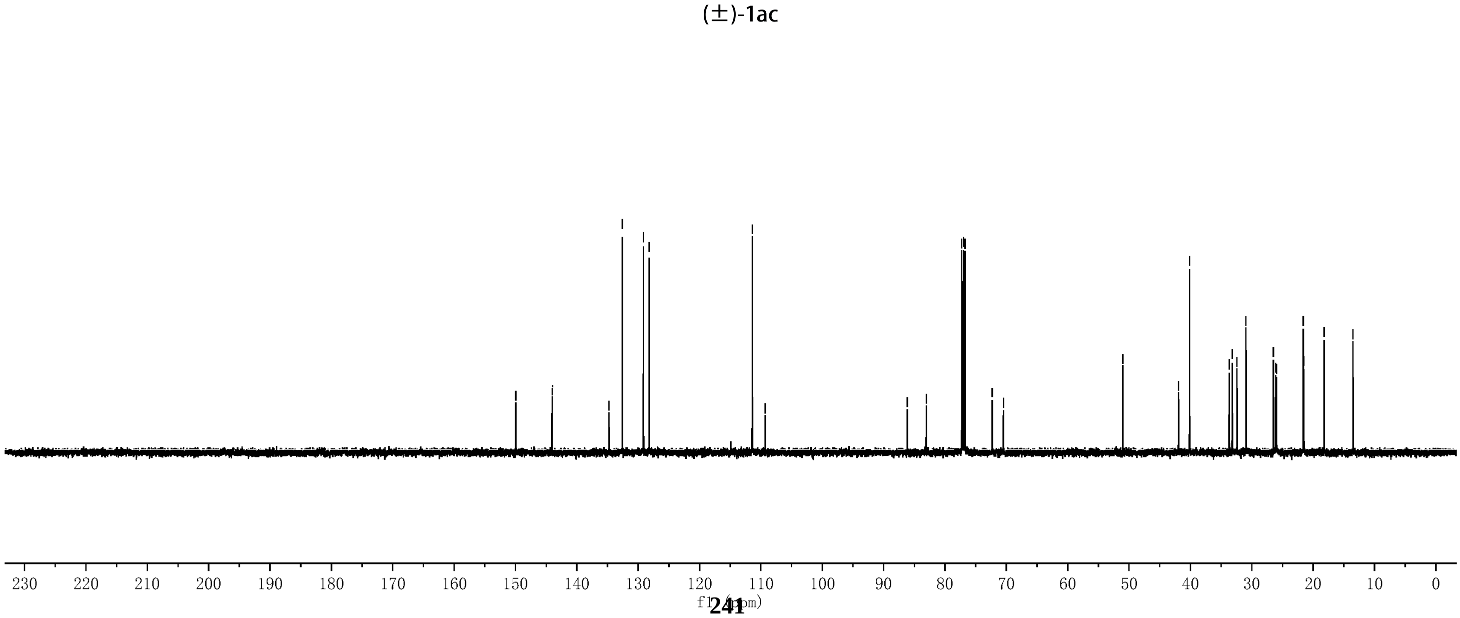

Supplementary Figure 141. ^{1}H and ^{13}C NMR spectra for (±)-1ac

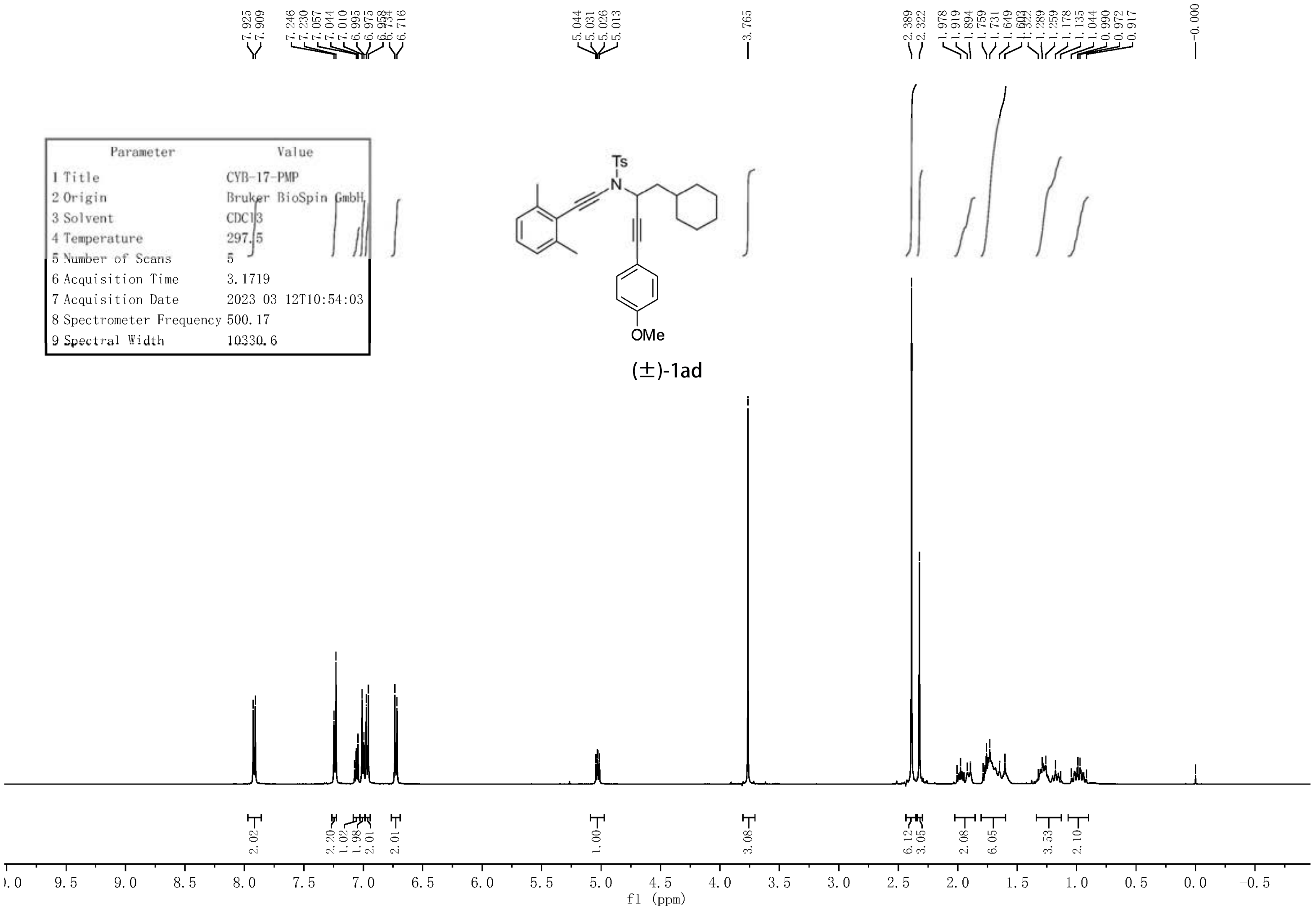

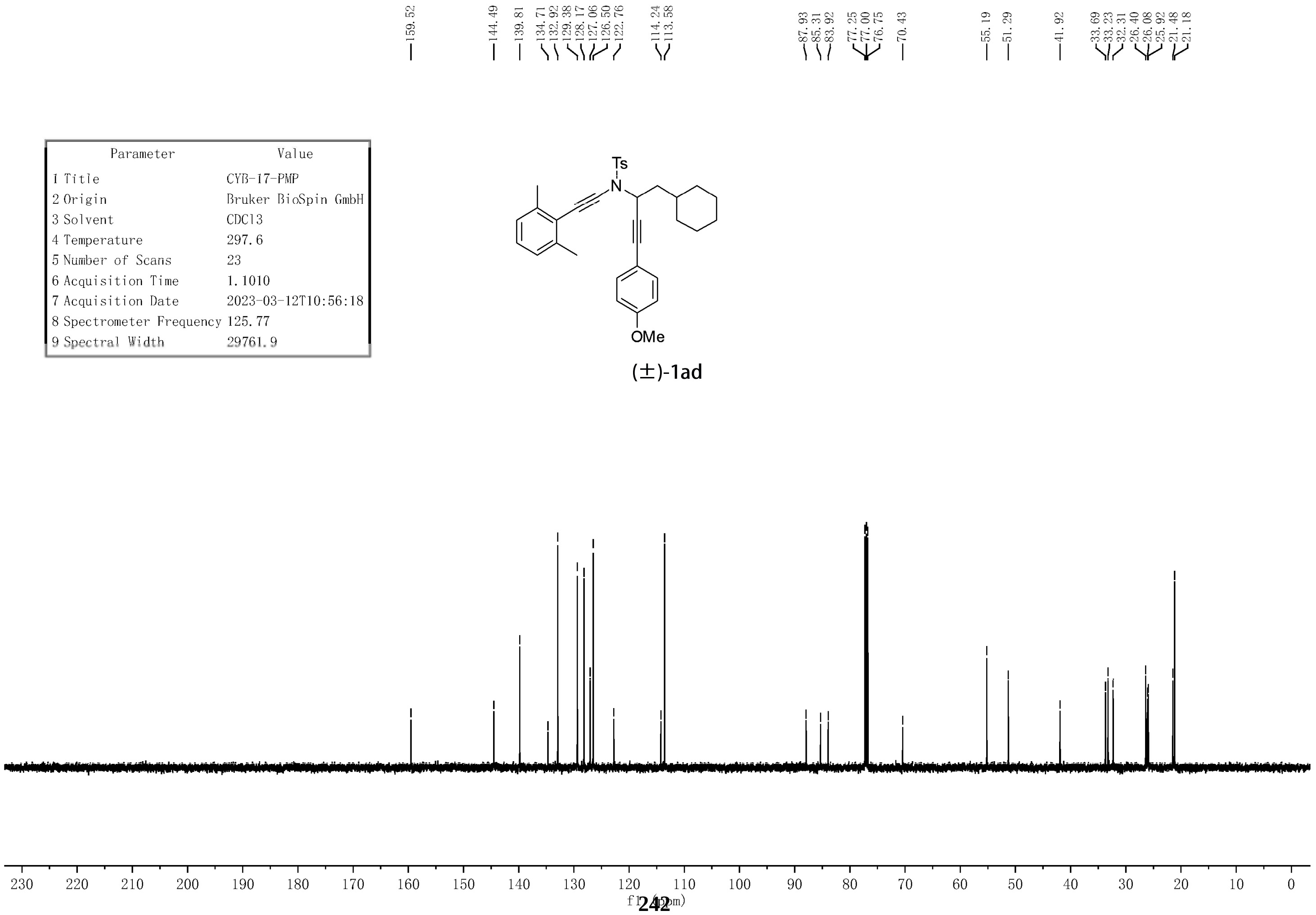

Supplementary Figure 142. ^{1}H and ^{13}C NMR spectra for (±)-1ad

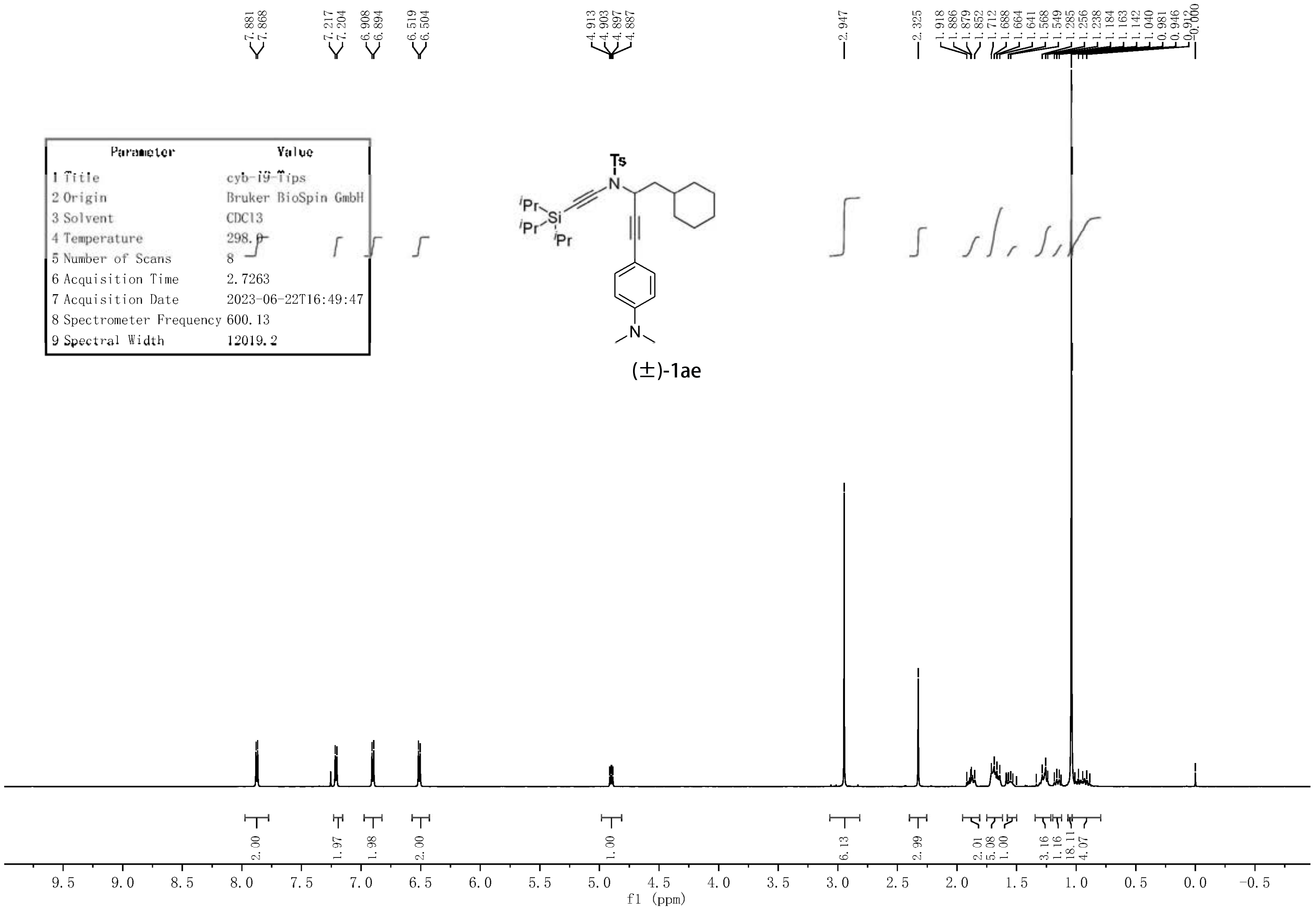

Parameter	Value
1 Title	cyb-19-Tips
2 Origin	Bruker BioSpin GmbH
3 Solvent	CDCl3
4 Temperature	298.0
5 Number of Scans	8
6 Acquisition Time	2.7263
7 Acquisition Date	2023-06-22T16:49:47
8 Spectrometer Frequency	600.13
9 Spectral Width	12019.2

(±)-1ae

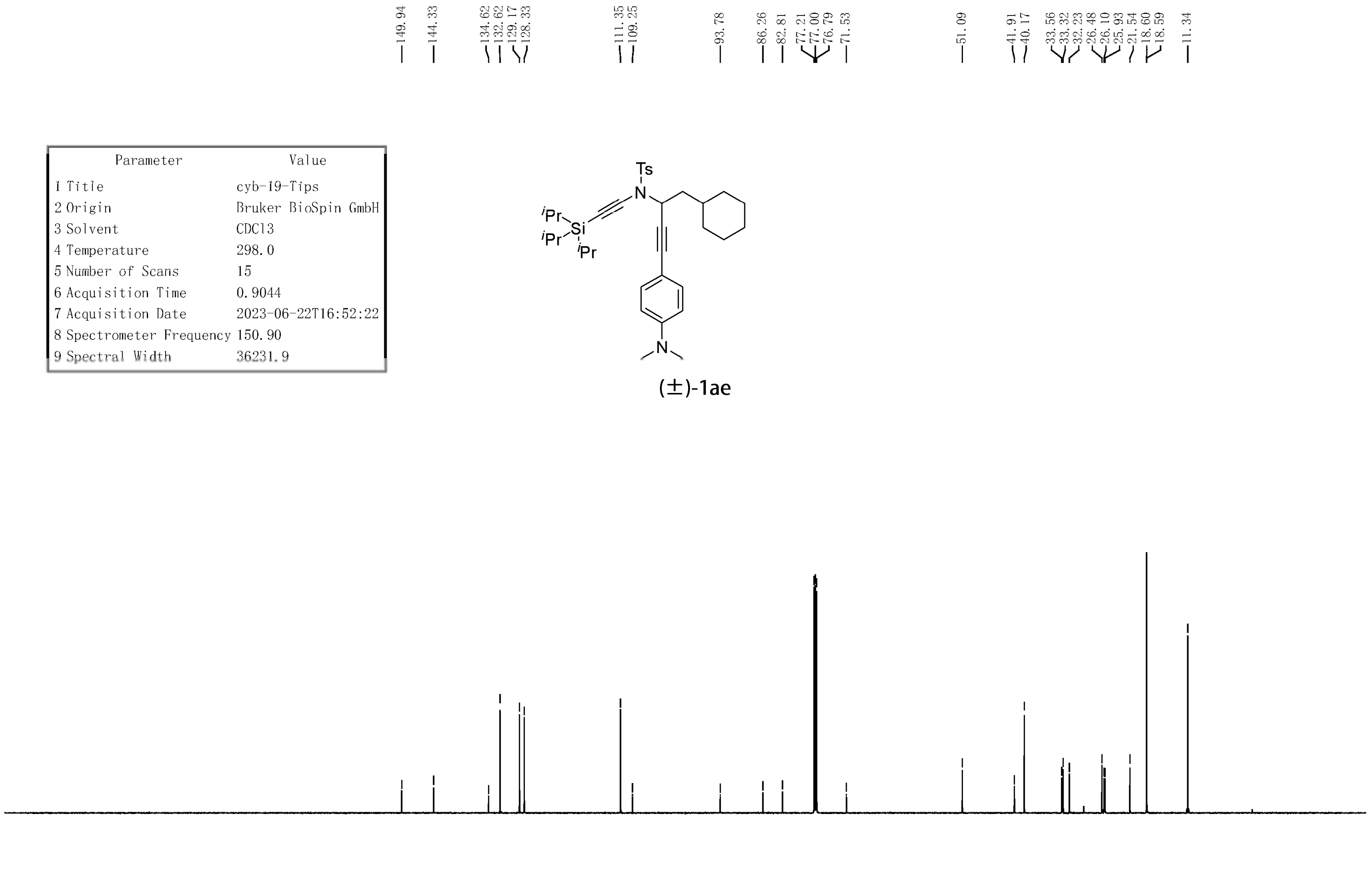

Parameter	Value
1 Title	cyb-19-Tips
2 Origin	Bruker BioSpin GmbH
3 Solvent	CDCl3
4 Temperature	298.0
5 Number of Scans	15
6 Acquisition Time	0.9044
7 Acquisition Date	2023-06-22T16:52:22
8 Spectrometer Frequency	150.90
9 Spectral Width	36231.9

(±)-1ae

Supplementary Figure 143. ^{1}H and ^{13}C NMR spectra for (±)-1ae

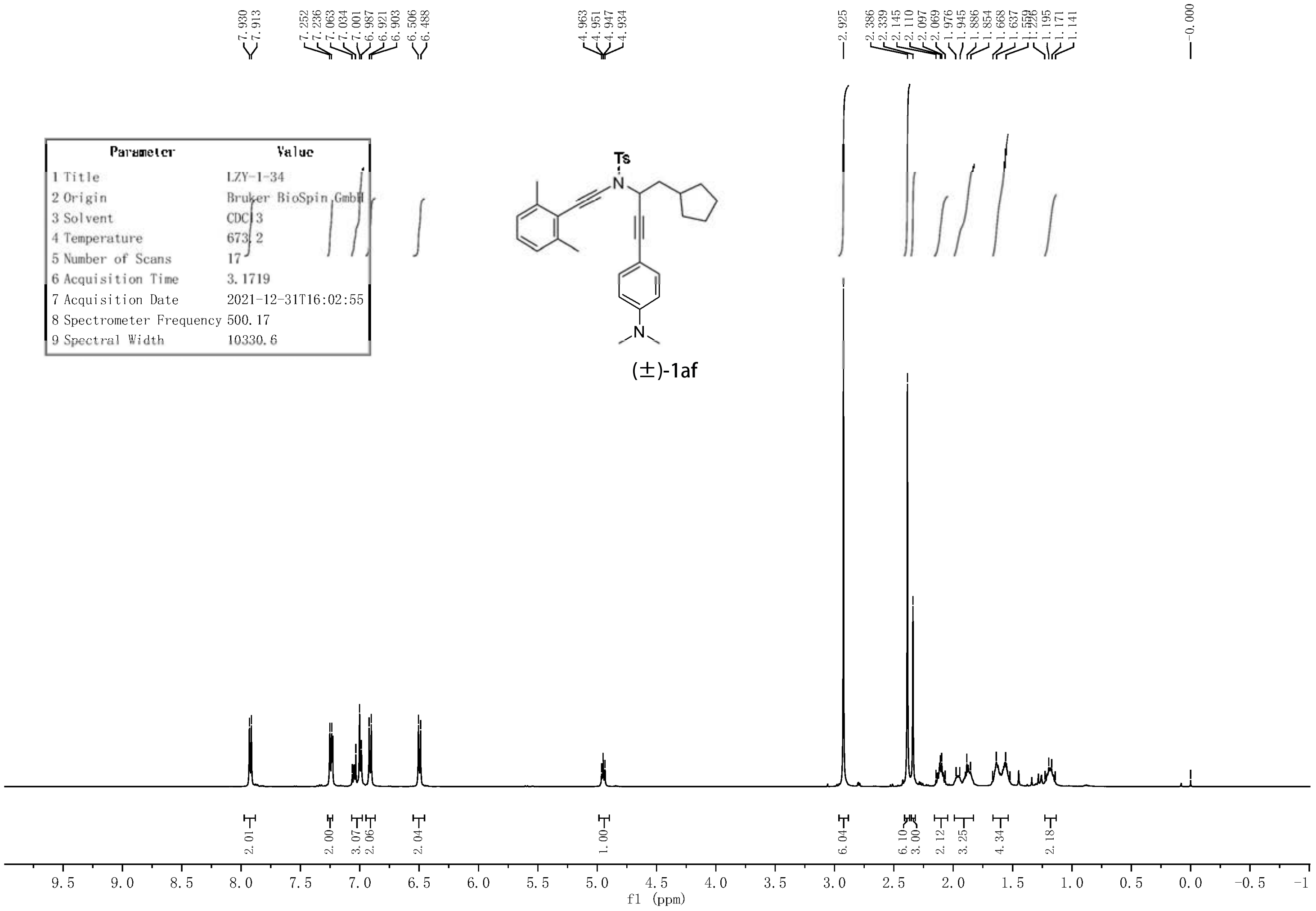

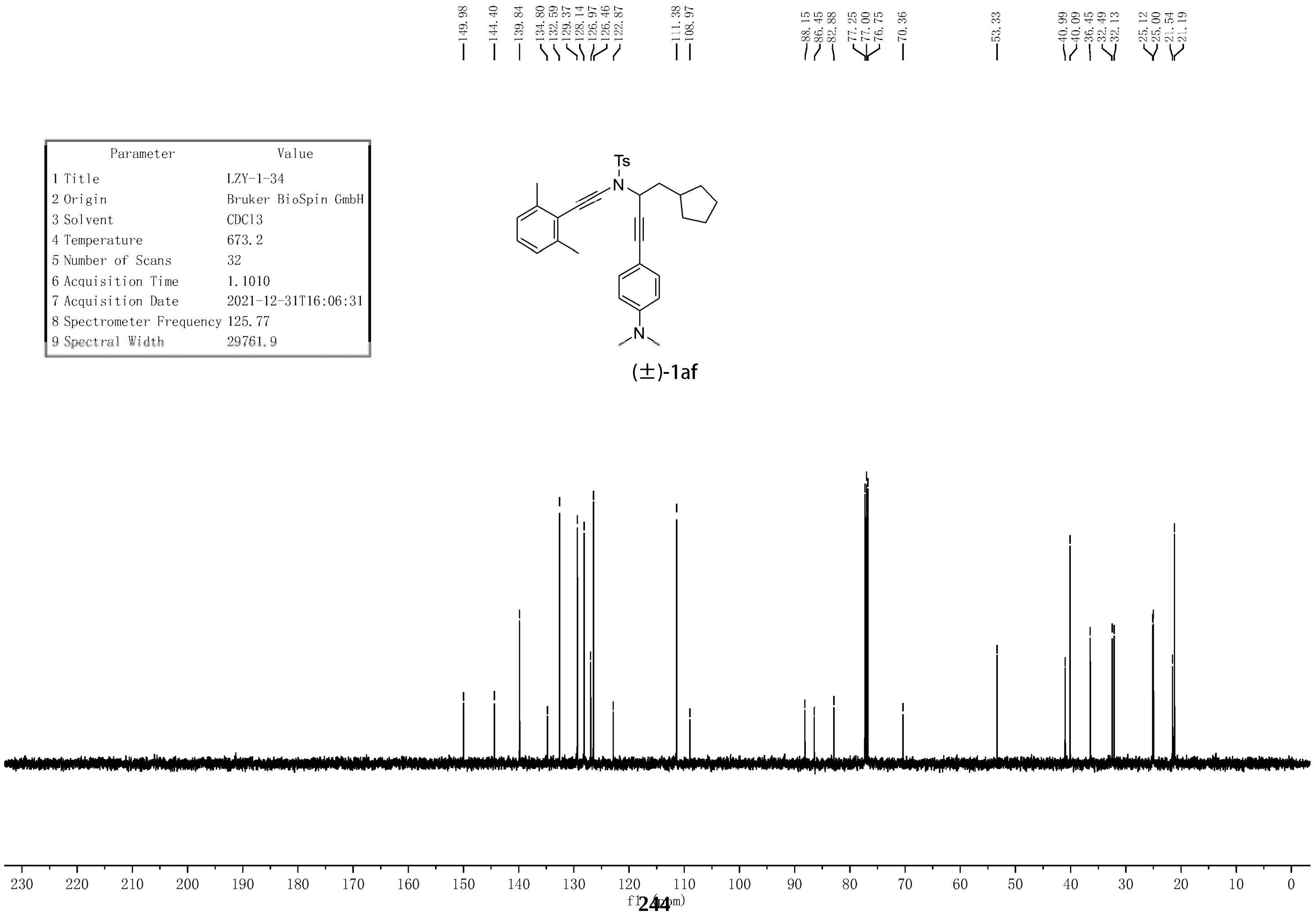

Supplementary Figure 144. ^{1}H and ^{13}C NMR spectra for (±)-1af

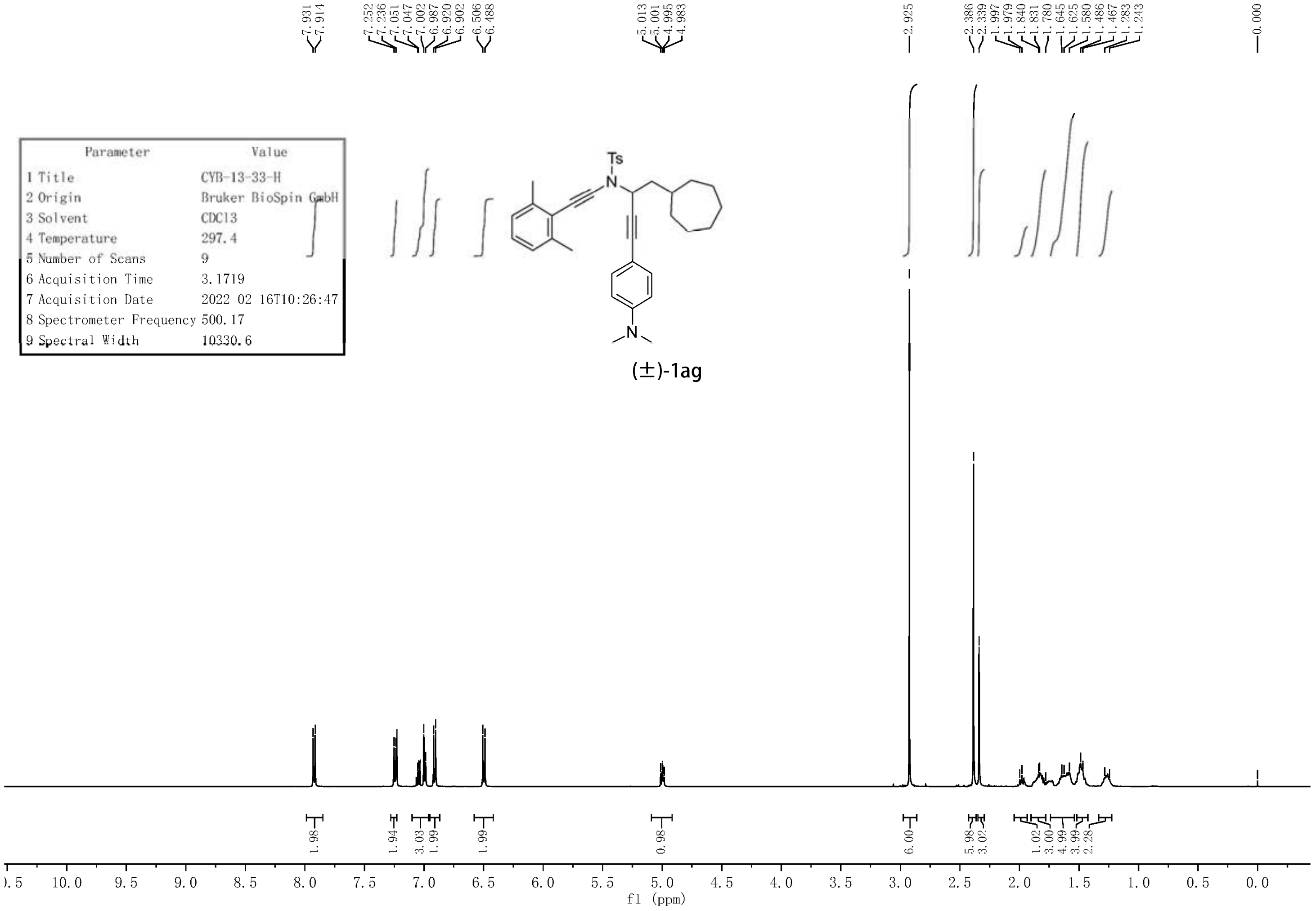

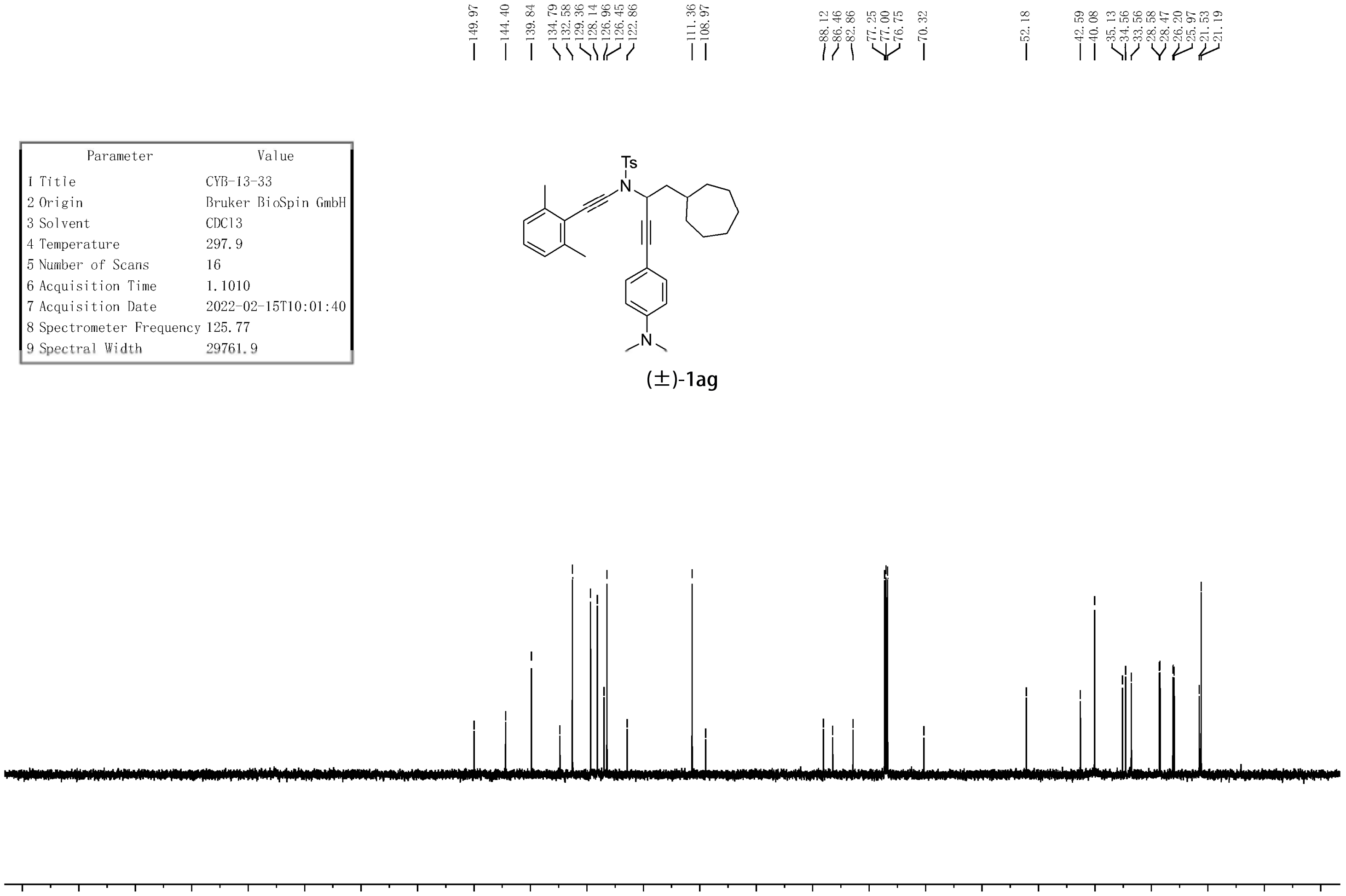

Supplementary Figure 145. ^{1}H and ^{13}C NMR spectra for (±)-1ag

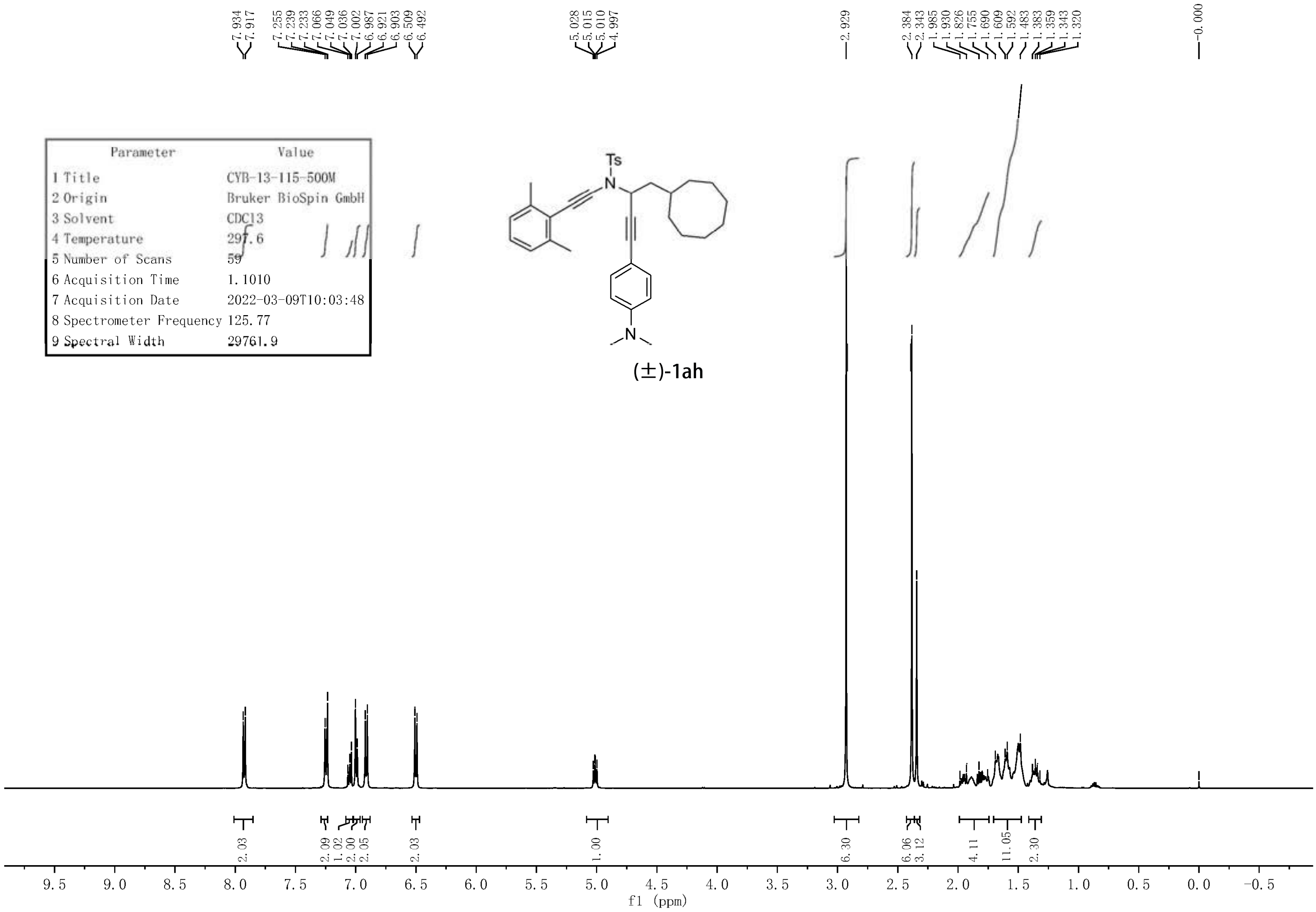

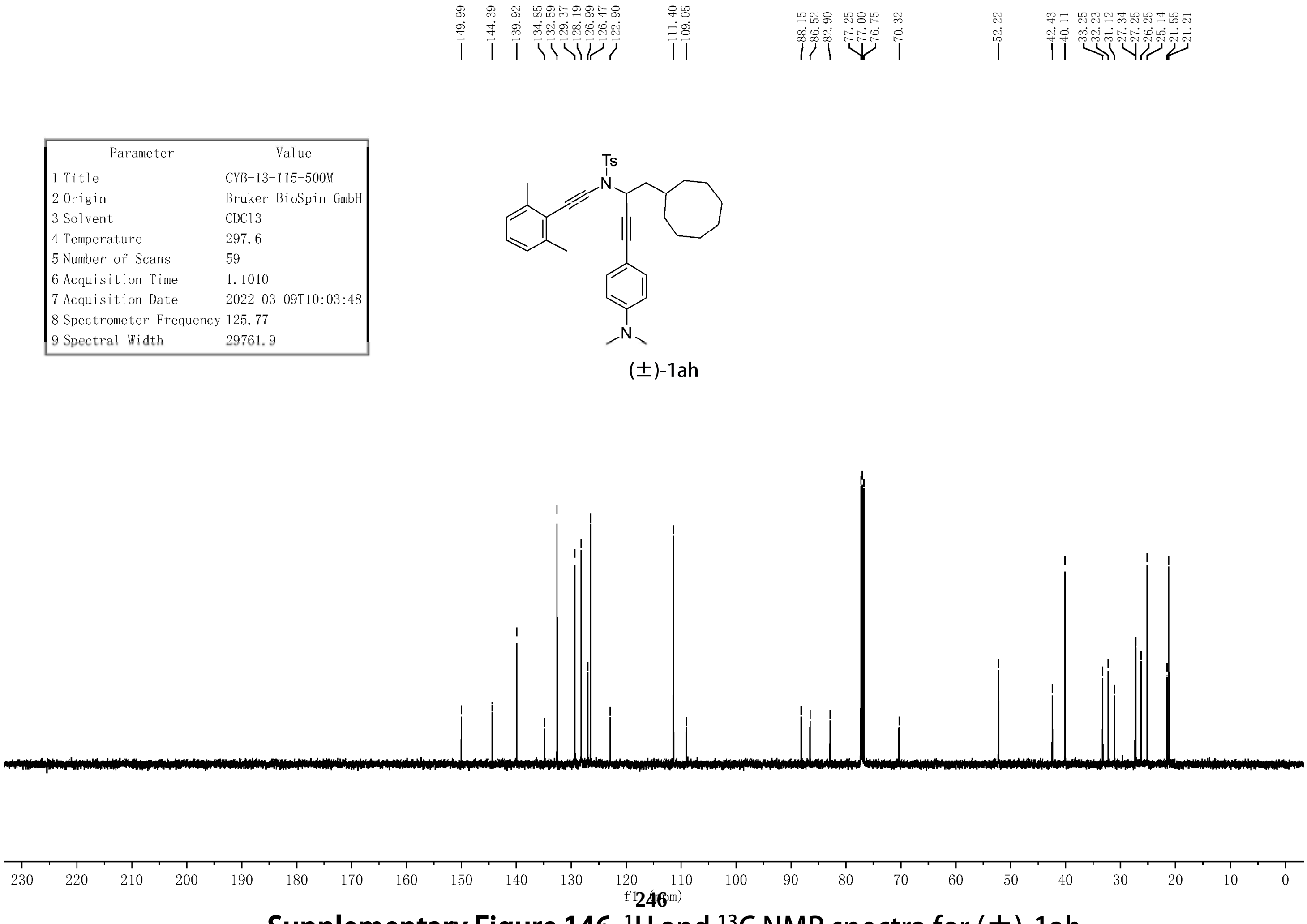

Supplementary Figure 146. ^{1}H and ^{13}C NMR spectra for (±)-1ah

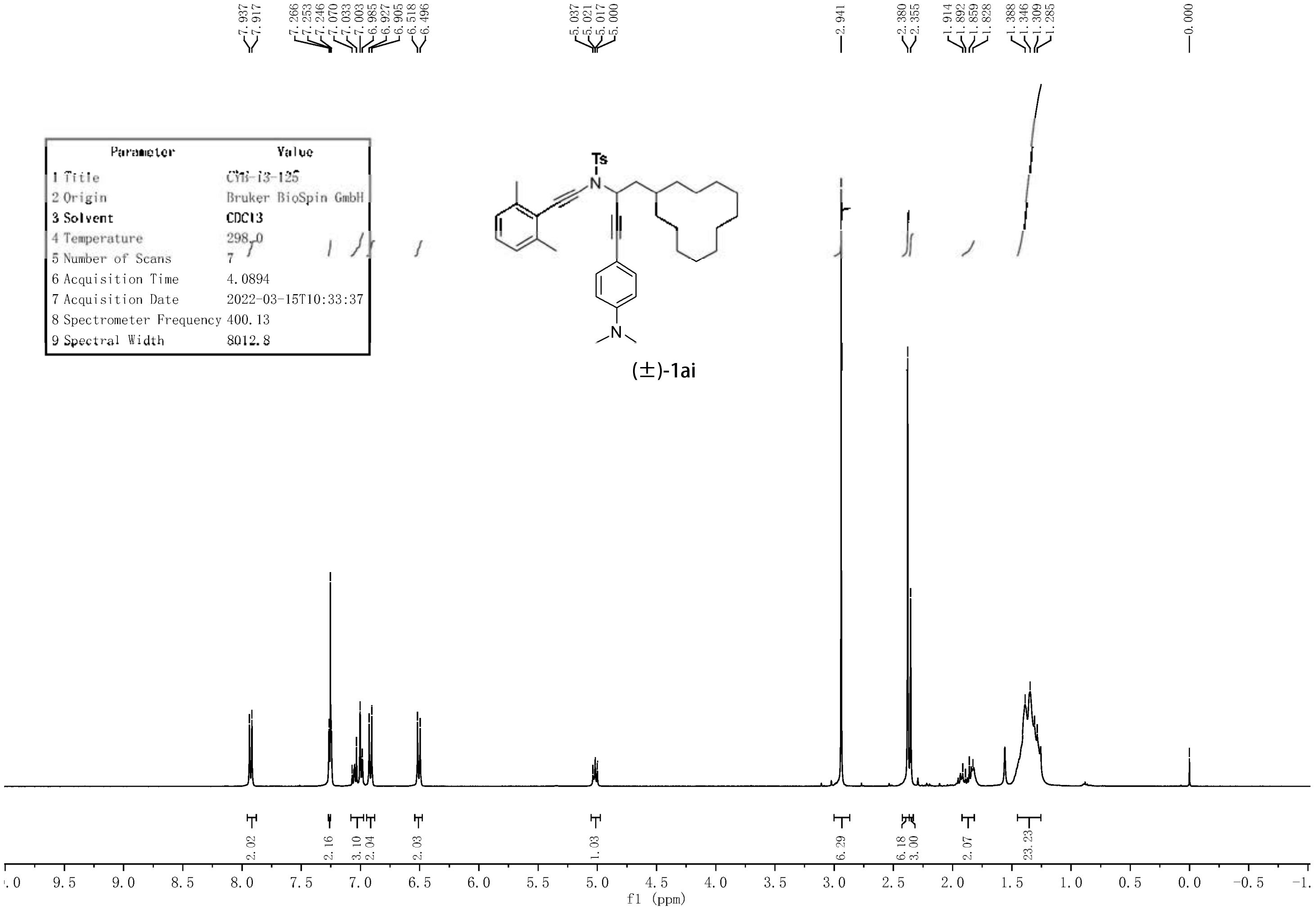

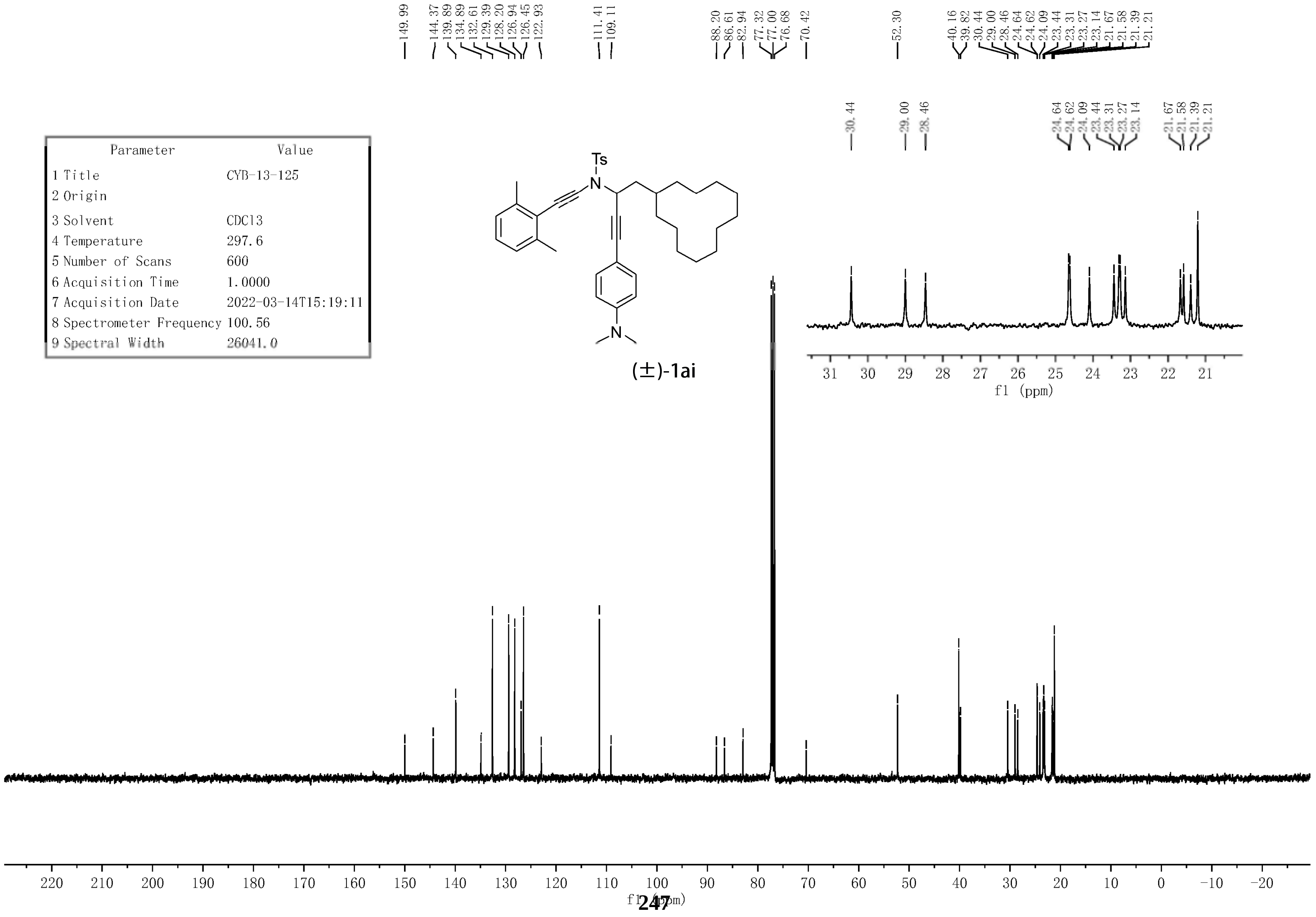

Supplementary Figure 147. ^{1}H and ^{13}C NMR spectra for (±)-1ai

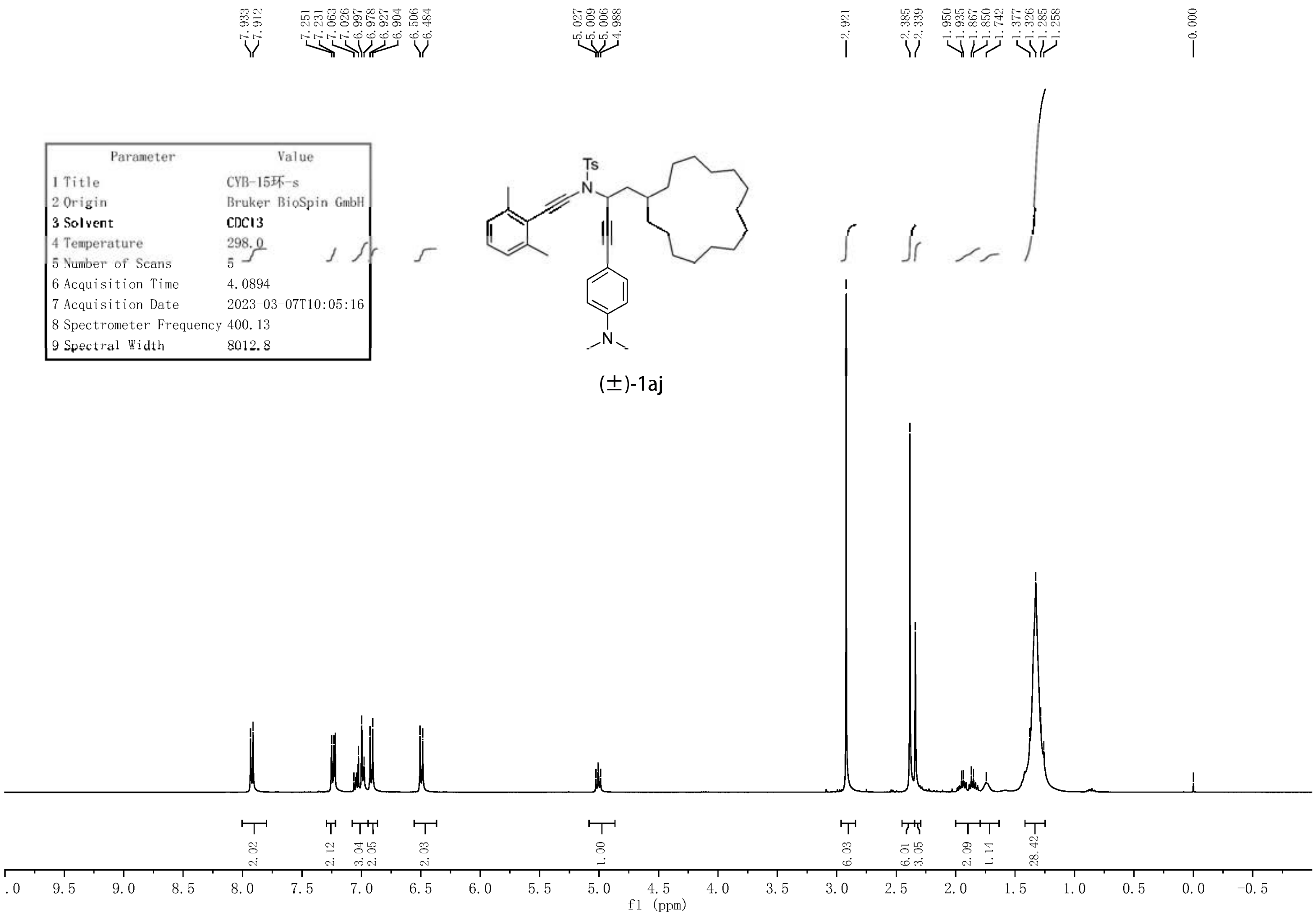

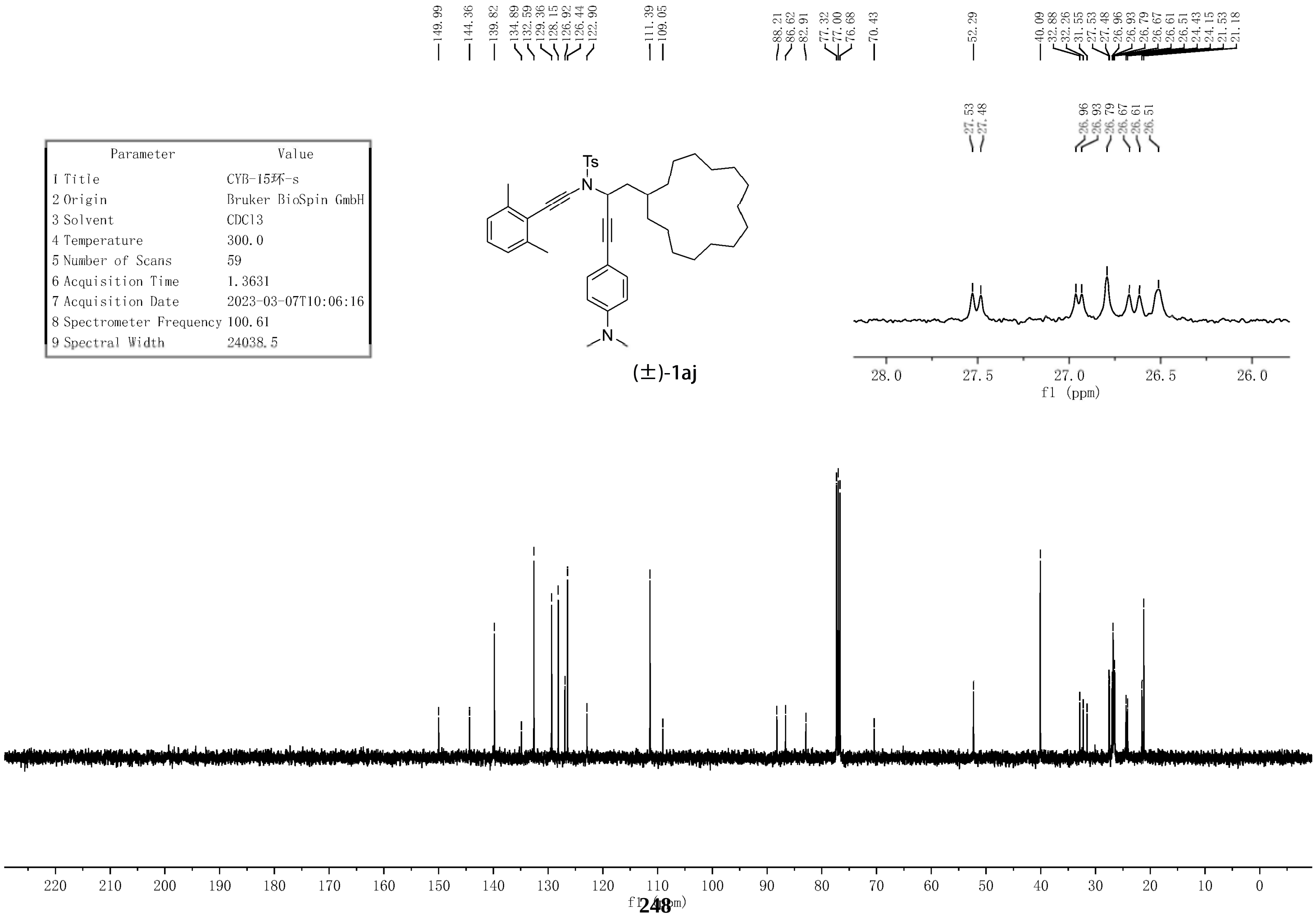

Supplementary Figure 148. ^{1}H and ^{13}C NMR spectra for (±)-1aj

Parameter	Value
1 Title	CYB-13-TsCy(OCH2CH2O)-s
2 Origin	Bruker BioSpin GmbH
3 Solvent	CDCl3
4 Temperature	297.7
5 Number of Scans	37
6 Acquisition Time	1.1010
7 Acquisition Date	2022-03-29T21:50:09
8 Spectrometer Frequency	125.77
9 Spectral Width	29761.9

(±)-1ak

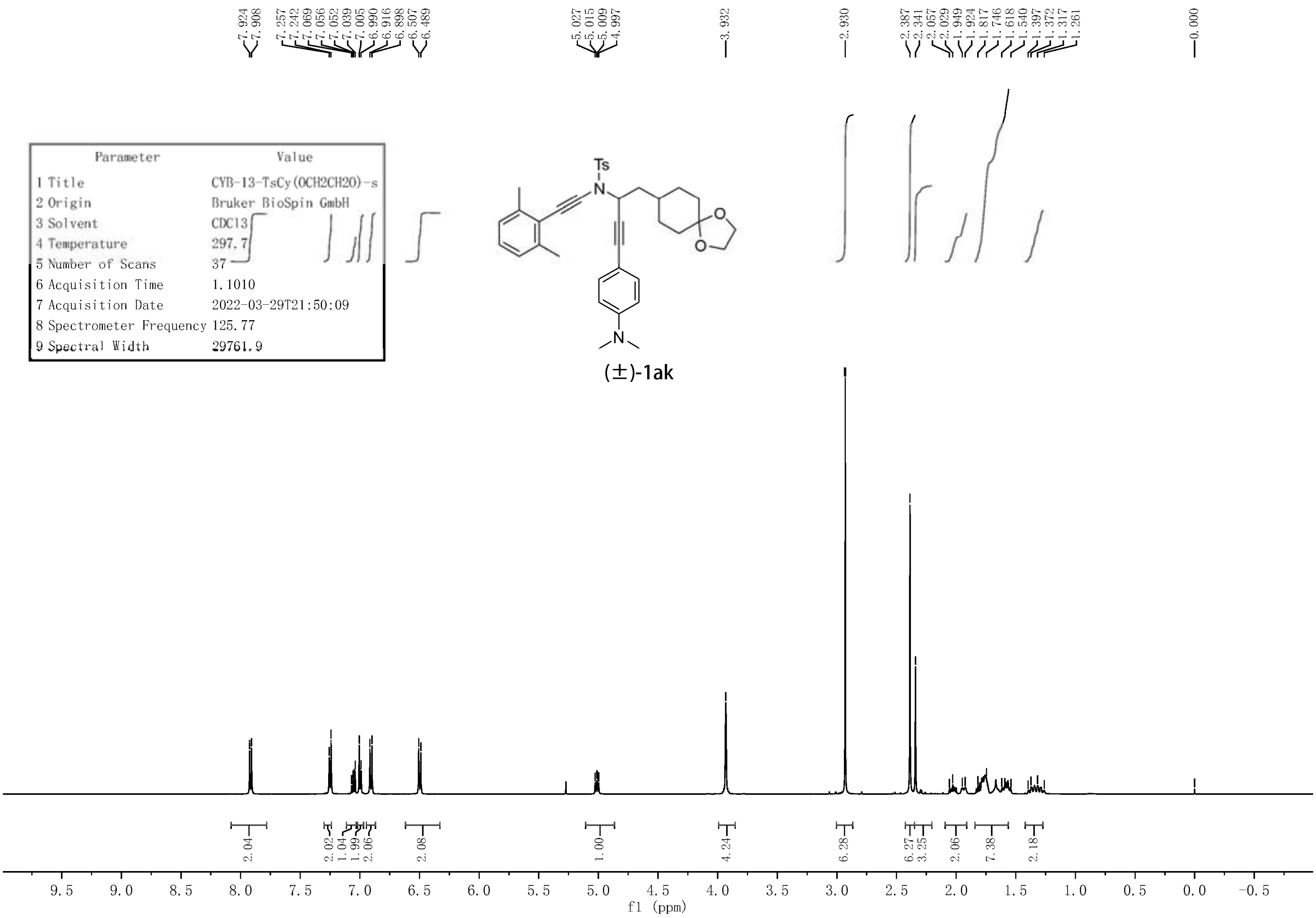

Parameter	Value
1 Title	CYB-13-TsCy(OCH2CH2O)-s
2 Origin	Bruker BioSpin GmbH
3 Solvent	CDCl3
4 Temperature	297.7
5 Number of Scans	37
6 Acquisition Time	1.1010
7 Acquisition Date	2022-03-29T21:50:09
8 Spectrometer Frequency	125.77
9 Spectral Width	29761.9

(±)-1ak

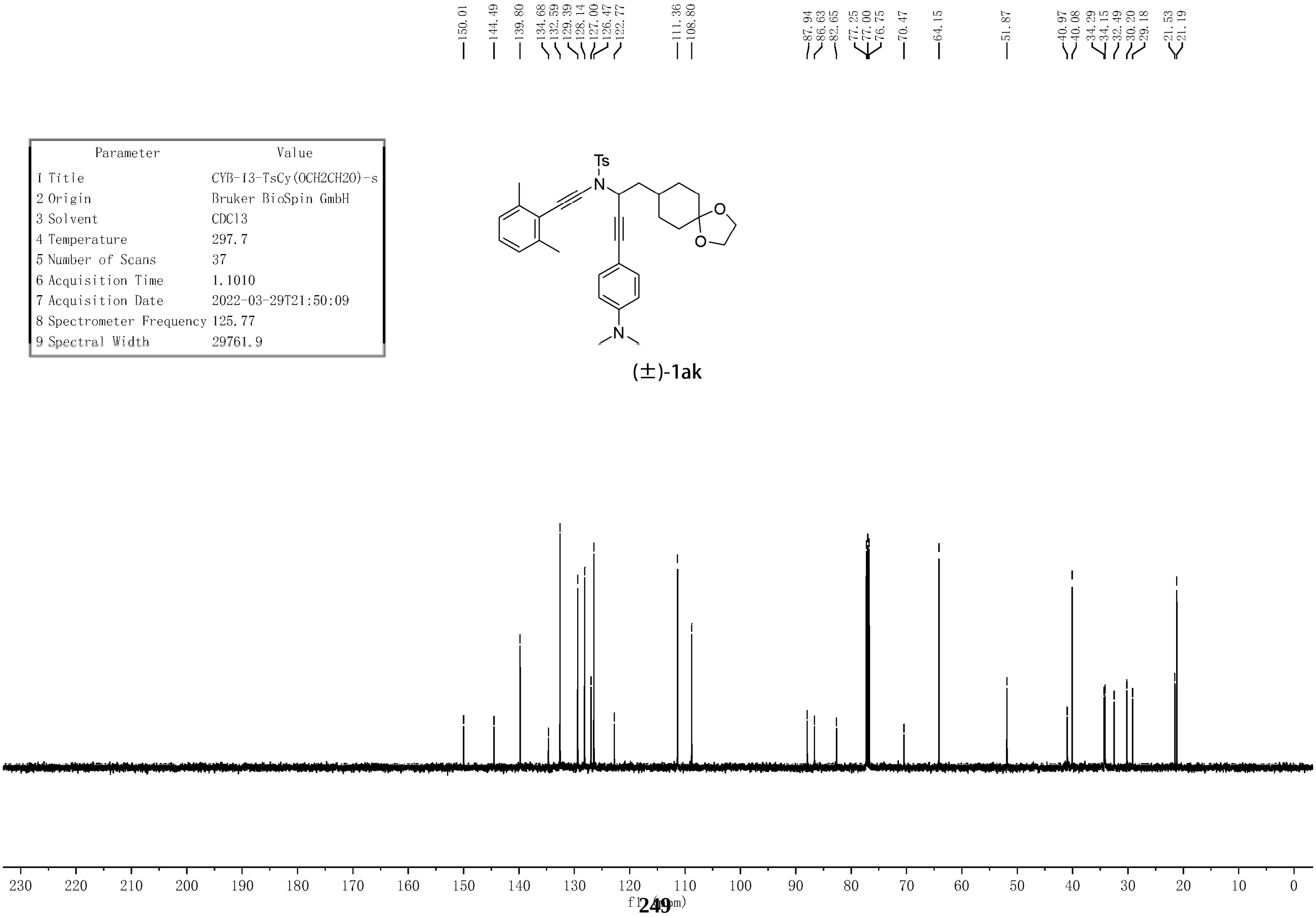

Supplementary Figure 149. ^{1}H and ^{13}C NMR spectra for (±)-1ak

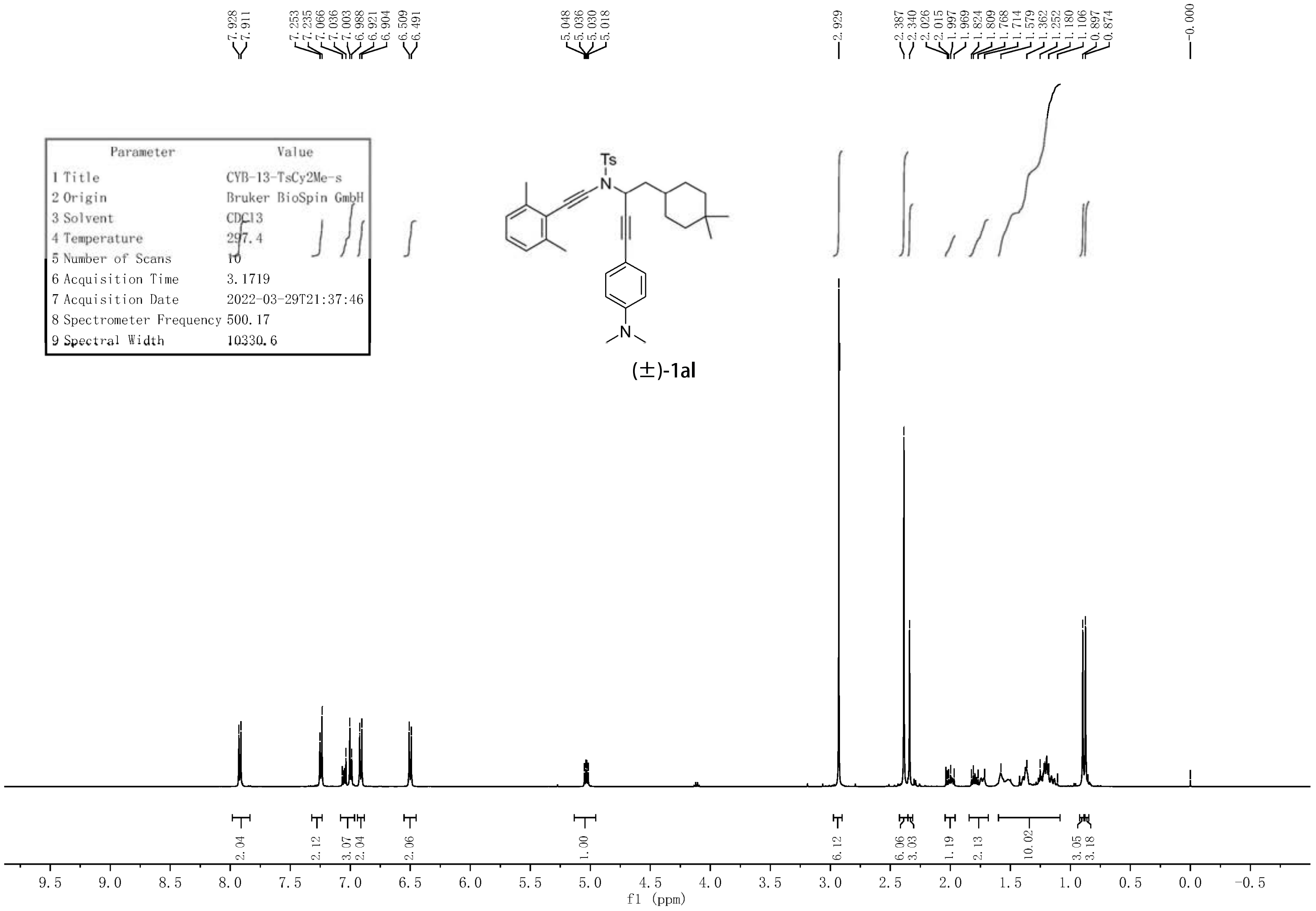

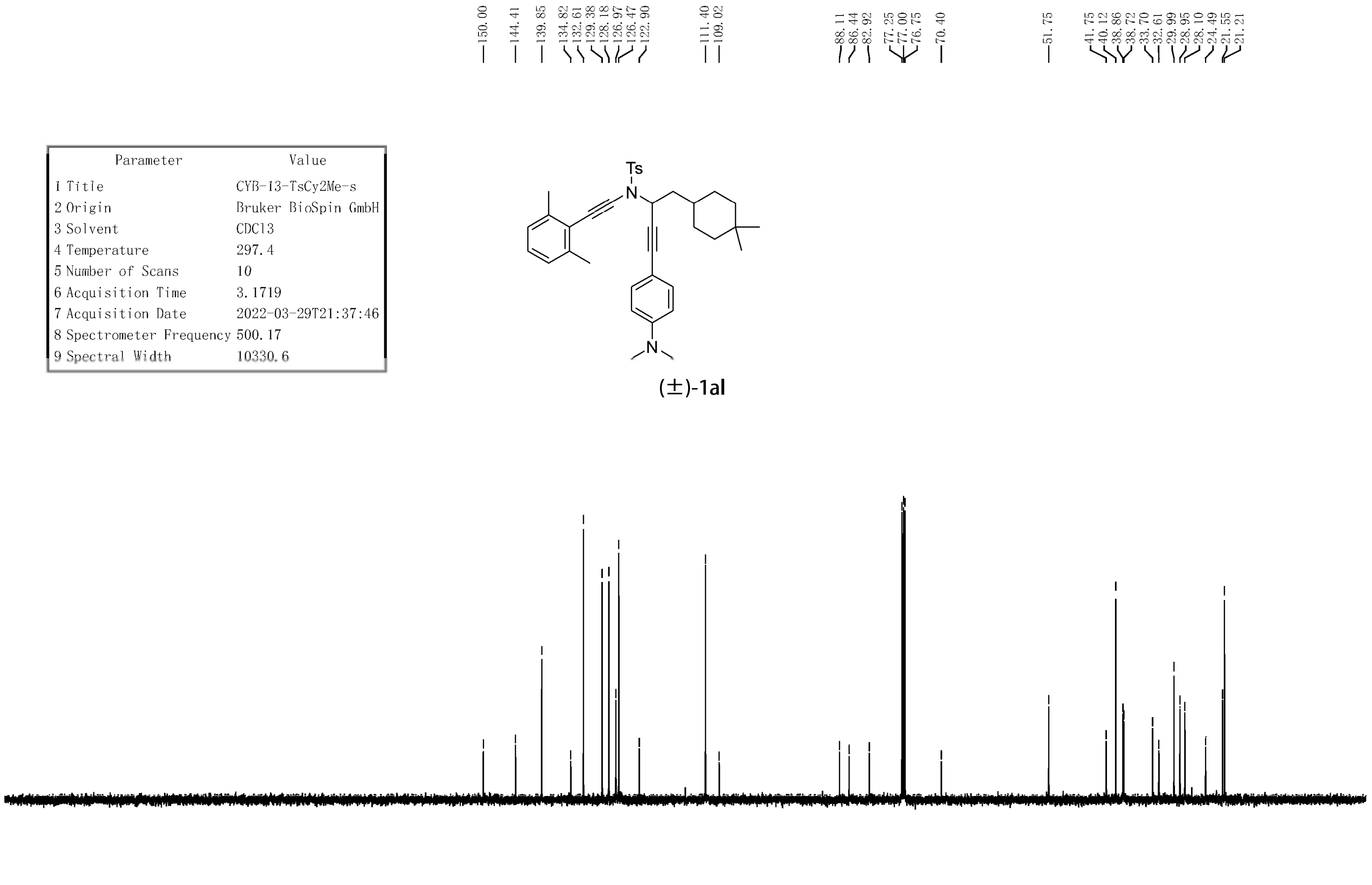

Supplementary Figure 150. ^{1}H and ^{13}C NMR spectra for (±)-1al

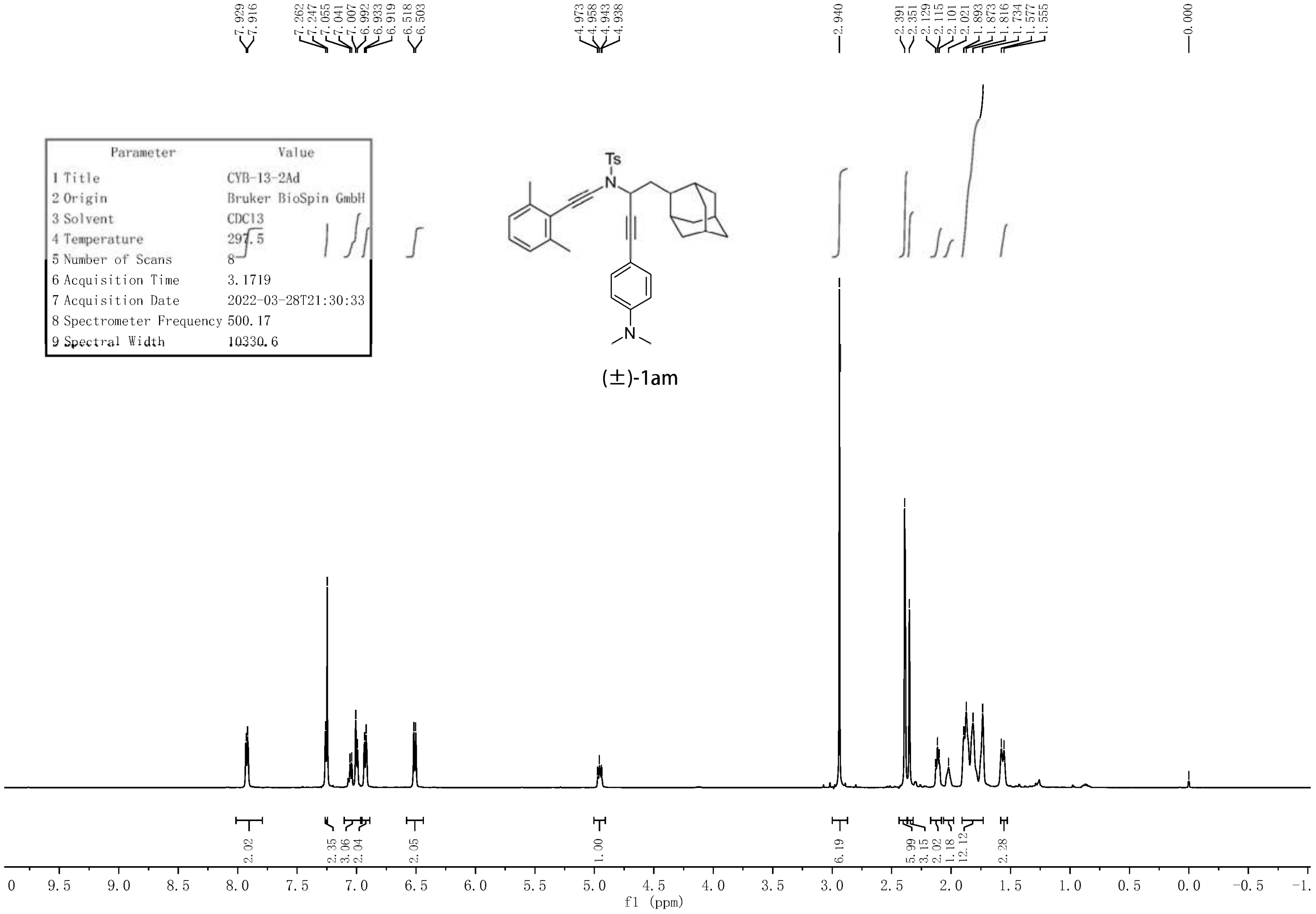

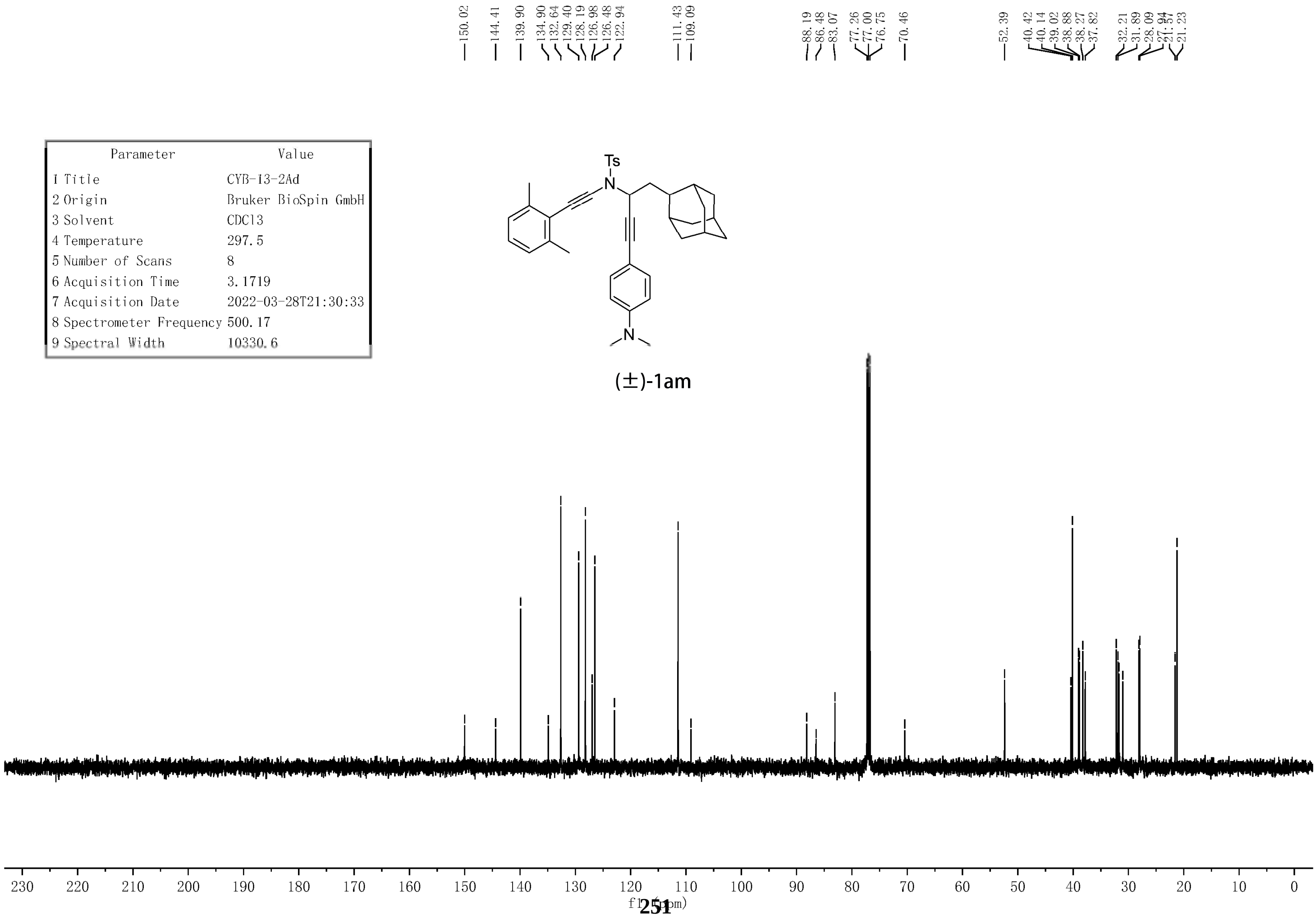

Supplementary Figure 151. ^{1}H and ^{13}C NMR spectra for (±)-1am

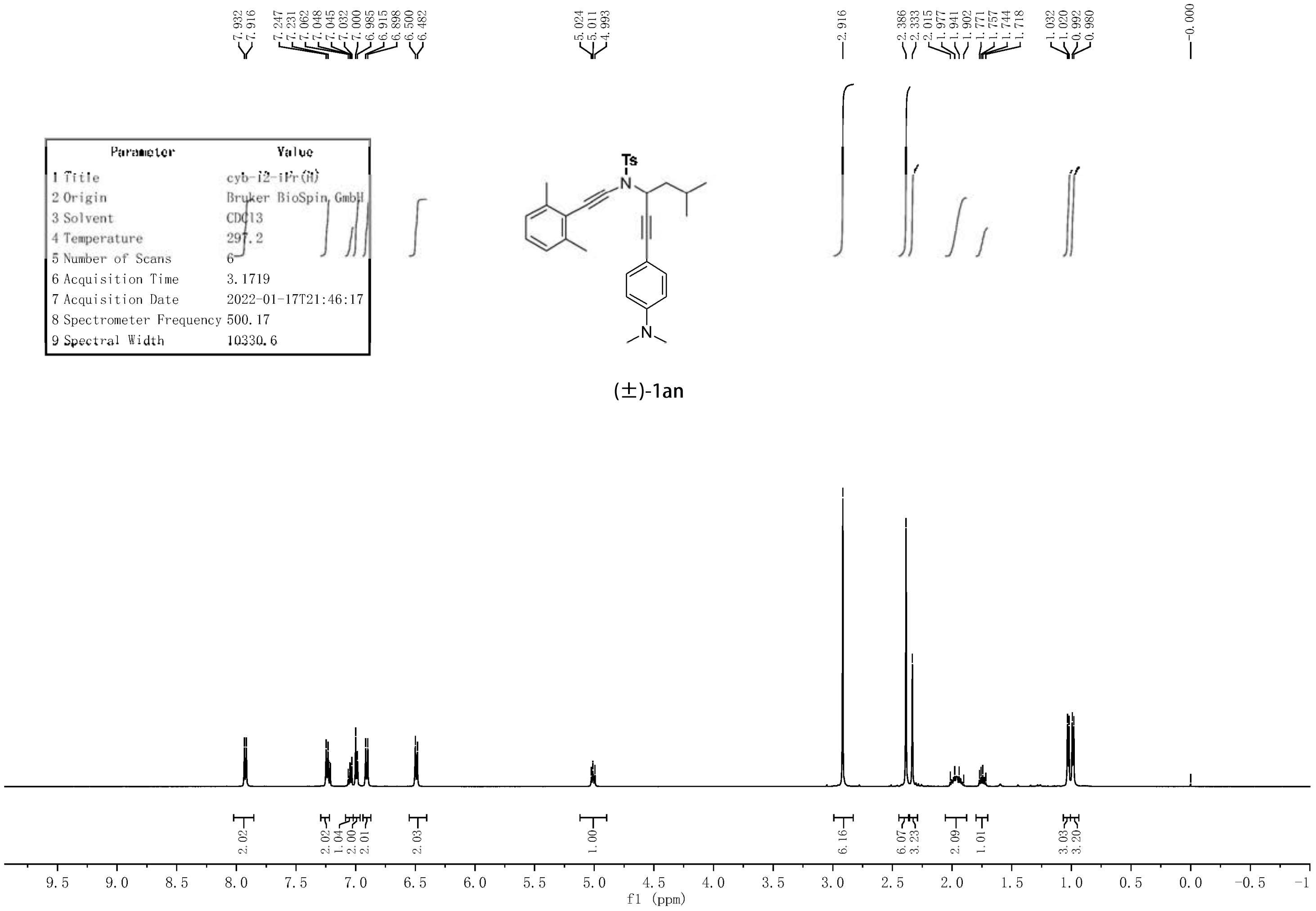

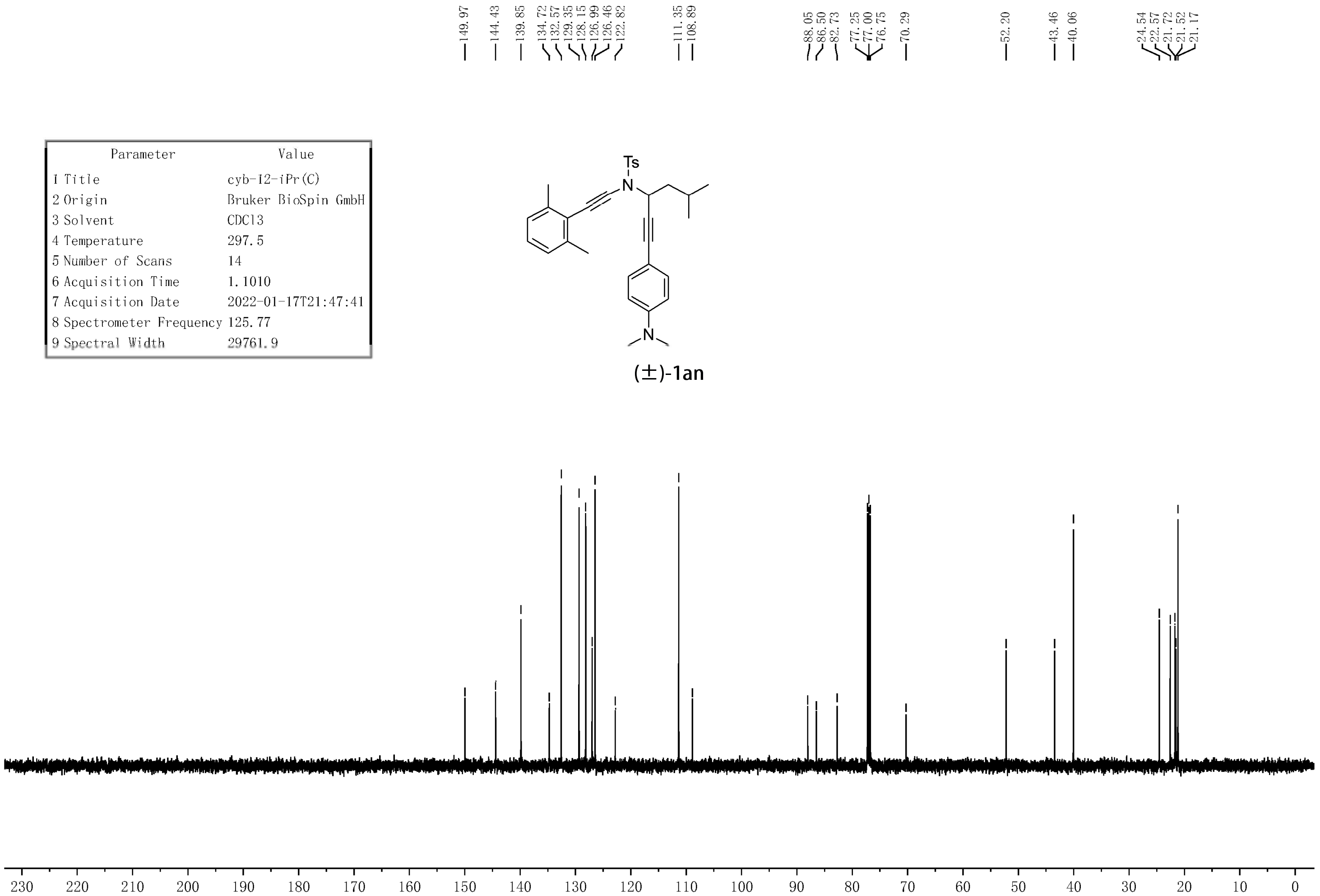

Supplementary Figure 152. ^{1}H and ^{13}C NMR spectra for (±)-1an

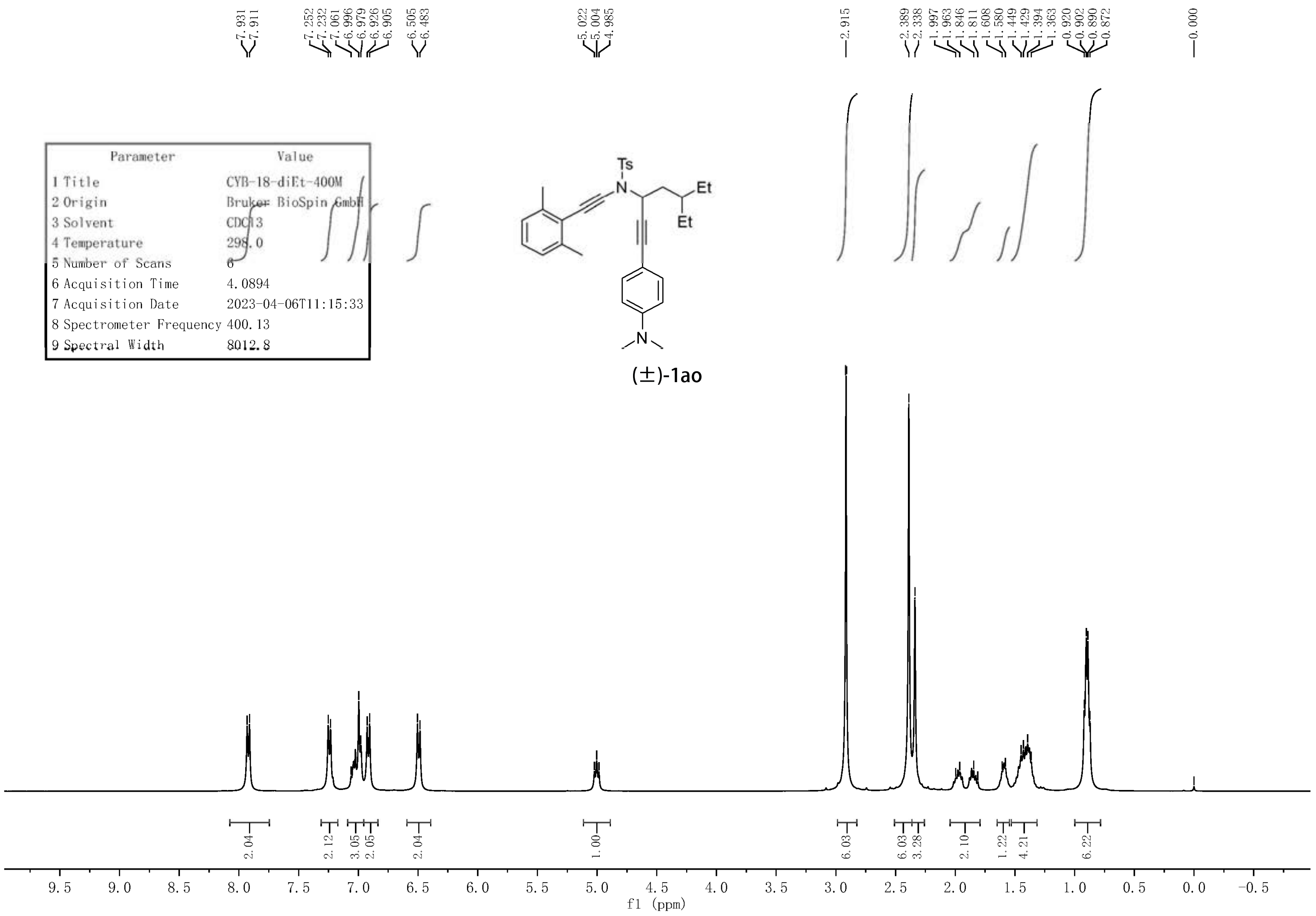

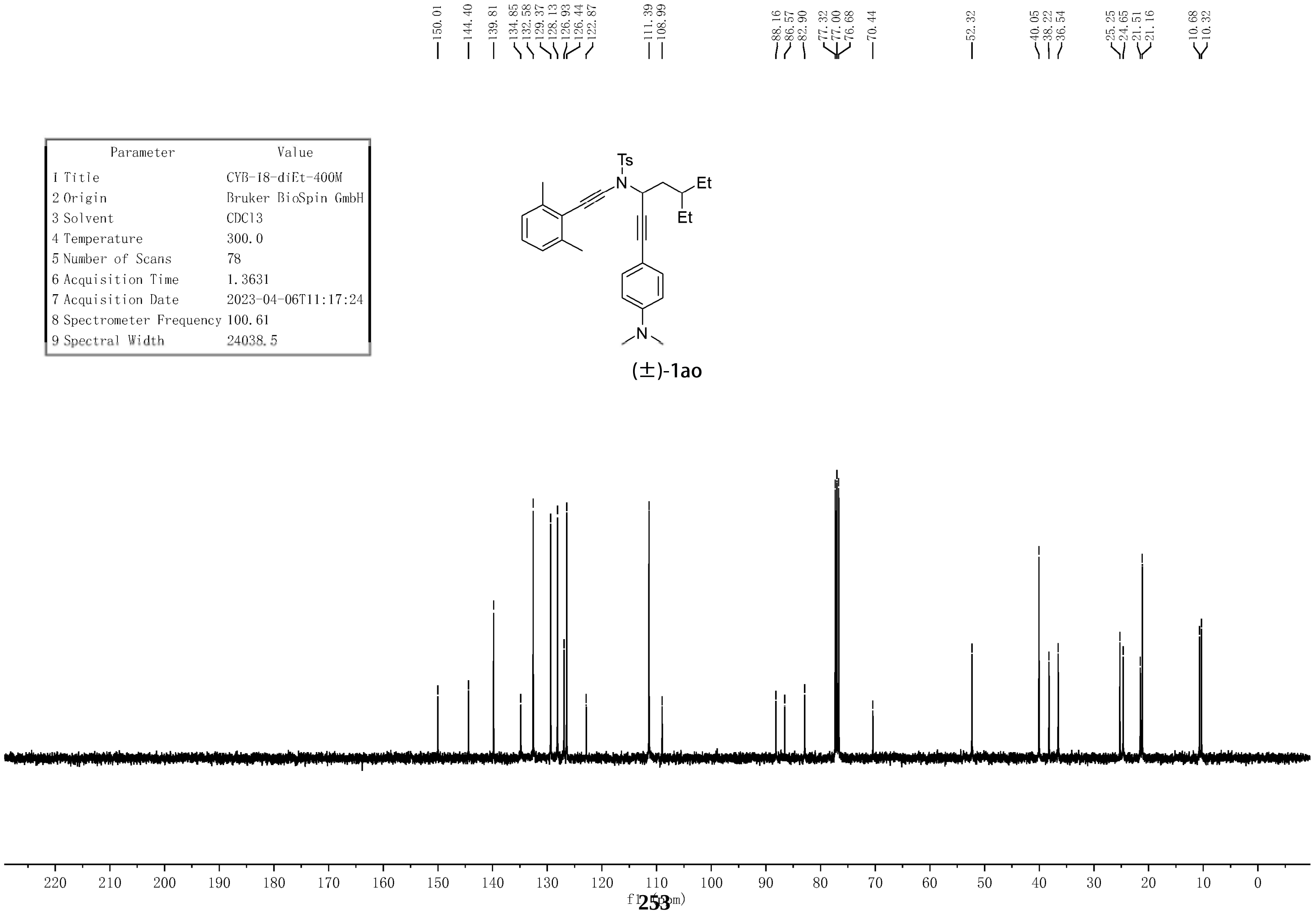

Supplementary Figure 153. ^{1}H and ^{13}C NMR spectra for (±)-1ao

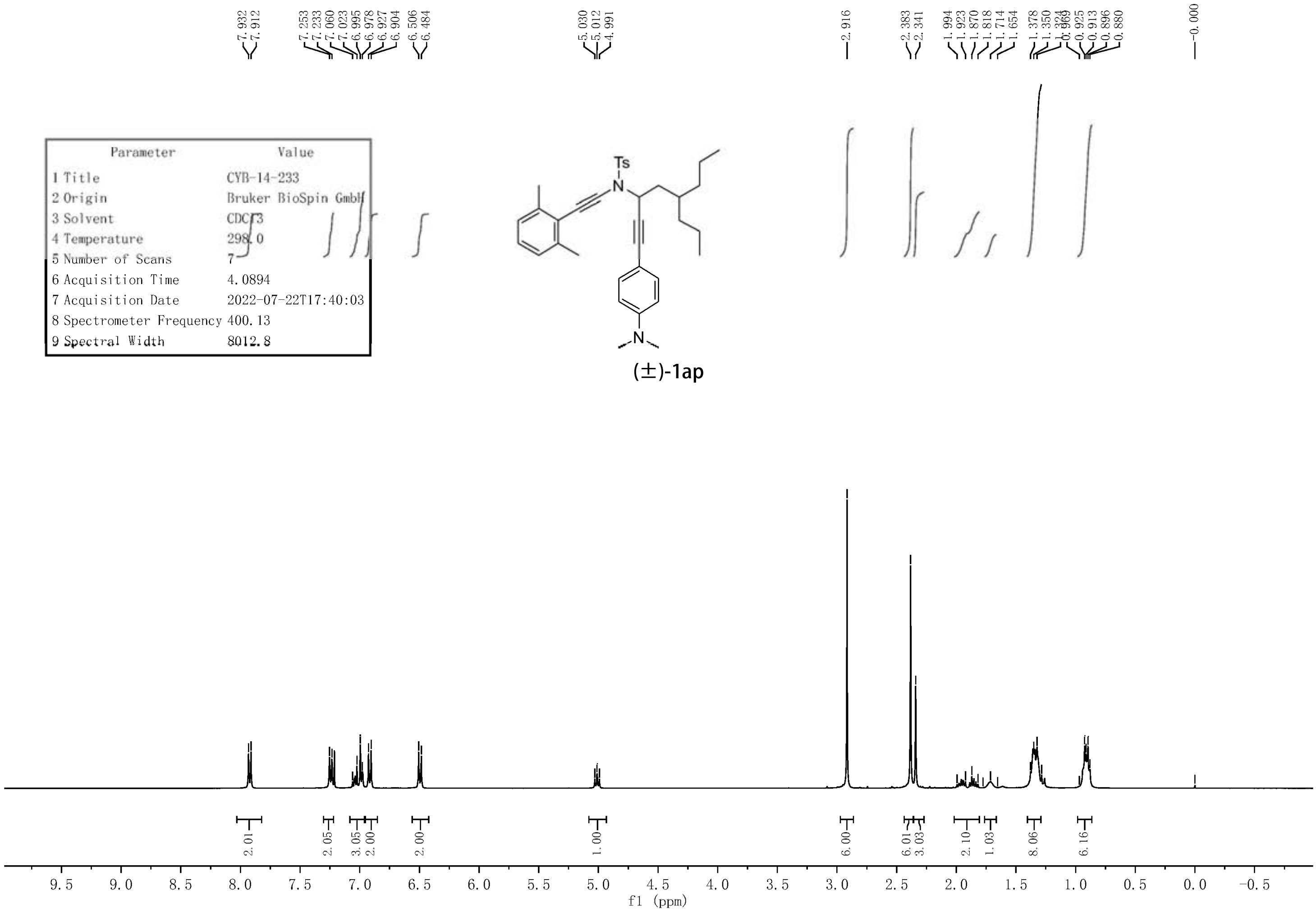

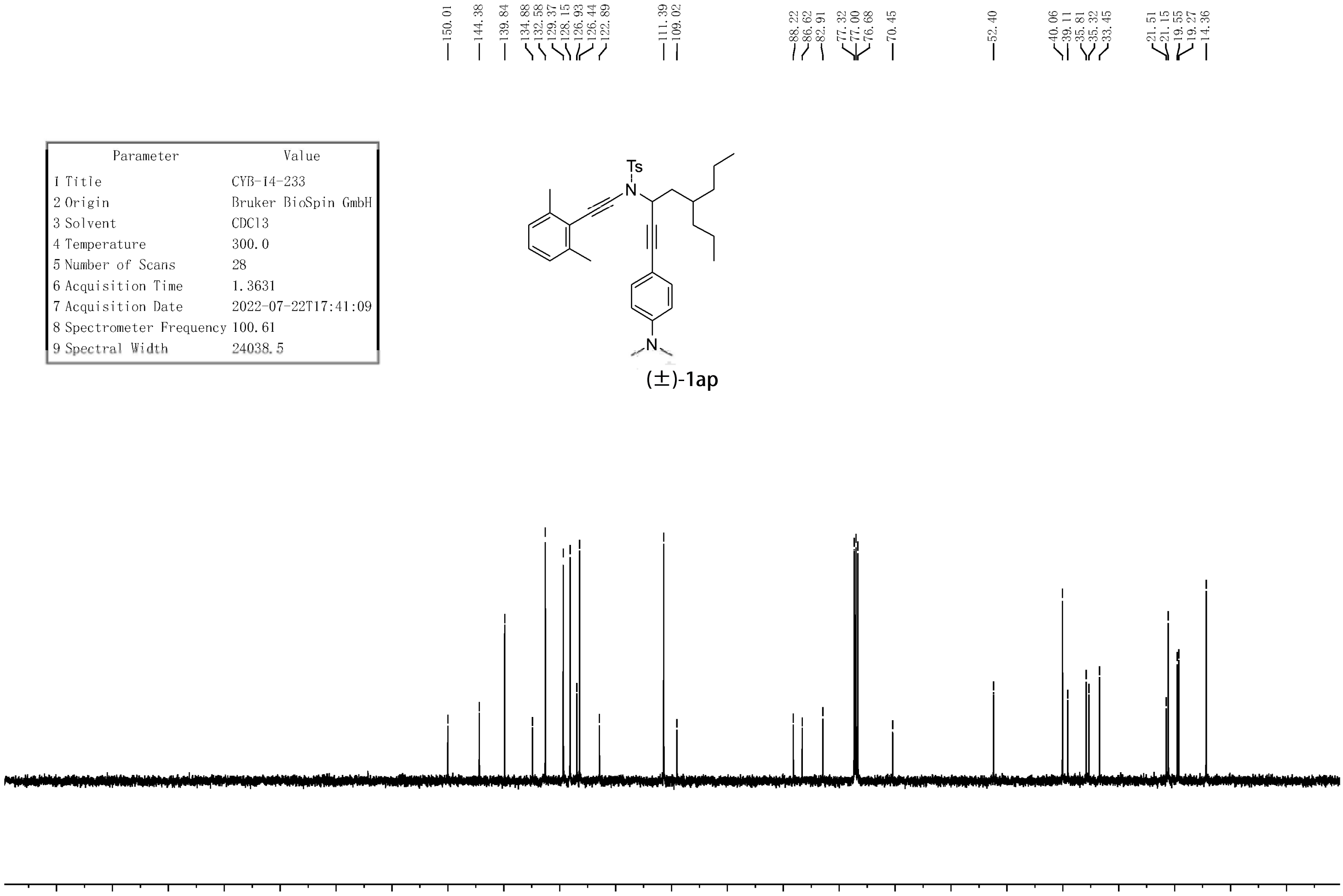

Supplementary Figure 154. ^{1}H and ^{13}C NMR spectra for (±)-1ap

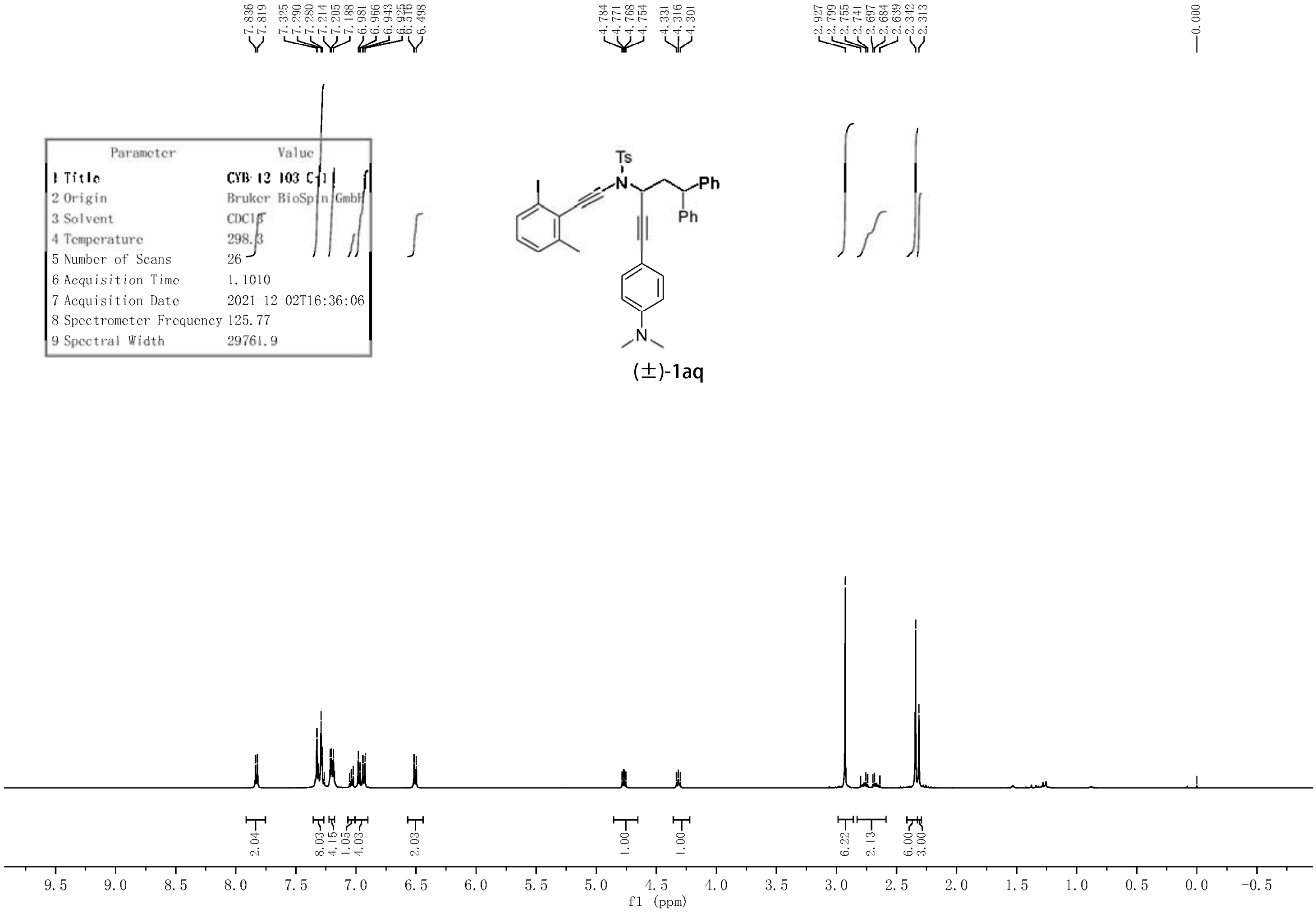

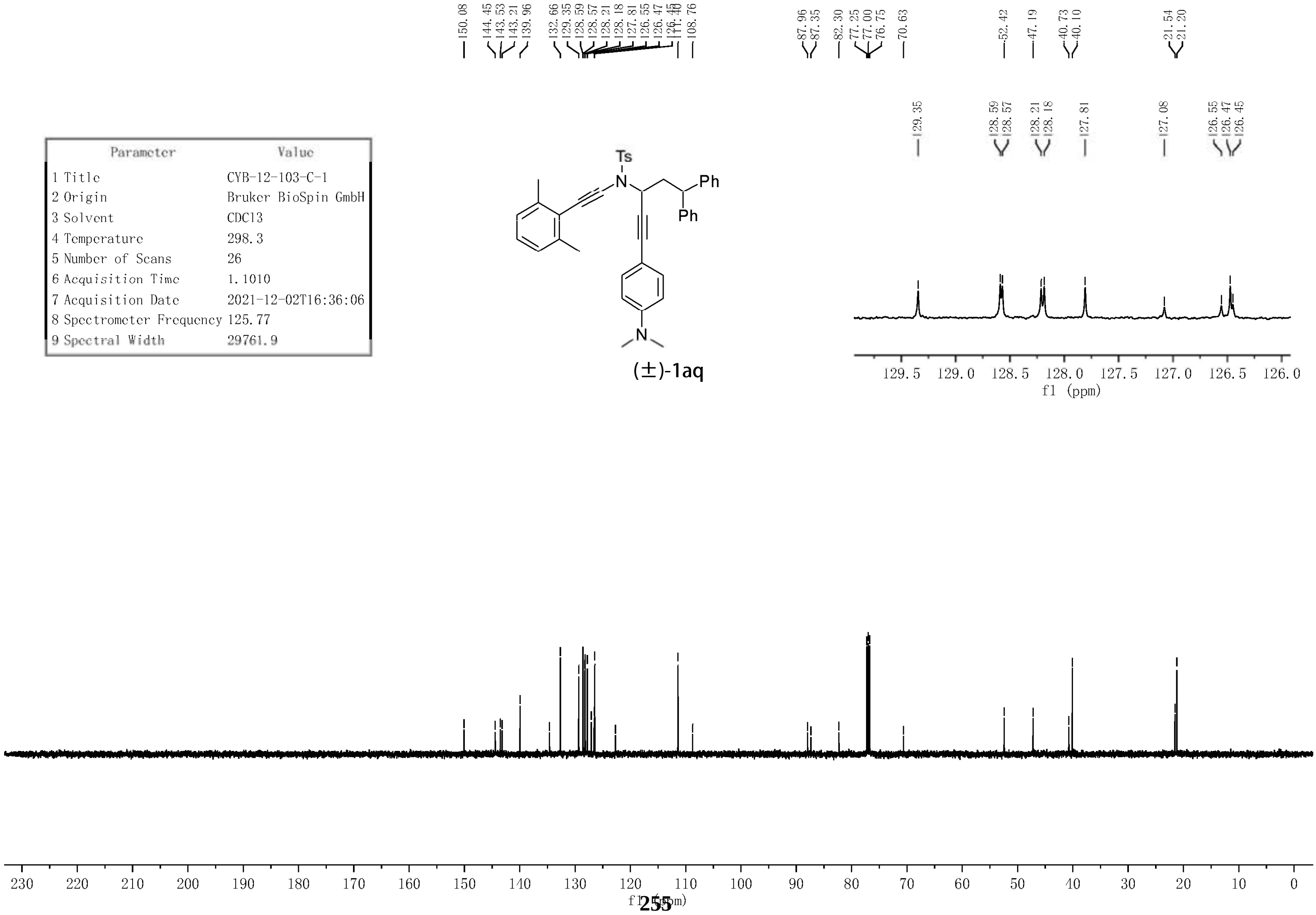

Supplementary Figure 155. ^{1}H and ^{13}C NMR spectra for (±)-1aq

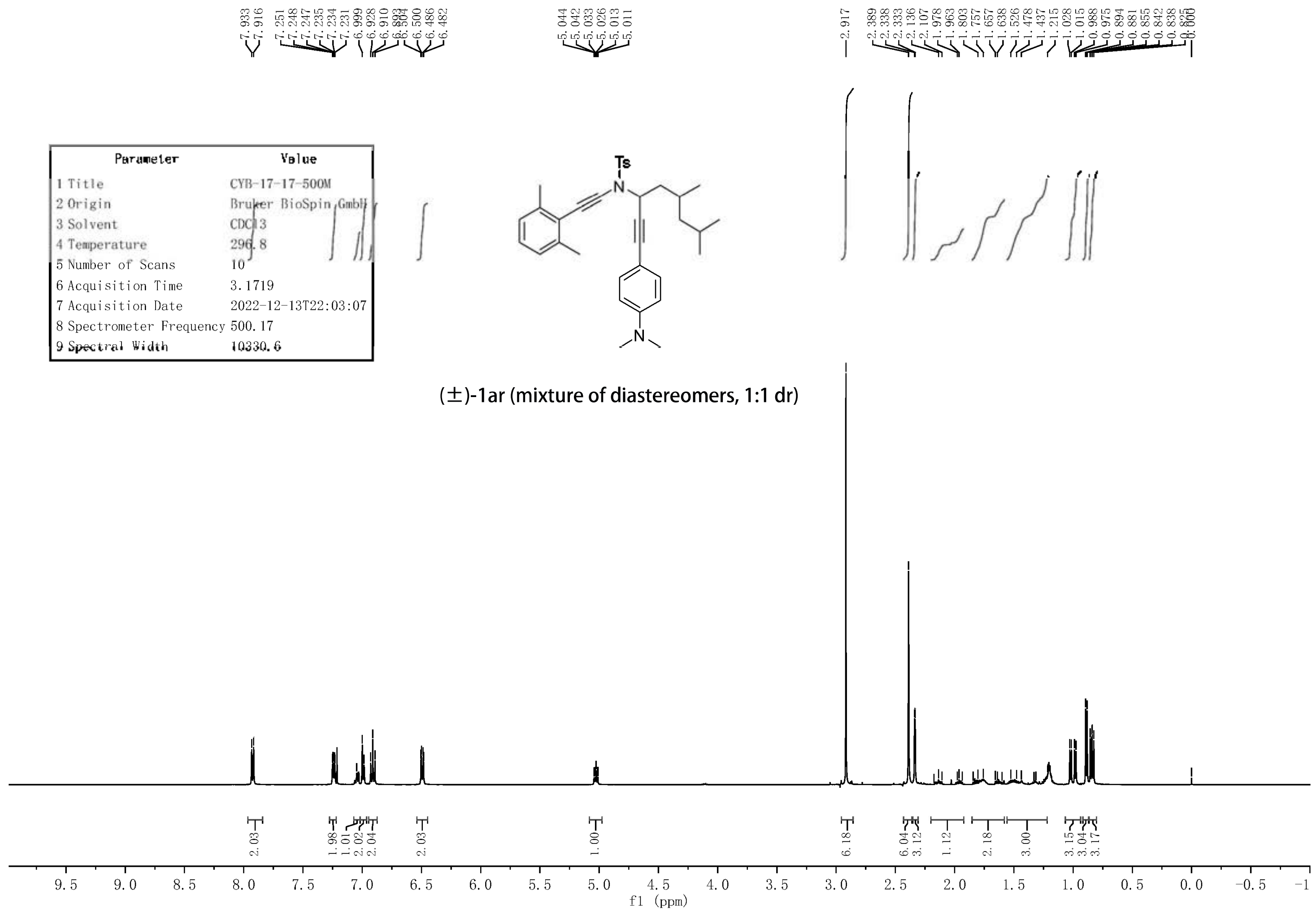

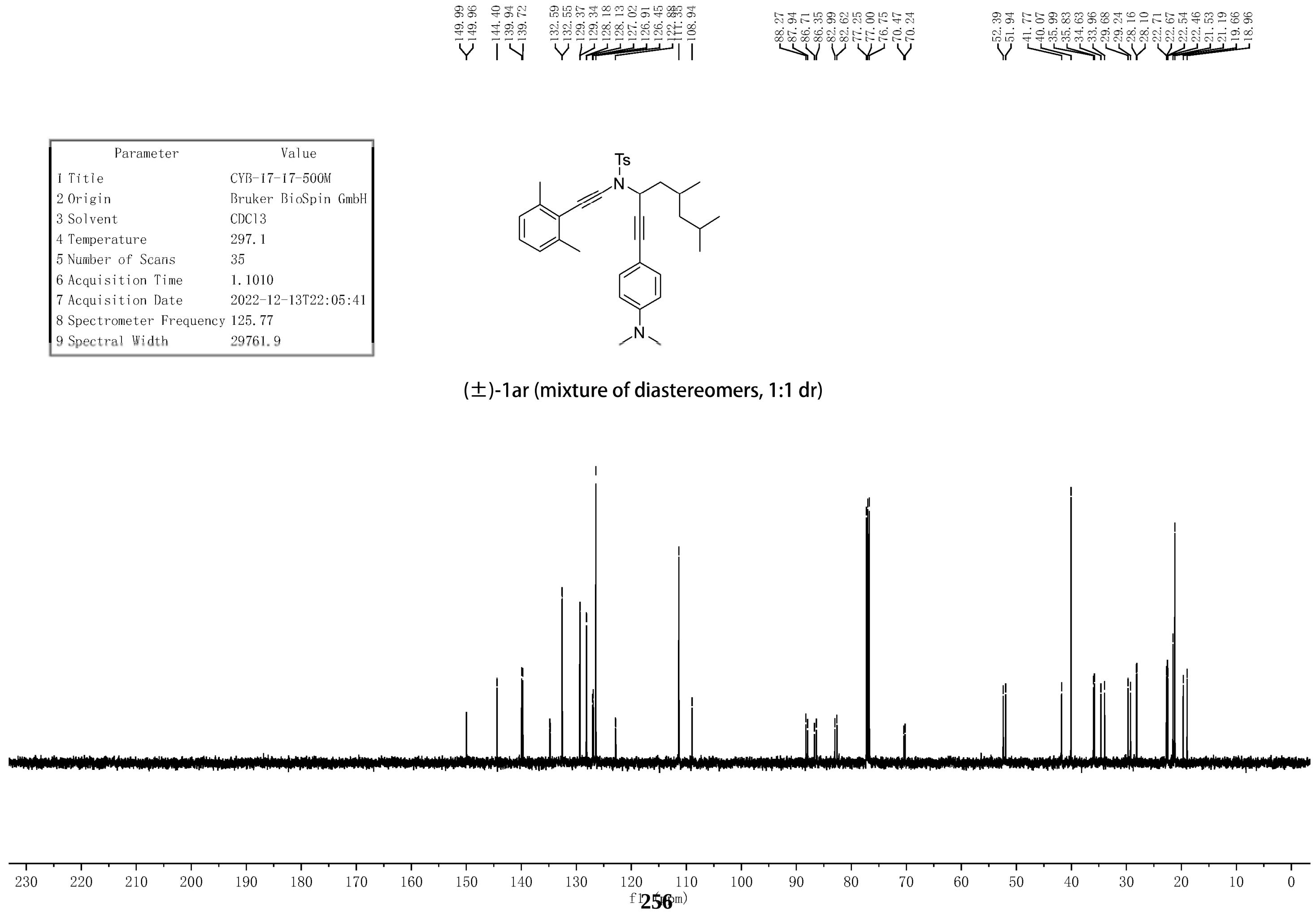

Supplementary Figure 156. ^{1}H and ^{13}C NMR spectra for (±)-1ar

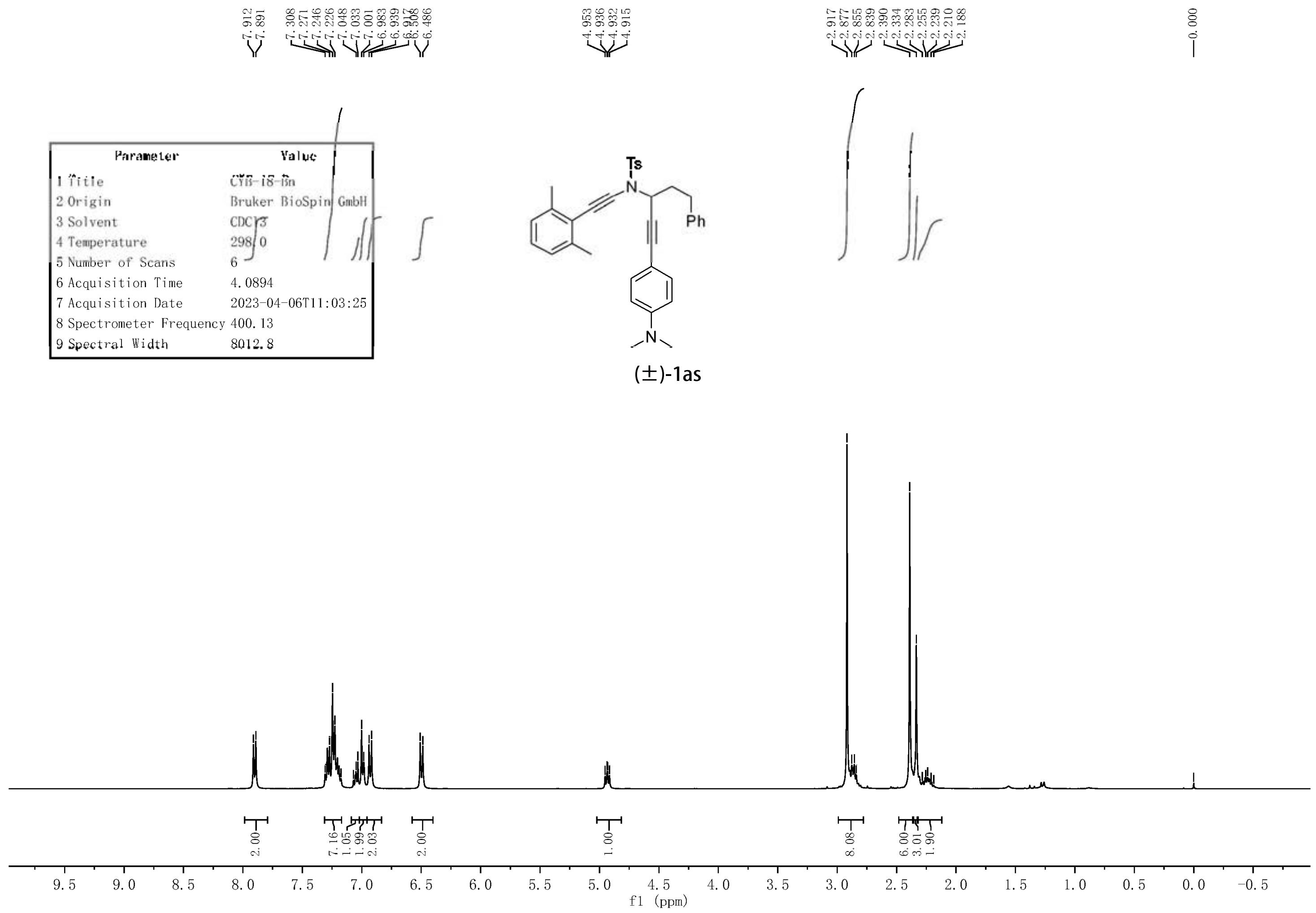

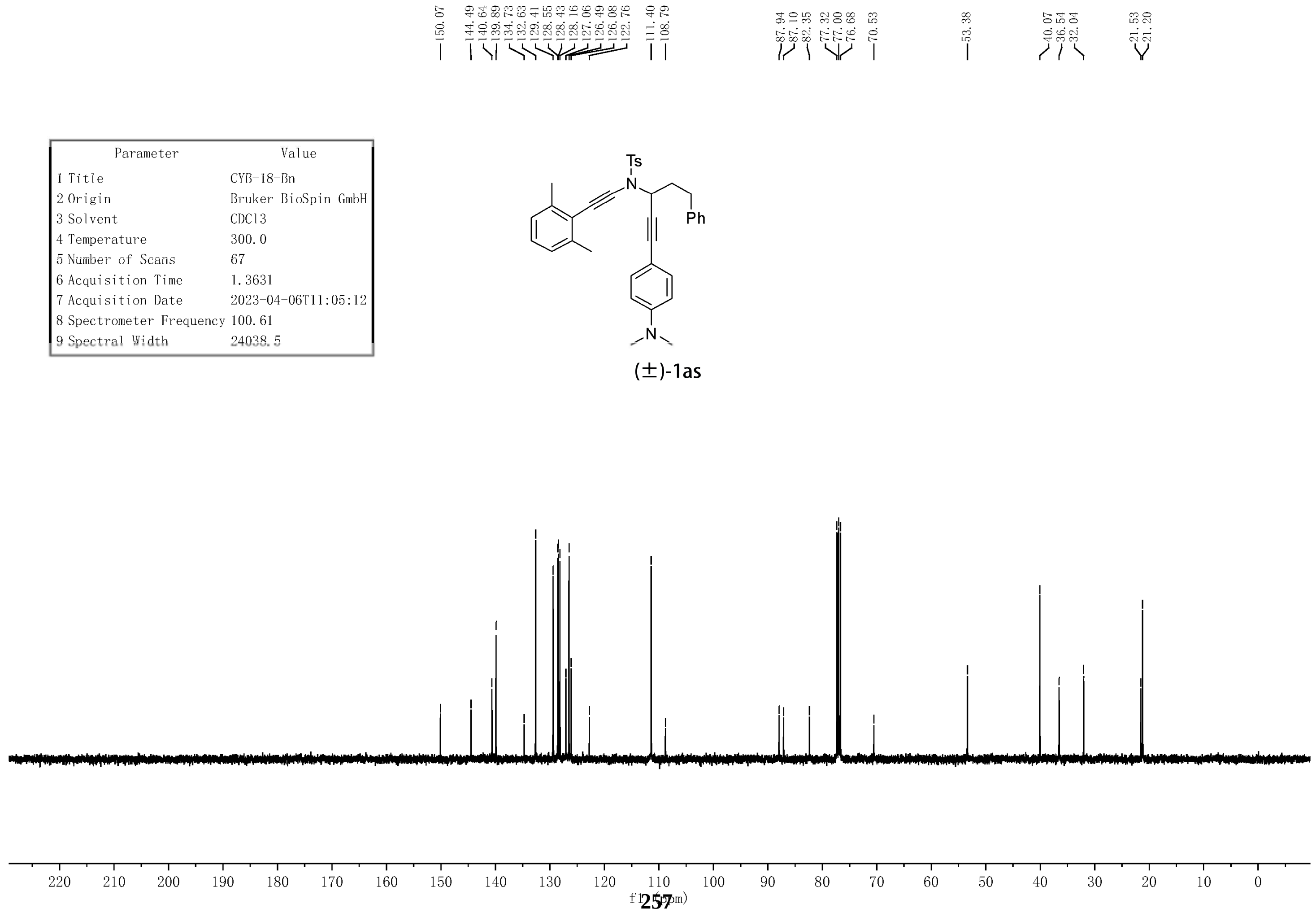

Supplementary Figure 157. ^{1}H and ^{13}C NMR spectra for (±)-1as

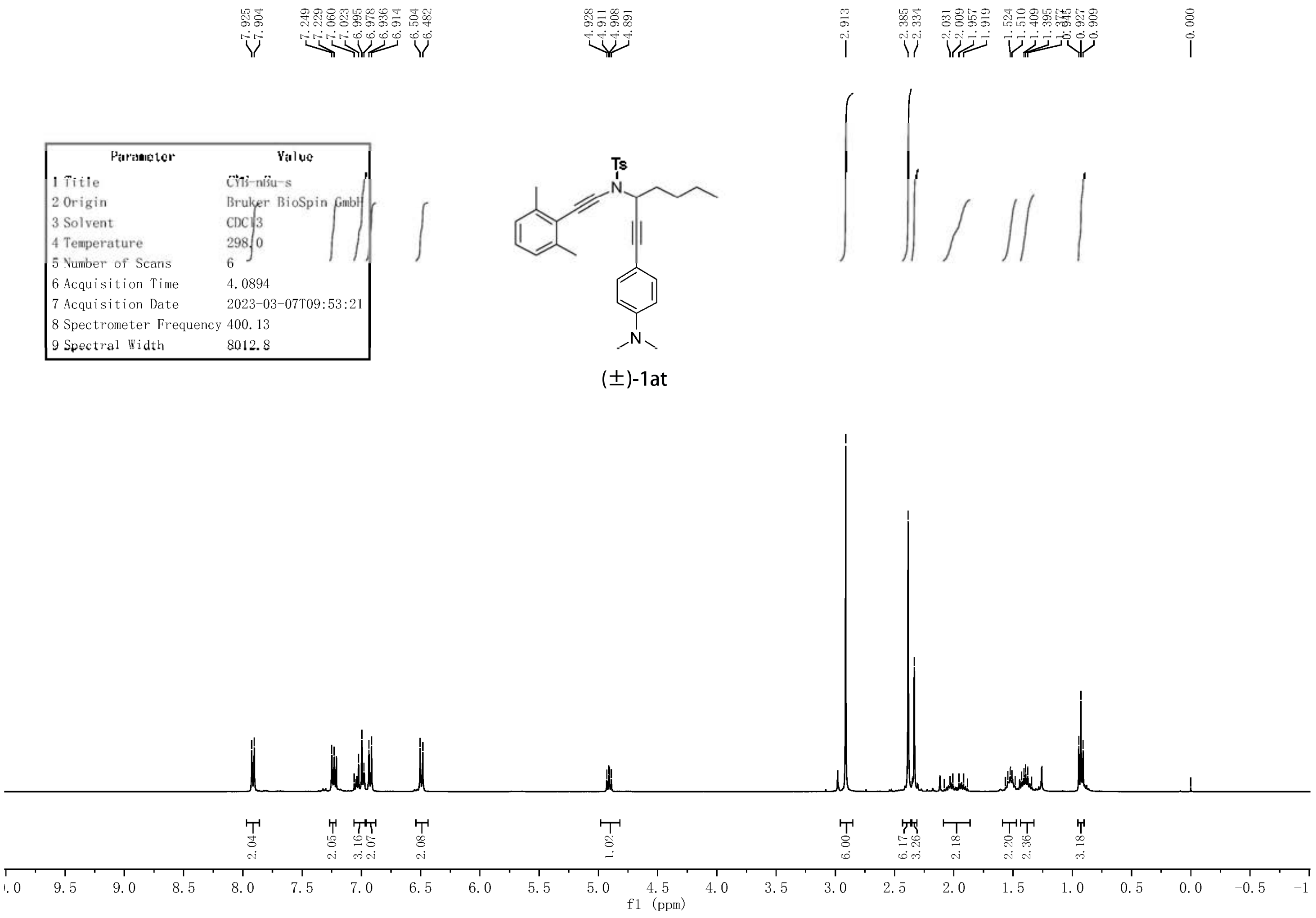

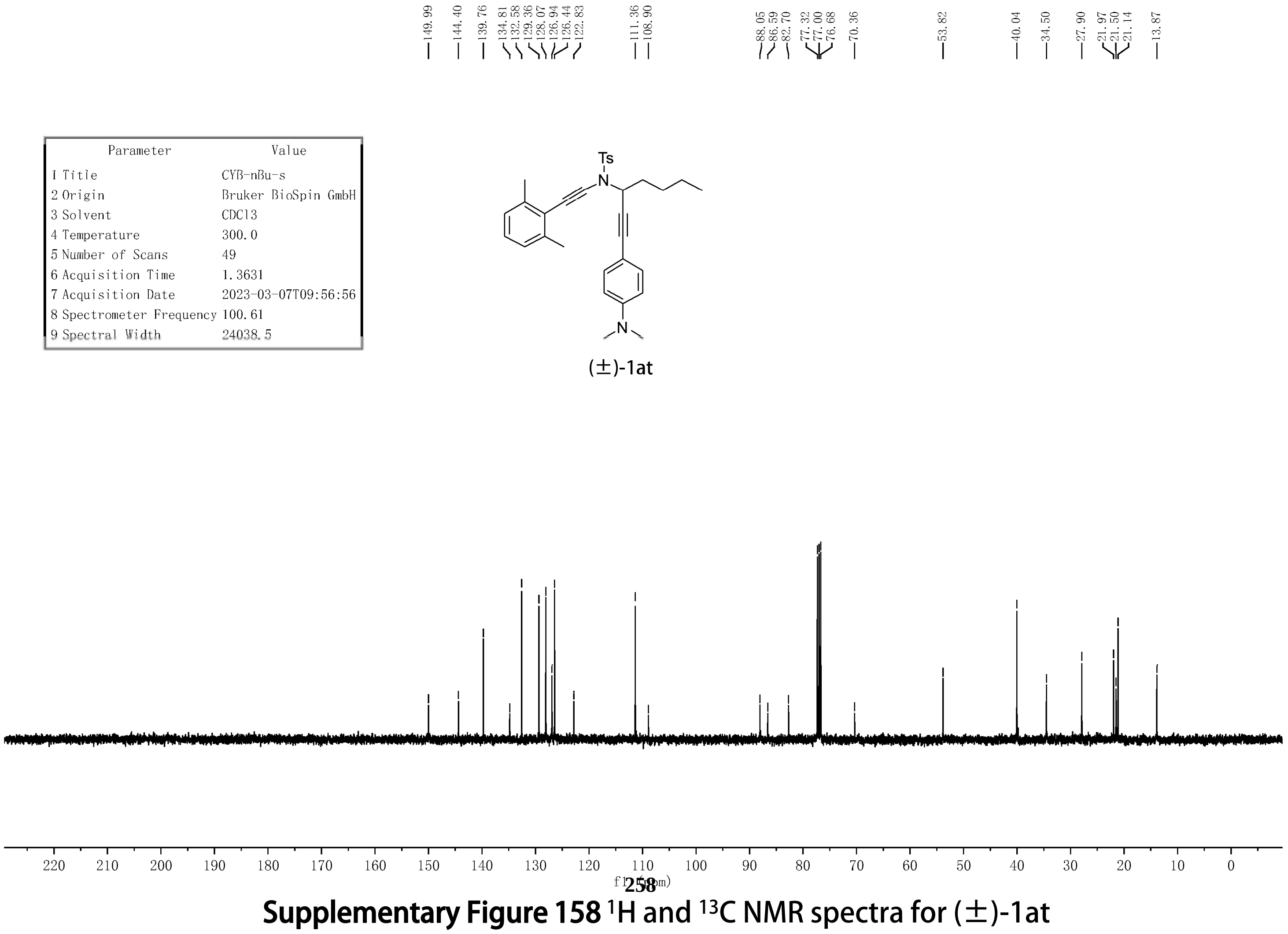

Supplementary Figure 158 ^1H and ^{13}C NMR spectra for (±)-1at

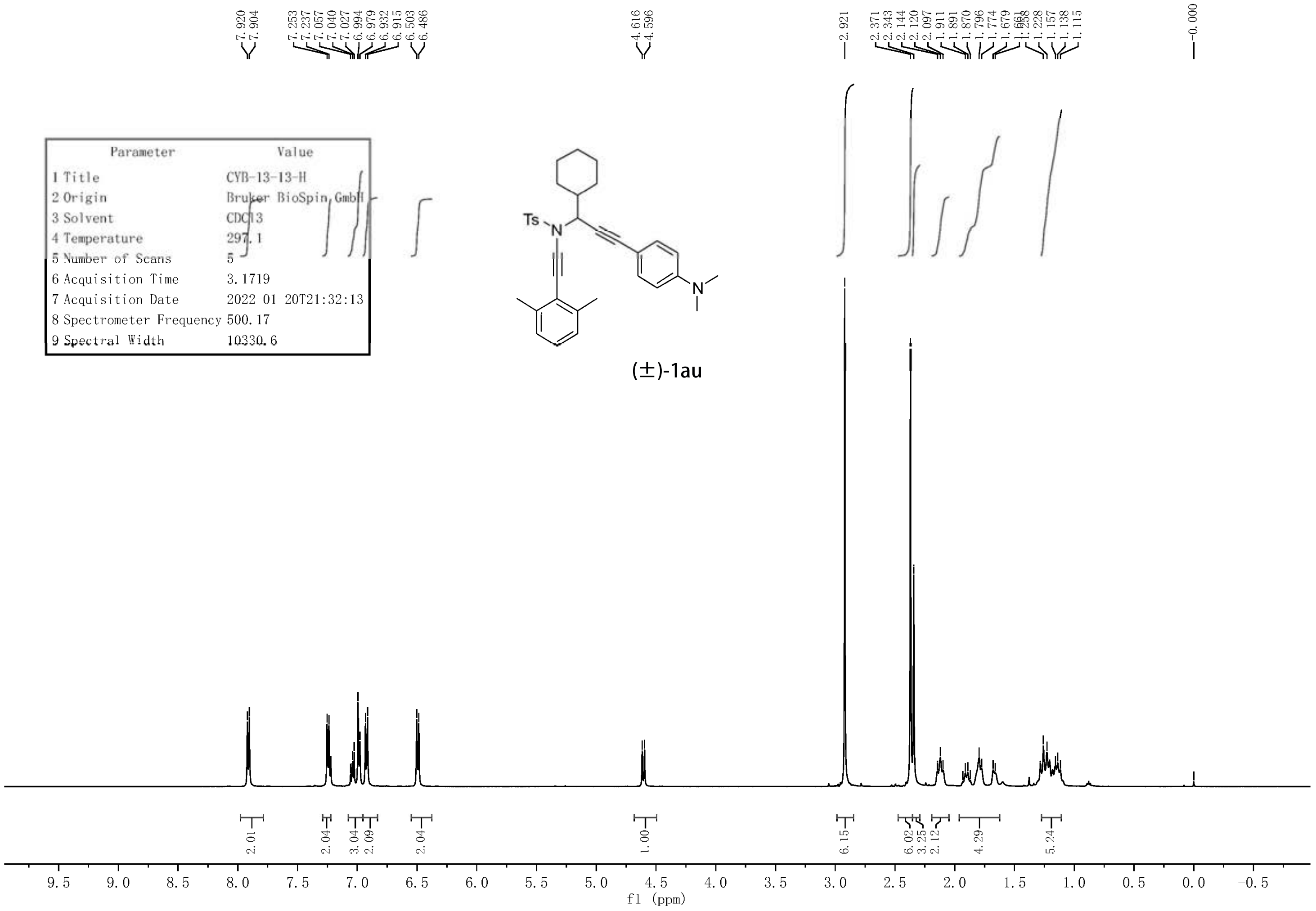

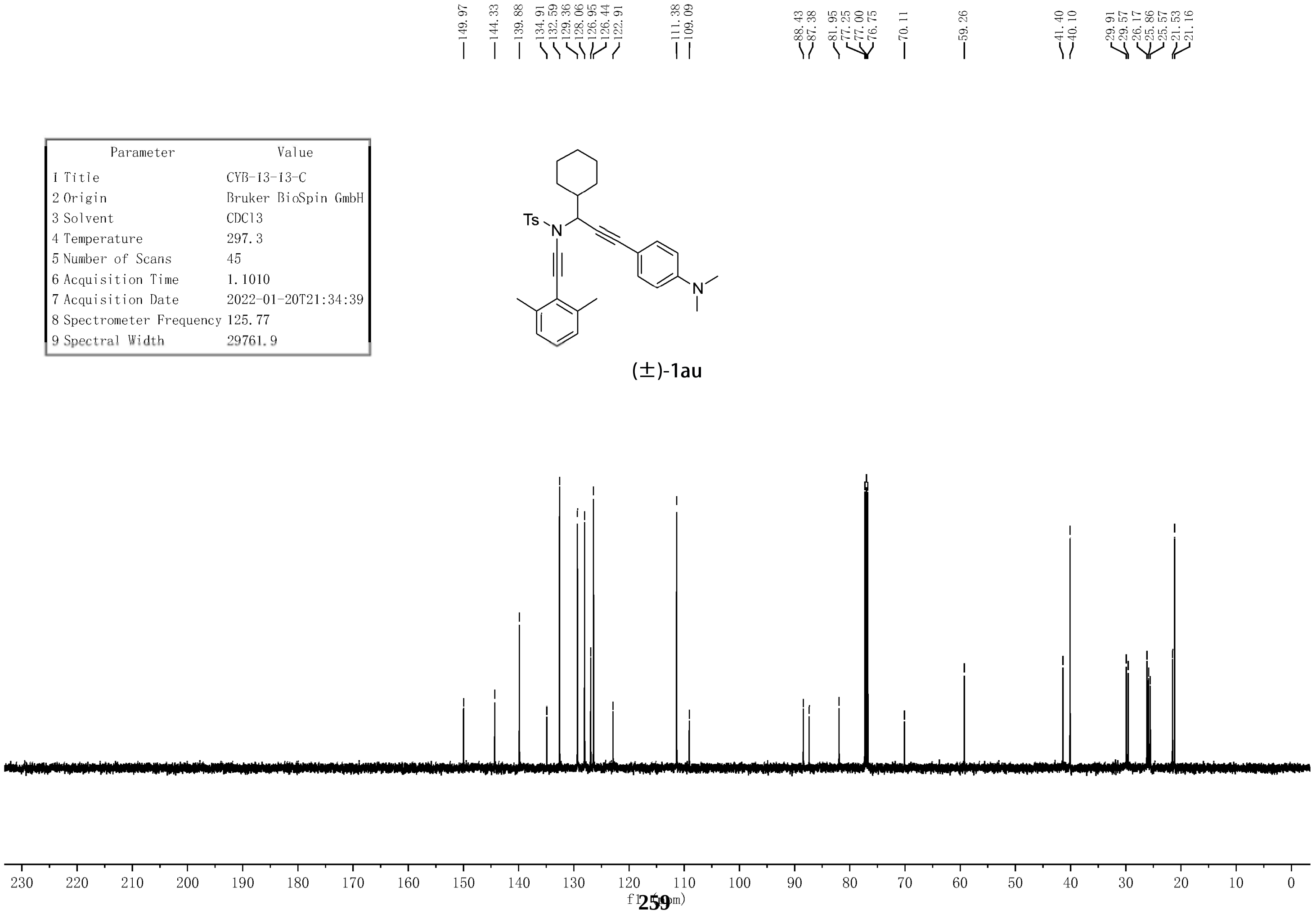

Supplementary Figure 159. ^{1}H and ^{13}C NMR spectra for (±)-1au

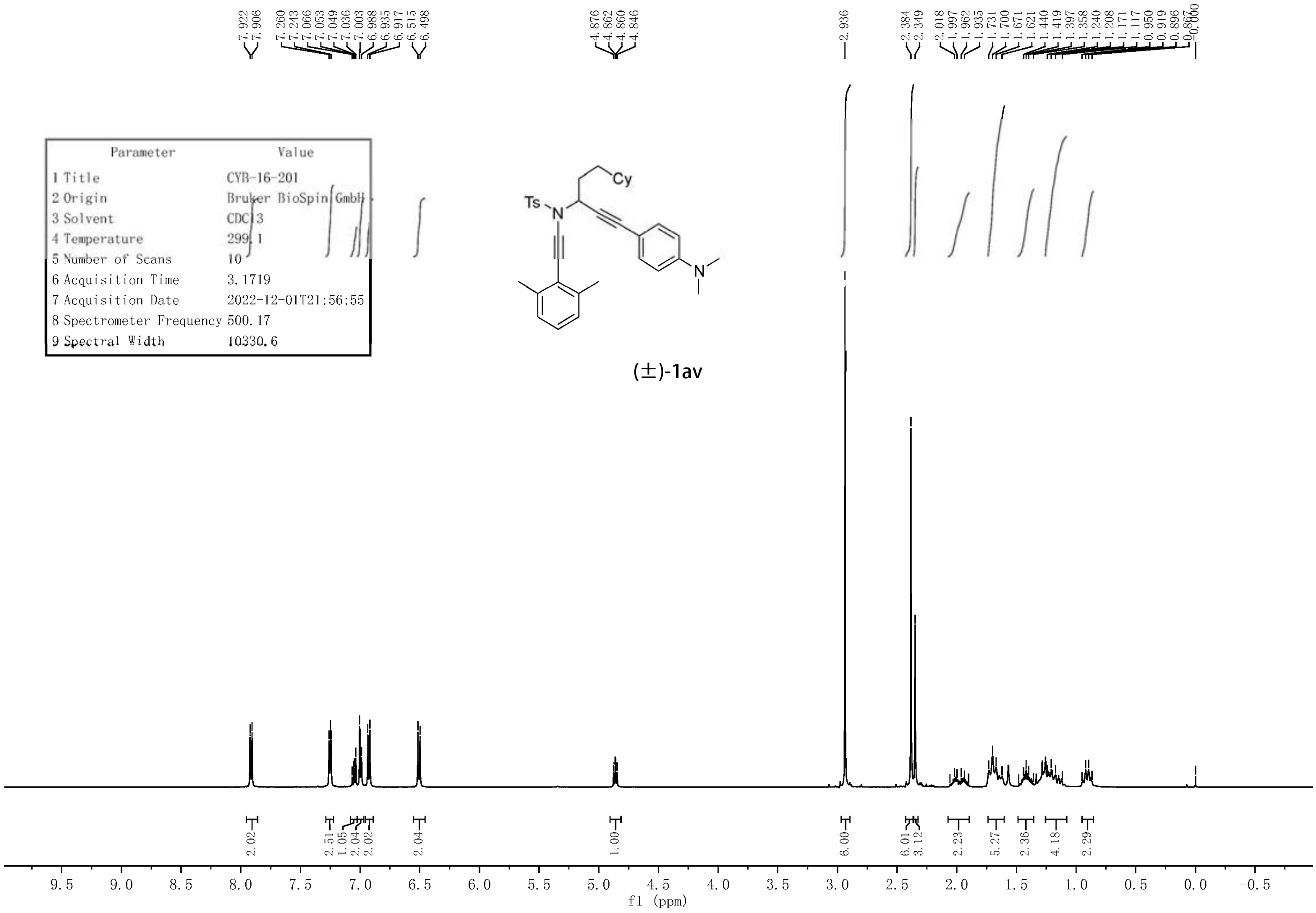

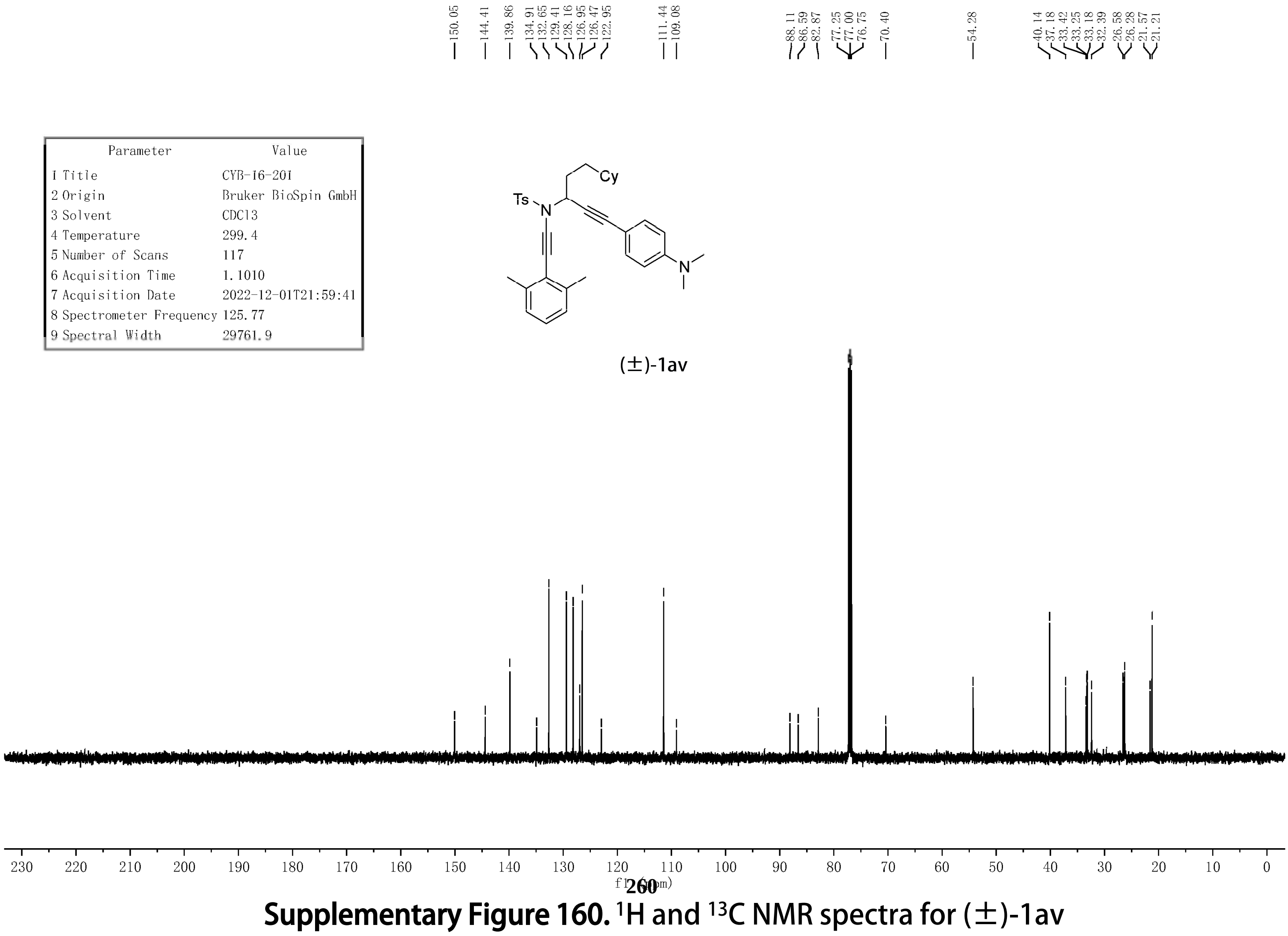

Supplementary Figure 160. ^{1}H and ^{13}C NMR spectra for (±)-1av

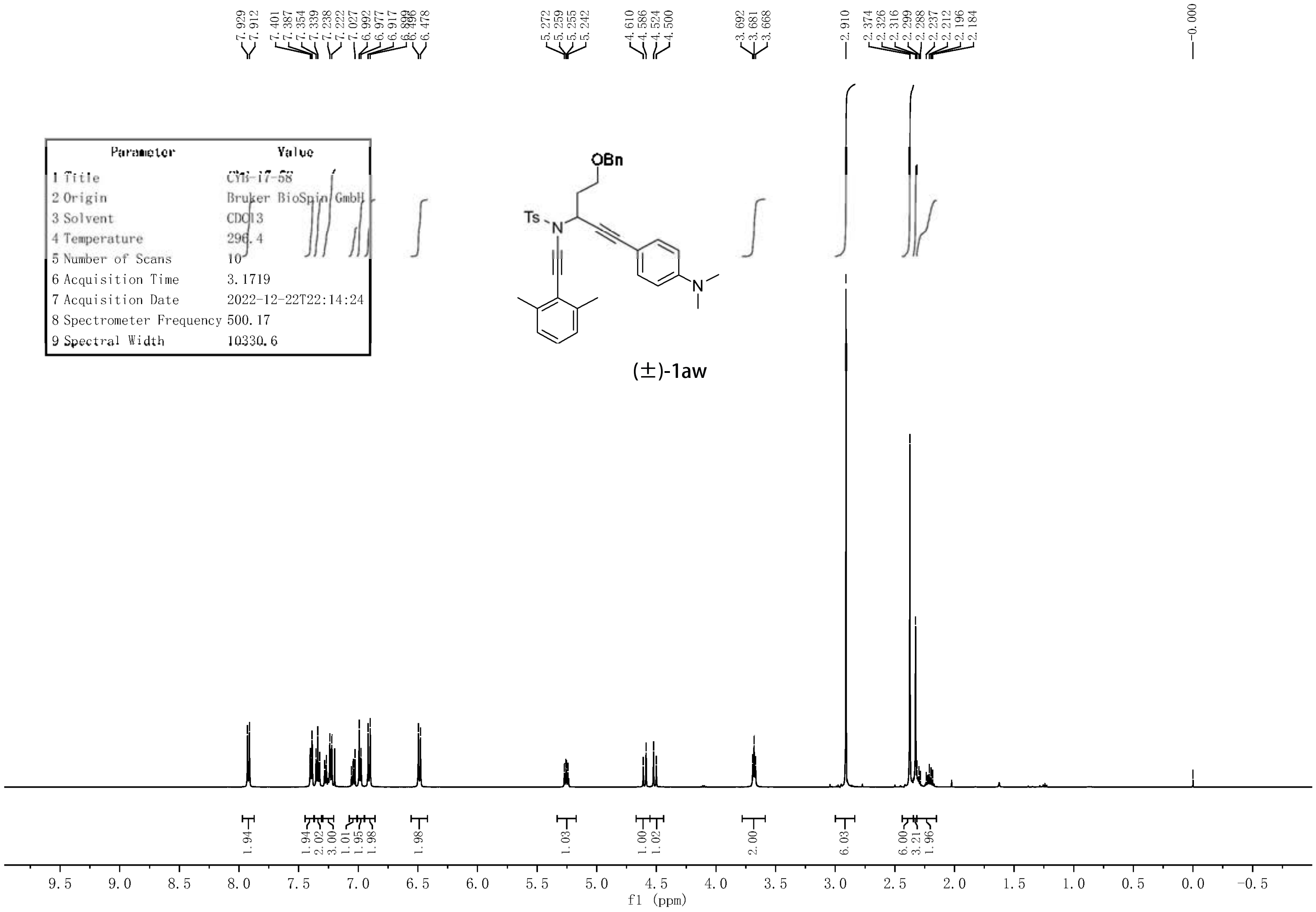

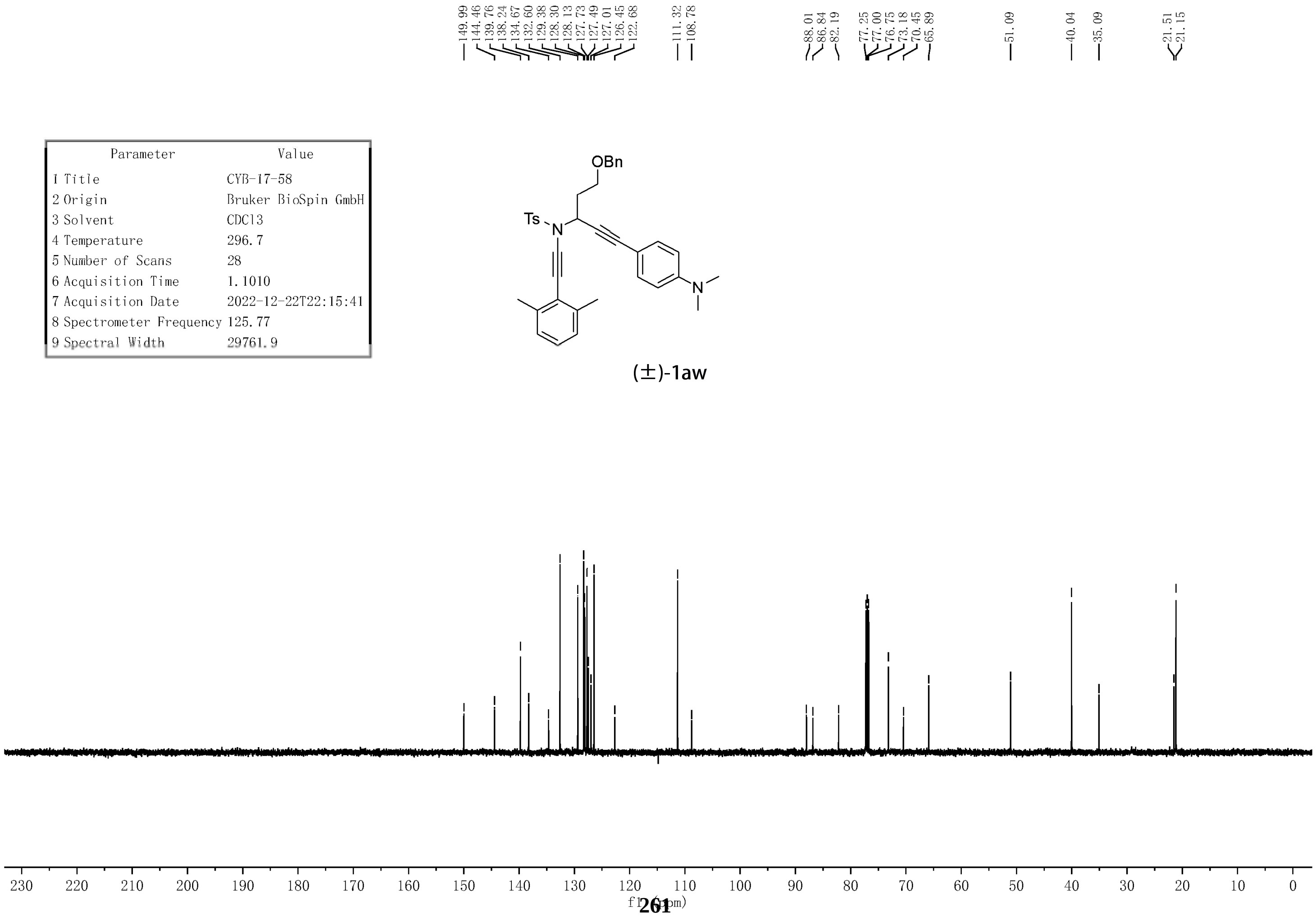

Supplementary Figure 161. ^{1}H and ^{13}C NMR spectra for (±)-1aw

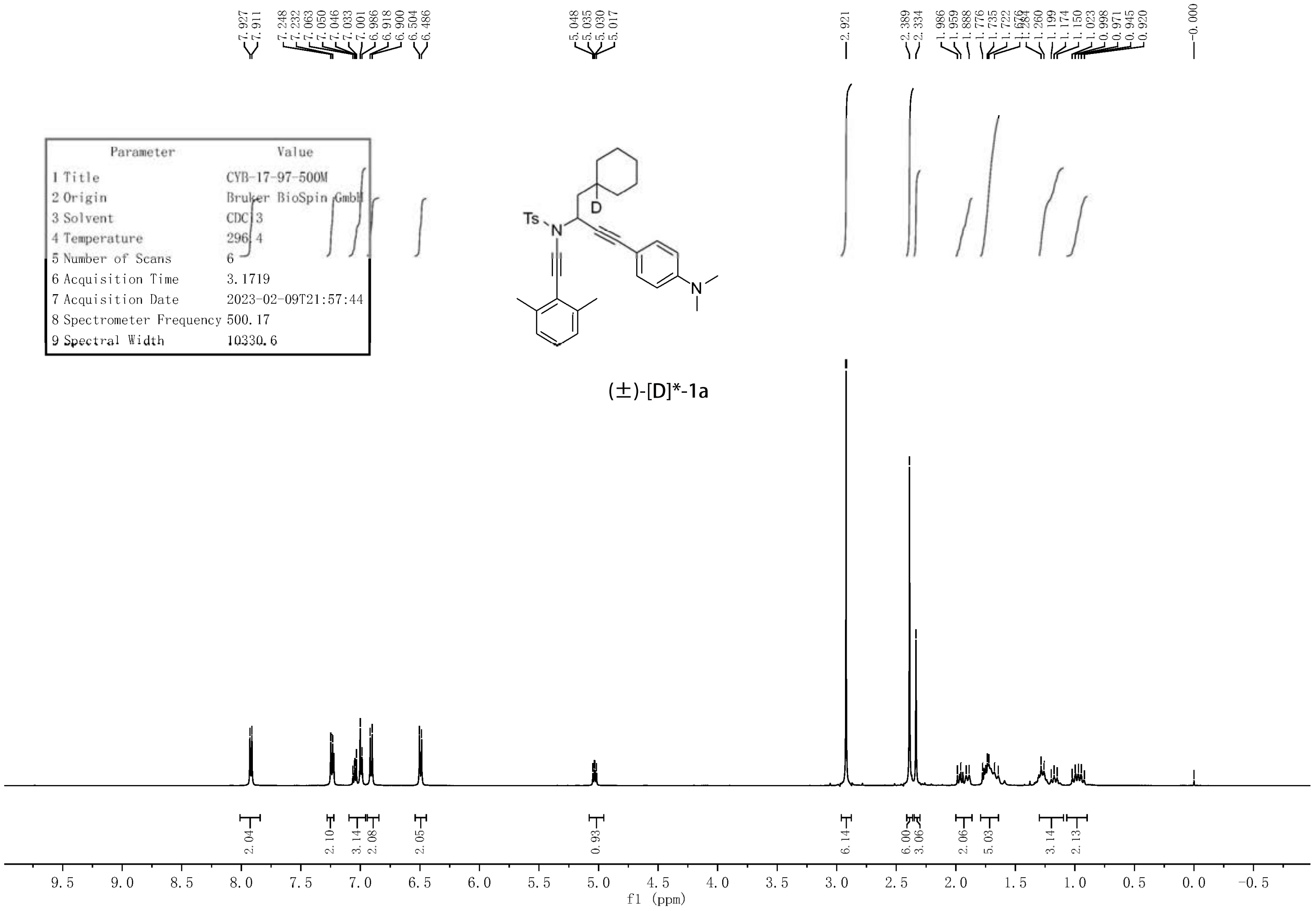

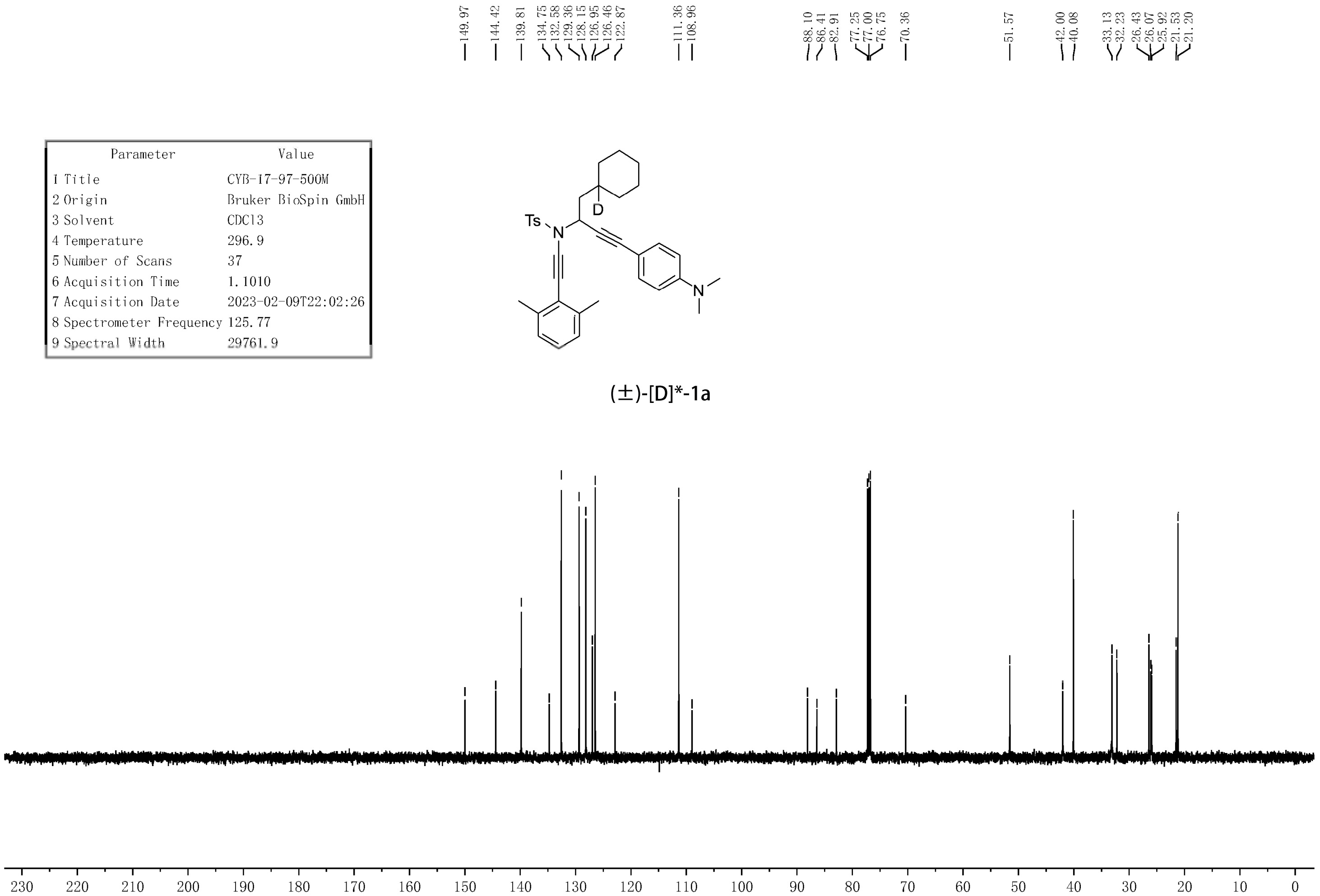

Supplementary Figure 162. ^{1}H and ^{13}C NMR spectra for (±)-[D]*-1a

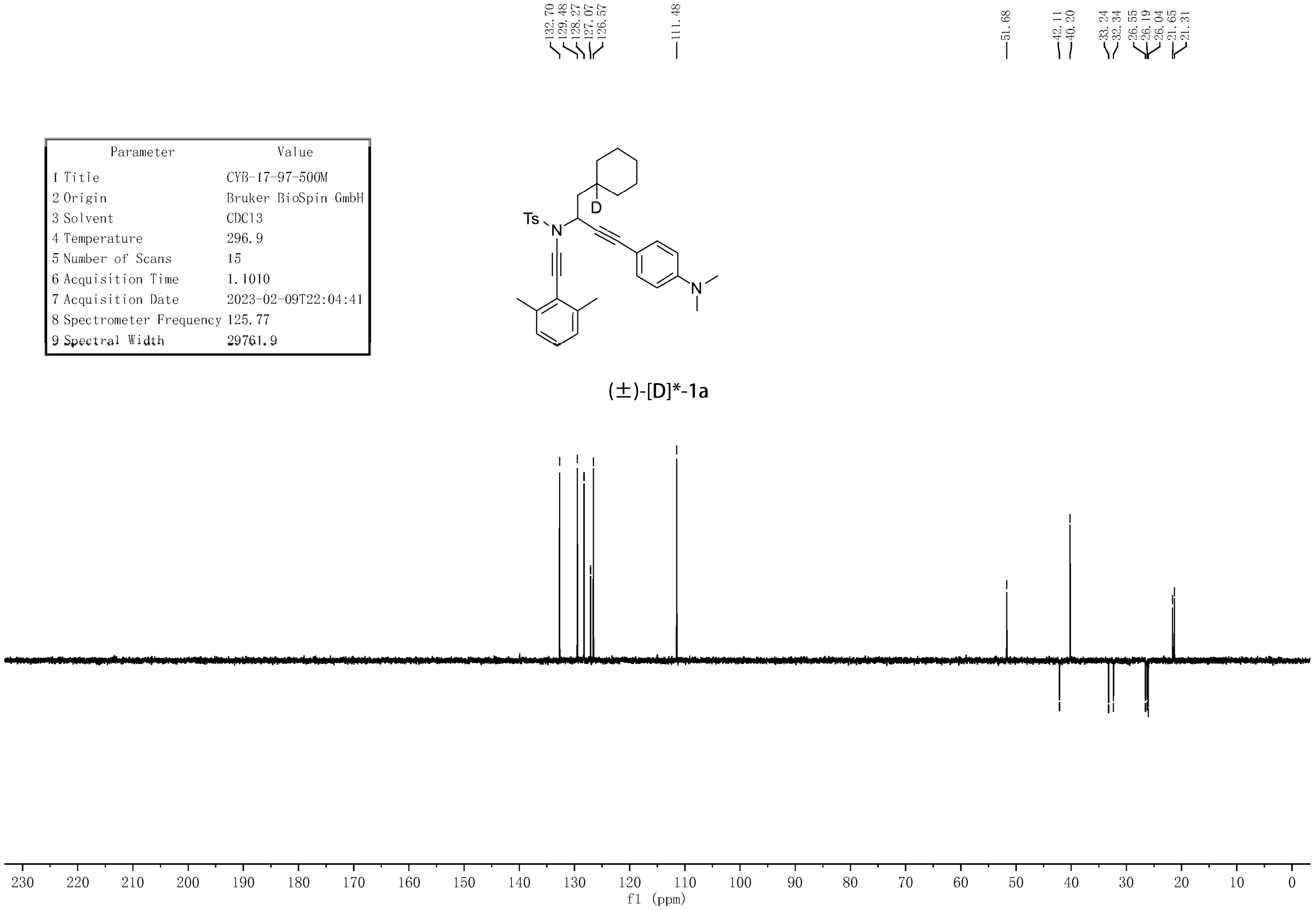

Parameter	Value
1 Title	CYB-17-97-500M
2 Origin	Bruker BioSpin GmbH
3 Solvent	CDCl3
4 Temperature	296.9
5 Number of Scans	15
6 Acquisition Time	1.1010
7 Acquisition Date	2023-02-09T22:04:41
8 Spectrometer Frequency	125.77
9 Spectral Width	29761.9

Supplementary Figure 163. DEPT-135 NMR spectra for (±)-[D]*-1a

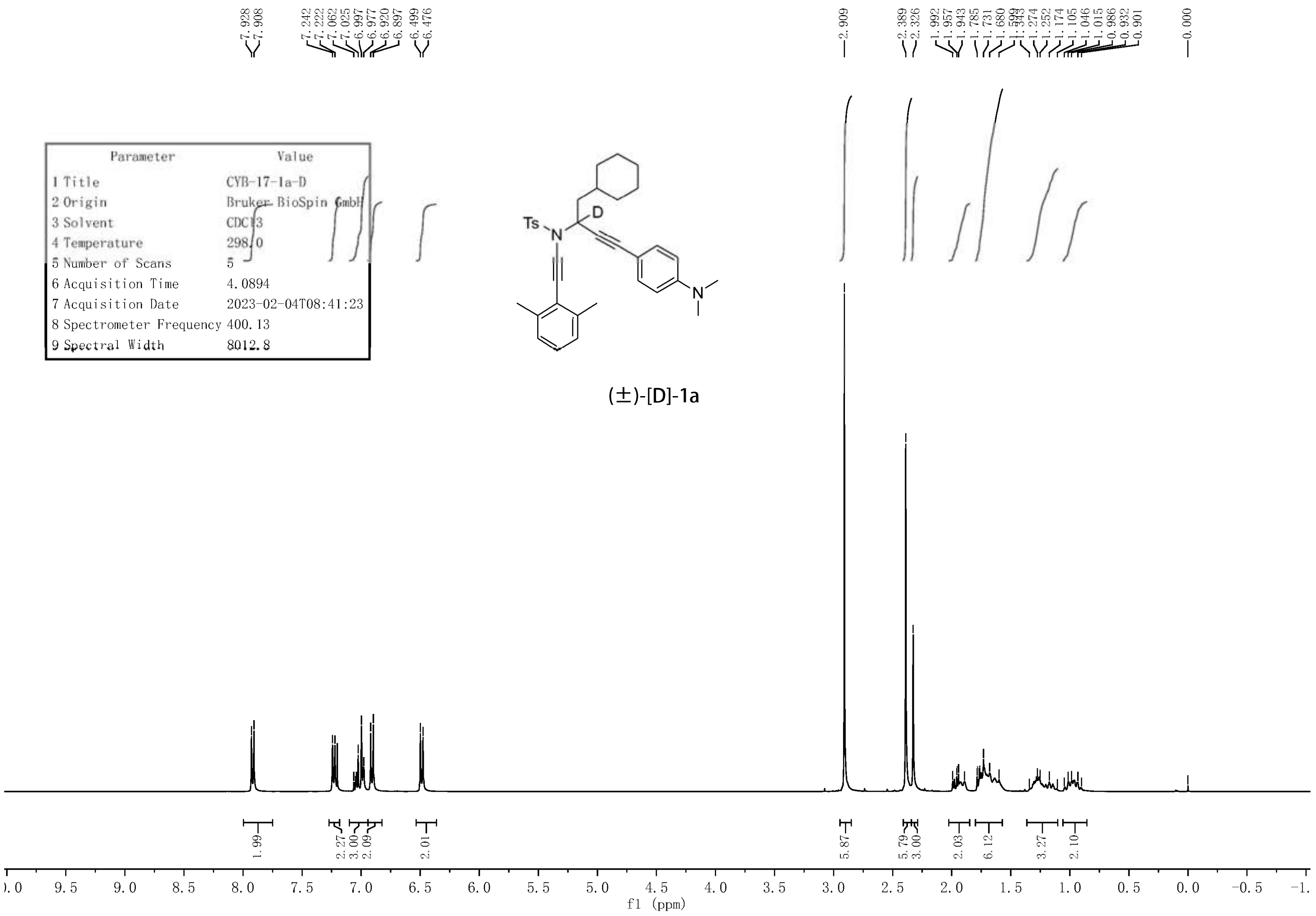

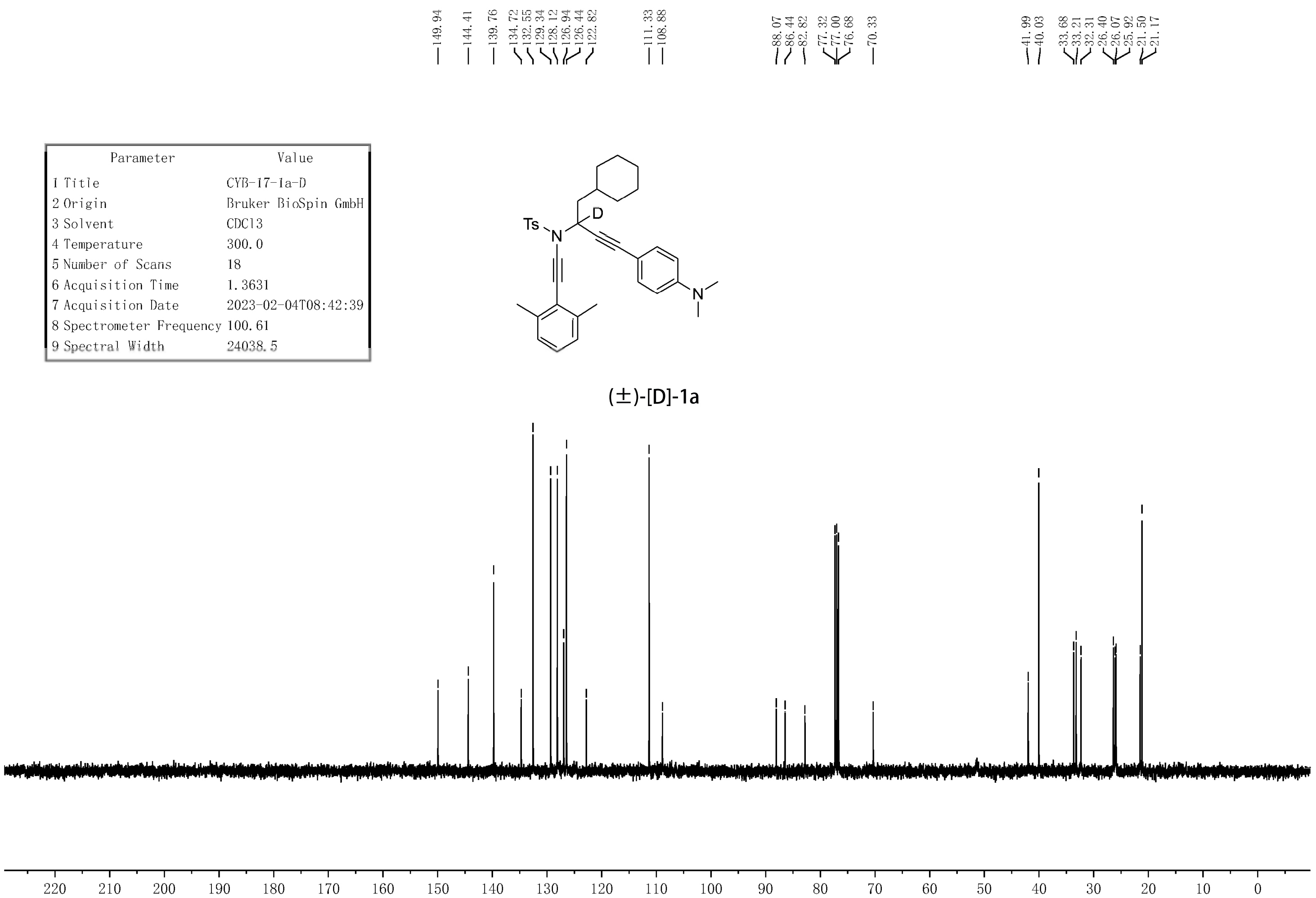

Supplementary Figure 164. ^{1}H and ^{13}C NMR spectra for (±)-[D]-1a

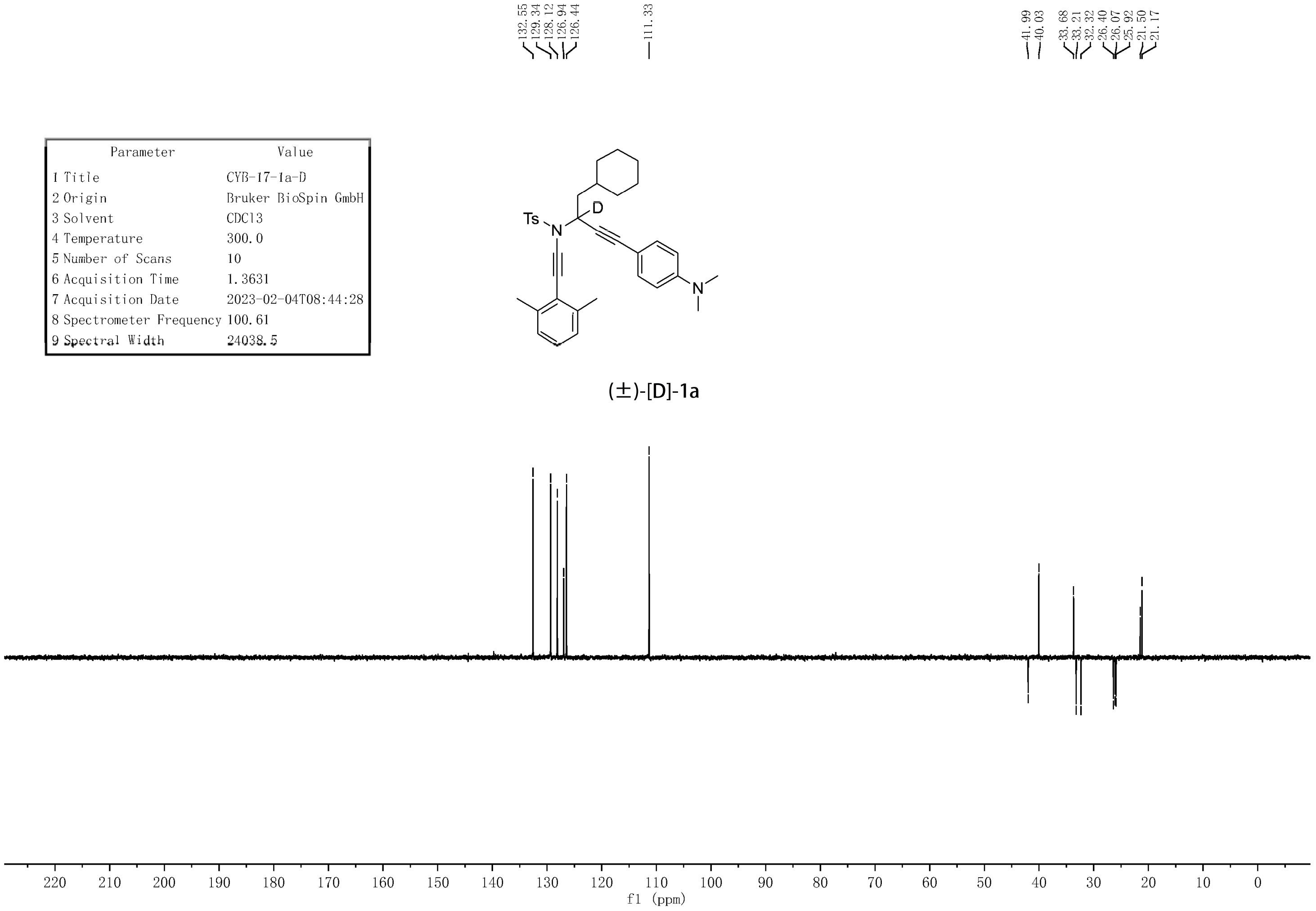

Parameter	Value
1 Title	CYB-17-1a-D
2 Origin	Bruker BioSpin GmbH
3 Solvent	CDCl3
4 Temperature	300.0
5 Number of Scans	10
6 Acquisition Time	1.3631
7 Acquisition Date	2023-02-04T08:44:28
8 Spectrometer Frequency	100.61
9 Spectral Width	24038.5

Supplementary Figure 165. DEPT-135 NMR spectra for (±)-[D]-1a

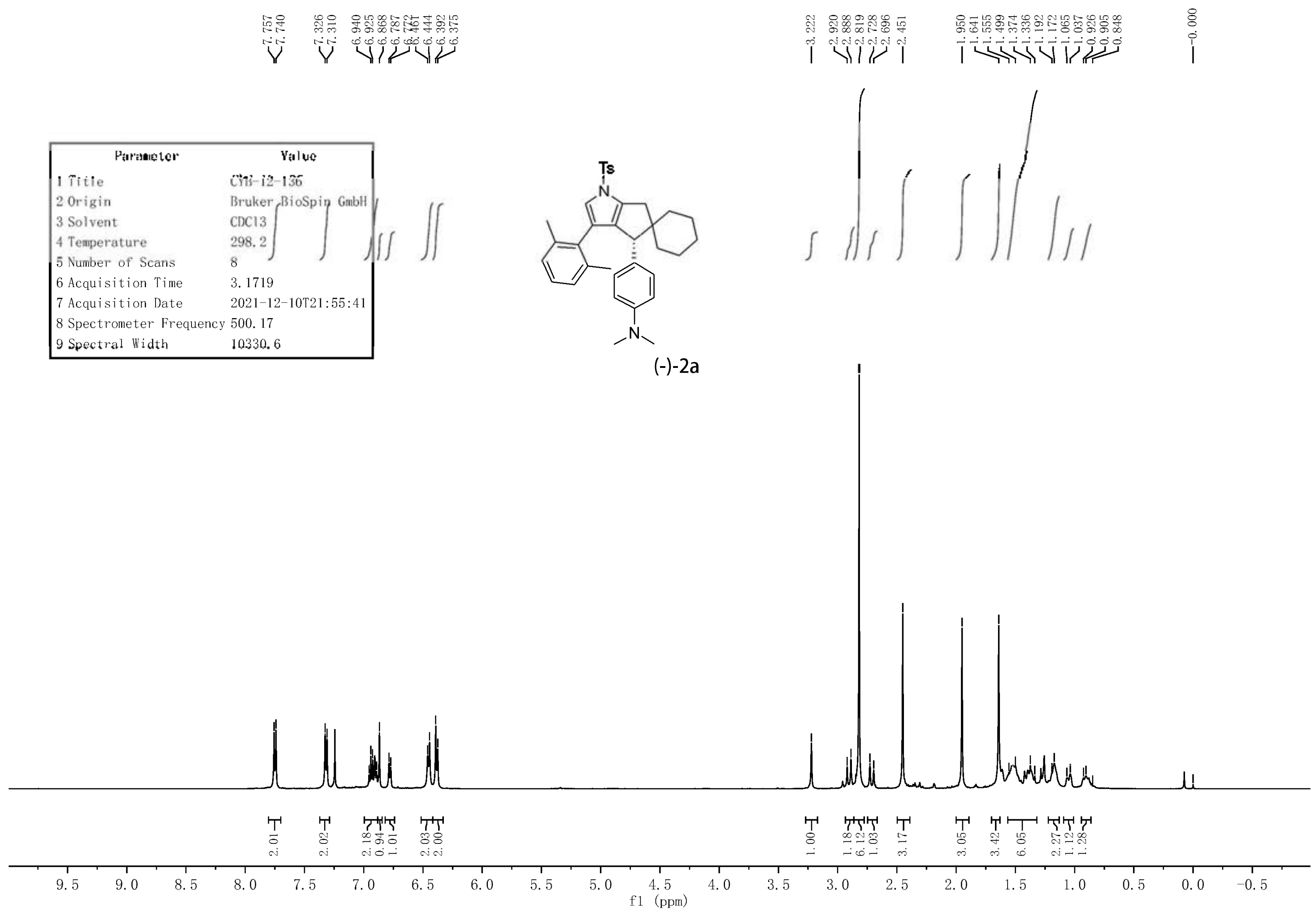

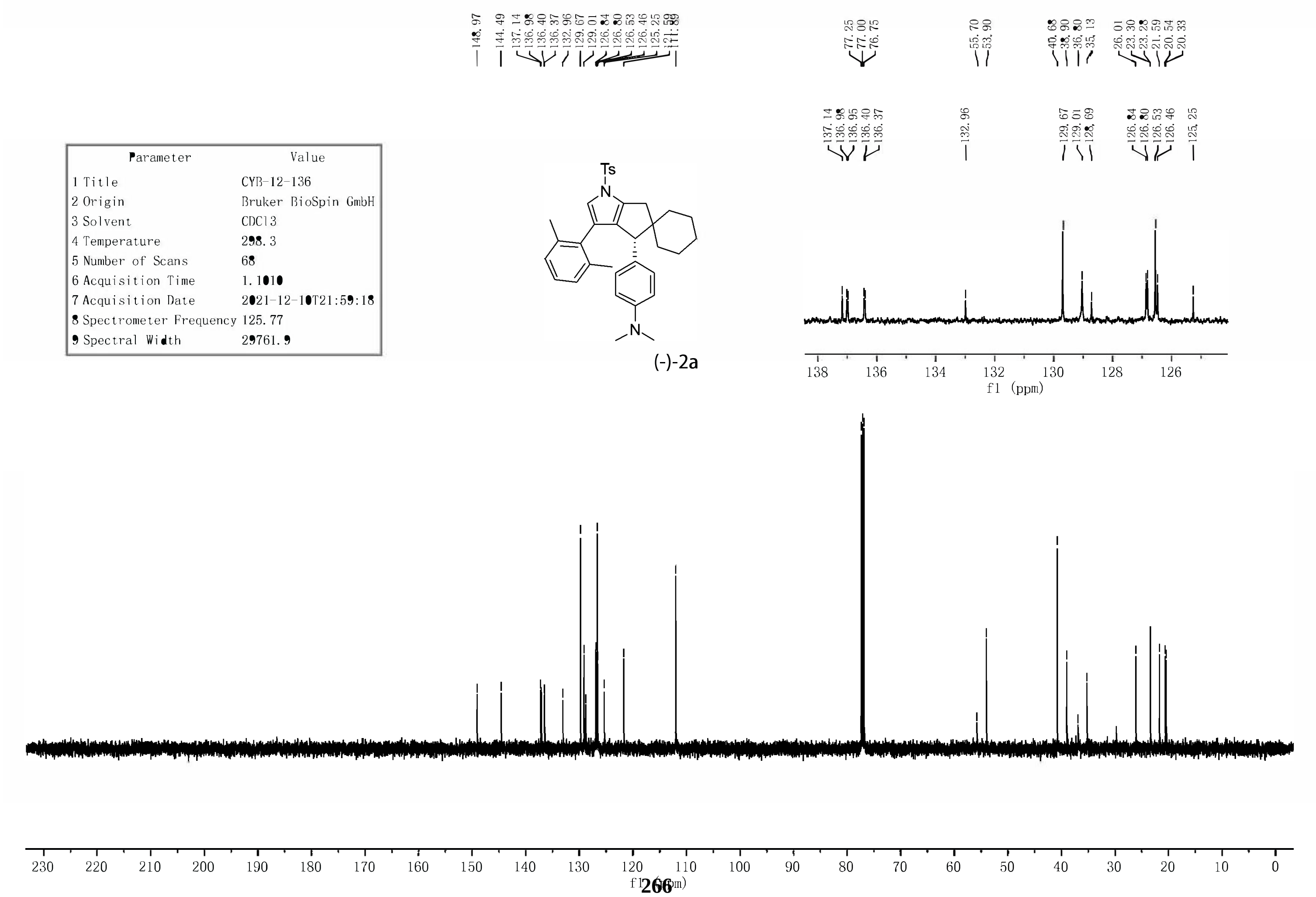

Supplementary Figure 166. ^{1}H and ^{13}C NMR spectra for (-)-2a

Parameter	Value
1 Title	CYB-12-136
2 Origin	Bruker BioSpin GmbH
3 Solvent	CDCl3
4 Temperature	298.8
5 Number of Scans	29
6 Acquisition Time	1.1010
7 Acquisition Date	2021-12-10T22:03:30
8 Spectrometer Frequency	125.77
9 Spectral Width	29761.9

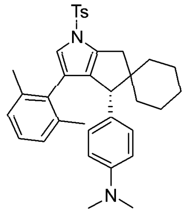

(-)-2a

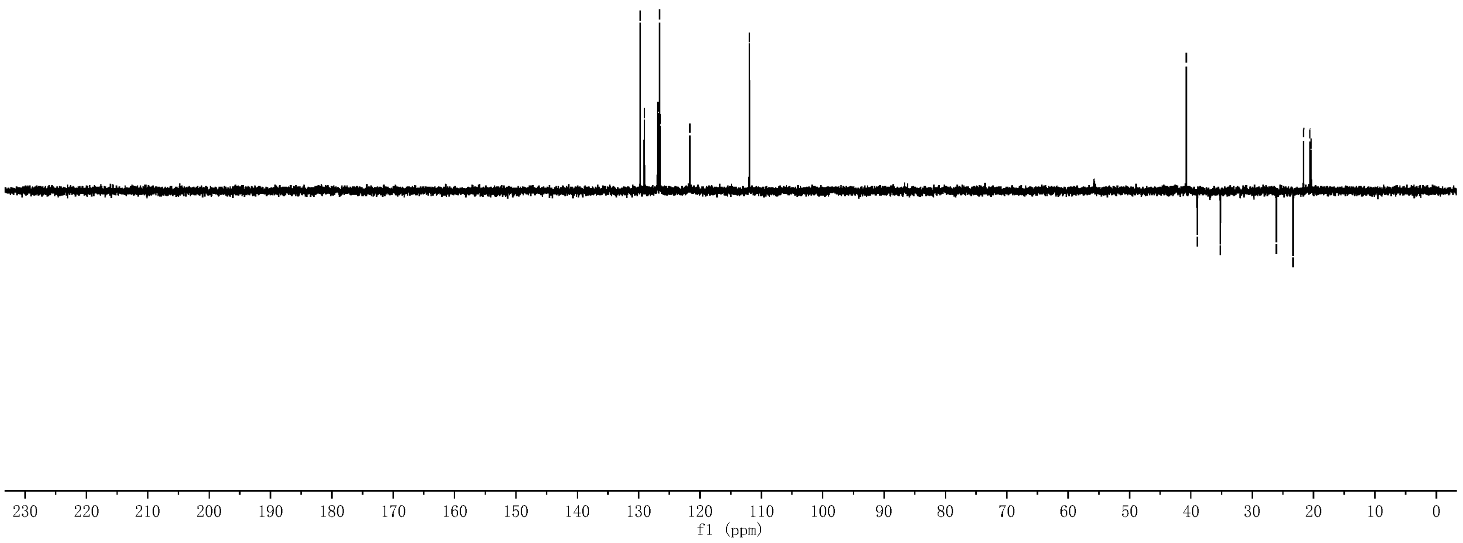

Supplementary Figure 167. DEPT-135 NMR spectra for (-)-2a

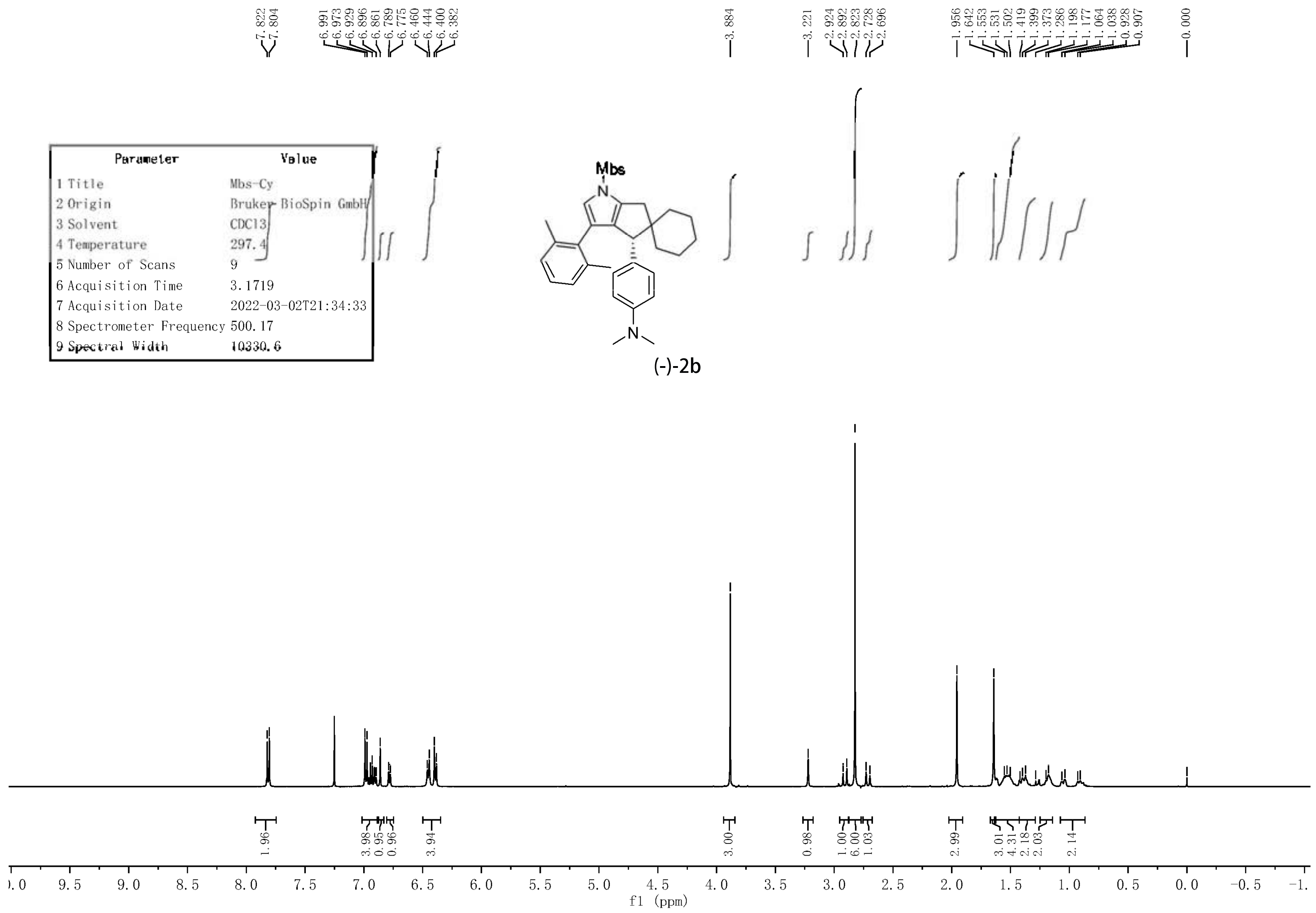

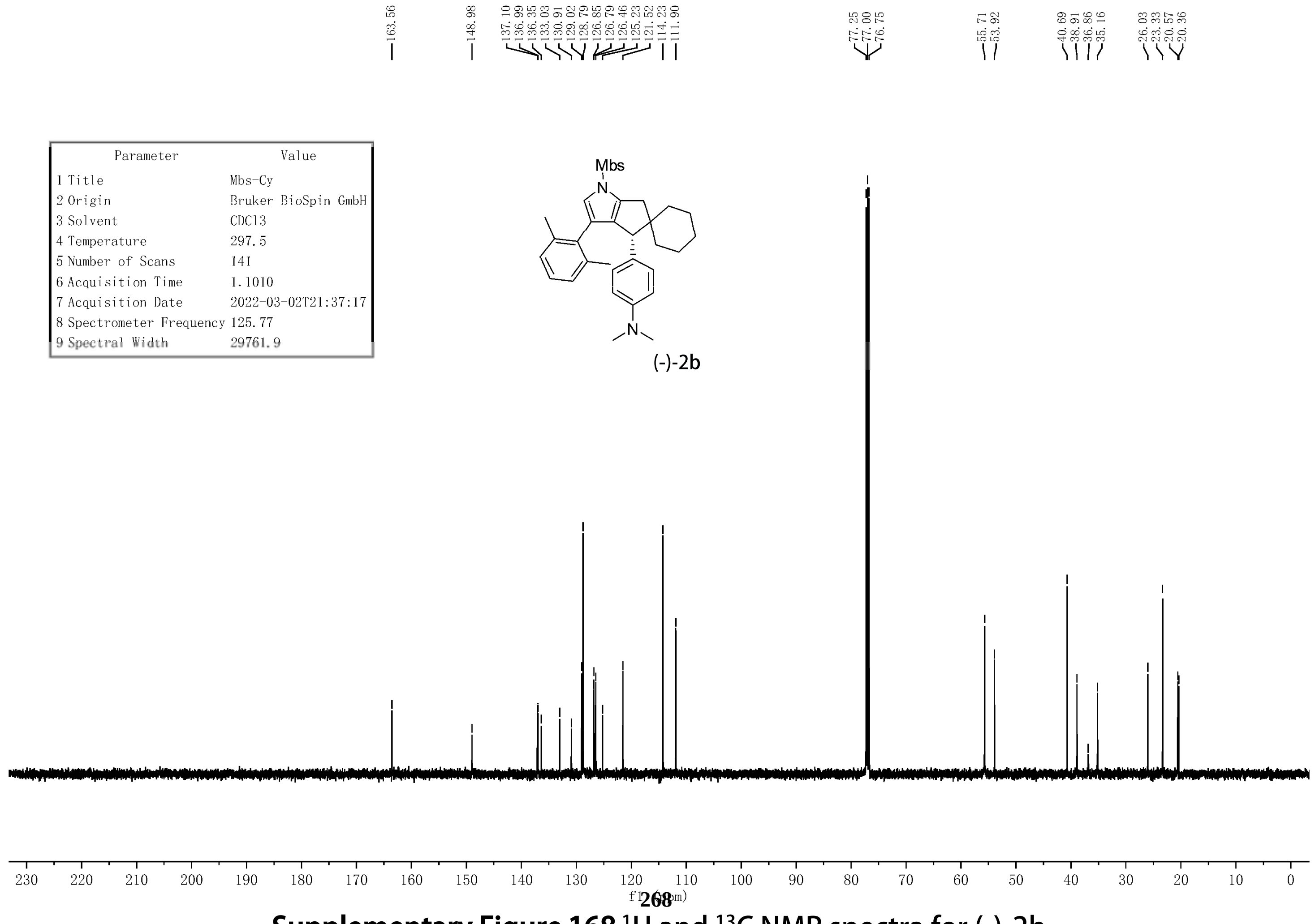

Supplementary Figure 168 ^{1}H and ^{13}C NMR spectra for (-)-2b

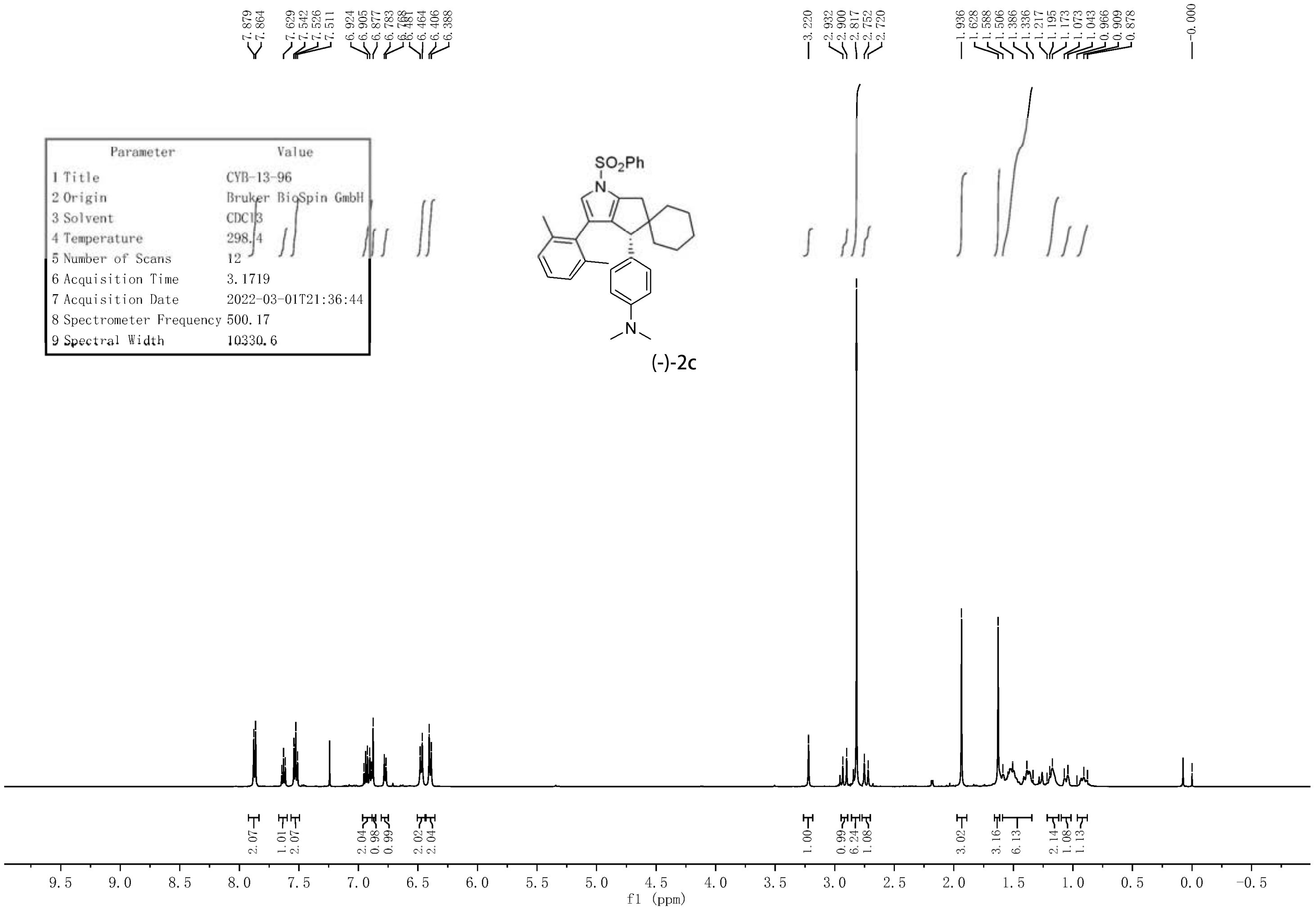

Parameter	Value
1 Title	CYB-13-96
2 Origin	Bruker BioSpin GmbH
3 Solvent	CDCl3
4 Temperature	298.4
5 Number of Scans	12
6 Acquisition Time	3.1719
7 Acquisition Date	2022-03-01T21:36:44
8 Spectrometer Frequency	500.17
9 Spectral Width	10330.6

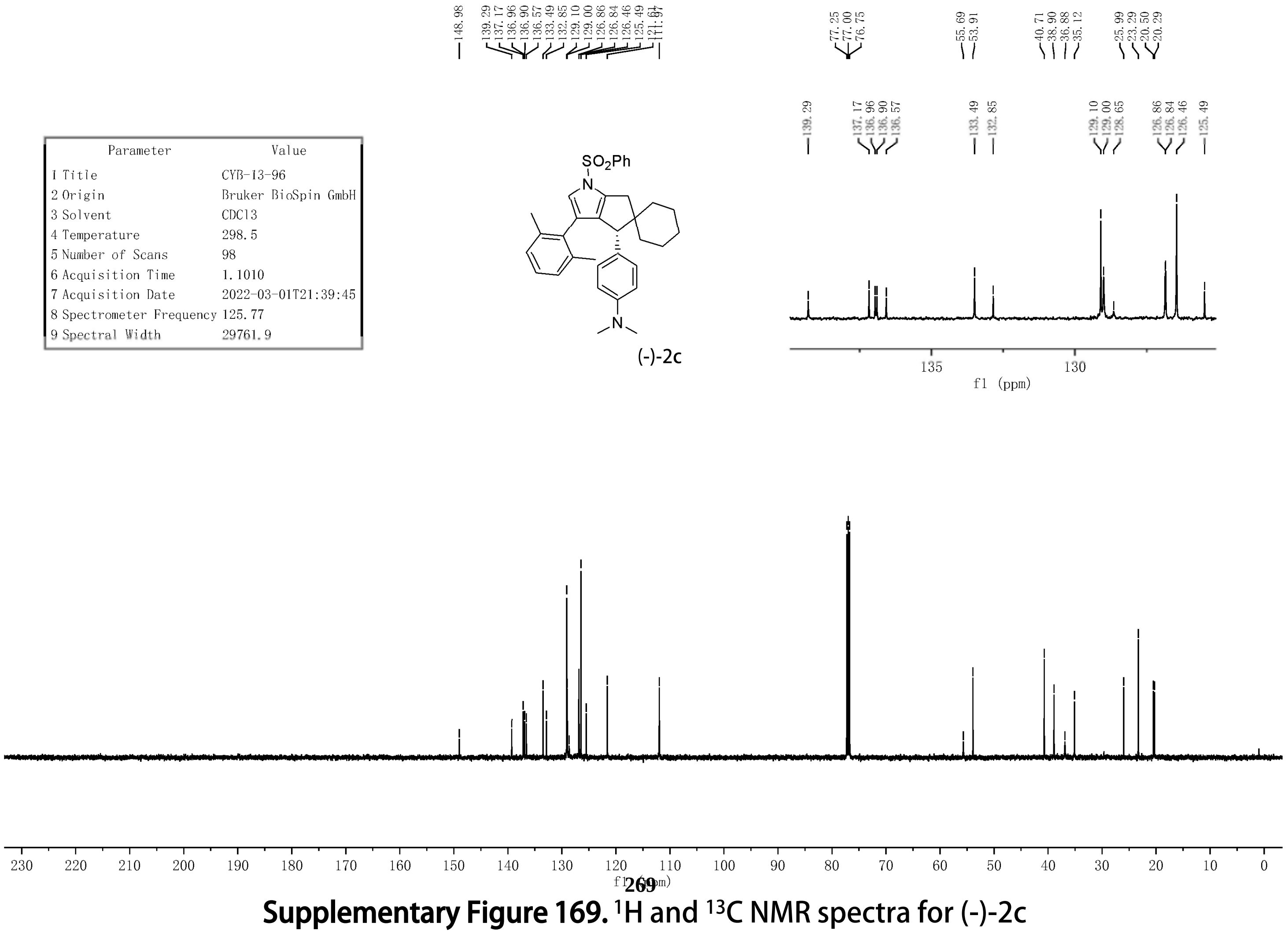

Parameter	Value
1 Title	CYB-13-96
2 Origin	Bruker BioSpin GmbH
3 Solvent	CDCl3
4 Temperature	298.5
5 Number of Scans	98
6 Acquisition Time	1.1010
7 Acquisition Date	2022-03-01T21:39:45
8 Spectrometer Frequency	125.77
9 Spectral Width	29761.9

Supplementary Figure 169. ^{1}H and ^{13}C NMR spectra for (-)-2c

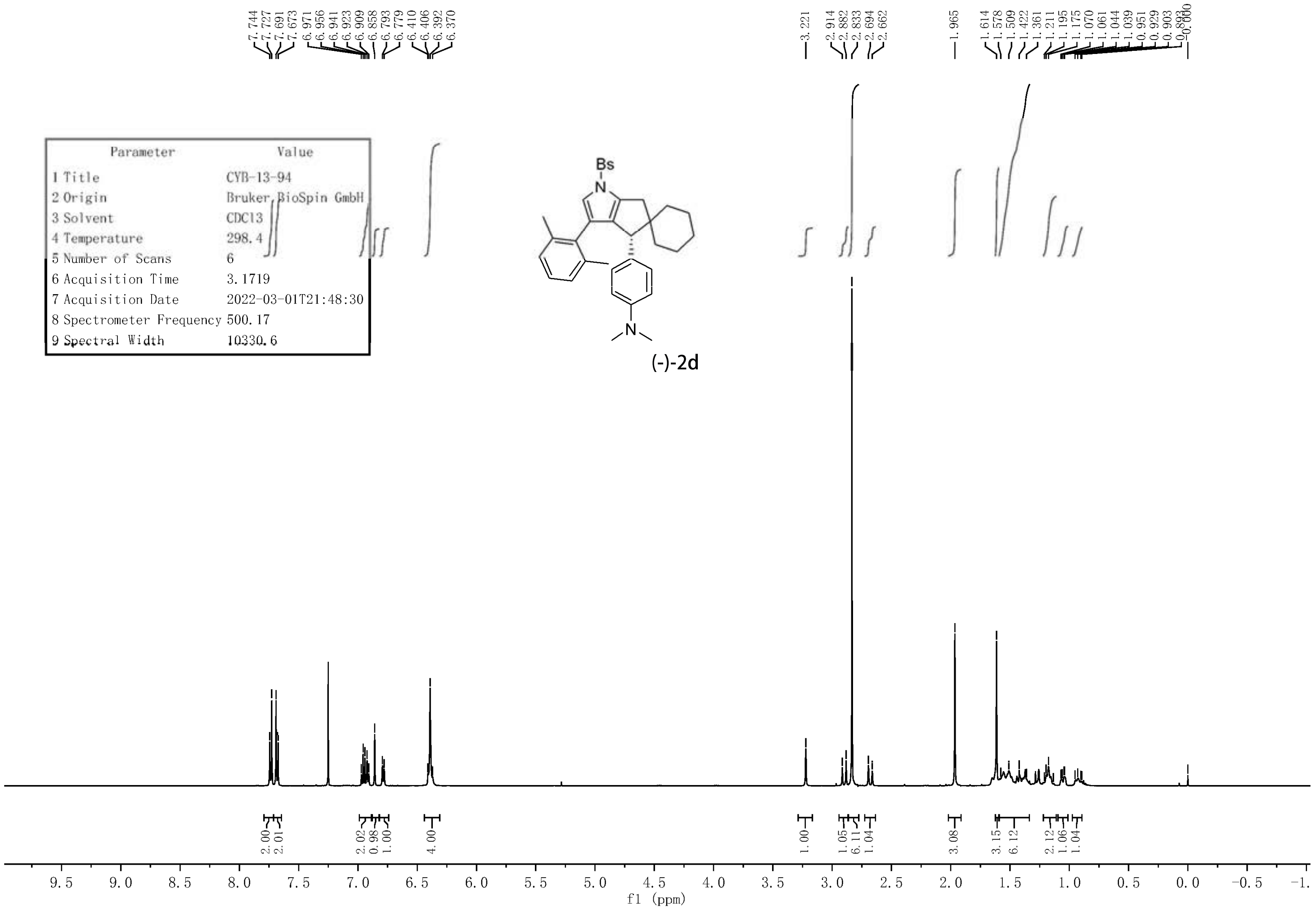

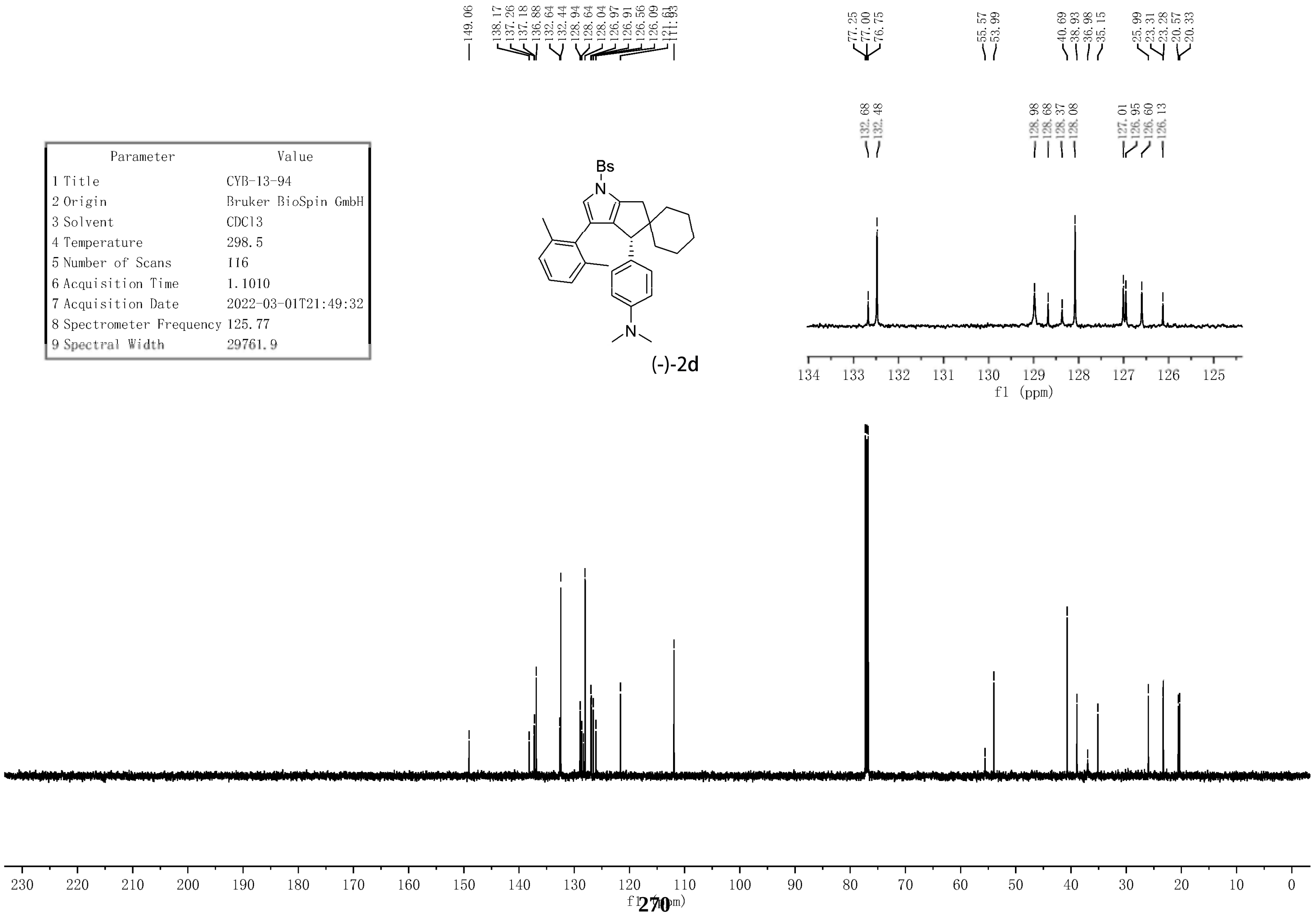

Supplementary Figure 170. ^{1}H and ^{13}C NMR spectra for (-)-2d

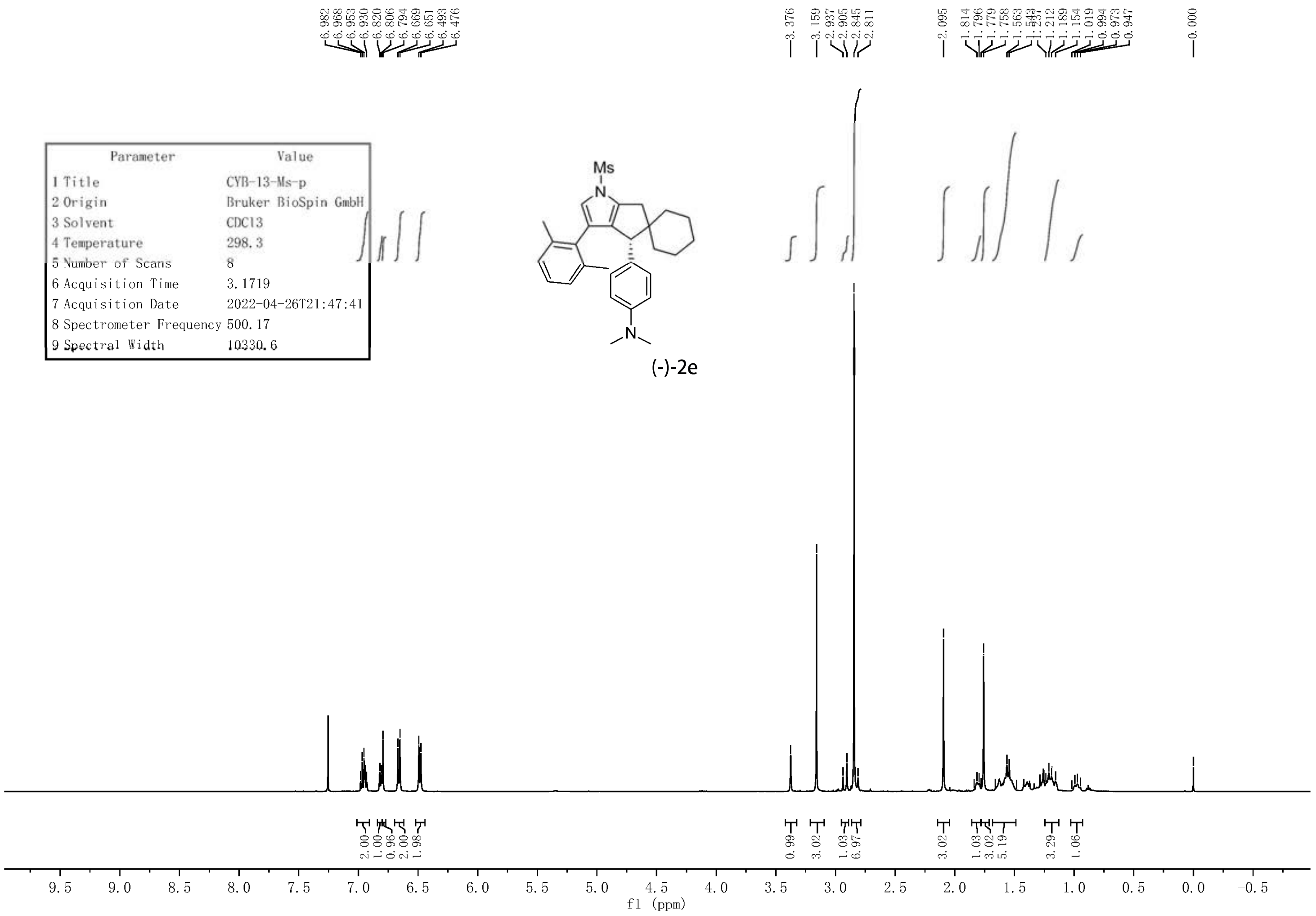

Parameter	Value
1 Title	CYB-13-Ms-p
2 Origin	Bruker BioSpin GmbH
3 Solvent	CDCl3
4 Temperature	298.3
5 Number of Scans	8
6 Acquisition Time	3.1719
7 Acquisition Date	2022-04-26T21:47:41
8 Spectrometer Frequency	500.17
9 Spectral Width	10330.6

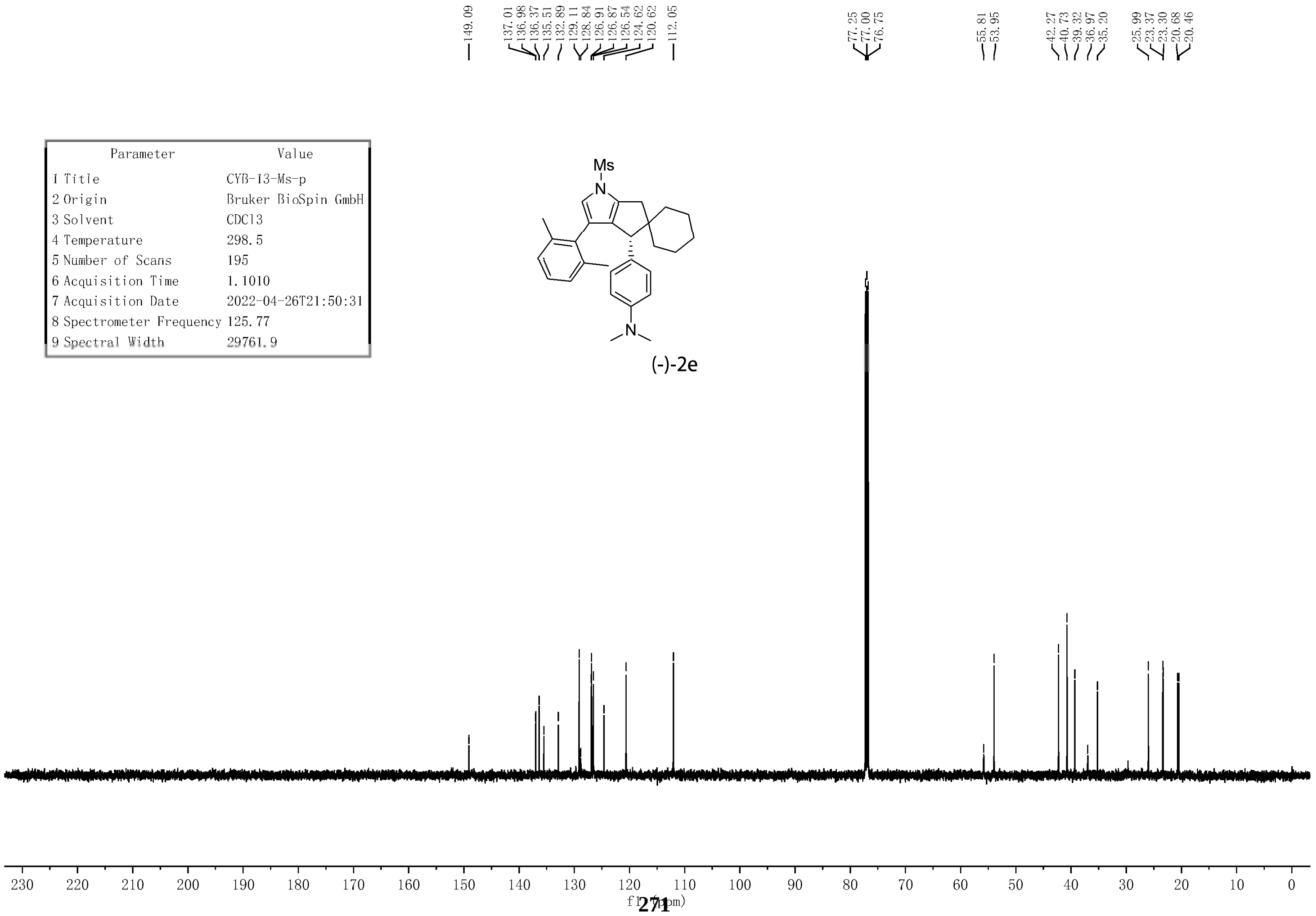

Parameter	Value
1 Title	CYB-13-Ms-p
2 Origin	Bruker BioSpin GmbH
3 Solvent	CDCl3
4 Temperature	298.5
5 Number of Scans	195
6 Acquisition Time	1.1010
7 Acquisition Date	2022-04-26T21:50:31
8 Spectrometer Frequency	125.77
9 Spectral Width	29761.9

Supplementary Figure 171. ^{1}H and ^{13}C NMR spectra for (-)-2e

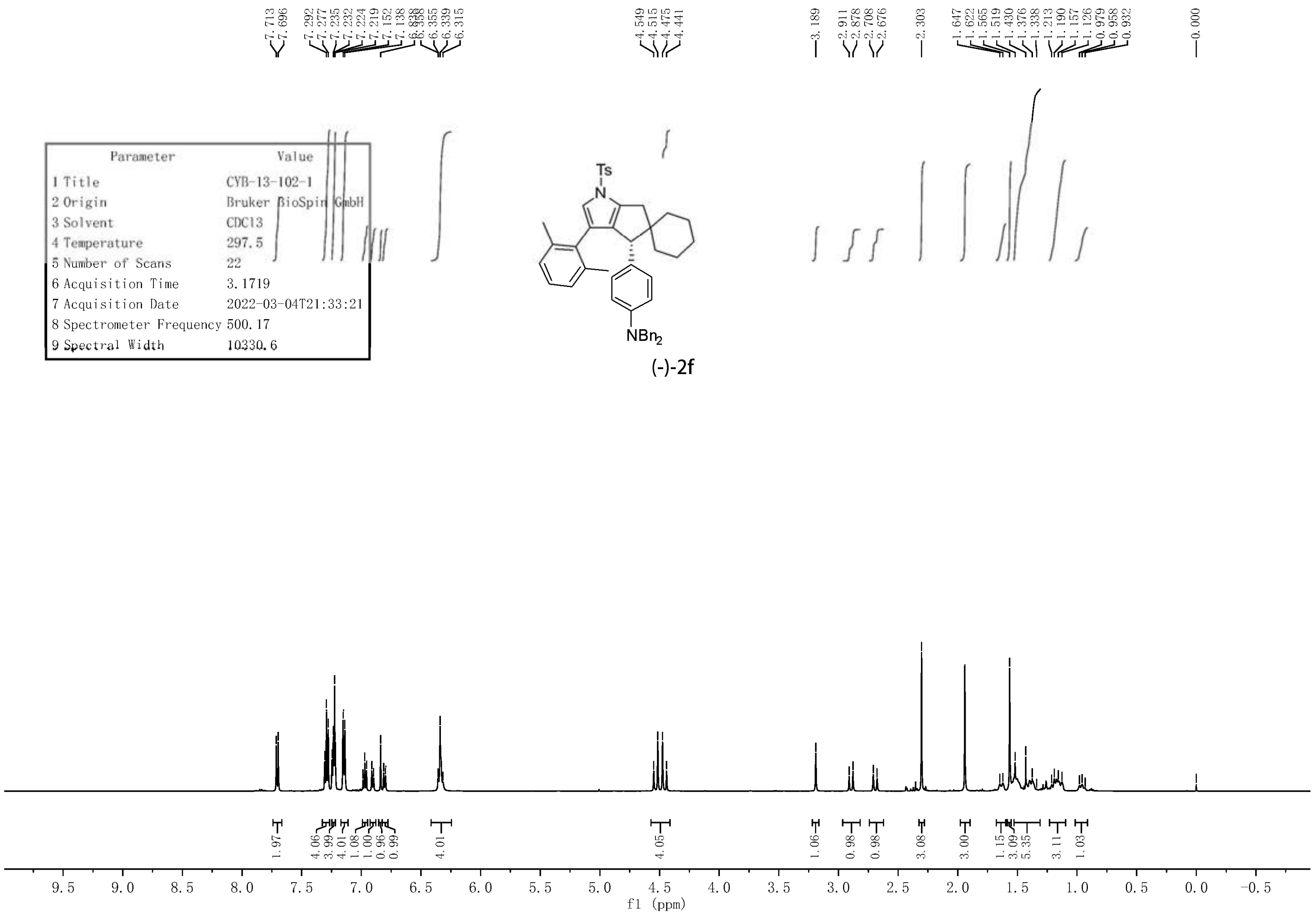

Parameter	Value
1 Title	CYB-13-102-1
2 Origin	Bruker BioSpin GmbH
3 Solvent	CDCl3
4 Temperature	297.5
5 Number of Scans	22
6 Acquisition Time	3.1719
7 Acquisition Date	2022-03-04T21:33:21
8 Spectrometer Frequency	500.17
9 Spectral Width	10330.6

(-)-2f

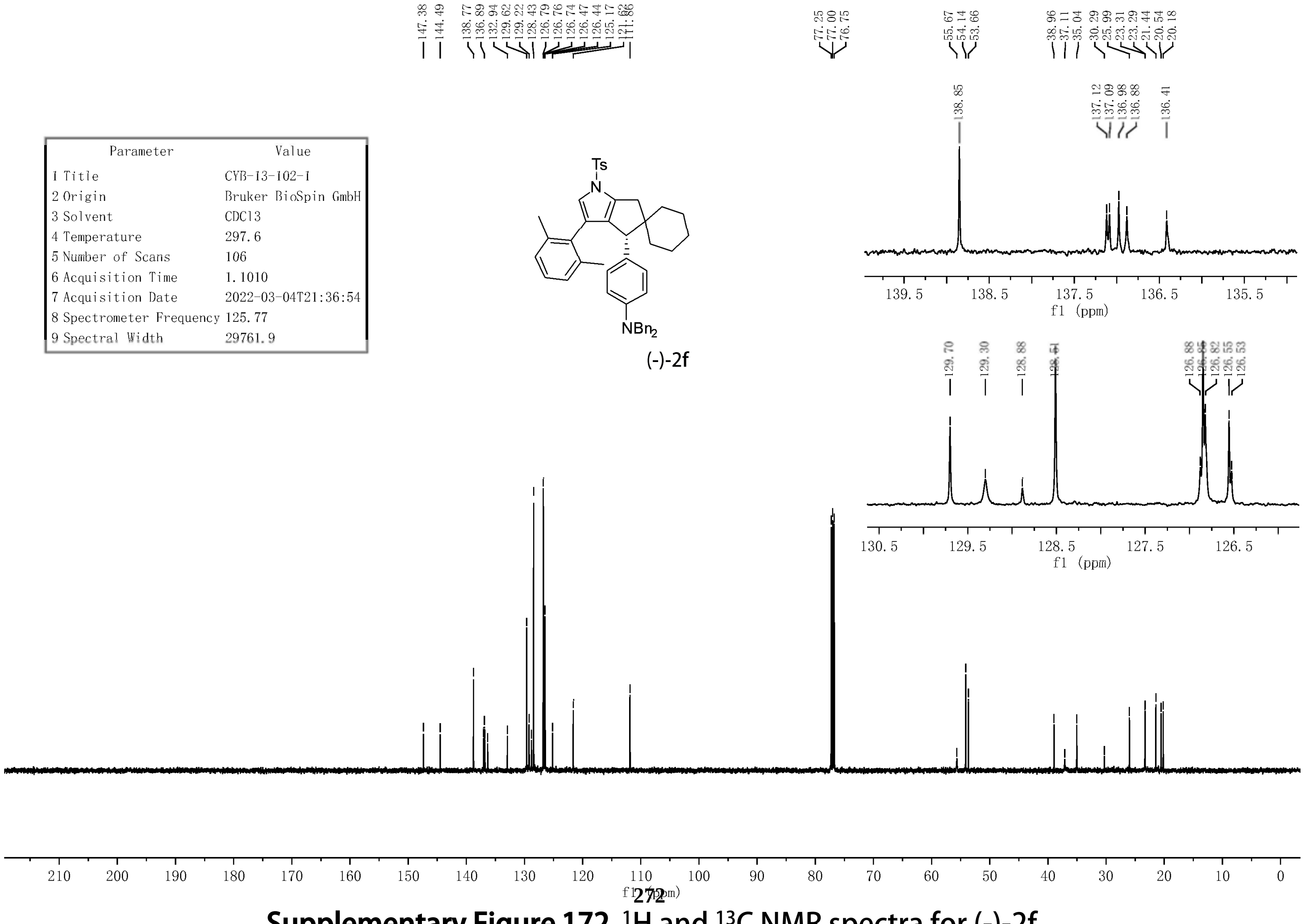

Parameter	Value
1 Title	CYB-13-102-1
2 Origin	Bruker BioSpin GmbH
3 Solvent	CDCl3
4 Temperature	297.6
5 Number of Scans	106
6 Acquisition Time	1.1010
7 Acquisition Date	2022-03-04T21:36:54
8 Spectrometer Frequency	125.77
9 Spectral Width	29761.9

(-)-2f

Supplementary Figure 172. ^{1}H and ^{13}C NMR spectra for (-)-2f

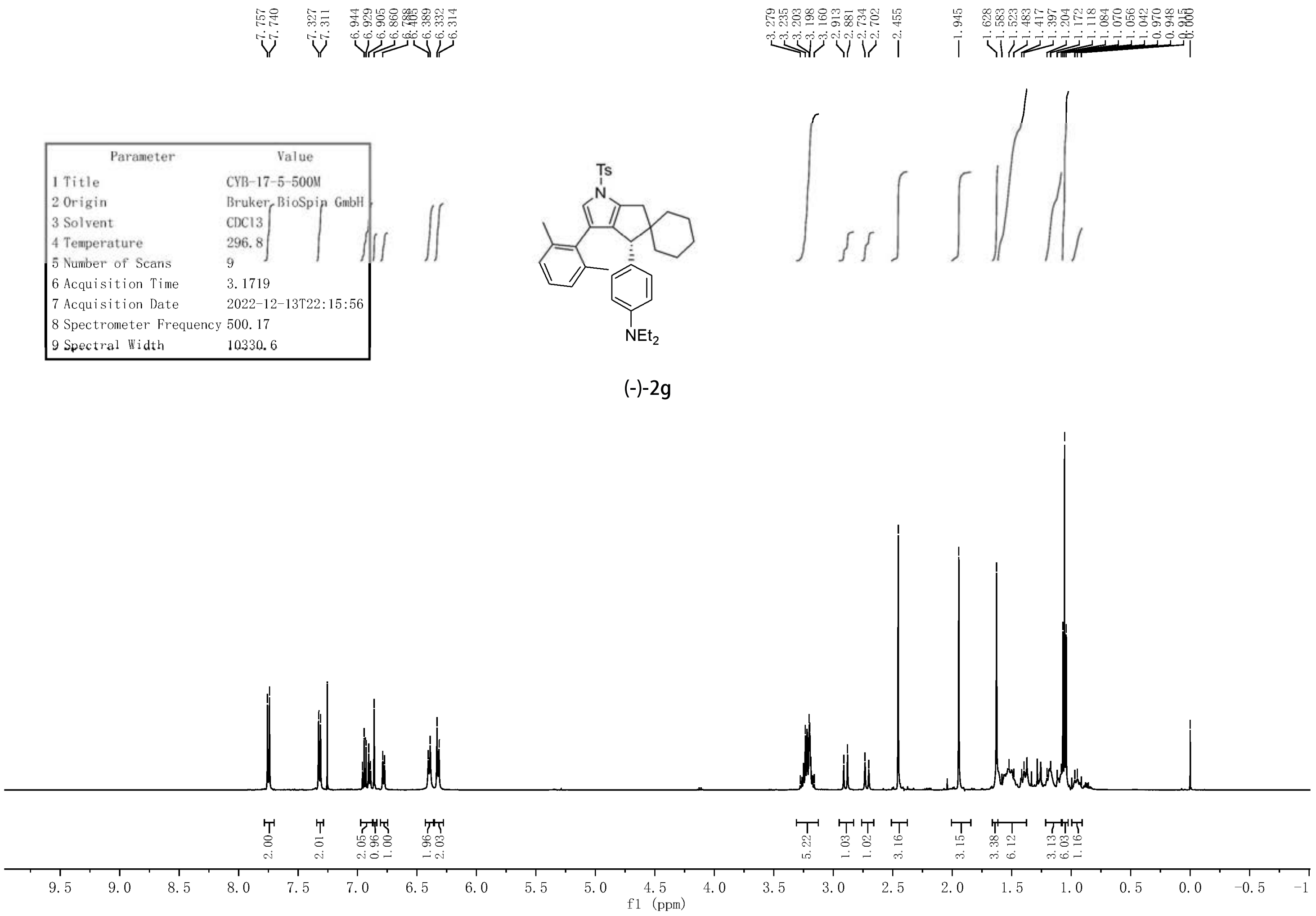

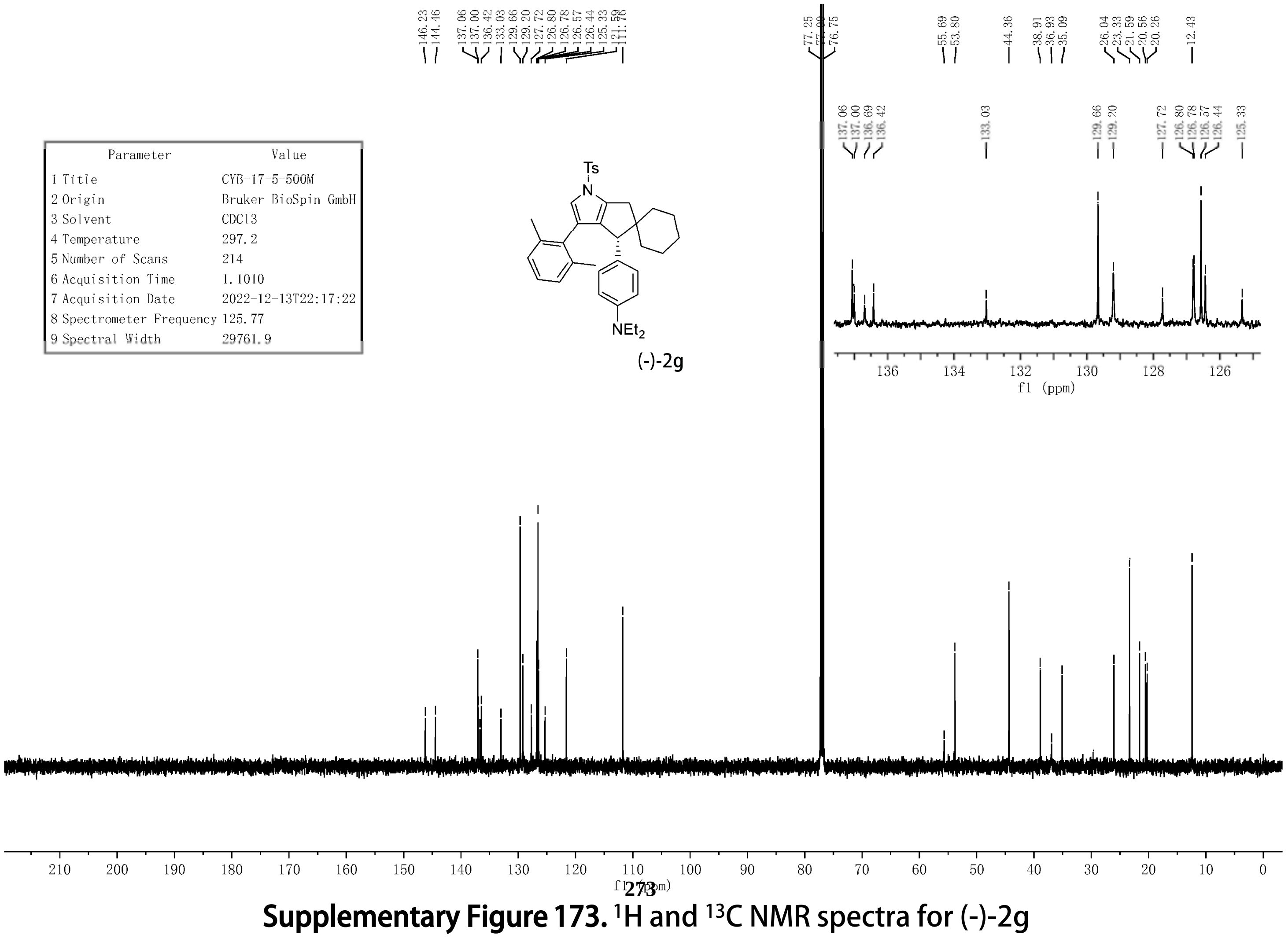

Supplementary Figure 173. ^{1}H and ^{13}C NMR spectra for (-)-2g

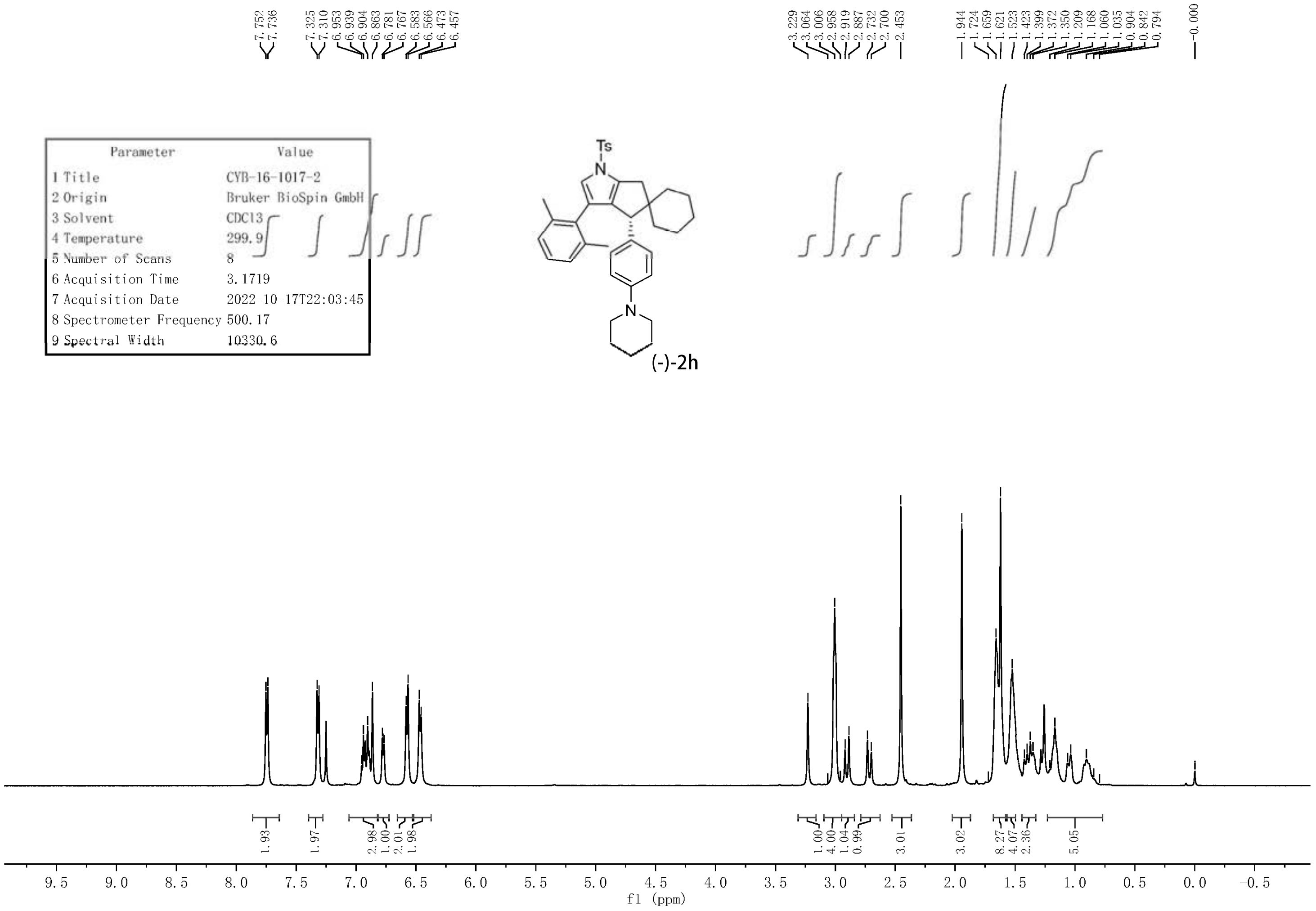

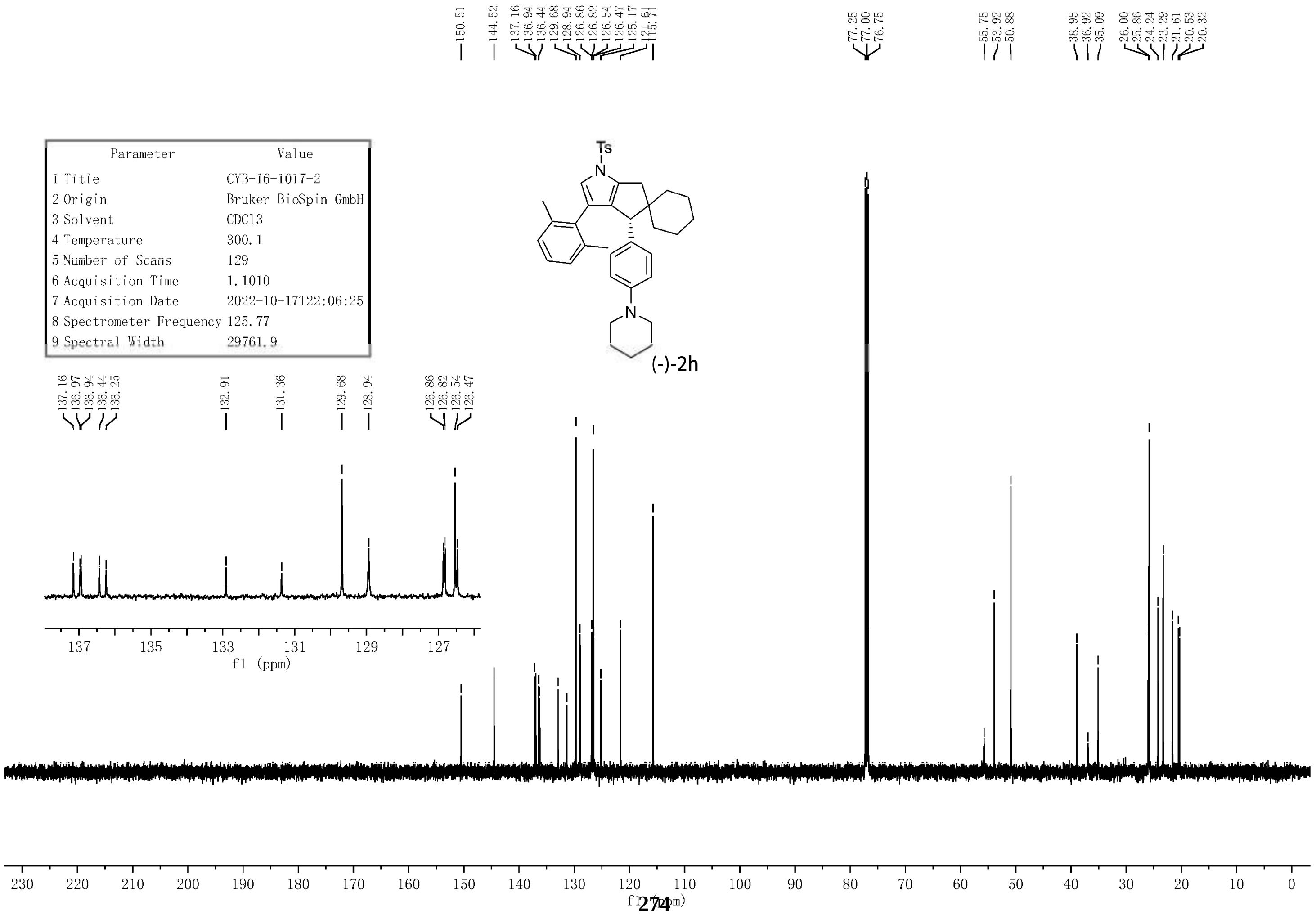

Supplementary Figure 174. ^{1}H and ^{13}C NMR spectra for (-)-2h

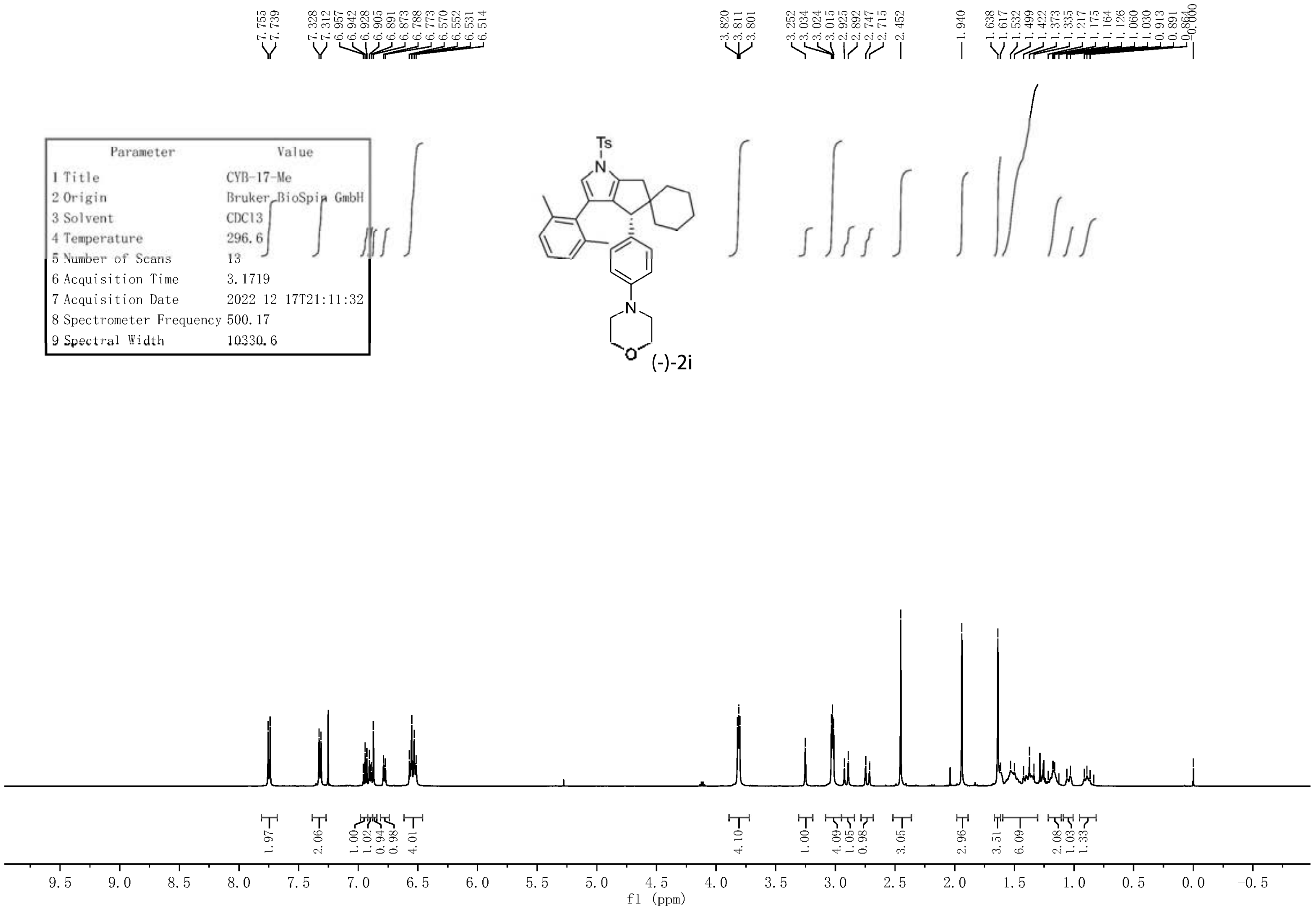

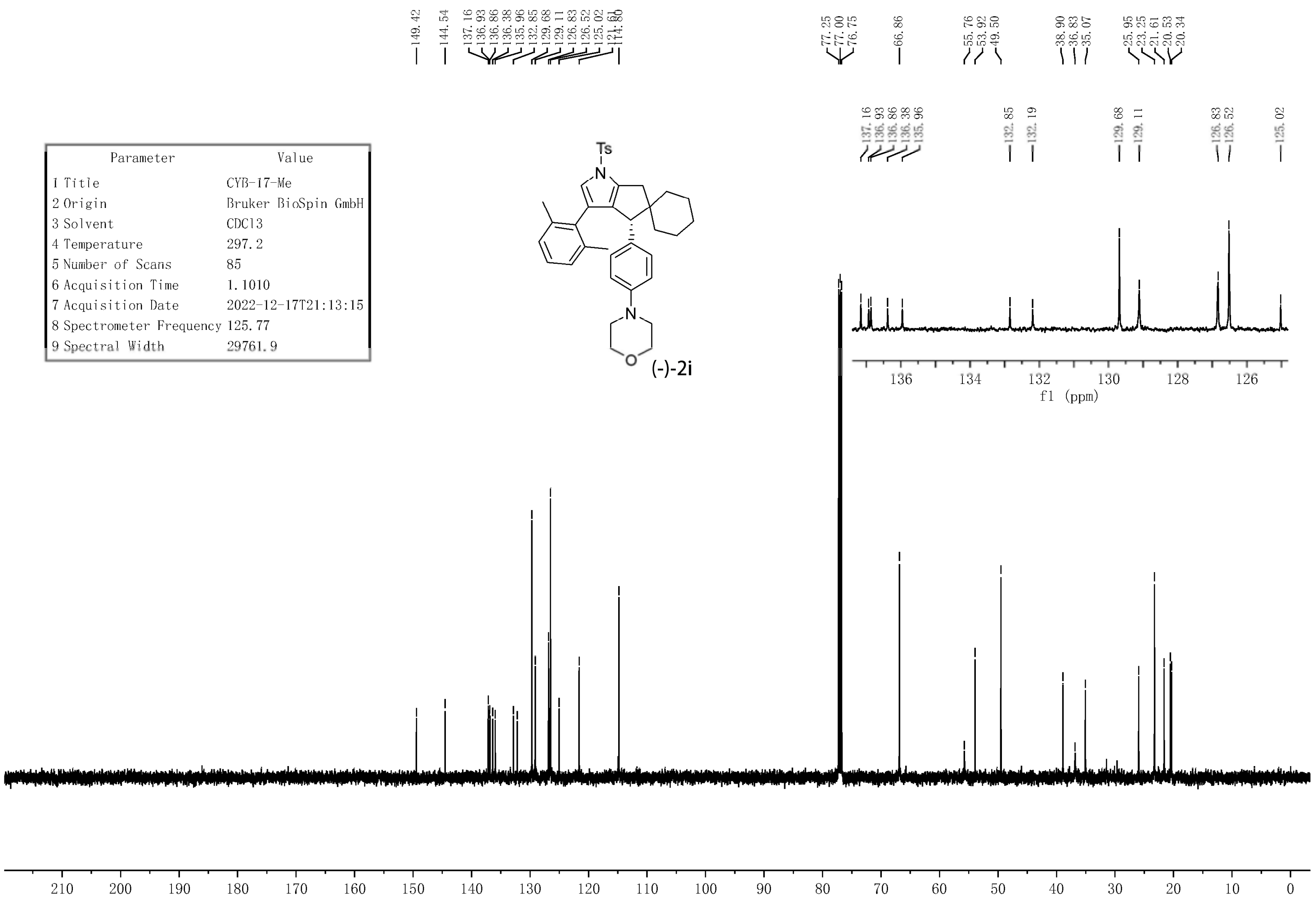

Supplementary Figure 175. ^{1}H and ^{13}C NMR spectra for (-)-2i

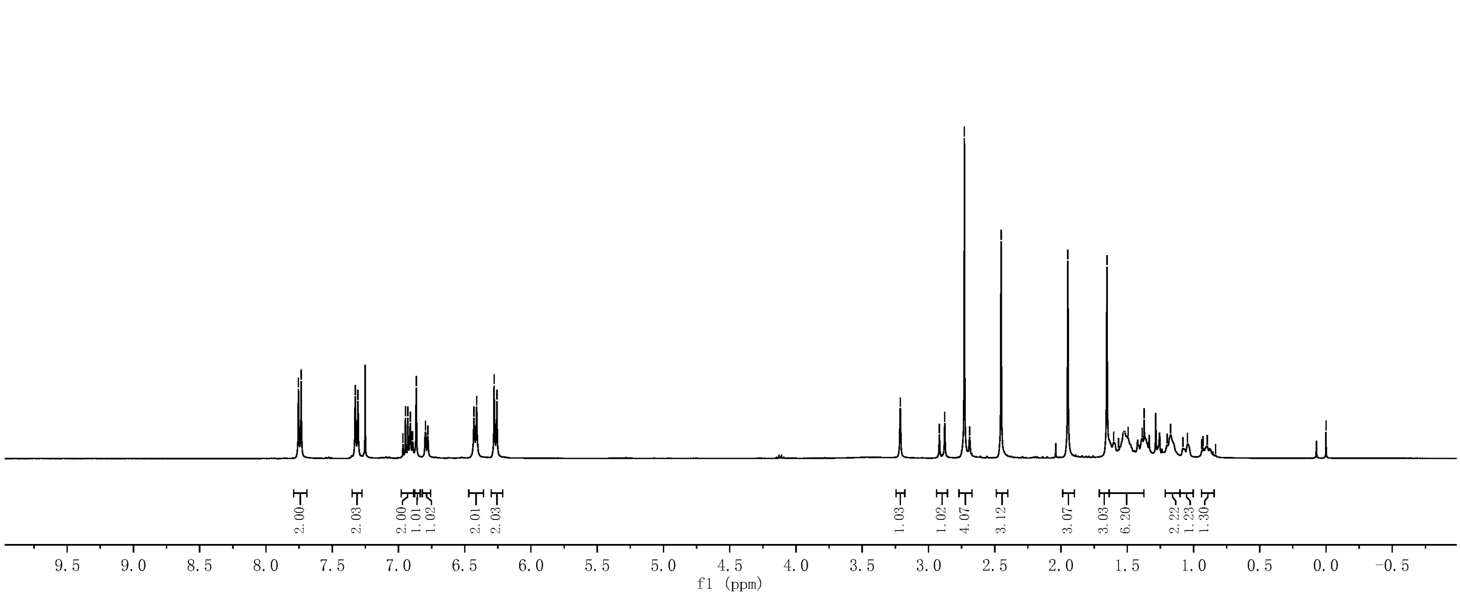

Parameter	Value
1 Title	CYB-17-210-400M
2 Origin	Bruker BioSpin GmbH
3 Solvent	CDCl3
4 Temperature	298.0
5 Number of Scans	256
6 Acquisition Time	4.0894
7 Acquisition Date	2023-03-08T23:30:38
8 Spectrometer Frequency	400.13
9 Spectral Width	8012.8

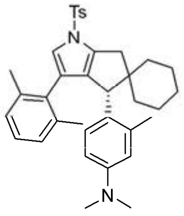

(-)-2j

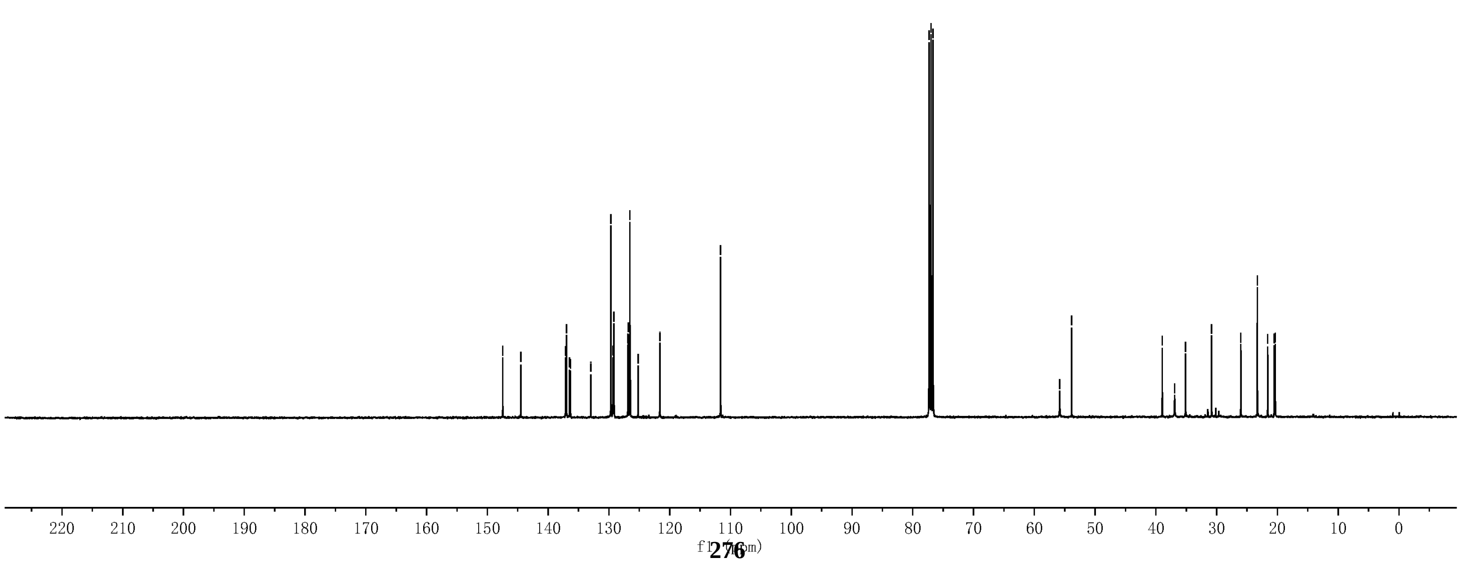

Parameter	Value
1 Title	CYB-17-210-400M
2 Origin	Bruker BioSpin GmbH
3 Solvent	CDCl3
4 Temperature	300.0
5 Number of Scans	4096
6 Acquisition Time	1.3631
7 Acquisition Date	2023-03-09T03:46:33
8 Spectrometer Frequency	100.61
9 Spectral Width	24038.5

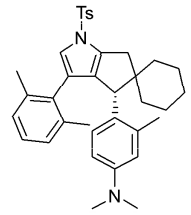

(-)-2j

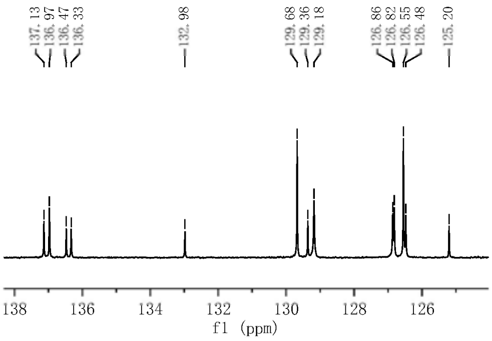

Supplementary Figure 176. ^{1}H and ^{13}C NMR spectra for (-)-2j

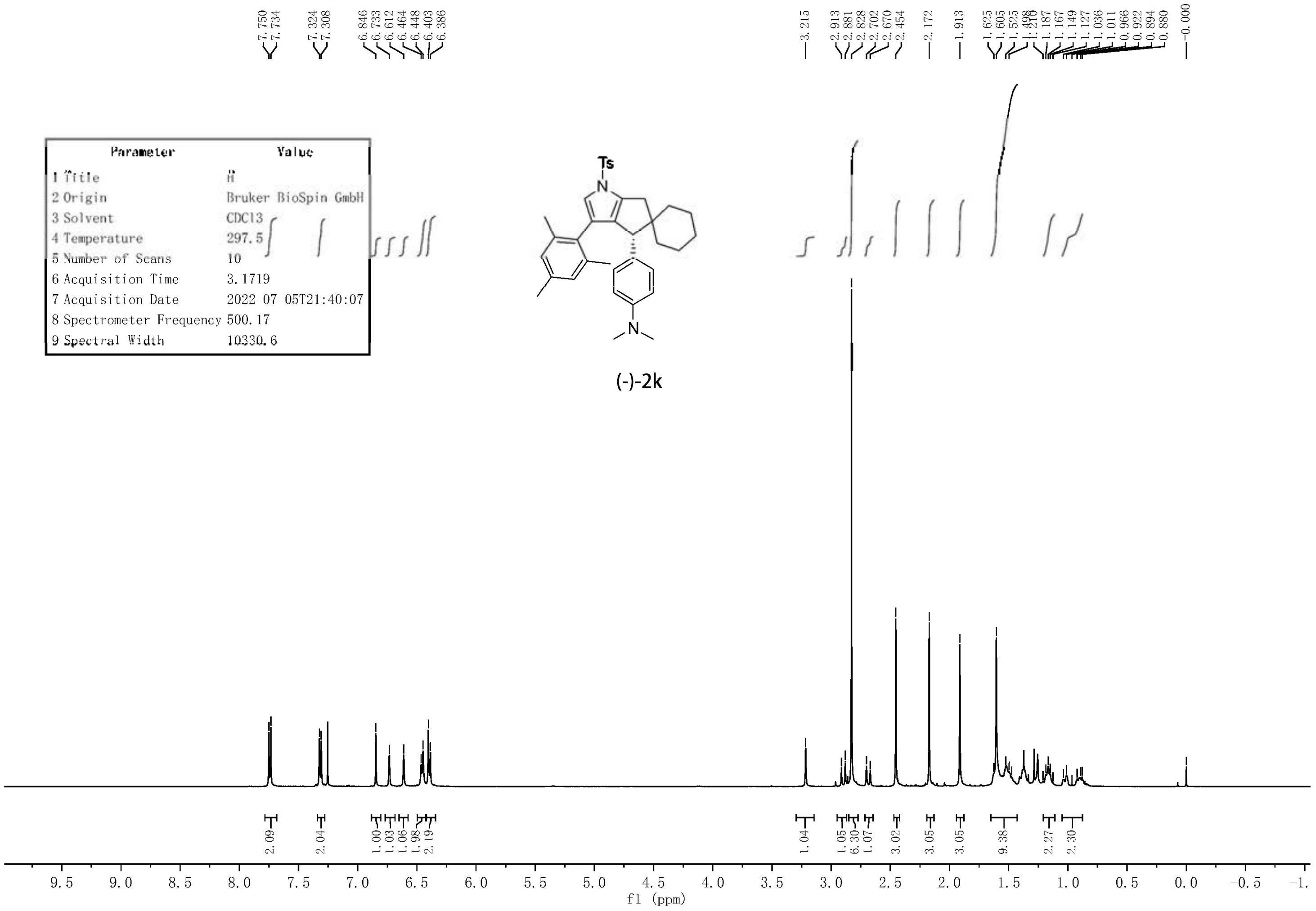

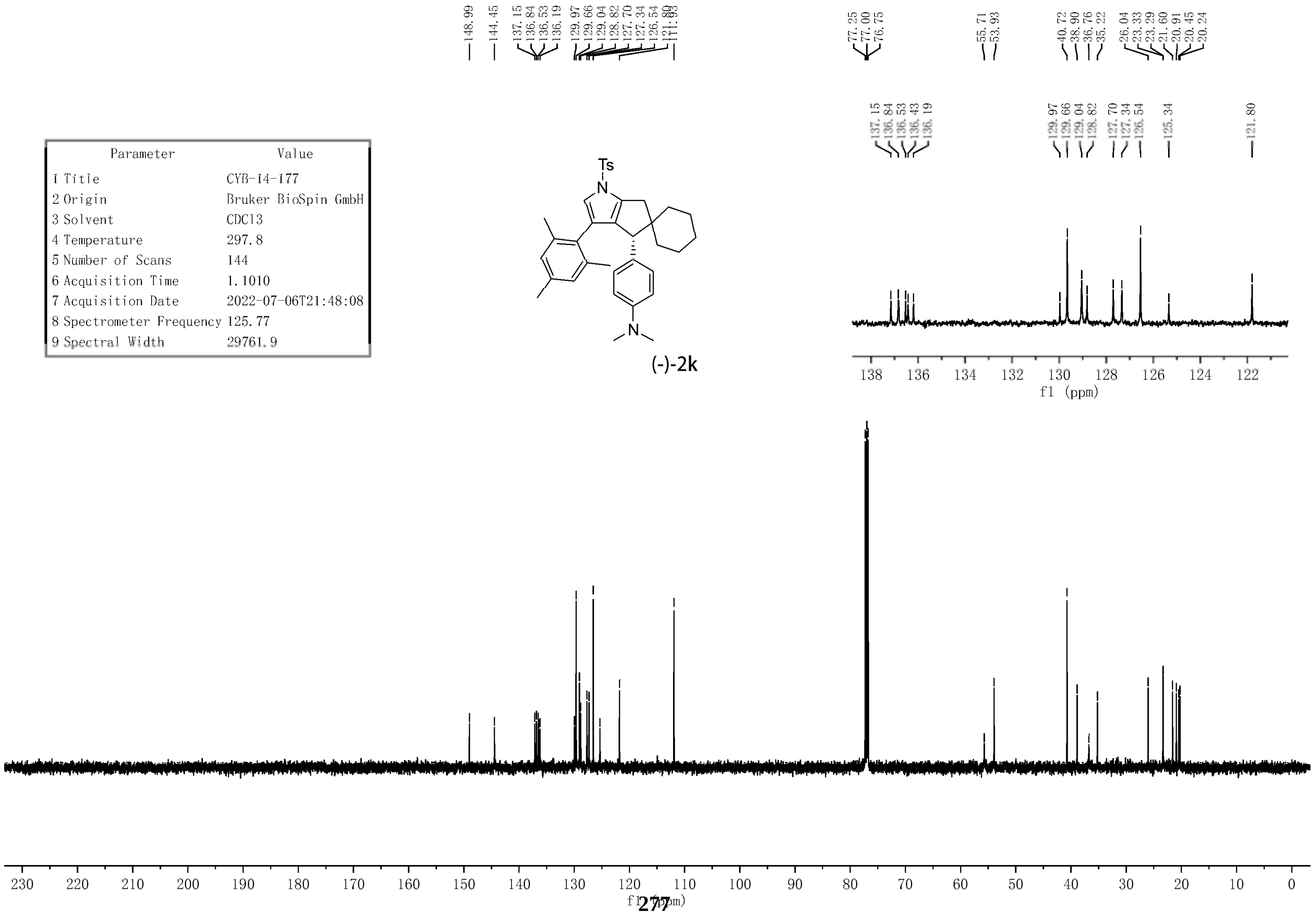

Supplementary Figure 177. ^{1}H and ^{13}C NMR spectra for (-)-2k

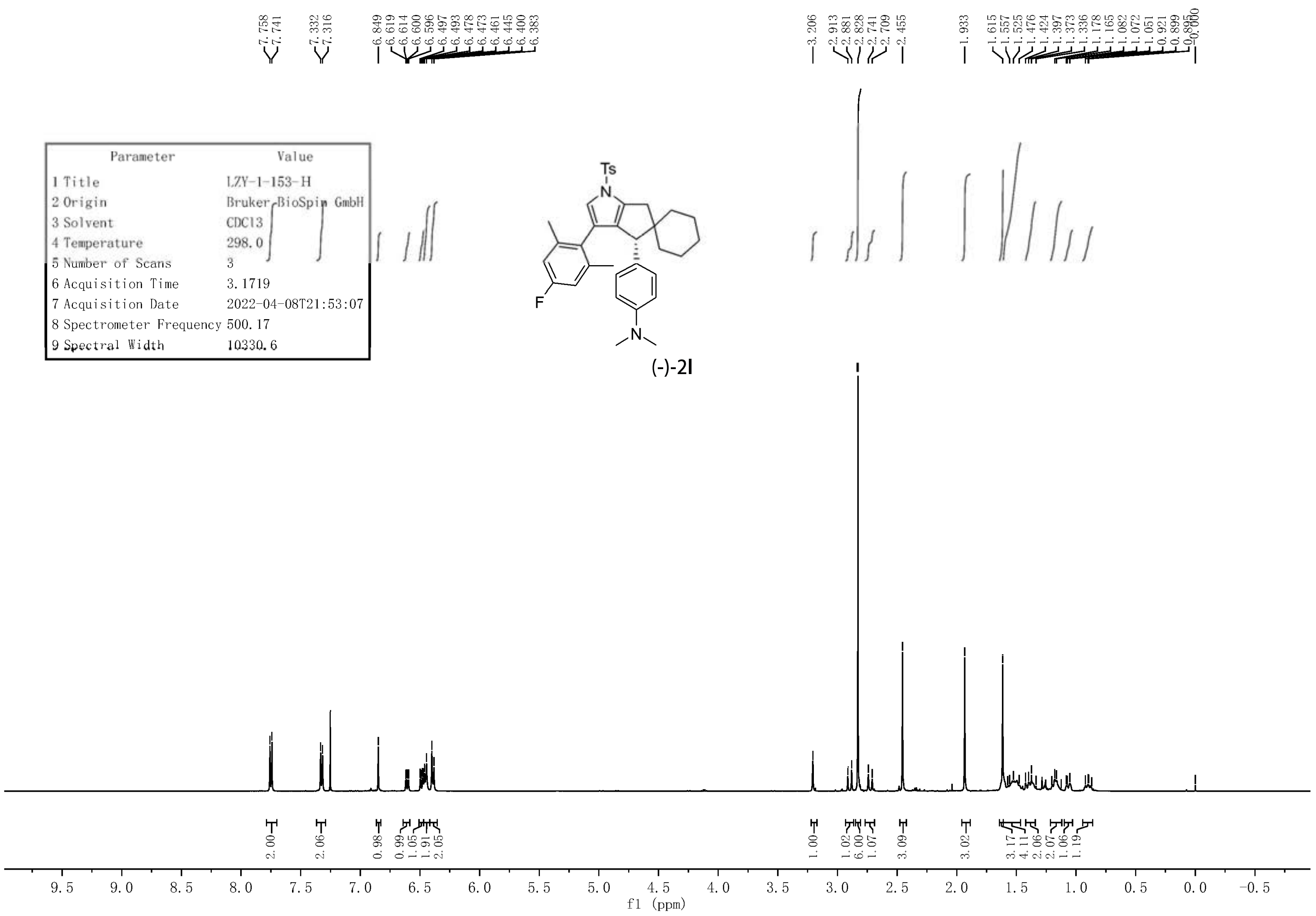

Parameter	Value
1 Title	LZY-1-153-H
2 Origin	Bruker BioSpin GmbH
3 Solvent	CDCl3
4 Temperature	298.0
5 Number of Scans	3
6 Acquisition Time	3.1719
7 Acquisition Date	2022-04-08T21:53:07
8 Spectrometer Frequency	500.17
9 Spectral Width	10330.6

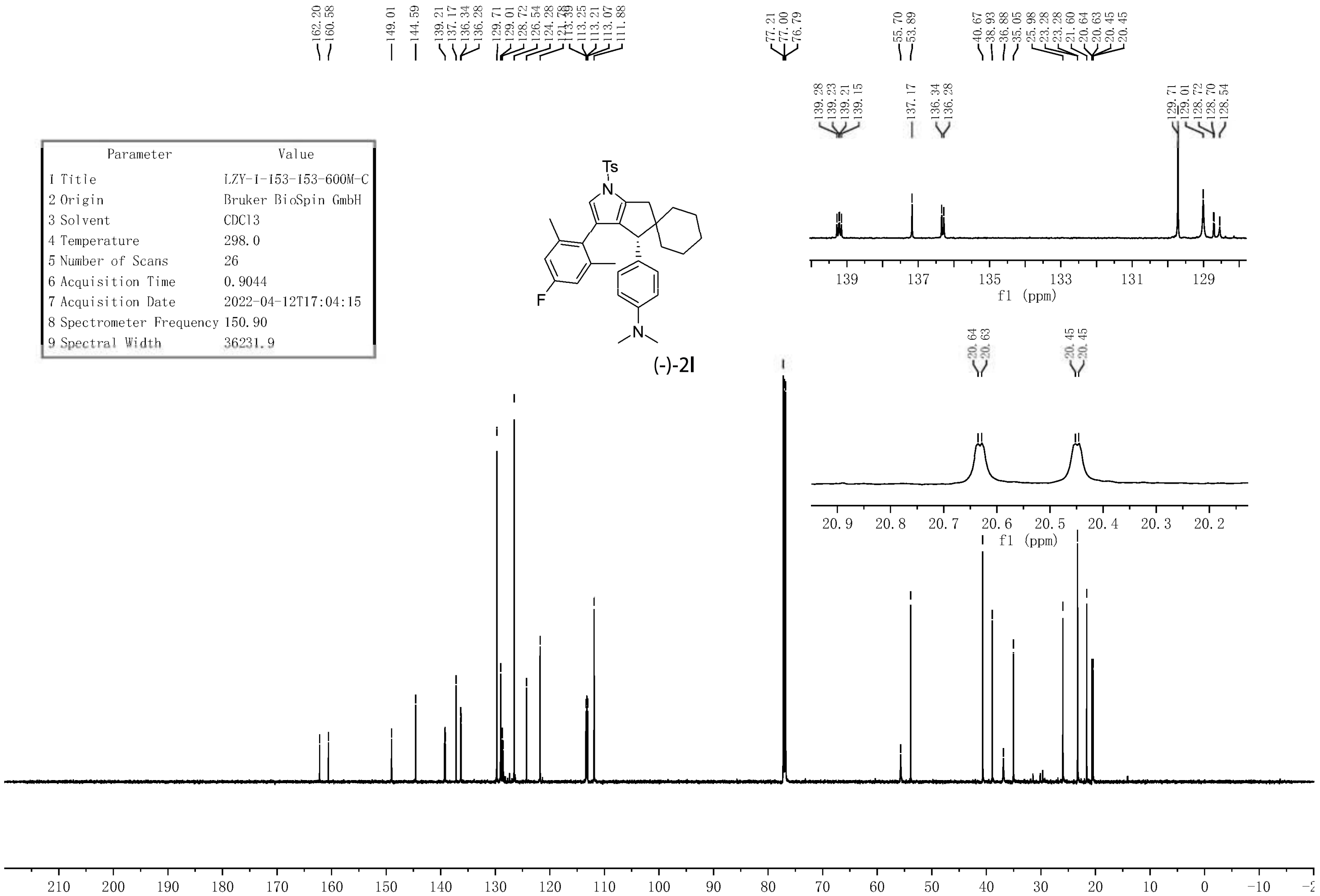

Parameter	Value
1 Title	LZY-1-153-153-600M-C
2 Origin	Bruker BioSpin GmbH
3 Solvent	CDCl3
4 Temperature	298.0
5 Number of Scans	26
6 Acquisition Time	0.9044
7 Acquisition Date	2022-04-12T17:04:15
8 Spectrometer Frequency	150.90
9 Spectral Width	36231.9

Supplementary Figure 178. ^{1}H and ^{13}C NMR spectra for (-)-2l

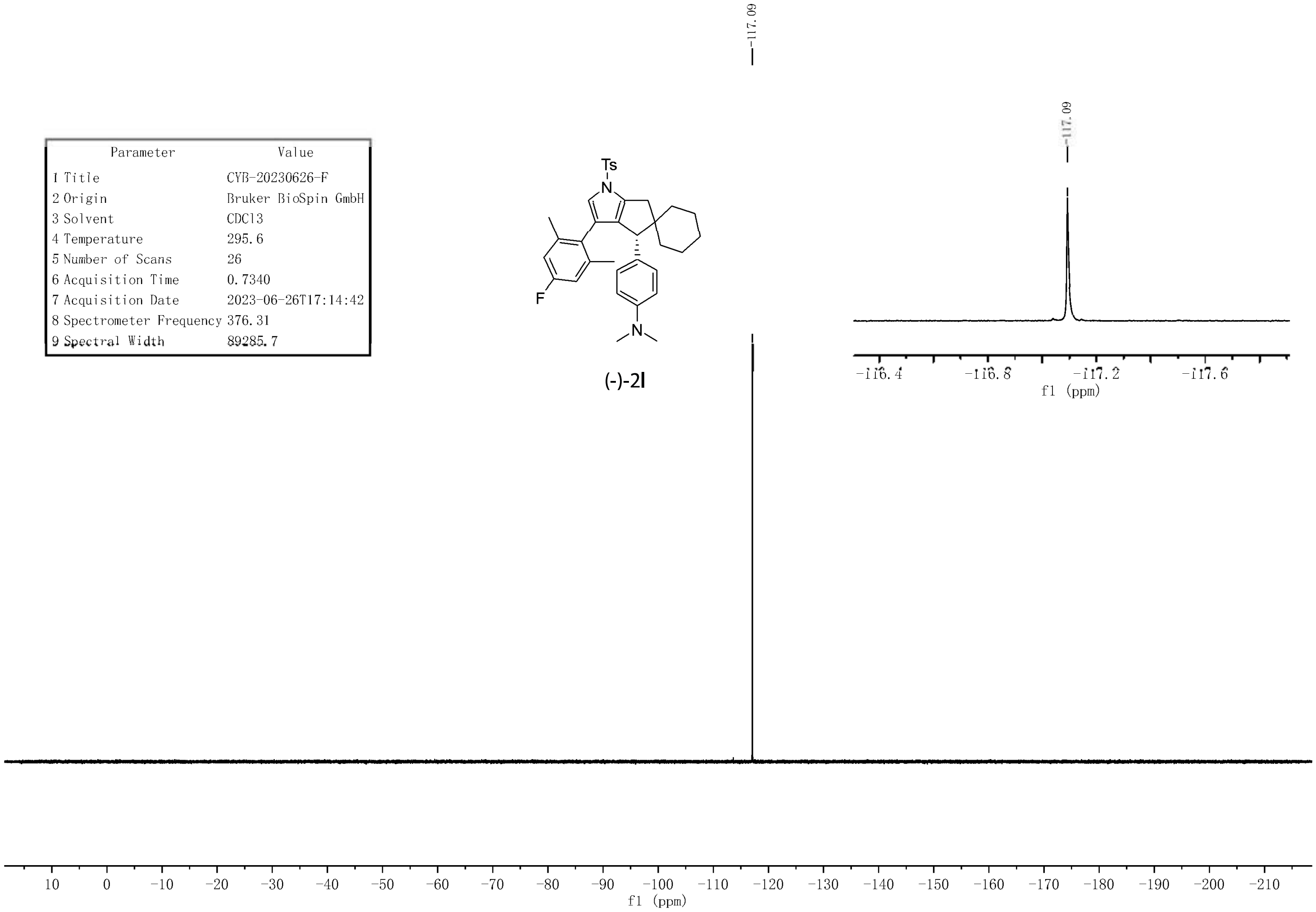

Supplementary Figure 179. ^{19}F NMR spectra for (-)-2l

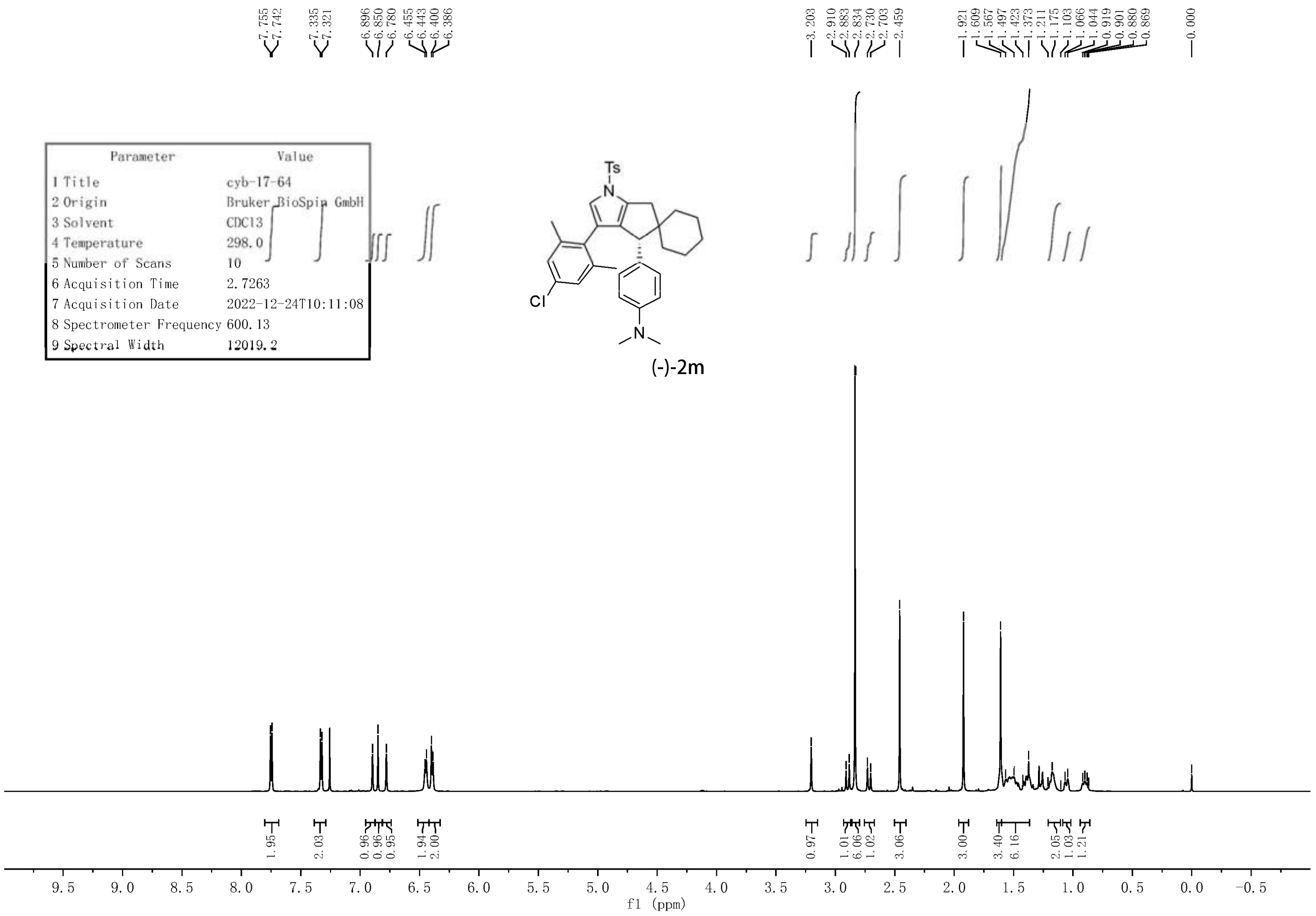

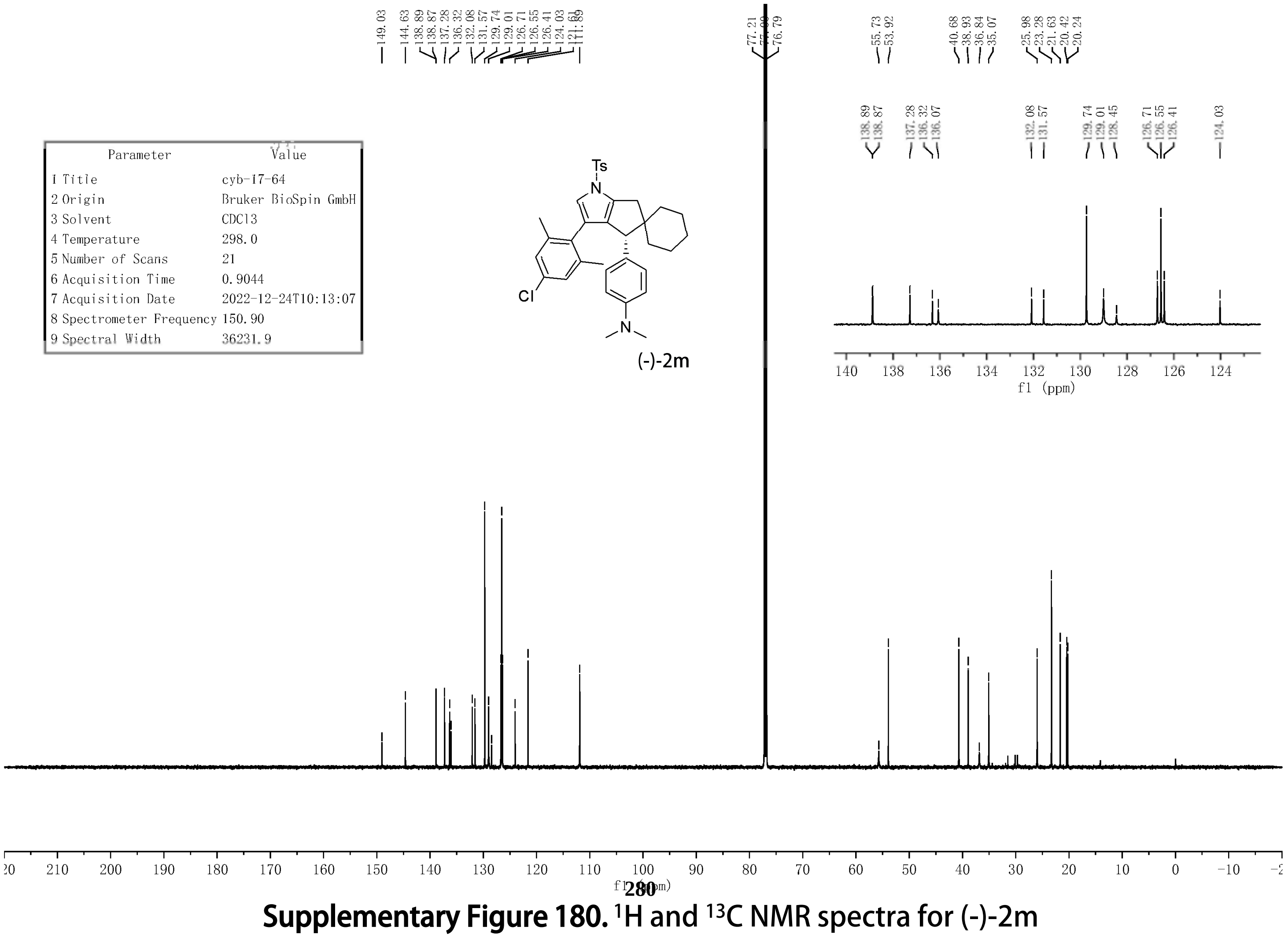

Supplementary Figure 180. ^{1}H and ^{13}C NMR spectra for (-)-2m

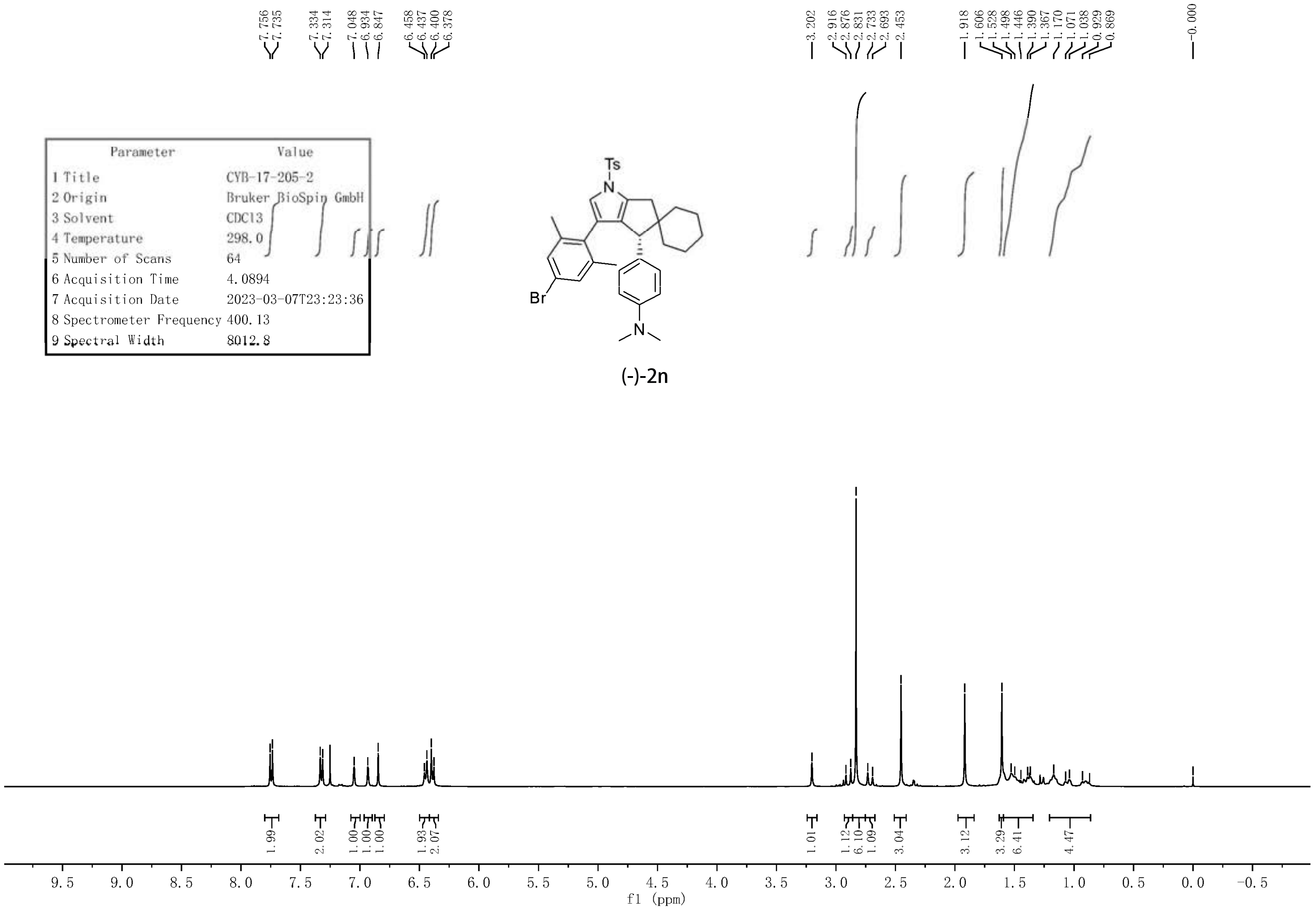

Parameter	Value
1 Title	CYB-17-205-2
2 Origin	Bruker BioSpin GmbH
3 Solvent	CDCl3
4 Temperature	298.0
5 Number of Scans	64
6 Acquisition Time	4.0894
7 Acquisition Date	2023-03-07T23:23:36
8 Spectrometer Frequency	400.13
9 Spectral Width	8012.8

(-)-2n

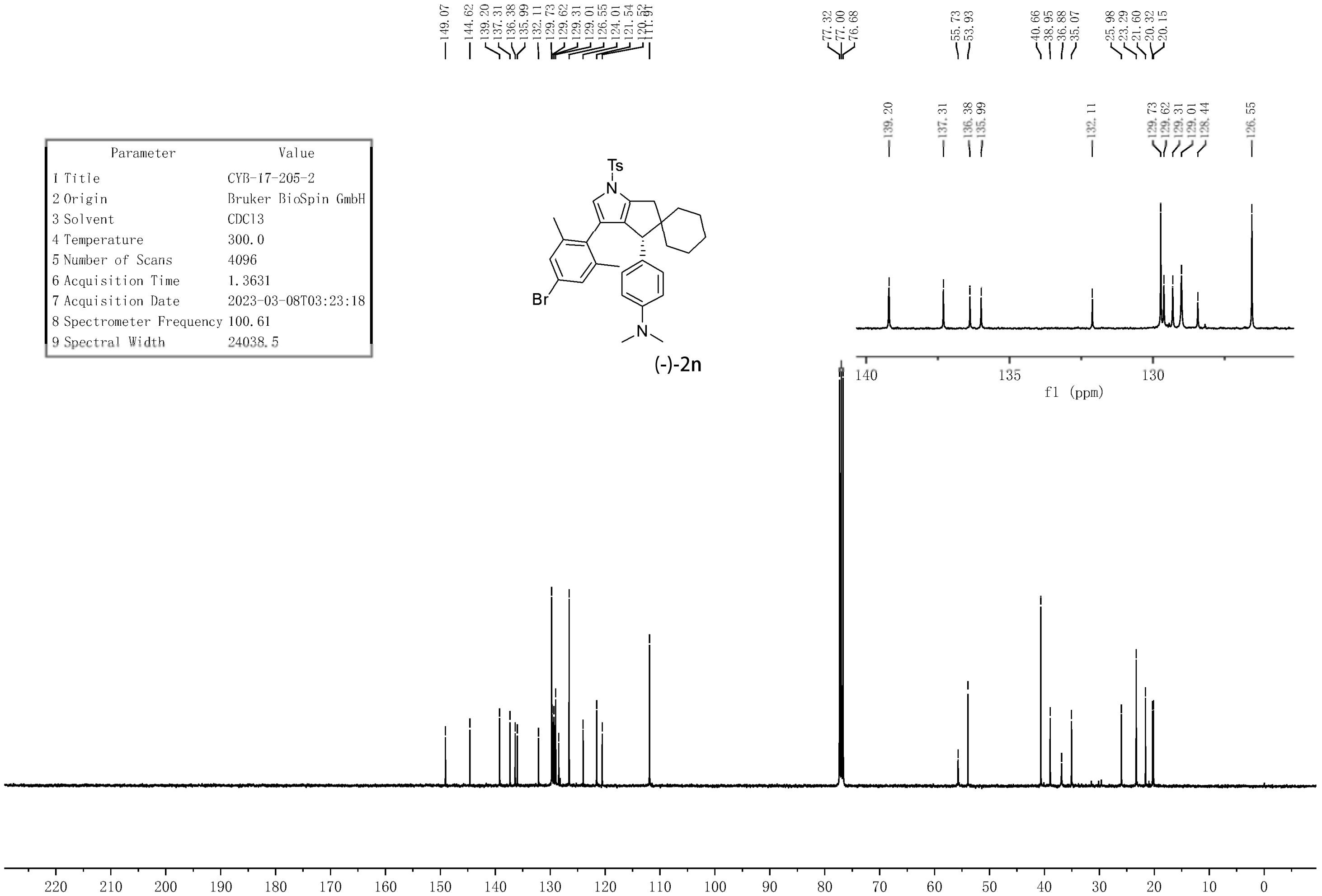

Parameter	Value
1 Title	CYB-17-205-2
2 Origin	Bruker BioSpin GmbH
3 Solvent	CDCl3
4 Temperature	300.0
5 Number of Scans	4096
6 Acquisition Time	1.3631
7 Acquisition Date	2023-03-08T03:23:18
8 Spectrometer Frequency	100.61
9 Spectral Width	24038.5

(-)-2n

Supplementary Figure 181. ^{1}H and ^{13}C NMR spectra for (-)-2n

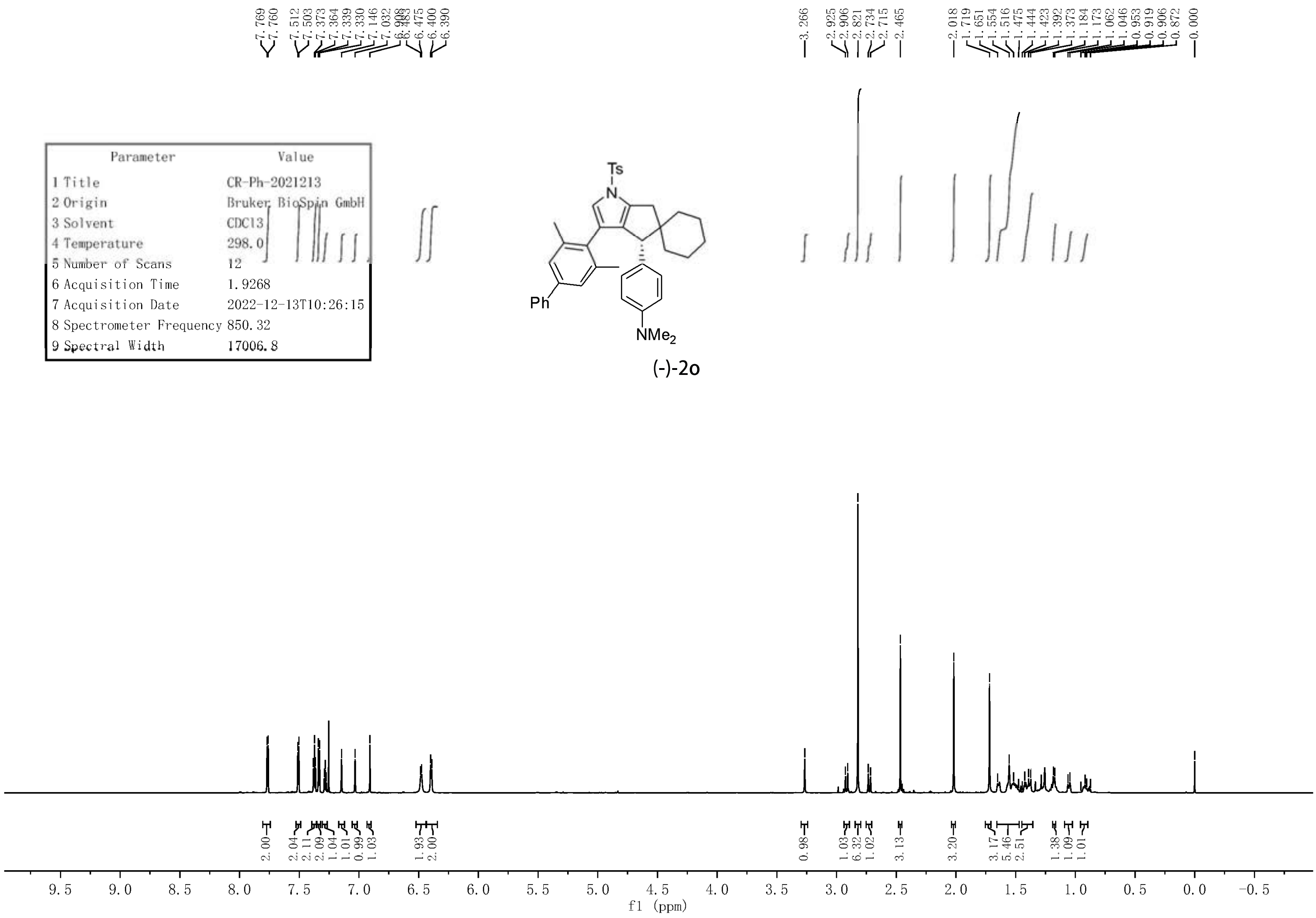

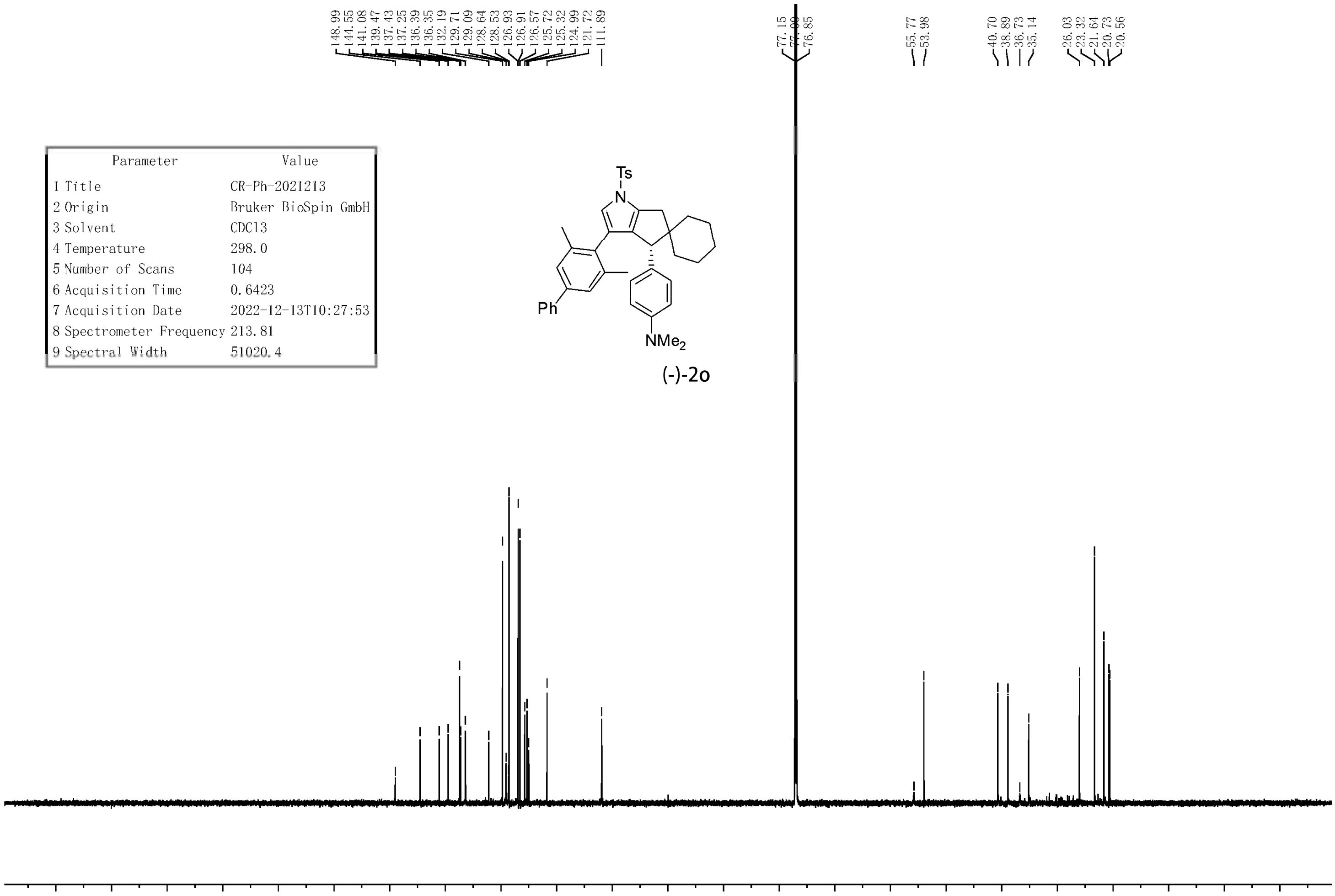

Supplementary Figure 182. ^{1}H and ^{13}C NMR spectra for (-)-2o

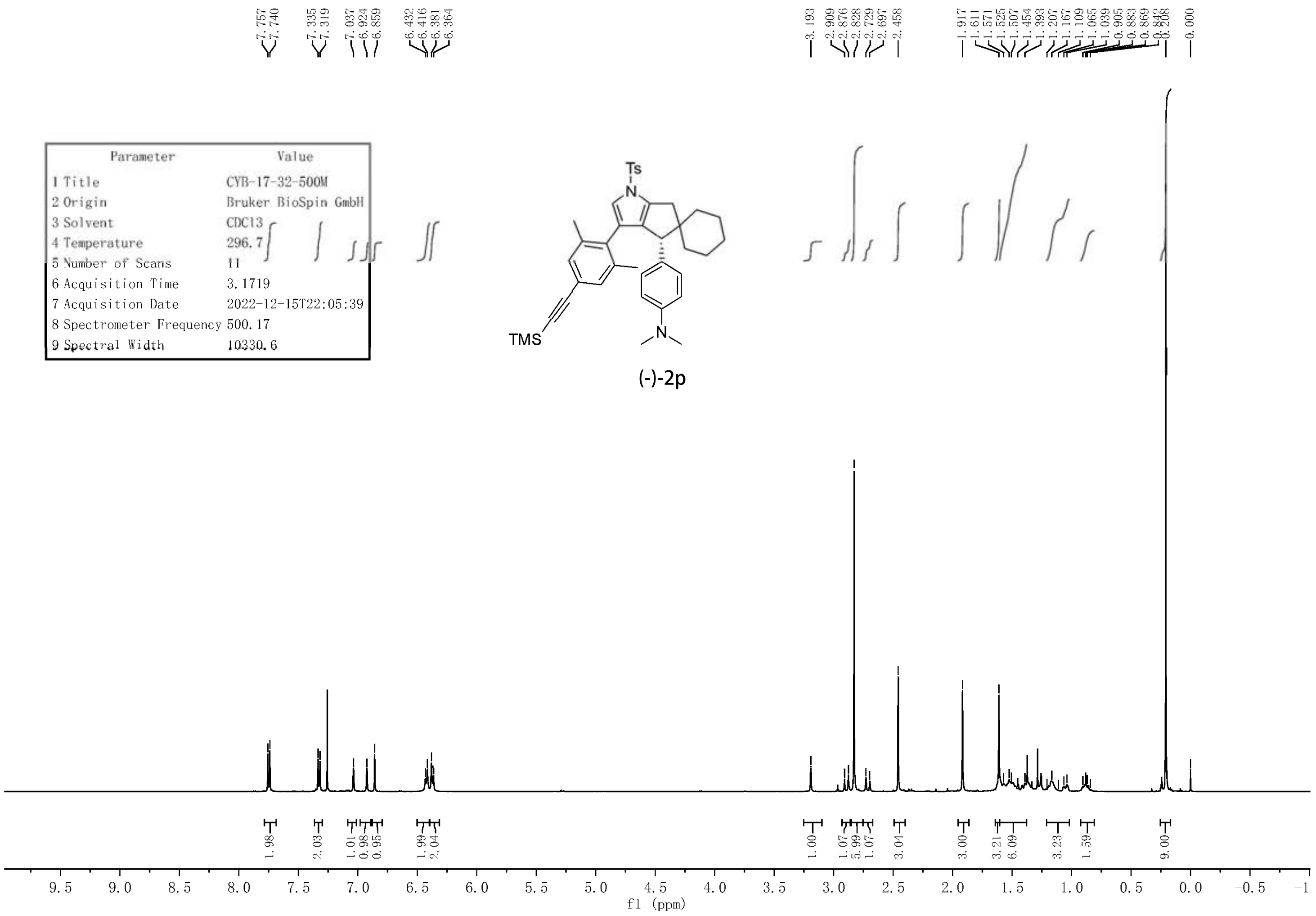

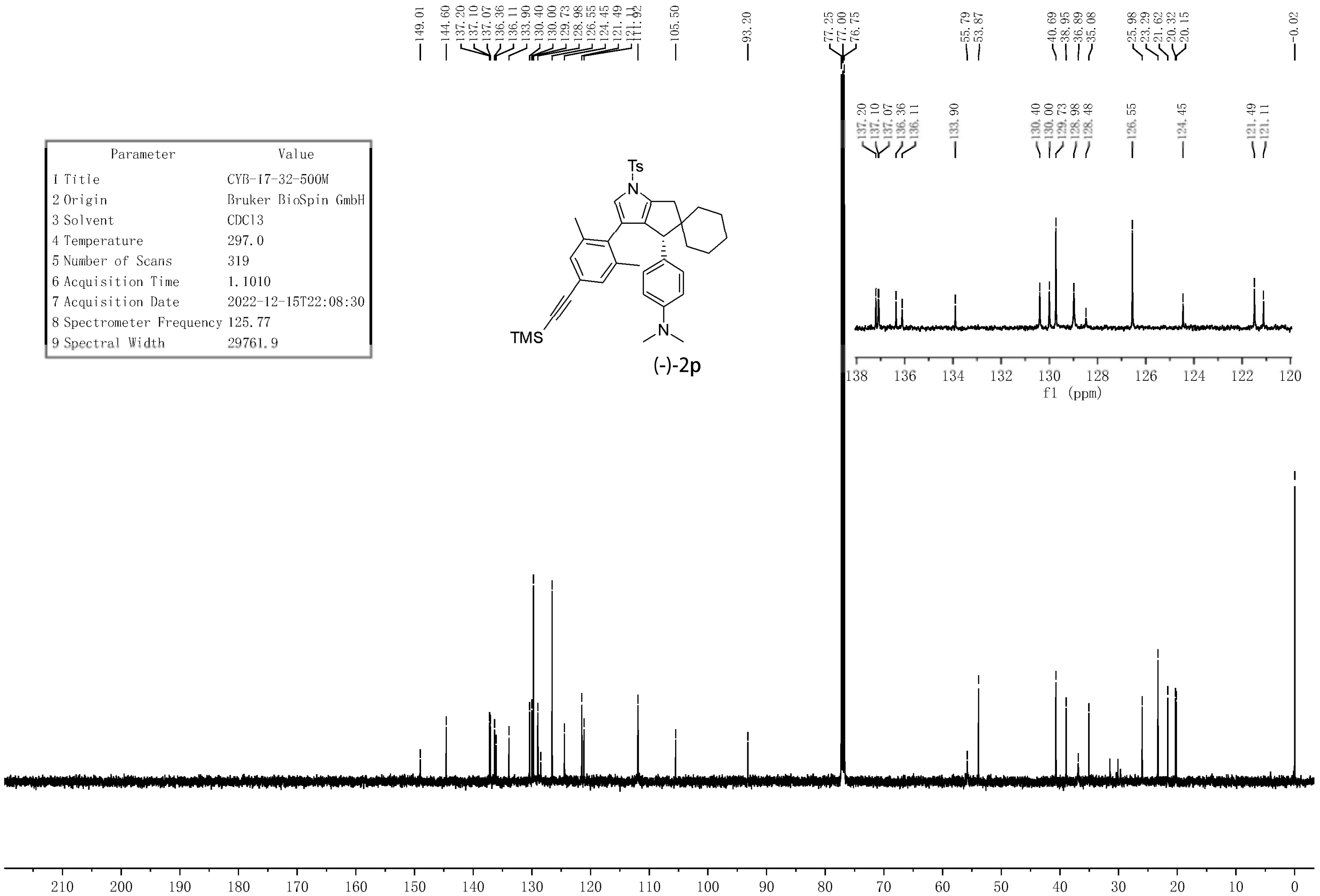

Supplementary Figure 183. ^{1}H and ^{13}C NMR spectra for (-)-2p

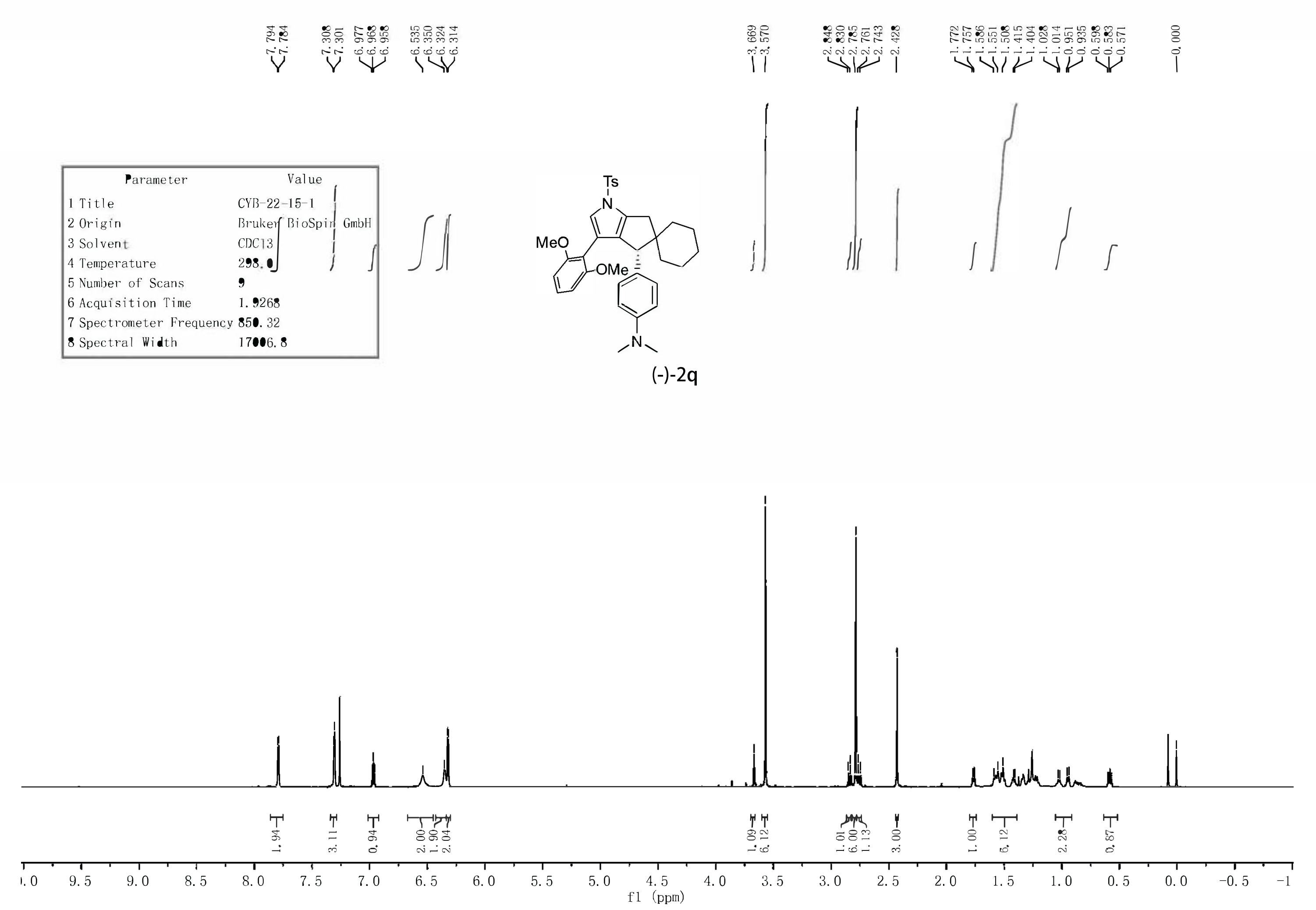

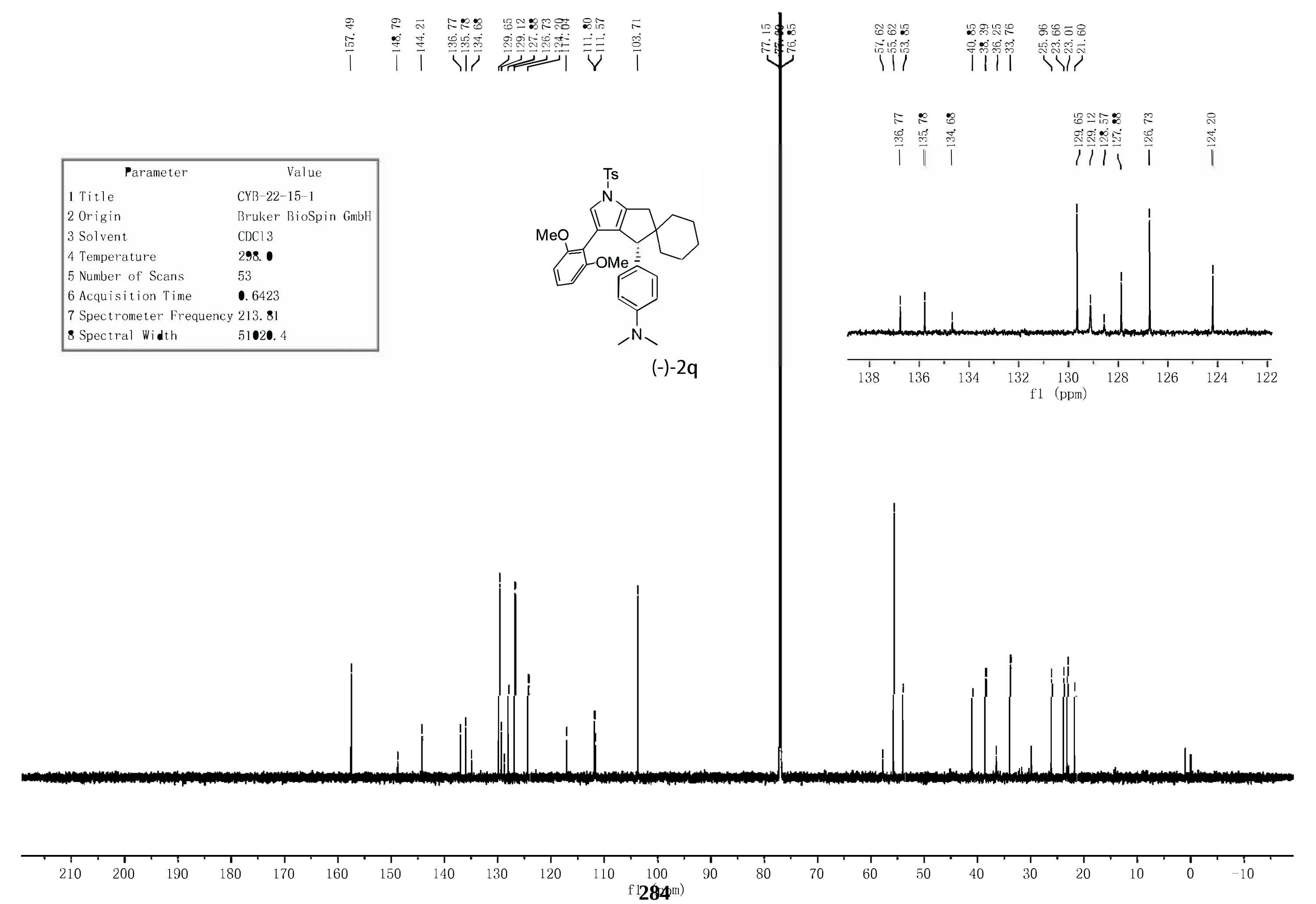

Supplementary Figure 184. ^{1}H and ^{13}C NMR spectra for (-)-2q

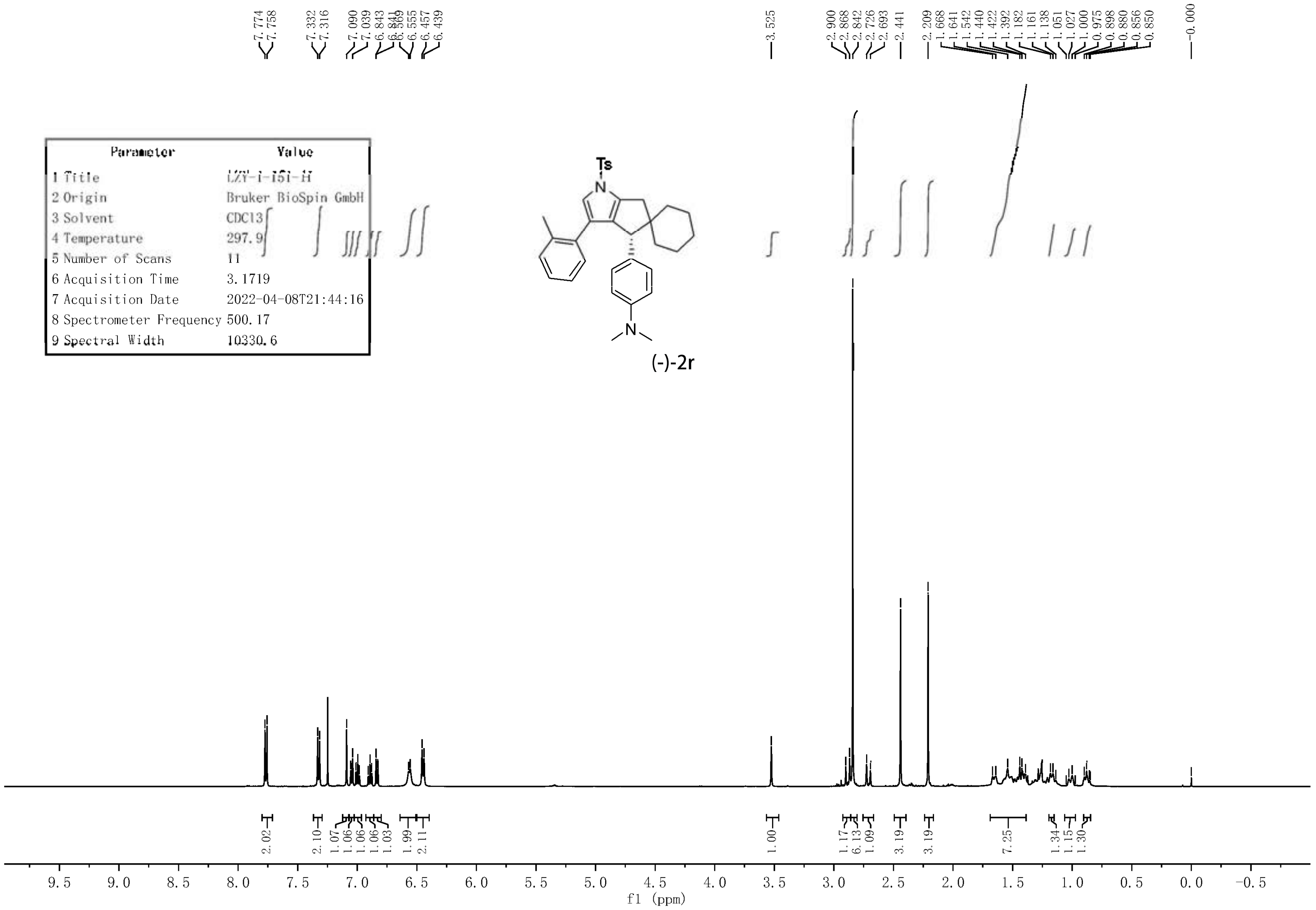

Parameter	Value
1 Title	LZY-1-151-H
2 Origin	Bruker BioSpin GmbH
3 Solvent	CDCl3
4 Temperature	297.9
5 Number of Scans	11
6 Acquisition Time	3.1719
7 Acquisition Date	2022-04-08T21:44:16
8 Spectrometer Frequency	500.17
9 Spectral Width	10330.6

(-)-2r

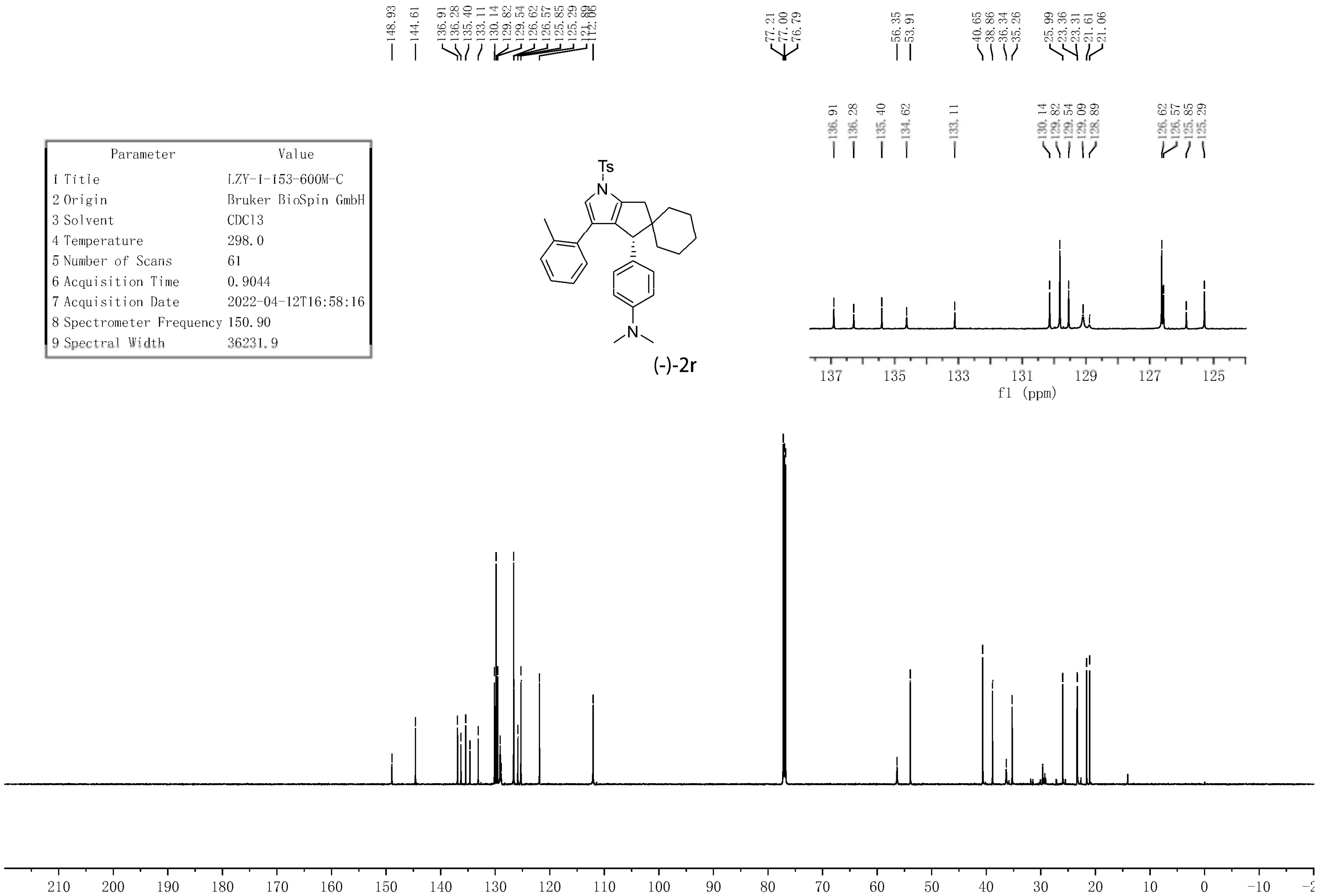

Parameter	Value
1 Title	LZY-1-153-600M-C
2 Origin	Bruker BioSpin GmbH
3 Solvent	CDCl3
4 Temperature	298.0
5 Number of Scans	61
6 Acquisition Time	0.9044
7 Acquisition Date	2022-04-12T16:58:16
8 Spectrometer Frequency	150.90
9 Spectral Width	36231.9

(-)-2r

Supplementary Figure 185. ^{1}H and ^{13}C NMR spectra for (-)-2r

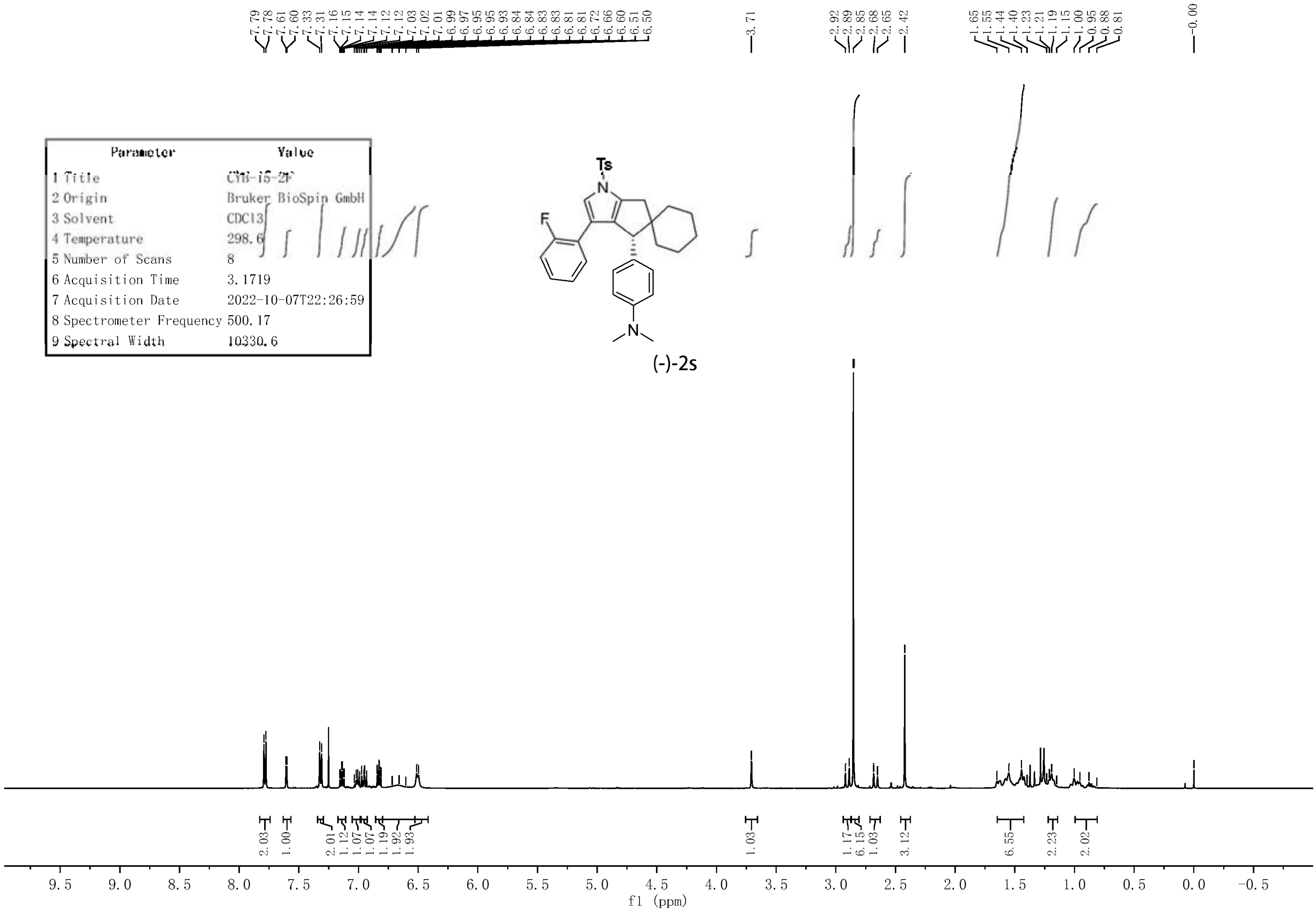

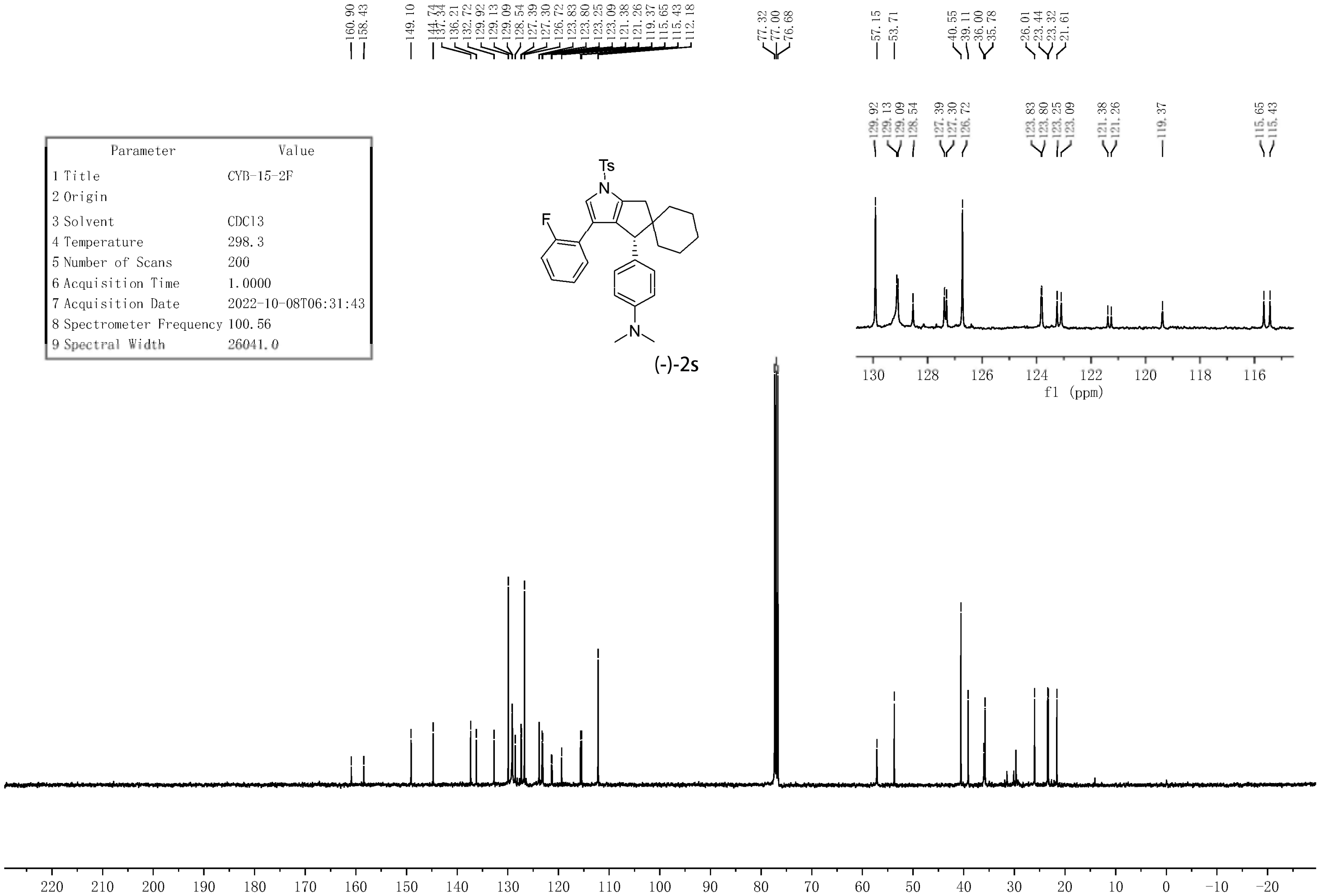

Supplementary Figure 186. ^{1}H and ^{13}C NMR spectra for (-)-2s

Parameter	Value
1 Title	CYB-15-2F
2 Origin	
3 Solvent	CDCl3
4 Temperature	298.4
5 Number of Scans	50
6 Acquisition Time	1.0000
7 Acquisition Date	2022-10-08T06:35:50
8 Spectrometer Frequency	376.28
9 Spectral Width	96153.0

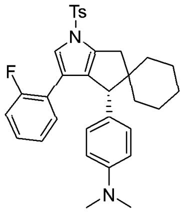

(-)-2s

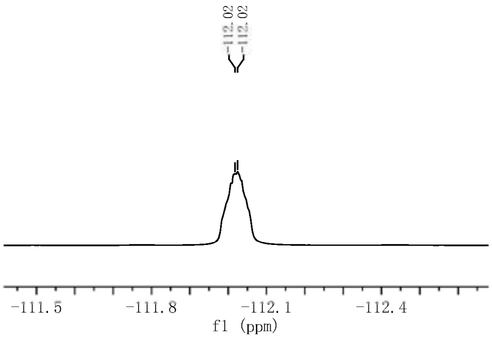

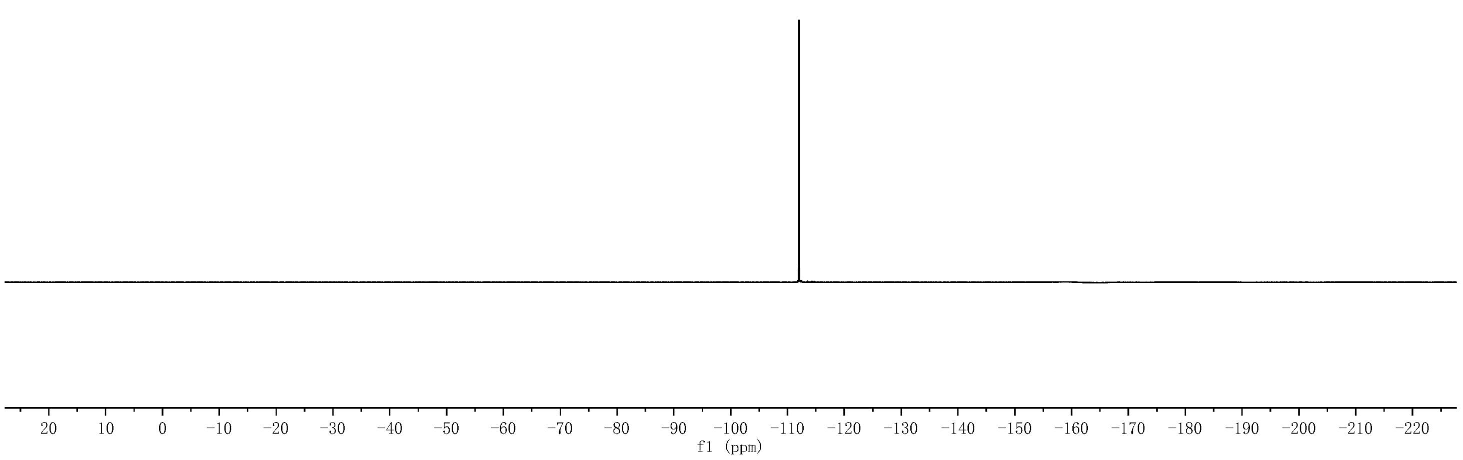

Supplementary Figure 187. ^{19}F NMR spectra for (-)-2s

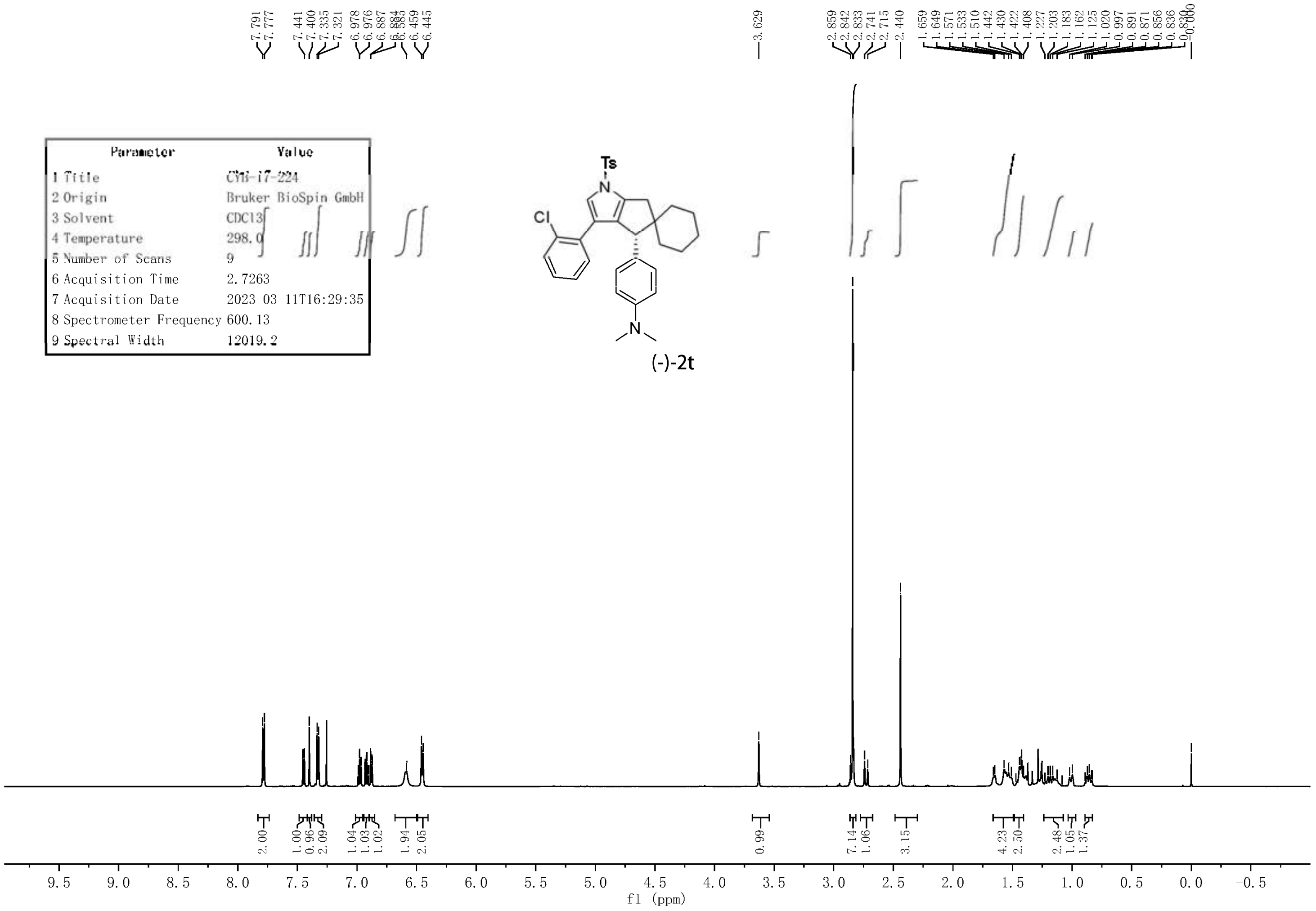

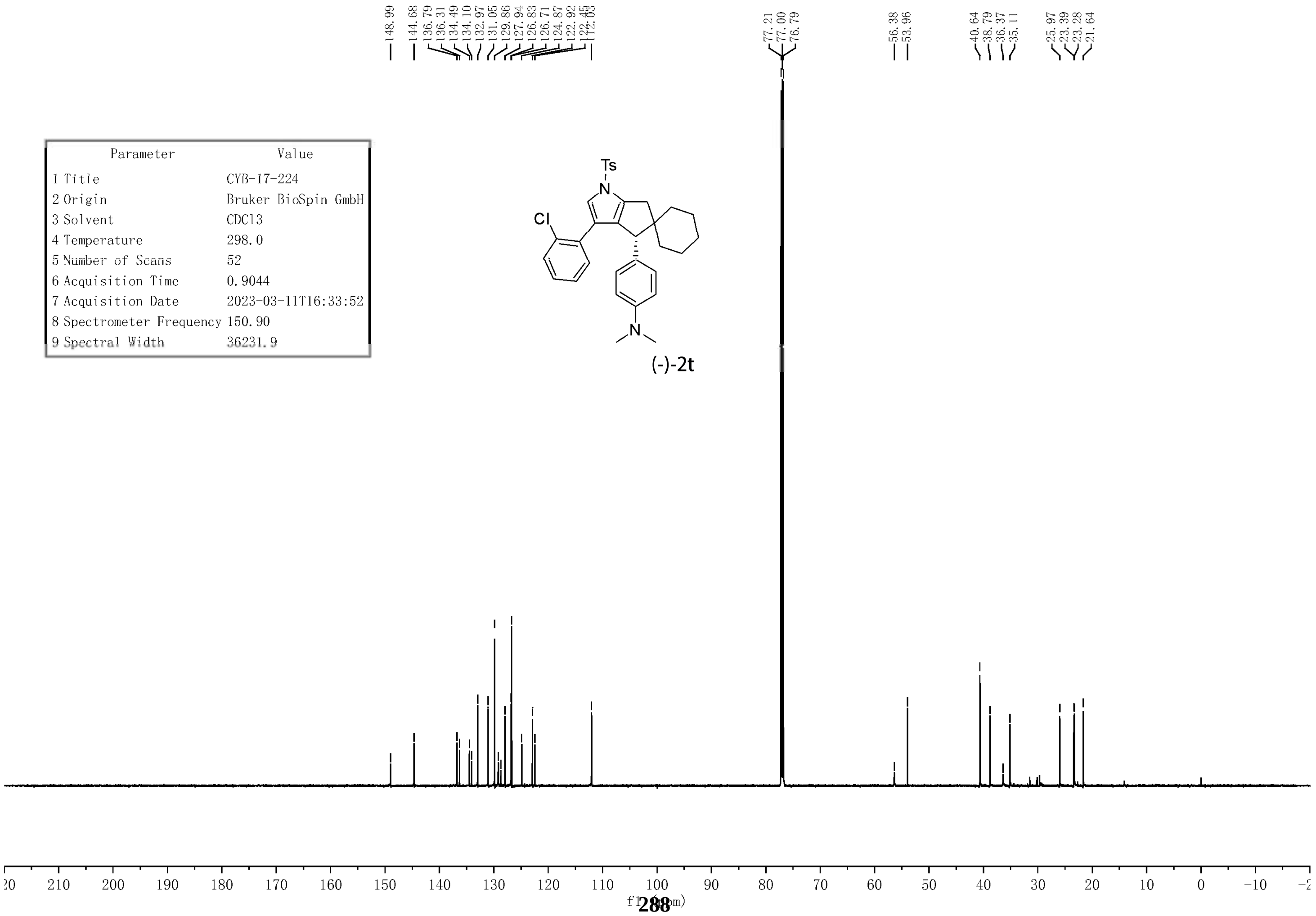

Supplementary Figure 188. ^{1}H and ^{13}C NMR spectra for (-)-2t

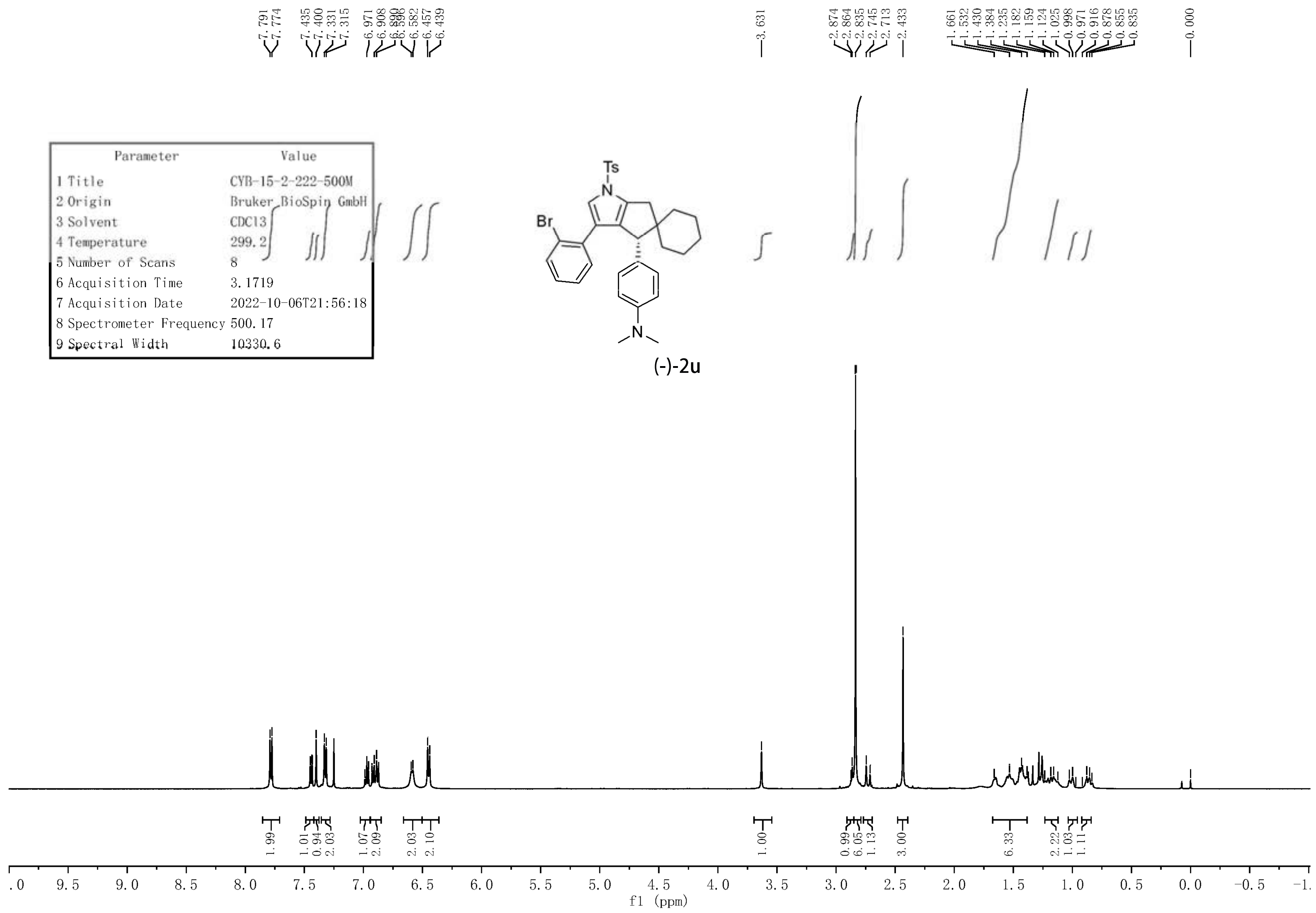

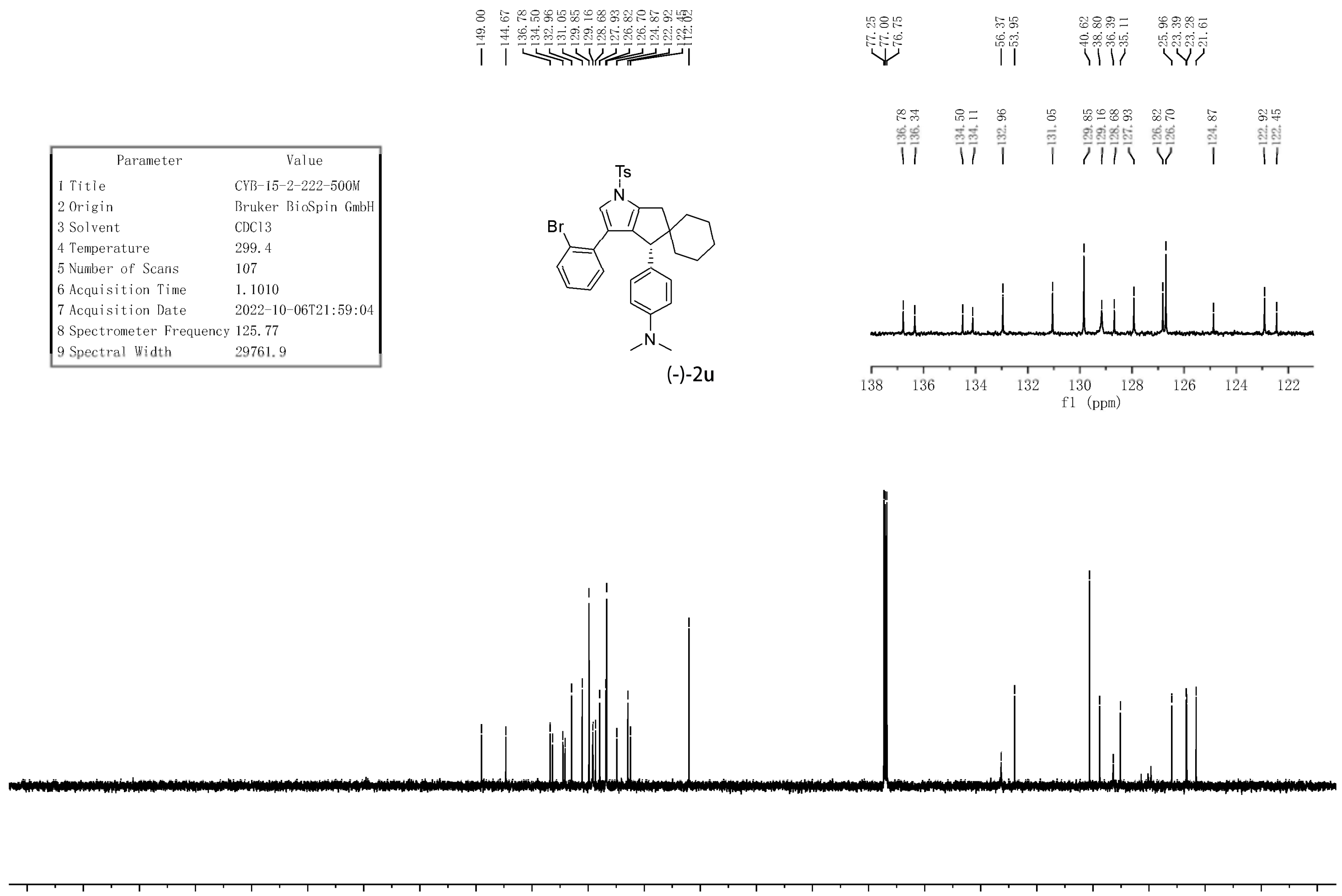

Supplementary Figure 189. ^{1}H and ^{13}C NMR spectra for (-)-2u

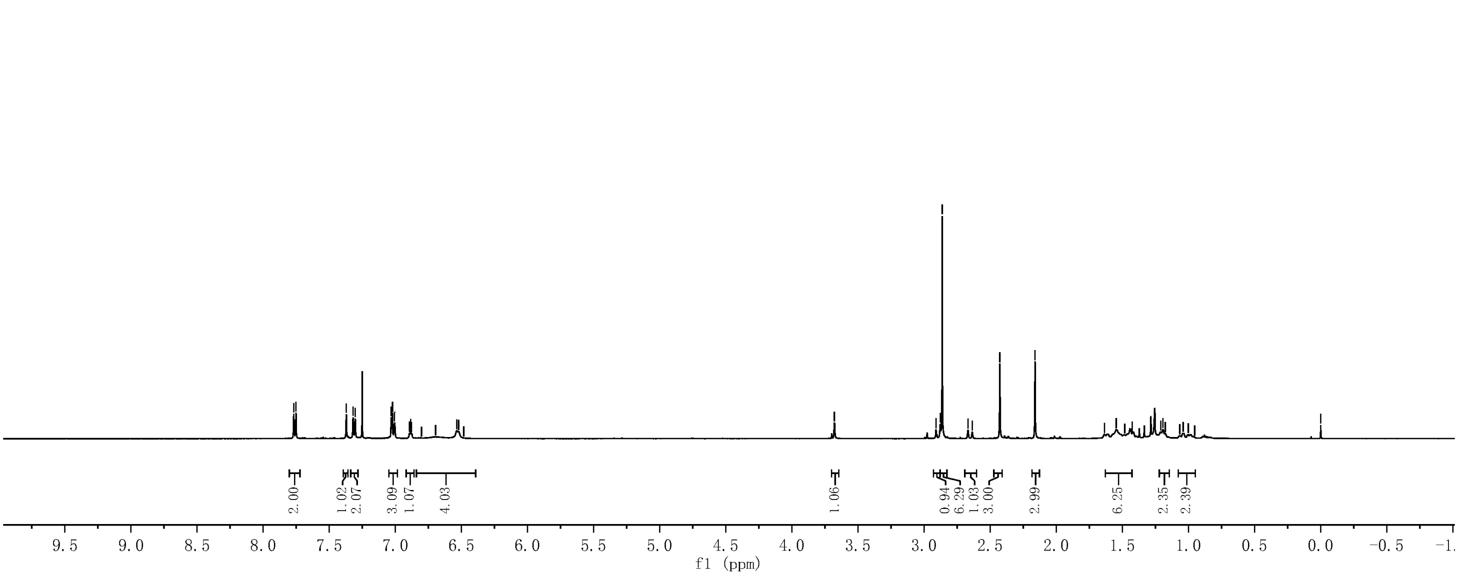

Parameter	Value
1 Title	CYB-16-8
2 Origin	Bruker BioSpin GmbH
3 Solvent	CDCl3
4 Temperature	299.3
5 Number of Scans	13
6 Acquisition Time	3.1719
7 Acquisition Date	2022-10-11T21:38:42
8 Spectrometer Frequency	500.17
9 Spectral Width	10330.6

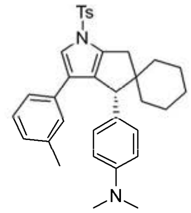

(-)-2v

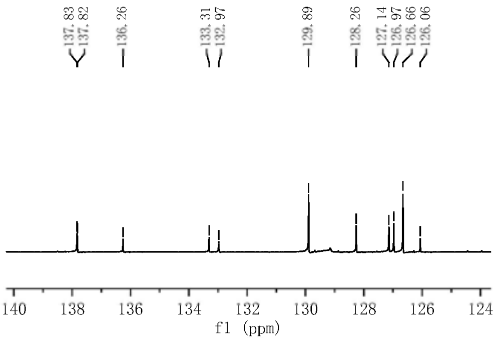

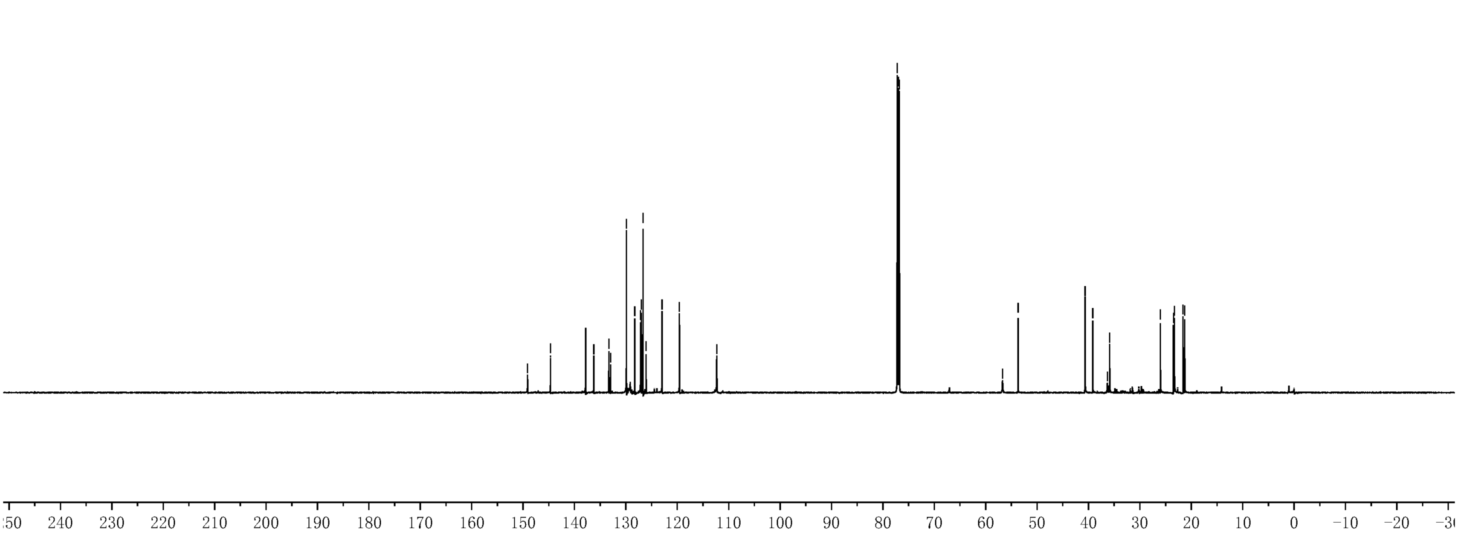

Parameter	Value
1 Title	CYB-16-8
2 Origin	Bruker BioSpin GmbH
3 Solvent	CDCl3
4 Temperature	298.0
5 Number of Scans	100
6 Acquisition Time	0.7690
7 Acquisition Date	2022-10-16T13:55:31
8 Spectrometer Frequency	150.90
9 Spectral Width	42613.6

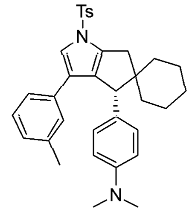

(-)-2v

Supplementary Figure 190. ^{1}H and ^{13}C NMR spectra for (-)-2v

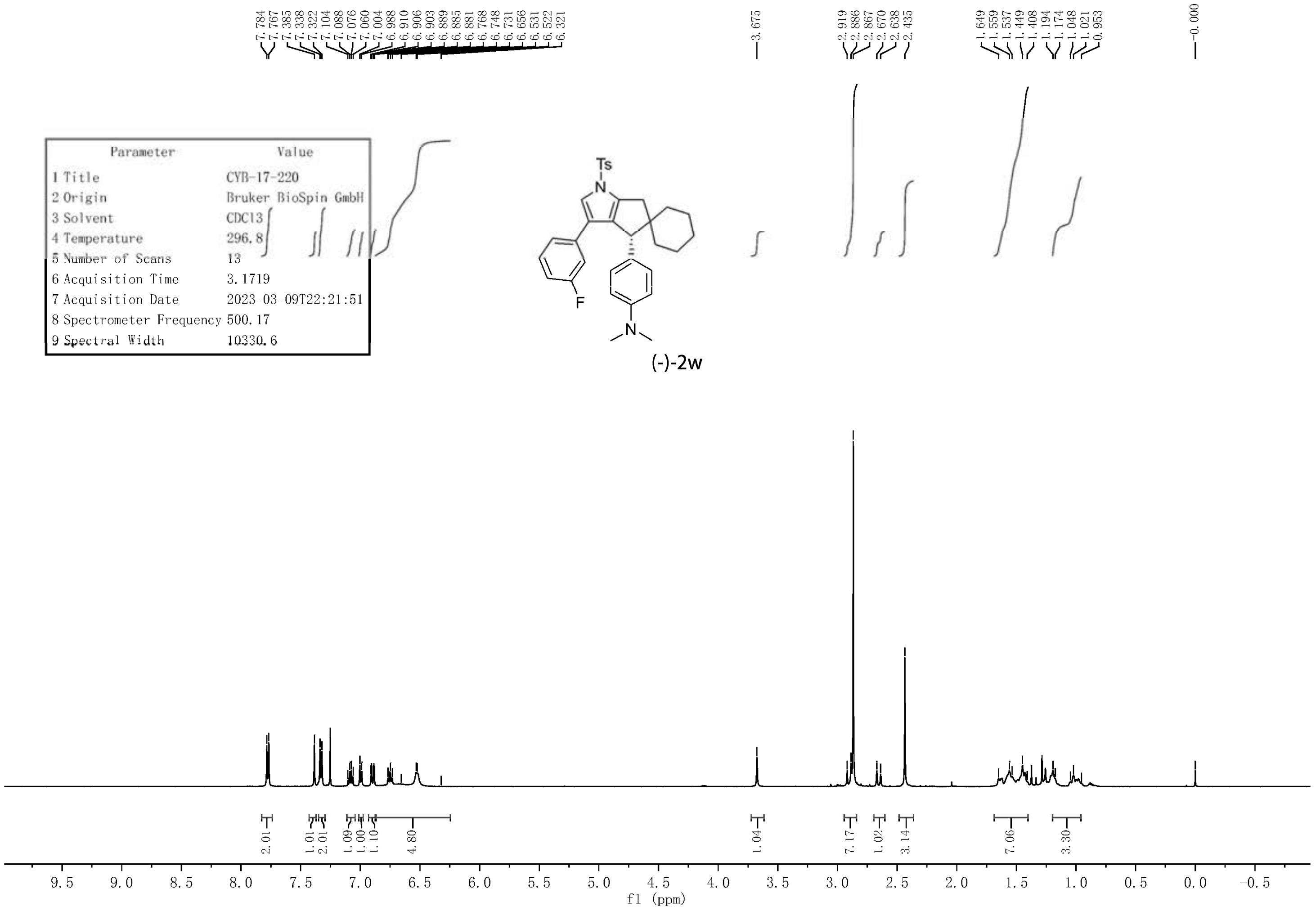

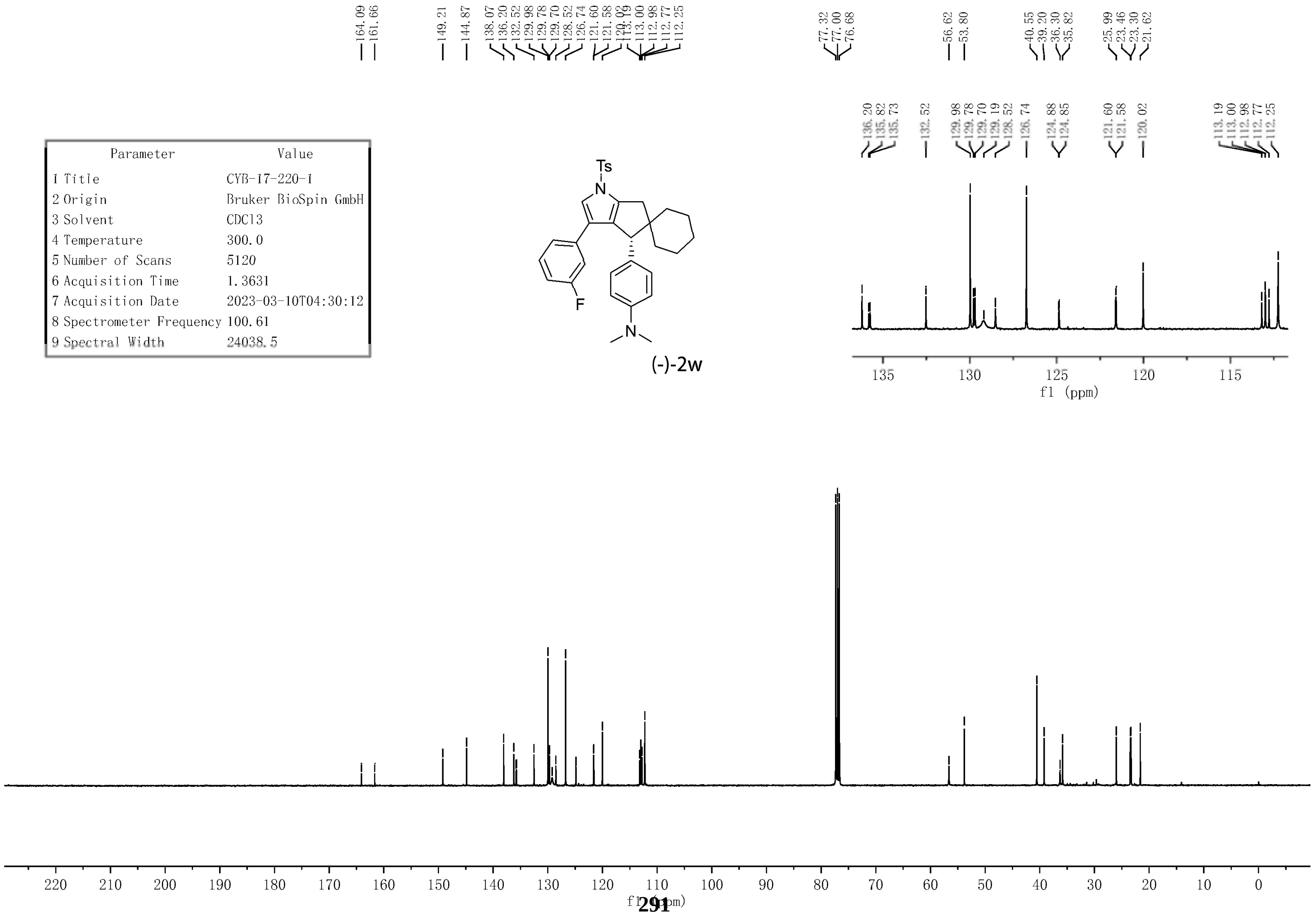

Supplementary Figure 191. ^{1}H and ^{13}C NMR spectra for (-)-2w

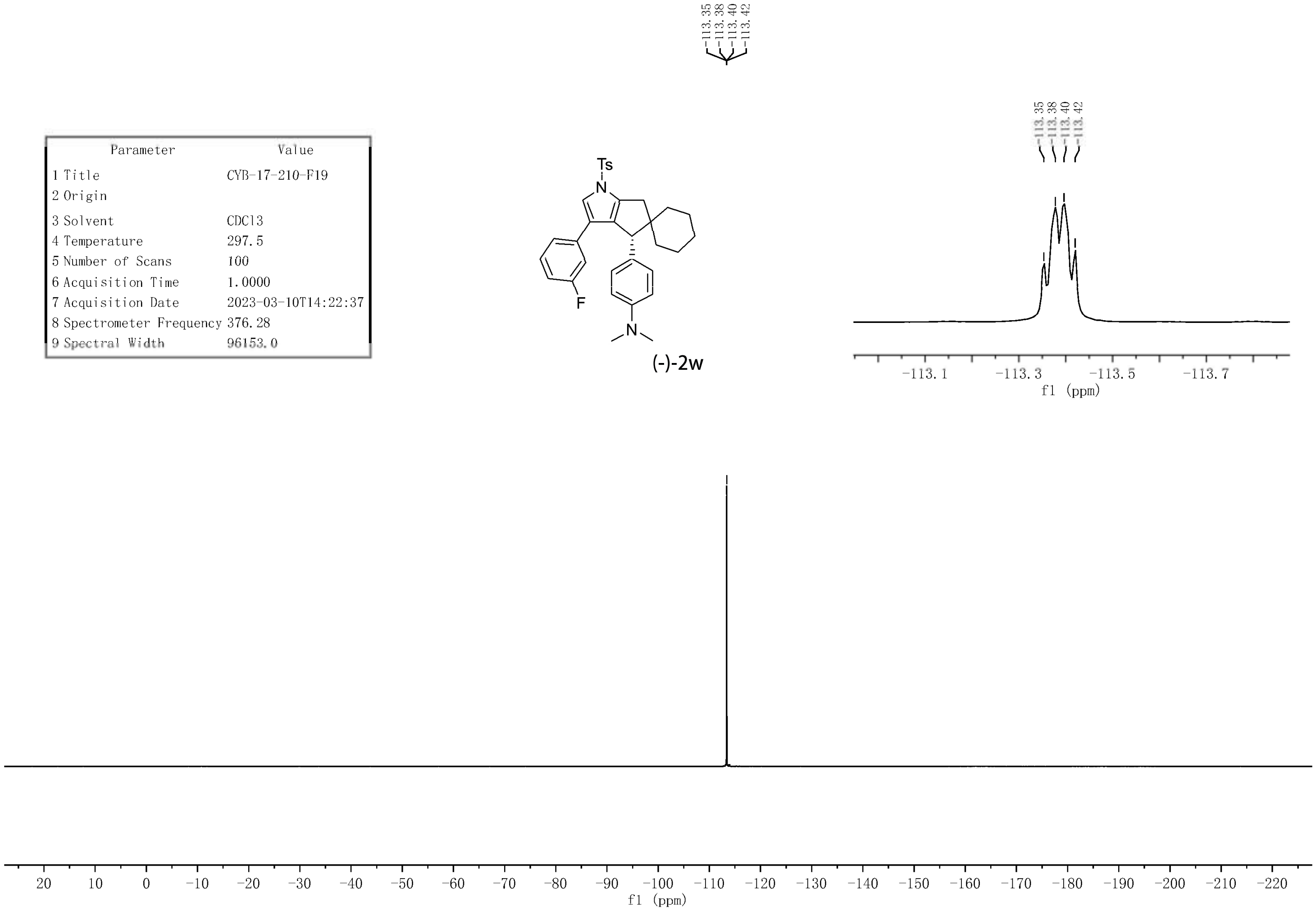

Supplementary Figure 192. ^{19}F NMR spectra for (-)-2w

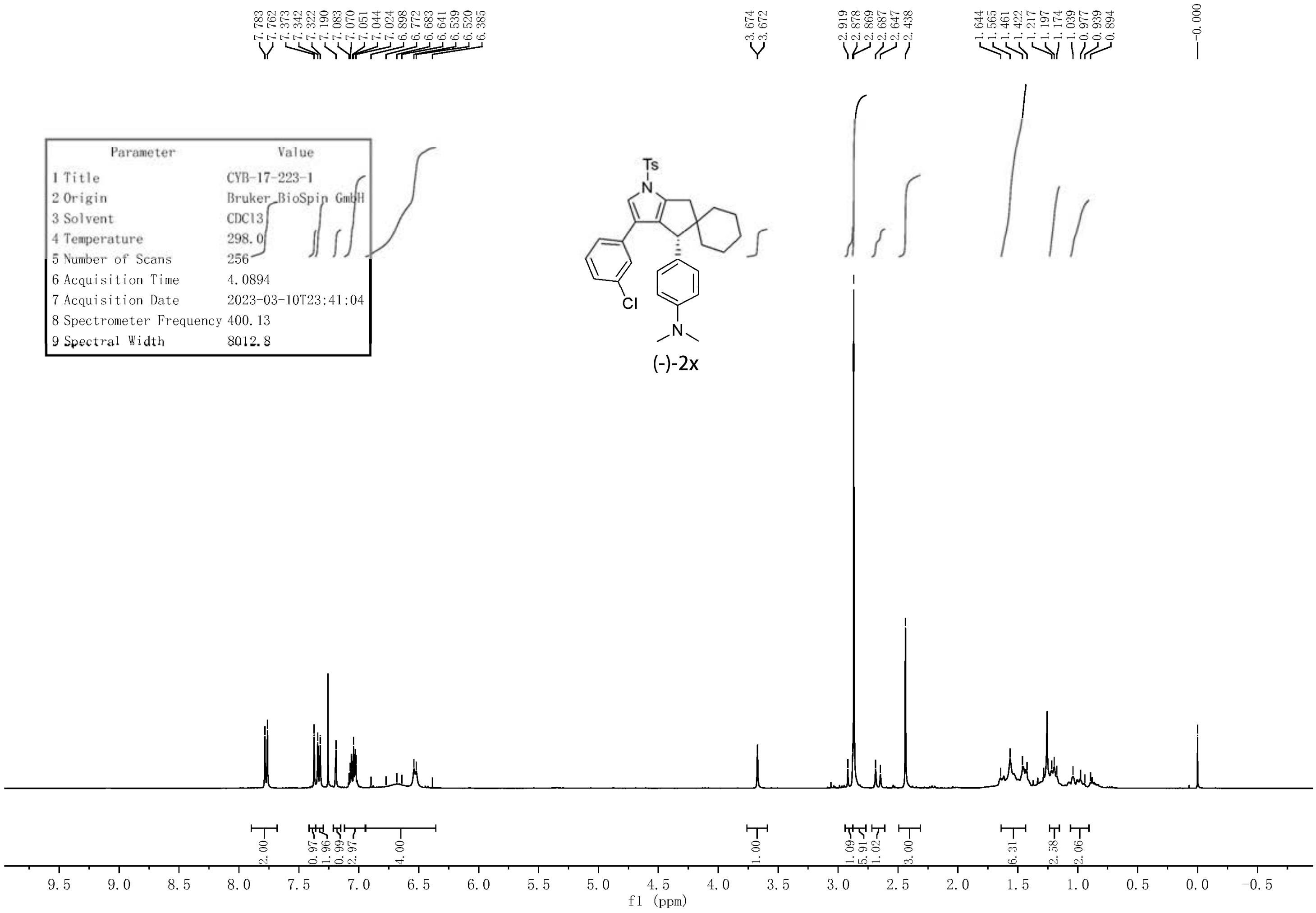

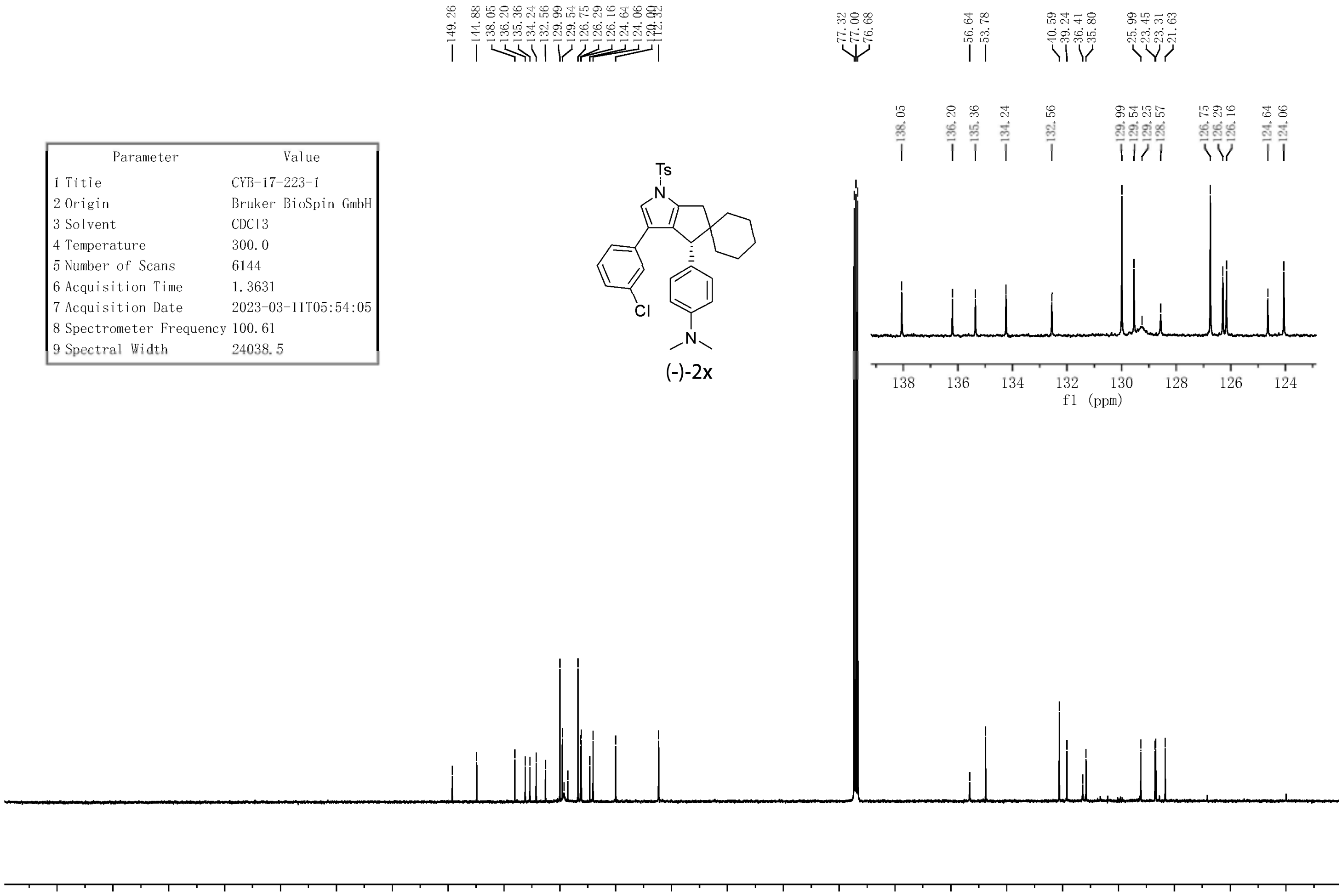

Supplementary Figure 193. ^{1}H and ^{13}C NMR spectra for (-)-2x

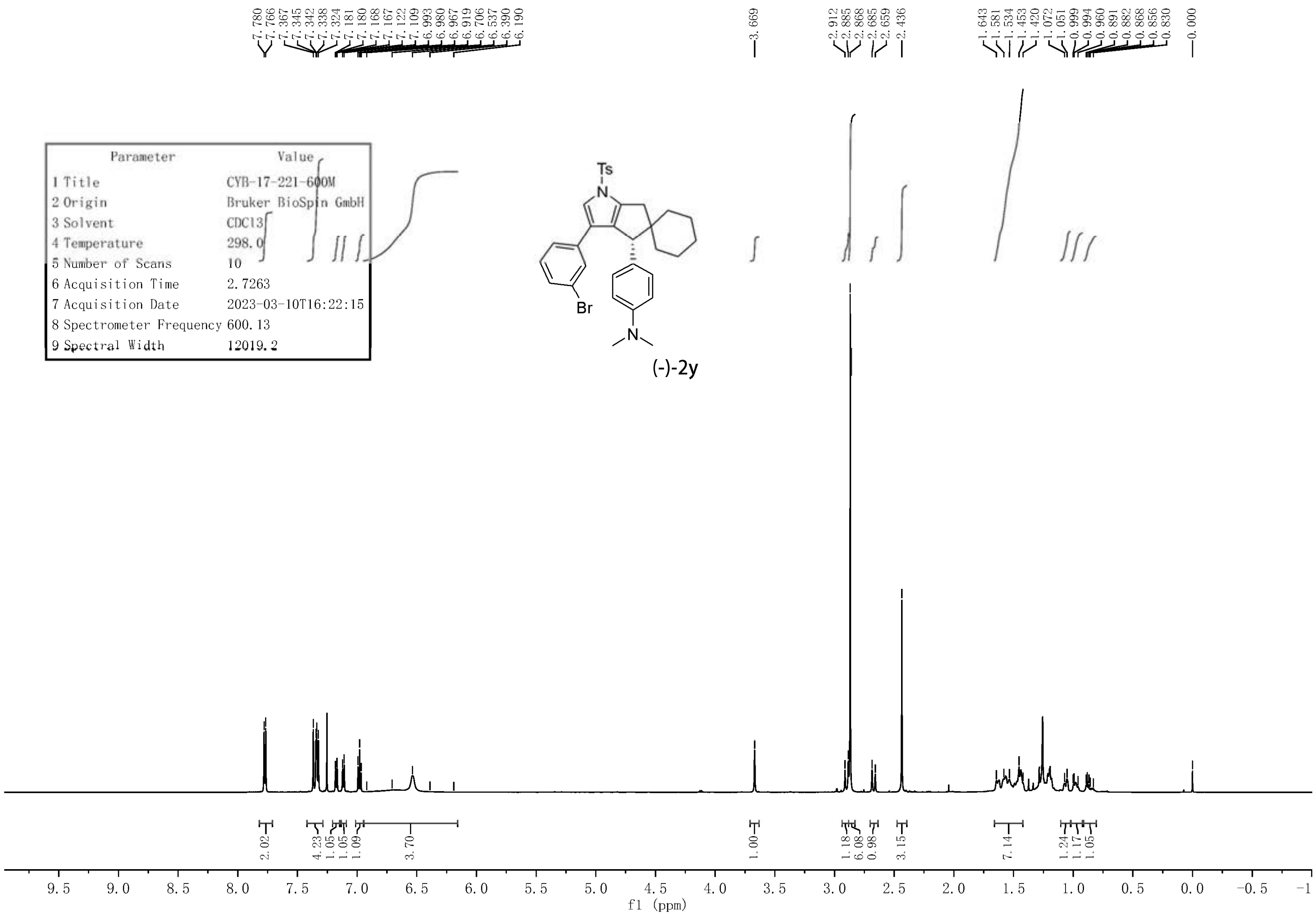

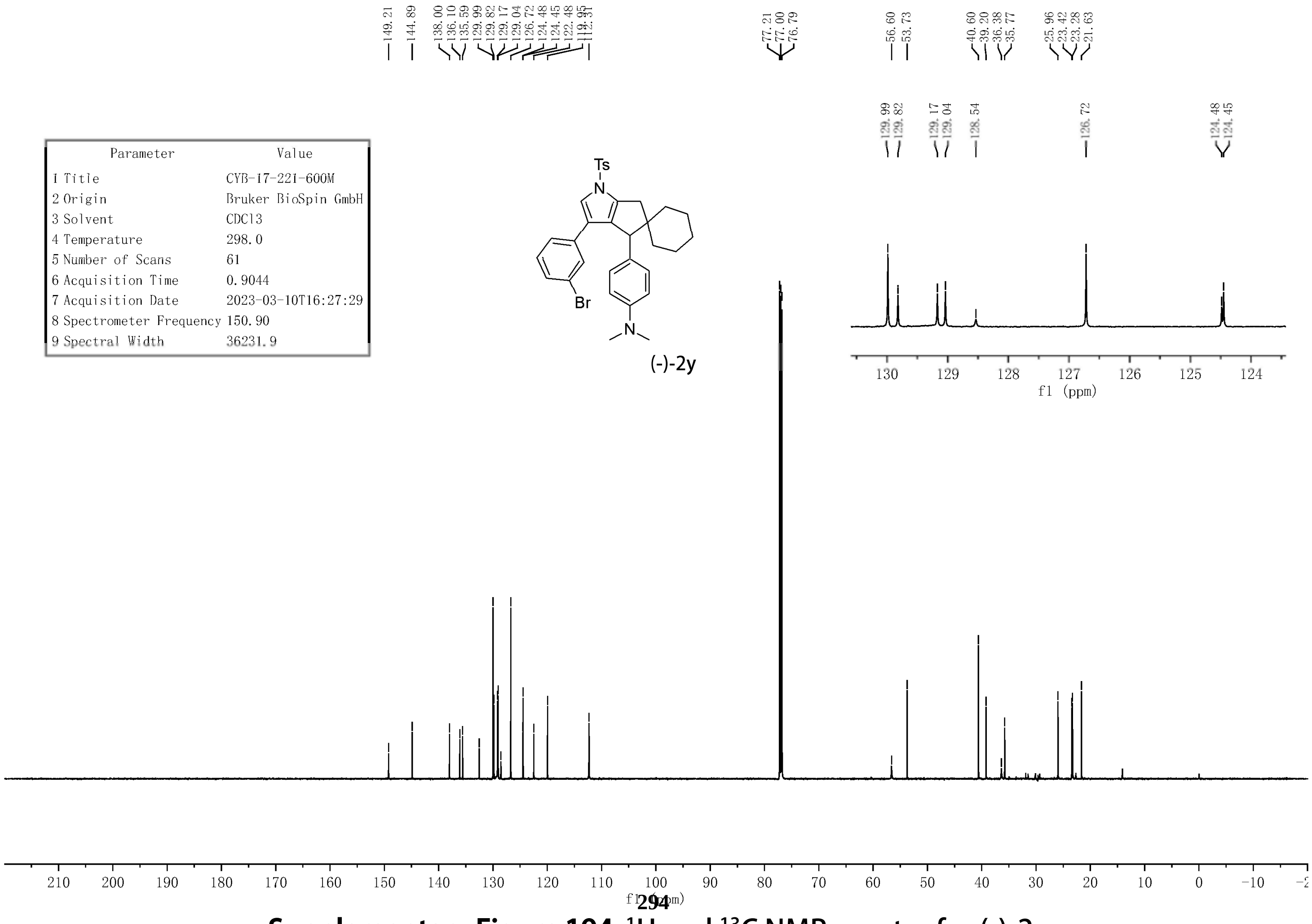

Supplementary Figure 194. ^{1}H and ^{13}C NMR spectra for (-)-2y

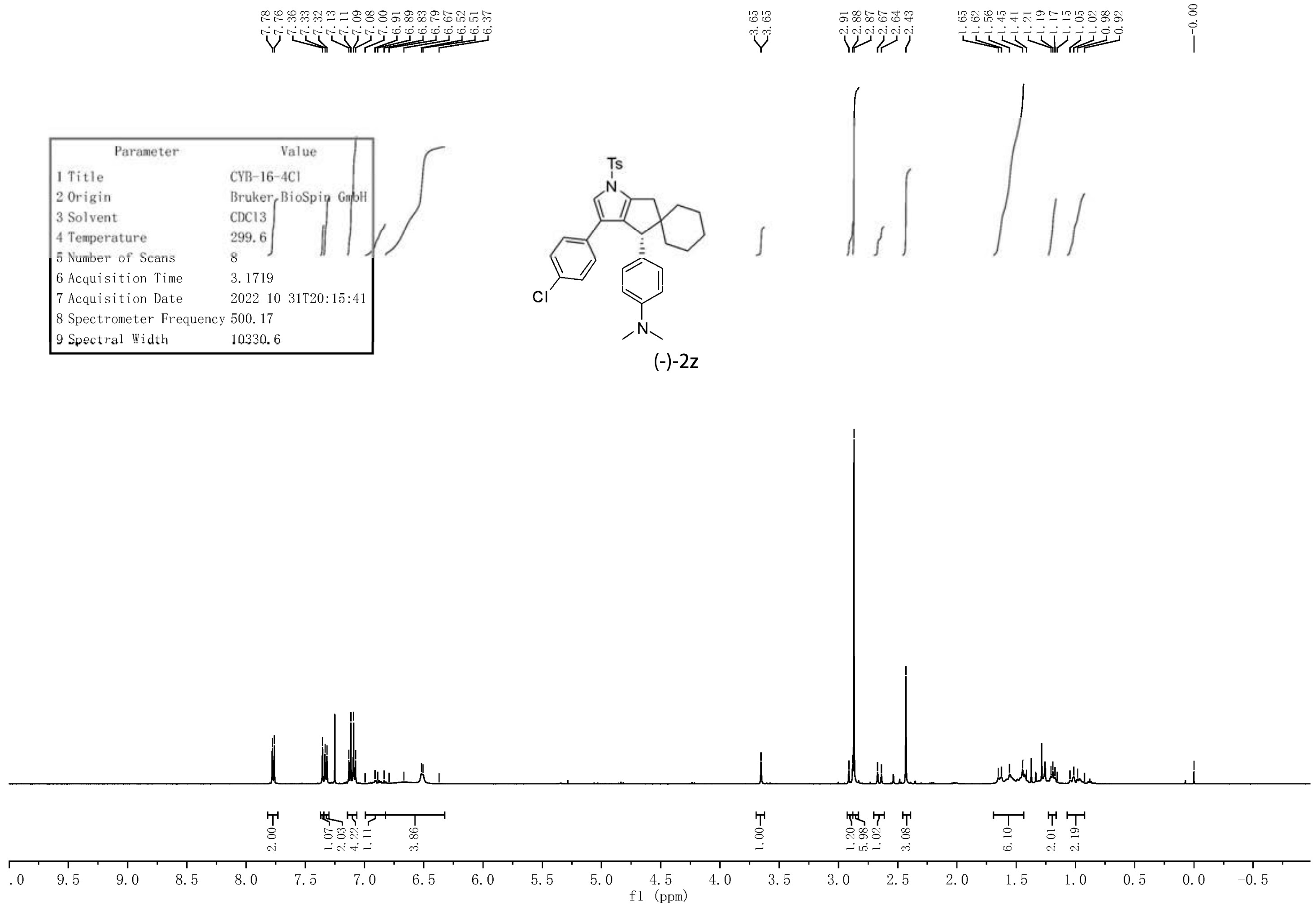

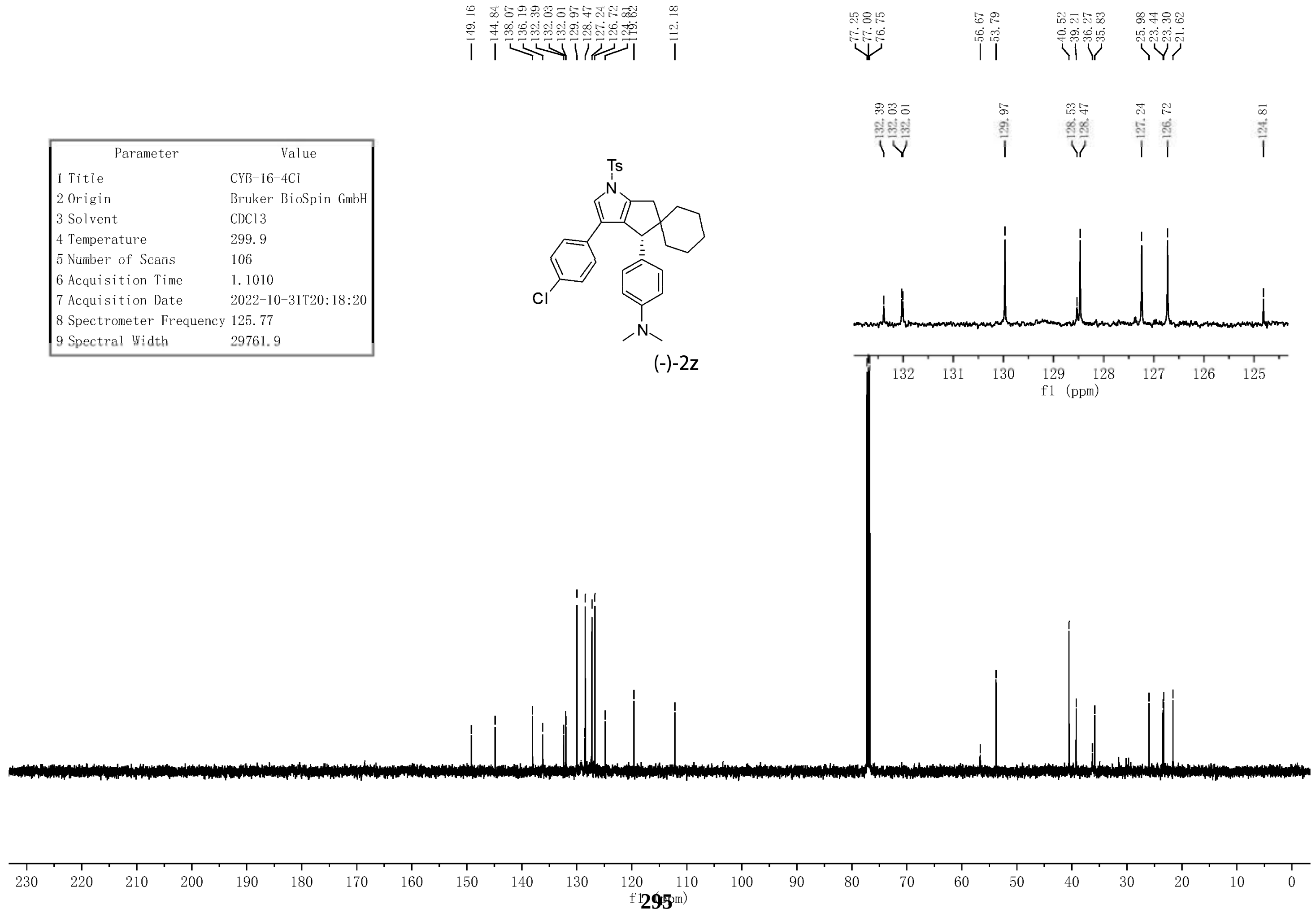

Supplementary Figure 195. ^{1}H and ^{13}C NMR spectra for (-)-2z

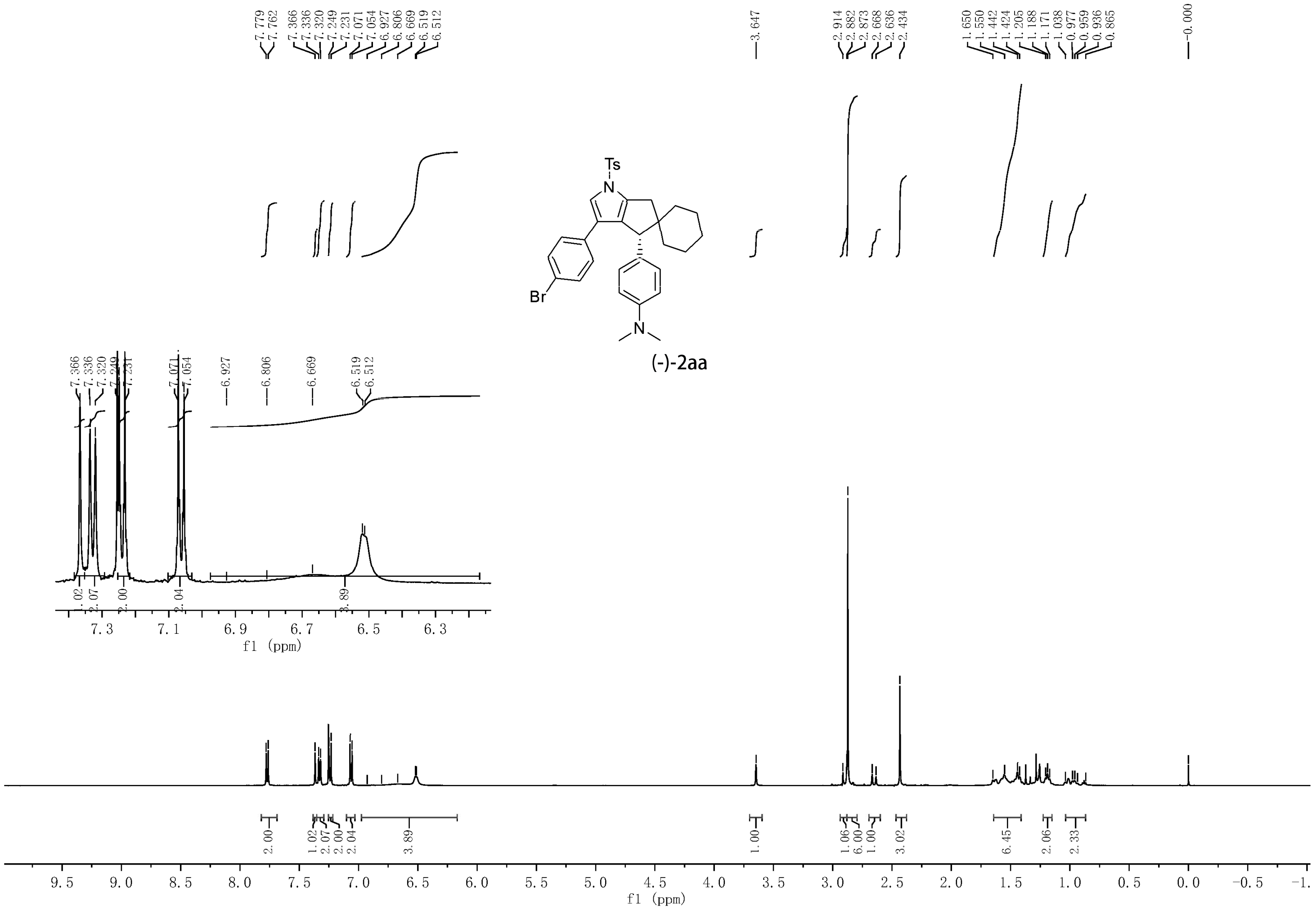

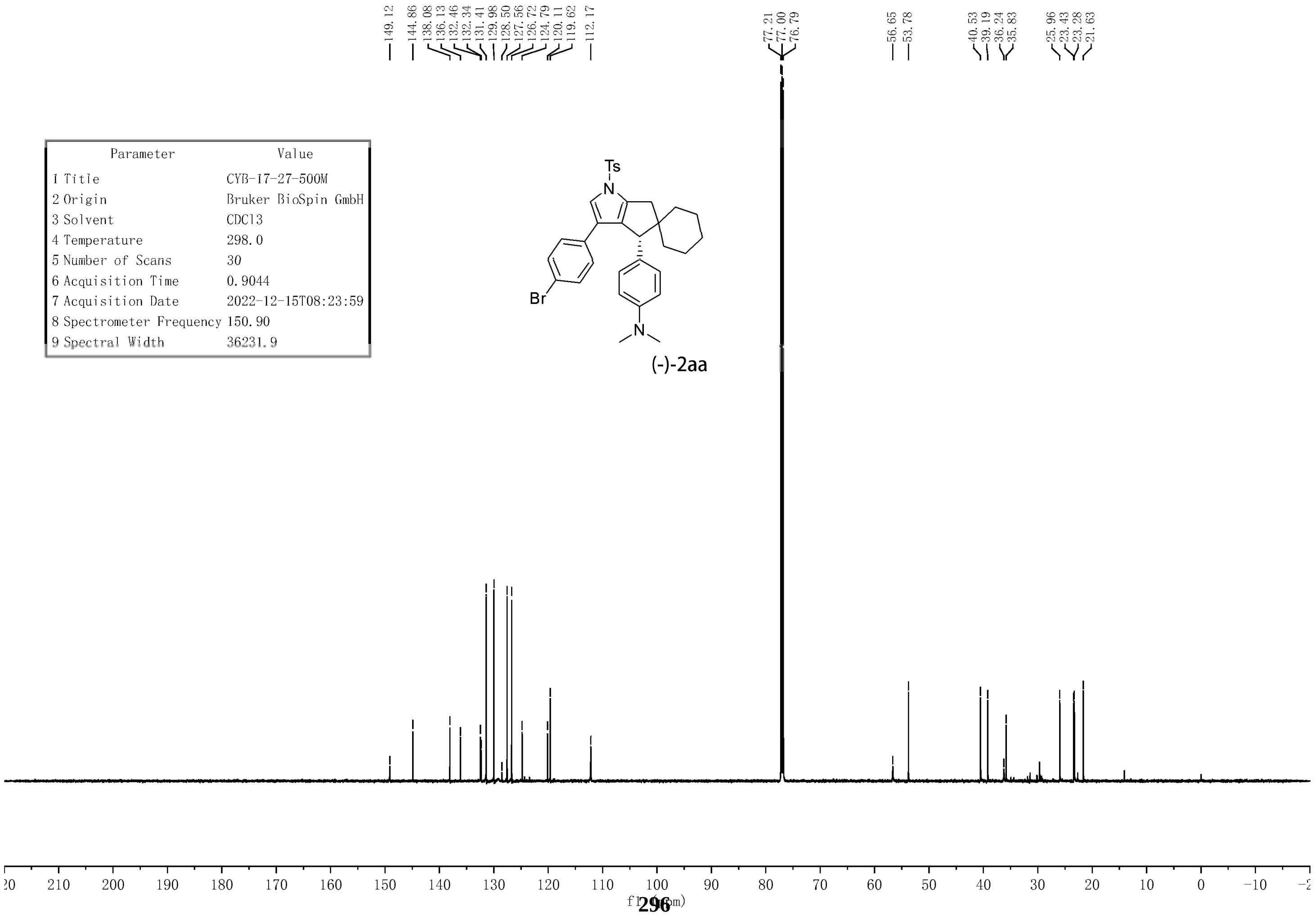

Supplementary Figure 196. ^{1}H and ^{13}C NMR spectra for (-)-2aa

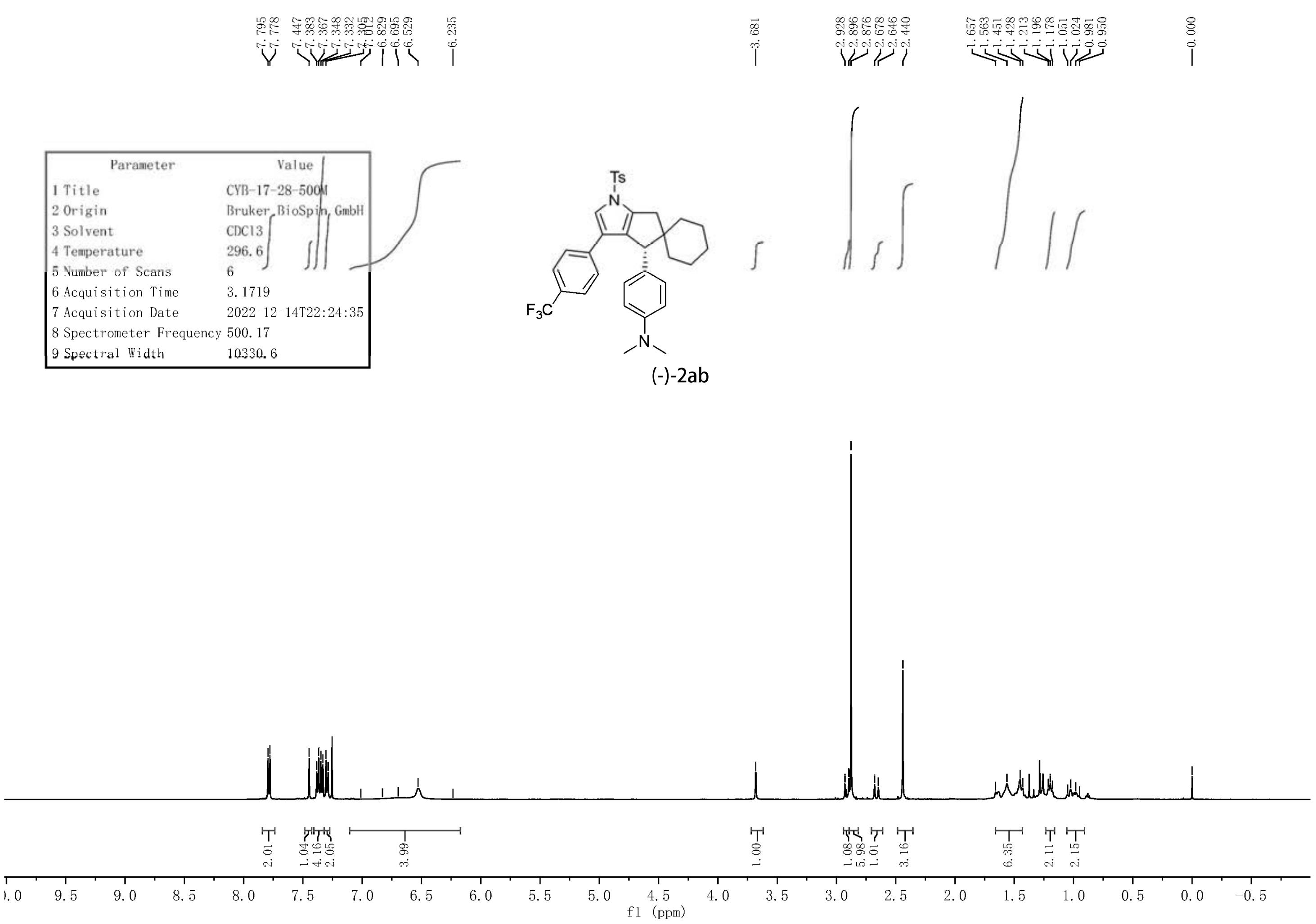

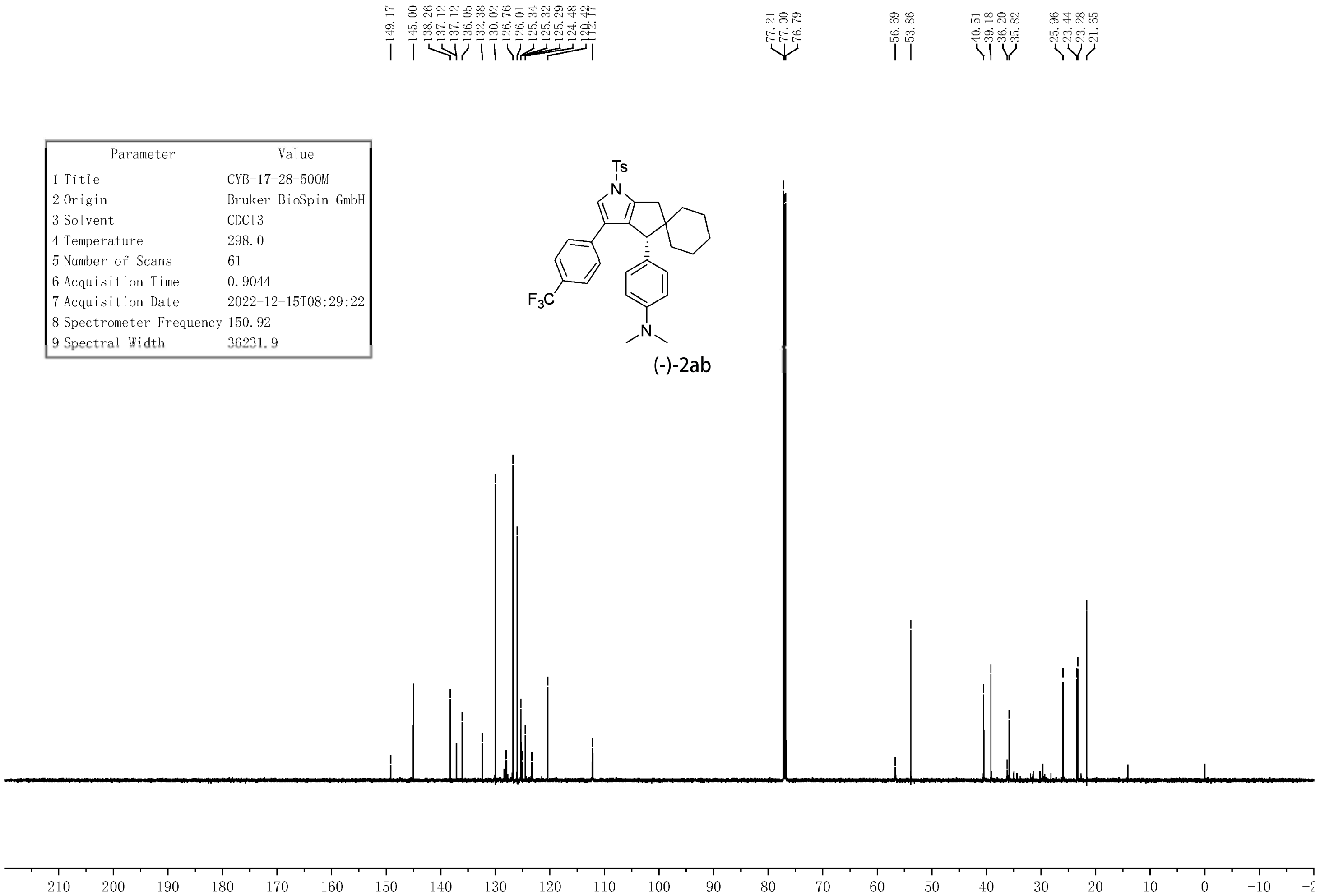

Supplementary Figure 197. ^{1}H and ^{13}C NMR spectra for (-)-2ab

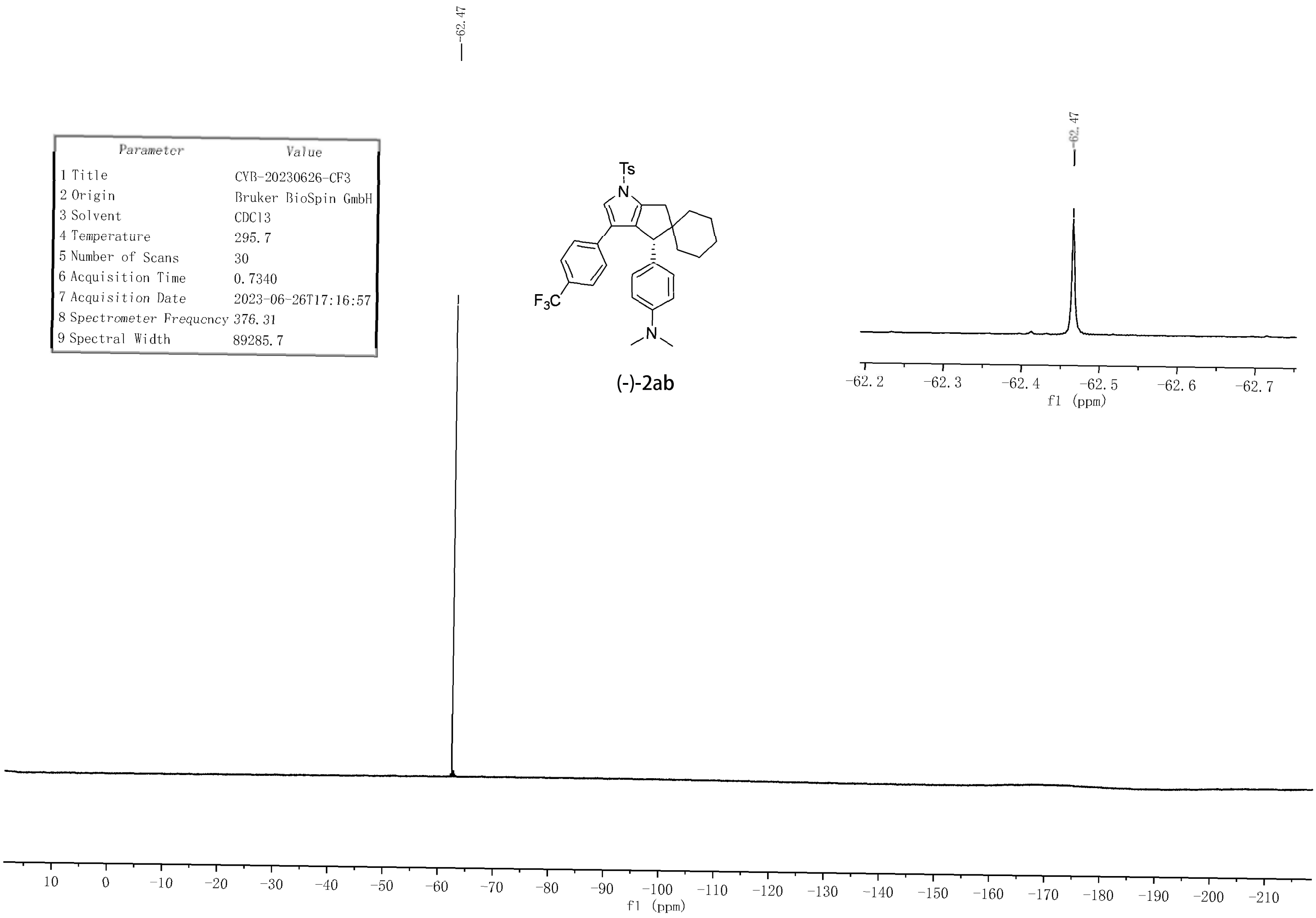

	Parameter	Value
1	Title	CYB-20230626-CF3
2	Origin	Bruker BioSpin GmbH
3	Solvent	CDCl3
4	Temperature	295.7
5	Number of Scans	30
6	Acquisition Time	0.7340
7	Acquisition Date	2023-06-26T17:16:57
8	Spectrometer Frequency	376.31
9	Spectral Width	89285.7

Supplementary Figure 198. ^{19}F NMR spectra for (-)-2ab

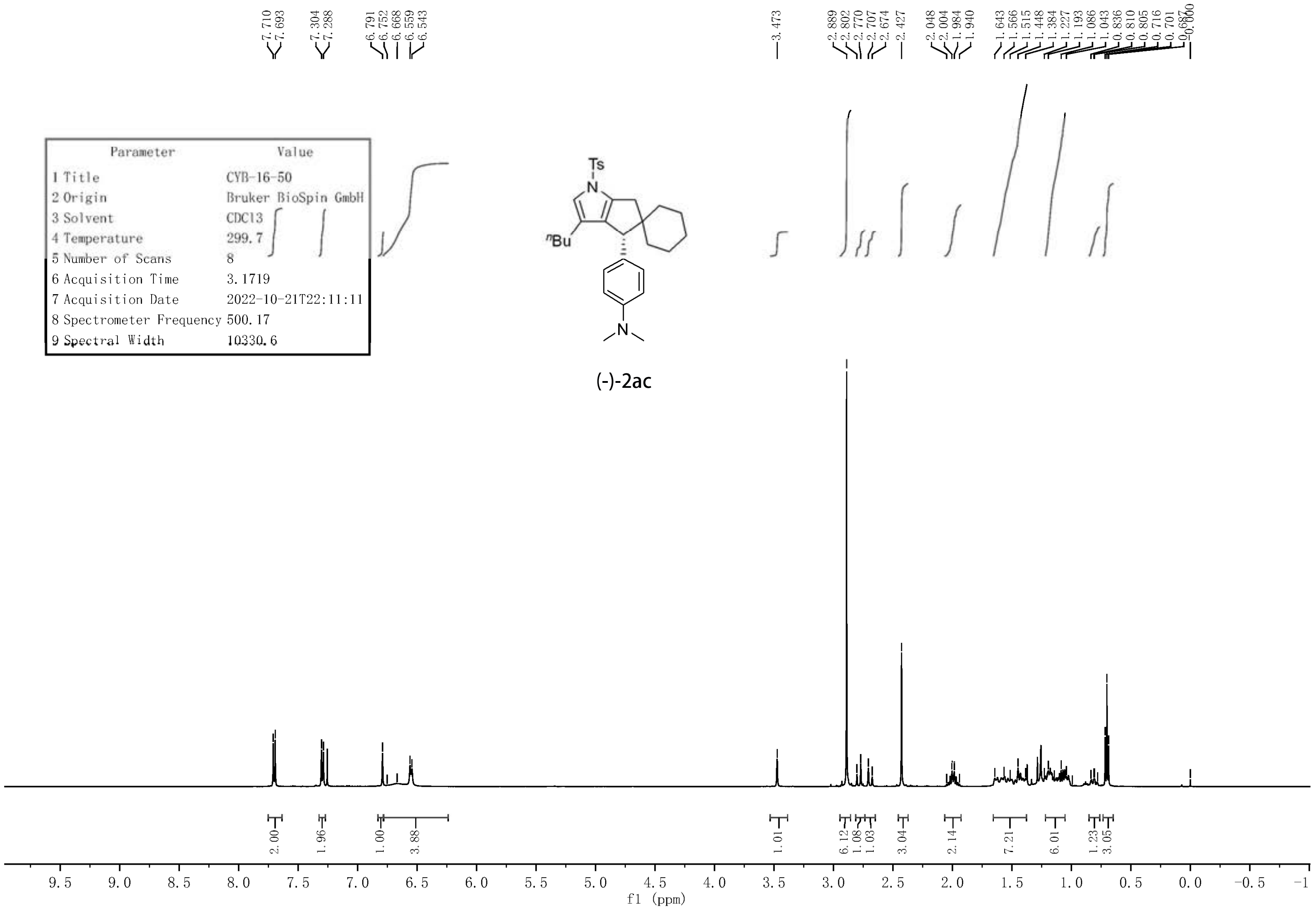

Parameter	Value
1 Title	CYB-16-50
2 Origin	Bruker BioSpin GmbH
3 Solvent	CDCl3
4 Temperature	299.7
5 Number of Scans	8
6 Acquisition Time	3.1719
7 Acquisition Date	2022-10-21T22:11:11
8 Spectrometer Frequency	500.17
9 Spectral Width	10330.6

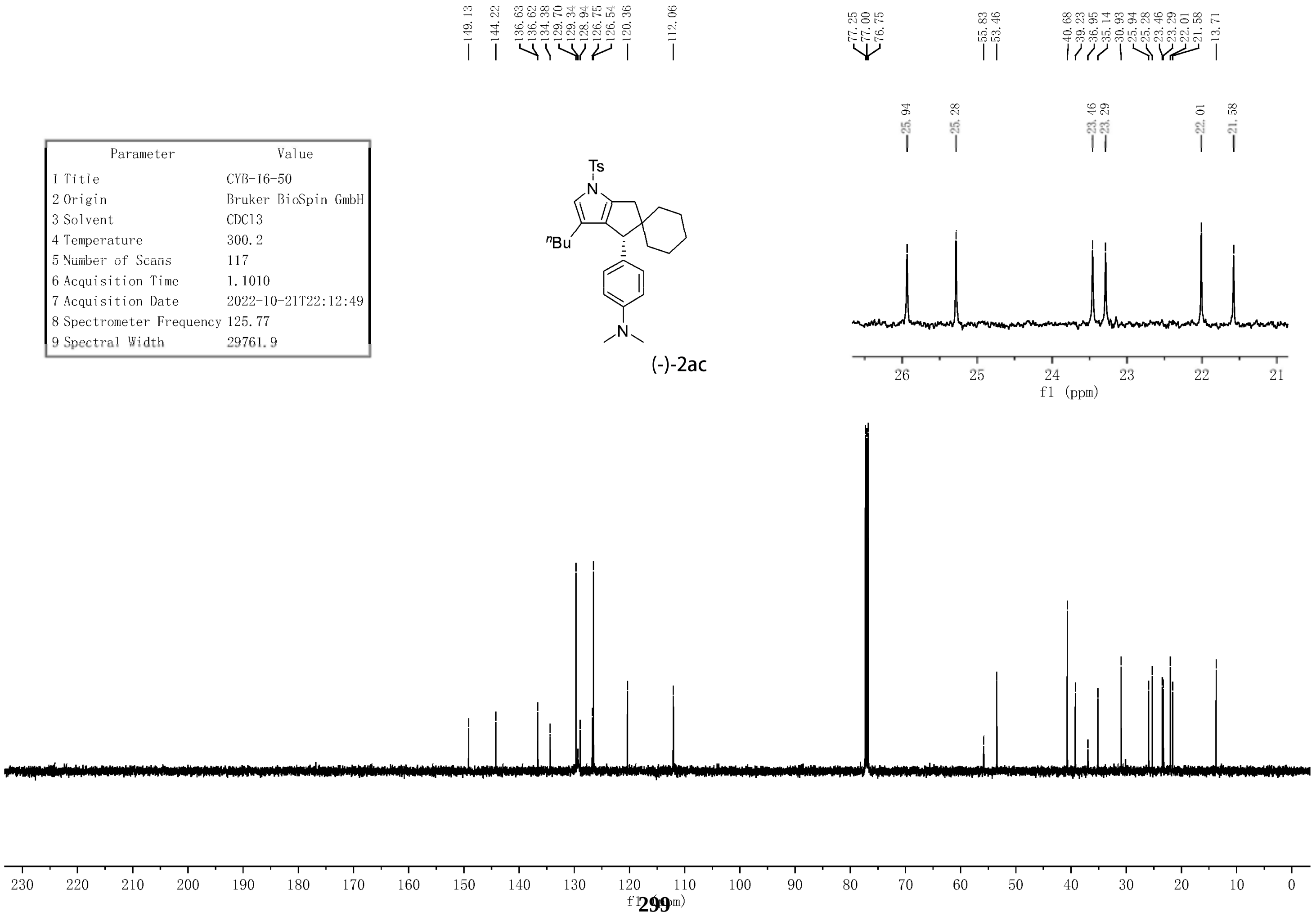

Parameter	Value
1 Title	CYB-16-50
2 Origin	Bruker BioSpin GmbH
3 Solvent	CDCl3
4 Temperature	300.2
5 Number of Scans	117
6 Acquisition Time	1.1010
7 Acquisition Date	2022-10-21T22:12:49
8 Spectrometer Frequency	125.77
9 Spectral Width	29761.9

Supplementary Figure 199. ^{1}H and ^{13}C NMR spectra for (-)-2ac

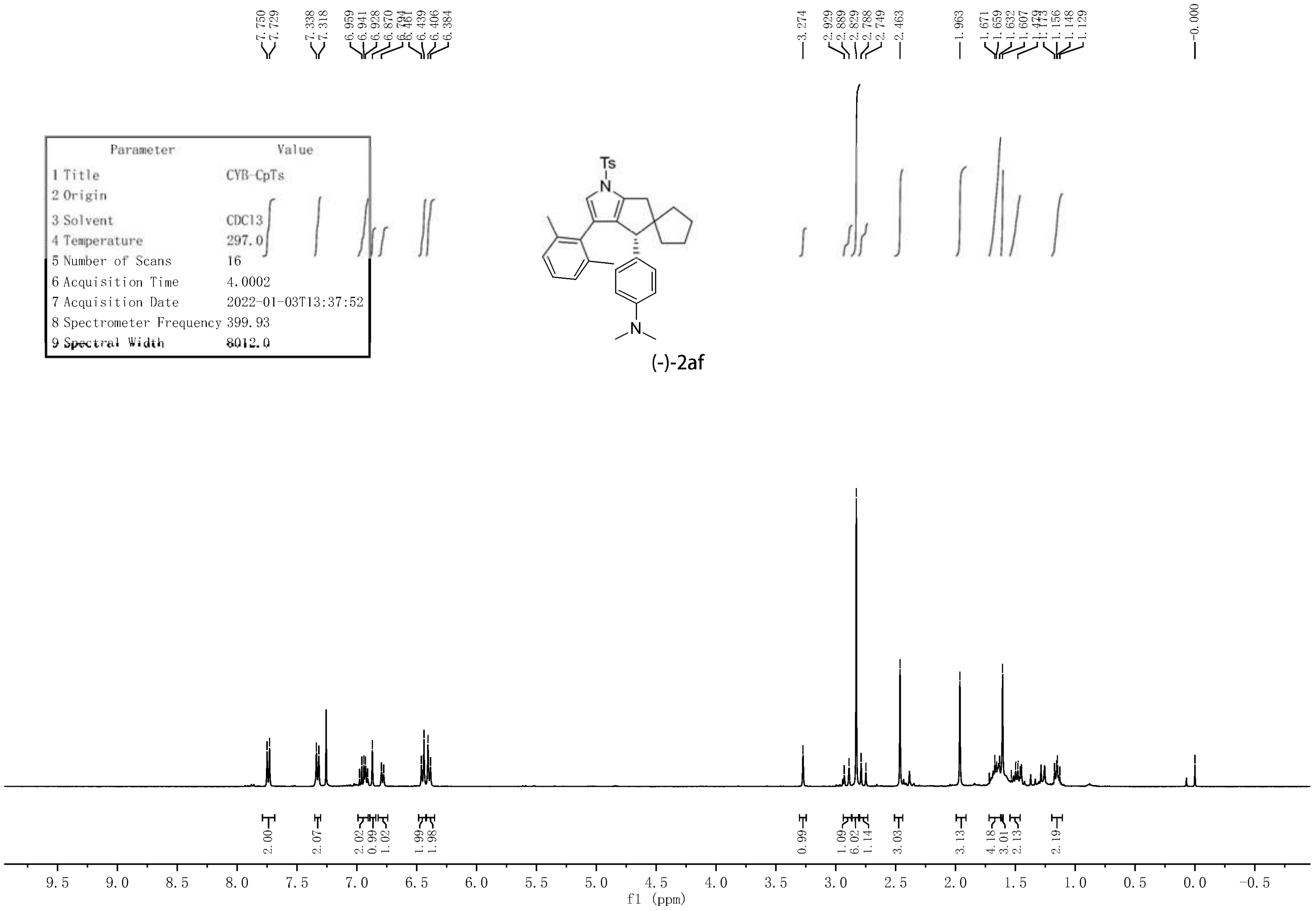

Parameter	Value
1 Title	CYB-CpTs
2 Origin	
3 Solvent	CDCl3
4 Temperature	297.0
5 Number of Scans	16
6 Acquisition Time	4.0002
7 Acquisition Date	2022-01-03T13:37:52
8 Spectrometer Frequency	399.93
9 Spectral Width	8012.0

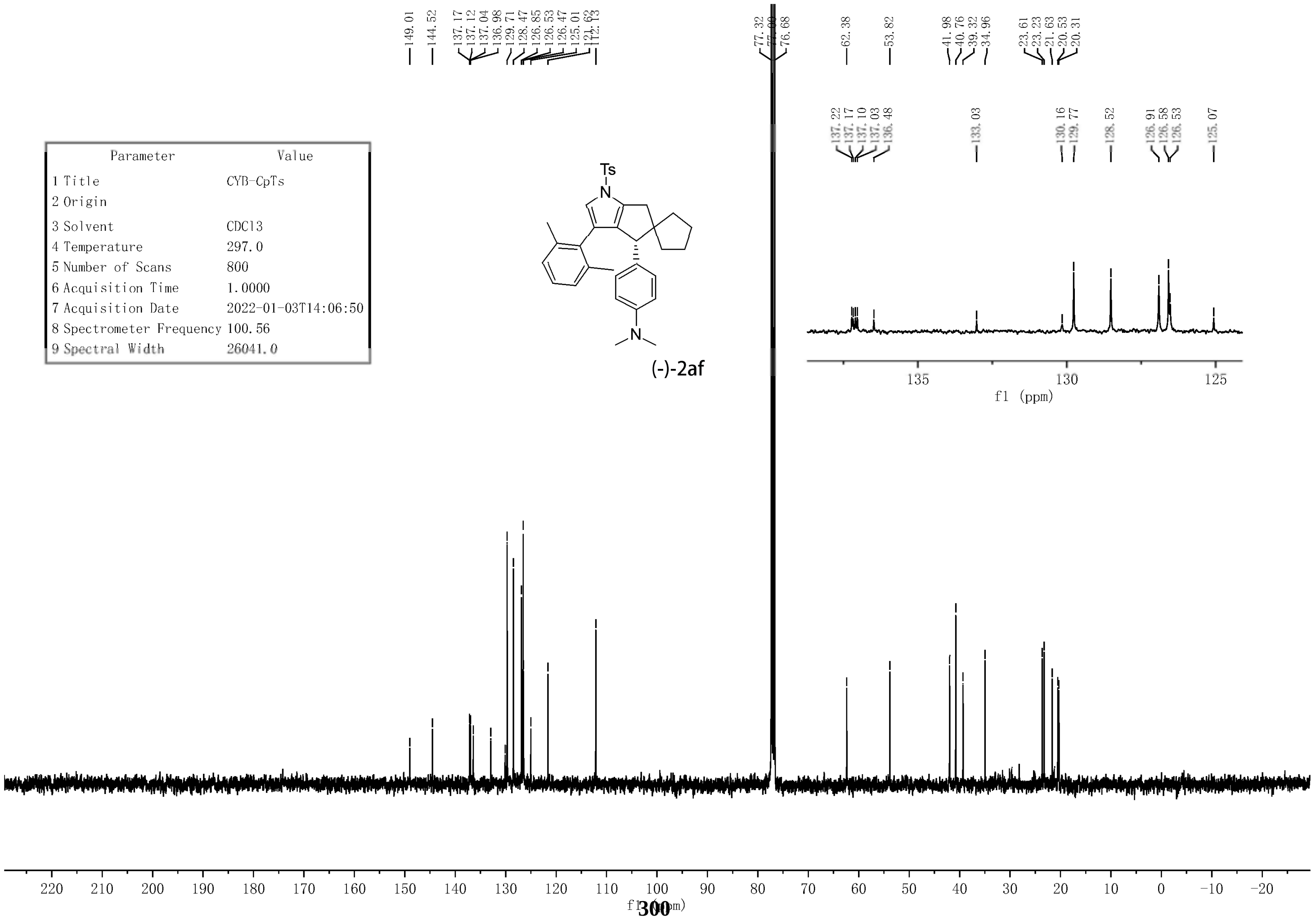

Parameter	Value
1 Title	CYB-CpTs
2 Origin	
3 Solvent	CDCl3
4 Temperature	297.0
5 Number of Scans	800
6 Acquisition Time	1.0000
7 Acquisition Date	2022-01-03T14:06:50
8 Spectrometer Frequency	100.56
9 Spectral Width	26041.0

Supplementary Figure 200. ^{1}H and ^{13}C NMR spectra for (-)-2af

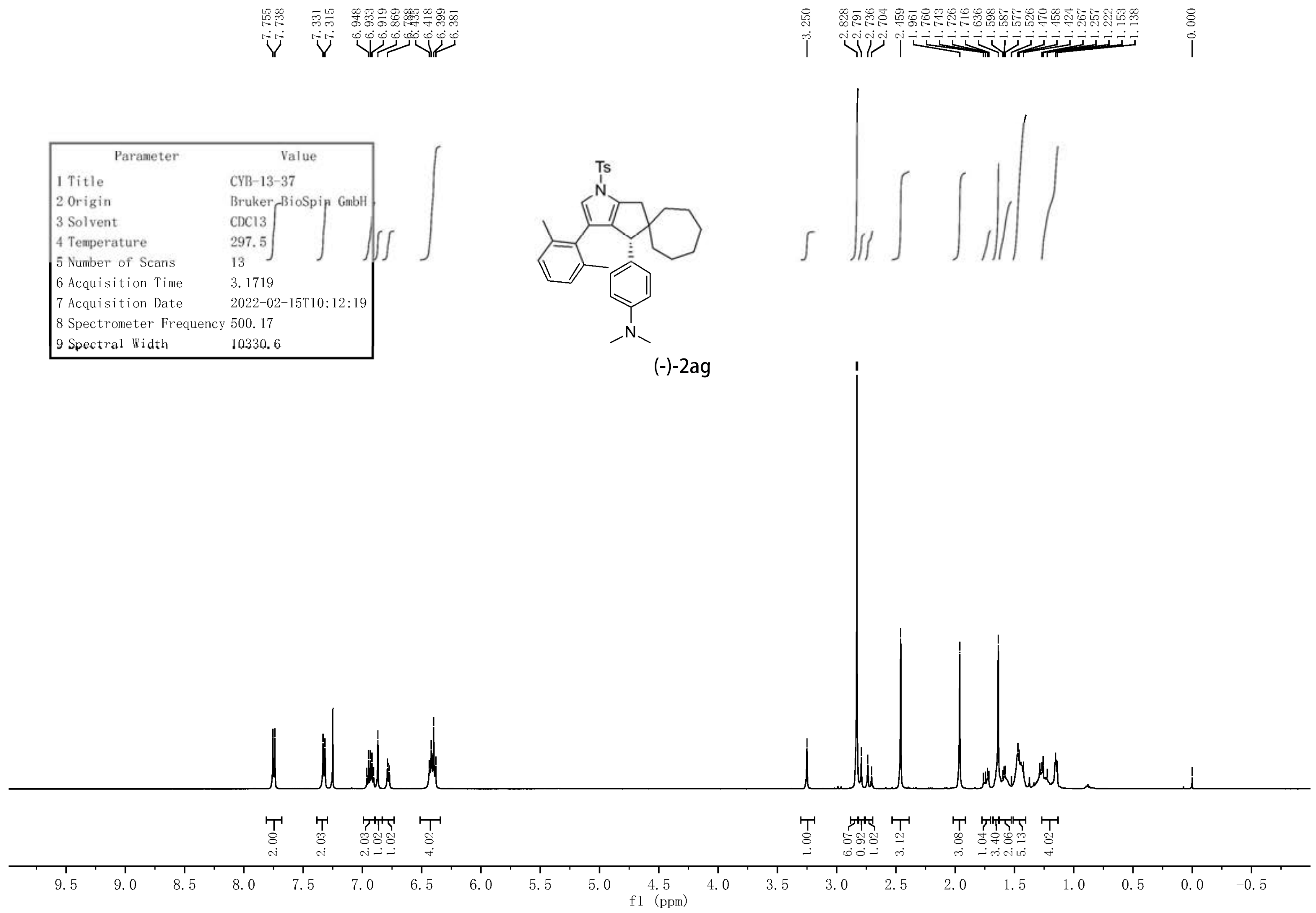

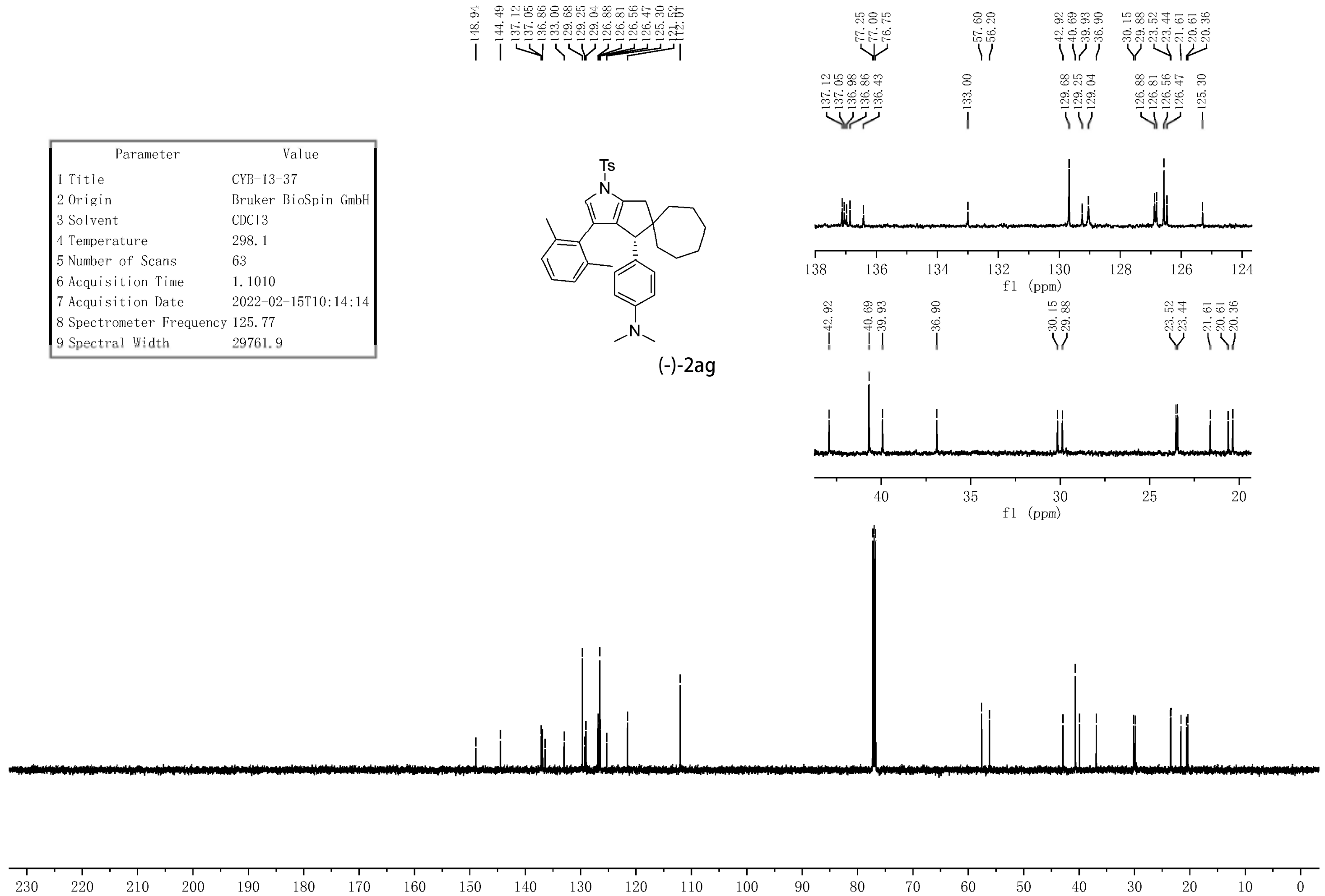

Supplementary Figure 201. ^{1}H and ^{13}C NMR spectra for (-)-2ag

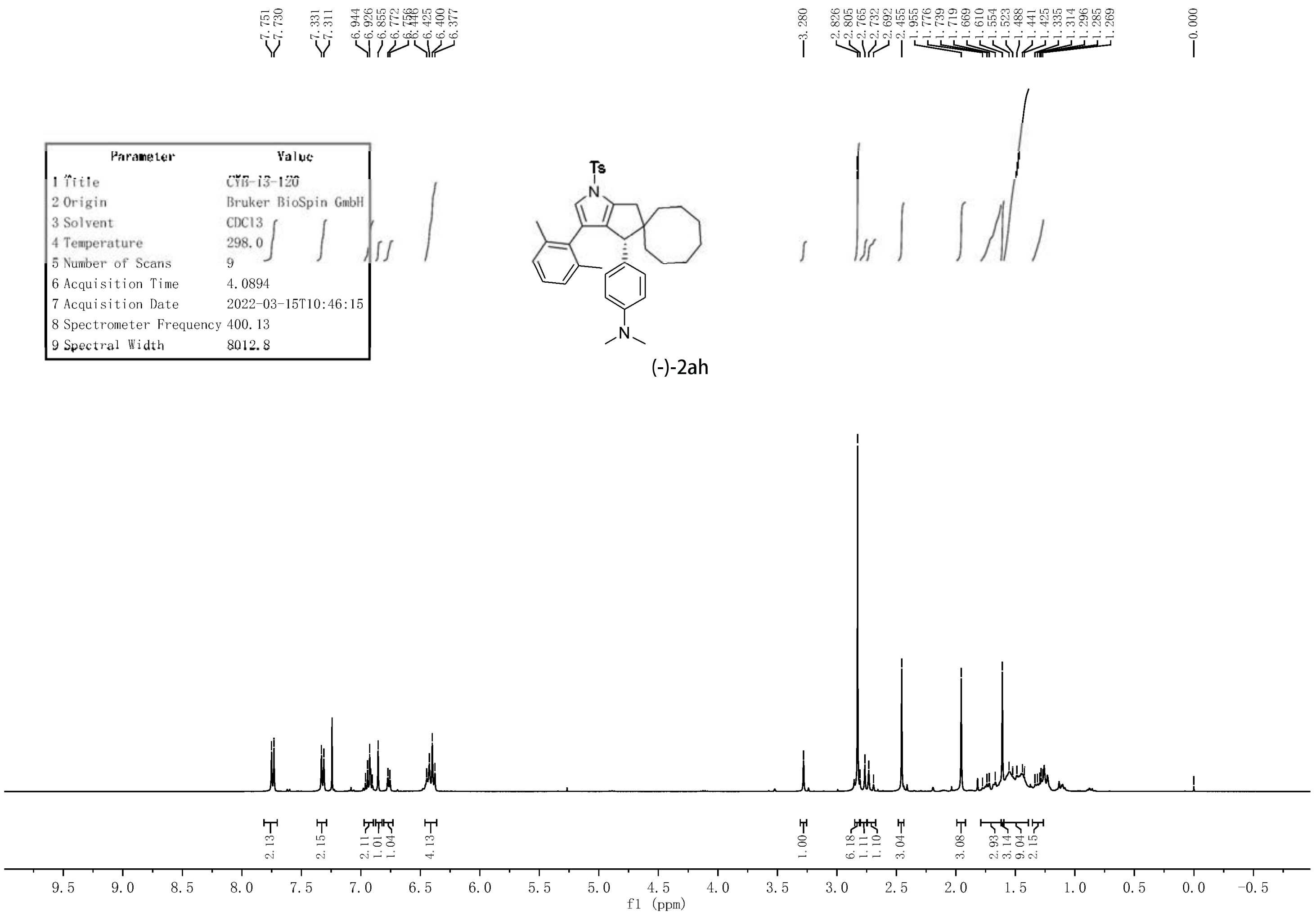

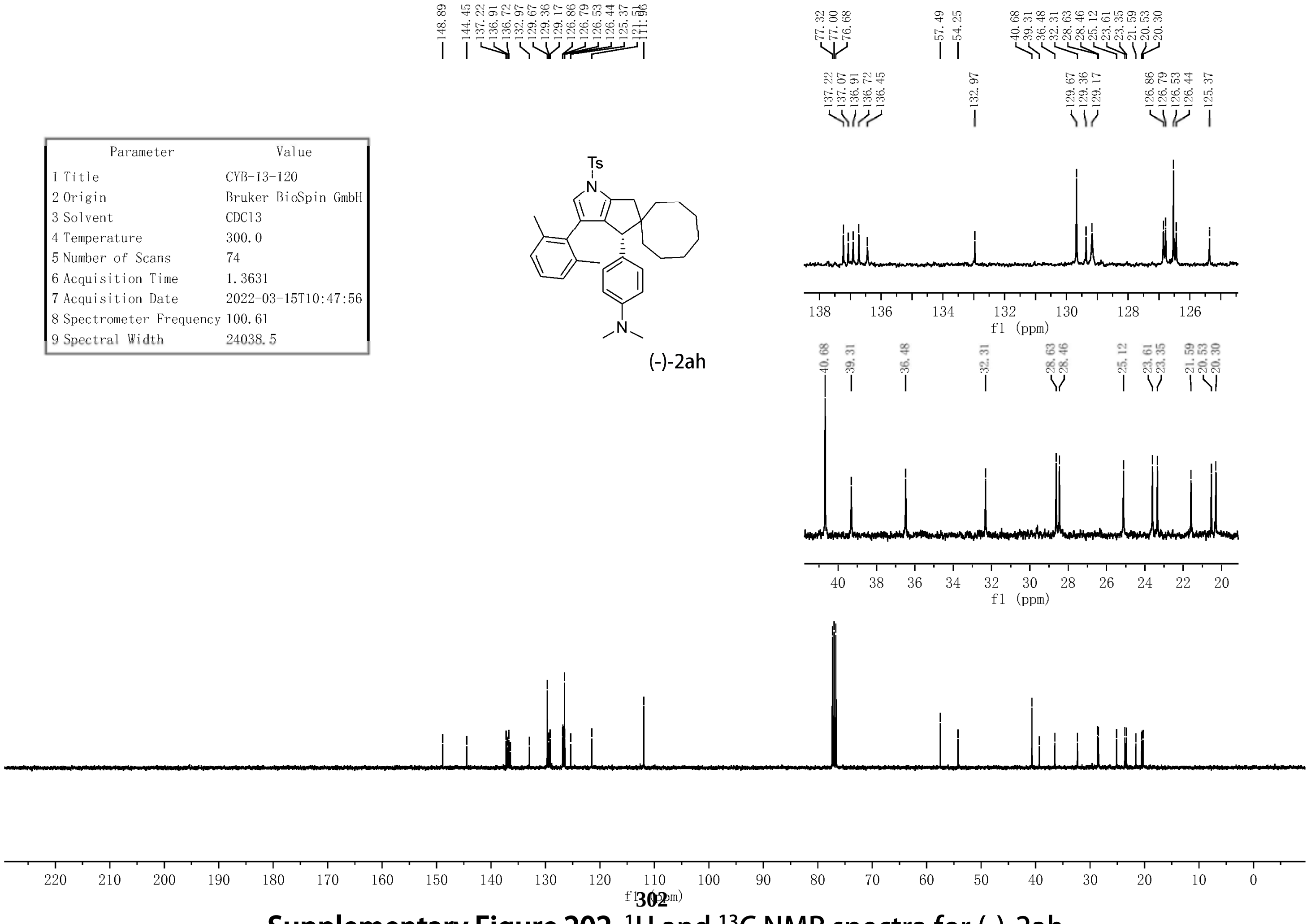

Supplementary Figure 202. ^{1}H and ^{13}C NMR spectra for (-)-2ah

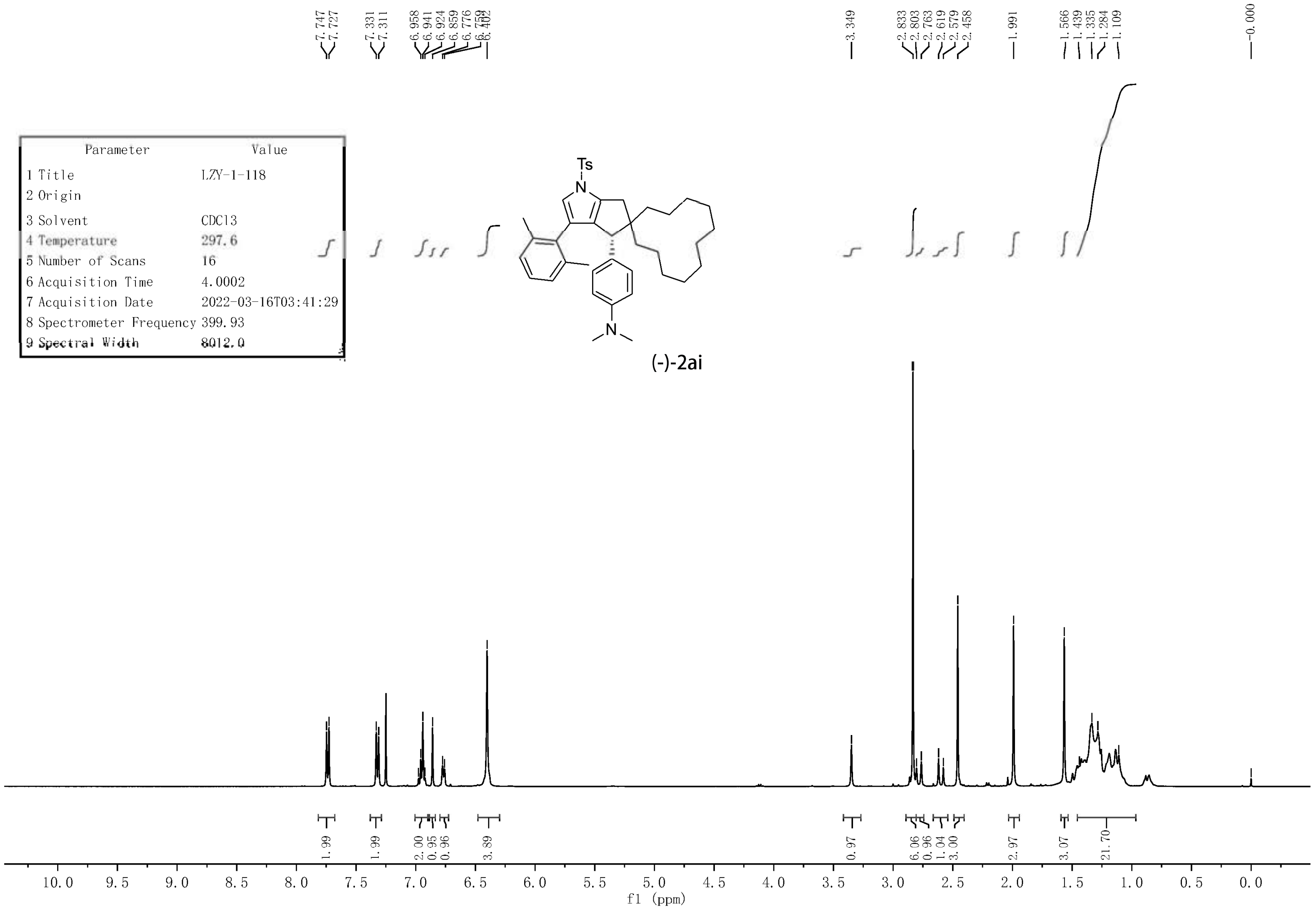

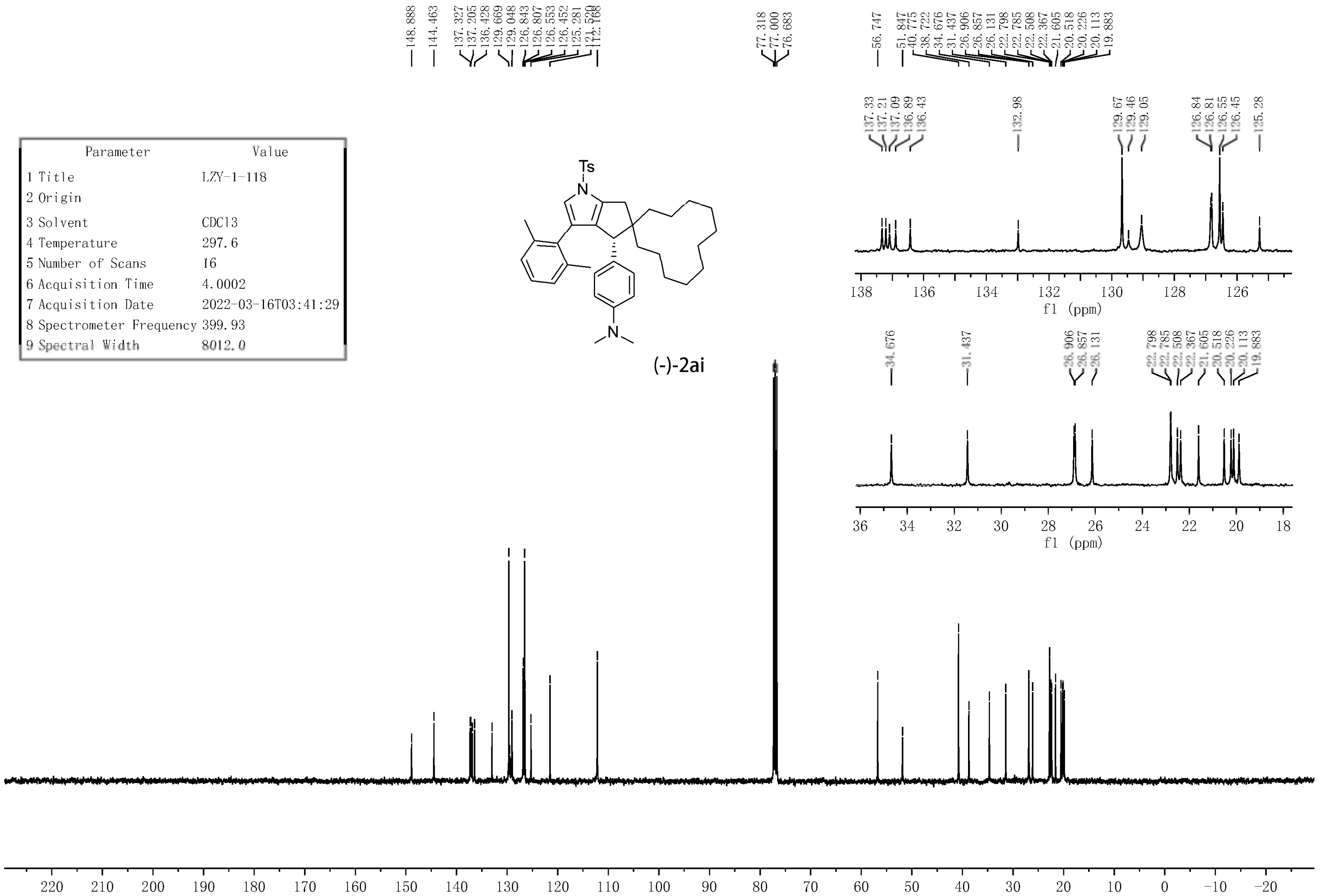

Supplementary Figure 203. ^{1}H and ^{13}C NMR spectra for (-)-2ai

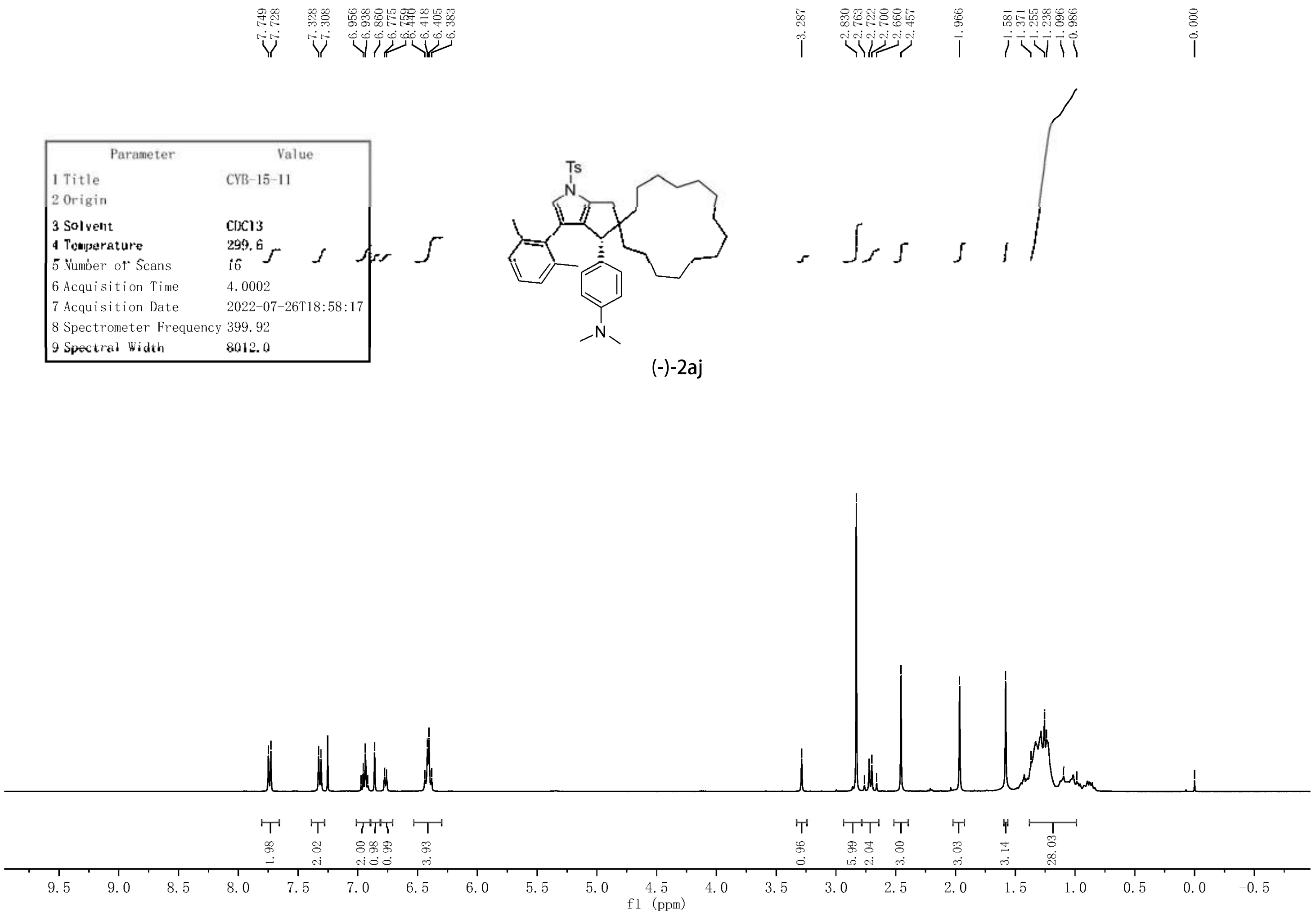

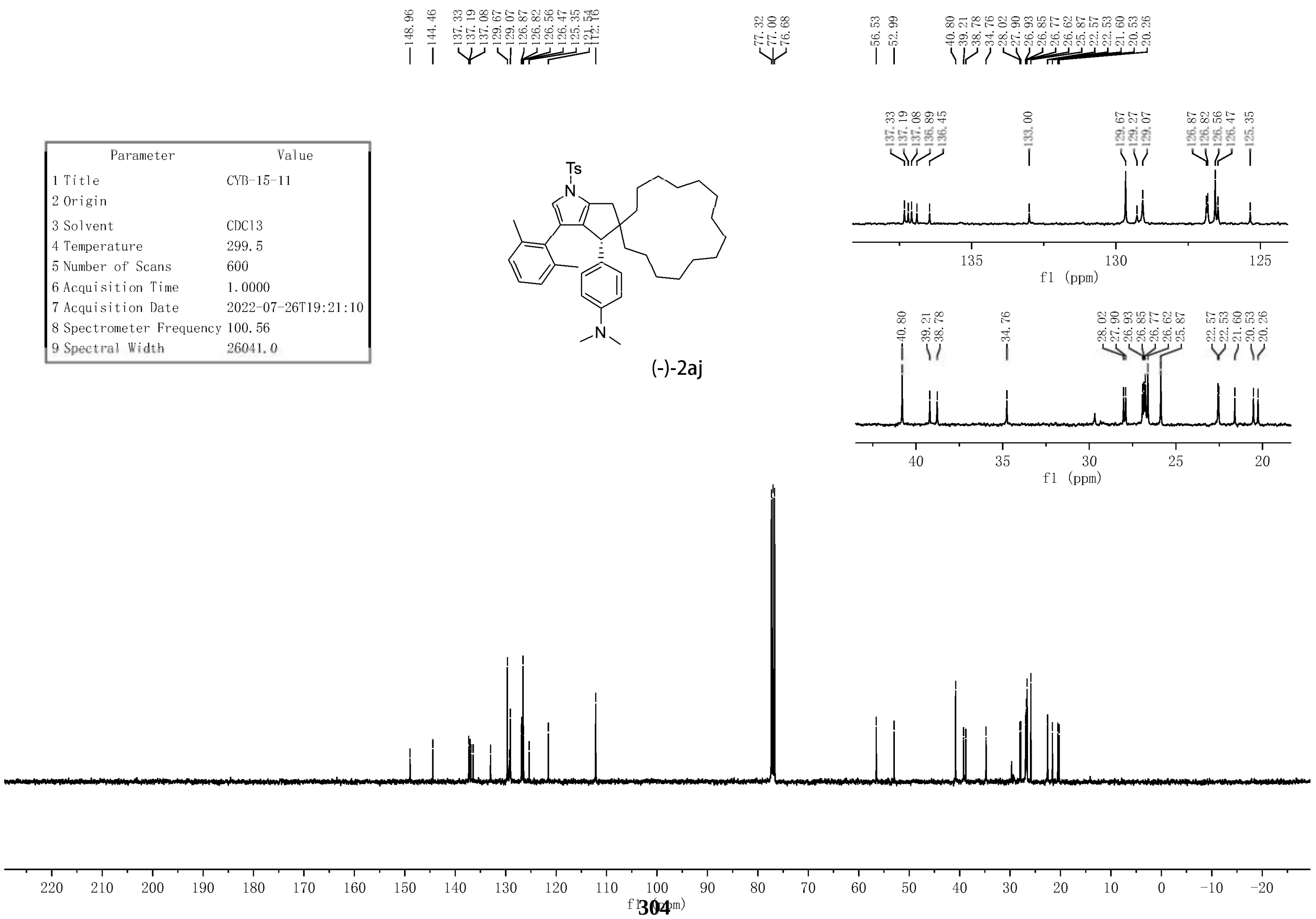

Supplementary Figure 204. ^{1}H and ^{13}C NMR spectra for (-)-2aj

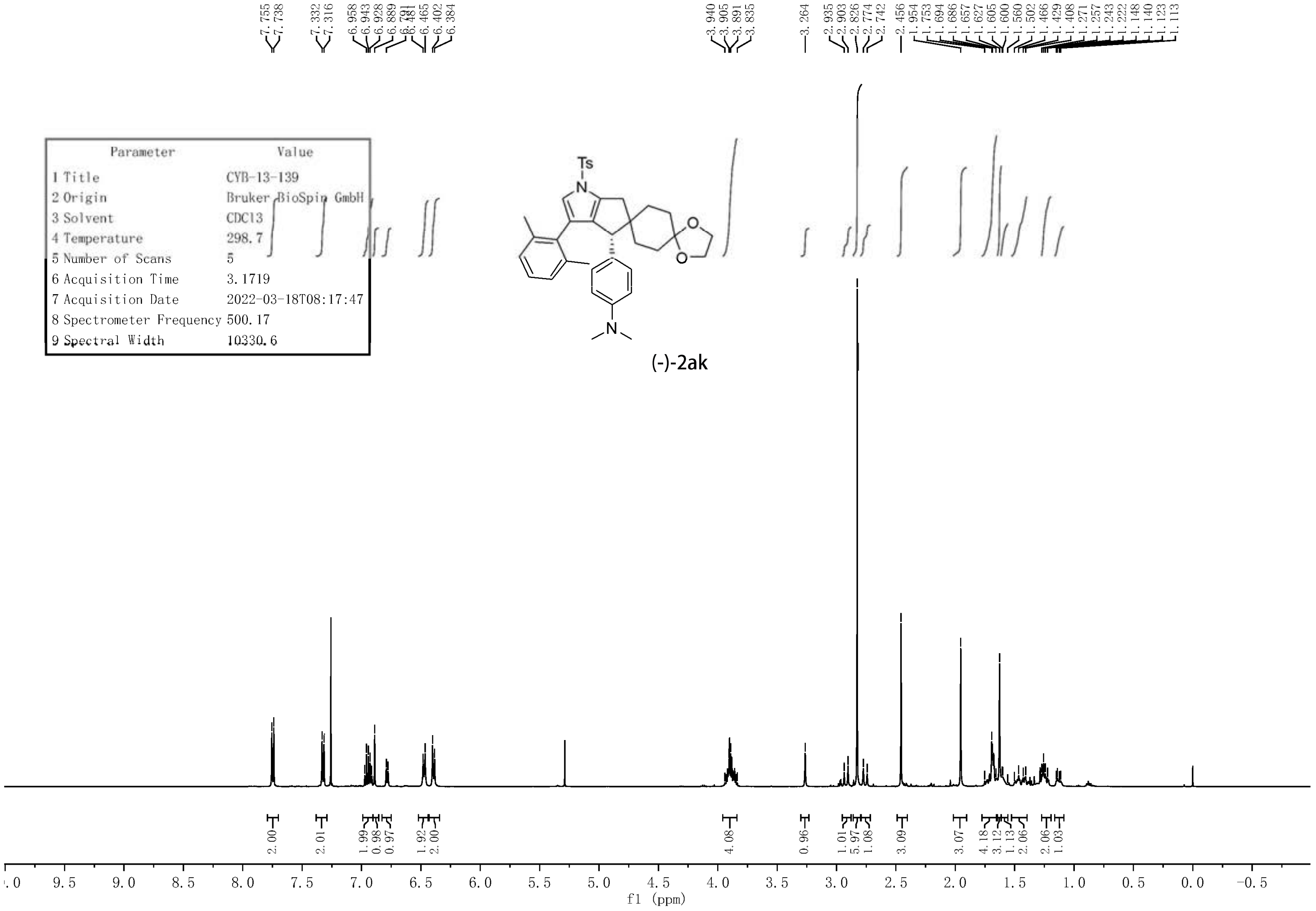

Parameter	Value
1 Title	CYB-13-139
2 Origin	Bruker BioSpin GmbH
3 Solvent	CDCl3
4 Temperature	298.7
5 Number of Scans	5
6 Acquisition Time	3.1719
7 Acquisition Date	2022-03-18T08:17:47
8 Spectrometer Frequency	500.17
9 Spectral Width	10330.6

(-)-2ak

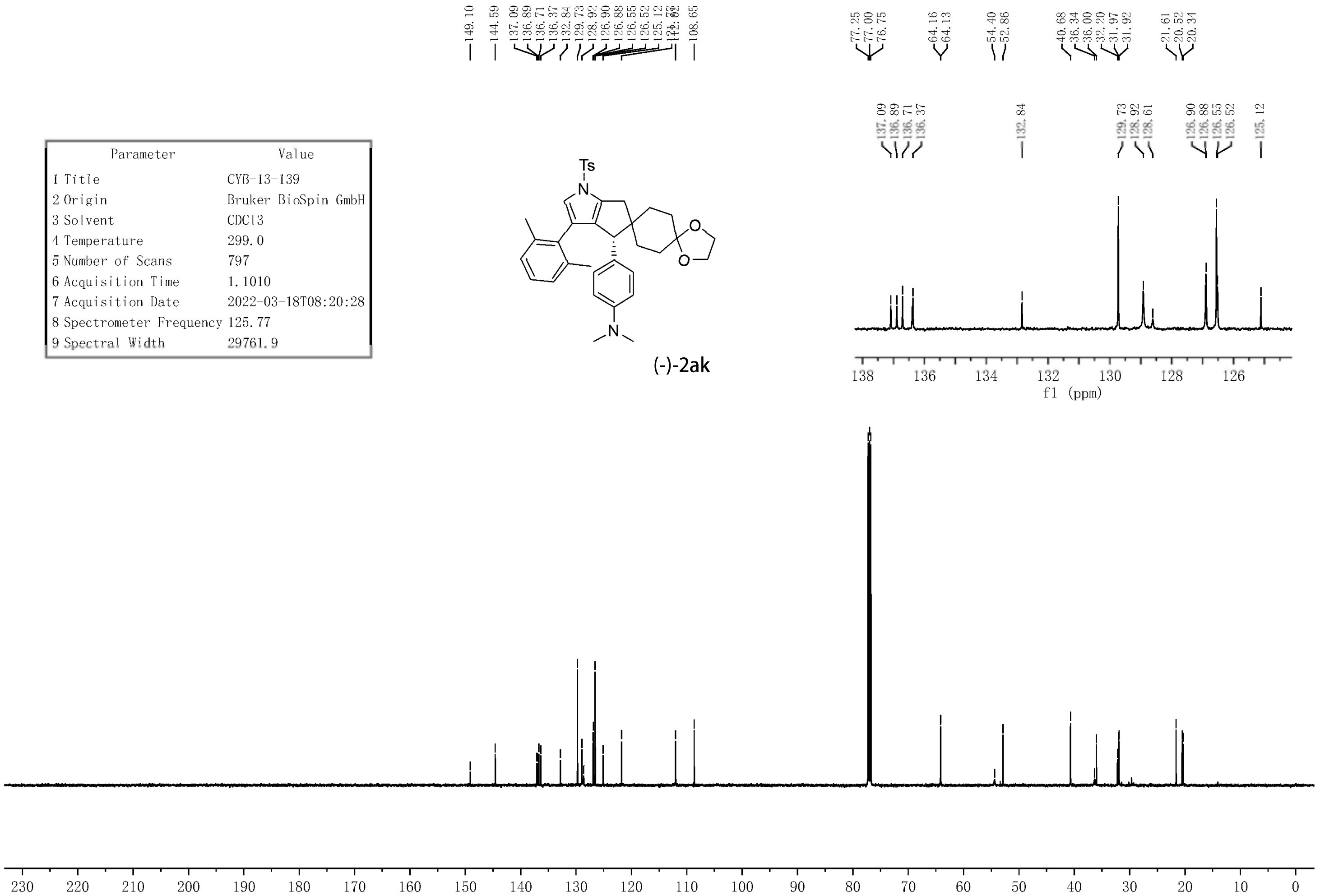

Parameter	Value
1 Title	CYB-13-139
2 Origin	Bruker BioSpin GmbH
3 Solvent	CDCl3
4 Temperature	299.0
5 Number of Scans	797
6 Acquisition Time	1.1010
7 Acquisition Date	2022-03-18T08:20:28
8 Spectrometer Frequency	125.77
9 Spectral Width	29761.9

(-)-2ak

Supplementary Figure 205. ^{1}H and ^{13}C NMR spectra for (-)-2ak

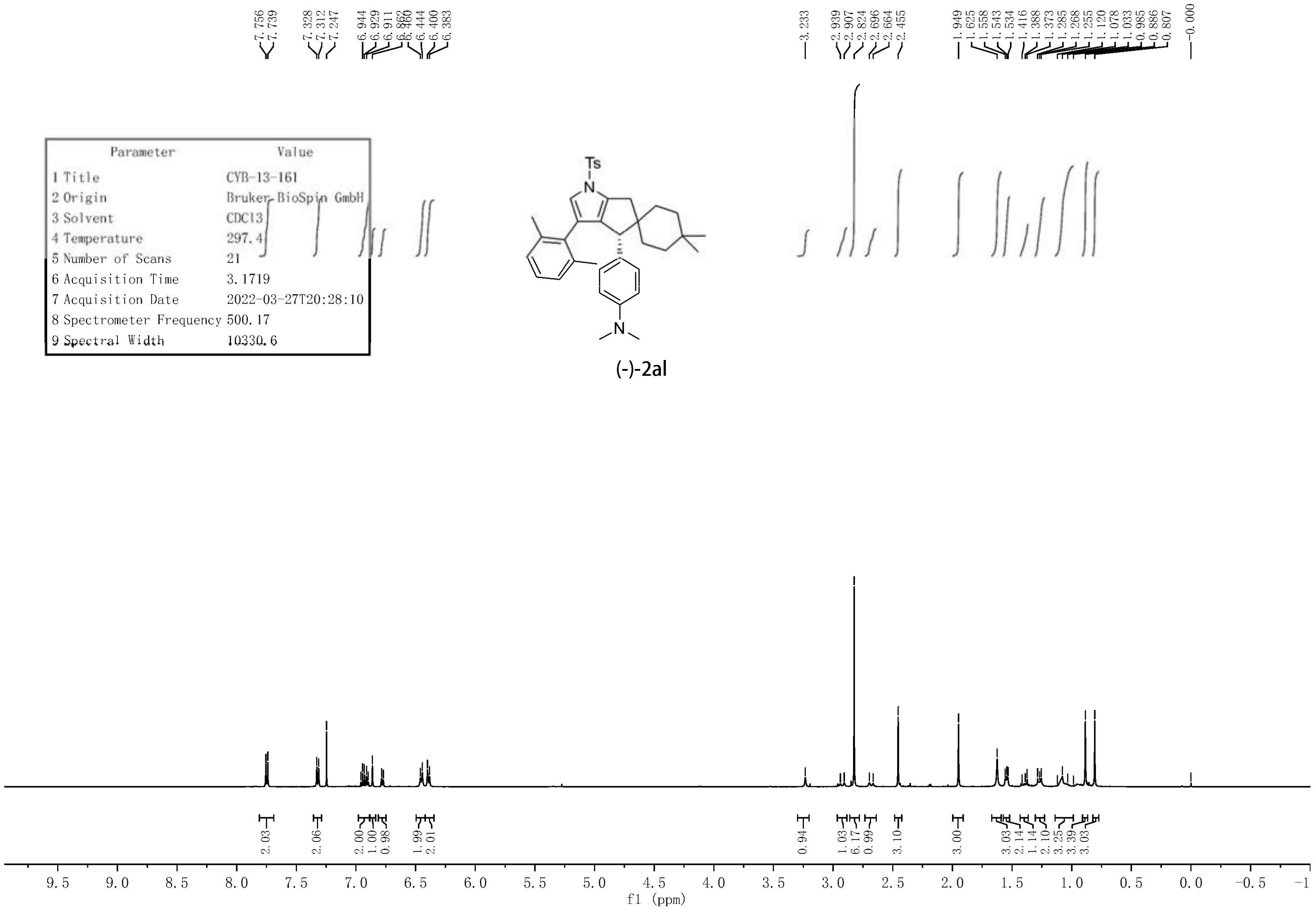

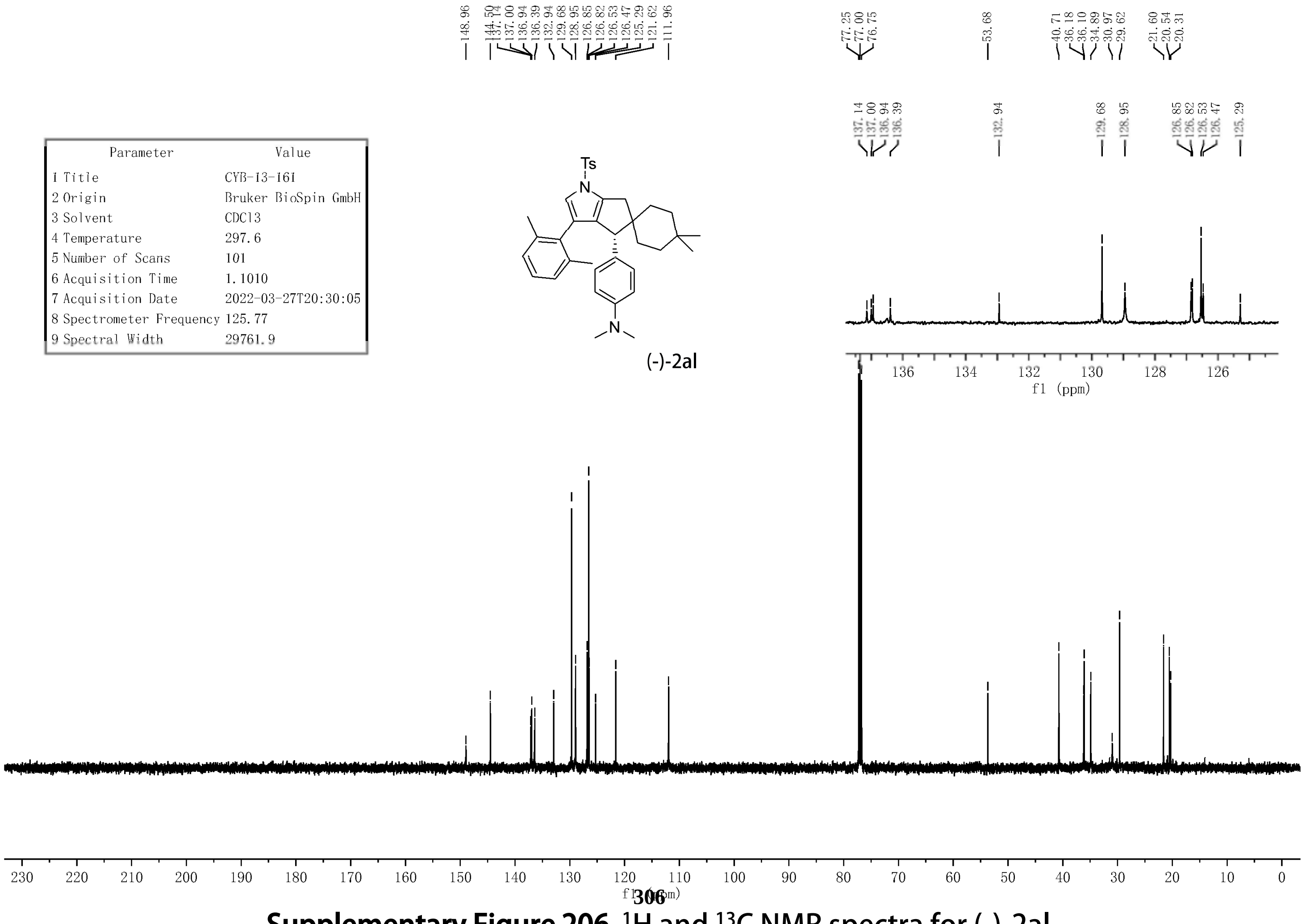

Supplementary Figure 206. ^{1}H and ^{13}C NMR spectra for (-)-2al

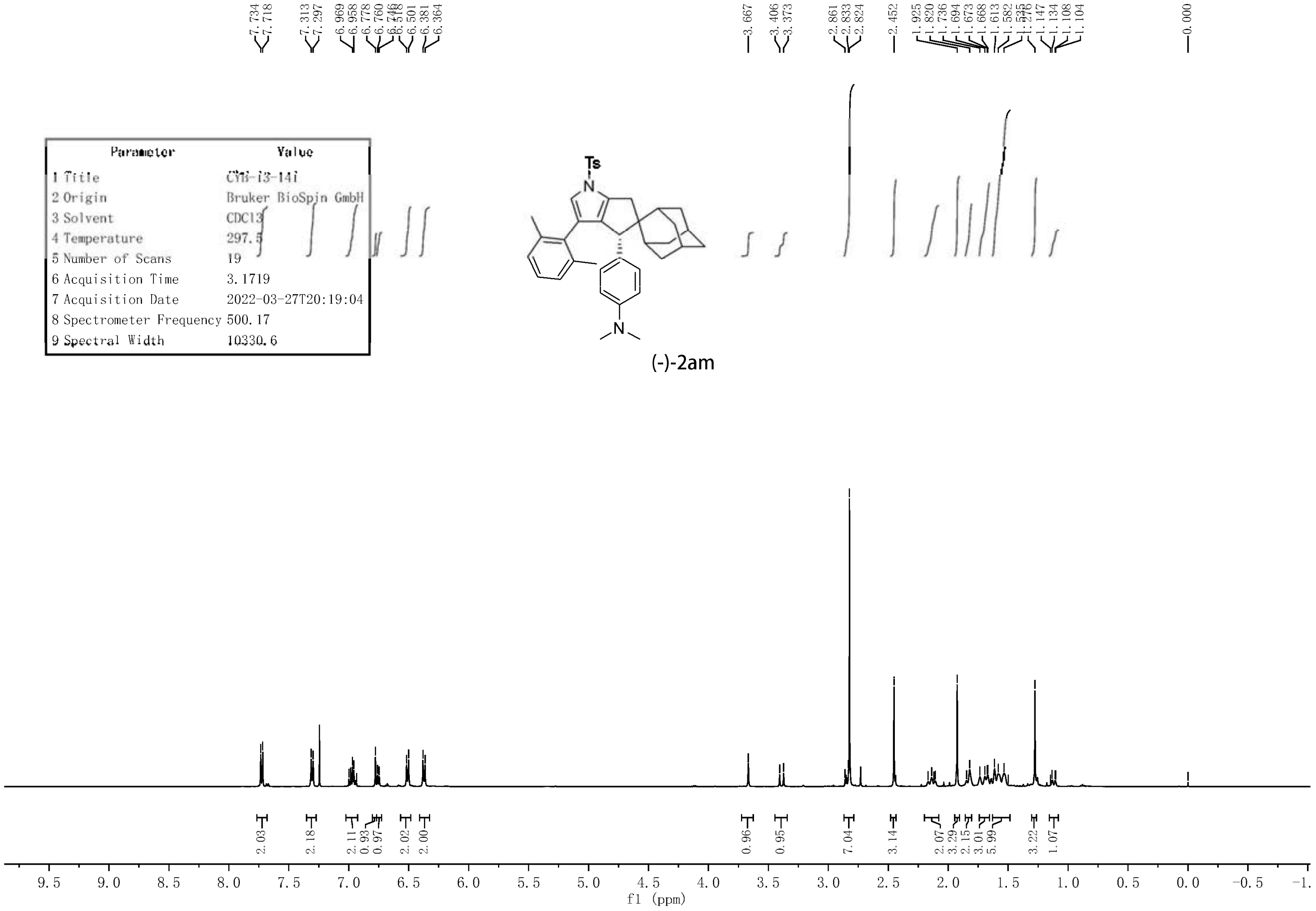

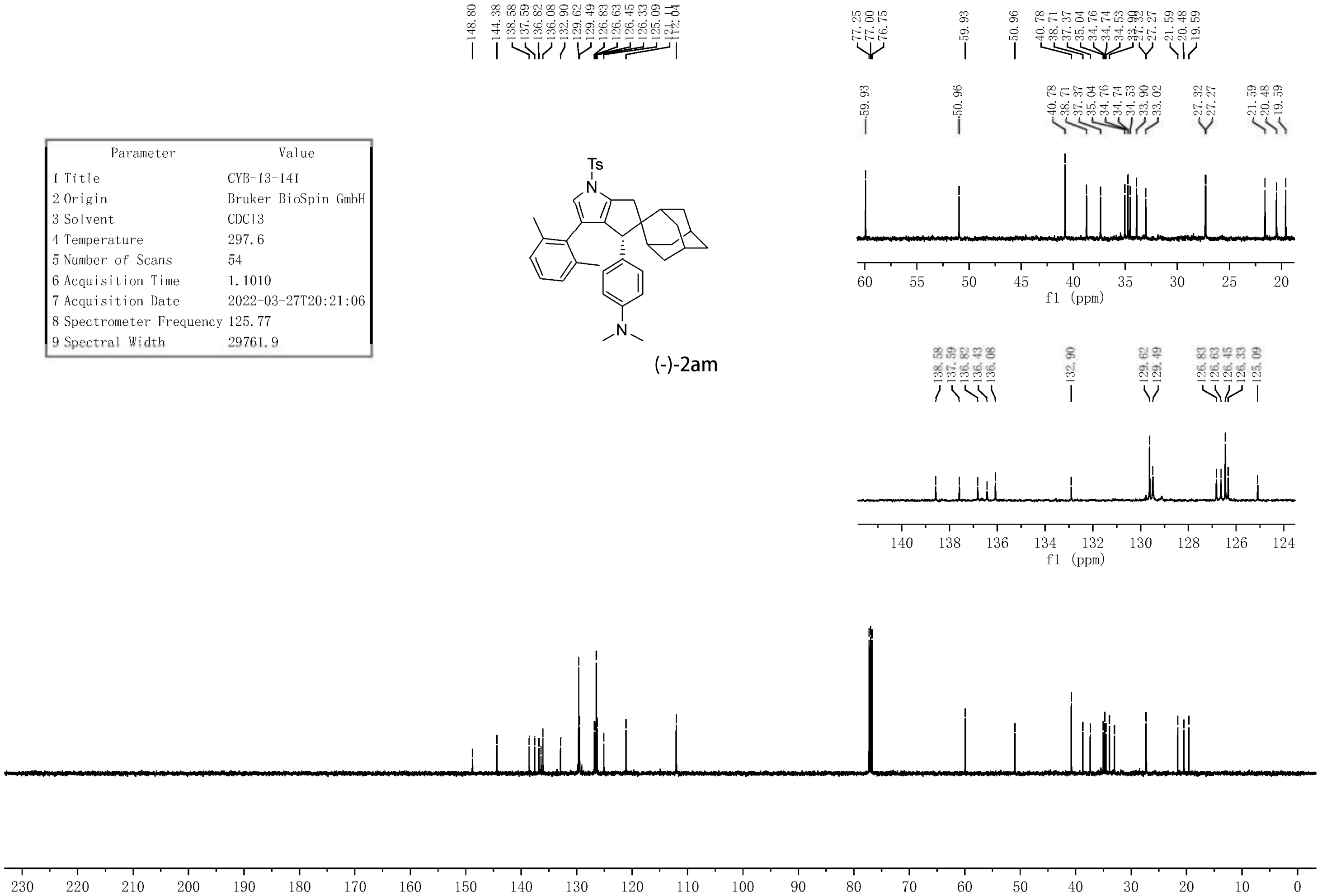

Supplementary Figure 207. ^{1}H and ^{13}C NMR spectra for (-)-2am

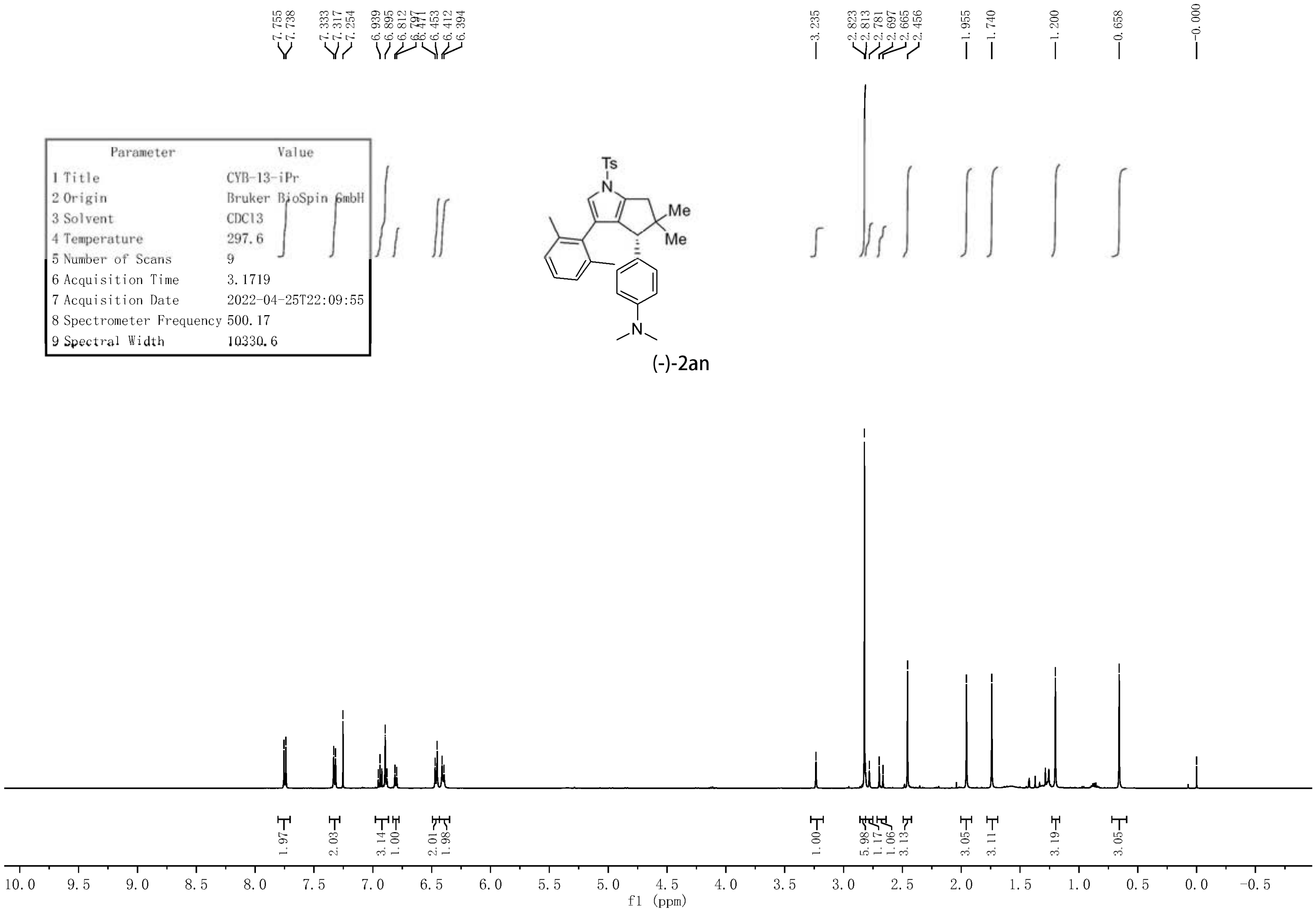

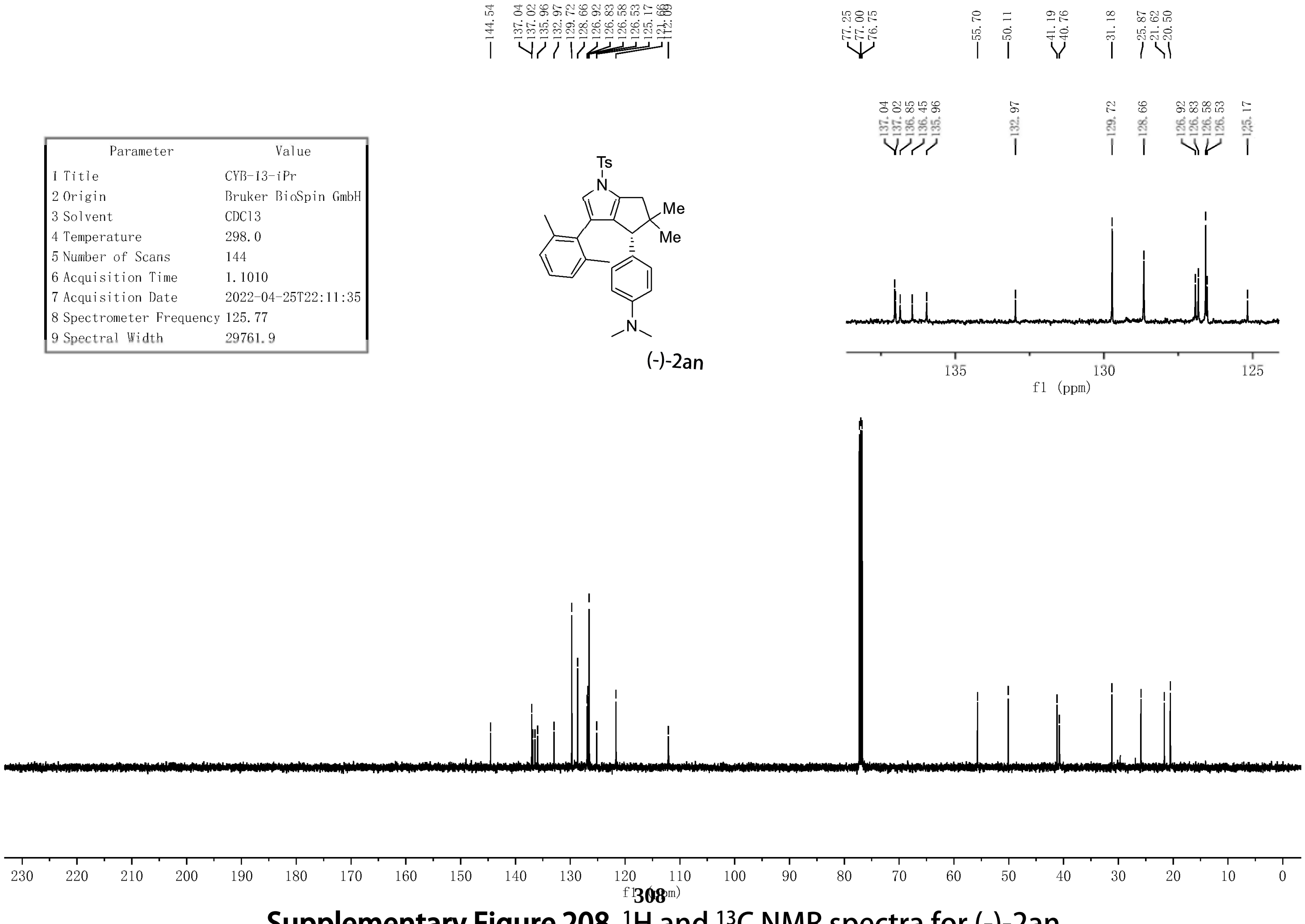

Supplementary Figure 208. ^{1}H and ^{13}C NMR spectra for (-)-2an

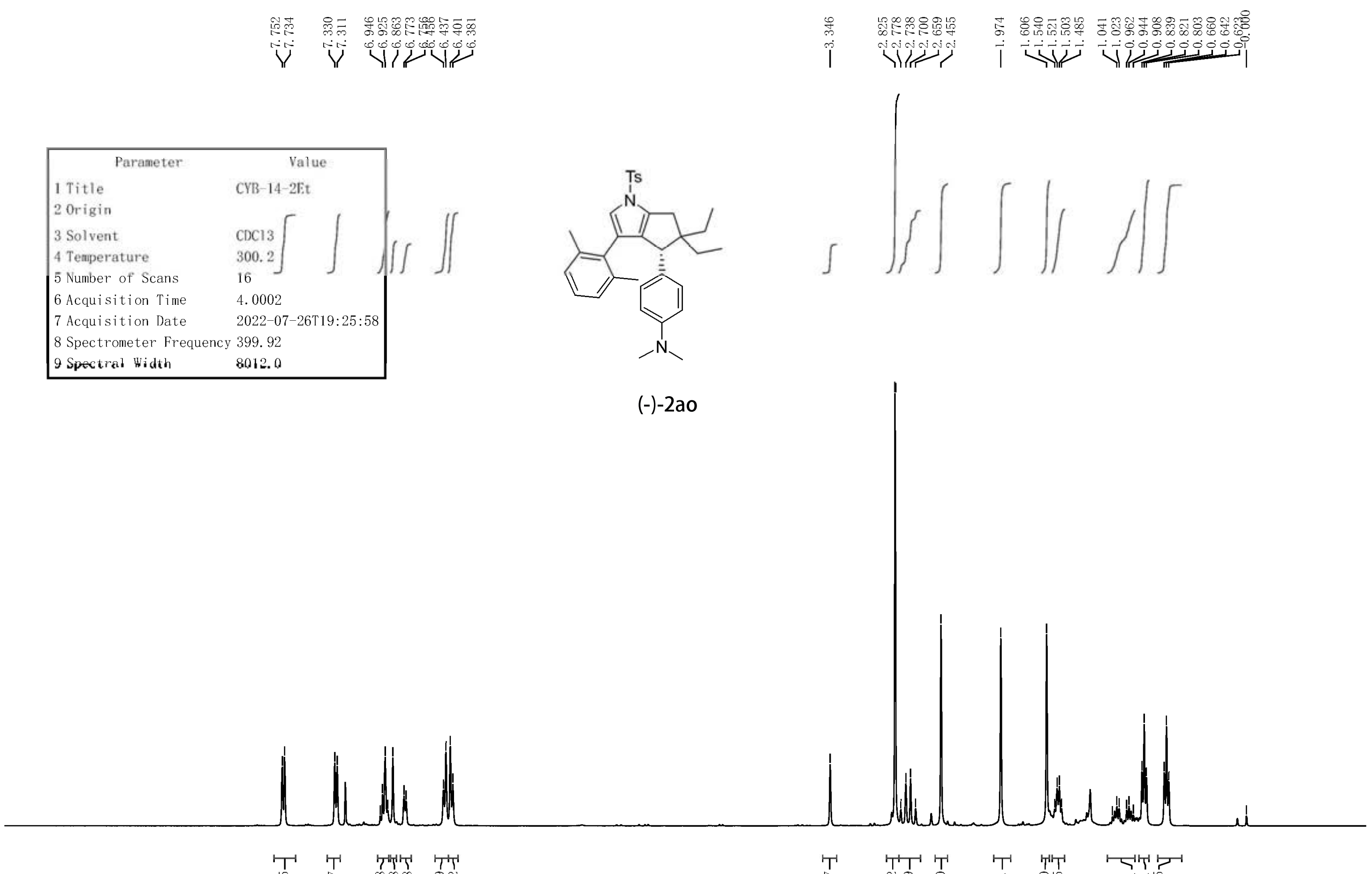

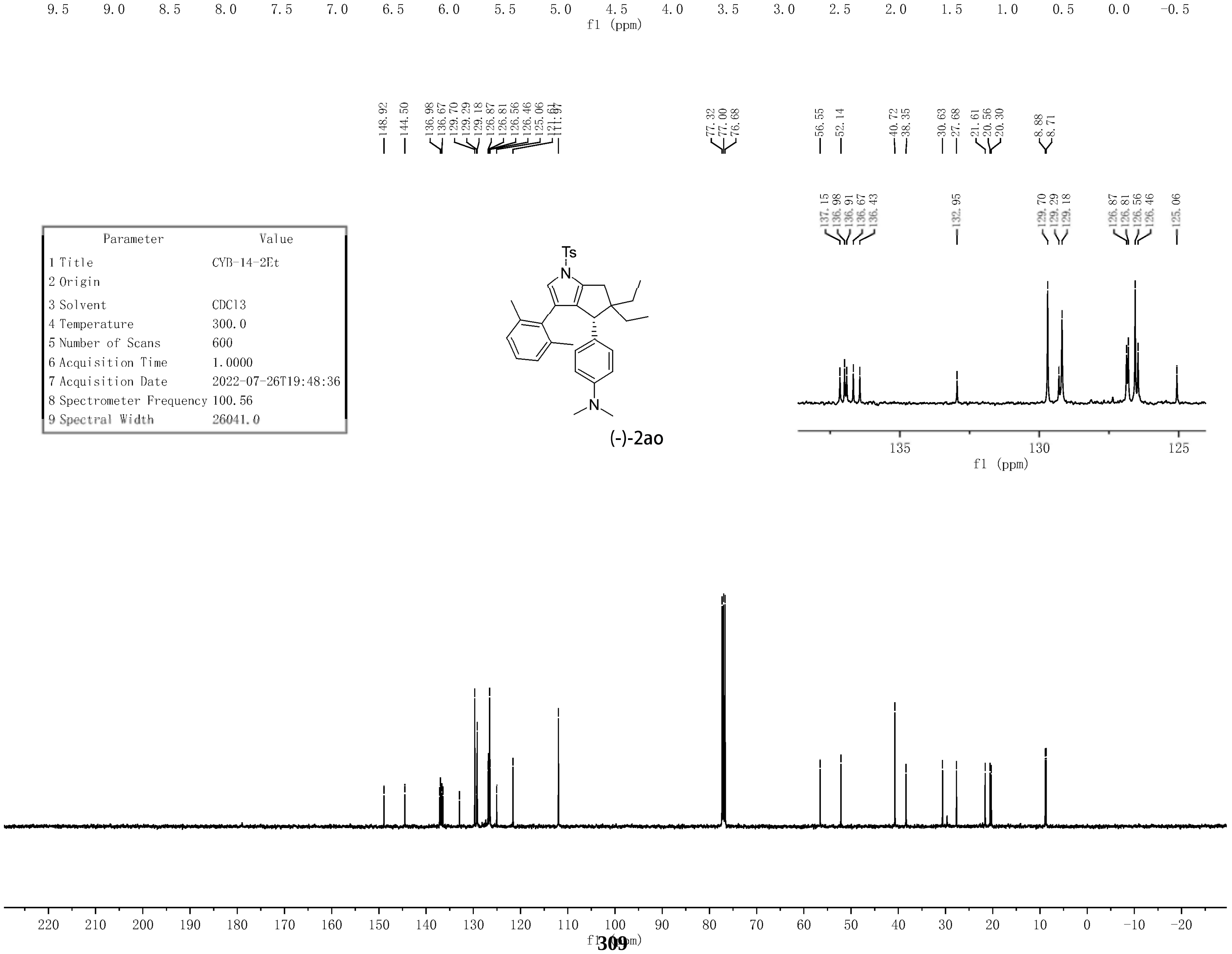

Supplementary Figure 209. ^{1}H and ^{13}C NMR spectra for (-)-2ao

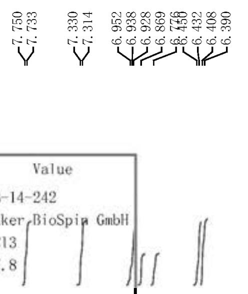

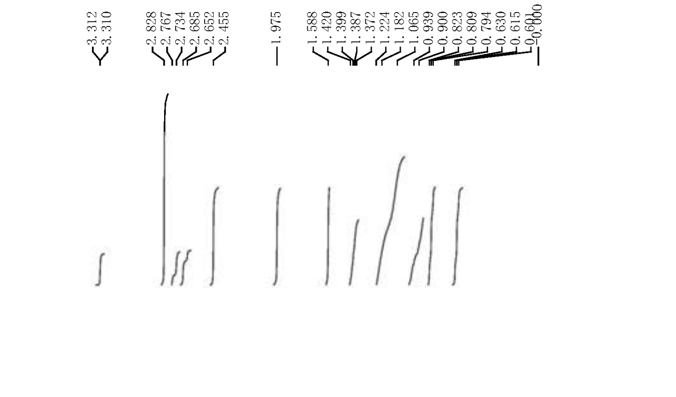

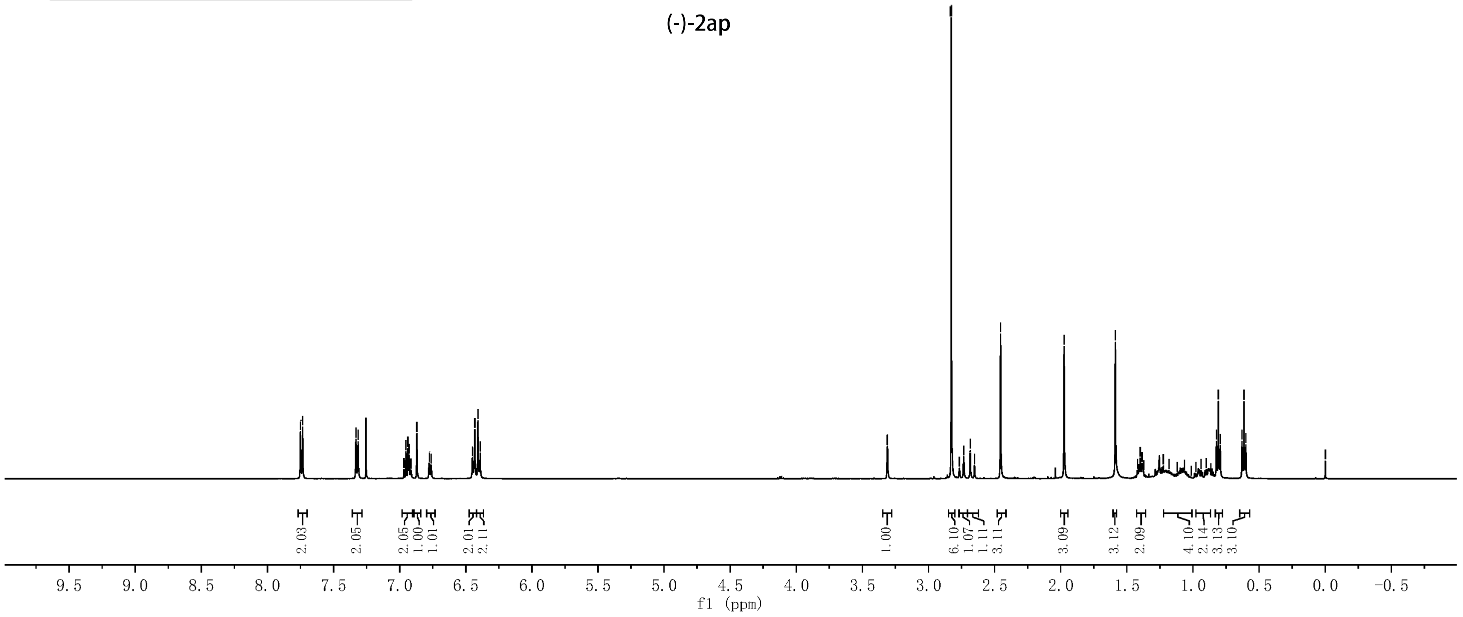

Parameter	Value
1 Title	CYB-14-242
2 Origin	Bruker BioSpin GmbH
3 Solvent	CDCl3
4 Temperature	297.8
5 Number of Scans	8
6 Acquisition Time	3.1719
7 Acquisition Date	2022-07-23T21:14:59
8 Spectrometer Frequency	500.17
9 Spectral Width	10330.6

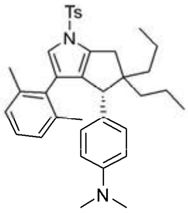

(-)-2ap

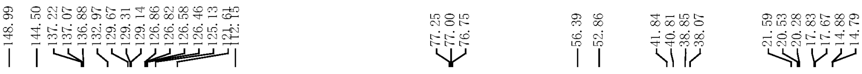

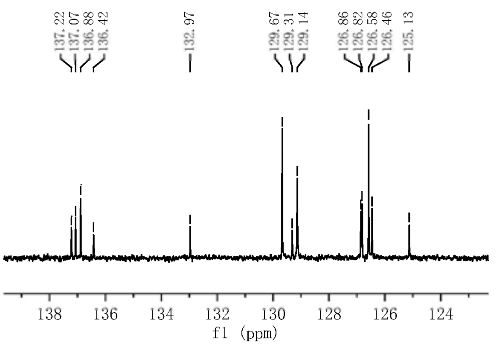

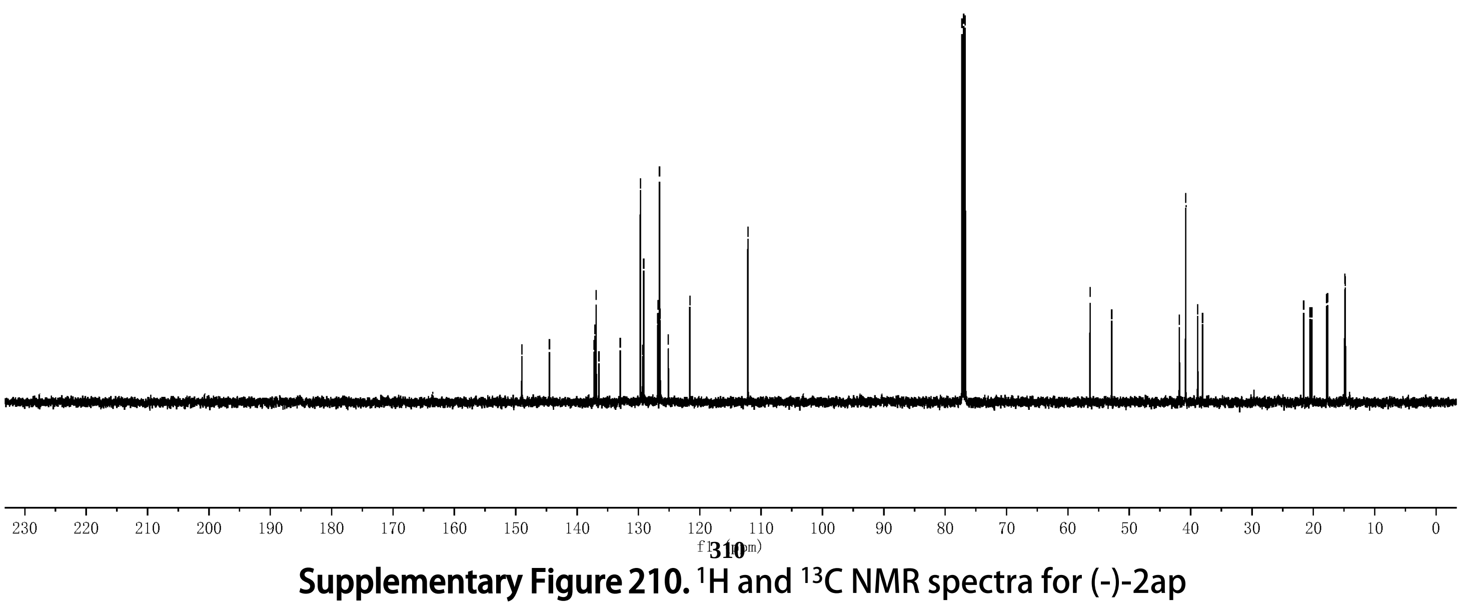

Parameter	Value
1 Title	CYB-14-242
2 Origin	Bruker BioSpin GmbH
3 Solvent	CDCl3
4 Temperature	298.1
5 Number of Scans	104
6 Acquisition Time	1.1010
7 Acquisition Date	2022-07-23T21:16:25
8 Spectrometer Frequency	125.77
9 Spectral Width	29761.9

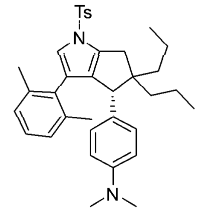

(-)-2ap

Supplementary Figure 210. ^{1}H and ^{13}C NMR spectra for (-)-2ap

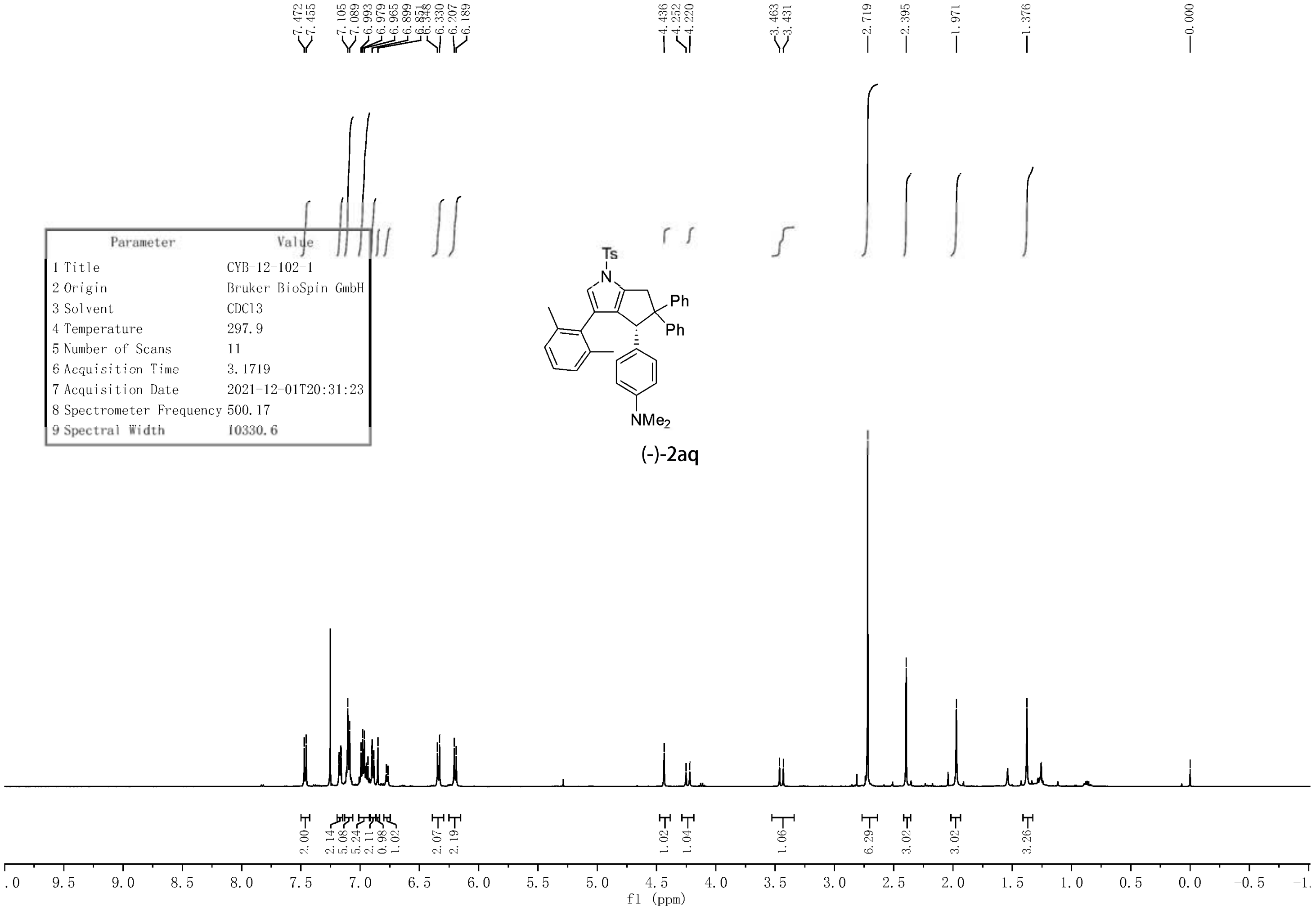

Parameter	Value
1 Title	CYB-12-102-1
2 Origin	Bruker BioSpin GmbH
3 Solvent	CDCl3
4 Temperature	297.9
5 Number of Scans	11
6 Acquisition Time	3.1719
7 Acquisition Date	2021-12-01T20:31:23
8 Spectrometer Frequency	500.17
9 Spectral Width	10330.6

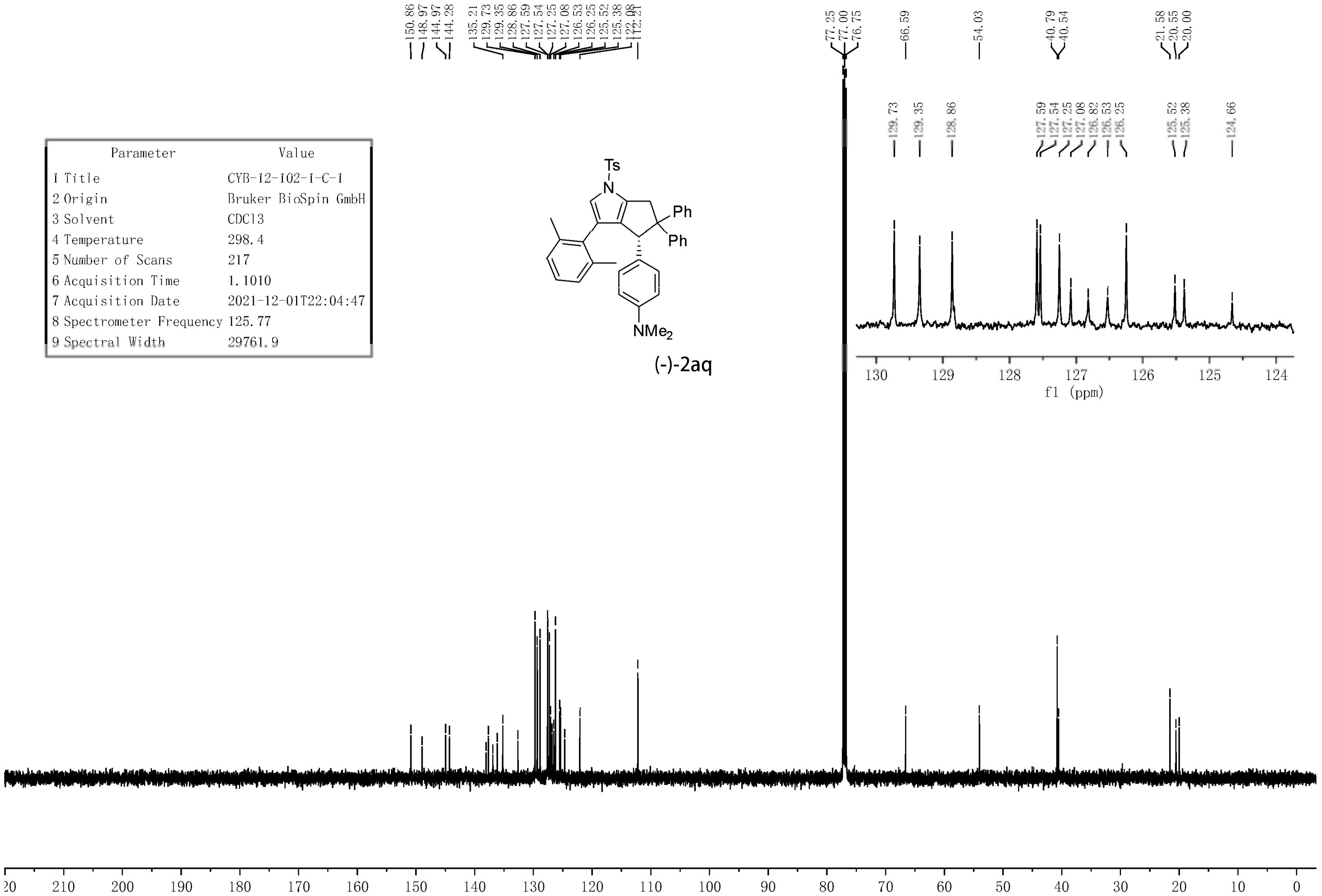

Parameter	Value
1 Title	CYB-12-102-1-C-1
2 Origin	Bruker BioSpin GmbH
3 Solvent	CDCl3
4 Temperature	298.4
5 Number of Scans	217
6 Acquisition Time	1.1010
7 Acquisition Date	2021-12-01T22:04:47
8 Spectrometer Frequency	125.77
9 Spectral Width	29761.9

Supplementary Figure 211. ^{1}H and ^{13}C NMR spectra for (-)-2aq

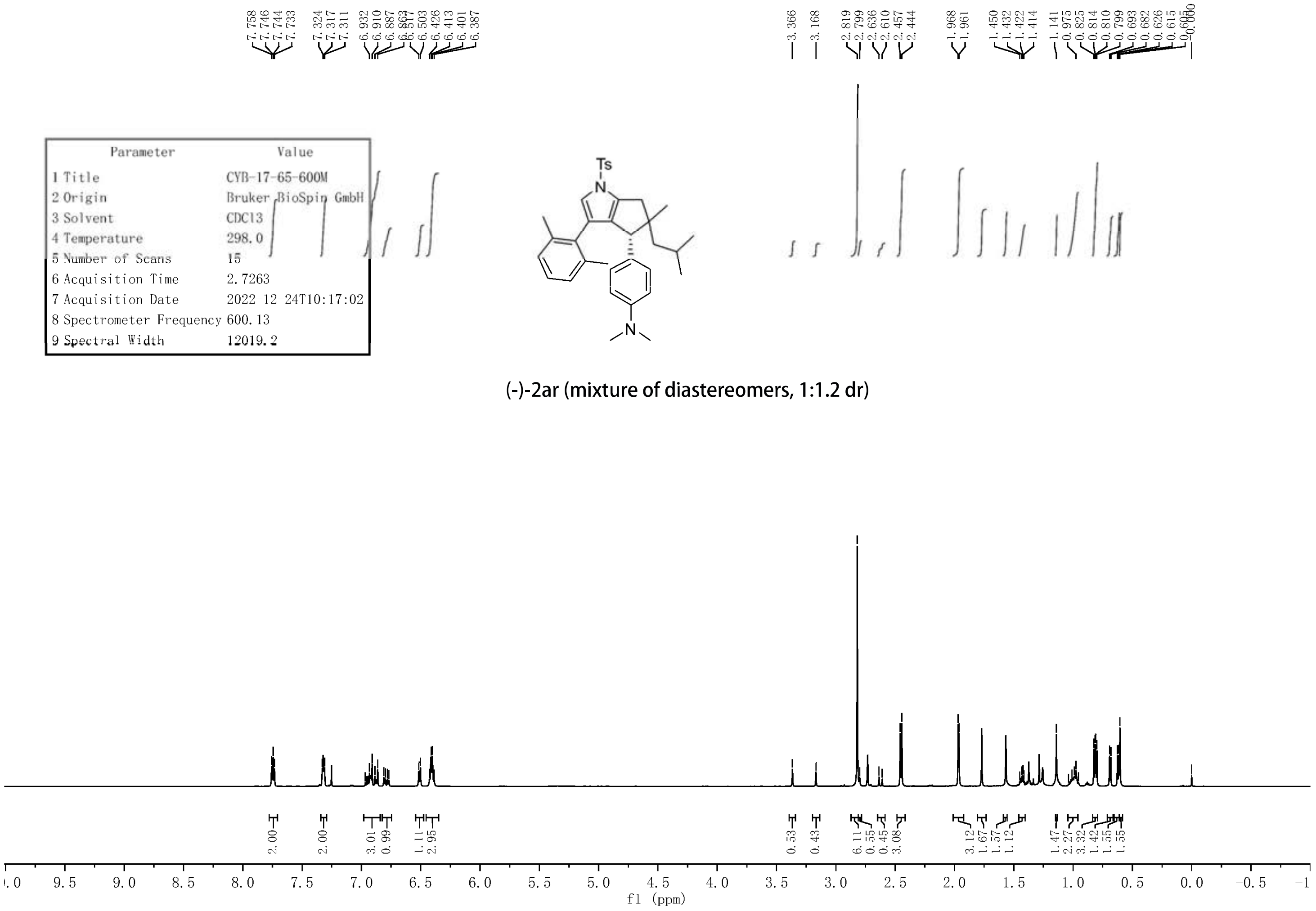

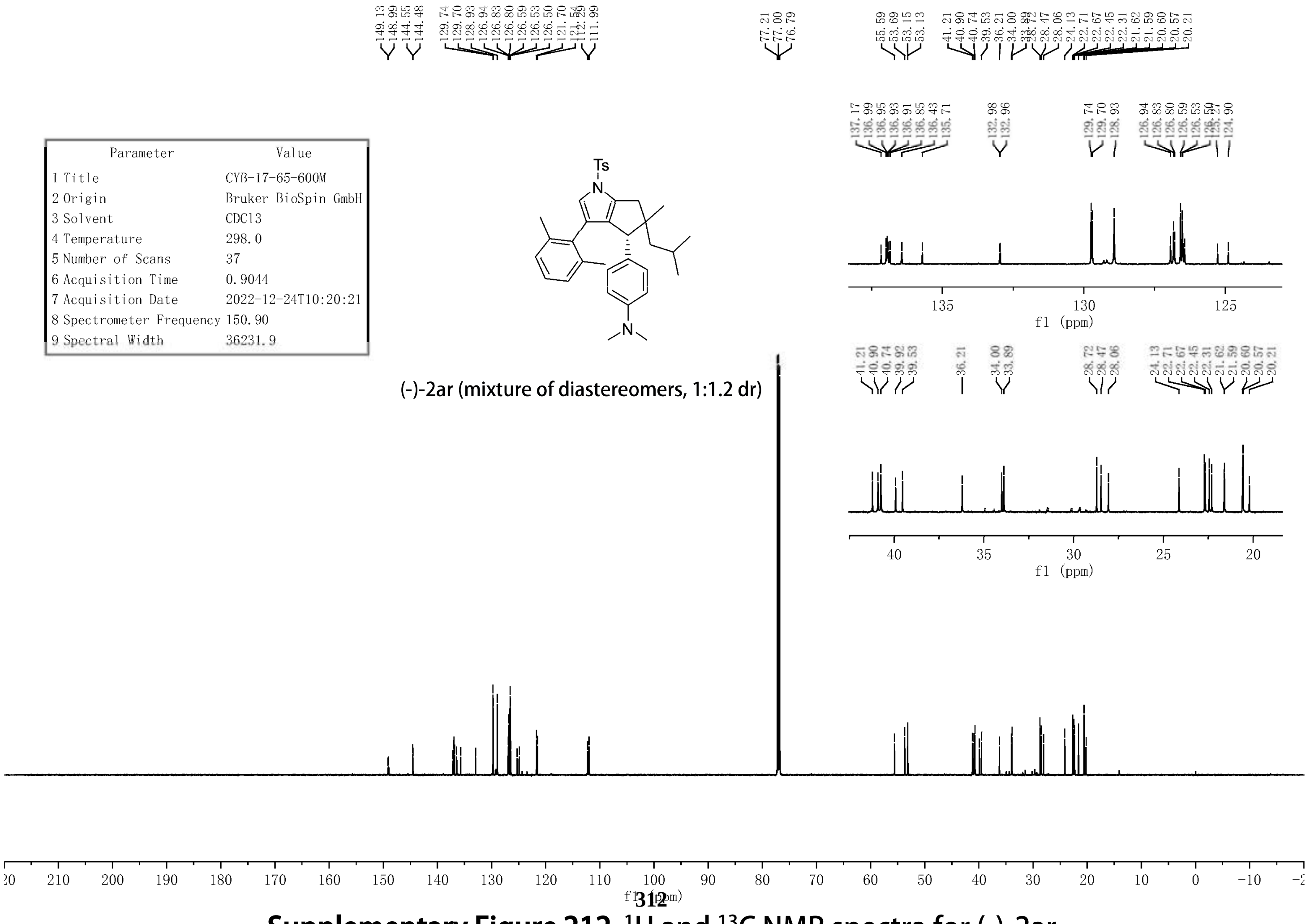

Supplementary Figure 212. ^{1}H and ^{13}C NMR spectra for (-)-2ar

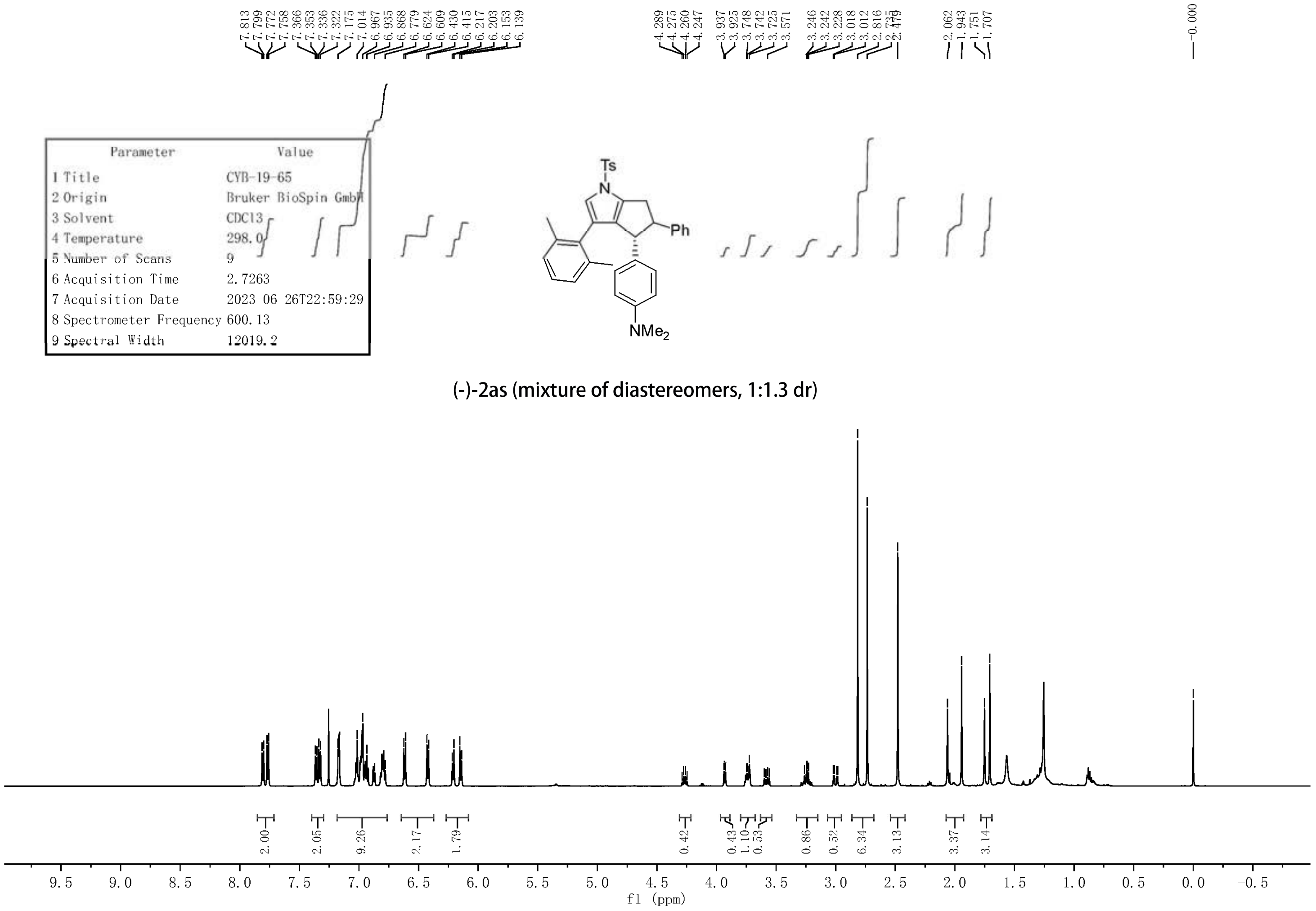

(-)-2as (mixture of diastereomers, 1:1.3 dr)

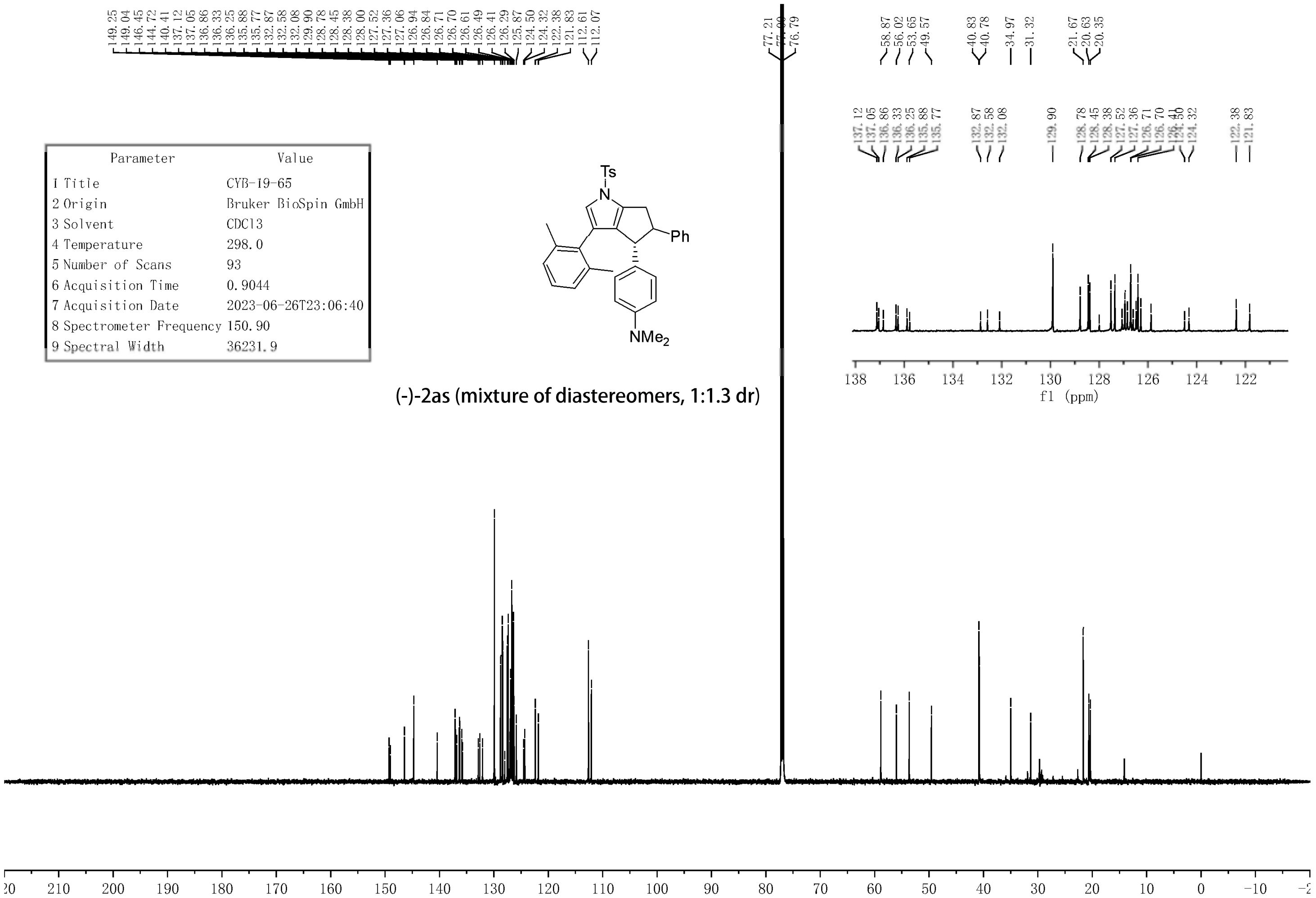

(-)-2as (mixture of diastereomers, 1:1.3 dr)

Supplementary Figure 213. ^{1}H and ^{13}C NMR spectra for (-)-2as

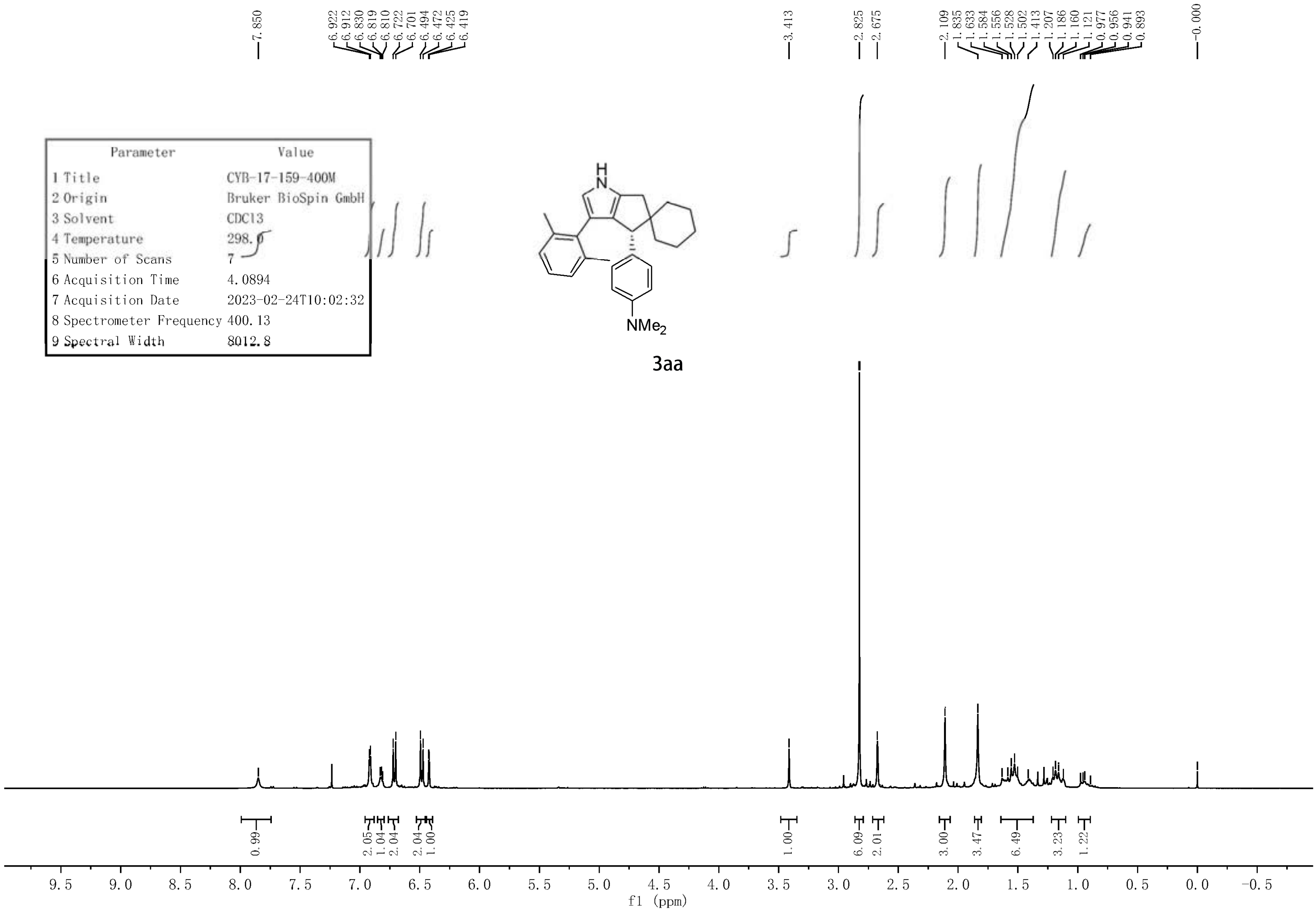

Parameter	Value
1 Title	CYB-17-159-400M
2 Origin	Bruker BioSpin GmbH
3 Solvent	CDCl3
4 Temperature	298.0
5 Number of Scans	7
6 Acquisition Time	4.0894
7 Acquisition Date	2023-02-24T10:02:32
8 Spectrometer Frequency	400.13
9 Spectral Width	8012.8

3aa

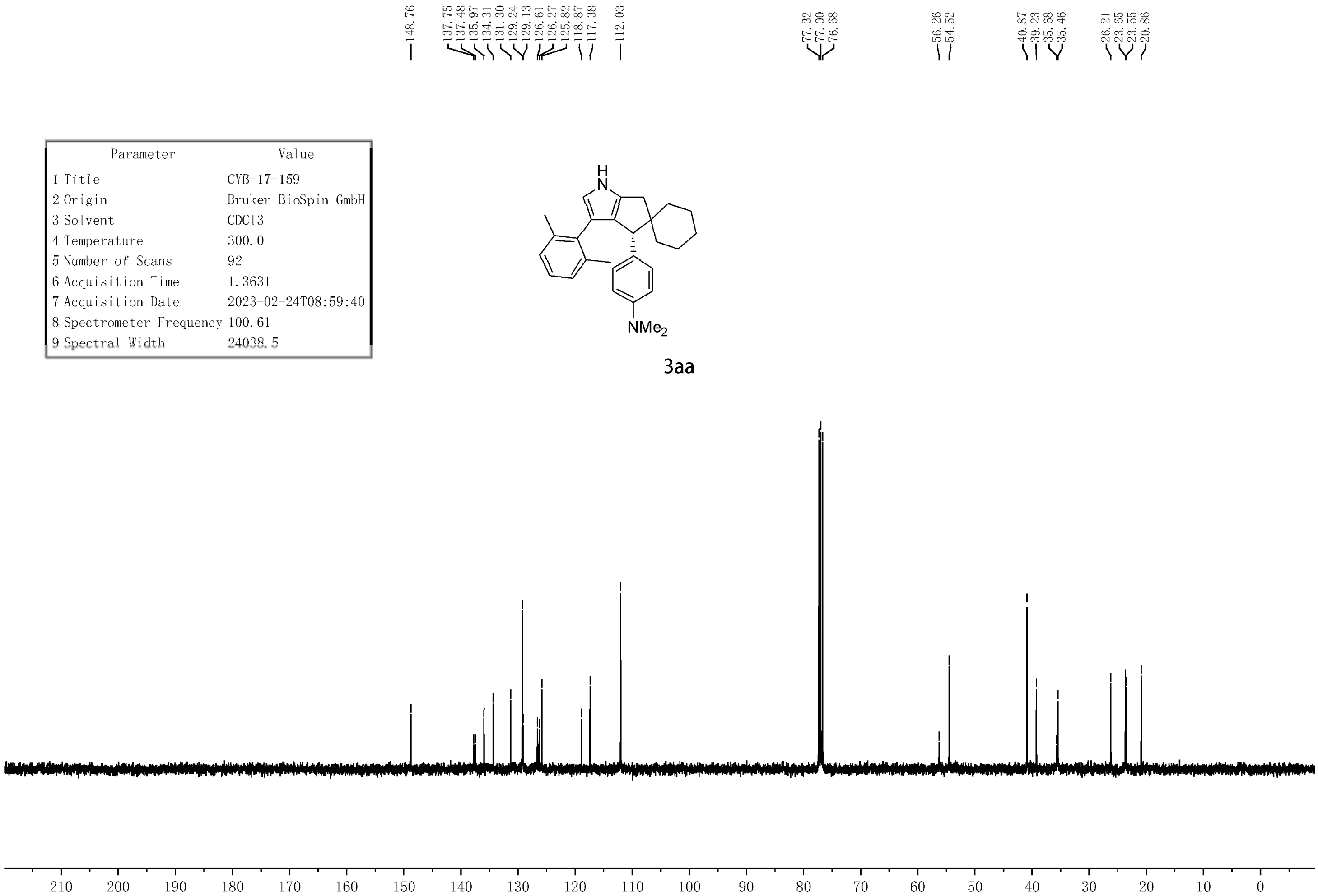

Parameter	Value
1 Title	CYB-17-159
2 Origin	Bruker BioSpin GmbH
3 Solvent	CDCl3
4 Temperature	300.0
5 Number of Scans	92
6 Acquisition Time	1.3631
7 Acquisition Date	2023-02-24T08:59:40
8 Spectrometer Frequency	100.61
9 Spectral Width	24038.5

3aa

Supplementary Figure 214. ^{1}H and ^{13}C NMR spectra for 3aa

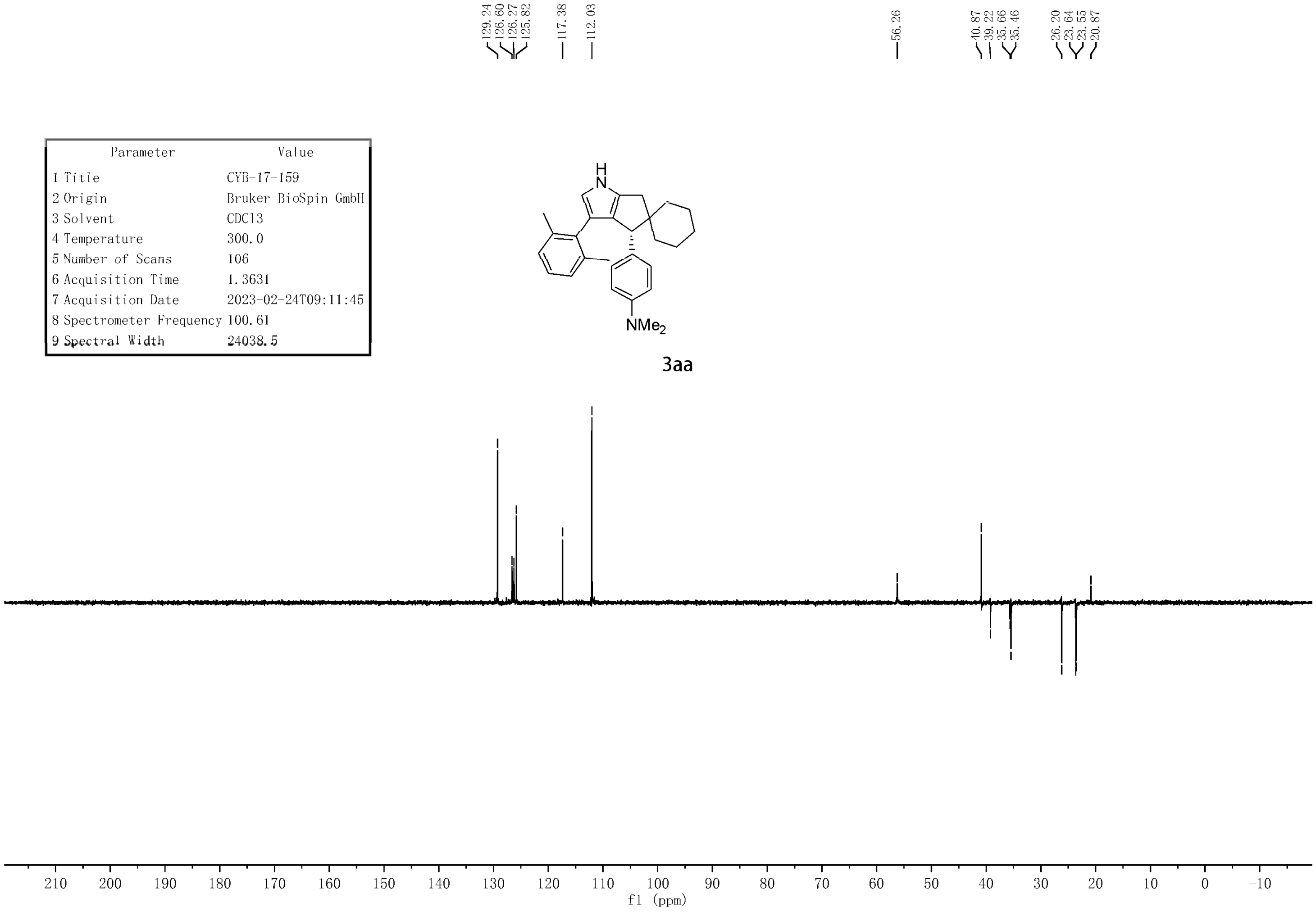

Supplementary Figure 215. DEPT-135 NMR spectra for 3aa

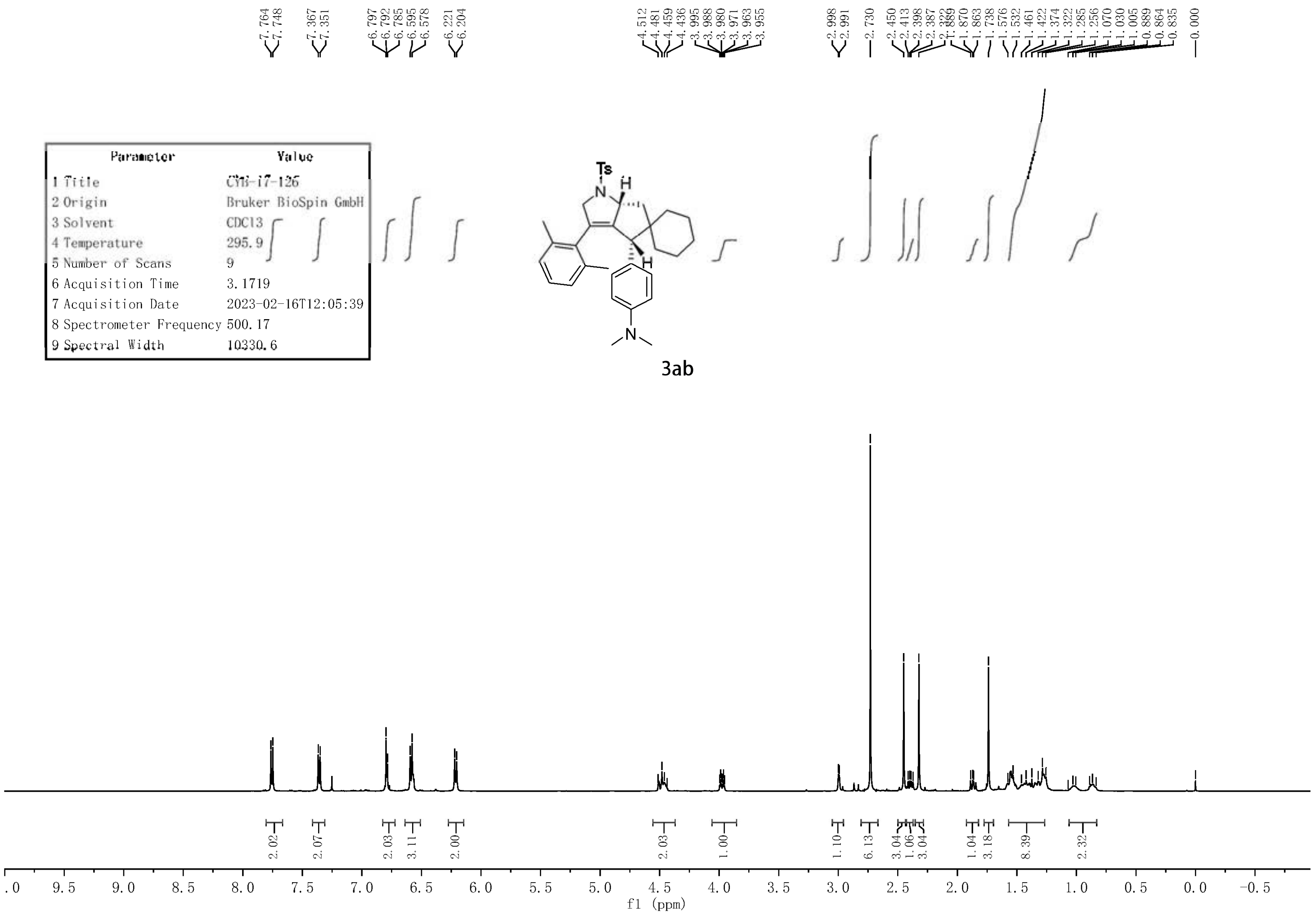

Parameter	Value
1 Title	CYB-17-126
2 Origin	Bruker BioSpin GmbH
3 Solvent	CDCl3
4 Temperature	295.9
5 Number of Scans	9
6 Acquisition Time	3.1719
7 Acquisition Date	2023-02-16T12:05:39
8 Spectrometer Frequency	500.17
9 Spectral Width	10330.6

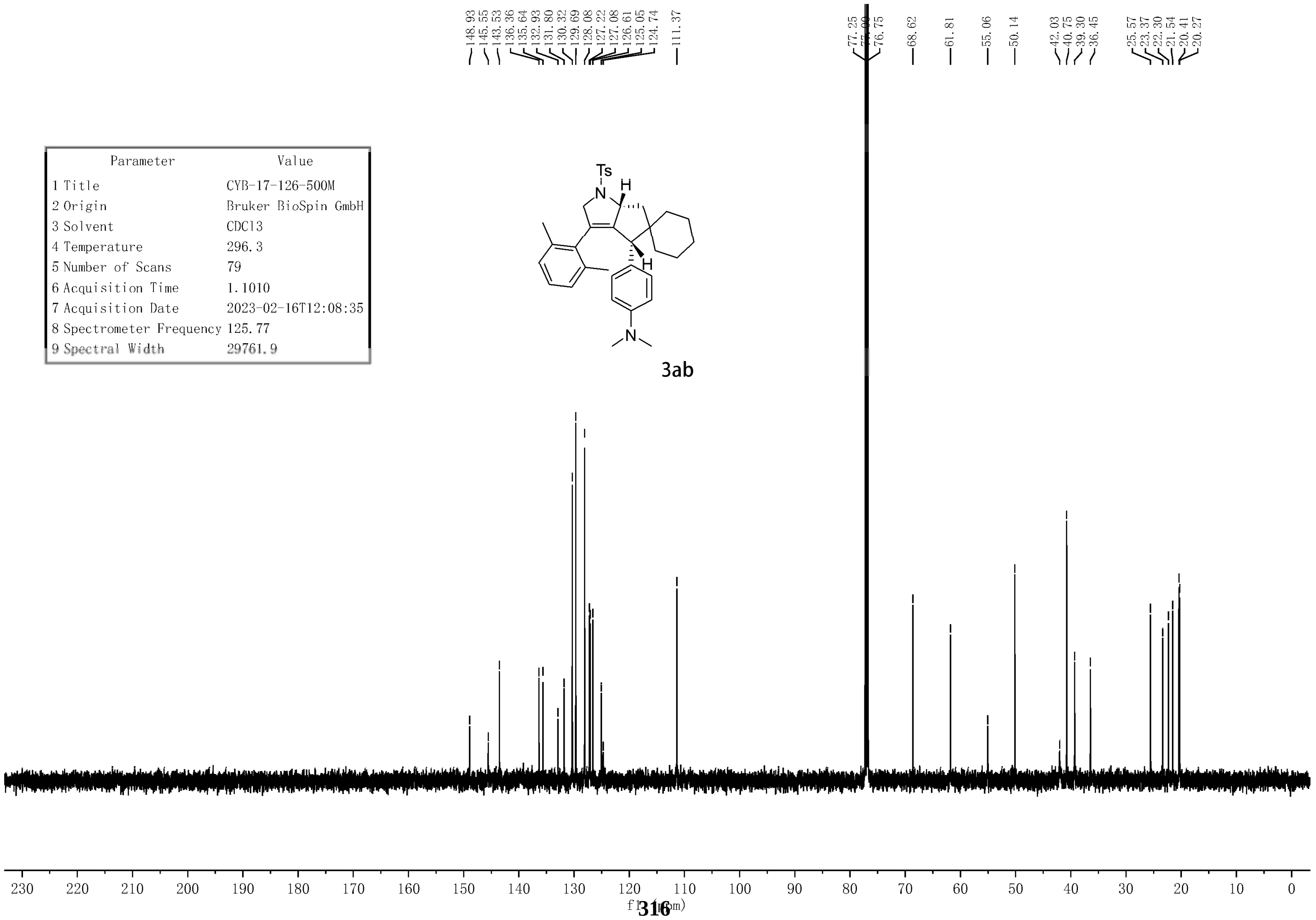

Parameter	Value
1 Title	CYB-17-126-500M
2 Origin	Bruker BioSpin GmbH
3 Solvent	CDCl3
4 Temperature	296.3
5 Number of Scans	79
6 Acquisition Time	1.1010
7 Acquisition Date	2023-02-16T12:08:35
8 Spectrometer Frequency	125.77
9 Spectral Width	29761.9

Supplementary Figure 216. ^{1}H and ^{13}C NMR spectra for 3ab

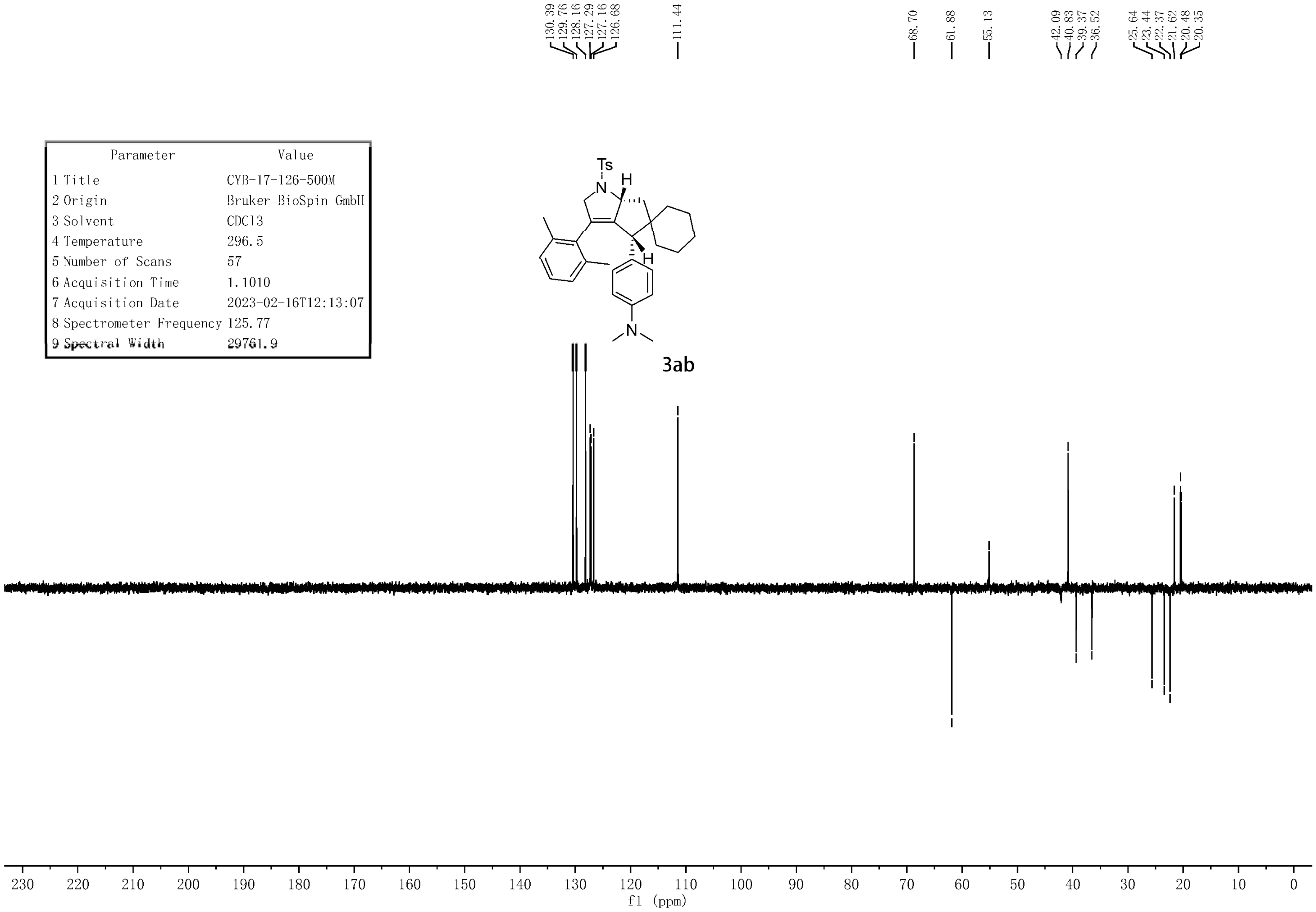

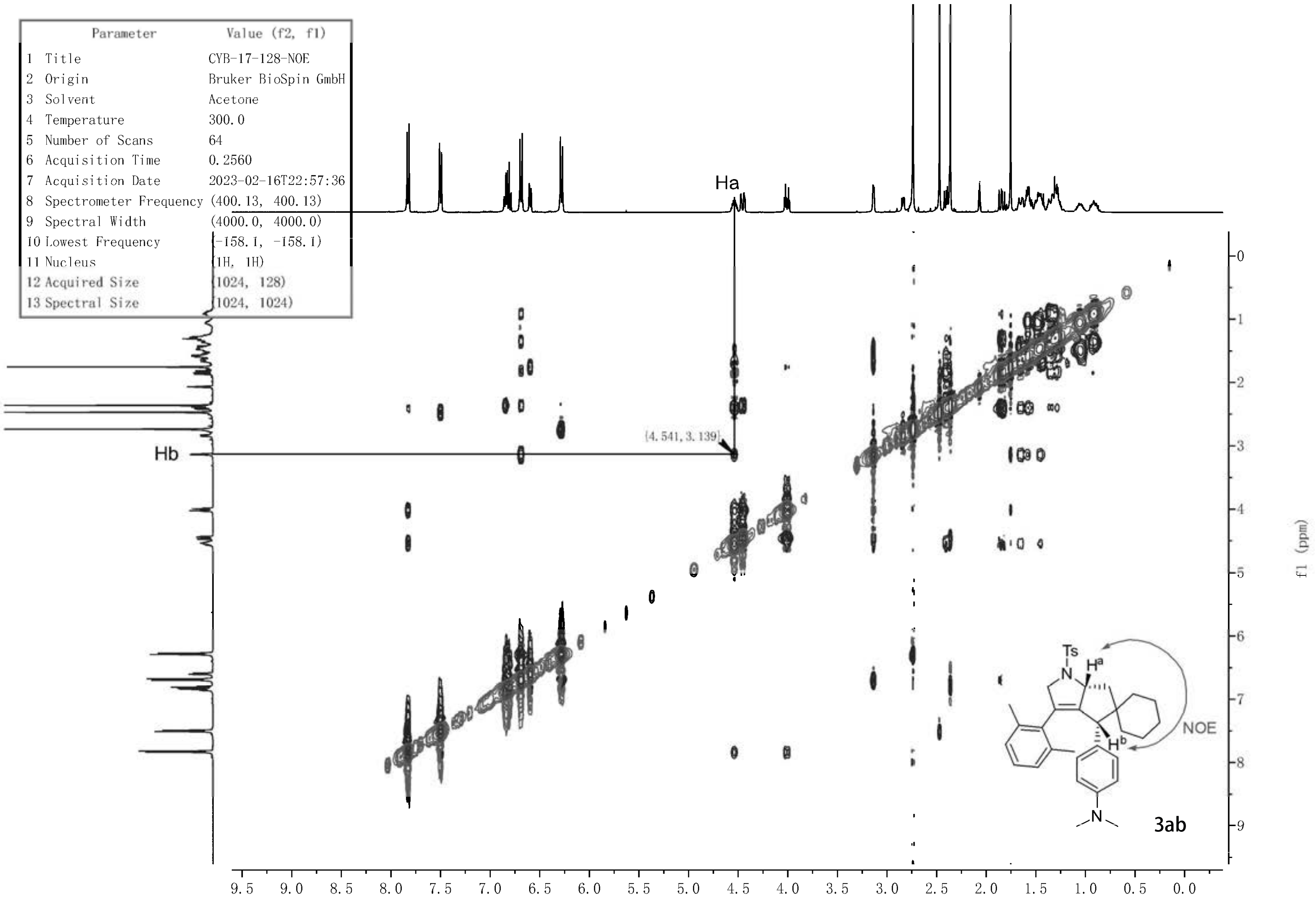

Supplementary Figure 217. DEPT-135 and NOESY NMR spectra for 3ab

Parameter	Value
1 Title	CYB-17-116-H
2 Origin	Bruker BioSpin GmbH
3 Solvent	CDCl3
4 Temperature	296.3
5 Number of Scans	4
6 Acquisition Time	3.1719
7 Acquisition Date	2023-02-14T21:39:55
8 Spectrometer Frequency	500.17
9 Spectral Width	10330.6

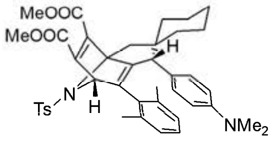

3ac

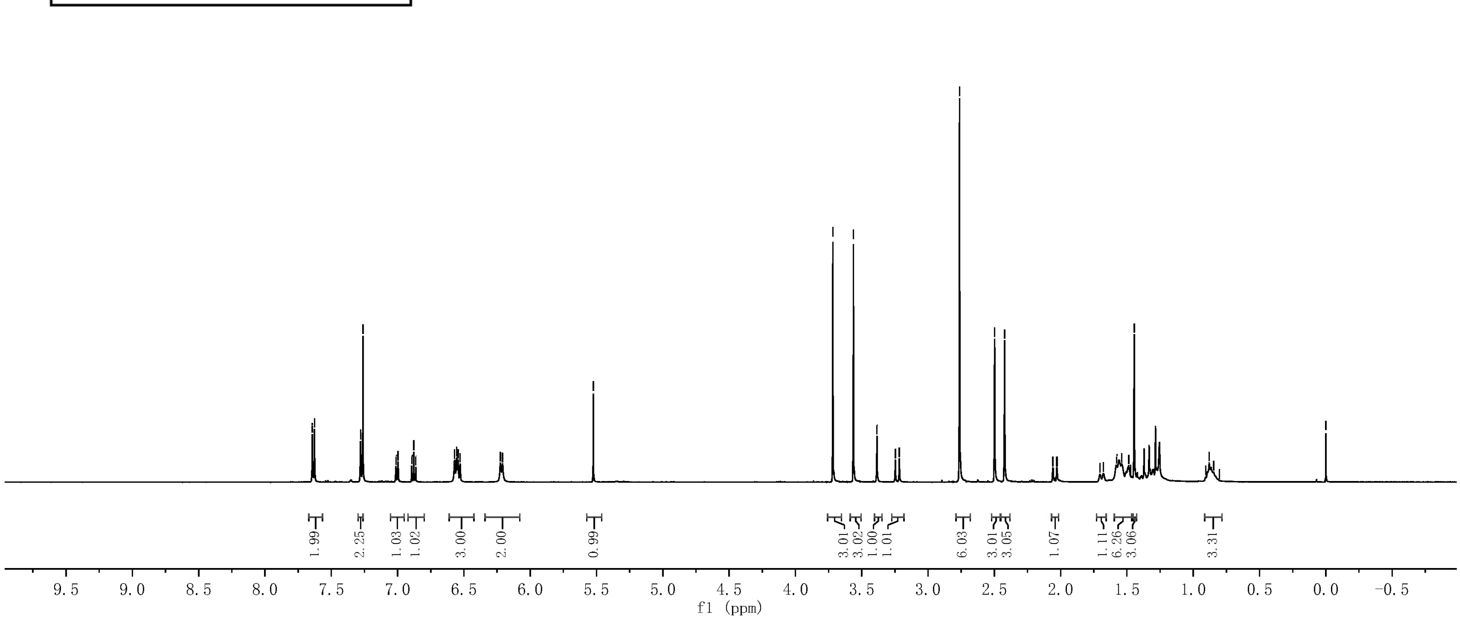

Parameter	Value
1 Title	CYB-17-139-400M
2 Origin	Bruker BioSpin GmbH
3 Solvent	CDCl3
4 Temperature	300.0
5 Number of Scans	1024
6 Acquisition Time	1.3631
7 Acquisition Date	2023-02-20T23:24:43
8 Spectrometer Frequency	100.61
9 Spectral Width	24038.5

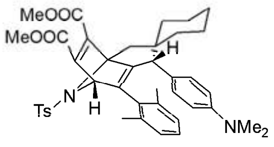

3ac

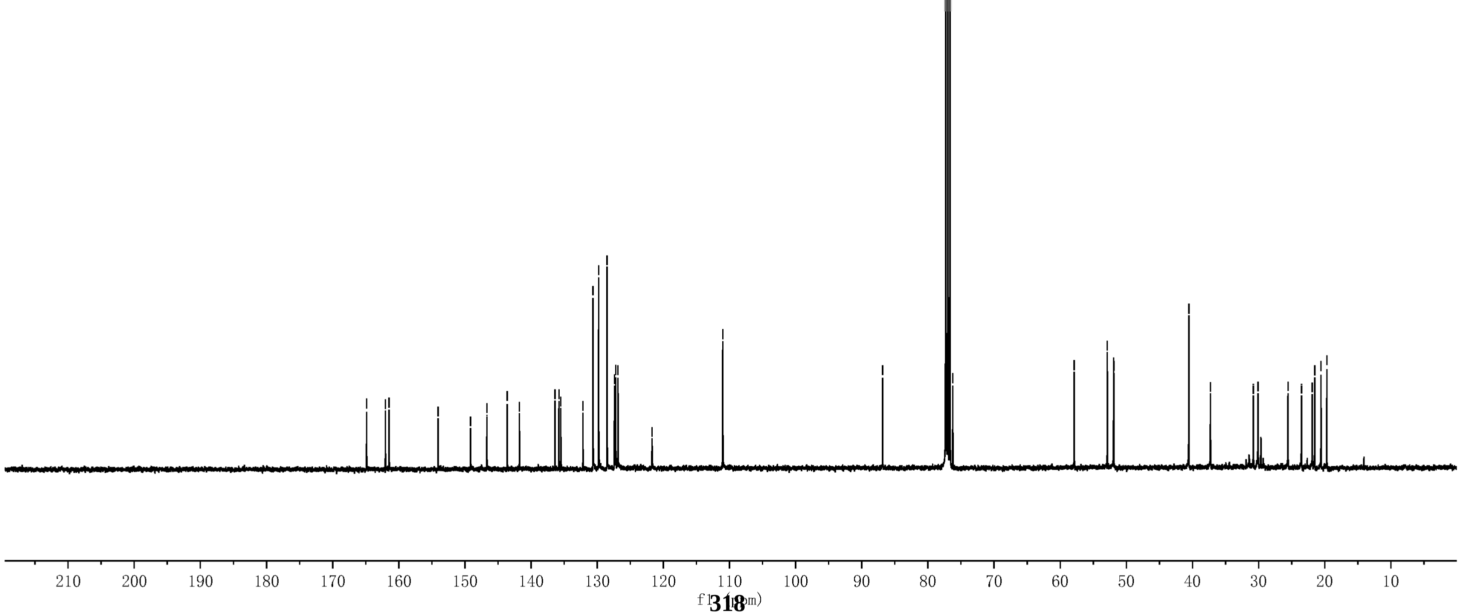

Supplementary Figure 218. ^{1}H and ^{13}C NMR spectra for 3ac

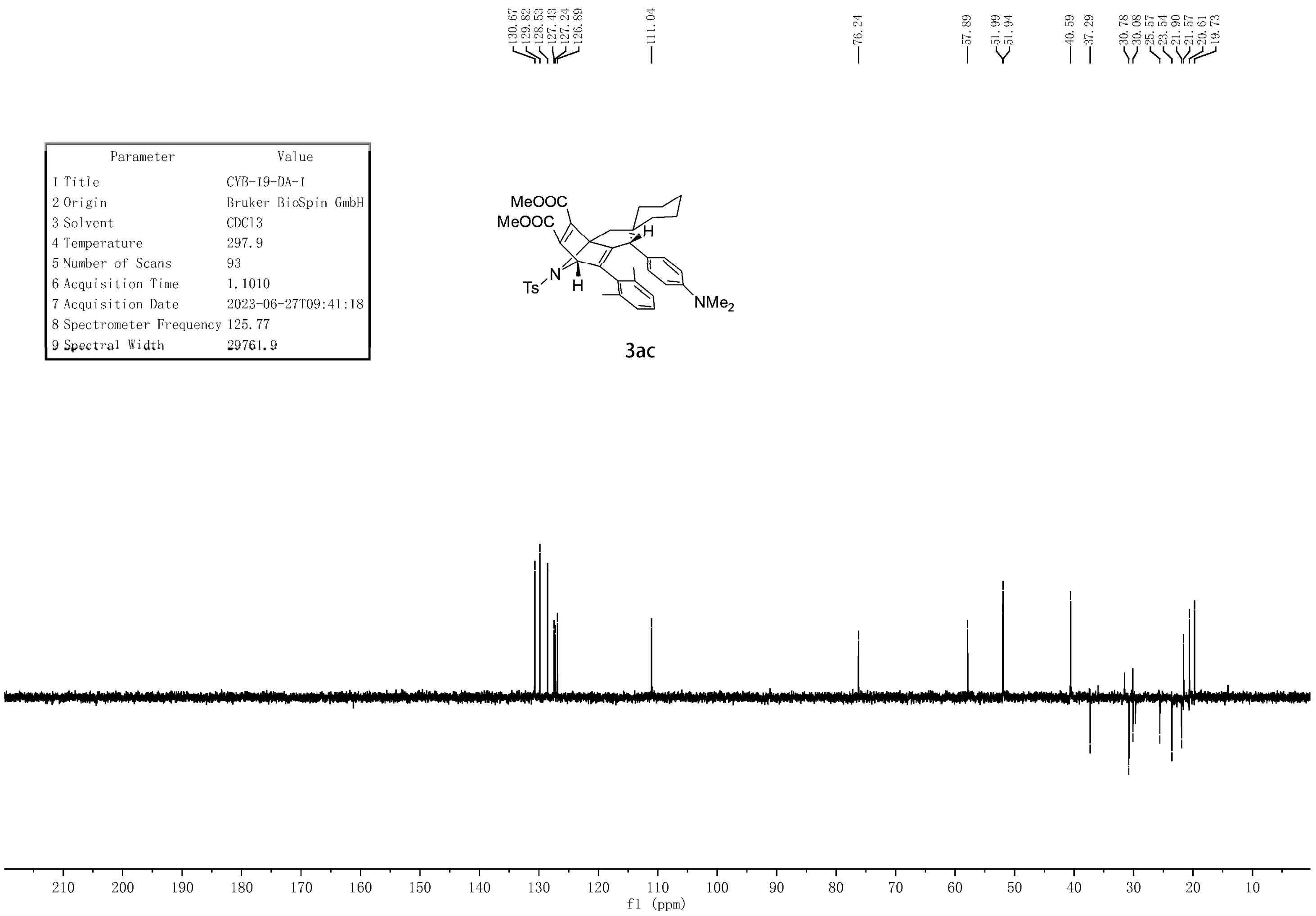

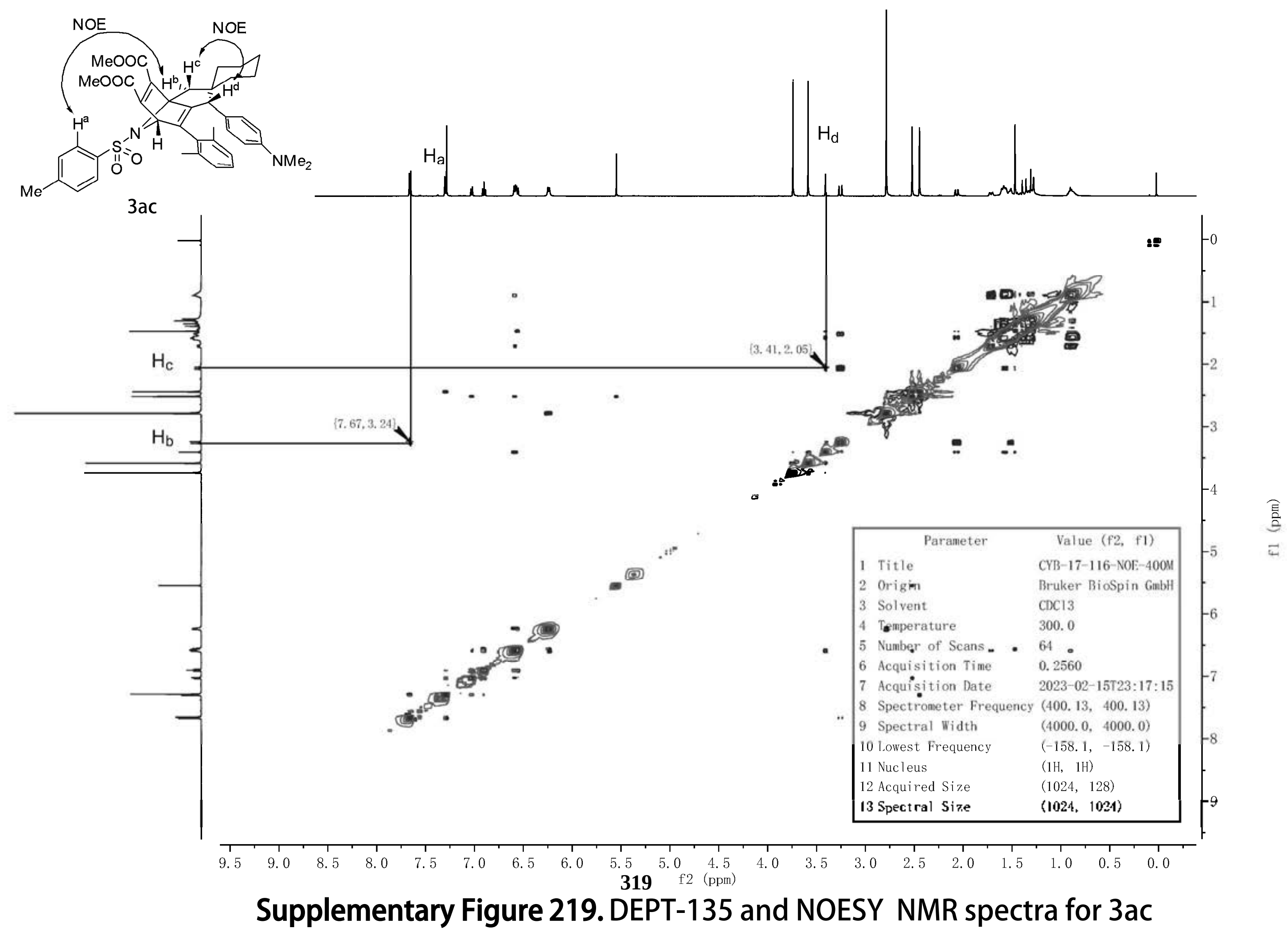

Supplementary Figure 219. DEPT-135 and NOESY NMR spectra for 3ac

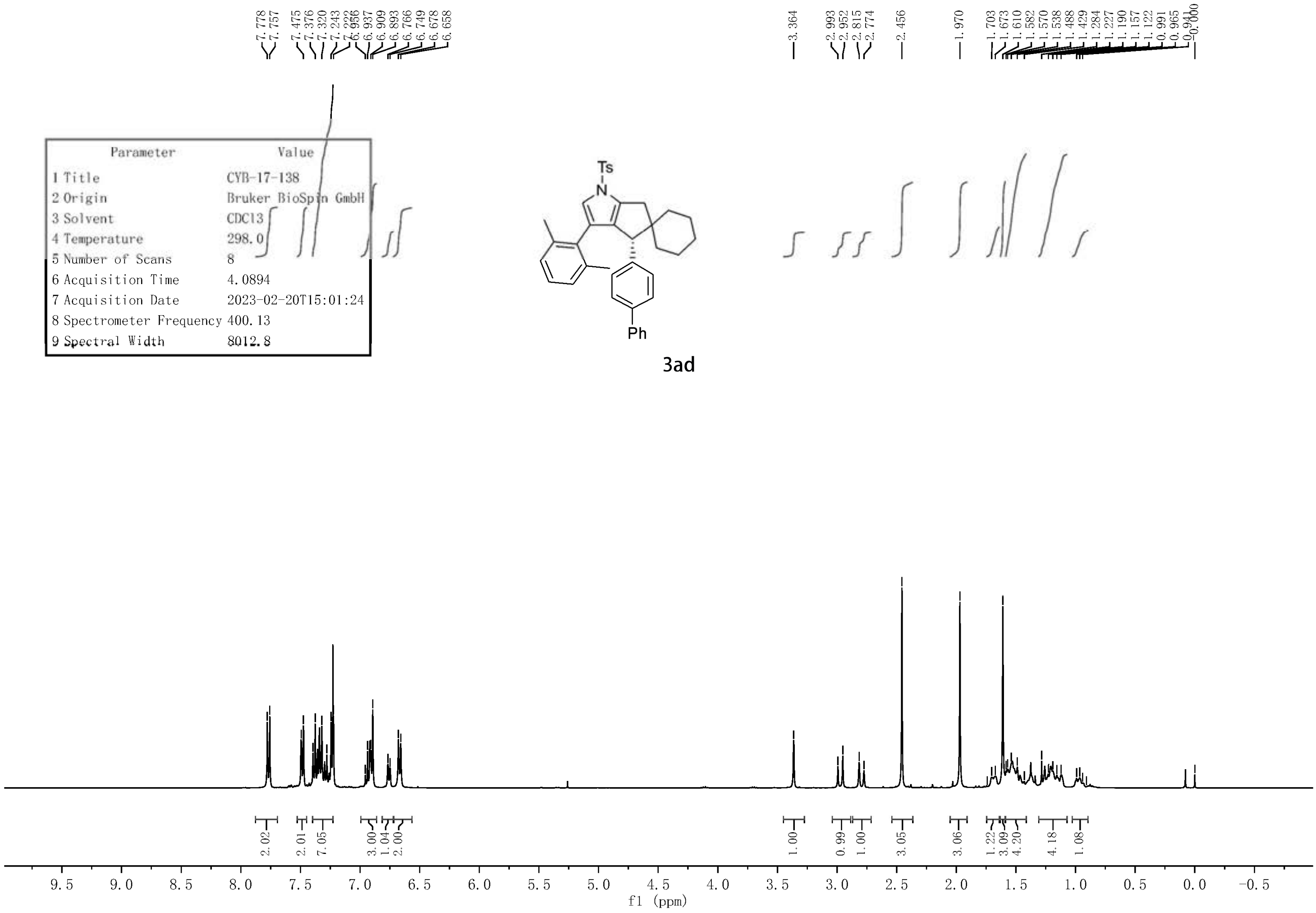

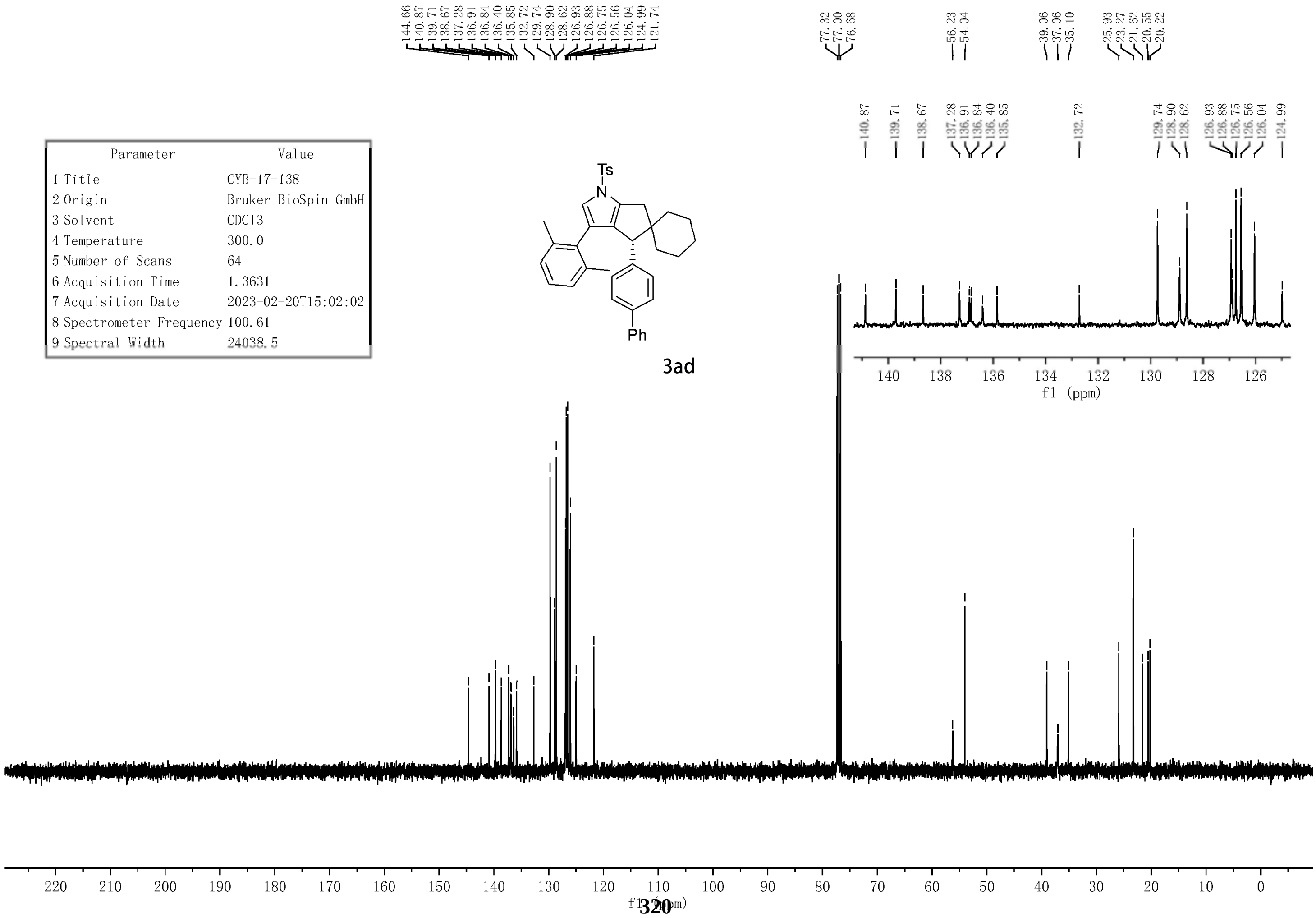

Supplementary Figure 220. ^{1}H and ^{13}C NMR spectra for 3ad

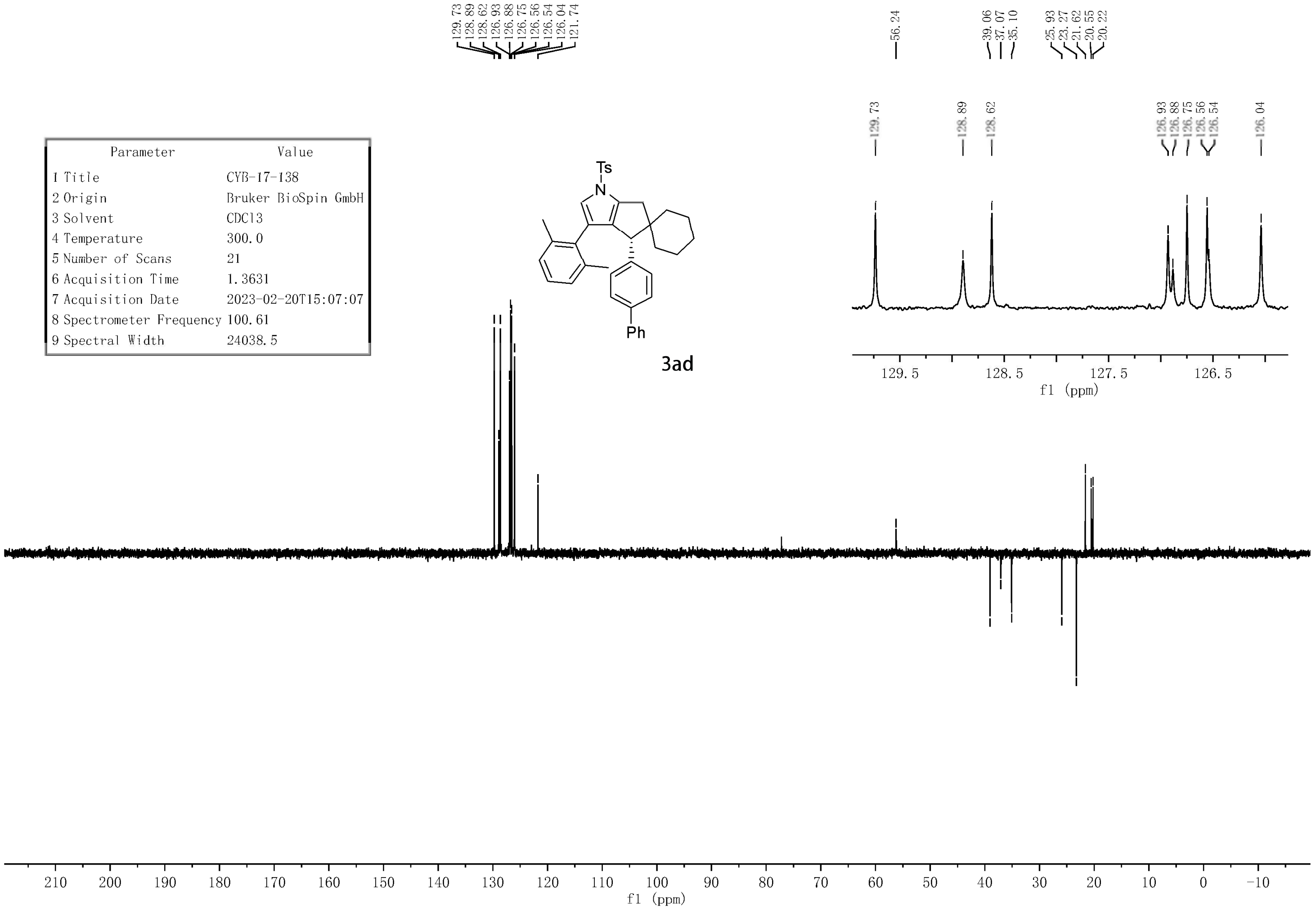

Parameter	Value
1 Title	CYB-17-138
2 Origin	Bruker BioSpin GmbH
3 Solvent	CDCl3
4 Temperature	300.0
5 Number of Scans	21
6 Acquisition Time	1.3631
7 Acquisition Date	2023-02-20T15:07:07
8 Spectrometer Frequency	100.61
9 Spectral Width	24038.5

Supplementary Figure 221. DEPT-135 NMR spectra for 3ad

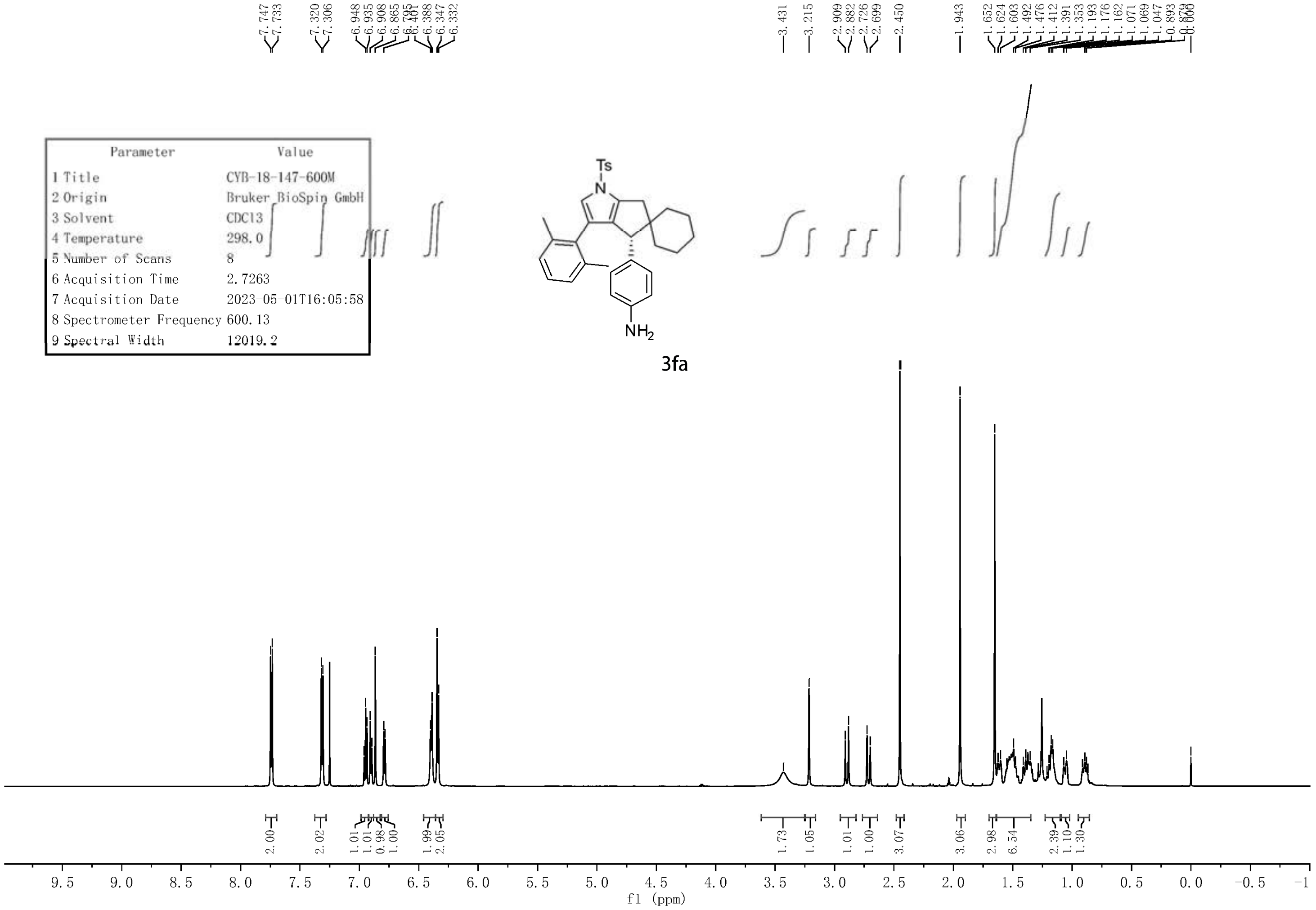

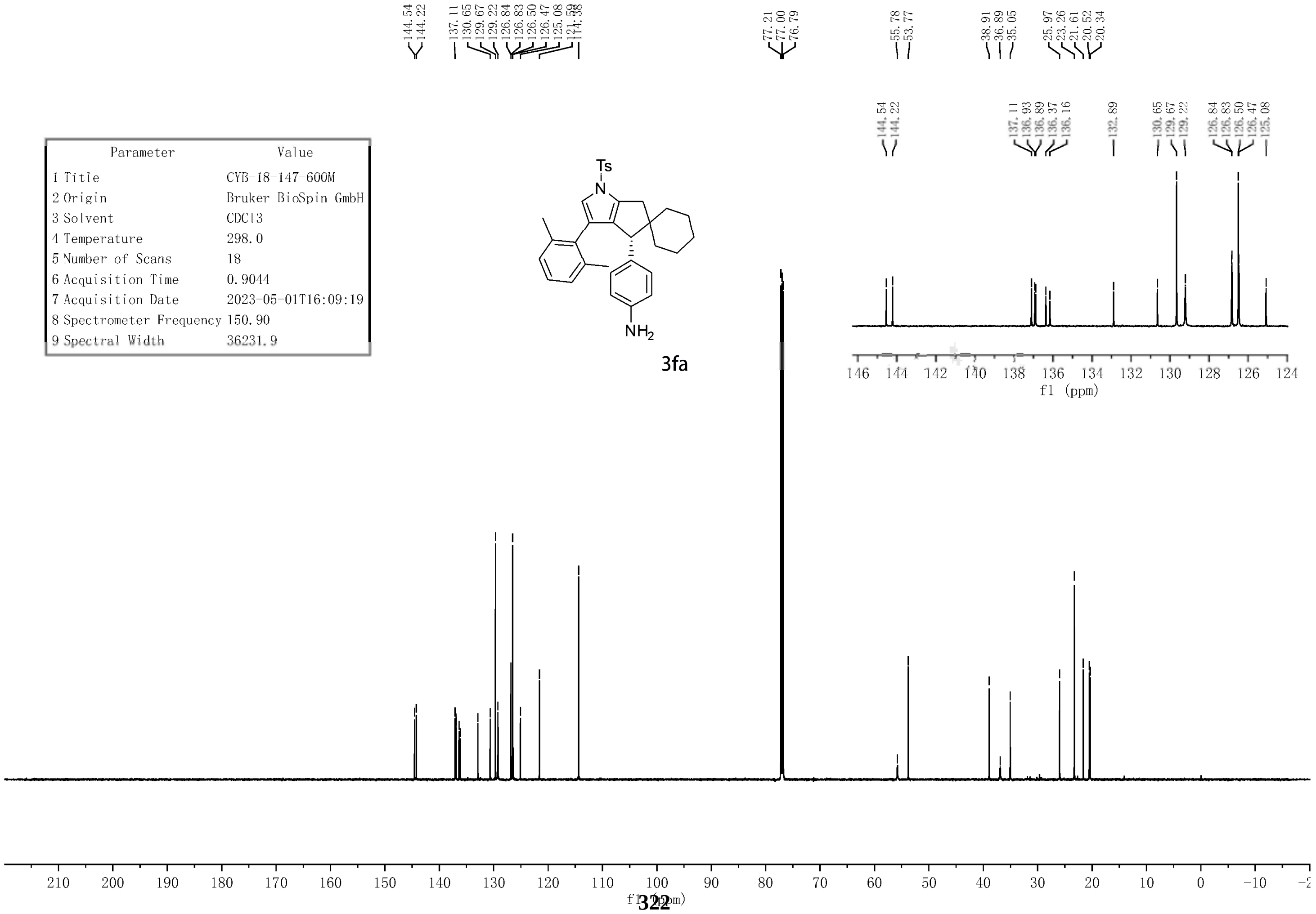

Supplementary Figure 222. ^{1}H and ^{13}C NMR spectra for 3fa

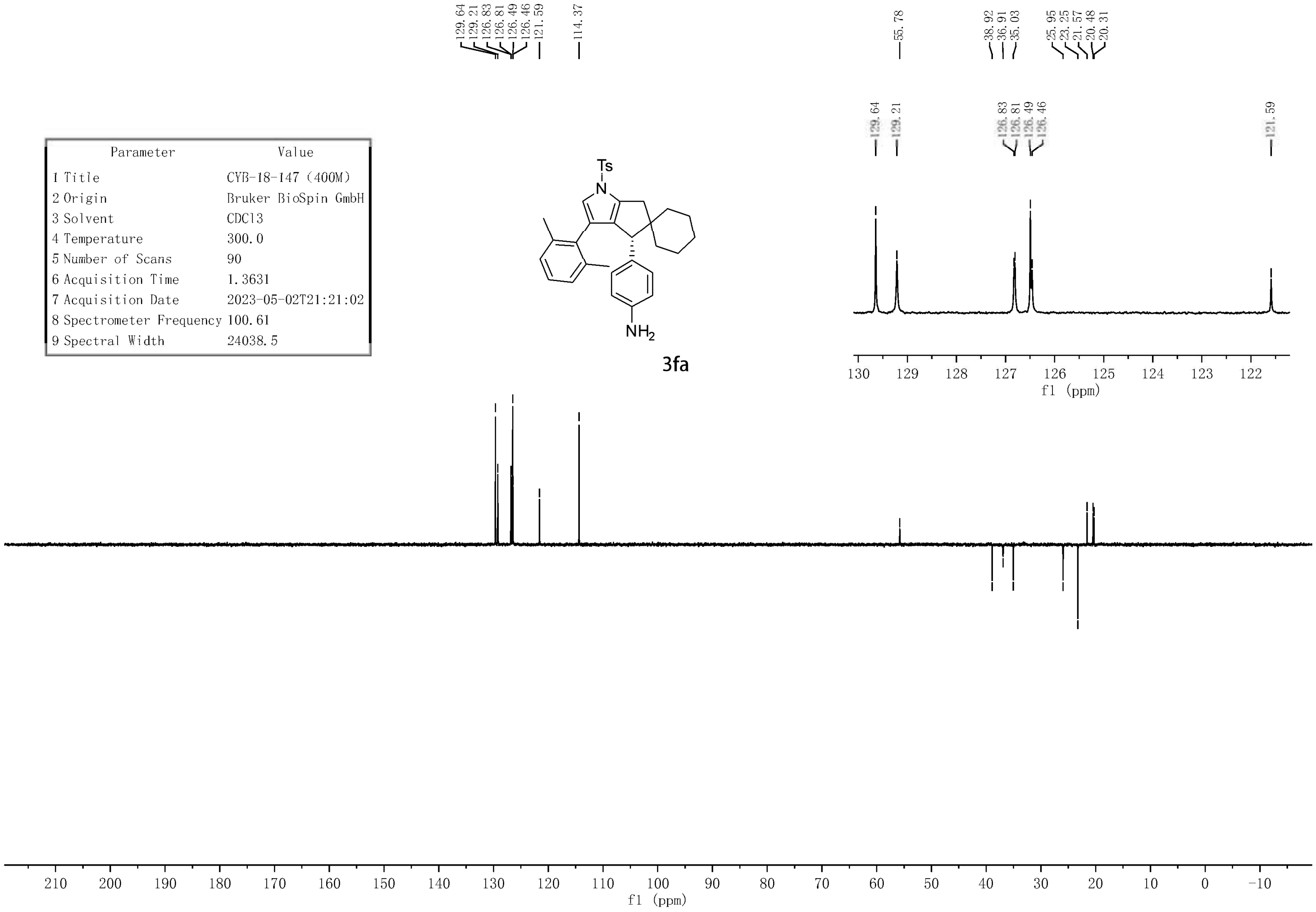

Supplementary Figure 223. DEPT-135 NMR spectra for 3fa

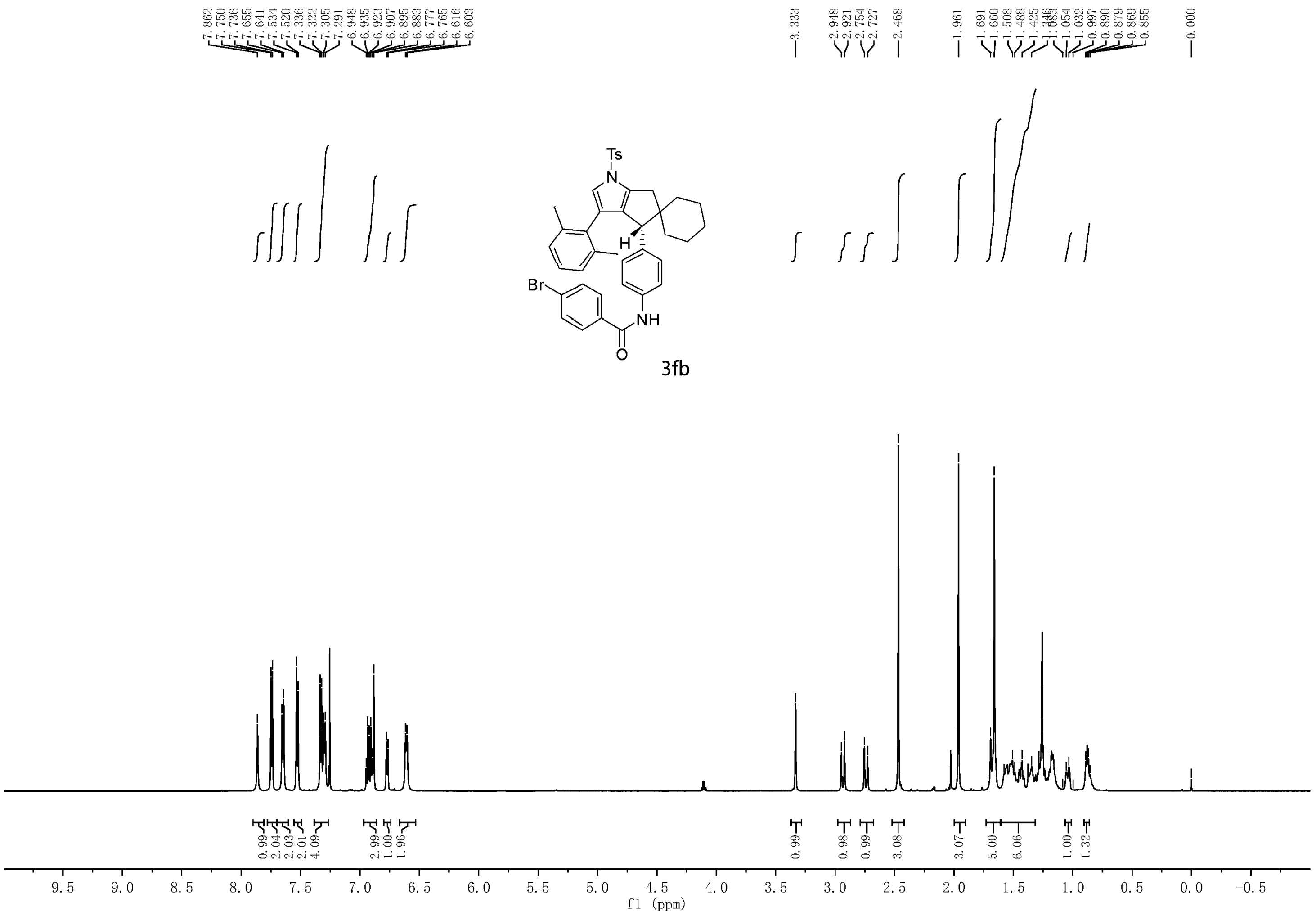

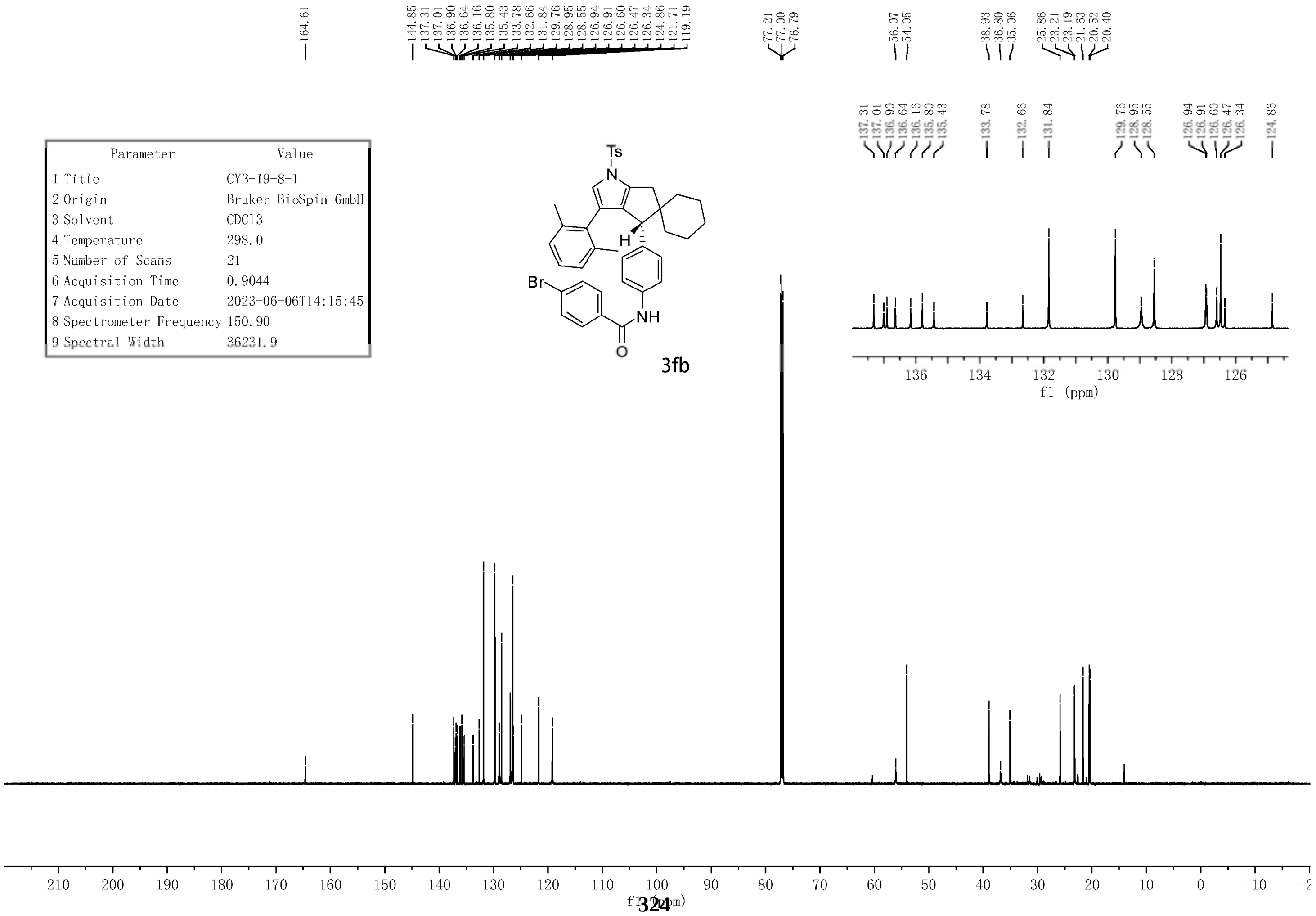

Parameter	Value
1 Title	CYB-19-8-1
2 Origin	Bruker BioSpin GmbH
3 Solvent	CDCl3
4 Temperature	298.0
5 Number of Scans	21
6 Acquisition Time	0.9044
7 Acquisition Date	2023-06-06T14:15:45
8 Spectrometer Frequency	150.90
9 Spectral Width	36231.9

Supplementary Figure 224. ^{1}H and ^{13}C NMR spectra for 3fb

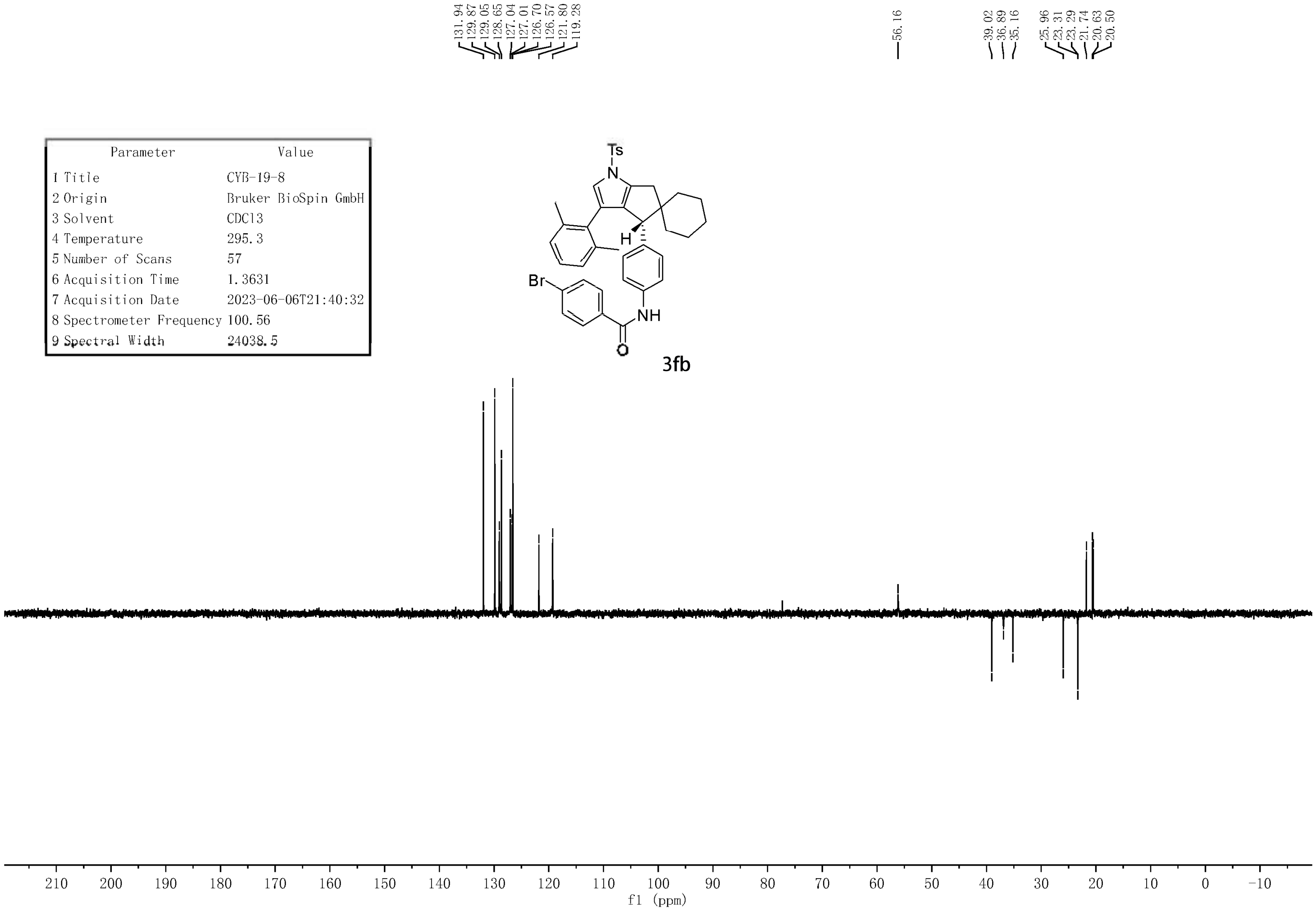

Supplementary Figure 225. DEPT-135 NMR spectra for 3fb

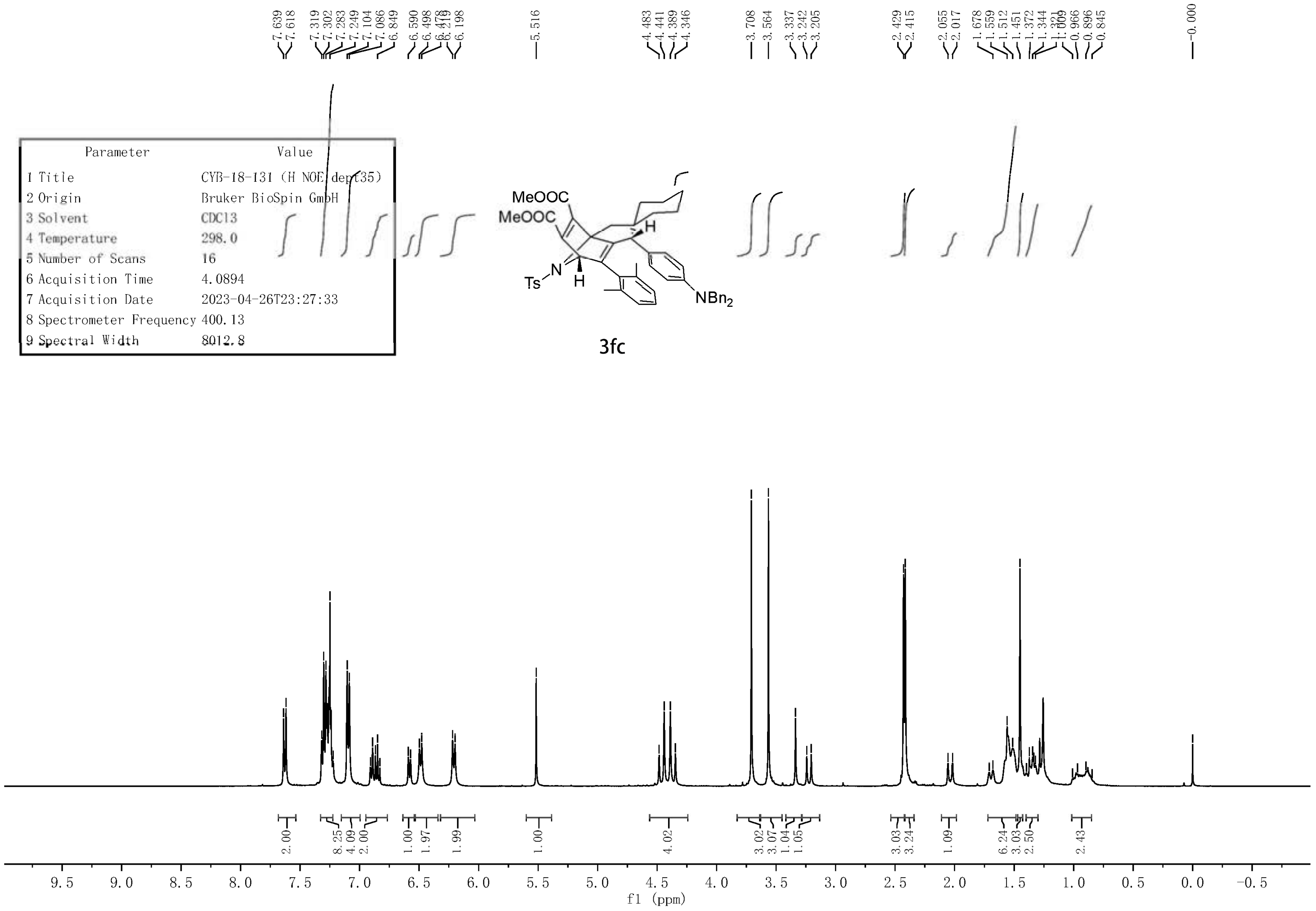

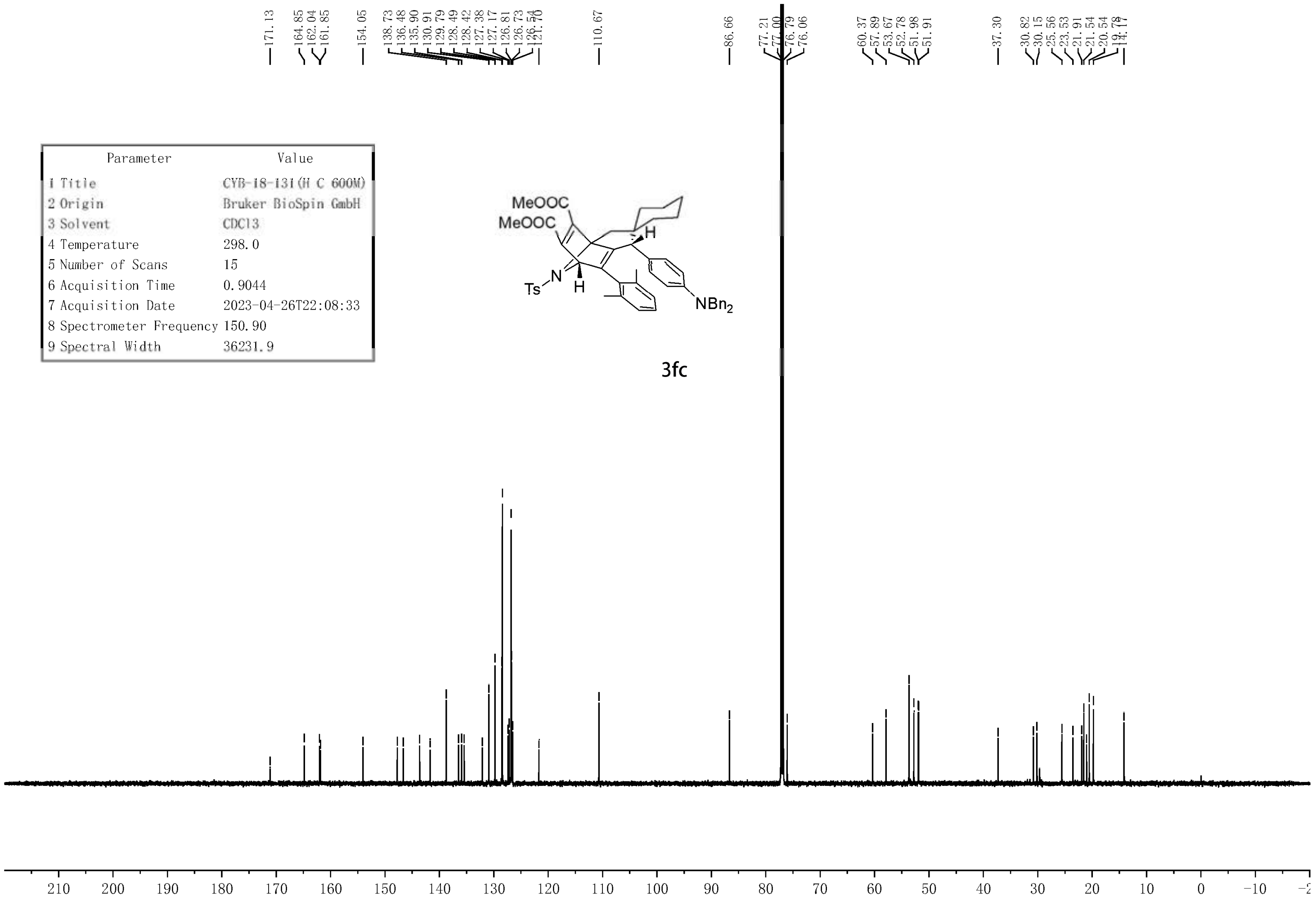

Supplementary Figure 226. ^{1}H and ^{13}C NMR spectra for 3fc

Parameter	Value
1 Title	CYB-18-131 (H NOE dept35)
2 Origin	Bruker BioSpin GmbH
3 Solvent	CDCl3
4 Temperature	300.0
5 Number of Scans	256
6 Acquisition Time	1.3631
7 Acquisition Date	2023-04-27T05:21:41
8 Spectrometer Frequency	100.62
9 Spectral Width	24038.5

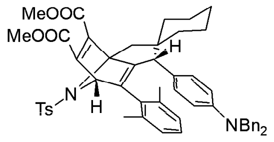

3fc

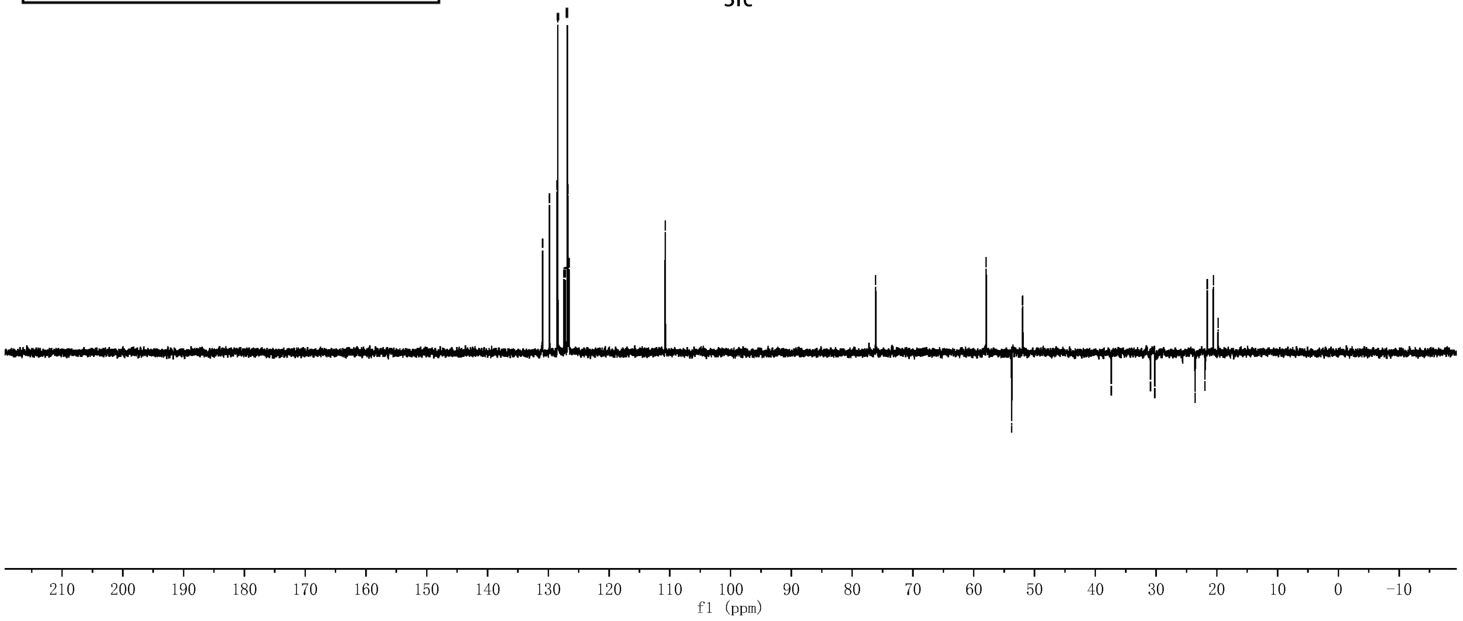

Supplementary Figure 227. DEPT-135 NMR spectra for 3fc

10. References

1. Meden, A. et al. Tryptophan-derived butyrylcholinesterase inhibitors as promising leads against Alzheimer's disease. *Chem. Commun.* **55**, 3765−3768 (2019).
2. Hong. F.-L. et al. Generation of donor/donor copper carbenes through copper-catalyzed diyne cyclization: enantioselective and divergent synthesis of chiral polycyclic pyrroles. *J. Am. Chem. Soc.* **141**, 16961–16970 (2019).
3. Zhu, X.-Q. et al. Copper-catalyzed asymmetric cyclization of alkenyl diynes: method development and new mechanistic insights. *Chem. Sci.* **12**, 9466–9474 (2021).
4. Su, X., Sun, Y., Yao, J., Chen, H. & Chen, C. Acid-promoted bicyclization of arylacetylenes to benzobicyclo[3.2.1]octanes through cationic rearrangements. *Chem. Commun.* **52**, 4537–4540 (2016).
5. Pascalis, L. D., Tekkam, S. & Finn, M.G. Azanorbornadienes as thiol-reactive cleavable linkers. *Org. Lett.* **22**, 6248–6251 (2020).
6. Frisch, M. J. et al. *Gaussian 16, Revision B.01, Gaussian, Inc., Wallingford CT,* ***2016***.
7. Becke, A. D. Density‐functional thermochemistry. III. The role of exact exchange. *J. Chem. Phys.* **98**, 5648–5652 (1993).
8. Lee, C., Yang, W. & Parr, R. G. Development of the Colle–Salvetti correlation-energy formula into a functional of the electron density. *Phys. Rev. B* **37**, 785–789 (1998).
9. Wadt, W. R. & Hay, P. J. Ab initio effective core potentials for molecular calculations. Potentials for main group elements Na to Bi. *J. Chem. Phys.* **82**, 284–298 (1985).
10. Hay, P. J. & Wadt, W. R. Ab initio effective core potentials for molecular calculations. Potentials for the transition metal atoms Sc to Hg. *J. Chem. Phys.* **82**, 270–283 (1985).
11. Woon, D. E. & Dunning, T. H. Jr. Gaussian basis sets for use in correlated molecular calculations. V. Core-valence basis sets for boron through neon. *J. Chem. Phys.* **103**, 4572–4585 (1995).

12. Adamo, C. & Barone, V. Toward reliable density functional methods without adjustable parameters: the PBE0 model. *J. Chem. Phys.* **110**, 6158–6169 (1999).
13. Weigend, F. Accurate Coulomb-fitting basis sets for H to Rn. *Phys. Chem. Chem. Phys.* **8**, 1057–1065 (2006).
14. Marenich, A. V., Cramer, C. J. & Truhlar, D. G. Universal solvation model based on solute electron density and on a continuum model of the solvent defined by the bulk dielectric constant and atomic surface tensions. *J. Phys. Chem. B* **113**, 6378–6396 (2009).
15. W. Humphrey, A. Dalke, K. Schulten, VMD: Visual Molecular Dynamics. *J. Mol. Graph. Model.* **14**, 33–38 (1996).
